# Supplementary material for: Bony-fish-like scales in a Silurian maxillate placoderm
Source: Nat Commun. 2023 Nov 22;14:7622. doi: 10.1038/s41467-023-43557-9 (PMC10665347; doi:10.1038/s41467-023-43557-9)
Supplement: Supplementary file 1 — Supplementary Information [file 41467_2023_43557_MOESM1_ESM.pdf]

**SUPPLEMENTARY INFORMATION**

# Bony-fish-like scales in a Silurian maxillate placoderm

Xindong Cui[1,2], Matt Friedman[3], Yilun Yu[2,4], You-an Zhu[2]*, Min Zhu[2,4]*

1 The Key Laboratory of Orogenic Belts and Crustal Evolution, School of Earth and Space Sciences, Peking University, Beijing 100871, China.

2 CAS Key Laboratory of Vertebrate Evolution and Human Origins, Institute of Vertebrate Paleontology and Paleoanthropology, Chinese Academy of Sciences, Beijing 100044, China.

3 Museum of Paleontology and Department of Earth and Environmental Sciences, University of Michigan, Ann Arbor, MI 48109, USA.

4 University of Chinese Academy of Sciences, Beijing 100049, China.

*Corresponding author: zhumin@ivpp.ac.cn and zhuyouan@ivpp.ac.cn

## Supplementary Notes

### Note 1: Detailed description of the *Entelognathus* scales

**Morphotype 1:** These scales exhibit an isosceles-trapezoid-like shape with a posterior edge that is half the length of the anterior edge. These scales have an average height of 8.22 mm and length of 3.57 mm, resulting in a mean aspect ratio of 2.28 (Supplementary Fig. 3b), the largest among the morphotypes. The dorsal and ventral margins (Supplementary Fig. 4a) are gently concave. The scales have a pronounced indentation in the rear position, dividing the scale into anterior and posterior parts. The anterior part of the crown is smooth, and is overlapped by the posterior dorsolateral plate, whereas the posterior part bears approximately 15 faint and longitudinally arranged ridges (Supplementary Fig. 4$a_1$). The scale base (Supplementary Fig. 4$a_2$) is slightly raised in the center of the scale, and gradually becomes thinner towards the margins.

**Morphotype 2:** They are the deepest type, with an average height of 9.17 mm (Supplementary Fig. 3b). Most scales have a half-moon profile and their mean aspect ratio is 1.6 (Supplementary Fig. 3b). The anterior edge is straight, whereas the dorsal, ventral and posterior edges together form a distorted semicircular arc (Supplementary Fig. 4b). The ventral part of the scales is slightly longer than the dorsal part. In basal view, the scales (Supplementary

Fig. $4b_2$) become thinner towards the periphery, particularly the anterior region.

Some scales proximal to those scales of Morphotype 1 have different profiles. The second lateral line scale (Ll02) (Fig. 4a) has a right triangle outline, and a depressed field along the concave dorsal edge of the crown. This marks an articulation with the ventral ledge of the dorsal adjacent scale. The lateral line groove extends across the crown, terminating in the deep notch on the posterior edge. The outline of three scales (1D01, 1D02, 2V01) (Fig. 4a) is transitional between the scales of Morphotype 1 and Morphotype 2. They are distinctly longer than Morphotype 1, but have a shorter straight posterior margin and straight dorsal and ventral edges.

**Morphotype 3:** These scales are similar to Morphotype 2 scales, but with a smaller average height (7.58 mm) and aspect ratio (1.26) (Supplementary Fig. 3b). The dorsal margin is convex and bears a dorsal process (Supplementary Fig. 4c). The ventral margin is slightly longer and more rounded than the dorsal one. The anterior margin is straight, and the posterior margin is convex. There are about ten anteroposteriorly oriented ridges in the middle and posterior parts of the scale crown (Supplementary Fig. $4c_1$). The ornamented field is 1.5 times as long as the smooth concealed field. The scale base (Supplementary Fig. $4c_2$) is flat, with slightly depressed margins covering adjacent scales.

**Morphotype 4:** These scales are rhombic, with an average length of 5.77 mm and height of 5.36 mm (Supplementary Fig. 3b). They are slightly taller than long, with an average aspect ratio of 1.08 (Supplementary Fig. 3b). The anterior edge has a bulge in the middle part, dividing it into anterodorsal and anteroventral parts that are gently convex (Supplementary Fig. 4$d_1$). The posterodorsal and posteroventral edges are slightly concave. They extend posteriorly and converge to form a short and rounded posterior edge instead of a sharp tip. In crown view, the ornamented field bears more than ten ridges and is about three times as long as the smooth concealed field (Supplementary Fig. 4$d_1$). The scale base (Supplementary Fig. 4$d_2$) is concave, and its marginal depressions are deeper than those of Morphotype 3 scales.

**Morphotype 5:** These scales, measuring 2.62 mm in mean height and 3.48 mm in mean length, are much smaller than other flank scales but proportionally thicker (Supplementary Fig. 3b). They have an almost trapezoidal outline with an average aspect ratio of 0.73 (Supplementary Fig. 3b). The anterior edge is the shortest and is gently convex (Supplementary Fig. 4e), while the dorsal margin is straight or convex. The convex ventral ledge is the longest, forming a posterior tip with the posterior edge. In crown view (Supplementary Fig. 4$e_1$), the ornamented ridges are slenderer than those of other flank scales. The smooth concealed field is present only along the anterior and dorsal edges. A shallow groove extends between the concealed field and ornamented crown.

In basal view, the dorsal and posterior parts (Supplementary Fig. 4$e_2$) have deep depressions that accommodate the concealed fields of the adjacent scales.

**Morphotype 6** consists of five scales. The first scale (2D01) (Fig. 2a) is large, 10.97 mm in height and 10.21 mm in length. It has a long dorsal margin, and becomes shorter ventrally, ending with a short, convex ventral margin. The scale has a convex and thin posterior edge, plus a concave and thick anterior edge. The scale crown (Fig. 2$a_1$) is ornamented by about 20 vermiform ridges that become slender posteriorly and ventrally. The smooth concealed field of the scale crown is depressed and half as long as the ornamented field (Fig. 2$a_1$). Between them, there is a concave triangular area with weak ridges. The dorsal part (Fig. 2$a_1$) of the concealed field is triangular and extends anterodorsally like the peg of early osteichthyan rhomboid scales. Moreover, the anterodorsal part (Fig. 2$a_1$) of the concealed field extends in a manner similar to the anterodorsal process of the osteichthyan scales. Between these two protruding processes, there is a deep triangular depression that articulates with the first post median dorsal scale. In basal view, the scale base (Fig. 2$a_2$) is gently concave.

The second scale (2D04) (Fig. 2b) is rhomboid, close to 2D01 in height (10.65 mm) but shorter in length (6.82 mm). Compared with 2D01, this scale

possesses roughly parallel anterior and posterior edges, and a less pronounced peg-and-socket articulation. The triangular peg-like dorsal concealed field (Fig. $2b_1$) extends dorsally, giving it a very osteichthyan-like appearance. In crown view (Fig. $2b_1$), the dorsal edge is concave and the ventral edge is convex. The ornamented field is twice as long as the concealed field. The scale base (Fig. $2b_2$) has concave ventral and posterior parts.

Other scales (3D02, 3D03, 3D04) (Fig. 2c–e) of Morphotype 6 are smaller than the first two but also have a rhomboid shape. The scale crowns bear 10–15 prominent ridges. Their base thickens in the middle, forming a vertical ridge, like the basal keel of the early osteichthyan rhomboid scales. In 3D02 and 3D03 (Fig. 2c, d), the dorsal and anterodorsal concealed fields are weaker and beak-shaped. Unlike 2D01 and 2D04, their depressions between the two processes lie on the basal side rather than the crown side. In basal view (Fig. $2c_2$, $d_2$), the ventral portion has a pronounced depression accommodating the concealed field of the more ventral scale. Remarkably, 3D03 stretches out a well-developed lingulate process (Fig. 2d) ventrally on the depression's dorsal margin, inserting into the dorsal depression of the ventral adjacent scale, resembling the ventral process of the scales in *Guiyu* and *Psarolepis*[1]. 3D04 (Fig. 2e) possesses the lowest aspect ratio (1.25), but its peg-and-socket, anterodorsal process, and keel-like structure are shared with other Morphotype 6 scales.

**Morphotype 7:** These fan-shaped scales (average height: 4.70 mm; average length: 5.63 mm) are absolutely thicker than the other types (Supplementary Fig. 3b), and come from near the dorsal and ventral midlines (Fig. 4b). The former (Supplementary Fig. 4f) have a straight dorsal margin and a convex ventral margin, whereas the latter (Supplementary Fig. 4g) have a convex dorsal margin and an almost straight ventral margin. The ornamented field bears about 13 ridges. The smooth concealed field is very narrow, only one-fifth of that of the ornamented field (Supplementary Fig. 4$f_1$, $g_1$). The concealed field extends along the dorsal and anterior margins and thins towards the margins. In basal view (Supplementary Fig. 4$f_2$, $g_2$), there is a broad shallow longitudinal groove in the middle part of the scale. The dorsal margin is very thick, forming a strong ridge. The ventral part of the base is depressed.

**Morphotype 8:** They have a large aspect ratio (1.77), exceeded only by that of Morphotype 1 scales (Supplementary Fig. 3b). Their anterior edge is the longest and straight, and the ventral edge is straight or slightly convex but shortest, forming an angle of ninety degrees between them (Supplementary Fig. 4h). The dorsal and posterior edges are gently convex. As the Morphotype 1 scales, these scales (Supplementary Fig. 4$h_1$) are divided into the anterior part which is thick and unornamented plus the posterior part which is thin and ornamented by ridges. The base (Supplementary Fig. 4$h_2$) of the scales is

smooth.

**Morphotype 9**: These scales resemble Morphotype 3 scales, but they are approximately square in shape (average aspect ratio: 1.10) and slightly smaller (average height: 6.27 mm; average length: 5.67 mm) (Supplementary Figs. 3b and 4i). The edges of these scales tend to be straighter compared with Morphotype 3 scales.

**Morphotype 10:** These scales are unique in having a rhomboid profile and their longest axis is clearly oblique to the longitudinal axis of the fish (Supplementary Fig. 4j), resembling the rhomboid scales of early osteichthyans. They also articulate with the ventral adjacent scales in a manner similar to osteichthyan rhomboid scales, with a concave area at the ventral part of the base for articulating with the dorsal depression of the adjacent scales. Their base (Supplementary Fig. $4j_2$) bears a weak vertical keel-like structure in the middle that is consistent with the basal keel of osteichthyan rhomboid scales.

**Morphotype 11:** These scales are semi-elliptic in shape, with their convex dorsal margin being the shortest, and the longer ventral margin being M-shaped (Supplementary Fig. 4k). The anterior and posterior edges are slightly convex. Their ornamentations on the crown are faint as they are covered by the posterior ventral plate (Supplementary Fig. $4k_1$).

**Morphotype 12:** These are the smallest scales (average height: 1.15 mm; average length: 2.54 mm) (Supplementary Fig. 3b), and locate on the tail and fins (Fig. 4a, b). They are teardrop-shaped or lanceolate, with the smallest average aspect ratio of 0.47 (Supplementary Fig. 3b). They become slender posteriorly, forming a sharp terminal point. In crown view (Supplementary Fig. $4l_1$), the dorsal concealed field is deep and well-developed, but the anterior concealed field is narrow. Most of the crown surface is ornamented by four or fewer long ridges. In basal view (Supplementary Fig. $4l_2$), the anterior part of the base is bulged and becomes thinner posteriorly. Some scales (Supplementary Fig. 4m) are strikingly tenuous, resembling willow leaves in shape, and may be part of the caudal fin *in vivo*.

**Lateral line scales:** The first scale (Ll01) (Fig. 4a) in the lateral line series bears a shallow groove but lacks a notch. The second lateral line scale (Ll02) (Fig. 4a) possesses a twisted shallow groove and a short notch. The remaining lateral line scales have a straight groove respectively (Fig. 4a; Supplementary Fig. 4n–p). The notch (Fig. 4a) is short on the third lateral line scale (Ll03), gradually becoming longer on the following scales, reaching a maximum length on the twelfth scale (Ll12), then becoming shorter and finally disappears on the 27$^{th}$ scale (Ll27) on the caudal fin lobe.

**Special scales:** Four scales (2D13, 3V04, 6V05, 6V08) were excluded from the above morphotypes due to their unique contours (Supplementary Fig. 4q–t).

2D13 (Supplementary Fig. 4q) is located behind the basal plate of the anterior dorsal fin, and has a distinct "Thracian-helmet-like" shape with a high and forward-inclined apex. This scale has a conspicuous lingulate anterodorsal horn which may be associated with the first dorsal fin, filling in the gap created by the basal plate of the anterior dorsal fin. The anterior edge is straight and bends back near the ventral third. The dorsal, posterior and ventral ledges join together to form a semicircular arch. The ornamented field is as long as the smooth concealed field. In basal view, the dorsal part of the scale is thicker than other parts. There is a pronounced depression along the dorsal margin.

3V04 (Supplementary Fig. 4r) has a round rectangle shape (6.44 mm in height and 7.30 mm in length). The crown has an extensive smooth concealed field on the anterior, dorsal, and ventral parts, as well as a reduced fan-shaped free field with about ten radial ridges. The base is flat with shallow depressions on the posterior, dorsal, and ventral parts.

6V05 (Supplementary Fig. 4s) is small (2.73 mm in height and 4.33 mm in length), with a low pentagon shape (aspect ratio of 0.63). The dorsal part of the

scale is triangular and has an outstanding bulge followed by a triangle depression. The crown bears faint ridged ornamentations. In basal view, the posterodorsal edge of the scale is thick and swells correspondingly with the depression on the crown.

6V08 (Supplementary Fig. 4t) is shaped in long oval and resembles the Morphotype 12 scales, but is quite large (3.58 mm in height and 9.29 mm in length). The dorsal and ventral edges are long and gently convex, whereas the anterior and posterior edges are short and remarkably convex. The angle between the longest axis of the scale and the longitudinal axis of the fish is about 27°. The concealed field is 42.3% of the scale length. The dorsal and ventral edges slightly shrink at the junction of the concealed and free fields of the crown. In basal view, the dorsal half part of the scale base is depressed.

**Note 2: Post median dorsal plates/scales**

The second post median dorsal scale (Fig. 2g) measures 11.55 mm in length and 0.63 mm in width. Its anterior and posterior lateral edges are slightly concave. The anterior lateral edges are long and gently convex, and the posterior edge is short and convex. Compared to the first post median dorsal scale, its concealed field is short and a bit depressed, and the free field on the crown possesses 20 ridges. A shallow groove (Fig. $2g_1$) separates the two fields. The base (Fig. $2g_{2,3}$) is similar to that of the first scale, except that the lingulate

humps are wider and bifurcated, articulated with 3D02 and 3D03.

The third one (Supplementary Fig. 3h) roughly has an oval shape. The concealed field is extremely shallow, and the free field bears faint ridges. As a result, the crown of the scale is comparatively smooth. In basal view, its lingulate humps are similar in morphology to those of the second scale and articulate with 3D04.

**Note 3: Detailed description of the squamation**

The median dorsal belt (mdb, Fig. 4b) comprises only one area, located between the median dorsal plate and the dorsal fin.

The dorsal belt contains the anterior (db.a, Fig. 4b) and posterior (db.p, Fig. 4b) areas. The dorsal belt includes the Morphotype 6 scales that are articulated with the post median dorsal scales. They have peg-and-socket and anterodorsal process articulations similar to the typical rhomboid scales of early osteichthyans. The posterior area of dorsal belt contains five pairs of Morphotype 7 scales meeting at the median dorsal line. They have a fan-shaped outline and are much thicker than the scales immediately ventrally to them, especially in the dorsal margin.

The middle belt consists of five areas: anterior (mb.a, Fig. 4b), middle (mb.m,

Fig. 4b), posterior (mb.p, Fig. 4b), precaudal (mb.pc, Fig. 4b), and caudal (mb.c, Fig. 4b) areas of middle belt. The anterior area of middle belt corresponds to Morphotype 2 scales. They are arranged in four rows, increasing ventrally from three to eight scales in each row. Their height and aspect ratio are significantly larger than those of the surrounding scales (Fig. 4d, f). The middle area of middle belt has the largest number of scales (Morphotype 3). They are located posterior and ventral to the anterior area of dorsal belt and anterior area of middle belt, plus three pairs of scales (2D14–16) lying anteriorly to the posterior area of dorsal belt. A special scale (2D13) with a protruding anterodorsal corner is quite distinct from the other scales in this area. Posterior to the middle area of middle belt is the posterior area of middle belt containing 3 rows of Morphotype 4 scales (1D16–22, LI15–23, 1V17–19). The precaudal area of middle belt (Morphotype 5) is located immediately posterior and ventral to the posterior area of middle belt, and the scales from the former are significantly lower than those from the latter, with a much smaller aspect ratio (Fig. 4f). The terminal area is the caudal area of middle belt comprising small elongate scales (Morphotype 12).

The ventral belt displays the largest disparity of scales among the squamous portion, with anterior (vb.a, Fig. 4b), dorsal (vb.d, Fig. 4b), middle (vb.m, Fig. 4b), posterior (vb.p, Fig. 4b), and ventral (vb.v, Fig. 4b) areas of ventral belt. The anterior area of ventral belt extends dorsoventrally, containing slim scales

of morphotypes 1, 8, 10, and 11. Posterior to that are the dorsal area of ventral belt (Morphotype 9) and the middle area of ventral belt (Morphotype 10). A branch of the middle area of ventral belt with three scales and an eccentric scale (3V04) invades the anterior part of the two areas. The posterior area of ventral belt possesses three pairs of Morphotype 7 scales that are thick. The ventral area of ventral belt is located around the pelvic fins and the median ventral line. This complex area is mosaic with scales of morphotypes 1, 4, 8–11 and two unusual scales (6V05, 6V08).

**Note 4: Taxon list**

*Acanthodes bronni*[2-8]

*Achoania jarviki*[9-11]

*Acronemus tuberculatus*[12]

*Akmonistion zangerli*[13-15]

*Andreolepis hedei*[16]

*Arabosteus variabilis*[17,18]

*Asterolepis ornata*[19-21]

*Austroptyctodus gardineri*[22]

*Bothriolepis* spp.[23]

*Brachyacanthus scutiger*[8]

*Brindabellaspis stensioi*[24]

*Brochoadmones milesi*[25]

*Buchanosteus confertituberculatus*[26,27]

*Campbellodus decipiens*[22]

*Cassidiceps vermiculatus*[28]

*Cephalaspis lyelli*[29,30]

*Cheiracanthus* spp.[31]

*Cheirolepis canadensis*[32,33]

*Cheirolepis trailli*[33,35]

*Chimaeroidei*[36]

*Chondrenchelys problematica*[37-39]

*Chuchinolepis dongmoensis*[40]

*Cladodoides wildungensis*[41]

*Cladoselache kepleri*[42-44]

*Climatius reticulatus*[6,8,45]

*Cobelodus aculeatus*[46]

*Coccosteus cuspidatus*[47]

*Compagopiscis croucheri*[48]

*Cowralepis mclachlani*[49]

*Culmacanthus stewarti*[50,51]

*Damocles serratus*[52,53]

*Debeerius ellefseni*[54]

*Diabolepis speratus*[55,56]

*Diandongpetalichthys liaojiaoshanensis*[57]

*Dialipina salgueiroensis*[58,59]

*Dicksonosteus arcticus*[60,61]

*Diplacanthus crassisimus*[6,8,62]

*Diplocercides* spp.[63]

*Diplodoselache woodi*[64]

*Dipterus* spp.[65,66]

*Dobunnacanthus waynensis*[6,67]

*Doliodus latispinosus*[68-70]

*Dwykaselachus oosthuizeni*[71]

*Eastmanosteus calliaspis*[72]

*Egertonodus basanus*[73]

*Ellopetalichthys scheii*[74]

*Entelognathus primordialis*[75]

*Epipetalichthys wildungensis*[76,77]

*Eurycaraspis incilis*[78]

*Eusthenopteron foordi*[79,80]

*Euthacanthus macnicoli*[6,8,81]

*Falcatus falcatus*[82]

*Gavinia syntrips*[83]

*Gavinaspis convergens*[84]

*Gemuendina stuertzi*[85]

*Gladbachus adentatus*[86]

*Glyptolepis groenlandica*[87,88]

*Gogonasus andrewsae*[88-92]

*Groenlandaspis antarcticus*[93,94]

*Guangxipetalichthys tiaomajianensis*[95]

*Guiyu oneiros*[1,96,97]

*Gyracanthides murrayi*[98]

*Halimacanthodes ahlbergi*[99]

*Hamiltonichthys mapesi*[100]

*Helodus simplex*[101]

*Holonema westolli*[102]

*Homalacanthus concinnus*[103]

*Homalodontus aplopagus*[104,105]

*Howqualepis rostridens*[106]

*Incisoscutum ritchiei*[107,108]

*Iniopera richardsoni*[109]

*Ischnacanthus gracilis*[6,8,110]

*Jagorina pandora*[111,112]

*Janusiscus schultzei*[113]

*Kansasiella eatoni*[114]

*Kathemacanthus rosulentus*[28,115]

*Kawichthys moodiei*[116]

*Kenichthys campbelli*[117,118]

*Kentuckia deani*[119,120]

*Kimaspis tienshanica*[121]

*Kolymaspis sibirica*[122]

*Kosoraspis peckai*[123,124]

*Kujdanowiaspis podolica*[125]

*Latviacanthus ventspilsensis*[126]

*Lawrenciella schaefferi*[127]

*Ligulalepis toombsi*[58,128-131]

*Lophosteus superbus*[132,133]

*Lunaspis broili*[134]

*Lupopsyrus pygmaeus*[135]

*Macropetalichthys rapheidolabis*[111,136,137]

*Materpiscis attenboroughi*[138,139]

*Meemannia eos*[140,141]

*Mesacanthus mitchelli*[6,8,142]

*Microbrachius dicki*[143,144]

*Miguashaia bureaui*[63,145]

*Mimipiscis toombsi*[119,146,147]

*Minicrania lirouyii*[148]

*Moythomasia durgaringa*[146]

*Mulgaspis evansorum*[149,150]

*Nerepisacanthus denisoni*[151]

*Notopetalichthys hillsi*[152,153]

*Obtusacanthus corroconis*[154]

*Onychodus jandemarrai*[155]

*Onychoselache traquari*[156,157]

*Orthacanthus*[158]

*Osorioichthys marginis*[159]

*Osteolepis macrolepidotus*[79,80,160,161]

*Ozarcus mapesae*[162]

*Pampetalichthys longhuaensis*[163,164]

*Parabuchanosteus murrumbidgeensis*[165,166]

*Parayunnanolepis xitunensis*[167,168]

*Parexus recurvus*[6,8,169]

*Paucicanthus vanelsti*[170]

*Pauropetalichthys magnoculus*[171]

*Porolepis* spp.[88,172]

*Powichthys thorsteinssoni*[173]

*Promesacanthus eppleri*[174]

*Psarolepis romeri*[9,10,175,176]

*Pterichthyodes milleri*[143]

*Ptomacanthus anglicus*[177,178]

*Ptyctolepis brachynotus*[179]

*Pucapampella rodrigae*[180]

*Qilinyu rostrata*[181]

*Qingmenodus yui*[182,183]

*Quasipetalichthys haikouensis*[78]

*Radotina kosorensis*[184]

*Radotina tesselata*[184,185]

*Ramirosuarezia boliviana*[186]

*Raynerius splendens*[187]

*Remigolepis* spp.[188]

*Rhadinacanthus longispinus*[62]

*Rhamphodopsis threiplandi*[22,189]

*Romundina stellina*[190,191]

*Shearsbyaspis oepiki*[74,192]

*Shenacanthus vermiformis*[193]

*Sigaspis lepidophora*[194]

*Sinolepis macrocephala*[195,196]

*Sinopetalichthys kueiyangensis*[197]

*Sparalepis tingi*[198]

*Squalus* spp.[199]

*Styloichthys changae*[200,201]

*Sudaspis chlupaci*[184]

*Synechodus dubrisiensis*[202]

*Tamiobatis vetustus*[44,203]

*Tetanopsyrus lindoei/breviacanthias*[204,205]

*Tlamaspis inopinatus*[184]

*Tribodus limae*[206-209]

*Triodus moorei*[210,211]

*Tristychius arcuatus*[156,212]

*Uraniacanthus curtus*[213]

*Uraniacanthus probaton*[213,214]

*Uranolophus wyomingensis*[215]

*Widjeaspis warrooensis*[216,217]

*Wuttagoonaspis fletcheri*[218]

*Xiushanosteus mirabilis*[193]

*Youngolepis praecursor*[219-221]

*Yunnanolepis* spp.[222,223]

*Zemlyacanthus menneri*[224,225]

**Note 5: Character list**

Skeletal tissues

1. Tessellate prismatic calcified cartilage: (0) absent; (1) present.

2. Prismatic calcified cartilage: (0) single layered; (1) multi-layered.

3. Extensive calcified cartilage: (0) absent; (1) present.

4. Perichondral bone: (0) present; (1) absent.

5. Extensive endochondral ossification: (0) absent; (1) present.

6. Three-layered exoskeleton: (0) absent; (1) present.

7. Cephalic dermoskeletal bone: (0) cellular; (1) acellular.

8. Perforated horizontal lamina in the sensory line canals and vascular system: (0) absent; (1) present.

9. Superficial glassy layer of dermal armour: (0) absent; (1) present.

10. Dentinous tissue: (0) absent; (1) present.

11. Dentine kind: (0) mesodentine; (1) semidentine; (2) orthodentine.

12. Plicidentine: (0) absent; (1) simple or generalized polyplacodont.

13. Enamel(oid) present on dermal bones and scales: (0) absent; (1) present.

14. Enamel: (0) single-layered; (1) multi-layered.

15. Enamel layers: (0) applied directly to one another (ganoine); (1) separated by layers of dentine.

16. Enamel(oid) on teeth: (0) absent; (1) present.

17. Cap of enameloid restricted to upper part of teeth (acrodin): (0) absent; (1) present.

18. Galeaspidin: (0) absent; (1) present.

19. Extensive pore canal network: (0) absent; (1) present.

20. Resorption and redeposition of odontodes: (0) lacking or partially developed; (1) developed.

21. Generations of odontodes: (0) buried; (1) areally growing; (2) resorbed.

22. Enamel and pore canals: (0) enamel absent from inner surface of pores; (1) enamel lines portions of pore canal.

23. Relative size of cosmine pores: (0) small; (1) large.

24. Rostral tubuli: (0) absent; (1) present.

25. Bone cell lacunae in body scale bases: (0) present; (1) absent.

26. Main dentinous tissue forming fin spine: (0) osteodentine; (1) orthodentine.

Squamation & related structures

27. Lepidotrichia or lepidotrichia-like scale alignment: (0) present; (1) absent.

28. Differentiated lepidotrichia: (0) absent; (1) present.

29. Epichordal lepidotrichia in caudal fin: (0) absent; (1) present.

30. Barbed lepidotrichial segments: (0) absent; (1) present.

31. Fringing fulcra: (0) absent; (1) present.

32. Scute-like ridge scales (basal fulcra): (0) absent; (1) present.

33. Flank scale alignment: (0) vertical rows; (1) oblique rows or hexagonal/rhombic packing; (2) disorganised.

34. Scales: (0) macromeric; (1) micromeric.

The coding of *Halimacanthodes ahlbergi*[99] is changed from 1 to 0.

35. Body scale growth pattern: (0) monodontode (monocuspid); (1) polyodontode (multicuspid).

36. Body scale growth concentric: (0) absent; (1) present.

37. Body scales with peg-and-socket articulation: (0) absent; (1) present.

Chondrichthyan scales without the peg-and-socket articulation are coded as 0 (absent), not unapplicable (-).

The coding of *Entelognathus primordialis* is changed from 0 to 1 based on new data in this work.

The coding of *Gogonasus andrewsae*[91] is changed from 0 to 1.

38. Peg on rhomboid scale: (0) longer than wide (narrow); (1) wider than long (broad).

The coding of *Entelognathus primordialis* is changed from unapplicable to 1 based on new data in present paper.

39. Body scale profile: (0) distinct crown and base demarcated by a constriction (neck); (1) flattened.

40. Body scales with bulging base: (0) absent; (1) present.

The coding of *Climatius reticulatus*[45] is changed from 0 to 1.

41. Body scales with flattened base: (0) absent; (1) present.

The coding of *Parayunnanolepis xitunensis*[226] is changed from 0 to 1.

The coding of *Buchanosteus confertituberculatus*[26] is changed from 0 to 1.

The coding of *Gavinia syntrips*[83] is changed from 0 to 1.

The coding of *Uranolophus wyomingensis*[215] is changed from 0 to 1.

42. Relationship of crown and base of isolated trunk scale: (0) crown fully covering the base; (1) crown sitting on the bony base, with an exposed depressed field overlapped by adjacent scale in articulation.

43. Profile of scales with constriction between crown and base: (0) neck similar in width to crown; (1) neck greatly constricted, resulting in anvil-like shape.

The coding of *Andreolepis hedei*[227] is changed from 1 to 0.

The coding of *Raynerius splendens* is changed from 1 to unapplicable because the scales are devoid necks[187].

The coding of *Brochoadmones milesi*[25] is changed from 0 to 1.

44. Body scales with basal canal or open basal vascular cavity (basal pores in scales): (0) absent; (1) present

45. Neck canal: (0) absent; (1) present.

The coding of *Buchanosteus confertituberculatus*[26] is changed from 0 to 1.

46. Keel of scale: (0) absent; (1) present.

Chondrichthyan scales without the keel are coded as 0 (absent), not unapplicable (-).

47. Posterior ledge (or secondary keel) of scale: (0) absent; (1) weak; (2) developed.

Chondrichthyan scales without the posterior ledge (or secondary keel) are coded as 0 (absent), not unapplicable (-).

The coding of *Glyptolepis groenlandica*[87,88] is changed from unapplicable to

0.

The coding of *Eusthenopteron foordi*[88] is changed from unapplicable to 0.

48. Anteroventral process of scale: (0) absent; (1) present.

Chondrichthyan scales without the anteroventral process are coded as 0 (absent), not unapplicable (-).

49. Ventral process of scale: (0) present; (1) absent.

Chondrichthyan scales without the ventral process are coded as 0 (absent), not unapplicable (-).

50. Anterodorsal process on scale: (0) absent; (1) present.

Chondrichthyan scales without the anterodorsal process are coded as 0 (absent), not unapplicable (-).

The coding of *Entelognathus primordialis* is changed from unapplicable to 1 based on new data.

51. Anterodorsal process and peg: (0) separated; (1) confluent.

52. Sensory line canal of body: (0) passes between or beneath scales; (1) passes over scales and/or is partially enclosed or surrounded by scales; (2) perforates and passes through scales.

53. Sensory line canal of head: (0) passes between or beneath scales; (1) passes over scales and/or is partially enclosed or surrounded by scales; (2) perforates and passes through scales.

54. Longitudinal scale alignment in fin webs: (0) present; (1) absent.

55. Sensory line scales/plates on head: (0) unspecialized; (1) apposed growth;

(2) paralleling canal; (3) semicylindrical C-shaped ring scales.

Cranial dermal skeleton

56. Dermal ornamentation: (0) smooth; (1) parallel, vermiform ridges; (2) concentric ridges; (3) tuberculate.

57. Sensory line network: (0) preserved as open grooves; (1) pass through canals enclosed within dermal bones.

58. Sensory canals/grooves: (0) contained within the thickness of dermal bones; (1) contained in prominent ridges on visceral surface of bone.

59. Dermal skull roof: (0) includes large dermal plates; (1) consists of undifferentiated plates or tesserae; (2) include both large dermal plates and tesserae.

60. Tesserae morphology: (0) large interlocking polygonal plates: (1) microsquamose, not larger than body tesserae.

61. Extent of dermatocranial cover: (0) complete; (1) incomplete (scale-free and elsewhere).

62. Unpaired median skull roofing bone in contact with unpaired plate bearing pineal eminence or foramen: (0) absent; (1) present.

63. Median rostral extension of the headshield: (0) absent; (1) present.

64. Lateral fields: (0) absent; (1) present.

65. Division of lateral fields: (0) absent; (1) divided once; (2) divided twice.

66. Lateral fields extend posterior to pectoral sinus: (0) absent; (1) present.

67. Lateral fields extend onto cornua: (0) absent; (1) present.

68. Median fields: (0) absent; (1) present.

69. Median field separation from pineal plate or foramen: (0) absent; (1) present.

70. Median dorsal opening: (0) absent; (1) present.

71. Cornual extensions: (0) absent; (1) present.

72. Corners: (0) absent; (1) present.

73. Fused scale rows on posterior of headshield: (0) absent; (1) present.

74. Dorsal spinal process of headshield: (0) absent; (1) present.

75. Oralobranchial covering: (0) minute scales; (1) tesserae (2); dermal plates; (3) one or two massive dermal plates.

76. Shape of median dorsal opening: (0) transverse slit-like; (1) oval-like (2); slender longitudinal oval.

77. Spines on corners: (0) absent; (1) present.

78. Headshield enclosed posteriorly behind oralobranchial chamber: (0) no; (1) yes.

79. Enlarged tubercles form symmetrical pattern on posterior part of head shield: (0) absent; (1) present.

80. T-shaped rostral plate: (0) absent; (1) present.

81. Single median element carrying the central, middle and posterior pit line: (0) absent; (1) present.

82. Postnuchal plates: (0) absent; (1) present.

83. Cutaneous sensory organ on suborbital plate: (0) absent; (1) present.

84. Cutaneous sensory organ on postsuborbital plate: (0) absent; (1) present.

85. Cutaneous sensory organ on skull roof posterior to orbits: (0) absent; (1) present.

86. Sclerotic ring incorporated into skull roof: (0) absent; (1) present.

87. Rostrocaudal groove on the inner surface of the premedian plate: (0) absent; (1) present.

88. Preorbital depression: (0) absent; (1) present.

89. Preorbital recess: (0) absent; (1) present.

90. Preorbital recess: (0) restricted to premedian plate; (1) extends onto lateral plates.

91. Submarginal articulation: (0) absent; (1) present.

92. Prelateral plate: (0) absent; (1) present.

93. Posterior descending lamina of skull roof: (0) absent; (1) present.

94. Mesial lamina on the internal surface of marginal plate: (0) absent; (1) present.

95. Nostrils enclosed in dermal skull roof: (0) yes; (1) no.

96. Lacrimal: (0) absent; (1) present.

97. Pineal and rostral: (0) contact; (1) separated.

98. Snout region fragmented into mosaic of small plates: (0) no; (1) yes.

99. B-bone: (0) absent; (1) present.

100. Series of bones lateral to supratemporal (postmarginal plate in placoderms): (0) absent; (1) single bone; (2) two bones.

101. Pore clusters: (0) absent; (1) present.

102. Prerostral plate: (0) absent; (1) present.

103. Interparietal: (0) absent; (1) present.

104. Supratemporal (marginal) in contact with postparietal (central): (0) absent; (1) present.

105. Supratemporal (marginal) contact with nasal (postnasal): (0) absent; (1) present.

106. Quadratojugal: (0) present; (1) absent.

107. Accessory operculum: (0) absent; (1) present.

108. Dermal bone (sarcopterygian postorbital) between jugal (suborbital) and intertemporal (postorbital): (0) absent; (1) present.

109. Lacrimal notch: (0) absent; (1) present.

110. Orbital process of maxilla: (0) absent; (1) present.

111. Dermal cranial joint at level of sphenoid-otic junction: (0) absent; (1) present.

112. Posterior nostril: (0) associated with orbit; (1) not associated with orbit.

113. Posterior nostril: (0) external; (1) palatal.

114. Posterior nostril in external position: (0) far from jaw margin; (1) at or close to jaw margin.

115. Choana: (0) absent; (1) present.

116. Lacrimal posteriorly enclosing posterior nostril: (0) absent; (1) present.

117. Premaxilla contributes to posterior nostril: (0) absent; (1) present.

118. Position of anterior nostril: (0) facial; (1) at oral margin.

119. Number of nasals: (0) many; (1) one or two.

120. Mesial margin of nasal: (0) not notched; (1) notched.

121. Dermintermedial process: (0) absent; (1) present.

122. Extended preorbital region between eyes and nasal capsule: (0) absent; (1) present.

123. Orbit dorsal or facing dorsolaterally: (0) present; (1) absent.

124. Orbits, surrounded laterally by endocranium: (0) absent; (1) partially surrounded; (2) surrounded.

125. Supraorbital (sensu Cloutier and Ahlberg 1996, including posterior tectal of Jarvik): (0) absent; (1) present.

126. Number of supraorbitals: (0) one; (1) two; (2) many.

127. Supraorbital, preorbital and nasal: (0) unfused; (1) fused.

128. Tectal (sensu Cloutier and Ahlberg 1996, not counting the posterior tectal of Jarvik): (0) absent; (1) present.

129. Pineal opening in braincase: (0) absent; (1) present.

130. Pineal opening perforation in dermal skull roof: (0) present; (1) absent.

131. Pineal eminence (in taxa lacking pineal foramen): (0) absent; (1) present.

132. Location of pineal foramen/eminence: (0) level with posterior margin of orbits; (1) well posterior of orbits.

133. Opening in dermal skull roof for spiracular bounded by bones carrying otic canal: (0) absent; (1) present.

134. Dermal plate associated with pineal eminence or foramen: (0) contributes to orbital margin; (1) plate bordered laterally by skull roofing bones.

135. Skull roof with broad supraorbital vaults: (0) absent; (1) present.

136. Parietals (preorbitals of placoderms): (0) absent; (1) present.

137. Condition of parietals/preorbitals: (0) do not meet in midline; (1) meet in midline; (2) single midline bone.

138. Parietals (preorbitals of placoderms) surround pineal foramen or eminence: (0) yes; (1) no.

139. Postparietals (centrals of placoderms): (0) absent; (1) present.

140. Condition of postparietals/centrals: (0) do not meet in midline; (1) meet in midline; (2) single midline bone.

141. Suture between paired skull roofing bones: (0) straight; (1) sinusoidal.

142. Large unpaired median skull roofing bone anterior to the level of nasal capsules (premedian plate): (0) absent; (1) present.

143. Position of premedian plate: (0) dorsal; (1) ventral.

144. Postnasal plate: (0) absent; (1) present.

145. Postmarginal plate: (0) absent; (1) present.

146. Obstantic margin of skull roof: (0) long; (1) short.

147. Large unpaired median bone contributing to posterior margin of skull roof (nuchal plate): (0) absent; (1) present.

148. Nuchal plate: (0) without orbital facets; (1) with orbital facets.

149. Nuchal reaching or almost reaching orbital margin: (0) absent; (1) present.

150. Paired pits on ventral surface of nuchal or median extrascapular plate: (0) absent; (1) present.

151. Contact of nuchal or centronuchal or median extrascapular plate with paired preorbital or parietal plates: (0) absent; (1) present.

152. Number of marginal bones alongside paired median skull roofing bones over the otico-occipital division of braincase: (0) single; (1) two or more.

153. Lateral plate: (0) absent; (1) present.

154. Paranuchal number: (0) one pair; (1) two pairs.

155. Median paranuchal plate: (0) absent; (1) present.

156. Posterior process of the paranuchal plate behind the nuchal plate (dorsal face): (0) absent; (1) present.

157. Medial processes of paranuchal wrapping posterolateral corners of nuchal plate: (0) absent; (1) present; (2) paranuchals precluded from nuchal by central or median paranuchal.

158. Posterior projection on posterior paranuchal plate: (0) absent; (1) present

159. Canal-bearing bone of skull roof extends far past posterior margin of parietals: (0) no; (1) yes.

160. Extratemporal: absent (0), present (1).

161. Westoll-lines: (0) absent; (1) present.

162. Anteriorly directed adductor fossae between neurocranium and skull roof: (0) absent; (1) present.

163. Anterior pit line of dermal skull roof: (0) absent; (1) present.

164. Position of anterior pit-line: (0) on paired median skull roofing bones over the otico-occipital division of braincase; (1) on paired median skull roofing bones over the sphenoid division of braincase.

165. Middle and posterior pit-lines on postparietal: (0) posteriorly situated; (1) mesially situated.

166. Position of middle and posterior pit lines: (0) close to midline; (1) near the central portion of each postparietal.

167. Junction of posterior pitline and main lateral line: (0) far in front of posterior margin of skull roof; (1) close to posterior margin of skull roof.

168. Ethmoid commissure: (0) absent; (1) present.

169. Ethmoid commissure fused into midline canal: (0) absent; (1) present.

170. Course of ethmoid commissure: (0) middle portion through median rostral; (1) sutural course; (2) through bone center of premaxillary.

171. Infraorbital canal follows premaxillary suture: (0) no; (1) yes.

172. Postmarginal canal: (0) absent; (1) present.

173. Postmarginal line issued from main lateral line: (0) on marginal or supratemporal; (1) on anterior paranuchal or tabular.

174. Central sensory line: (0) absent; (1) present.

175. Supraorbital sensory canals: (0) absent; (1) present.

176. Course of supraorbital canal: (0) between anterior and posterior nostrils; (1) anterior to both nostrils.

177. Course of supraorbital canal: (0) straight; (1) lyre-shaped.

178. Posterior end of supraorbital canal: (0) in postparietal (central); (1) in parietal (preorbital); (2) in intertemporal; (3) in nuchal plate; (4) in postpineal plate.

179. Posteriorly converging supraorbital canals: (0) absent; (1) present.

180. Supraorbital canals and posterior pitlines convergence: (0) absent; (1) converge without contact; (2) converge with contact.

181. Contact between otic and supraorbital canals: (0) not in contact; (1) in contact.

182. Contact of supraorbital and infraorbital canals: (0) in contact rostrally; (1) not in contact rostrally.

183. Otic canal: (0) runs through skull roof; (1) follows edge of skull roof.

184. Otic canal extends through postparietals (central): (0) absent; (1) present.

185. Jugal portion of infraorbital canal joins supramaxillary canal: (0) present; (1) absent.

186. Infra-orbital sensory line: (0) crosses lateral field; (1) does not cross lateral field.

187. Festooned pattern of sensory canals: (0) absent; (1) present.

188. Median transverse canals: (0) two or more; (1) one; (2) absent.

189. Multiply branched sensory canal system associated with the posterior end of the supraorbital canal: (0) absent; (1) present.

In the present dataset, the character is only present in *Wenshanaspis*.

190. Branching end of lateral transverse canals: (0) absent; (1) present.

In the present dataset, the character is only present in *Wenshanaspis*.

191. Median dorsal canal: (0) absent; (1) present.

192. Infraorbital and otic sensory line grooves run along mesial margin of marginal plate: (0) no; (1) yes.

193. Semicircular pit line: (0) absent; (1) present.

In the present dataset, the character is only present in *Bothriolepis*.

194. Horizontal sensory line canal on cheek: (0) absent; (1) present.

195. Preopercular canal: (0) absent; (1) present.

196. Preopercular canal meets otic canal: (0) absent; (1) present.

197. Supraoral canal: (0) absent; (1) present.

198. Extension of otic canal beyond infraorbital canal ("P" canal): (0) absent; (1) present.

199. Posterior pitline and postmarginal canal in contact: (0) absent; (1) present.

In the present dataset, the character is only present in *Entelognathus*.

200. Supraorbital canal joins infraorbital canal: (0) anterior to supraoral canal; (1) posterior to supraoral canal.

201. Sensory line commissure across extrascapular bones (nuchal and paranuchal): (0) absent; (1) present.

202. Sensory canal or pit-line associated with maxilla: (0) absent; (1) present.

203. Endolymphatic ducts open in dermal skull roof: (0) present; (1) absent.

204. External endolymphatic duct openings' location in relation to median field: (0) internal; (1) external.

205. Endolymphatic ducts with oblique course through dermal skull bones: (0) absent; (1) present.

206. Endolymphatic duct relationship to median skull roof bone (i.e. nuchal plate): (0) within median bone; (1) on bones flanking the median bone (e.g. paranuchals).

207. Sclerotic ring: (0) absent; (1) present.

208. Number of sclerotic plates: (0) four or less; (1) more than four.

209. Number of extrascapulars: (0) uneven; (1) paired.

210. Number of paired extrascapulars: (0) one pair; (1) two pairs.

211. Consolidated cheek plates: (0) absent; (1) present.

212. Foramina (similar to infradentary foramina) on cheek bones: (0) absent; (1) present.

213. Most posterior major bone of cheek bearing preopercular canal (preopercular) extending forward, close to orbit: (0) absent; (1) present.

214. Number of cheek bones bearing preopercular canal posterior to jugal: (0) one; (1) two.

215. Bone bearing both quadratojugal pit-line and preopercular canal: (0) absent; (1) present.

216. Anterior portion of preopercular canal: (0) present; (1) absent.

217. Vertical canal associated with preopercular/suborbital canal: (0) absent; (1) present.

218. Cheek plate: (0) undivided; (1) divided (i.e., squamosal and preopercular).

219. Subsquamosals in taxa with divided cheek: (0) absent; (1) present.

220. Preopercular shape: (0) rhombic; (1) bar-shaped.

221. Preoperculosubmandibular: (0) absent; (1) present.

222. Dermohyal: (0) absent; (1) present.

Gardiner and Schaeffer (1989, ch.A2) and Coates (1998, ch.A2) defined this character as a dermohyal covering the head of the hyomandibular which notches the supratemporal or the dermosphenotic (Cloutier and Arratia, 2004).

223. Enlarged postorbital tesserae separated from orbital series: (0) absent; (1) present.

224. Bony hyoidean gill-cover series (branchiostegals): (0) absent; (1) present.

225. Branchiostegal plate series along ventral margin of lower jaw: (0) absent; (1) present.

226. Branchiostegal ossifications: (0) plate-like; (1) narrow and ribbon-like.

227. Branchiostegal ossifications: (0) ornamented; (1) unornamented.

228. Imbricated branchiostegal ossifications: (0) absent; (1) present.

229. Opercular flap/gill slits: (0) complete or partial; (1) separate gill covers and gill slits.

230. Opercular (submarginal) ossification: (0) absent; (1) present.

231. Shape of opercular (submarginal) ossification: (0) broad plate that tapers towards its proximal end; (1) narrow, rod-shaped.

232. Ventral lamina of opercular (submarginal) ossification: (0) absent; (1)

present.

233. Ventral lamina of suborbital (jugal): (0) absent; (1) present.

234. Notch in anterior margin of jugal: (0) absent; (1) present

235. Anterodorsal process of opercular (submarginal) ossification attaching onto skull: (0) absent; (1) present.

236. Subopercular ossification: (0) absent; (1) present.

237. Lateral gular plates: (0) absent; (1) present.

238. Size of lateral gular plates: (0) extending most of length of the lower jaw; (1) restricted to the anterior third of the jaw (no longer than the width of three or four branchiostegals.

239. Median gular: (0) present; (1) absent.

Dentition, dermal jaw & tooth-bearing bones

240. Oral dermal tubercles borne on jaw cartilages: (0) absent; (1) present.

241. Oral dermal tubercles patterned in organised rows (teeth): (0) absent; (1) present.

242. Teeth ankylosed to dermal bones: (0) absent; (1) present.

243. Dermal jaw plates on biting surface of jaw cartilages: (0) absent; (1) present.

244. Large dermal plates forming outer dental arcade: (0) only with denticles; (1) with large monolinear tooth row.

245. Dermal plates on mesial (lingual) surfaces of Meckel’s cartilage and

palatoquadrate: (0) absent; (1) present.

246. Gnathal plates mesial to and/or above (or below) jaw cartilage: (0) absent; (1) present.

247. Deep, high supragnathal bone with durophagous occlusal surface: (0) absent; (1) present.

248. Posterior supragnathal with vertical pipe-like ridges: (0) absent; (1) present.

249. Strongly curved infragnathals with wide flat non-biting region: (0) absent; (1) present.

250. Number of fang pairs on ectopterygoid: (0) none; (1) one; (2) two.

251. Enlarged anterior tooth on premaxilla: (0) absent; (1) present.

252. Number of tooth rows on outer dental arcade: (0) single row; (1) two rows, with large teeth lingually and small teeth labially.

253. Number of infradentaries: (0) one; (1) two; (2) more than 2.

254. Number of fang pairs on posterior coronoid: (0) none; (1) one; (2) two.

255. Teeth radial rows on prearticular: (0) absent; (1) present.

256. ’Symplectic’ articulation: (0) absent; (1) present.

257. Processus ascendens of palatoquadrate: (0) absent; (1) present.

258. Grooved, curved upper toothplates attached to median labial element: (0) absent; (1) present.

259. Two divergent processes extending from anterior of palatoquadrate: (0) absent; (1) present.

260. Extramandibular dentition: (0) absent; (1) present.

261. Bilateral series of labial cartilages: (0) absent; (1) present.

262. Maxilla and premaxilla sensu lato (upper gnathal plates lateral to jaw cartilage): (0) absent; (1) present.

263. Maxilla and premaxilla sensu stricto (upper gnathal plates lateral to jaw cartilage without palatal lamina): (0) absent; (1) present.

264. Tooth-bearing median rostral: (0) absent; (1) present.

265. Premaxillae with inturned symphysial processes: (0) absent; (1) present.

266. Premaxilla forming part of orbit: (0) absent; (1) present.

267. Premaxilla: (0) extends under orbit; (1) restricted anterior to orbit.

268. Preorbital process of premaxilla: (0) absent; (1) present.

269. Ventral margin of maxilla: (0) straight; (1) curved.

270. Posterior expansion of maxilla (maxilla cleaver-shaped): (0) present; (1) absent.

271. Contribution by maxilla to posterior margin of cheek: (0) present; (1) absent.

272. Dentary marginal bone of mouth: (0) absent; (1) present.

273. Teeth of dentary: (0) reaching anterior end of dentary; (1) not reaching anterior end.

274. Pair of tooth plates (anterior supragnathals or vomers) on ethmoidal plate: (0) absent; (1) present.

275. Fused anterior supragnathals: (0) absent; (1) present.

276. Vomerine fangs: (0) absent; (1) present.

277. Vomeral area with grooves and raised areas: (0) absent; (1) present.

278. Posterior process of vomers: (0) absent; (1) present.

279. Median dermal bone of palate (parasphenoid): (0) absent; (1) present.

280. Buccohypophysial canal in parasphenoid: (0) single; (1) paired.

281. Ascending process of parasphenoid: (0) absent; (1) present.

282. Shape of parasphenoid denticulated field: (0) broad rhomboid or lozengeshaped; (1) broad, splint-shaped; (2) slender, splint-shaped.

283. Parasphenoid denticulated field with multifid anterior margin: (0) absent; (1) present.

284. Parasphenoid: (0) protruding forward into ethmoid region of endocranium; (1) behind ethmoid region.

285. Posterior of parasphenoid: (0) restricted to ethmosphenoid region; (1) extends to otic region.

286. Denticulated field of parasphenoid: (0) without spiracular groove; (1) with spiracular groove.

287. Parasphenoid denticle field with anteriorly divergent lateral margins: (0) absent; (1) present.

288. Parasphenoid denticle field: (0) terminates at or anterior to level of foramina for internal carotid arteries; (1) extends posterior to foramina for internal carotid arteries.

289. Anterior portion of parasphenoid (pre-buccohypophyseal foramen) of greater length than posterior portion (post-foramen): (0) absent; (1) present.

290. Coronoids: (0) present, (1) absent.

291. Number of coronoids: (0) more than three; (1) three.

292. Fangs of coronoids (sensu stricto): (0) absent; (1) present.

293. Dentition on coronoids: (0) broad marginal ‘tooth field’; (1) narrow or single marginal tooth row.

294. Posterior coronoid: (0) similar to anterior coronoids; (1) forms expanded coronoid process.

295. Infradentary: (0) absent; (1) present.

296. Extent of infradentaries: (0) along much of ventral margin of dentary; (1) restricted to posterior half of dentary.

297. Infradentary foramen and groove: (0) present; (1) absent.

298. Large ventromesially directed flange of symphysial region of mandible: (0) absent; (1) present.

299. Extensive flange composed of prearticular and Meckelian bone that extends beyond ventral edge of outer dermal series: (0) absent; (1) present.

300. Strong ascending flexion of symphysial region of mandible: (0) absent; (1) present.

301. Parasymphysial plate: (0) detachable tooth whorl; (1) long with posterior corner, sutured to coronoid, denticulated or with tooth row; (2) absent.

302. Anterior end of prearticular: (0) far from jaw symphysis; (1) near jaw symphysis.

303. Prearticular - dentary contact: (0) present; (1) absent.

304. Number of dermopalatines: (0) one; (1) two; (2) more than 2.

305. Entopterygoids: (0) separated; (1) contact along midline.

306. Proportions of entopterygoid: (0) anterior end level with processus ascendens; (1) anterior end considerably anterior to processus ascendens.

307. Course of mandibular canal: (0) passing through dentary; (1) not passing through dentary.

308. Pharyngeal teeth or denticles: (0) absent; (1) present.

309. Lingual torus: (0) absent; (1) present.

310. Basolabial shelf: (0) absent; (1) present.

311. Tooth whorls: (0) absent; (1) present.

312. Distribution of tooth whorls: (0) upper and lower jaws; (1) lower jaws only; (2) upper jaws only.

313. Bases of tooth whorls: (0) single, continuous plate; (1) some or all whorls consist of separate tooth units.

314. Enlarged adsymphysial tooth whorl: (0) absent; (1) present.

315. Tooth families/whorls: (0) restricted to symphysial region; (1) distributed along jaw margin.

316. Number of tooth families/whorls per jaw ramus: (0) 15 or fewer; (1) 20 or more.

317. Tooth families/whorls: (0) continuous; (1) discontinuous.

318. Toothplates consolidated into one to three large posterior plates, and one to three smaller anterior tooth plates, occupying each quadrant of the jaw: (0)

absent; (1) present.

319. Toothplate complement restricted to two pairs in the upper jaw and a single pair in the lower jaw: (0) absent; (1) present.

320. Length of dentary: (0) constitutes a majority of jaw length; (1) half the length of jaw or less.

321. Labial pit: (0) absent; (1) present.

322. Prearticular symphysis: (0) absent; (1) present.

323. Retroarticular process: (0) absent; (1) present.

Mandibular arch

324. Mandibular arch: (0) absent; (1) present.

325. Position of mandibular arch articulations: (0) terminal; (1) subterminal.

326. Palatoquadrate relationship to dermal cheek bones: (0) articulation narrow and restricted; (1) broad articulation.

327. Articulation between neurocranium and palatoquadrate posterodorsal to orbit (suprapterygoid articulation): (0) absent; (1) present.

328. Articulation surface of the palatoquadrate with the postorbital (suprapteryoid) process: (0) directed anteriorly; (1) laterally; (2) dorsally.

329. Large otic process of the palatoquadrate: (0) absent; (1) present.

330. Laterally extending palatoquadrate: (0) absent; (1) present.

331. Insertion area for jaw adductor muscles on palatoquadrate: (0) ventral; (1) lateral.

332. Oblique ridge or groove along medial face of palatoquadrate: (0) absent; (1) present.

333. Fenestration of palatoquadrate at basipterygoid articulation: (0) absent; (1) present.

334. Perforate or fenestrate anterodorsal (metapterygoid) portion of palatoquadrate: (0) absent; (1) present.

335. Metapterygoid with developed medial ventral protrusion: (0) absent; (1) present.

336. Autopalatine and quadrate: (0) comineralized; (1) separate mineralizations.

337. Palatoquadrate fused with neurocranium: (0) absent; (1) present.

338. Contact between palatoquadrate and dermal cheek bones: (0) continuous contact of metapterygoid and autopalatine; (1) metapterygoid and autopalatine contacts separated by gap between commissural lamina of palatoquadrate and cheek bones.

339. Position of upper mandibular arch cartilage (and associated cheek plate where present): (0) entirely suborbital; (1) with a postorbital extension.

340. Scalloped oral margin on Meckel’s cartilage and palatoquadrate: (0) absent; (1) present.

341. Mandibular symphysis fused: (0) absent; (1) present.

342. Pronounced dorsal process on Meckelian bone or cartilage: (0) absent; (1) present.

343. Meckelian bone exposed immediately anterior to first coronoid: (0) yes; (1)

no.

344. Preglenoid process: (0) absent; (1) present.

345. Biconcave glenoid on lower jaw: (0) absent; (1) present.

346. Jaw articulation located on rearmost extremity of mandible: (0) absent; (1) present.

Hyoid and gill arches

347. Foramen in hyomandibular: (0) absent; (1) present.

348. Interhyal: (0) absent; (1) present.

349. Hypohyal: (0) absent; (1) present.

350. Disposition of the interbranchial ridges of the oralobranchial chamber roof: (0) oligobranchiate; (1) orthobranchiate; (2) nectaspidoform.

351. Number of branchial fossae: (0) 5-7; (1) 9-17; (2) more than 20.

352. Basibranchial elements: (0) unpaired; (1) paired.

353. Sublingual rod: (0) absent; (1) present.

354. Dense array of hyoid arch rays covers gill area: (0) absent; (1) present.

355. Endoskeletal urohyal: (0) absent; (1) present.

356. Urohyal shape (vertical plate): (0) absent; (1) present.

357. Basihyal: (0) absent; (1) present.

358. Ceratohyal smooth with posterior, lateral fossa: (0) absent; (1) present.

359. Anterior most unpaired element of branchial skeleton contacted by: (0) present; (1) absent.

360. Multiple unpaired branchial mineralisations: (0) absent; (1) present.

361. Posterior two ventral branchial arches:0 separate; (1) articulate ventrally.

362. Posterior two dorsal branchial arches: (0) separate; (1) articulate dorsally.

363. Gill arches: (0) largely restricted to region under braincase; (1) extend far posterior to braincase.

364. Gill skeleton extends posteriorly beyond occiput: (0) absent; (1) present.

365. First branchial arch meets neurocranium: (0) ventral to otic region; (1) posterior to otic region.

366. Separate supra- and infra-pharyngobranchials: (0) absent; (1) present.

367. Pharyngobranchial orientation: (0) directed anteriorly; (1) posteriorly.

368. Posteriormost branchial arch bears epibranchial unit: (0) absent; (1) present.

369. Epibranchials bear posterior flange: (0) absent; (1) present.

370. Hypobranchial orientation: (0) directed anteriorly; (1) hypobranchials of second and more posterior gill arches directed posteriorly.

Neurocranium

371. Endoskeletal intracranial joint: (0) absent; (1) present.

372. Discrete division of the ethmoid and more posterior braincase at the level of the optic tract canal (optic fissue): (0) absent; (1) present.

373. Ventral cranial fissure: (0) absent; (1) present.

374. Metotic (otic-occipital) fissure: (0) absent; (1) present.

375. External nasal opening: (0) single median; (1) paired.

376. Nasal opening(s): (0) dorsal, placed between orbits; (1) ventral and anterior to orbits.

377. Nasohypophyseal opening shape: (0) unconstructed; (1) constriction between nasal and hypophysial divisions; (2) split into nasal and hypophysial divisions.

378. Endoskeletal lamina (postnasal wall) separating posterior nostril and orbit: (0) absent; (1) present.

379. Orbitonasal lamina dorsoventrally deep: (0) absent; (1) present.

380. Size of profundus canal in postnasal wall: (0) small; (1) large.

381. Three large pores (in addition to nostrils) associated with each side of ethmoid: (0) absent; (1) present.

382. Ventral face of nasal capsule in taxa with mineralized ethmoid: (0) complete; (1) fenestra ventrolateralis; (2) entire floor unmineralized.

383. Fenestra ventrolateralis: (0) absent; (1) present; (2) common ventral fenestra for anterior and posterior nostrils.

384. Precerebral fontanelle: (0) absent; (1) present.

385. Olfactory tracts: (0) short, with olfactory capsules situated close to telencephalon cavity; (1) elongate and tubular (much longer than wide).

386. Olfactory tracts: (0) parallel or near-parallel; (1) significantly diverged.

387. Prominent pre-orbital rostral expansion of the neurocranium: (0) present; (1) absent.

388. Ethmoid region elongate with dorsoventrally deep lateral walls: (0) absent; (1) present.

389. Ethmoid articulation for palatoquadrate: (0) placed on postnasal wall; (1) extends posteriorly to the level of N.II.

390. Internasal vacuities or pits: (0) absent; (1) present.

391. Morphology of internasal vacuities: (0) undifferentiated or anterior palatal fossa; (1) shallow, paired pits with strong midline ridge; (2) deep, peer-shaped pits.

392. Basicranial morphology: (0) platybasic; (1) tropibasic.

393. Narrow interorbital septum: (0) absent; (1) present.

394. Optic lobes: (0) narrower than cerebellum; (1) same width or wider than cerebellum.

395. Space for forebrain and (at least) proximal portion of olfactory tracts narrow and elongate, extending between orbits: (0) absent; (1) present.

396. Rostral bar: (0) absent; (1) present.

397. Anteriormost articulation for the mandibular arch: (0) located anterior to the nasal capsules, terminal; (1) immediately below or posterior to nasal capsules, subterminal.

398. Palatobasal (or orbital) articulation: (0) posterior to the optic foramen; (1) anterior to the optic foramen, grooved, and overlapped by process or flange of palatoquadrate; (2) anterior to optic foramen, smooth, and overlaps or flanks articular surface on palatoquadrate.

399. Close association of pineal organ and nasal cavities: (0) absent; (1) present.

400. Trochlear nerve foramen anterior to optic nerve foramen: (0) absent; (1) present.

401. Pronounced sub-ethmoidal keel: (0) absent; (1) present.

402. Eyestalk or unfinished area on neurocranial wall for eyestalk: (0) absent; (1) present.

403. Eye stalk position: (0) positioned laterally on the orbital wall; (1) positioned ventrally on the subocular shelf wall.

404. Position of myodome for superior oblique eye muscles: (0) posterior and dorsal to foramen for nerve II; (1) anterior and dorsal to foramen.

405. Orbit directed mostly laterally and free of flanking endocranial cartilage or bone: (0) absent; (1) present.

406. Orbit dorsal or facing dorsolaterally, surrounded laterally by endocranium: (0) present; (1) absent.

407. Orbit larger than otic capsule: (0) absent; (1) present.

408. Paired pineal and parapineal tracts: (0) absent; (1) present.

409. Endoskeletal spiracular canal: (0) open; (1) partial enclosure or spiracular bar; 2 complete enclosure in canal.

410. Developed postorbital cavity: (0) absent; (1) present.

411. Unconstricted cranial notochord: (0) absent; (1) present.

412. Descending process of sphenoid (with its posterior extremity lacking

periostegeal lining): (0) absent; (1) present.

413. Opercular suspension on braincase: (0) absent; (1) present.

414. Ophthalmic foramen in anterodorsal extremity of orbit communicates with cranial interior: (0) absent; (1) present.

415. Internal carotids: (0) entering single or paired openings in the basicranium from a posterolateral angle; (1) entering basicranial opening(s) head-on from an extreme, lateral angle; (2) absent.

416. Entrance of internal carotids: (0) through separate openings flanking the hypophyseal opening or recess; (1) through a common opening at the central midline of the basicranium.

417. Postorbital process: (0) absent; (1) present.

418. Elongated distance between postorbital process and the articulation for hyomandibular: (0) absent; (1) present.

419. Postorbital process articulates with palatoquadrate: (0) absent; (1) present.

420. Postorbital process and arcade: (0) short and deep - width not more than maximum braincase width (excluding arcade); (1) process and arcade wide - width exceeds maximum width of braincase, and anteroposteriorly narrow; (2) process and arcade massive; (3) arcade forms postorbital pillar.

421. Postorbital process downturned, with anhedral angle relative to basicranium: (0) absent; (1) present.

422. Canal for jugular in postorbital process: (0) absent; (1) present.

423. Jugular canal diameter: (0) small; (1) large; (2) canal absent.

424. Jugular canal: (0) long (invested in otic region along length of skeletal labyrinth); (1) short (restricted to region anterior of skeletal labyrinth); (2) absent (jugular vein uninvested in otic region).

425. Canal, likely for trigeminal nerve (V) mandibular ramus, passes through the postorbital process from proximal dorsal entry to distal and ventral exit: (0) absent; (1) present.

426. Postorbital process expanded anteroposteriorly: (0) absent; (1) present.

427. C-bout notch separates postorbital process from supraotic shelf: (0) absent; (1) present.

428. Series of perforations for innervation of supraorbital sensory canal in supraorbital shelf: (0) absent; (1) present.

429. Spiracular groove on basicranial surface: (0) absent; (1) present.

430. Spiracular groove on lateral commissure: (0) absent; (1) present.

431. Subpituitary fenestra: (0) absent; (1) present.

432. Supraorbital shelf broad with convex lateral margin: (0) absent; (1) present.

433. Nerve VIII bifurcates before entering the labyrinth cavity: (0) bifurcates; (1) does not bifurcate.

434. Prehypophysial diencephalon: (0) the prehypophysial ventral “step” is absent or insignificantly captured by endocast, the ventral aspect of telencephalon is continuous with the anterior boundary of the hypophysial recess; (1) significant prehypophysial diencephalon, indicated by a “step” between the optic nerve canal marking the start of the diencephalon, and the

anterior boundary of the hypophysial recess.

435. Otic or pre-vagus section of myelencephalon: (0) long, longer than metencephalon; (1) short, shorter than metencephalon.

436. Extended prehypophysial portion of sphenoid: (0) absent; (1) present.

437. Main trunk of facial nerve: (0) elongate and passes anterolaterally through orbital floor; (1) stout and divides within otic capsule at the level of the postorbital process.

438. Hyoid ramus of facial nerve exits through posterior jugular opening: (0) absent; (1) present.

439. Ascending basisphenoid pillar pierced by common internal carotid: (0) absent; (1) present.

440. Canal for efferent pseudobranchial artery within basicranial cartilage: (0) absent; (1) present.

441. Position of basal/basipterygoid articulation: (0) same anteroposterior level as hypophysial opening; (1) anterior to hypophysial opening.

442. Basipterygoid process (basal articulation) with vertically oriented component: (0) absent; (1) present.

443. Expanded articular area anterior to basipterygoid process: (0) absent; (1) present.

444. Pituitary vein canal: (0) dorsal to level of basipterygoid process; (1) flanked posteriorly by basipterygoid process.

445. Pituitary vein canal: (0) discontinuous, enters the cranial cavity; (1)

discontinuous, enters hypophysial recess; (2) continuous transverse vein.

446. Pituitary vein in a transverse canal connecting the orbit: (0) absent; (1) present.

447. Short otico-occipital region of braincase: (0) absent; (1) present.

448. Position of hyomandibula articulation on neurocranium: (0) absent; (1) present.

449. Articulation facet with hyomandibular: (0) single-headed; (1) double-headed.

450. Position of hyomandibula articulation relative to structure of skeletal labyrinth: (0) anterior or lateral to skeletal labyrinth; (1) at level of posterior semicircular canal.

451. Hyomandibula articulates with neurocranium beneath otic shelf: (0) absent; (1) present.

452. Hyoid arch articulation: (0) on lateral commissure; (1) on otic capsule wall.

453. Relative position of jugular groove and hyomandibular articulation: (0) hyomandibula dorsal or same level (i.e. on bridge); (1) jugular vein passing dorsal or lateral to hyomandibula.

454. Hyomandibular facets where they straddle the jugular vein: (0) narrowly separated; (1) widely separated.

455. Hypophyseal chamber: (0) projects posteroventrally; (1) projects ventrally or anteroventrally.

456. Cuccullaris fossa (trapezius fossa): (0) open posteriorly; (1) constrained

posteriorly.

457. Cranial cavity and labyrinth: (0) widely spaced; (1) closely spaced.

458. Labyrinth cavity: (0) separated from the main neurocranial cavity by a cartilaginous or ossified capsular wall; (1) skeletal capsular wall absent.

459. External (horizontal) semicircular canal: (0) absent; (1) present.

460. External (horizontal) semicircular canal: (0) joins the vestibular region dorsal to posterior ampulla; (1) joins level with posterior ampulla.

461. Horizontal semicircular canal in dorsal view: (0) medial to path of jugular vein; (1) dorsal to jugular vein.

462. Crus commune connecting anterior and posterior semicircular canals: (0) present; (1) absent.

463. Crus commune of anterior and posterior semicircular canals: (0) dorsal to endocranial roof; (1) ventral to endocranial roof.

464. Angle of external semicircular canal: in lateral view, straight line projected through canal intersects anterior ampulla, external ampullae, and base of foramen magnum: (0) absent; (1) present.

465. Left and right external semicircular canals approach or meet the posterodorsal midine of the hindbrain roof: (0) absent; (1) present.

466. Preampullary portion of posterior semicircular canal: (0) absent; (1) present.

467. Sinus superior: (0) absent or indistinguishable from union of anterior and posterior canals with saccular chamber; (1) present.

468. Supraotic cavity: (0) absent; (1) present.

469. Lateral cranial canal: (0) absent; (1) present.

470. Subcircular endolymphatic foramen: (0) absent; (1) present.

471. External opening for endolymphatic ducts anterior to crus commune: (0) absent; (1) present.

472. Endolymphatic ducts: (0) posteriodorsally angled tubes; (1) tubes oriented vertically through median endolymphatic fossa.

473. Ampullary ends of anterior semicircular canal and external semicircular canal: (0) separated by the bulbous utricular chamber; (1) join before entering utricular chamber.

474. Orientation of saccular cavity in anterior view: (0) flat inclined; (1) steeply inclined or vertical.

475. Sacculus position: (0) restricted ventral to external semicircular canal; (1) extends dorsal to semicircular canal.

476. Vestibular cavity of the bony labyrinth shape: (0) drum-shaped; (1) irregularly shaped.

477. Endolymphatic complex shape: (0) simple and tube-like; (1) differentiated into distinctive sections.

478. Endolymphatic complex position: (0) lateral to the otic cartilaginous wall, close to inner ear; (1) mesial to the cartilaginous wall, close to brain cavity.

479. Endolymphatic duct distal direction in lateral view: (0) posteriorly directed; (1) vertically directed.

480. Endolymphatic duct distal direction in coronal view: (0) parallelled directed; (1) mesially directed; (2) laterally directed.

481. Endolymphatic fossa: (0) absent; (1) present.

482. Endolymphatic sacs: (0) absent; (1) present; (2) medially oriented endolymphatic fossae; (3) laterally oriented endolymphatic fossae.

483. Endolymphatic fossa elongate (slot-shaped), dividing dorsal otic ridge along midline: (0) absent; (1) present.

484. Perilymphatic fenestra within the endolymphatic fossa: (0) absent; (1) present.

485. Trigemino-facial recess: (0) absent; (1) present.

486. Posterior dorsal fontanelle: (0) absent; (1) present.

487. Shape of posterior dorsal fontanelle: (0) approximately as long as broad; (1) much longer than wide, slot-shaped.

488. Posterior dorsal fontanelle: (0) connected to persistent otico-occipital fissure; (1) separated from the fissure by posterior tectum.

489. Course of hyoid ramus of facial nerve (N. VII) relative to jugular canal: (0) traverses jugular canal, with separate exit in otic region; (1) intersects jugular canal, with exit through posterior jugular foramen.

490. Relationship of cranial endocavity to basisphenoid: (0) endocavity occupies full depth of sphenoid; (1) enodcavity dorsally restricted.

491. Supraotic shelf broad: (0) absent; (1) present.

492. Dorsal otic ridge: (0) absent; (1) present.

493. Dorsal otic ridge forms a crest posteriorly: (0) absent; (1) present.

494. Vestibular fontanelle: (0) absent; (1) present.

495. Hypotic lamina (and dorsally directed glossopharyngeal canal): (0) absent; (1) present.

496. Basicranial fenestra: (0) absent; (1) present.

497. Channel for dorsal aorta and/or lateral dorsal aortae: (0) passes through basicranium: 1 external to basicranium.

498. Dorsal aorta divides into lateral dorsal aortae: (0) posterior to occipital level; (1) anterior to level of the occiput.

499. Transverse otic process: (0) present; (1) absent.

500. Subcranial ridges: (0) absent; (1) present.

501. Synotic tectum: (0) absent; (1) present.

502. Shape of median dorsal ridge anterior to endolymphatic fossa: (0) developed as a squared-off ridge or otherwise ungrooved; (1) bears a midline groove.

503. Medial recess of the posteroventral mydome: (0) absent; (1) present.

504. Abducens, trigeminal nerves and pituitary vein: (0) opening via different foramina on the orbital wall; (1) sharing the same foramen on the orbital wall.

505. Number of "sel" canals: (0) five; (1) less than 5.

506. ’sel’ 1 canal bifurcation: (0) between orbit and field; (1) adjacent to lateral field; (2) adjacent to orbit.

507. Marginal vein: (0) absent; (1) present.

508. Profundus nerve: (0) emerges from the cranial cavity separately from the trigeminal nerve; (1) emerges together with the trigeminal nerve.

509. Transverse otic process: (0) not extending in front of orbits; (1) extending in front of orbits.

510. Nasal capsules in anterolateral corners of orbit: (0) no; (1) yes.

511. Vagal process: (0) forked; (1) unforked.

512. Rostral processes: (0) absent; (1) present.

513. Median rostral dorsal process of the braincase: (0) absent; (1) present.

514. Posttemporal fossae: (0) absent; (1) present.

515. Rostral organ: (0) absent; (1) present

516. Prespiracular dental plate: (0) absent; (1) present.

517. Suprapterygoid process: (0) absent; (1) present.

518. Processus supraorbitalis lateralis: (0) absent; (1) present.

519. Anterolateral fenestra in roof of otoccipital: (0) absent; (1) present.

520. Ventral cranial fissure connects with vestibular fontanelles: (0) absent; (1) present.

521. Bar across spiracular groove: (0) absent; (1) present.

522. Hypophysial opening in braincase: (0) absent; (1) present.

523. Hypophysial organ projection: (0) anterior (1) anteroventral (2) posteroventral.

524. Ventral rounded processes on preotic part of braincase: (0) absent; (1) present.

525. Notochord short, ending at the occipital cotylus: (0) absent; (1) present.

526. Accessory processes extend from ventral surface of nasal capsule: (0) absent; (1) present.

527. Internal carotid meets efferent pseudobranchial in orbit: (0) absent; (1) present.

528. Jugular vein passes through cranioquadrate passage: (0) absent; (1) present.

529. Anterior margin of ventral fissure: (0) straight; (1) sinusoidal.

530. Bulbous otic and auxiliary condyles for palatoquadrate articulation: (0) absent; (1) present.

531. Basal fenestra opening into floor of orbit: (0) absent; (1) present.

532. Nasal sacs: (0) unpaired; (1) paired.

533. 4 carotid foramina in parasphenoid: (0) absent; (1) present.

534. Parotic dental plates: (0) absent; (1) present.

535. Branchial ridges: (0) present; (1) reduced to vagal process; 2 absent (articulation made with bare cranial wall).

536. Periotic process: (0) absent; (1) present.

537. Sub-otic occipital fossa: (0) absent; (1) present.

538. Postotic process: (0) absent; (1) present.

539. Otic capsule extends posterolaterally relative to occipital arch: (0) absent; (1) present.

540. Otic capsules: (0) widely separated; (1) approaching dorsal midline.

541. Otic capsules project anteriorly between postorbital processes: (0) absent; (1) present.

542. Endocranial roof anterior to otic capsules domelike, smoothly convex dorsally and anteriorly: (0) absent; (1) present.

543. Roof of skeletal cavity for cerebellum and mesencephalon significantly higher than dorsal-most level of semicircular canals: (0) absent; (1) present.

544. Roof of the endocranial space for telencephalon and olfactory tracts offset ventrally relative to level of mesencephalon: (0) absent; (1) present.

545. Double octaval nerve foramina in chondrified mesial wall of otic capsule: (0) absent; (1) present.

546. Glossopharyngeal nerve exit: (0) foramen situated posteroventral to otic capsule and anterior to metotic fissure; (1) through metotic fissure.

547. Glossopharyngeal and vagus nerves share common exit from neurocranium: (0) absent; (1) present.

548. Ventral portion of occipital arch wedged between rear of otic capsules: (0) absent; (1) present.

549. Dorsal portion of occipital arch wedged between otic capsules: (0) absent; (1) present.

550. Craniospinal process ("supravagal process" in Stensio): (0) absent; (1) present.

551. Parachordal shape: (0) forming a broad, flat surface as wide as the otic capsules; (1) mediolaterally constricted relative to the otic capsules.

552. Ventral notch between parachordals: (0) absent; (1) present or entirely unfused.

553. Stalk-shaped parachordal/occipital region: (0) absent; (1) present.

554. Size of aperture to notochordal canal: (0) much smaller than foramen magnum; (1) as large, or larger, than foramen magnum.

555. Spino-occipital nerve foramina: (0) two or more, aligned horizontally; (1) one or two, dorsoventrally offset.

556. Occipital crest anteroposteriorly elongate, and extends from the roof of the posterior tectum: (0) absent; (1) present.

557. Paired occipital condyles: (0) absent; (1) present.

Axial and appendicular skeleton

558. Macromeric dermal shoulder girdle: (0) present; (1) absent.

559. Dermal neck-joint between paired main-lateral-line-bearing bones of skull and shoulder girdle: (0) absent; (1) present.

560. Dorsal articular lamina on trunk armour: (0) absent; (1) present.

561. Cranial fossa receiving the ventral articular lamina of the trunk: (0) absent; (1) present.

562. Trunk ventral articular lamina develops into flange or condyle: (0) absent, continuous along the articular lamina of the anterior dorsolateral plate; (1) present, the articular lamina develops into distinctive flange or condyle.

563. Rotatory contact of the articulation: (0) absent; (1) present

564. Lateral ridges on skull roof laterally defines the articulation: (0) absent; (1) present.

565. Dermal shoulder girdle composition: (0) ventral and dorsal (scapular) components; (1) ventral components only.

566. Dermal shoulder girdle forming a complete ring around the trunk: (0) present; (1) absent.

567. Pectoral fenestra completely encircled by dermal shoulder armour: (0) present; (1) absent.

568. Median dorsal plate: (0) absent; (1) present.

569. Pronounced internal crista (keel) on median dorsal surface of shoulder girdle: (0) absent; (1) present.

570. Anterior median dorsal plate: (0) absent; (1) present.

571. Anterior margin of unpaired anterior median dorsal plate: (0) broad; (1) pointed.

572. Anterior median dorsal plate (MD1) relative to posterior median dorsal plate (MD2) in length: (0) MD1 shorter than MD2; (1) MD1 longer than MD2.

573. Anterior lateral plate: (0) absent; (1) present.

574. Postbranchial lamina of trunk armour: (0) lateral; (1) internal.

575. Crista internalis of dermal shoulder girdle: (0) absent; (1) present.

576. Anteroventral plate: (0) absent; (1) present.

577. Number of median ventral plates: (0) two; (1) one.

578. Interolateral plate: (0) paired; (1) fused (unpaired semilunar plate).

579. Anterior ventrolateral plates of both sides: (0) in contact; (1) separated.

580. Brachial process: (0) absent; (1) present.

581. Presupracleithrum: (0) absent; (1) present.

582. Anocleithrum: (0) element developed as postcleithrum; (1) element developed as anocleithrum sensu stricto.

583. Dorsal cleithrum (AL of the Placodermi), ventral cleithrum (AVL of the Placodermi) and pectoral spine (SP of the Placodermi): (0) not fused; (1) fused.

584. Shape of dorsal blade of dermal shoulder girdle: (0) spatulate; (1) pointed.

585. Posterior dorsolateral plate or equivalent: (0) absent; (1) present.

586. Relationship of clavicle to cleithrum: (0) ascending process of clavicle overlapping cleithrum laterally; (1) ascending process of clavicle wrapping round anterior edge of cleithrum, overlapping it both laterally and mesially.

587. Paired fins relation to cephalic shield: (0) continuous;(1) delimited at pectoral sinus.

588. Intromittent organ for internal fertilization (’claspers’): (0) absent; (1) present.

589. Entepicondyle on humerus: (0) present; (1) absent.

590. PL and PDL overlap: (0) simple; (1) insertion.

591. Left and right posterior dorsolateral plates contact below the median dorsal plate: (0) absent; (1) present.

592. PDL plate visible externally: (0) present; (1) absent.

The original character state formulation was incorrect by comparison to their codings in the data set[193].

593. Posteriorly produced spine on MD plate: (0) absent; (1) present.

594. Joint in macromeric armoured pectoral fin: (0) absent; (1) present.

595. Cd1 (first dorsal central) and Cd2 (second dorsal central) plates: (0) in contact; (1) separated.

596. Clavicles/interolateral plates: (0) large plates, comparable in size to cleithrum; (1) paired small semilunar plates; (2) unpaired semilunar plates.

597. Chang's apparatus: (0) absent; (1) present.

598. Number of median dorsal plates: (0) one; (1) two; (2) three.

599. Anocleithrum sensu stricto: (0) exposed; (1) subdermal.

600. Median ventral trunk plates: (0) absent; (1) present.

601. Extracleithrum: (0) absent; (1) present.

602. Pectoral fin spine small (bivalve-like): (0) absent; (1) present.

603. Dorsal branch of main lateral line canal on posterior dorsolateral plate: (0) present; (1) absent.

The original character state formulation was incorrect by comparison to their codings in the data set[193].

604. Sharp downward bend in posterior dorsolateral plate sensory line: (0) absent; (1) present.

605. Horizontal caudal lobe: (0) absent; (1) present.

606. Triphycercal tail: (0) absent; (1) present.

607. Spine-brush complex: (0) absent; (1) present.

608. Series of median hexagonal scutes anterior to first dorsal fin: (0) absent; (1) present.

609. Intermediate spines with finlets: (0) absent; (1) present.

610. Median ventral prepectoral spine: (0) absent; (1) present.

611. Prepectoral spines form "necklace": (0) absent; (1) present.

612. Longitudinal rows of enlarged keeled scutes: (0) absent; (1) present.

613. Endoskeletal supports in pectoral fin: (0) multiple elements articulating with girdle; (1) single element ("humerus") articulating with girdle.

614. Triradiate scapulocoracoid: (0) absent; (1) present.

615. Flange on trailing edge of scapulocoracoid: (0) absent; (1) present.

616. Horizontal plate of scapulocoracoid: (0) absent; (1) present.

617. Subscapular foramen: (0) absent; (1) present.

618. Scapular process of shoulder endoskeleton: (0) absent; (1) present.

619. Scapular process with posterodorsal angle: (0) absent; (1) present.

620. Scapular infundibulum: (0) absent; (1) present.

621. Ventral margin of separate scapular ossification: (0) horizontal; (1) deeply angled.

622. Cross sectional shape of scapular process: (0) flattened or strongly ovate; (1) subcircular.

623. Endoskeletal postbranchial lamina on scapular process: (0) present; (1) absent.

624. Mineralisation of internal surface of scapular blade: (0) mineralised all around; (1) unmineralised on internal face forming a hemicylindrical crosssection.

625. Coracoid process: (0) absent; (1) present.

626. Procoracoid mineralisation: (0) absent; (1) present.

627. Paired (pectoral) fins: (0) absent; (1) present.

628. Pectoral fins covered in macromeric dermal armour: (0) absent; (1) present.

629. Armoured pectoral appendage: (0) unjointed; (1) jointed.

630. Pectoral fin base has large, hemispherical dermal component: (0) absent; (1) present.

631. Pectoral fin articulation: (0) monobasal; (1) dibasal; 2 three or more basals.

632. Fin base articulation on scapulocoracoid: (0) deeper than wide (stenobasal); (1) wider than deep (eurybasal).

633. Number of mesomeres in metapterygial axis: (0) five or fewer; (1) seven or more.

634. Biserial pectoral fin endoskeleton: (0) absent; (1) present.

635. Filamentous extension of pectoral fin from axillary region: (0) absent; (1) present.

636. Metapterygium pectinate subtriangular plate or bar supporting numerous (six or more) radials along distal edge: (0) absent; (1) present.

637. Metapterygial whip: (0) absent; (1) present.

638. Pectoral propterygium: (0) absent; (1) present.

639. Perforate propterygium: (0) absent; (1) present.

640. Distal articulation of propterygium: (0) with fin rays; (1) with a second enlarged element; (2) no articulation.

641. Pelvic fins: (0) absent; (1) present.

642. Pelvic girdle with substantial dermal component: (0) yes; (1) no.

643. Dermal pelvic clasper ossifications: (0) absent; (1) present.

644. Pelvic fin: (0) monobasal; (1) polybasal.

645. Intromittent organ containing bone, not associated with pelvic fins: (0) absent; (1) present.

646. Intromittent organ with one large J-shaped element: (0) absent; (1) present.

647. Intromittent organ ('clasper') consisting entirely of cartilage, formed from distal part of pelvic fin: (0) absent; (1) present.

648. Pelvic girdle with fused puboischiadic bar: (0) absent; (1) present.

649. Mixipterygial/mixopterygial claspers: (0) absent; (1) present.

650. Pre-pelvic clasper or tenaculum: (0) absent; (1) present.

651. Number of dorsal fins, if present: (0) one; (1) two.

652. Posterior dorsal fin shape: (0) base approximately as broad as tall, not broader than all of other median fins; (1) base much longer than the height of the fin, substantially longer than any of the other dorsal fins.

653. Basal plate in dorsal fin (Friedman & Brazeau (2010: character 42)): (0) absent; (1) present.

654. Branching radial structure articulating with dorsal fin basal plate: (0) absent;

(1) present.

655. Branching radials in paired fins: (0) absent; (1) present.

656. Posterior or pelvic-level dorsal fin with calcified base plate: (0) absent; (1) present.

657. Posterior dorsal fin with delta-shaped cartilage: (0) absent; (1) present.

658. Anal fin: (0) absent; (1) present.

659. Basal plate in anal fin (Friedman & Brazeau (2010: character 42)): (0) absent; (1) present.

660. Anal fin base narrow, posteriormost proximal segments radials broad: (0) absent; (1) present.

661. Caudal radials: (0) extend beyond level of body wall and deep into hypochordal lobe; (1) restricted to axial lobe.

662. Series of thoracic supraneurals: (0) absent; (1) present.

663. Supraneurals in axial lobe of caudal fin: (0) absent; (1) present.

664. Caudal neural and/or supraneural spines or radials: (0) short; (1) long, expanded, and supporting high aspect-ratio (lunate) tail with notochord extending to posterodorsal extremity; (2) notochord terminates pre-caudal extremity, neural and heamal radial lengths near symmetrical and support epichordal and hypochordal lobes respectively.

665. Synarcual: (0) absent; (1) present.

666. Calcified vertebral centra: (0) absent; (1) present.

667. Chordacentra: (0) absent; (1) present.

668. Chordacentra polyspondylous and consist of narrow closely packed rings: (0) absent; (1) present.

669. Brush complex of bilaterally distributed calcified tubes flanking or embedded in calcified cartilage core: (0) absent; (1) present.

Spines: fins, cranial and elsewhere

670. Dorsal fin spine: (0) absent; (1) present.

671. Dorsal fin spine at anterior (pectoral level) location only: (0) absent; (1) present.

672. Anal fin spine: (0) absent; (1) present.

673. Pectoral fin spine: (0) absent; (1) present.

674. Pelvic fin spine: (0) absent; (1) present.

675. Median fin spine insertion: (0) shallow, not greatly deeper than dermal bones / scales; (1) deep.

676. Intermediate fin spines: (0) absent; (1) present.

677. Intermediate spines when present: (0) one pair; (1) multiple pairs.

678. Prepectoral fin spines: (0) absent; (1) present.

679. Anteriormost intermediate spine associated with shoulder girdle: (0) absent; (1) present.

680. Cephalic spines: (0) absent; (1) present.

681. Pectoral fin spine with denticles along posterior surface: (0) absent; (1) present.

682. Fin spines with ridges: (0) absent; (1) present.

683. Fin spines with nodes: (0) absent; (1) present.

684. Fin spines with rows of large retrorse denticles: (0) absent; (1) present.

685. Fin spines (dorsal) with rows of large denticles: (0) absent; (1) on posterior surface; (2) on lateral surface.

686. Fin spine cross-section: (0) round or horseshoe shaped; (1) flat-sided, with rectangular profile.

687. Expanded spine rib on leading edge of spine: (0) absent; (1) present.

688. Spine ridges: (0) converging at the distal apex of the spine; (1) converging on leading edge of spine.

689. Dorsal fin spine cross section: (0) horseshoe shaped; (1) flat sided, with rectangular profile; (2) subcircular.

690. Anterior dorsal fin spine leading edge concave in lateral view: (0) absent; (1) present.

691. Shape of the trunk scales: (0) rhombic; (1) small/round; (2) cycloid.

692. Dermal ornament with parallel vermiform ridges on the trunk scales: (0) absent; (1) present.

693. Dermal ornament with concentric ridges on the trunk scales: (0) absent; (1) present.

694. Dermal ornament with tubercles on the trunk scales: (0) absent; (1) present.

## Supplementary Figures

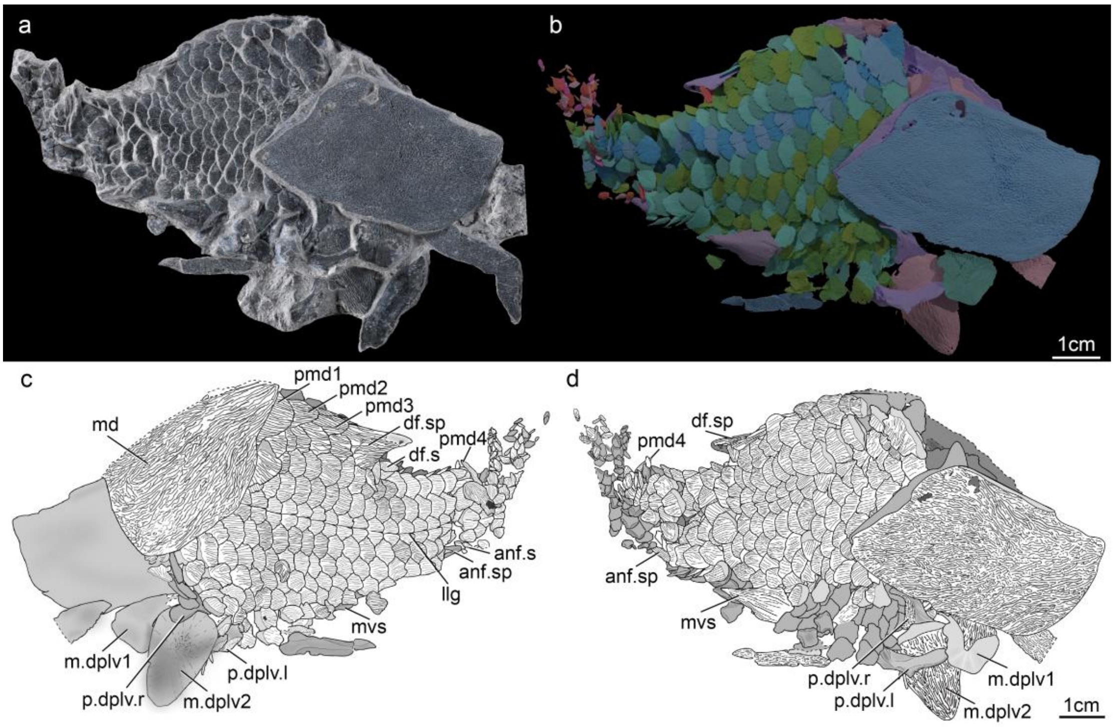


**Supplementary Fig. 1** ***Entelognathus primordialis*** **(IVPP V32322). a** Photograph in right lateral view. **b** Virtual rendering in right lateral view. **c** Interpretive drawing in left lateral view. **d** Interpretive drawing in right lateral view. anf.s anal fin scales, anf.sp anal fin spine, df.s dorsal fin scales, df.sp dorsal fin spine, llg lateral line groove, md median dorsal plate, m.dplv1 first median dermal pelvic plate, m.dplv2 second median dermal pelvic plate, pmd1–4 first to fourth post median dorsal scales, p.dplv.l left dermal pelvic plate, p.dplv.r right dermal pelvic plate, mvs median ventral scale.

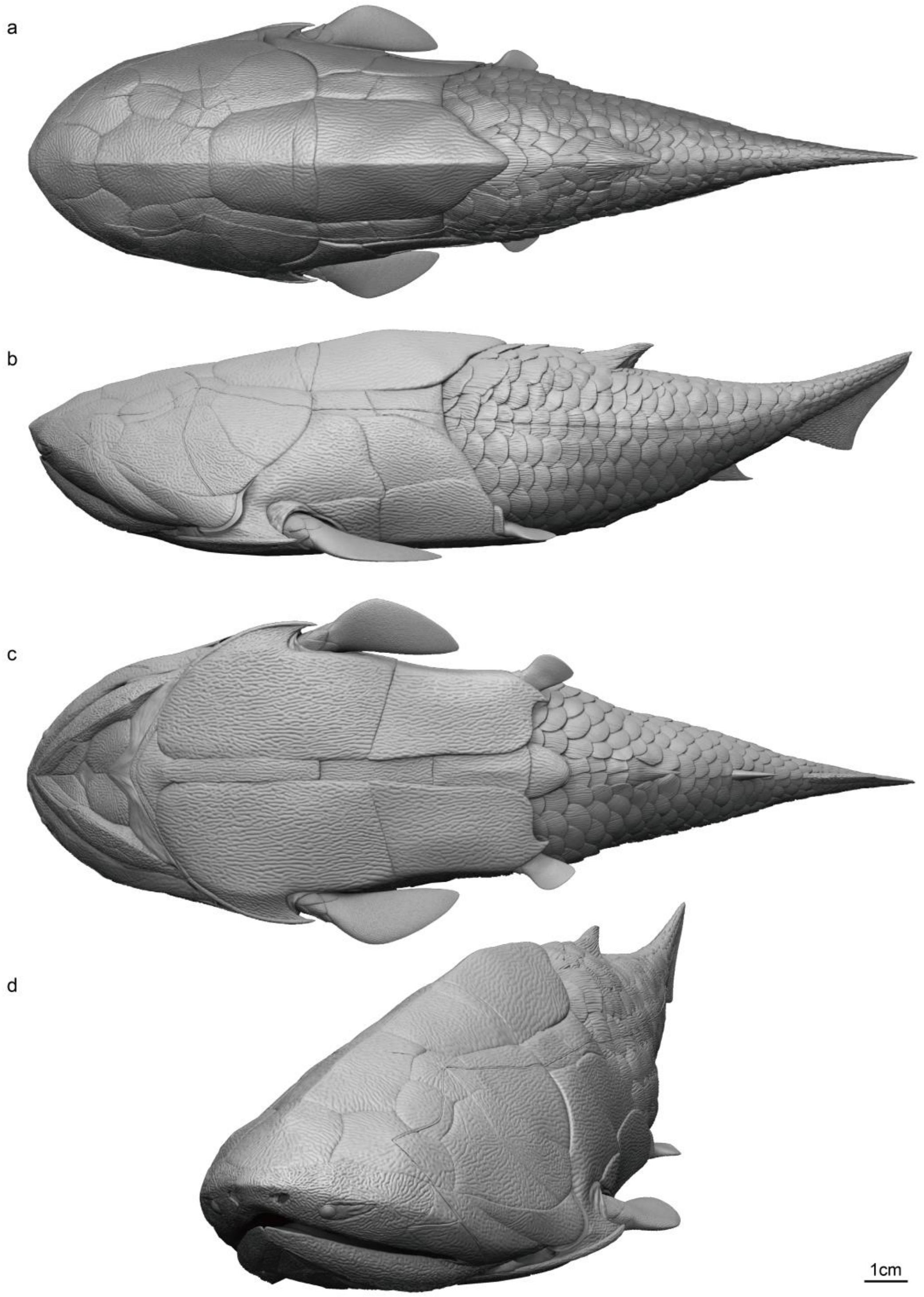


**Supplementary Fig. 2 Reconstruction of *Entelognathus primordialis*. a** dorsal view. **b** lateral view. **c** ventral view. **d** anterolateral view.

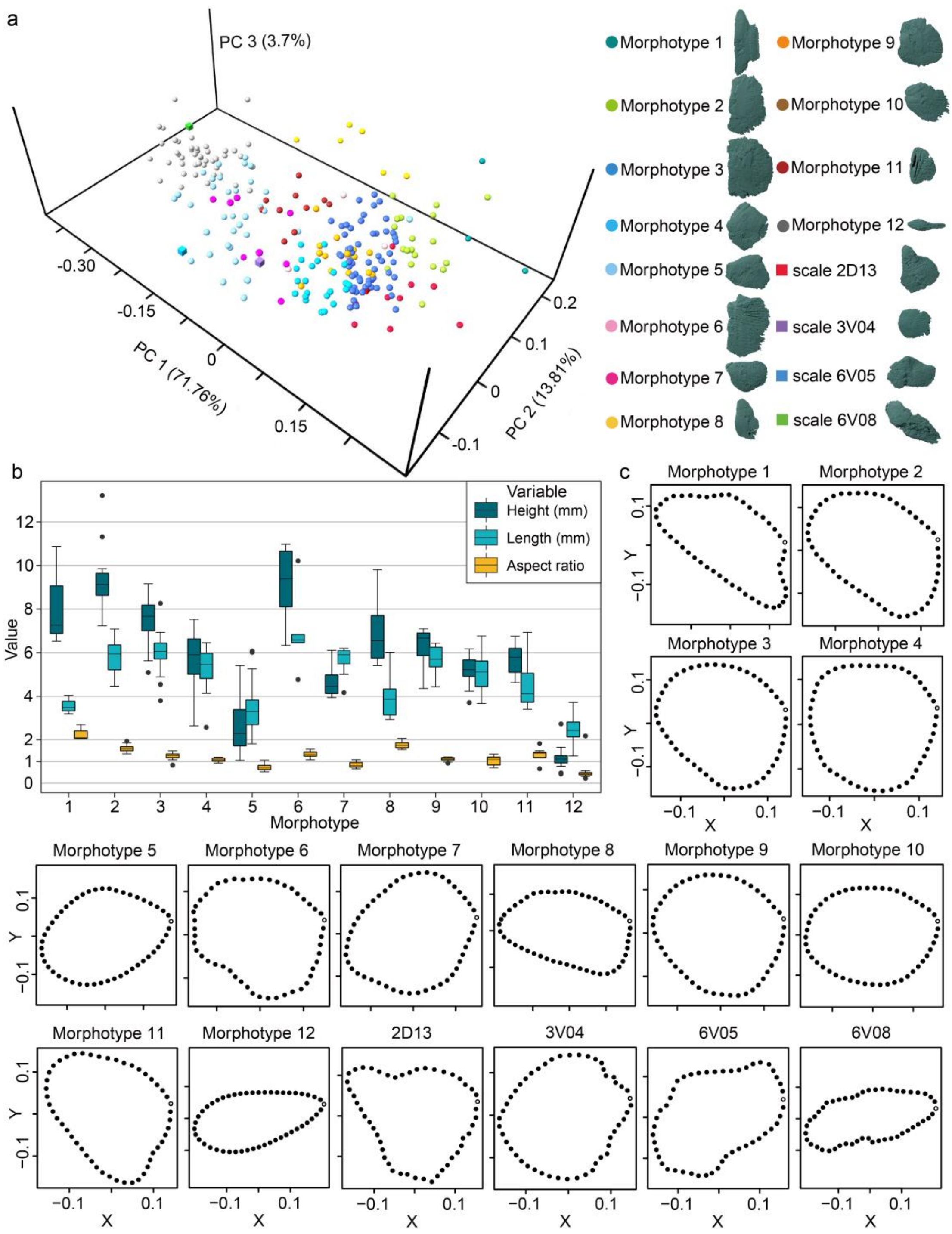


**Supplementary Fig. 3 Geometric Morphometric Analysis results of *Entelognathus primordialis* scales. a** 3D principal component analysis plot of the first three principal components showing the shape space of *Entelognathus* scales (n = 237 flank scales) based on landmark-based geometric morphometrics. **b** Average height, length, and aspect ratio of 12

morphotypes. **c** Mean shapes of 12 morphotypes and four special scales (the small circle represents the first landmark).

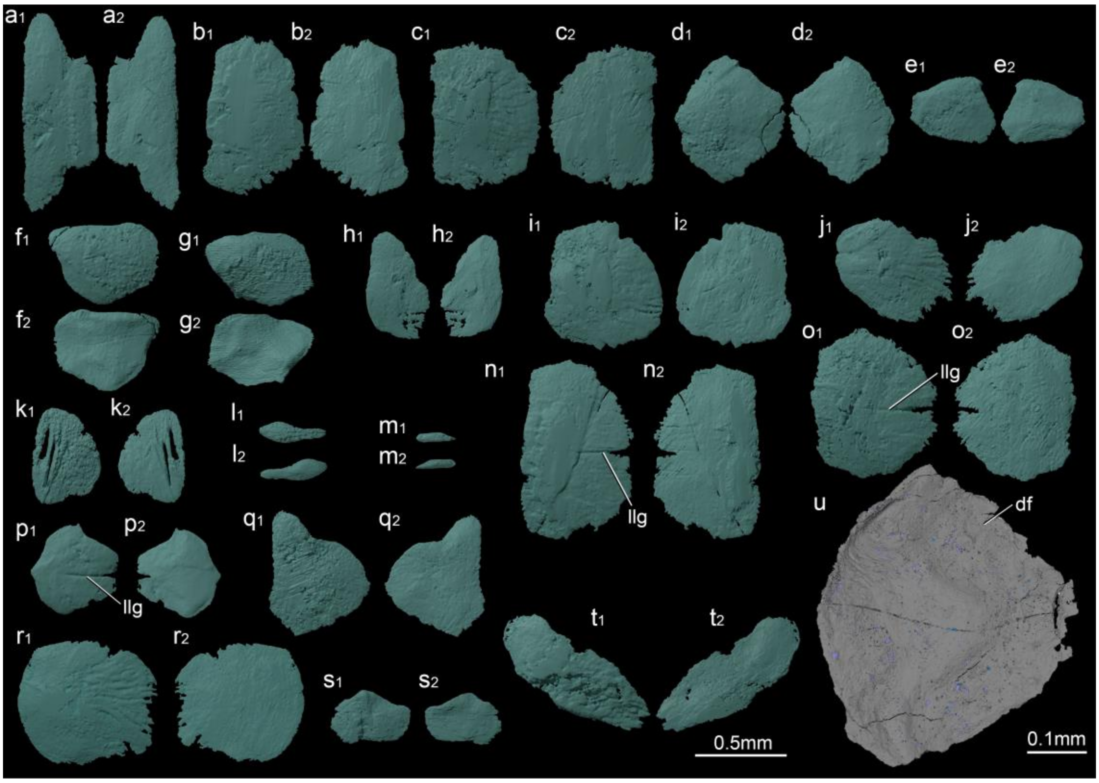


**Supplementary Fig. 4 3D virtual models of *Entelognathus primordialis* (IVPP V32322) scales. a** Morphotype 1 (1V01) in crown view (**a**$_1$) and basal view (**a**$_2$). **b** Morphotype 2 (1D03) in crown view (**b**$_1$) and basal view (**b**$_2$). **c** Morphotype 3 (2V06) in crown view (**c**$_1$) and basal view (**c**$_2$). **d** Morphotype 4 (1D17) in crown view (**d**$_1$) and basal view (**d**$_2$). **e** Morphotype 5 (2V20) in crown view (**e**$_1$) and basal view (**e**$_2$). **f** Morphotype 7 (2D19) in crown view (**f**$_1$) and basal view (**f**$_2$). **g** Morphotype 7 (3V17) in crown view (**g**$_1$) and basal view (**g**$_2$). **h** Morphotype 8 (3V01) in crown view (**h**$_1$) and basal view (**h**$_2$). **i** Morphotype 9 (3V07) in crown view (**i**$_1$) and basal view (**i**$_2$). **j** Morphotype 10 (4V04) in crown view (**j**$_1$) and basal view (**j**$_2$). **k** Morphotype 11 (5V01) in

crown view (**$k_1$**) and basal view (**$k_2$**). **l** Morphotype 12 (vf09) in crown view (**$l_1$**) and basal view (**$l_2$**). **m** Morphotype 12 (ts04) in crown view (**$m_1$**) and basal view (**$m_2$**). **n** Lateral line scale of Morphotype 2 (Ll04) in crown view (**$n_1$**) and basal view (**$n_2$**). **o** Lateral line scale of Morphotype 3 (Ll12) in crown view (**$o_1$**) and basal view (**$o_2$**). **p** Lateral line scale of Morphotype 4 (Ll21) in crown view (**$p_1$**) and basal view (**$p_2$**). **q** Special scale (2D13) in crown view (**$q_1$**) and basal view (**$q_2$**). **r** Special scale (3V04) in crown view (**$r_1$**) and basal view (**$r_2$**). **s** Special scale (6V05) in crown view (**$s_1$**) and basal view(**$s_2$**). **t** Special scale (6V08) in crown view (**$t_1$**) and basal view (**$t_2$**). **u** Morphotype 4 scale (V32323.5) in basal view. llg lateral line groove, df depressed field.

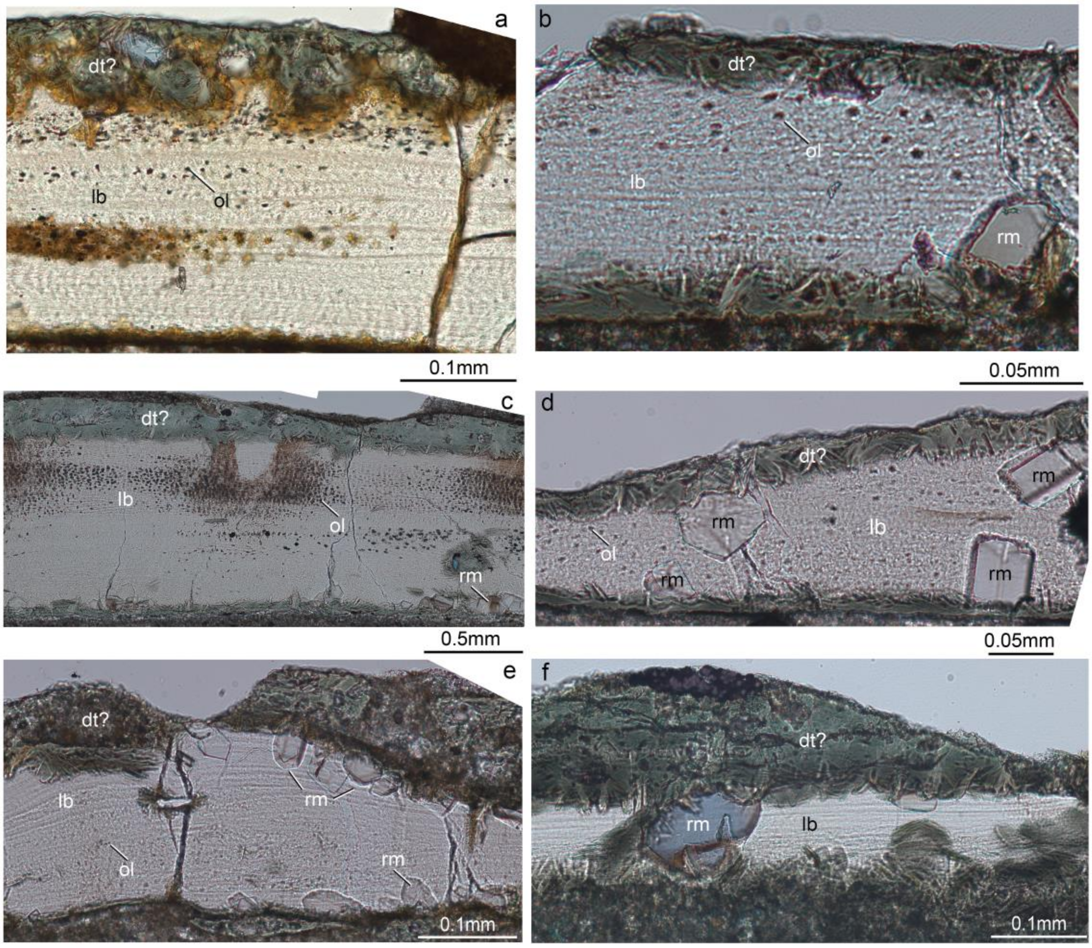


**Supplementary Fig. 5 Thin sections of *Entelognathus primordialis* and *Guiyu oneiros.*** **a** Vertical transverse section of a posterior lateral plate of *Entelognathus primordialis* (IVPP V32323.1). **b** Vertical longitudinal section of a scale of *Entelognathus primordialis* (V32323.3). **c** Vertical longitudinal section of an anterior ventrolateral plate (V32323.2). **d** Vertical transverse section of a scale (V32323.4). **e** Vertical transverse section of a lower jaw of *Guiyu oneiros* (V32324.1). **f** Vertical transverse section of a scale of *Guiyu oneiros* (V32324.2). dt? supposed dentine, lb lamellar bone, ol osteocyte lacunae, rm recrystallized minerals.

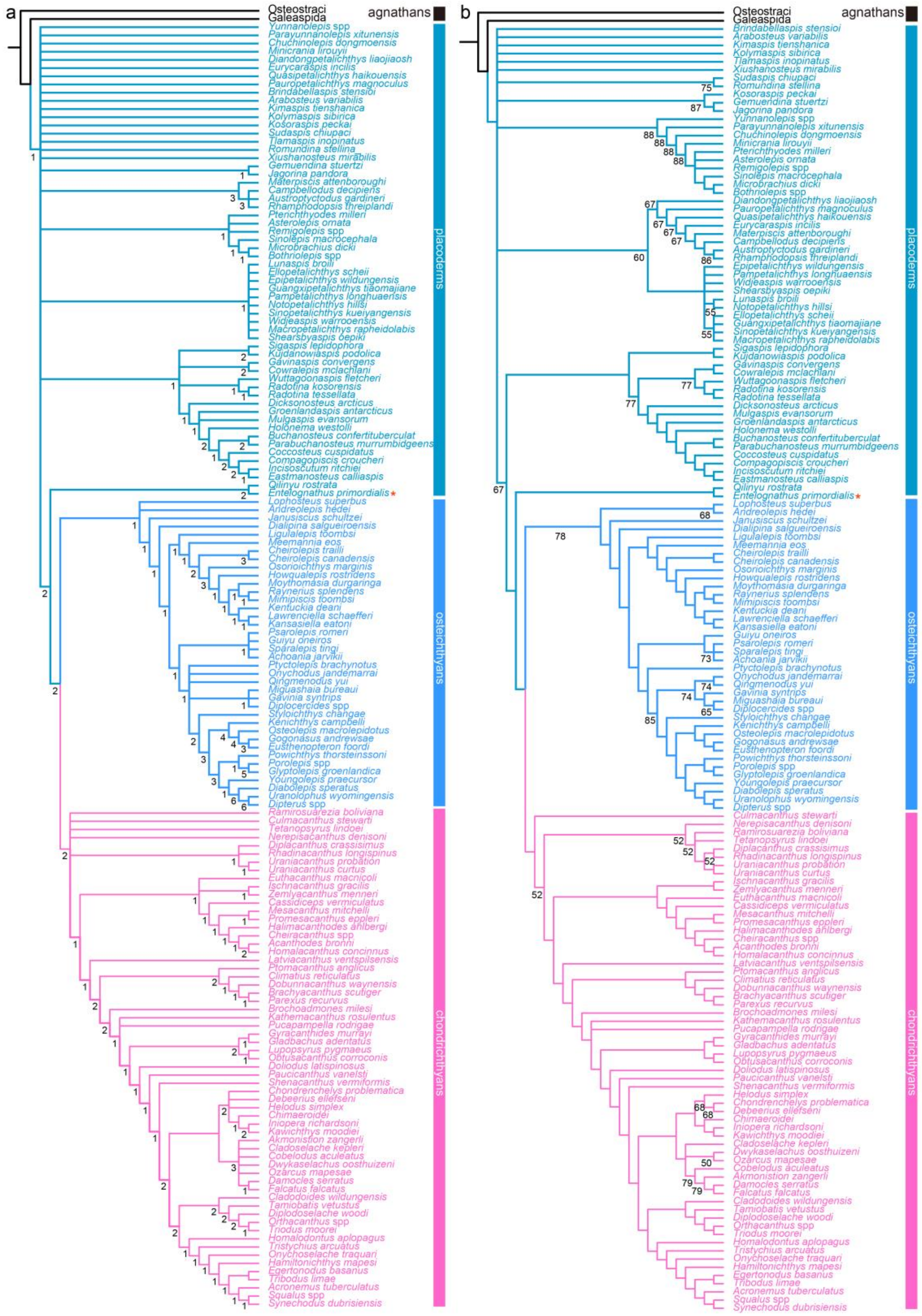


**Supplementary Fig. 6 Results of phylogenetic analysis. a** Strict consensus tree of 100,000 most parsimonious trees. Numbers below branches represent Bremer decay indices. **b** 50% Majority-rule consensus tree. Numbers below

branches indicate the percentage of most-parsimonious trees that contain a particular clade (100% unless otherwise indicated).

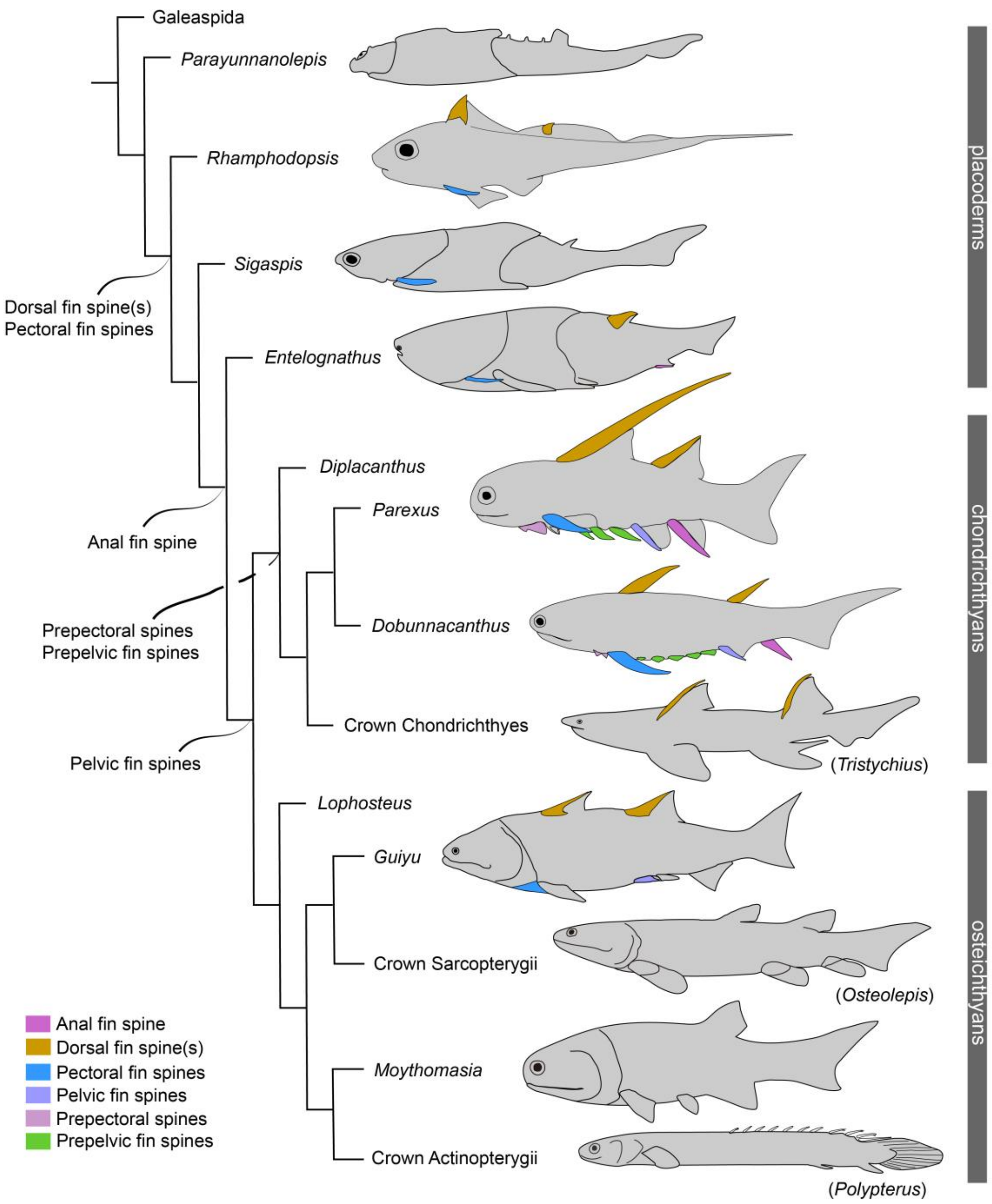


**Supplementary Fig. 7 Summary phylogeny of early jawed vertebrates showing the transformation in fin spines.** This cladogram is simplified from the strict consensus tree of the 100,000 most parsimonious trees

(Supplementary Fig. 6a). The character transformations mapped on the tree are based on the results of phylogenetic analyses. Drawing sources: *Parayunnanplepis*[226]; *Rhamphodopsis*[189]; *Sigaspis*[194]; *Parexus*[169]; *Dobunnacanthus*[67]; *Tristychius*[212]; *Guiyu*[1]; *Osteolepis*[228]; *Moythomasia*[229]; *Polypterus*[230].

## Supplementary References

1 Cui, X., Qiao, T. & Zhu, M. Scale morphology and squamation pattern of *Guiyu oneiros* provide new insights into early osteichthyan body plan. *Sci. Rep.* **9**: 4411 (2019).

2 Brazeau, M. D. & Valerie, D. W. The hyoid arch and braincase anatomy of *Acanthodes* support chondrichthyan affinity of 'acanthodians'. *Proc. R. Soc. B* **282**: 20152210 (2015).

3 Coates, M. I. The origin of vertebrate limbs. *Dev., Suppl.* 169–180 (1994).

4 Davis, S. P., Finarelli, J. A. & Coates, M. I. *Acanthodes* and shark-like conditions in the last common ancestor of modern gnathostomes. *Nature* **486**: 247–250 (2012).

5 Gross, W. Histologische studien am Aussenskelett fossiler agnathen und fische. *Palaeontogr. Abt. A* **83**: 1–60 (1935).

6 Miles, R. S. Articulated acanthodian fishes from the Old Red Sandstone of England, with a review of the structure and evolution of the acanthodian shoulder-girdle. *Bull. Br. Mus. Nat. Geol.* **24**: 111–213 (1973).

7 Miles, R. S. in *Interrelationships of Fishes* (eds. Greenwood, P. H., Miles, R. S. & Patterson, C.) 63–103 (Academic Press, 1973).

8 Watson, D. M. S. The acanthodian fishes. *Philos. Trans. R. Soc.* **228**: 49–146 (1937).

9 Zhu, M. & Yu, X.-B. in *Recent Advances in the Origin and Early Radiation of Vertebrates* (eds. Arratia, G. M., Wilson, V. H., & Cloutier, R.) 271–286 (Verlag Dr. Friedrich Pfeil, 2004).

10 Zhu, M. & Yu, X.-B. Stem sarcopterygians have primitive polybasal fin articulation. *Biol. Lett.* **5**: 372–375 (2009).

11 Zhu, M., Yu, X.-B. & Ahlberg, P. E. A primitive sarcopterygian fish with an eyestalk. *Nature* **410**: 81–84 (2001).

12 Maisey, J. G., 2011. The braincase of the Middle Triassic shark *Acronemus tuberculatus* (Bassani, 1886). *Palaeontology* **54**: 417–428.

13 Coates, M. I. & Sequeira, S. E. K., 1998. The braincase of a primitive shark. *Earth Environ. Sci. Trans. R. Soc. Edinb.* **89**: 63–85.

14 Coates, M. I. & Sequeira, S. E. K. A new stethacanthid chondrichthyan from the Lower Carboniferous of Bearsden, Scotland. *J. Vertebr. Paleontol.* **21**: 438–459 (2001).

15 Coates, M. I., Sequeira, S. E. K., Sansom, I. J. & Smith, M. M. Spines and tissues of ancient sharks. *Nature* **396**: 729–730 (1998).

16 Chen, D.-L., Blom, H., Sanchez, S., Tafforeau, P. & Ahlberg, P. E. The stem osteichthyan *Andreolepis* and the origin of tooth replacement. *Nature* **539**: 237–241 (2016).

17 Olive, S., Goujet, D., Lelièvre, H. & Janjou, D. A new placoderm fish (Acanthothoraci) from the Early Devonian Jauf Formation (Saudi Arabia). *Geodiversitas* **33**: 393–409 (2011).

18 Olive, S., Goujet, D., Lelièvre, H. & Janvier, P. The growth of the skull roof plates in *Arabosteus variabilis* (Acanthothoraci, Placodermi) from the Early Devonian Jauf Formation (Saudi Arabia): Preliminary results. *Paleontol. J.* **48**: 992–1002 (2014).

19 Lukševičs, E. The orbito-nasal area of *Asterolepis ornata*, a Middle Devonian placoderm fish. *J. Vertebr. Paleontol.* **21**: 687–692 (2001).

20 Upeniece, I. The unique fossil assemblage from the Lode Quarry (Upper Devonian, Latvia). *Mitt. Mus. Natkd. Berl. Geowiss.* **4**: 101–119 (2001).

21 Vaskaninova, V. *Asterolepis ornate* Eichwald, 1840 (Placodermi) ve sbirkach Ceske geologicke sluzby. *Zpravy o geologickych vyzkumech* **2008**: 131–132 (2009).

22 Long, J. A. Ptyctodontid fishes (Vertebrata, Placodermi) from the Late Devonian Gogo Formation, Western Australia, with a revision of the European genus *Ctenurella* Ørvig, 1960. *Geodiversitas* **19**: 515–555 (1997).

23 Long, J. A. & Trinajstic, K. The Late Devonian Gogo Formation Lägerstatte of western Australia: exceptional early vertebrate preservation and diversity. *Annu. Rev. Earth. Planet. Sci.* **38**: 255–279 (2010).

24 Young, G. C. A new Early Devonian placoderm from New South Wales, Australia, with a discussion of placoderm phylogeny. *Palaeontogra. Abt. A* **167**: 10–76 (1980).

25 Hanke, G. F. & Wilson, M. V. H. Anatomy of the Early Devonian acanthodian *Brochoadmones milesi* based on nearly complete body fossils, with comments on the evolution and development of paired fins. *J. Vertebr. Paleontol.* **26**: 526–537 (2006).

26 Burrow, C. J. & Turner, S. Devonian placoderm scales from Australia. *J. Vertebr. Paleontol.* **18**: 677–695 (1998).

27 Long, J. A., Mark-Kurik, E. & Young, G. C. Taxonomic revision of buchanosteoid placoderms (Arthrodira) from the Early Devonian of south-eastern Australia and Arctic Russia. *Aust. J. Zool.* **62**: 26–43 (2014).

28 Gagnier, P. Y. and Wilson, M. V. H. Early Devonian acanthodians from northern Canada. *Palaeontology* **39**: 241–258 (1996).

29 Stensiö, E. A. *The Cephalaspids of Great Britain*. (British Museum Natural History, 1932).

30 White, E. I. in *Studies on Fossil Vertebrates* (ed. Westoll, T.S.) 212–234 (London,1958).

31 Burrow, C. J., den Blaauwen, J. & Newman, M. A redescription of the three longest-known species of the acanthodian *Cheiracanthus* from the Middle Devonian of Scotland. *Palaeontol. Electron.* **23**: a15 (2020).

32 Arratia, G. and Cloutier, R. in *Devonian Fishes and Plants of Miguasha, Quebec, Canada* (eds. Schultze, H.-P. & Cloutier, R.) 165–197 (Verlag Dr Friedrich Pfeil, 1996).

33 Pearson, D. M. & Westoll, T. S. The Devonian actinopterygian *Cheirolepis* Agassiz. *Earth Environ. Sci. Trans. R. Soc. Edinb.* **70**: 337–399 (1979).

35 Giles, S. *et al.* Endoskeletal structure in *Cheirolepis* (Osteichthyes, Actinopterygii), an early ray-finned fish. *Palaeontology* **58**: 849–870 (2015).

36 Didier, D. A. Phylogenetic systematics of extant chimaeroid fishes (Holocephali, Chimaeroidei). *Am. Mus. Novit.* **3119**: 1–86 (1995).

37 Finarelli, J. A. & Coates, M. I. First tooth-set outside the jaws in a vertebrate. *Proc. R. Soc. B*. **279**: 775–779 (2011).

38 Finarelli, J. A. & Coates, M. I. *Chondrenchelys problematica* (Traquair, 1888) redescribed: a Lower Carboniferous, eel-like holocephalan from Scotland. *Earth Environ. Sci. Trans. R. Soc. Edinb.* **105**: 35–59 (2014).

39 Moy-Thomas, J. A. The structure and affinities of *Chondrenchelys problematica. Proc. Zool. Soc. Lond*. **105**: 391–404 (1935).

40 Tông-Dzuy, T. & Janvier, P. Les Vertébrés du Dévonien inférieur du Bac Bo oriental (provinces de Bac Thaï et Lang Son, Viêt Nam). *Bull. Mus. Natl. Hist. Nat., 4C* **12**: 143–223 (1990).

41 Maisey, J. G. Braincase of the Upper Devonian shark *Cladodoides wildungensis* (Chondrichthyes, Elasmobranchii), with observations on the braincase in early chondrichthyans. *Bull. Am. Mus. Nat.* **288**: 1–103 (2005).

42 Bendix-Almgreen, S. E. in *Problèmes actuels de Paléontologie-Evolution des Vertébrés* (ed. Lehman, J. P.) 111–123 (Colloques Internationaux du Centre National de la Recherche Scientifique, Paris, 1975).

43 Maisey, J. G. The braincase in Paleozoic symmoriiform and cladoselachian sharks. *Bull. Am. Mus. Nat.* **307**: 1–122 (2007).

44 Schaeffer, B. The xenacanth shark neurocranium, with comments on elasmobranch monophyly. *Bull. Am. Mus. Nat.* **169**: 1–66 (1981).

45 Burrow, C. J., Davidson, R. G., Den Blaauwen, J. L. & Newman, M. J. Revision of *Climatius reticulatus* Agassiz, 1844 (Acanthodii, Climatiidae), from the Lower Devonian of Scotland, based on new histological and morphological data. *J. Vertebr. Paleontol.* **35**: e913421 (2015).

46 Ivanov, A. Early Permian chondrichthyans of the middle and south urals. *Rev. Bras. Paleontol.* **8**: 127–138 (2005).

47 Miles, R. S. & Westoll, T. S. The placoderm fish *Coccosteus cuspidatus* Miller ex Agassiz from the Middle Old Red Sandstone of Scotland. Part I. descriptive morphology. *Earth Environ. Sci. Trans. R. Soc. Edinb.* **67**: 373–476 (1968).

48 Gardiner, B. G. & Miles, R. S. Eubrachythoracid arthrodires from Gogo, Western Australia. *Zool. J. Linn. Soc.* **112**: 443–477 (1994).

49 Ritchie, A. *Cowralepis*, a new genus of phyllolepid fish (Pisces, Placodermi) from the Late Middle Devonian of New South Wales, Australia. *Proc. Linn. Soc. N.S.W.* **126**: 215–259 (2005).

50 Burrow, C. J. & Young, G. C. New information on *Culmacanthus* (Acanthodii: Diplacanthiformes) from the ?Early-middle Devonian of southeastern Australia. *P. Linn. Soc. N. S. W.* **134**: 21–29 (2012).

51 Long, J. A. A new diplacanthoid acanthodian from the Late Devonian of Victoria. *Mem. Assoc. Australas. Palaeontol.* **1**: 51–65 (1983).

52 Lund, R. On *Damocles serratus*, nov. gen. et sp. (Elasmobranchii: Cladodontida) from the Upper Mississippian Bear Gulch Limestone of Montana. *J. Vertebr. Paleontol.* **6**: 12–19 (1986).

53 Lund, R. & Grogan, E. D. Relationships of the Chimaeriformes and the basal radiation of the Chondrichthyes. *Rev. Fish Biol. Fish.* **7**: 65–123 (1997).

54 Grogan, E. D. & Lund, R. *Debeerius ellefseni* (fam. nov., gen. nov., spec. nov.), an autodiastylic chondrichthyan from the Mississippian bear gulch limestone of Montana (USA), the relationships of the Chondrichthyes, and comments on gnathostome evolution. *J. Morphol.* **243**: 219–245 (2000).

55 Chang, M.-M. *Diabolepis* and its bearing on the relationships between porolepiforms and dipnoans. *Bull. Mus. Natl. Hist. Nat., 4C* **17**: 235–268 (1995).

56 Chang, M.-M. & Yu, X.-B. Structure and phylogenetic significance of *Diabolichthys speratus* gen. et sp. nov., a new dipnoan-like form from the

Lower Devonian of eastern Yunnan, China. *Proc. Linn. Soc. N. S. W*. **107**: 171–184 (1984).

57 Zhu, M. in *Early Vertebrates and Related Problems of Evolutionary Biology* (eds. Chang, M.-M., Liu, Y.-H. & Zhang, G.-R.) 179–194 (Science Press, 1991).

58 Schultze, H.-P. Palaeoniscoidea-schuppen aus dem Unterdevon Australiens und Kansas und aus dem Mitteldevon Spitzbergens. *Bull. Br. Mus. Nat. Hist. Geol.* **16**: 343–368 (1968).

59 Schultze, H.-P. & Cumbaa, S. L. in *Major Events in Early Vertebrate Evolution: Palaeontology, Phylogeny, Genetics and Development* (ed. Ahlberg, P. E.) 315–332 (Taylor & Francis, 2001).

60 Goujet, D. F. in *Problèmes actuels de Paléontologie-Evolution des Vertébrés* (ed. Lehman, J. P.) 81–99 (Colloques Internationaux du Centre National de la Recherche Scientifique, 1975).

61 Goujet, D. F. *Les poissons placodermes du Spitsberg. Arthrodires Dolichothoraci de la Formation de Wood Bay (Dévonien inférieur)* (Editions Centre National Recherche Scientifique, Cahiers de Paléontologie,1984).

62 Burrow, C. J., den Blaauwen, J., Newman, M. & Davidson, R. The diplacanthid fishes (Acanthodii, Diplacanthiformes, Diplacanthidae) from the Middle Devonian of Scotland. *Palaeontol. Electron.* **19**: 1–83 (2016).

63 Forey, P. L. *History of the Coelacanth Fishes*. (Chapman & Hall, 1998).

64 Dick, J. R. F. *Diplodoselache woodi* gen. et sp. nov., an early Carboniferous shark from the Midland Valley of Scotland. *Earth Environ. Sci. Trans. R. Soc. Edinb.* **72**: 99–113 (1981).

65 Ahlberg, P. E. & Trewin, N. H. The postcranial skeleton of the Middle Devonian lungfish *Dipterus valenciennesi*. *Earth Environ. Sci. Trans. R. Soc. Edinb.* **85**: 159–175 (1995).

66 White, E. I. The head of *Dipterus valenciennesi* Sedgwick & Murchison. *Bull. Br. Mus. Nat. Hist. Geol.* **11**: 1–45 (1965).

67 Dearden, R. P. *et al.* A revision of *Vernicomacanthus* Miles with comments on the characters of stem-group chondrichthyans. *Pap. Palaeontol.* **7**, 1949–1976 (2021).

68 Burrow, C. J. & Desbiens, S. Teeth and tooth whorls of the stem chondrichthyan *Doliodus* from the Early Devonian of the Gaspé Sandstone Group, Gaspé Peninsula, Quebec, Canada. *Span. J. Palaeontol*. **38**: 15–22 (2023).

69 Maisey, J. G., Miller, R. & Turner, S. The braincase of the chondrichthyan *Doliodus* from the Lower Devonian Campbellton Formation of New Brunswick, Canada. *Acta Zool.* **90**: 109–122 (2009).

70 Miller, R. F., Cloutier, R. & Turner, S. The oldest articulated chondrichthyan from the Early Devonian period. *Nature* **425**: 501–504 (2003).

71 Coates, M. I. *et al.* A symmoriiform chondrichthyan braincase and the origin of chimaeroid fishes. *Nature* **541**: 208–211 (2017).

72 Dennis-Bryan, K. A new species of eastmanosteid arthrodire (Pisces: Placodermi) from Gogo, Western Australia. *Zool. J. Linn. Soc.* **90**: 1–64 (1987).

73 Teng, Y.-H. *et al.* First Cretaceous fish fauna from Malaysia. *J. Vertebr. Paleontol.* **39**: e1573735 (2019).

74 Castiello, M. *Neurocranial anatomy of three unusual placoderms revealed by computed tomography scanning, and their implications for early gnathostomes evolution*. (Imperial College, 2018).

75 Zhu, M. *et al.* A Silurian placoderm with osteichthyan-like marginal jaw bones. *Nature* **502**: 188–193 (2013).

76 Ørvig, T. Notes on some Paleozoic lower vertebrates from Spitsbergen and North America. *Nor. J. Geol.* **37**: 285–353 (1957).

77 Sallan, L. C. and Coates, M. I. End-Devonian extinction and a bottleneck in the early evolution of modern jawed vertebrates. *Proc. Natl. Acad. Sci. U.S.A.* **107**: 10131–10135 (2010).

78 Liu, Y.-H., 1991. in *Early Vertebrates and Related Problems of Evolutionary Biology* (eds. Chang, M.-M., Liu, Y.-H. & Zhang, G.-R.) 139–177 (Science Press, 1991).

79 Jarvik, E. *Basic Structure and Evolution of Vertebrates, Volume 1*. (Academic Press, 1980).

80 Jarvik, E. *Basic Structure and Evolution of Vertebrates, Volume 2*. (Academic Press, 1980).

81 Newman, M. J., Burrow, C. J., Den Blaauwen, J. L. & Davidson, R. G. The Early Devonian acanthodian *Euthacanthus macnicoli* Powrie, 1864 from the Midland Valley of Scotland. *Geodiversitas* **36**: 321–348 (2014).

82 Lund, R. The morphology of *Falcatus falcatus* (St. John and Worthen), a Mississippian stethacanthid chondrichthyan from the Bear Gulch Limestone of Montana. *J. Vertebr. Paleontol.* **5**: 1–19 (1985).

83 Long, J. A. A new genus of fossil coelacanth (Osteichthyes: Coelacanthiformes) from the Middle Devonian of southeastern Australia. *Rec. West. Aust. Mus.* Suppl. **57**: 37–53 (1999).

84 Dupret, V & Zhu, M. The earliest phyllolepid (Placodermi, Arthrodira) from the Late Lochkovian (Early Devonian) of Yunnan (South China). *Geol. Mag.* **145**: 257–278 (2008).

85 Gross, W. *Gemuendina stuertzi* Traquair. Neuuntersuchung. *Notizbl. hess. Landesami. Bodenforsch.* **91**: 36–73 (1963).

86 Coates, M. I. *et al.* An early chondrichthyan and the evolutionary assembly of a shark body plan. *Proc. R. Soc. B* **285**: 20172418 (2018).

87 Ahlberg, P. E. Paired fin skeletons and relationships of the fossil group Porolepiformes (Osteichthyes: Sarcopterygii). *Zool. J. Linn. Soc.* **96**: 119–166 (1989).

88 Jarvik, E. Middle and Upper Devonian Porolepiformes from East Greenland with special reference to *Glyptolepis groenlandica* n. sp. and a discussion on the structure of the head in the Porolepiformes. *Meddel. Grønland* **187**: 1–307 (1972).

89 Holland, T. Pectoral girdle and fin anatomy of *Gogonasus andrewsae* Long, 1985: implications for tetrapodomorph limb evolution. *J. Morphol.* **274**: 147–164 (2013).

90 Holland, T. The endocranial anatomy of *Gogonasus andrewsae* Long, 1985 revealed through micro CT-scanning. *Earth Environ. Sci. Trans. R. Soc. Edinb.* **105**: 9–34 (2014).

90 Long, J. A. A new osteolepidid fish from the Upper Devonian Gogo Formation, western Australia. *Rec. West. Aust. Mus.* **12:** 361–377 (1985).

91 Long, J. A., Barwick, R. E. & Campell, K. S. Osteology and functional morphology of the osteolepiform fish *Gogonasus andrewsae* Long, 1985, from the Upper Devonian Gogo Formation, Western Australia. *Rec. West. Aust. Mus.* Suppl. **53**: 1–89 (1997).

92 Long, J. A. *et al.* An exceptional Devonian fish from Australia sheds light on tetrapod origins. *Nature* **444**: 199–202 (2006).

93 Ritchie, A. *Groenlandaspis* in Antarctica, Australia and Europe. *Nature* **254**: 569–573 (1975).

94 Young, G. C. The Aztec fish fauna (Devonian) of Southern Victoria Land: evolutionary and biogeographic significance. *Geol. Soc. Spec. Publ. Lond.* **47**: 43–62 (1989).

95 Ji, S.-A. & Pan, J. The macropetalichthyids (Placodermi) from Guangxi and Hunan, China. *Vert. PalAsiat.* **35**: 18–34 (1997).

96 Qiao, T. & Zhu, M. Cranial morphology of the Silurian sarcopterygian *Guiyu oneiros* (Gnathostomata: Osteichthyes). *Sci. China Earth Sci* **53**: 1836–1848 (2010).

97 Zhu, M. *et al.* The oldest articulated osteichthyan reveals mosaic gnathostome characters. *Nature* **458**: 469–474 (2009).

98 Warren, A., Currie, B. P., Burrow, C. & Turner, S. A redescription and reinterpretation of *Gyracanthides murrayi* Woodward 1906 (Acanthodii, Gyracanthidae) from the Lower Carboniferous of the Mansfield Basin, Victoria, Australia. *J. Vertebr. Paleontol.* **20**: 225–242 (2000).

99 Burrow, C. J., Trinajstic, K. & Long, J. A. First acanthodian from the Upper Devonian (Frasnian) Gogo Formation, Western Australia. *Hist. Biol.* **24**: 349–357 (2012).

100 Maisey, J. G. *Hamiltonichthys mapesi*, g. & sp. nov. (Chondrichthyes; Elasmobranchii), from the Upper Pennsylvanian of Kansas. *Am. Mus. Novit.* **2931**: 1–42 (1989).

101 Moy-Thomas, J. A. On the structure and affinities of the Carboniferous cochliodont *Helodus simplex*. *Geol. Mag.* **73**: 488–503 (1936).

102 Bechard, I., Arsenault, F., Cloutier, R. & Kerr, J. The Devonian placoderm fish *Bothriolepis canadensis* revisited with three-dimensional digital imagery. *Palaeontol. Electron.* **17**: 1–19 (2014).

103 Russell, L. S. Acanthodians of the Upper Devonian Escuminac Formation, Maguasha, Quebec. *Ann. Mag. Nat. Hist.* **4**: 401–407 (1951).

104 Mutter, R. J., De Blanger, K. & Neuman, A. G. Elasmobranchs from the Lower Triassic Sulphur Mountain Formation near Wapiti Lake (BC, Canada). *Zool. J. Linn. Soc.* **149**: 309–337 (2007).

105 Mutter, R. J., Neuman, A. G. & De Blanger, K. *Homalodontus* nom. nov., a replacement name for *Wapitiodus* Mutter, de Blanger and Neuman, 2007 (Homalodontidae nom. nov.,? Hybodontoidea), preoccupied by *Wapitiodus* Orchard, 2005. *Zool. J. Linn. Soc.* **154**: 419–420 (2008).

106 Long, J. A. New palaeoniscoid fishes from the Late Devonian and Early Carboniferous of Victoria. *Mem. Assoc. Australas. Palaeontol.* **7**: 1–64 (1988).

107 Dennis, K. and Miles, R. S. A pachyosteomorph arthrodire from Gogo, Western Australia. *Zool. J. Linn. Soc.* **73**: 213–258 (1981).

108 Giles, S., Rücklin, M. & Donoghue, P. C. J. Histology of “placoderm” dermal skeletons: Implications for the nature of the ancestral gnathostome. *J. Morphol.* **274**: 627–644 (2013).

109 Zangerl, R. and Case, G. R. Iniopterygia, a new order of chondrichthyan fishes from the Pennsylvanian of North America. *Fieldiana Zool.* **6**: 1–67 (1973).

110 Burrow, C. J. *et al.* The early Devonian ischnacanthiform acanthodian *Ischnacanthus gracilis* (Egerton, 1861) from the Midland Valley of Scotland. *Acta Geol. Pol.* **68**(3), 335–362 (2018).

111 Stensiö, E. A. in *Traité de Paléontologie* (ed. Piveteau, J.) 71–692 (Masson, 1969)

112 Young, G. C. The relationships of placoderm fishes. *Zool. J. Linn. Soc.* **88**: 1–57 (1986).

113 Giles, S., Friedman, M. and Brazeau, M.D. Osteichthyan-like cranial conditions in an Early Devonian stem gnathostome. *Nature* **520**: 82–85 (2015).

114 Poplin, C. *Kansasiella* nomen novum remplacant *Kansasia* Poplin 1974 (Poissons: Palaeonisciformes). *Bull. Soc. Géol. Fr* **17**, 26 (1975).

115 Hanke, G. F. & Wilson, M. V. H. in *Morphology, Phylogeny and Paleobiogeography of Fossil Fishes* (Eds. Elliott, D. K., Maisey, J. G., Yu, X.-B. & Miao, D.-S.) 159–182 (Verlag Dr. Friedrich Pfeil, 2010).

116 Pradel, A., Tafforeau, P., Maisey, J. G. & Janvier, P. A new Paleozoic Symmoriiformes (Chondrichthyes) from the Late Carboniferous of Kansas (USA) and cladistic analysis of early chondrichthyans. *PLoS One* **6**: e24938 (2011).

117 Chang, M.-M. & Zhu, M. A new Middle Devonian osteolepidid from Qujing, Yunnan. *Mem. Assoc. Australas. Palaeontol.* **15**: 183–198 (1993).

118 Zhu, M. & Ahlberg, P. E. The origin of the internal nostril of tetrapods. *Nature* **432**: 94–97 (2004).

119 Giles, S. and Friedman, M. Virtual reconstruction of endocast anatomy in early ray-finned fishes (Osteichthyes, Actinopterygii). *J. Paleontol.* **88**: 636–651 (2014).

120 Rayner, D. H. On the cranial structure of an early palaeoniscid, *Kentuckia* gen. nov. *Earth Environ. Sci. Trans. R. Soc. Edinb.* **62**: 58–83 (1951).

121 Mark-Kurik, E. *Kimaspis*, a new palaeacanthaspid from the Early Devonian of Central Asia. *Eesti NSV Teaduste Akadeemia Toimetised, Geoloogia* **22**: 322–330 (1973).

122 Bystrow, A. P. *Kolymaspis sibirica* g. n., s. n., a new representative of the Lower Devonian Agnatha. *Vestn. Leningr. Univ. Geol. Geogr.* **18**: 5–13 (1956).

123 Denison, R. H. in Handbook of Paleoichthyology, vol. 2. (ed. Schultze, H.-P.) 1–128 (Gustav Fischer Verlag, 1978).

124 Gross, W. Arthrodiren aus dem Obersilur der Prager Mulde. *Palaeontogr. Abt. A* **113**: 1–35 (1959).

125 Dupret, V. Revision of the genus *Kujdanowiaspis* Stensiö, 1942 (Placodermi, Arthrodira, “Actinolepida”) from the Lower Devonian of Podolia (Ukraine). *Geodiversitas* **32**: 5–63 (2010).

126 Schultze, H.-P. & Zidek, J. Ein primitiver Acanthodier (Pisces) aus dem Unterdevon Lettlands. *Palaontol. Z* **56**: 95–105 (1982).

127 Hamel, M.-H. & Poplin, C. The braincase anatomy of *Lawrenciella schaefferi*, actinopterygian from the Upper Carboniferous of Kansas (USA). *J. Vertebr. Paleontol.* **28**: 989–1006 (2008).

128 Basden, A. M. & Young, G. C. A primitive actinopterygian neurocranium from the Early Devonian of southeastern Australia. *J. Vertebr. Paleontol.* **21**: 754–766 (2001).

129 Basden, A. M., Young, G. C., Coates, M. I. & Ritchie, A. The most primitive osteichthyan braincase? *Nature* **403**: 185–188 (2000).

130 Burrow, C. J. Form and function in scales of *Ligulalepis toombsi* Schultze, a palaeoniscoid from the Early Devonian of Australia. *Rec. West. Aust. Mus.* **27**: 175–185 (1994).

131 Burrow, C. J., Young, G. C. & Lu, J. Dermal skeleton of the stem osteichthyan *Ligulalepis* from the Lower Devonian of New South Wales (Australia). *Span. J. Palaeontol*. **38**: 23–36 (2023).

132 Pickett, J. W. *et al.* in Palaeobiogeography of Australasian faunas and floras (eds. Wright, A. J., Young, G. C., Talent, J. A. & Laurie, J. R.) 127–165 (Memoir 23 of the Association of Australian Palaeontologists, 2000).

133 Schultze, H.-P. & Märss, T. Revisiting *Lophosteus* Pander 1856, a primitive osteichthyan. *Acta. Univ. Latv.* **674**: 57–78 (2004).

134 Gross, W. *Lunaspis broilii* und *Lunaspis heroldi* aus dem Hunsrückschiefer (Unterdevon, Rheinland). *Notizbl. hess. Landesami. Bodenforsch.* **89**: 17–43 (1961).

135 Hanke, G. F. & Davis, S. P. A re-examination of *Lupopsyrus pygmaeus* Bernacsek & Dineley, 1977 (Pisces, Acanthodii). *Geodiversitas* **34**: 469–487 (2012).

136 Stensiö, E. A. On the head of the macropetalichthyids with certain remarks on the head of the other arthrodires. *Geol. Ser.* **4**: 87–197 (1925).

137 Stensiö, E. A. The brain and the cranial nerves in fossil lower craniate vertebrates. *Skr. Norske. VidenskAkad. Oslo, Mat.-Naturv. Kl.* **13**: 1–120 (1963).

138 Long, J. A., Trinajstic, K., Young, G. C. & Senden, T. Live birth in the Devonian period. *Nature* **453**: 650–652 (2008).

139 Trinajstic, K. *et al.* New morphological information on the ptyctodontid fishes (Placodermi, Ptyctodontida) from Western Australia. *J. Vertebr. Paleontol.* **32**: 757–780 (2012).

140 Zhu, M. *et al.* A primitive fish provides key characters bearing on deep osteichthyan phylogeny. *Nature* **441**: 77–80 (2006).

141 Zhu, M., Wang, W. & Yu, X.-B. in, *Morphology, Phylogeny and Paleobiogeography of Fossil Fishes* (eds. Elliott, D.K., Maisey, J.G., Yu, X.-B. & Miao, D.-S.) 199–214 (Verlag Dr. Friedrich Pfeil, 2010).

142 Burrow, C. J., den Blaauwen, J. L. & Newman, M. New information on the Early Devonian acanthodian *Mesacanthus mitchelli* from the Midland Valley of Scotland. *Scot. J. Geol.* **58**: sjg2021-004 (2022).

143 Hemmings, S. K. The Old Red Sandstone antiarchs of Scotland: *Pterichthyodes* and *Microbrachius*. *Palaeontogr. Soc. Monogr.* **131**: 1–64 (1978).

144 Long J. *et al.* Copulation in antiarch placoderms and the origin of gnathostome internal fertilization. *Nature* **517**: 196–199 (2015).

145 Cloutier, R. in *Devonian Fishes and Plants of Miguasha, Quebec, Canada* (eds. Schultze, H.-P. & Cloutier, R.) 227–247 (Verlag Dr. Freidrich Pfeil, 1996).

146 Gardiner, B. G. The relationships of the palaeoniscid fishes, a review based on new specimens of *Mimia* and *Moythomasia* from the Upper Devonian of Western Australia. *Bull. Br. Mus. Nat. Hist. Geol. Suppl.* **37**: 173–428 (1984).

148 Zhu, M. & Janvier, P. A small antiarch, *Minicrania lirouyii* gen. et sp. nov., from the Early Devonian of Qujing, Yunnan (China), with remarks on antiarch phylogeny. *J. Vertebr. Paleontol.* **16**: 1–15 (1996).

149 Janvier, P. & Clément, G. A new groenlandaspidid arthrodire (Vertebrata: Placodermi) from the Famennian of Belgium. *Geol. Belg.* **8**: 51–67 (2005).

150 Ritchie, A. A new genus and two new species of groenlandaspidid arthrodire (Pisces: Placodermi) from the Early-Middle Devonian Mulga Downs Group of western New South Wales, Australia *Foss. Strata.* **50**: 56–81 (2004).

151 Burrow, C. J. & Rudkin, D. Oldest near-complete acanthodian: the first vertebrate from the Silurian Bertie Formation Konservat-Lagerstätte, Ontario. *PLOS ONE* **9**: e104171 (2014).

152 Woodward, A. S. The head shield of a new macropetalichthyid fish (*Notopetalichthys hillsi*, gen. et sp. nov.) from the Middle Devonian of Australia. *J. Nat. Hist. Ser. 11* **8**: 91–96 (1941).

153 Young, G. C. Large brachythoracid arthrodires (Placoderm Fishes) from the Early Devonian of Wee Jasper, New South Wales, Australia, with a discussion of basal brachythoracid characters. *J. Vertebr. Paleontol.* **24**: 1–17 (2004).

154 Hanke, G. F. & Wilson, M. V. H. in *Recent Advances in the Origin and Early Radiation of Vertebrates* (eds. Arratia, G., Wilson, M. V. H. & Cloutier, R.)189–216 (Verlag Dr. Friedrich Pfeil, 2004).

155 Andrews, S. M. *et al.* The structure of the sarcopterygian *Onychodus jandemarrai* n. sp. from Gogo, Western Australia: with a functional interpretation of the skeleton. *Earth Environ. Sci. Trans. R. Soc. Edinb*. **96**: 197–307 (2005).

156 Coates, M. I. & Gess, R. W. A new reconstruction of *Onychoselache* Traquairi, comments on early chondrichthyan pectoral girdles and hybodontiform phylogeny. *Palaeontology* **50**: 1421–1446 (2007).

157 Dick, J. R. F. & Maisey, J.G. The Scottish Lower Carboniferous shark *Onychoselache traquairi*. *Palaeontology* **23**: 363–374 (1980).

158 Heidtke, U. Der Xenacanthidae *Orthacanthus senckenbergianus* aus dem pfälzischen Rotliegended (Unter-Perm). *Pollichia* **70**: 65–86 (1982).

159 Taverne, L. *Osorioichthys marginis*, "paleonisciform" from the Fammenian of Belgium, and the phylogeny of the Devonian actinopterygians (Pisces). *Bull. Inst. R. Sci. Nat. Belg.* **67**: 57–78 (1997).

160 Thomson, K.S. The endocranium and associated structures in the Middle Devonian rhipidistian fish *Osteolepis*. *Proc. Linn. Soc. Lond.* **176**: 181–195 (1965).

161 Westoll, T. S. On the structures of the dermal ethmoid shield of *Osteolepis*. *Geol. Mag.* **73**: 157–171 (1936).

162 Pradel, A. *et al.* Palaeozoic shark with osteichthyan-like branchial arches. *Nature* **509**: 608–611 (2014).

163 Zhu, M. Catalogue of Devonian vertebrates in China, with notes on bio-events. *Cour. Forsch. Inst. Senckenberg* **223**: 373–390 (2000).

164 Zhu, M. & Wang, J.-Q. A new macropetalichthyid from China, with special reference to the historical zoogeography of the Macropetalichthyidae (Placodermi). *Vert. PalAsiat.* **34**: 253–268 (1996).

165 White, E. I. & Toombs, H. A. The buchanosteid arthrodires of Australia. *Bull. Br. Mus. Nat. Hist. Geol.* **22**: 379–419 (1972).

166 Young, G. C. New information on the structure and relationships of *Buchanosteus* (Placodermi: Euarthrodira) from the Early Devonian of New South Wales. *Zool. J. Linn. Soc.* **66**: 309–352 (1979).

167 Zhang, G.-R., Wang, J.-Q. & Wang, N.-Z. The structure of pectoral fin and tail of Yunnanolepidoidei, with a discussion of the pectoral fin of chuchinolepids. *Vert. PalAsiat.* **39**: 1–13 (2001).

168 Zhu, M. *et al*. An antiarch placoderm shows that pelvic girdles arose at the root of jawed vertebrates. *Biol. Lett.* **8**: 453–456 (2012).

169 Burrow, C. J., Newman, M. J., Davidson, R. G. & Blaauwen, J. L. D. Redescription of *Parexus recurvus*, an Early Devonian acanthodian from the Midland Valley of Scotland. *Alcheringa* **37**: 393–414 (2013).

170 Hanke, G. F. *Paucicanthus vanelsti* gen. et sp. nov., an Early Devonian (Lochkovian) acanthodian that lacks paired fin-spines. *Can. J. Earth Sci.* **39**: 1071–1083 (2002).

171 Pan, Z.-H., Zhu, M., Zhu, Y.-A. & Jia, L.-T. A new petalichthyid placoderm from the Early Devonian of Yunnan, China. *C. R. Palevol.* **14**: 125–137 (2015).

172 Clément, G. Nouvelles données anatomiques et morphologie générale des 'Porolepidae' (Dipnomorpha, Sarcopterygii). *Rev. Paléobiol.* **9**: 193–211 (2004).

173 Jessen, H. L. Lower Devonian Porolepiformes from the Canadian Arctic with special reference to *Powichthys thorsteinssoni* Jessen. *Palaeontogr. Abt.* A **167**: 180–214 (1980).

174 Hanke, G. F. & Davis, S. P. Redescription of the acanthodian *Gladiobranchus probation* Bernacsek & Dineley, 1977, and comments on diplacanthid relationships. *Geodiversitas* **30**: 303–330 (2008).

175 Yu, X.-B. A new porolepiform-like fish, *Psarolepis romeri*, gen. et sp. nov. (Sarcopterygii, Osteichthyes) from the Lower Devonian of Yunnan, China. *J. Vertebr. Paleontol.* **18**: 261–274 (1998).

176 Zhu, M., Yu, X.-B. & Janvier, P. A primitive fossil fish sheds light on the origin of bony fishes. *Nature* **397**: 607–610 (1999).

177 Brazeau, M. D. A revision of the anatomy of the Early Devonian jawed vertebrate *Ptomacanthus anglicus* Miles. *Palaeontology* **55**: 355–367 (2012).

178 Dineley, D. L. in *Fossil Fishes of Great Britain* (eds. Dineley D. L. & Metcalf, S.J.) 1–29 (Joint Nature Conservation Committee, Peterborough, 1999).

179 Lu, J., Giles, S., Friedman, M. & Zhu, M. A new stem sarcopterygian illuminates patterns of character evolution in early bony fishes. *Nat. Commun.* **8**: 1932 (2017).

180 Maisey, J. G. & Anderson, M. E. A primitive chondrichthyan braincase from the Early Devonian of South Africa. *J. Vertebr. Paleontol.* **21**: 702–713 (2001).

181 Zhu, M. et al. A Silurian maxillate placoderm illuminates jaw evolution. *Science* **354**: 334–336 (2016).

182 Lu, J. & Zhu, M. An onychodont fish (Osteichthyes, Sarcopterygii) from the Early Devonian of China, and the evolution of the Onychodontiformes. *Proc. R. Soc. B* **277**: 293–299 (2010).

183 Lu, J. *et al.* A Devonian predatory fish provides insights into the early evolution of modern sarcopterygians. *Sci. Adv.* **2**: e1600154 (2016).

184 Vaškaninová, V. & Ahlberg, P.E. Unique diversity of acanthothoracid placoderms (basal jawed vertebrates) in the Early Devonian of the Prague Basin, Czech Republic: a new look at *Radotina* and *Holopetalichthys*. *PLoS One* **12**: e0174794 (2017).

185 Gross, W. Über die älterste Arthrodiren-Gattung. *Notizbl. hess. Landesami. Bodenforsch.* **86**: 7–30 (1958).

186 Pradel, A., Maisey, J. G., Tafforeau, P. & Janvier, P. An enigmatic gnathostome vertebrate skull from the Middle Devonian of Bolivia. *Acta Zool.* **90**: 123–133 (2009).

187 Giles, S. *et al.* An exceptionally preserved Late Devonian actinopterygian provides a new model for primitive cranial anatomy in ray-finned fishes. *Proc. R Soc. B* **282**: 20151485 (2015).

188 Andrews, S. M. A possible occurrence of *Remigolepis* in the topmost Old Red Sandstone of Berwickshire. *Scott. J. Geol.* **14**: 311–315 (1978).

189 Miles, R. S. Observations on the ptyctodont fish, *Rhamphodopsis* Watson. *Zool. J. Linn. Soc.* **47**: 99–120 (1967).

190 Dupret, V. *et al.* A primitive placoderm sheds light on the origin of the jawed vertebrate face. *Nature* **507**: 500–503 (2014).

191 Ørvig, T. in *Problèmes actuels de Paléontologie-Evolution des Vertébrés* (ed. Lehman, J. P.) 41–71 (Colloques Internationaux du Centre National de la Recherche Scientifique, 1975).

192 Young, G. C. Further petalichthyid remains (placoderm fishes, Early Devonian) from the Taemas-Wee Jasper region, New South Wales. *BMR J. Aust. Geol. Geophys.* **9**: 121–131 (1985).

193 Zhu, Y.-A. *et al.* The oldest complete jawed vertebrates from the early Silurian of China. *Nature* **609**: 954–958 (2022).

194 Goujet, D. F. *Sigaspis*, un nouvel arthrodire du Dévonien inférieur du Spitsberg. *Palaeontogr. Abt. A* **143**: 73–88 (1973).

195 Liu, T.-S. & P'an, K. Devonian fishes from Wutung Series near Nanking, China. *Palaeontogr. Sin. C,* **141**: 1–41 (1958).

196 Ritchie, A., Wang, S.-T., Young, G. C. & Zhang, G.-R. The Sinolepidae, a family of antiarchs (placoderm fishes) from the Devonian of South China and eastern Australia. *Rec. Aust. Mus.* **44**: 319–370 (1992).

197 Zhao, W.-J. & Zhu, M. Siluro-Devonian vertebrate biostratigraphy and biogeography of China. *Palaeoworld* **19**: 4–26 (2010).

198 Choo, B. *et al.* A new osteichthyan from the late Silurian of Yunnan, China. *PLoS One* **12**: e0170929 (2017).

199 Gans, C. & Parsons, T. S. *A photographic atlas of shark anatomy: the gross morphology of Squalus acanthias*. (Academic Press, 1964).

200 Friedman, M. *Styloichthys* as the oldest coelacanth: implications for early osteichthyan interrelationships. *J. Syst. Palaeontol.* **5**: 289–343 (2007).

201 Zhu, M. & Yu, X.-B. A primitive fish close to the common ancestor of tetrapods and lungfish. *Nature* **418**: 767–770 (2002).

202 Maisey, J. G. Cranial morphology of the fossil elasmobranch *Synechodus dubrisiensis*. *Am. Mus. Novit.* **2804**: 1–28 (1985).

203 Williams, M. E. A new specimen of *Tamiobatis vetustus* (Chondrichthyes, Ctenacanthoidea) from the Late Devonian Cleveland Shale of Ohio. *J. Vertebr. Paleontol.* **18**: 251–260 (1998).

204 Gagnier, P. Y., Hanke, G. F. & Wilson, M. V. H. *Tetanopsyrus lindoei* gen. et sp. nov., an Early Devonian acanthodian from the Northwest Territories, Canada. *Acta Geol. Pol.* **49**: 81–96 (1999).

205 Hanke, G. F., Davis, S. P. & Wilson, M. V. H. New species of the acanthodian genus *Tetanopsyrus* from northern Canada, and comments on related taxa. *J. Vertebr. Paleontol.* **21**: 740–753 (2001).

206 Lane, J. A. Morphology of the braincase in the Cretaceous hybodont shark *Tribodus limae* (Chondrichthyes: Elasmobranchii), based on CT scanning. *Am. Mus. Novit.* **3681**: 1–70 (2010).

207 Lane, J. A. & Maisey, J. G. Pectoral anatomy of *Tribodus limae* (Elasmobranchii: Hybodontiformes) from the Lower Cretaceous of northeastern Brazil. *J. Vertebr. Paleontol.* **29**: 25–38 (2009).

208 Lane, J. A. & Maisey, J. G. The visceral skeleton and jaw suspension in the durophagous hybodontid shark *Tribodus limae* from the Lower Cretaceous of Brazil. *J. Paleontol.* **86**: 886–905 (2012).

209 Maisey, J. G. & Denton, J. S. S. Dermal denticle patterning in the Cretaceous hybodont shark *Tribodus limae* (Euselachii, Hybodontiformes), and its implications for the evolution of patterning in the chondrichthyan dermal skeleton. *J. Vertebr. Paleontol.* **36**: 5, e1179200 (2016).

210 Heidtke, U. H. J., Schwind, C. & Krätschmer, K. Über die Organisation des Skelettes und die verwandschaftlichen Beziehungen der Gattung Triodus Jordan 1849 (Elasmobranchii: Xenacanthida). *Mainz. Geowiss. Mitt.* **32**: 9–54 (2004).

211 Soler-Gijon, R. & Hampe, O. Evidence of *Triodus* Jordan 1849 (Elasmobranchii: Xenacanthidae) in the Lower Permian of the Autun Basin (Muse, France). *Neues Jahrb. Geol. Paläontol., Monatsh.* **6**: 335–348 (1998).

212 Dick, J. R. F. On the Carboniferous shark *Tristychius arcuatus* Agassiz from Scotland. *Earth Environ. Sci. Trans. R. Soc. Edinb.* **70**: 63–109 (1978).

213 Newman, M. J., Davidson, R. G., Blaauwen, J. L. D. & Burrow, C. J. The Early Devonian acanthodian *Uraniacanthus curtus* (Powrie, 1870) n. comb. from the Midland Valley of Scotland. *Geodiversitas* **34**: 739–759 (2012).

214 Hanke, G. F. *Promesacanthus eppleri* n. gen., n. sp., a mesacanthid (Acanthodii, Acanthodiformes) from the Lower Devonian of northern Canada. *Geodiversitas*, **30**: 287–302 (2008).

215 Denison, R. H. Early Devonian lungfishes from Wyoming, Utah, and Idaho. *Fieldiana, Geol.* **17**: 353–413 (1968).

216 Barker, C. E. & Bone, Y. The minimal response to contact metamorphism by the Devonian Buchan Caves Limestone, Buchan Rift, Victoria, Australia. *Org. Geochem.* **22**: 151–164 (1995).

217 Basden, A. M. Emsian (Early Devonian) microvertebrates from the Buchan and Taemas areas of southeastern Australia. *Rec. West. Aust. Mus. Suppl.* **57**: 15–21 (1999).

218 Ritchie, A. *Wuttagoonaspis* gen. nov., an unusual arthrodire from the Devonian of Western New South Wales, Australia. *Palaeontogr. Abt. A* **143**: 58–72 (1973).

219 Chang, M.-M., 1982. *The braincase of Youngolepis, a Lower Devonian crossopterygian from Yunnan, south-western China* (University of Stockholm, Department of Geology, 1982).

220 Chang, M.-M. in *Early Vertebrates and Related Problems of Evolutionary Biology* (eds. Chang, M.-M., Liu, Y.-H. & Zhang, G.-R.) 355–378 (Science Press, 1991).

221 Chang, M.-M. & Yu, X.-B. A new crossopterygian, *Youngolepis praecursor*, gen. et sp. nov., from Lower Devonian of eastern Yunnan, China. *Sci. Sin.* **24**: 89–99 (1981).

222 Zhang, M.-M. Preliminary note on a Lower Devonian antiarch from Yunnan, China. *Vert. PalAsiat.* **18**: 179–190 (1980).

223 Zhu, M. The phylogeny of the Antiarcha (Placodermi, Pisces), with the description of Early Devonian antiarchs from Qujing, Yunnan, China. *Bull. Mus. Natl. Hist. Nat.* **18**: 233–347 (1996).

224 Valiukevicius, J. in *Fossil Fishes as Living Animals* (ed. Mark-Kurik, E.) 193–214 (Academy of Sciences of Estonia, 1992).

225 Vergoossen, J. M. J., Ivanov, A., Wilson, M. V. H. & Zhuravlev, A., 1997. in *Palaeozoic strata and fossils of the Eurasian Arctic* (eds. Ivanov, A., Wilson, M.V.H. & Zhuravalov, A.) 44–46 (Ichthyolith Issues Special Publication,1997)

226 Wang, Y. & Zhu, M. Squamation and scale morphology at the root of jawed vertebrates. *eLife* **11**, e76661 (2022).

227 Chen, D., Janvier, P., Ahlberg, P. E. & Blom, H. Scale morphology and squamation of the late Silurian osteichthyan *Andreolepis* from Gotland, Sweden. *Hist. Biol.* **24**, 411–423 (2012).

228 Jarvik, E. On the morphology and taxonomy of the Middle Devonian osteolepid fishes of Scotland. *K. Svenska VetenskAkad. Handl.* **3**: 1–301 (1948).

229 Jessen, H. L. *Moythomasia nitida* Gross und *M*. cf. *striata* Gross, Devonische Palaeonisciden aus dem oberen Plattenkalk der Bergisch-Gladbach - Paffrather Mudle (Rheinisches Schiefergebirge). *Palaeontogr. Abt. A* **128**, 87–114 (1968).

230 Gemballa, S. & Bartsch, P. Architecture of the integument in lower teleostomes: Functional morphology and evolutionary implications. *J. Morphol.* **253**, 290–309 (2002).
