# Supplementary material for: Bony-fish-like scales in a Silurian maxillate placoderm
Source: Nat Commun. 2023 Nov 22;14:7622. doi: 10.1038/s41467-023-43557-9 (PMC10665347; doi:10.1038/s41467-023-43557-9)
Supplement: Supplementary file 3 — Description of Additional Supplementary Files [file 41467_2023_43557_MOESM3_ESM.pdf]

Description of Additional Supplementary Files

File Name: Supplementary Data 1
Description: Matrix for phylogenetic analyses

File Name: Supplementary Data 2
Description: Raw data of Geometric Morphometric Analyses

File Name: Supplementary Data 3
Description: Landmarks of Geometric Morphometric Analyses

File Name: Supplementary Data 4
Description: Measurement data of scales

File Name: Supplementary Movie 1
Description: Video of the 3D reconstruction of *Entelognathus primordialis*.

File Name: Supplementary Code 1
Description: : Geometric Morphometric Analyses R
