# Supplementary figures and images for: Bony-fish-like scales in a Silurian maxillate placoderm (part 2 of 2)
Source: Nat Commun. 2023 Nov 22;14:7622. doi: 10.1038/s41467-023-43557-9 (PMC10665347; doi:10.1038/s41467-023-43557-9)

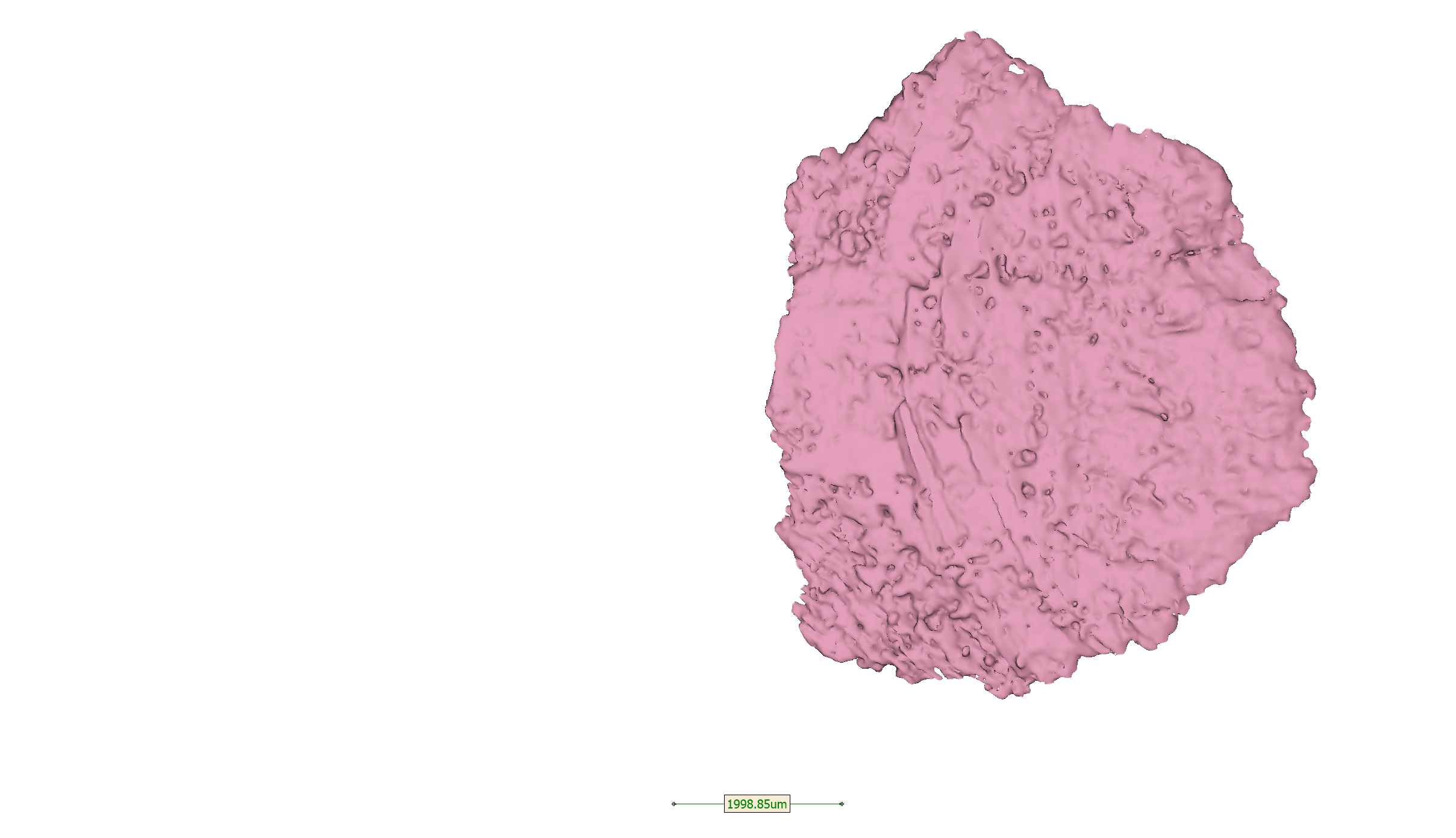

Supplement: Supplementary file 5 — Supplementary Data 2 [file 41467_2023_43557_MOESM5_ESM.zip › Supplementary Data 2/Supplementary Data 2 Raw data of Geometric Morphometric Analyses/12 Morphotypes/Morphotype 3/l1d11.jpg]

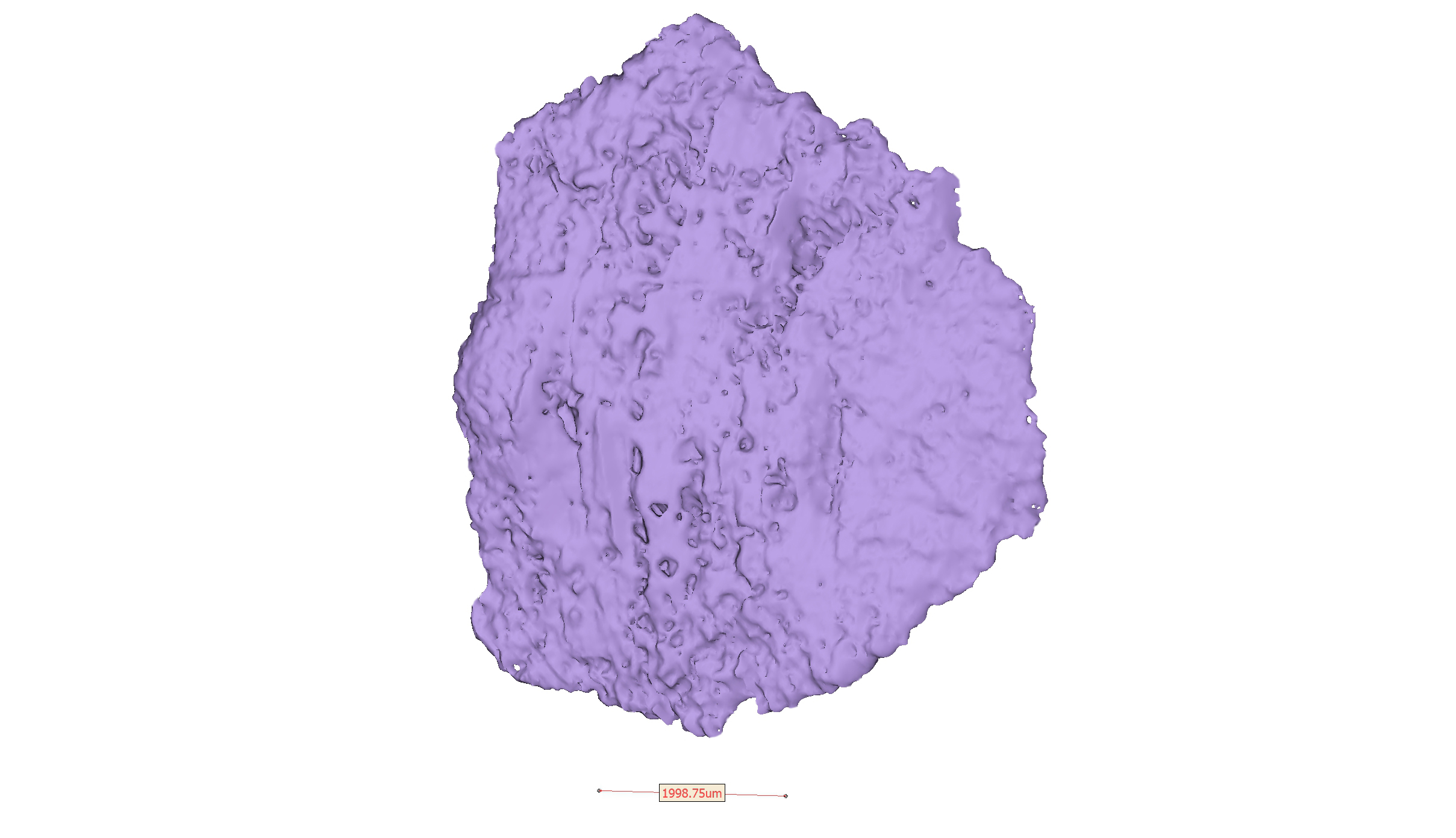

Supplement: Supplementary file 5 — Supplementary Data 2 [file 41467_2023_43557_MOESM5_ESM.zip › Supplementary Data 2/Supplementary Data 2 Raw data of Geometric Morphometric Analyses/12 Morphotypes/Morphotype 3/l1d12.jpg]

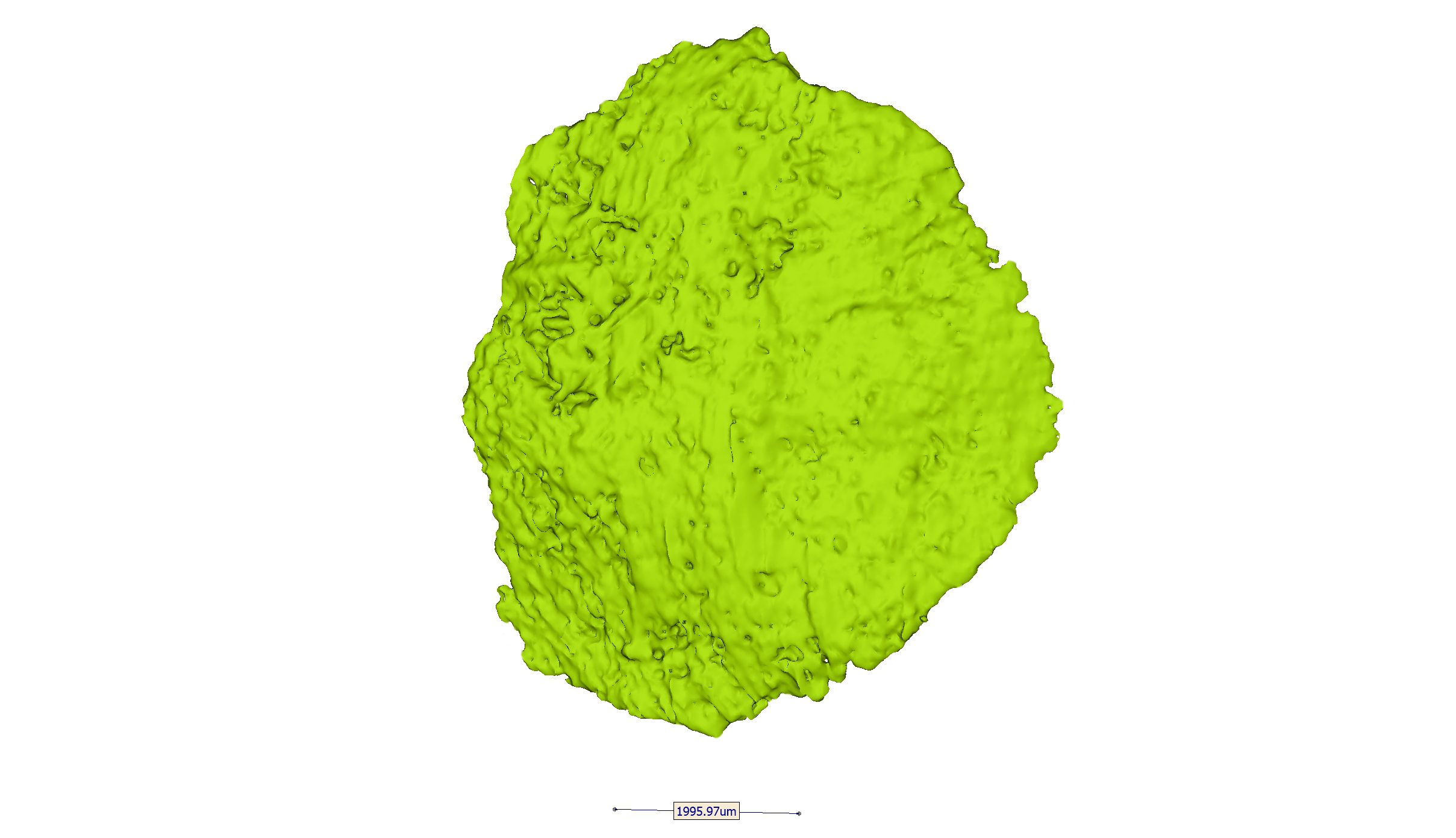

Supplement: Supplementary file 5 — Supplementary Data 2 [file 41467_2023_43557_MOESM5_ESM.zip › Supplementary Data 2/Supplementary Data 2 Raw data of Geometric Morphometric Analyses/12 Morphotypes/Morphotype 3/l1d13.jpg]

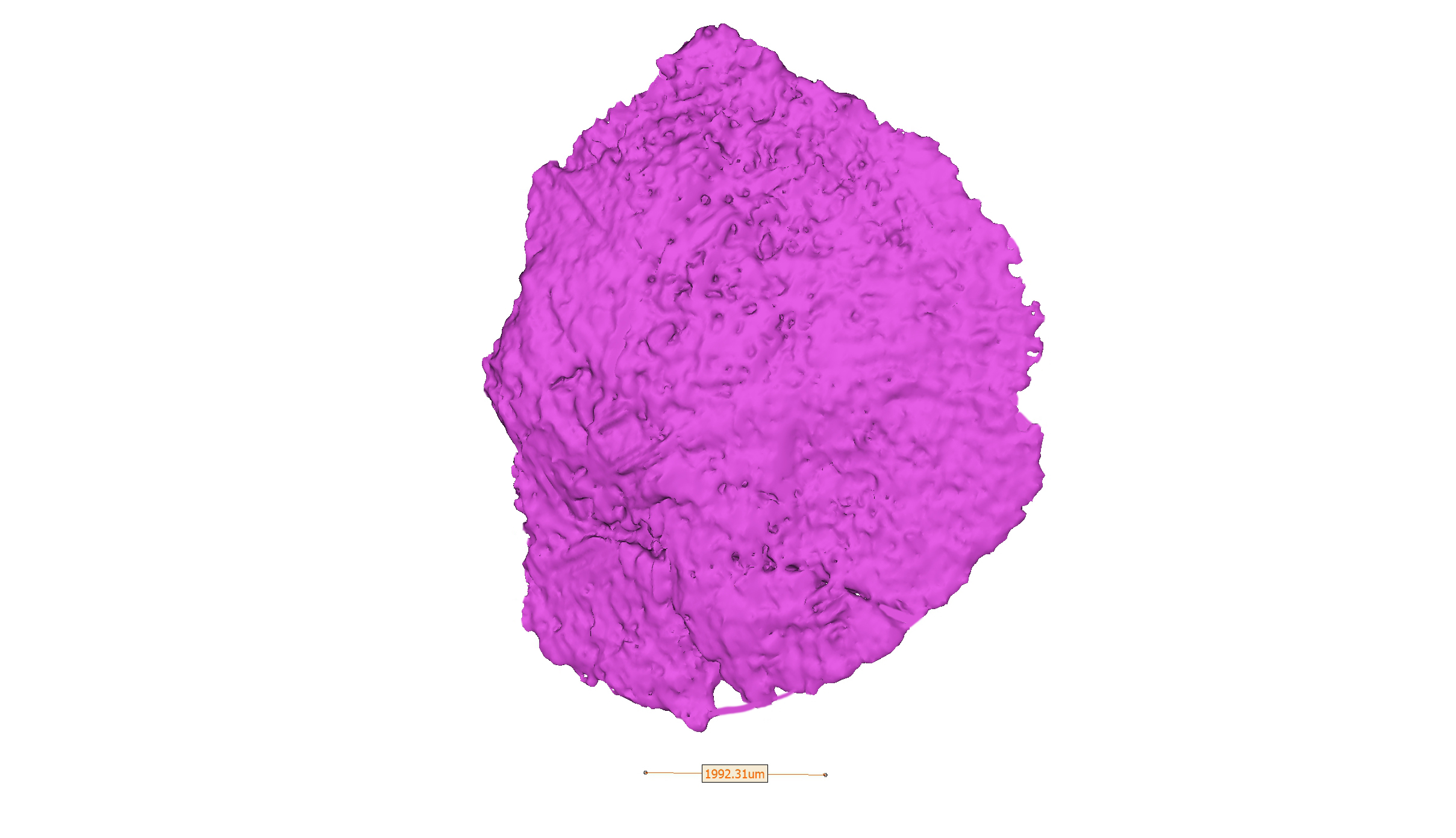

Supplement: Supplementary file 5 — Supplementary Data 2 [file 41467_2023_43557_MOESM5_ESM.zip › Supplementary Data 2/Supplementary Data 2 Raw data of Geometric Morphometric Analyses/12 Morphotypes/Morphotype 3/l1d14.jpg]

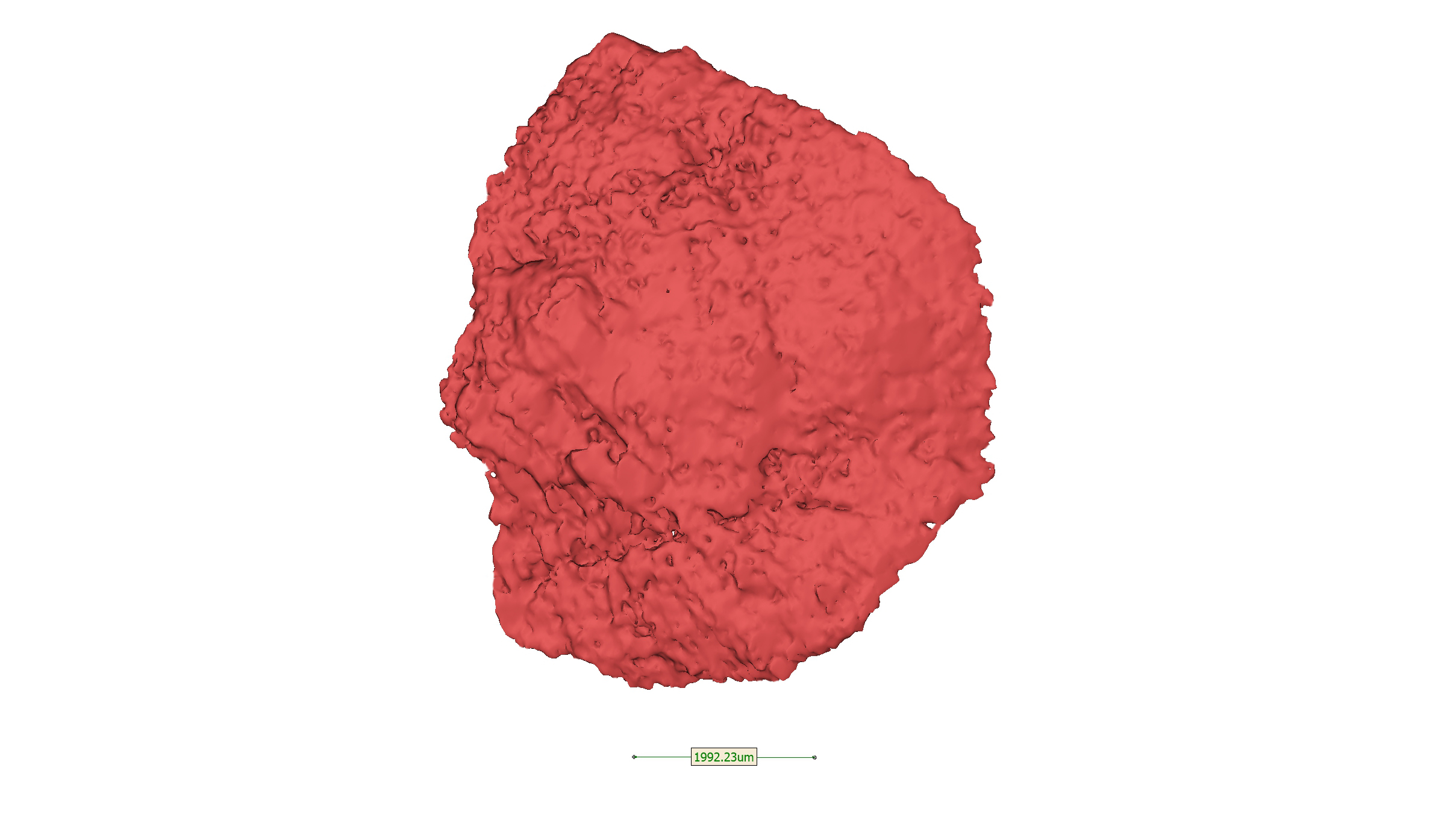

Supplement: Supplementary file 5 — Supplementary Data 2 [file 41467_2023_43557_MOESM5_ESM.zip › Supplementary Data 2/Supplementary Data 2 Raw data of Geometric Morphometric Analyses/12 Morphotypes/Morphotype 3/l1d15.jpg]

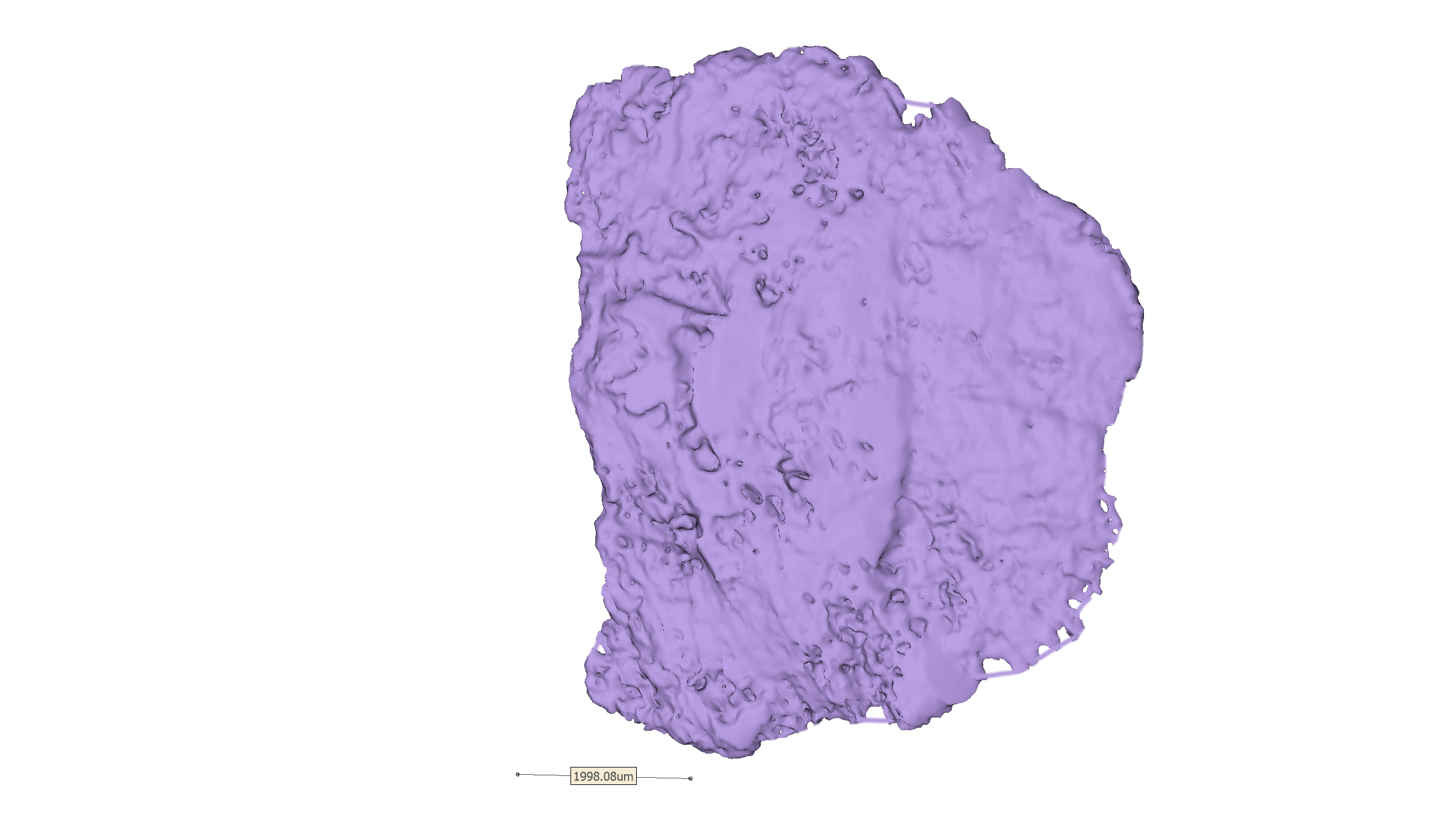

Supplement: Supplementary file 5 — Supplementary Data 2 [file 41467_2023_43557_MOESM5_ESM.zip › Supplementary Data 2/Supplementary Data 2 Raw data of Geometric Morphometric Analyses/12 Morphotypes/Morphotype 3/l1v09.jpg]

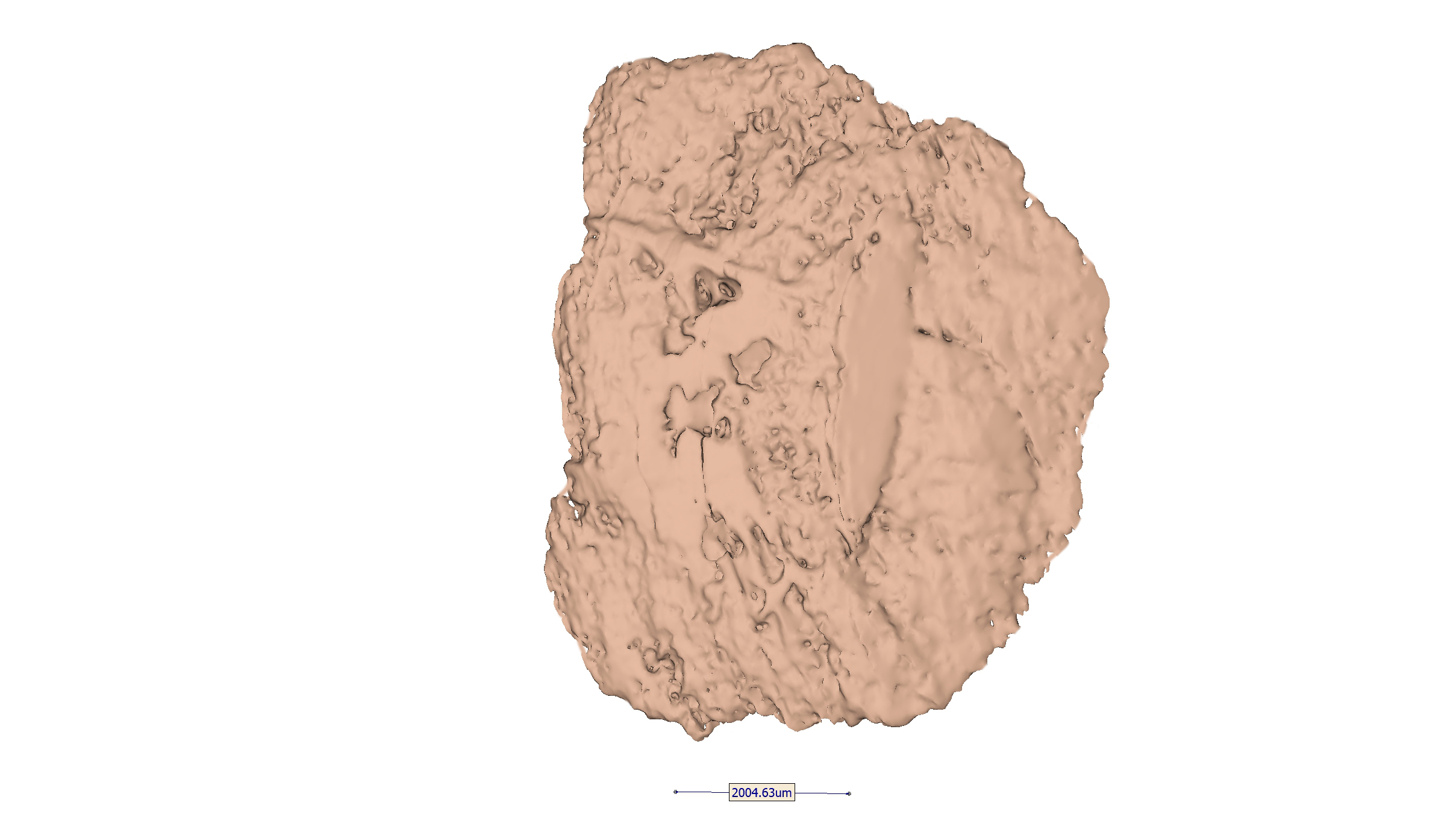

Supplement: Supplementary file 5 — Supplementary Data 2 [file 41467_2023_43557_MOESM5_ESM.zip › Supplementary Data 2/Supplementary Data 2 Raw data of Geometric Morphometric Analyses/12 Morphotypes/Morphotype 3/l1v10.jpg]

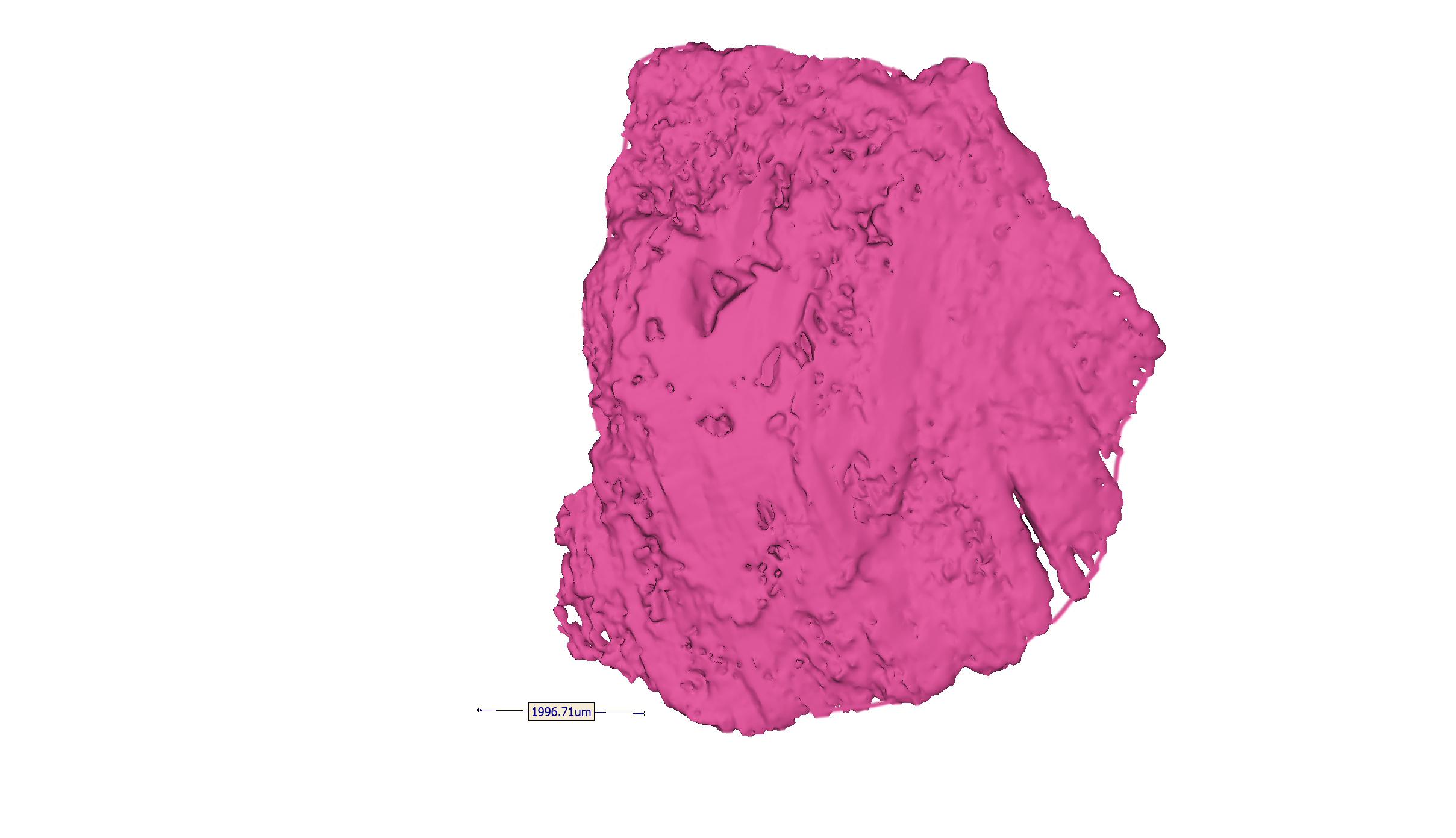

Supplement: Supplementary file 5 — Supplementary Data 2 [file 41467_2023_43557_MOESM5_ESM.zip › Supplementary Data 2/Supplementary Data 2 Raw data of Geometric Morphometric Analyses/12 Morphotypes/Morphotype 3/l1v11.jpg]

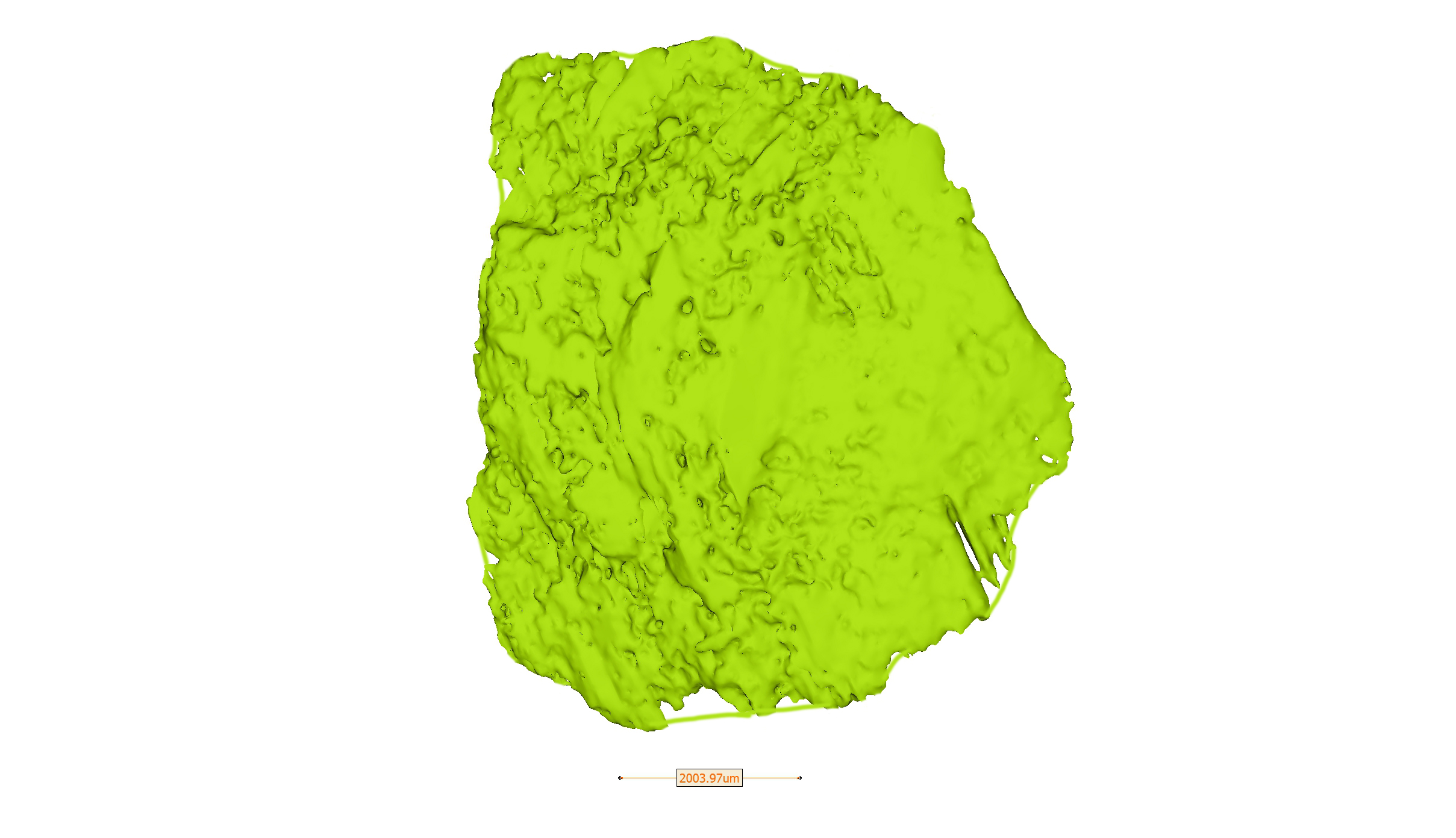

Supplement: Supplementary file 5 — Supplementary Data 2 [file 41467_2023_43557_MOESM5_ESM.zip › Supplementary Data 2/Supplementary Data 2 Raw data of Geometric Morphometric Analyses/12 Morphotypes/Morphotype 3/l1v12.jpg]

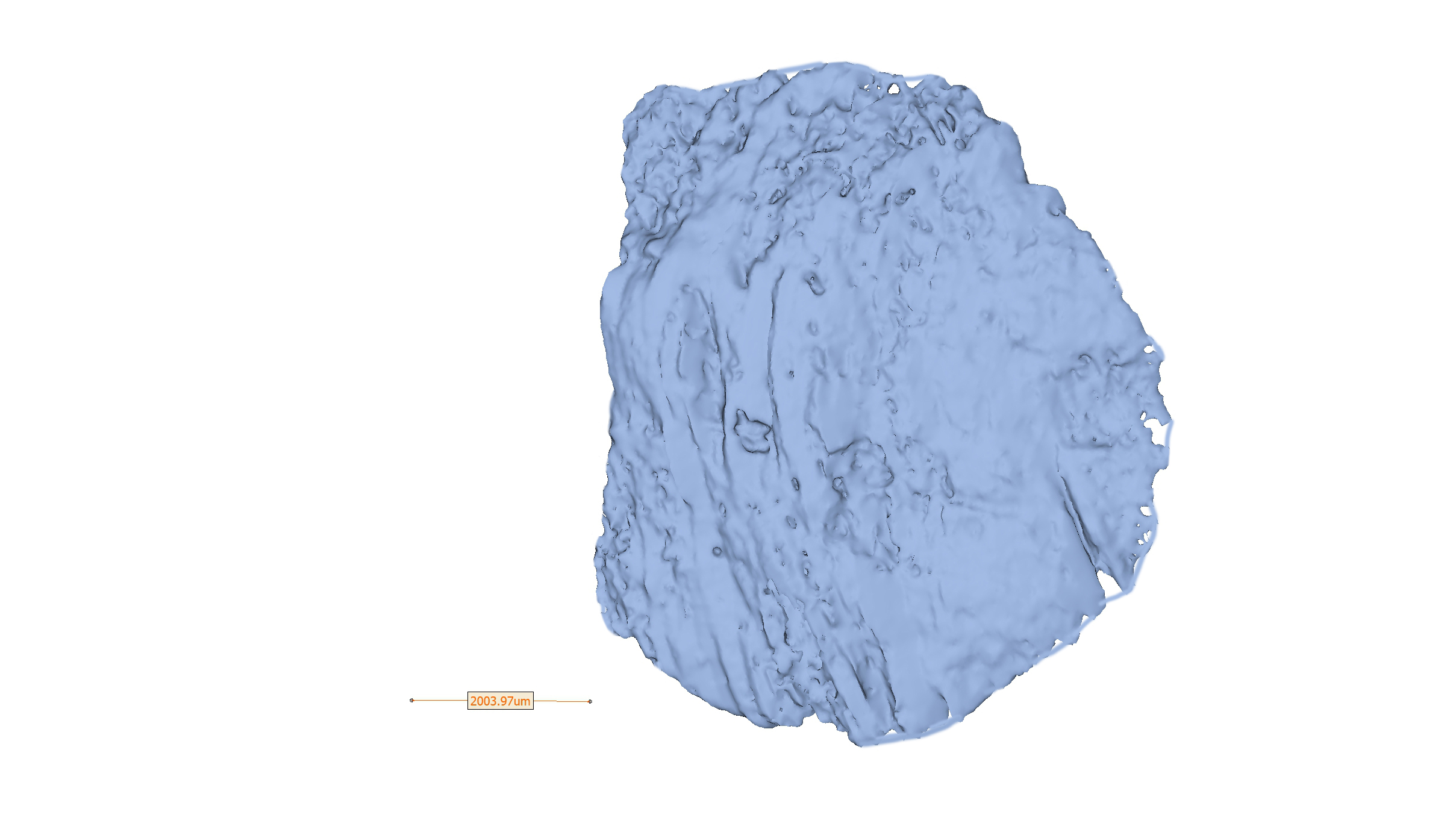

Supplement: Supplementary file 5 — Supplementary Data 2 [file 41467_2023_43557_MOESM5_ESM.zip › Supplementary Data 2/Supplementary Data 2 Raw data of Geometric Morphometric Analyses/12 Morphotypes/Morphotype 3/l1v13.jpg]

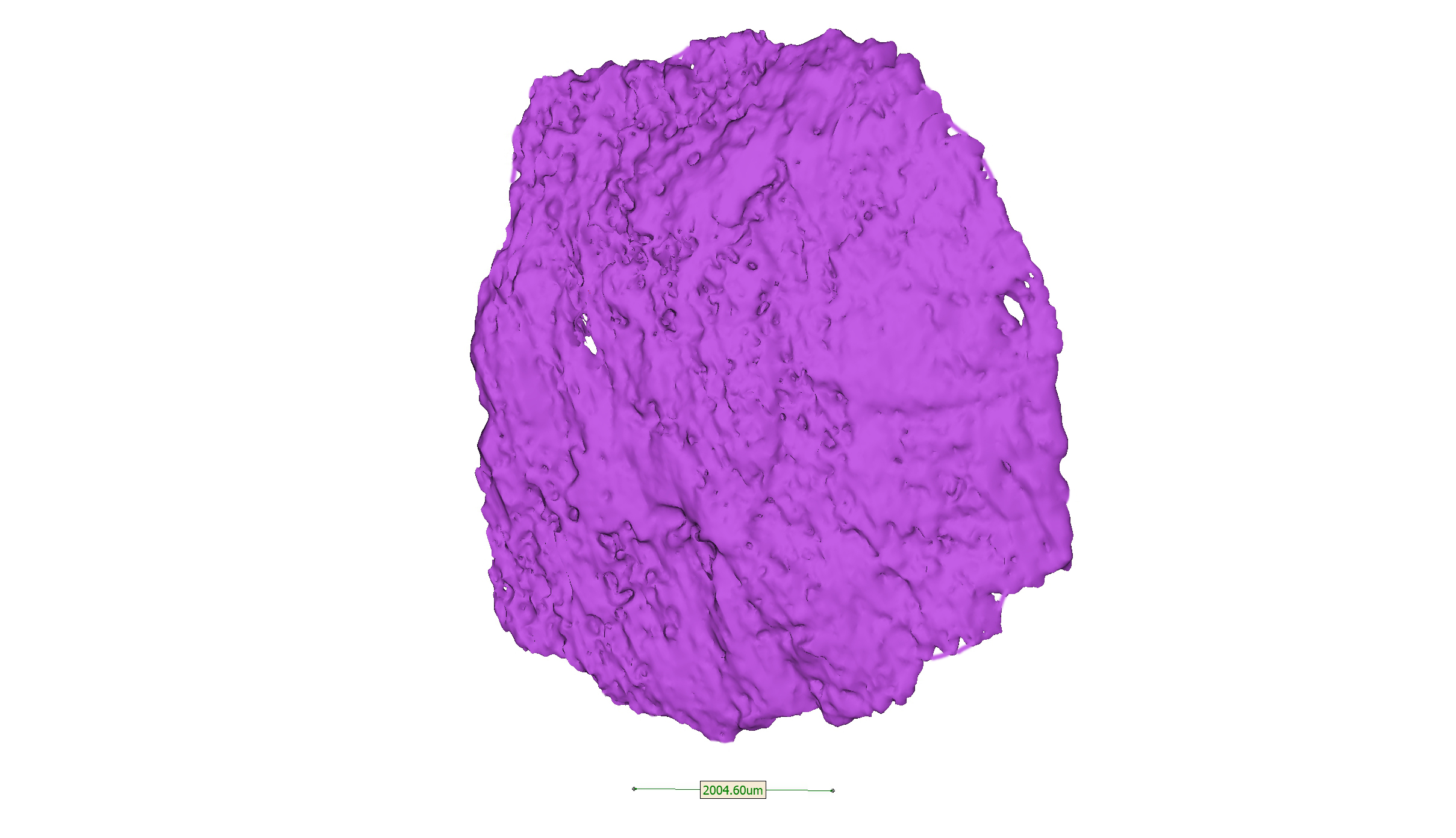

Supplement: Supplementary file 5 — Supplementary Data 2 [file 41467_2023_43557_MOESM5_ESM.zip › Supplementary Data 2/Supplementary Data 2 Raw data of Geometric Morphometric Analyses/12 Morphotypes/Morphotype 3/l1v14.jpg]

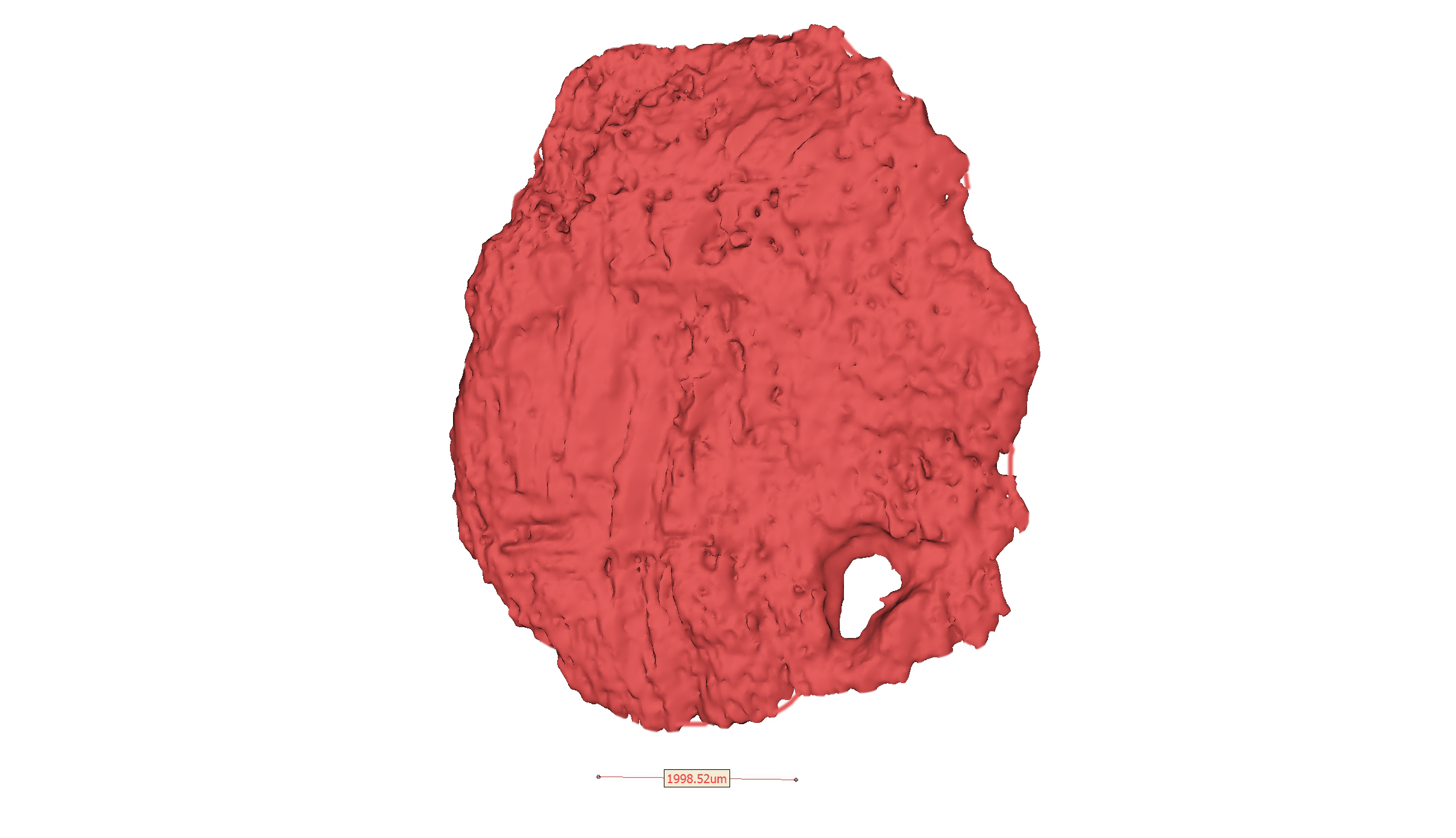

Supplement: Supplementary file 5 — Supplementary Data 2 [file 41467_2023_43557_MOESM5_ESM.zip › Supplementary Data 2/Supplementary Data 2 Raw data of Geometric Morphometric Analyses/12 Morphotypes/Morphotype 3/l1v15.jpg]

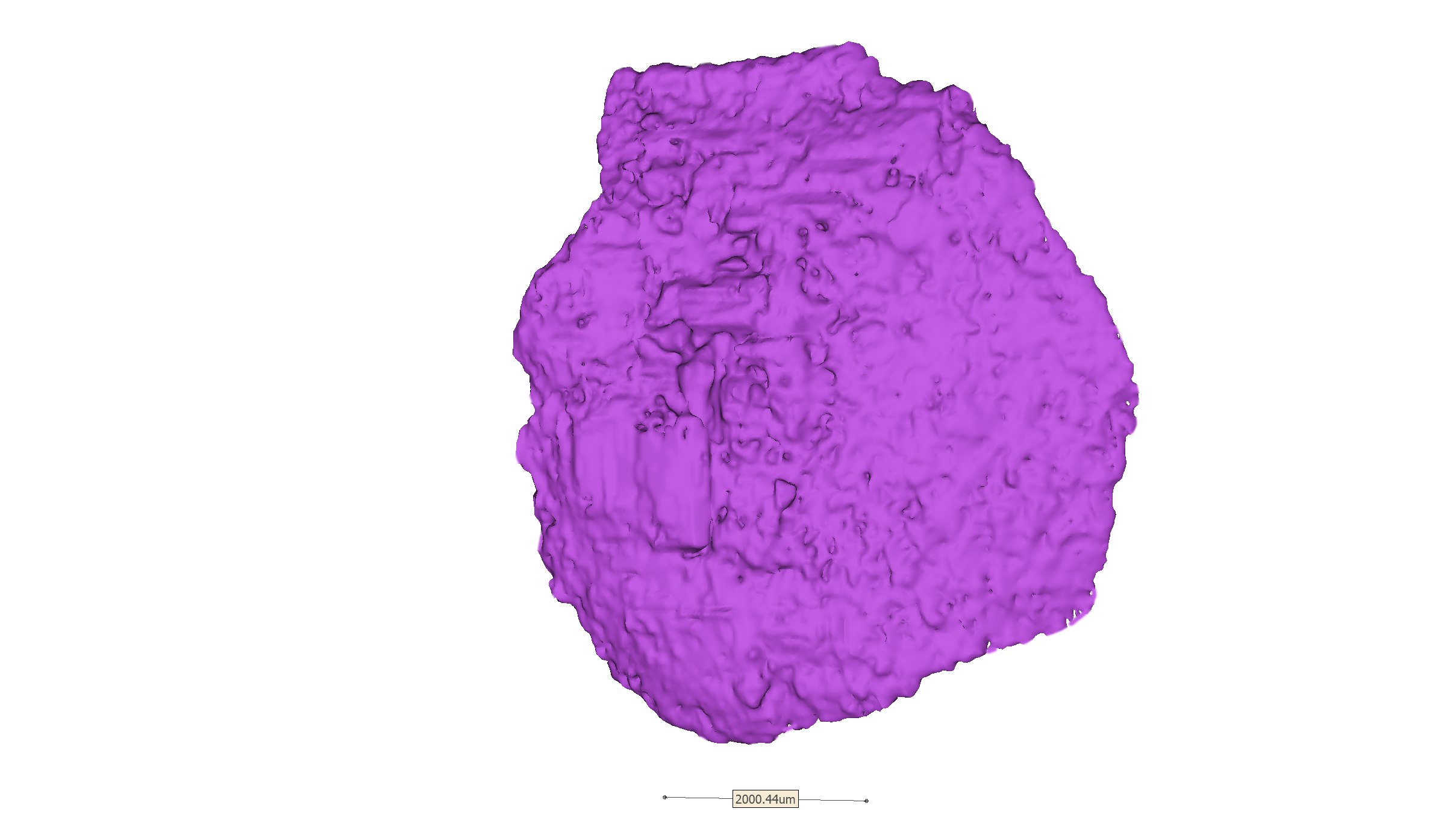

Supplement: Supplementary file 5 — Supplementary Data 2 [file 41467_2023_43557_MOESM5_ESM.zip › Supplementary Data 2/Supplementary Data 2 Raw data of Geometric Morphometric Analyses/12 Morphotypes/Morphotype 3/l1v16.jpg]

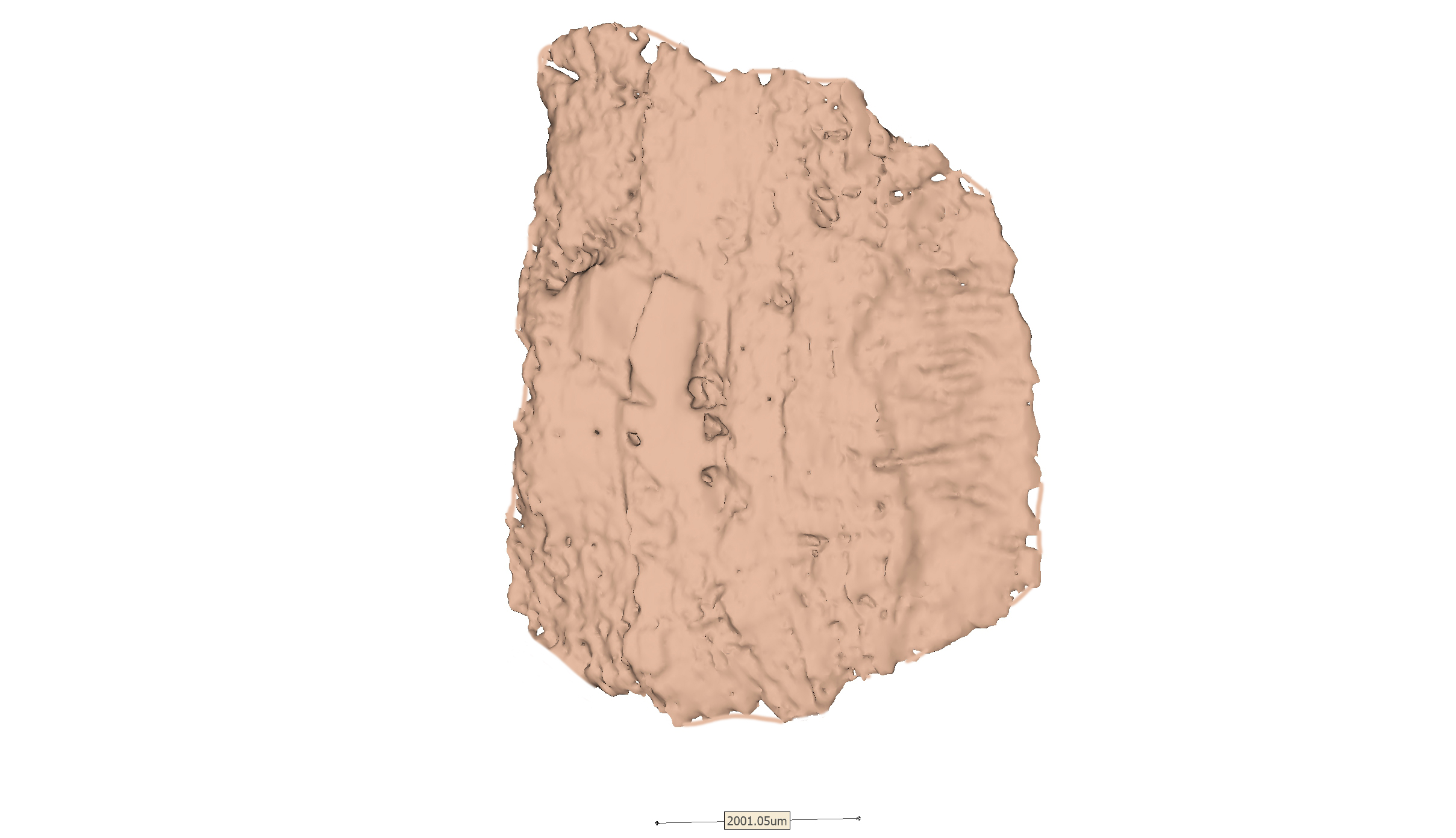

Supplement: Supplementary file 5 — Supplementary Data 2 [file 41467_2023_43557_MOESM5_ESM.zip › Supplementary Data 2/Supplementary Data 2 Raw data of Geometric Morphometric Analyses/12 Morphotypes/Morphotype 3/l2d05.jpg]

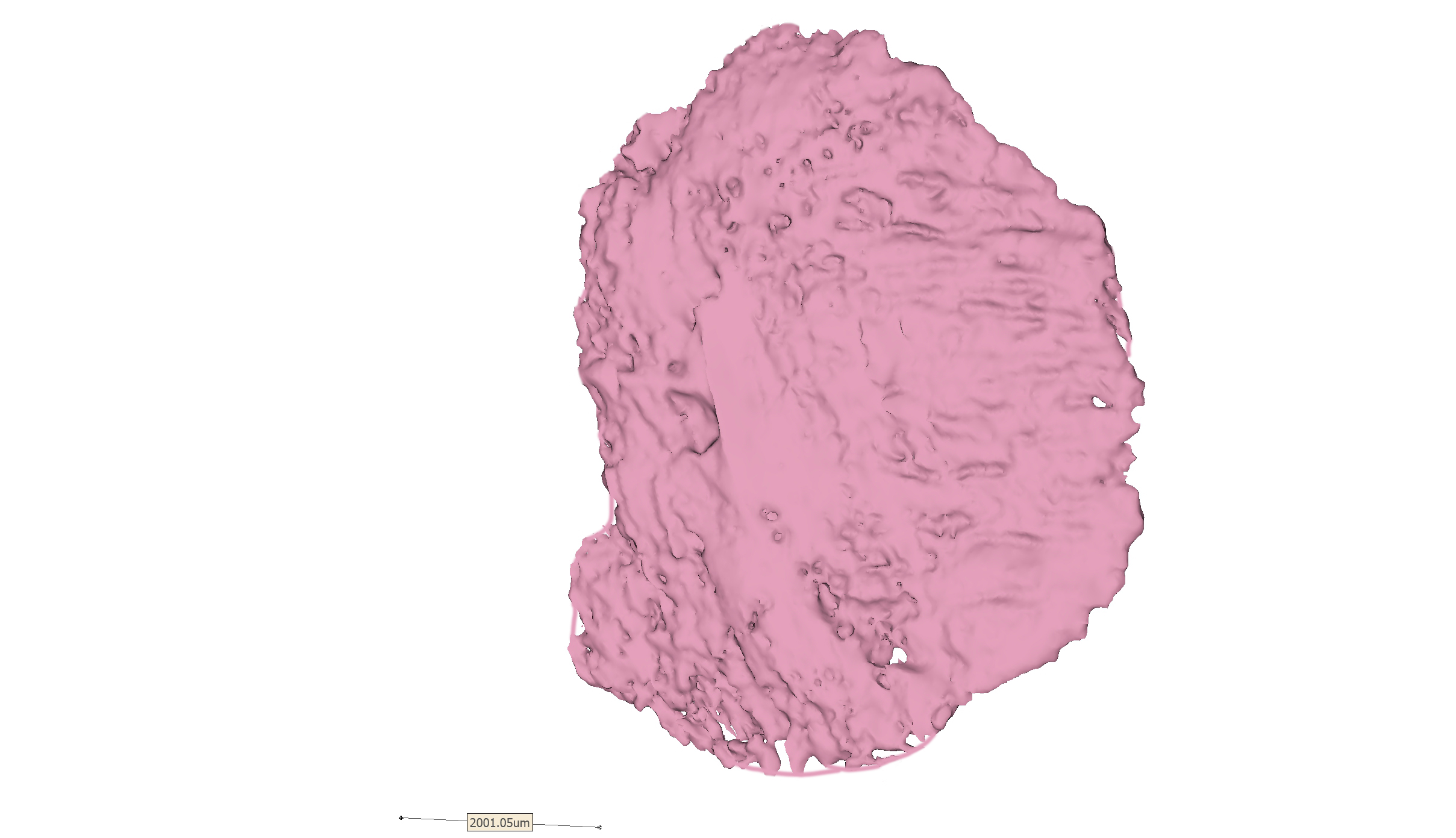

Supplement: Supplementary file 5 — Supplementary Data 2 [file 41467_2023_43557_MOESM5_ESM.zip › Supplementary Data 2/Supplementary Data 2 Raw data of Geometric Morphometric Analyses/12 Morphotypes/Morphotype 3/l2d06.jpg]

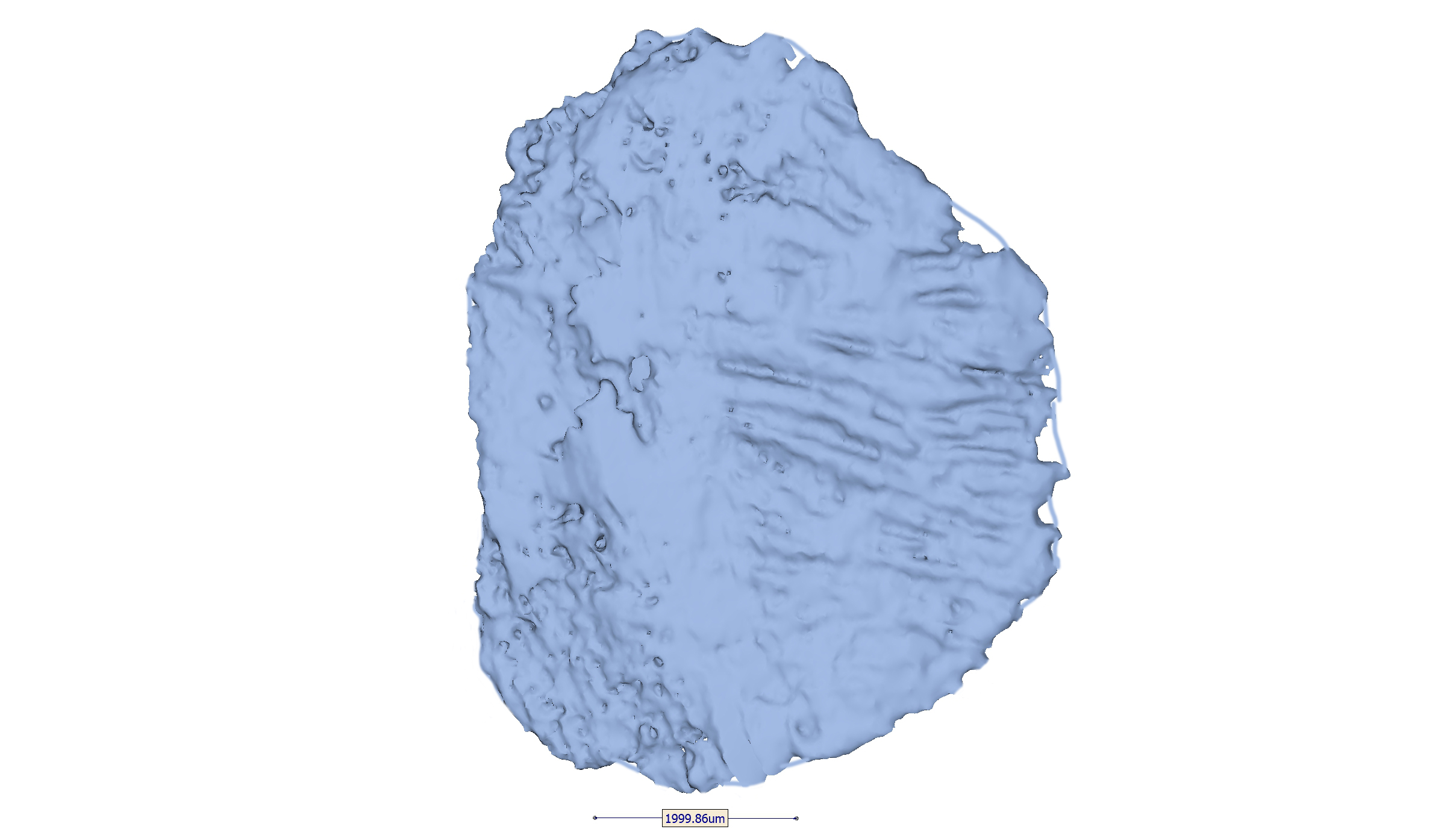

Supplement: Supplementary file 5 — Supplementary Data 2 [file 41467_2023_43557_MOESM5_ESM.zip › Supplementary Data 2/Supplementary Data 2 Raw data of Geometric Morphometric Analyses/12 Morphotypes/Morphotype 3/l2d07.jpg]

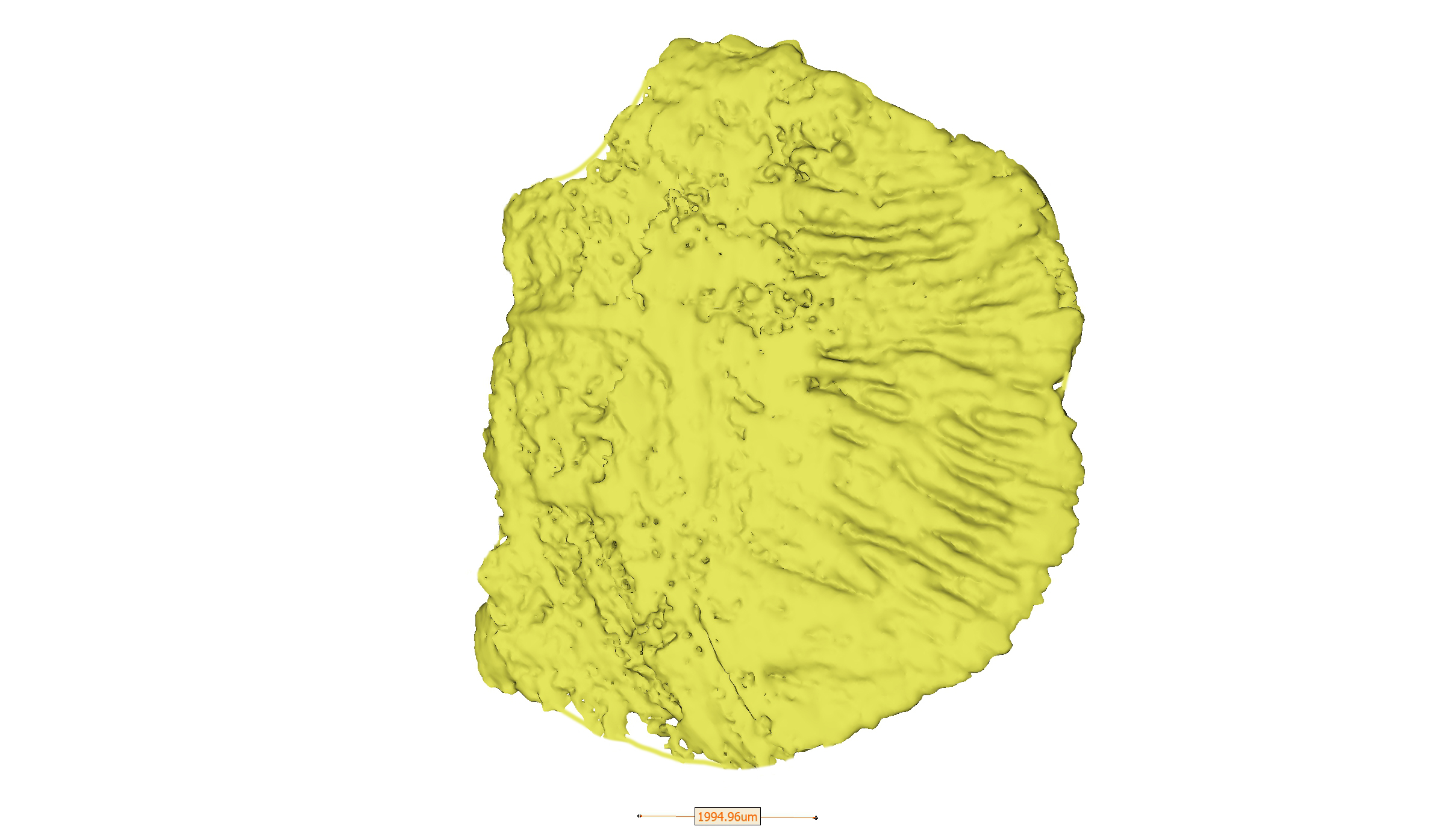

Supplement: Supplementary file 5 — Supplementary Data 2 [file 41467_2023_43557_MOESM5_ESM.zip › Supplementary Data 2/Supplementary Data 2 Raw data of Geometric Morphometric Analyses/12 Morphotypes/Morphotype 3/l2d08.jpg]

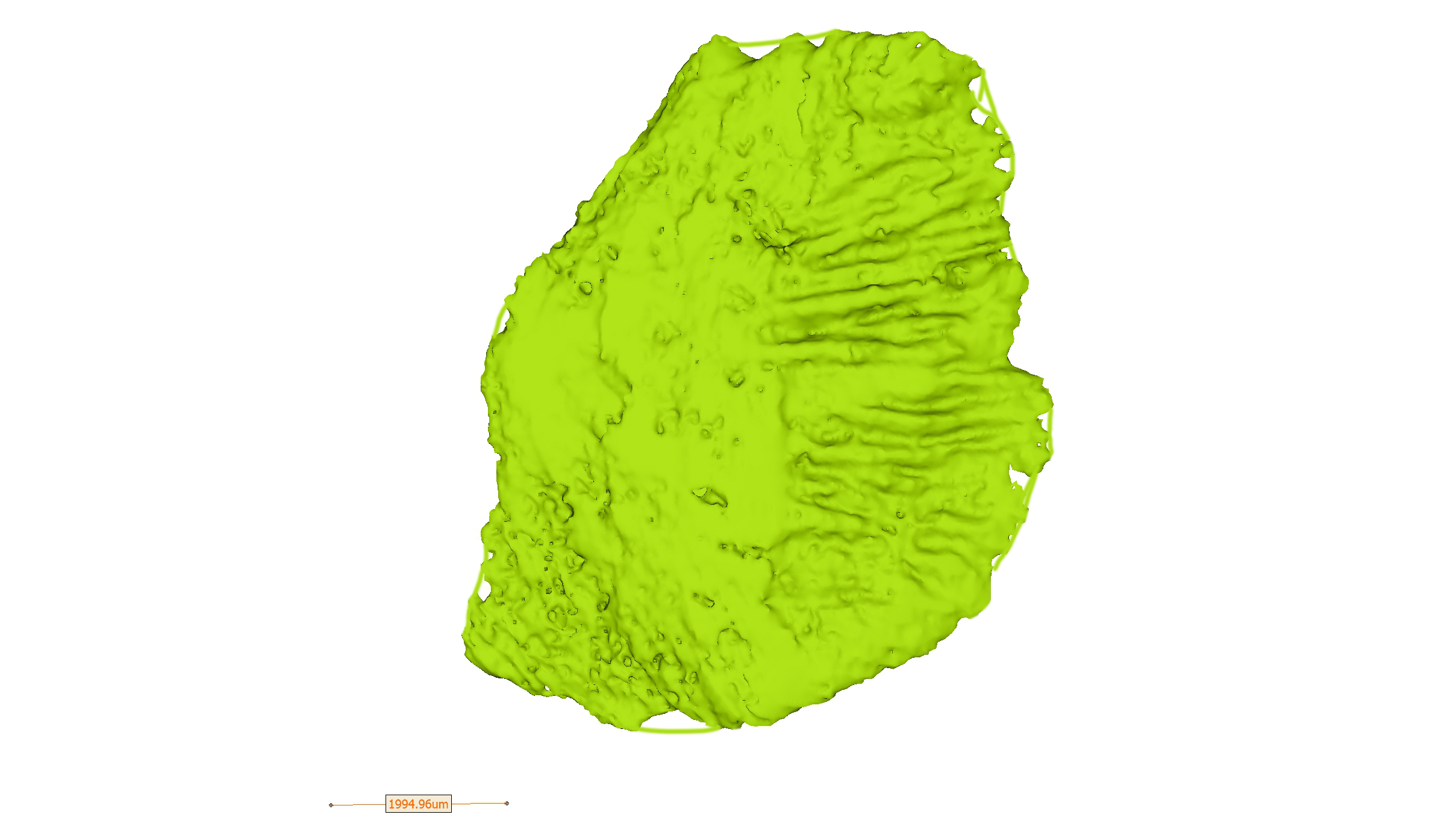

Supplement: Supplementary file 5 — Supplementary Data 2 [file 41467_2023_43557_MOESM5_ESM.zip › Supplementary Data 2/Supplementary Data 2 Raw data of Geometric Morphometric Analyses/12 Morphotypes/Morphotype 3/l2d09.jpg]

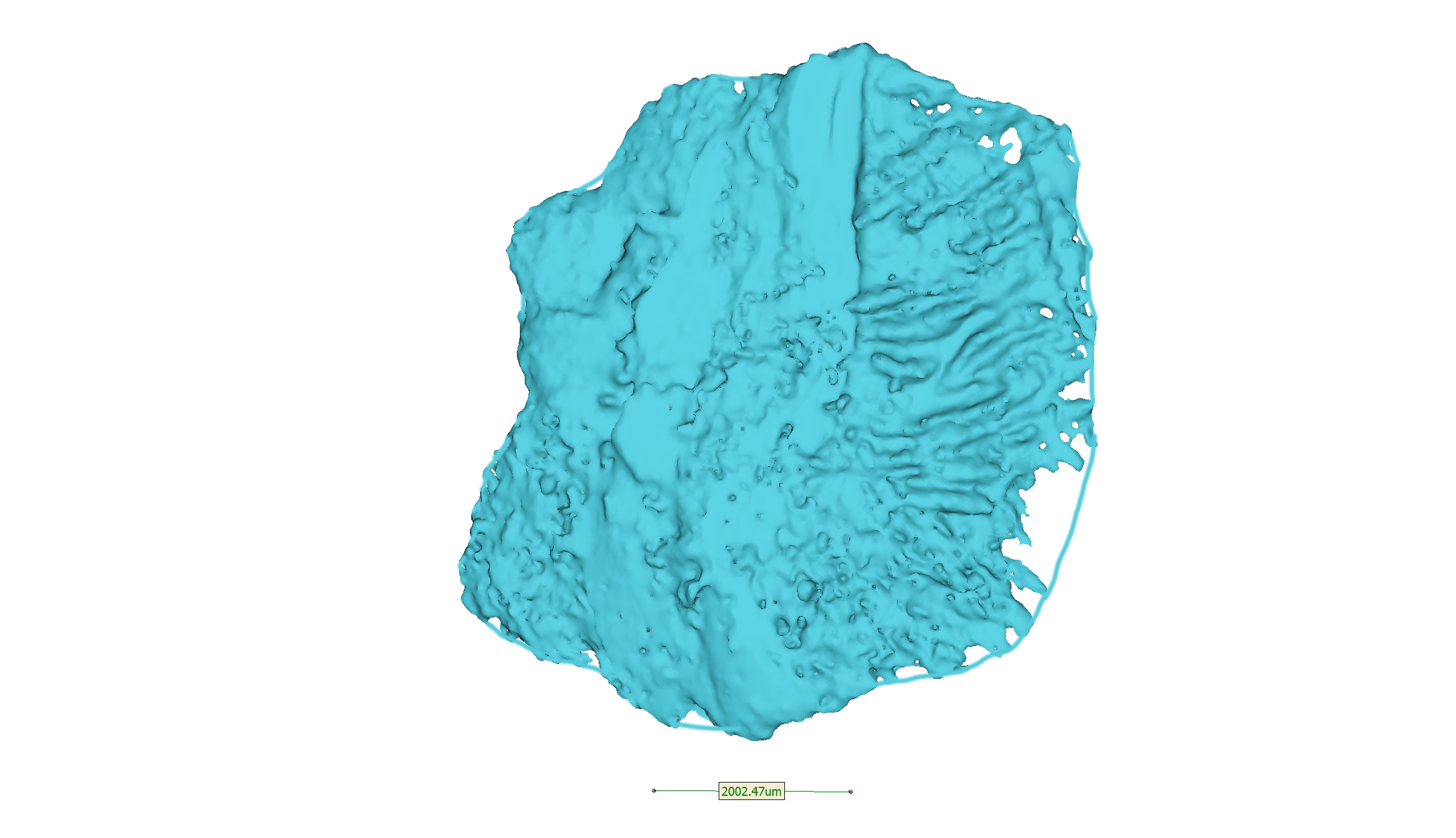

Supplement: Supplementary file 5 — Supplementary Data 2 [file 41467_2023_43557_MOESM5_ESM.zip › Supplementary Data 2/Supplementary Data 2 Raw data of Geometric Morphometric Analyses/12 Morphotypes/Morphotype 3/l2d10.jpg]

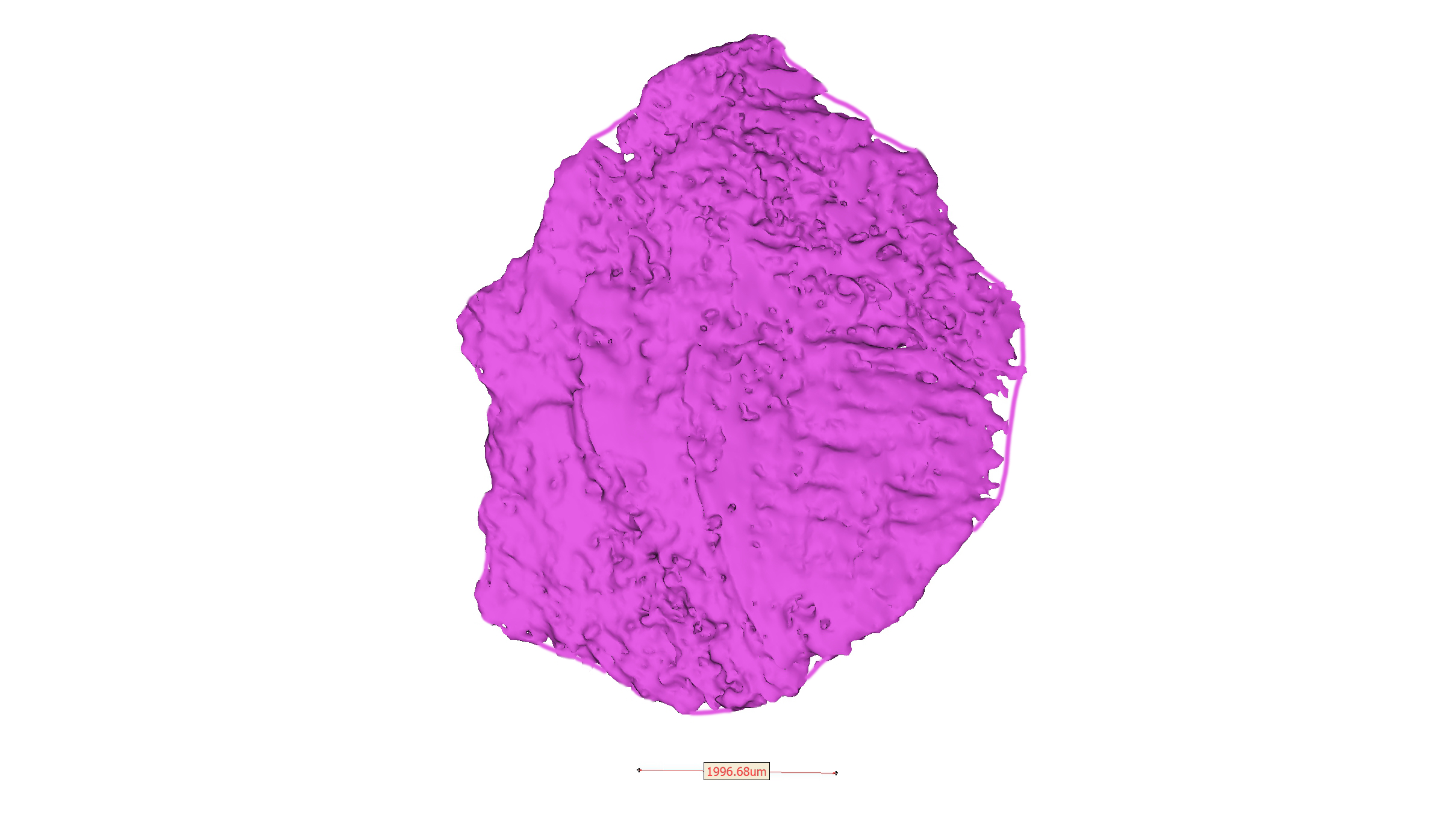

Supplement: Supplementary file 5 — Supplementary Data 2 [file 41467_2023_43557_MOESM5_ESM.zip › Supplementary Data 2/Supplementary Data 2 Raw data of Geometric Morphometric Analyses/12 Morphotypes/Morphotype 3/l2d11.jpg]

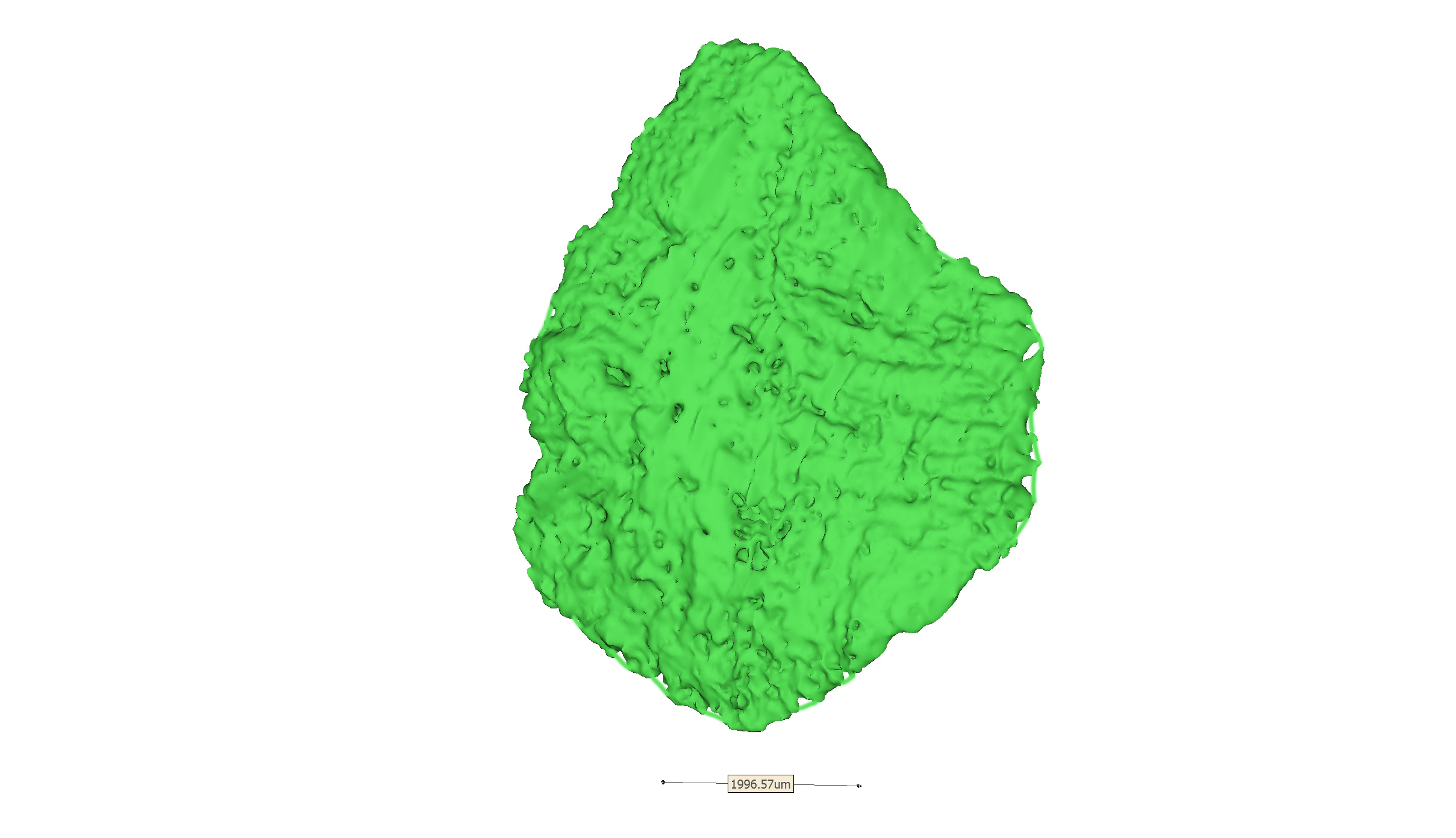

Supplement: Supplementary file 5 — Supplementary Data 2 [file 41467_2023_43557_MOESM5_ESM.zip › Supplementary Data 2/Supplementary Data 2 Raw data of Geometric Morphometric Analyses/12 Morphotypes/Morphotype 3/l2d12.jpg]

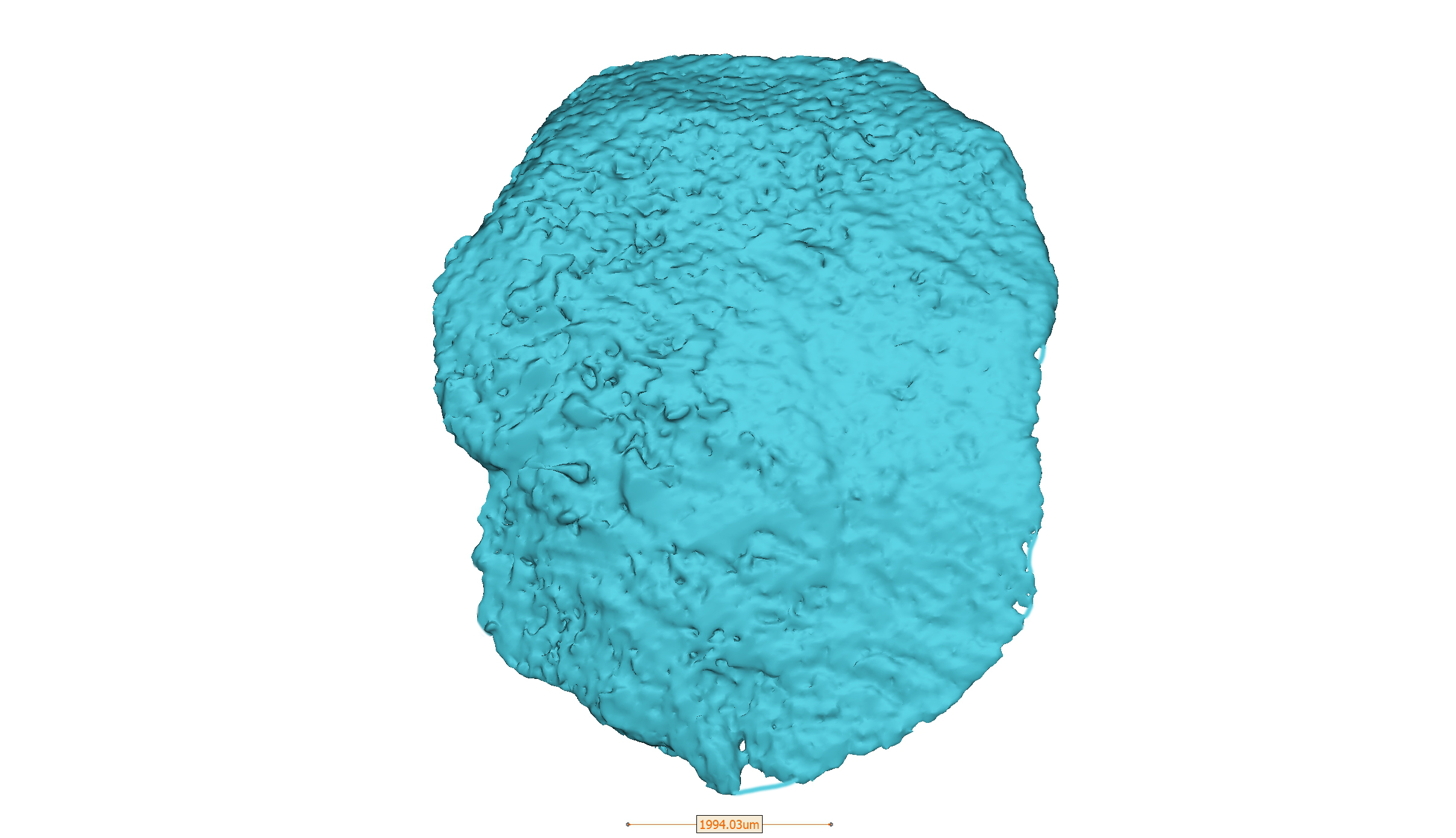

Supplement: Supplementary file 5 — Supplementary Data 2 [file 41467_2023_43557_MOESM5_ESM.zip › Supplementary Data 2/Supplementary Data 2 Raw data of Geometric Morphometric Analyses/12 Morphotypes/Morphotype 3/l2d14.jpg]

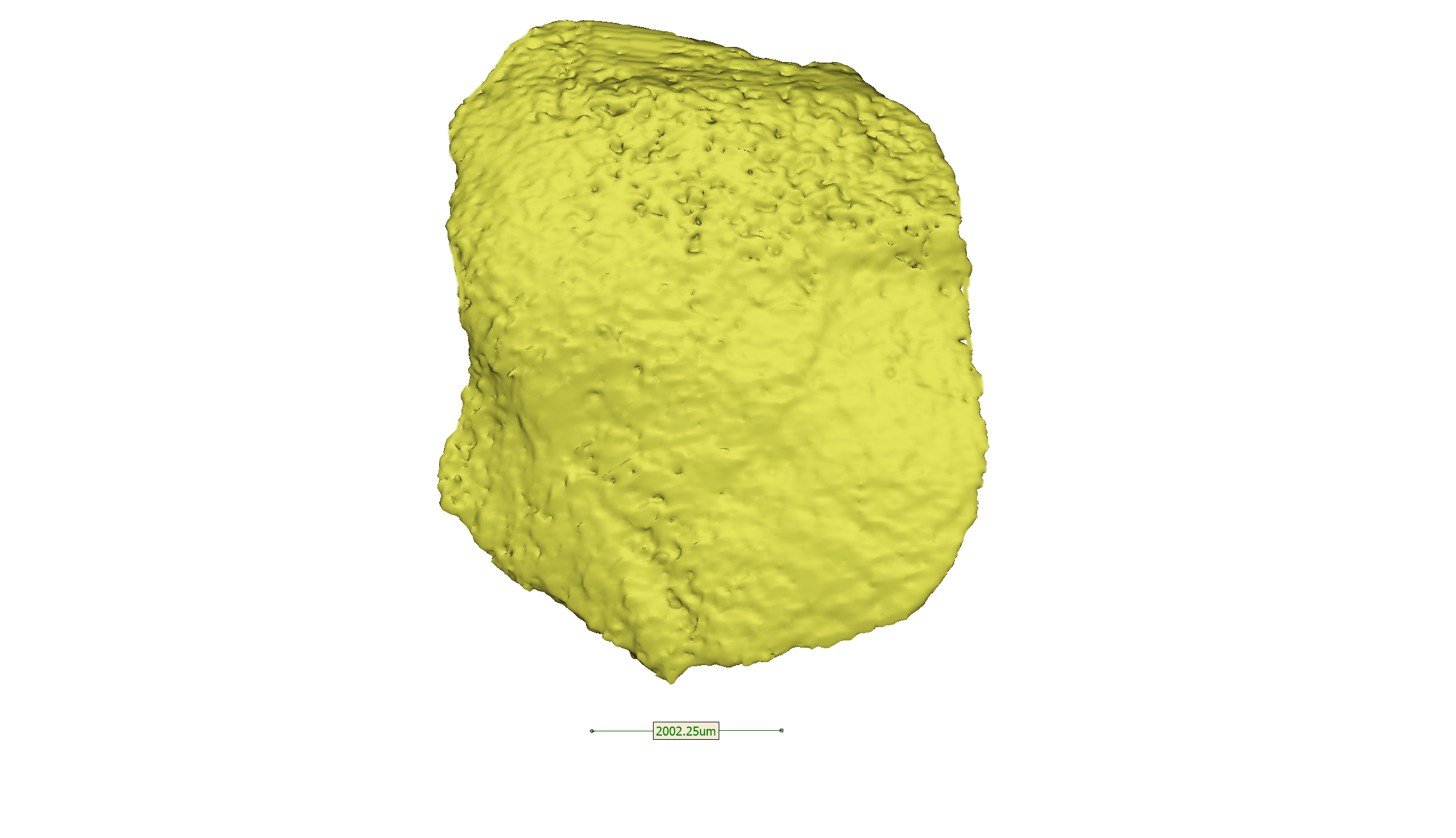

Supplement: Supplementary file 5 — Supplementary Data 2 [file 41467_2023_43557_MOESM5_ESM.zip › Supplementary Data 2/Supplementary Data 2 Raw data of Geometric Morphometric Analyses/12 Morphotypes/Morphotype 3/l2d15.jpg]

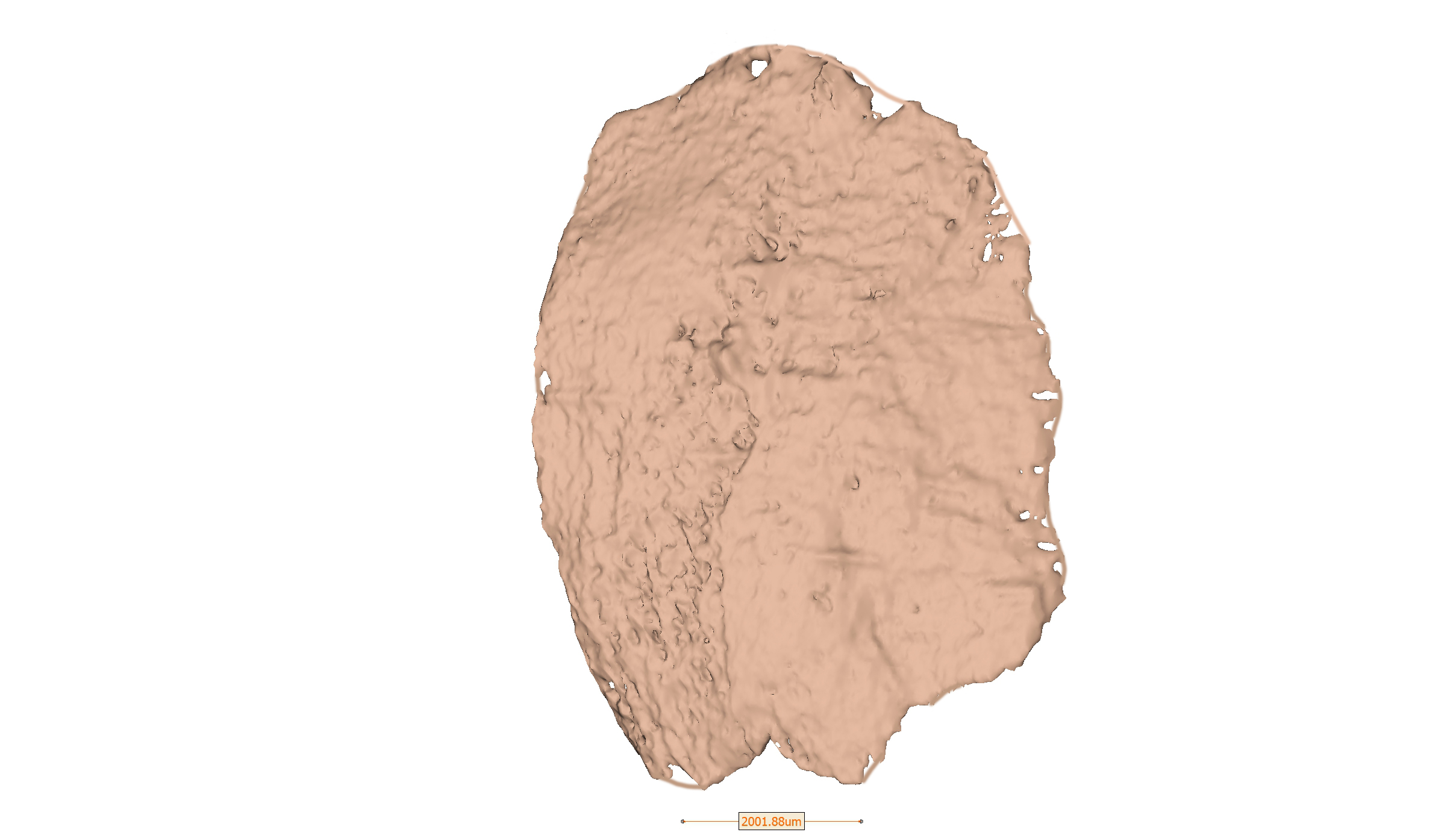

Supplement: Supplementary file 5 — Supplementary Data 2 [file 41467_2023_43557_MOESM5_ESM.zip › Supplementary Data 2/Supplementary Data 2 Raw data of Geometric Morphometric Analyses/12 Morphotypes/Morphotype 3/l2v02 ú¿╨í╨▐ú⌐.jpg]

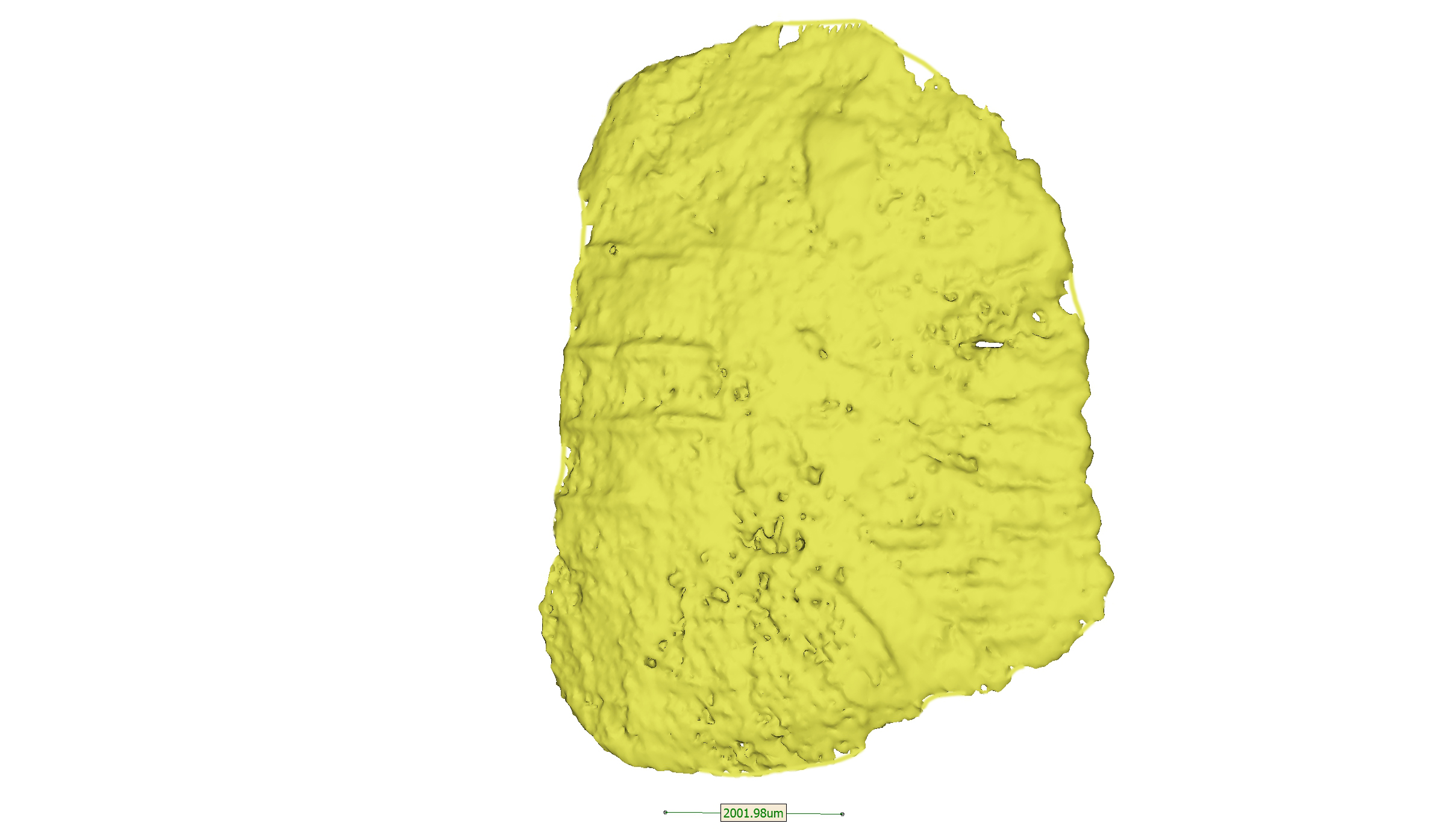

Supplement: Supplementary file 5 — Supplementary Data 2 [file 41467_2023_43557_MOESM5_ESM.zip › Supplementary Data 2/Supplementary Data 2 Raw data of Geometric Morphometric Analyses/12 Morphotypes/Morphotype 3/l2v03.jpg]

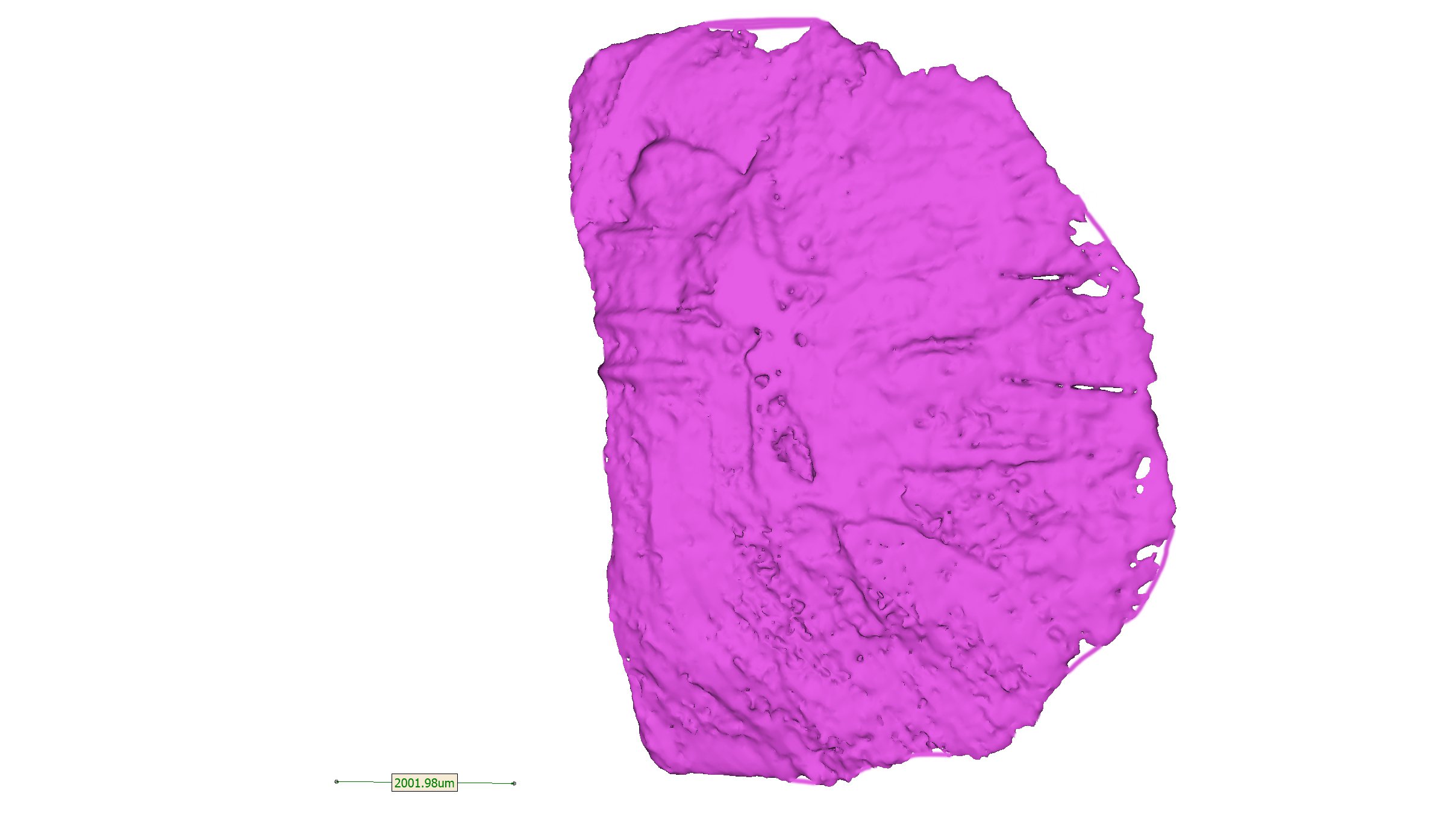

Supplement: Supplementary file 5 — Supplementary Data 2 [file 41467_2023_43557_MOESM5_ESM.zip › Supplementary Data 2/Supplementary Data 2 Raw data of Geometric Morphometric Analyses/12 Morphotypes/Morphotype 3/l2v04.jpg]

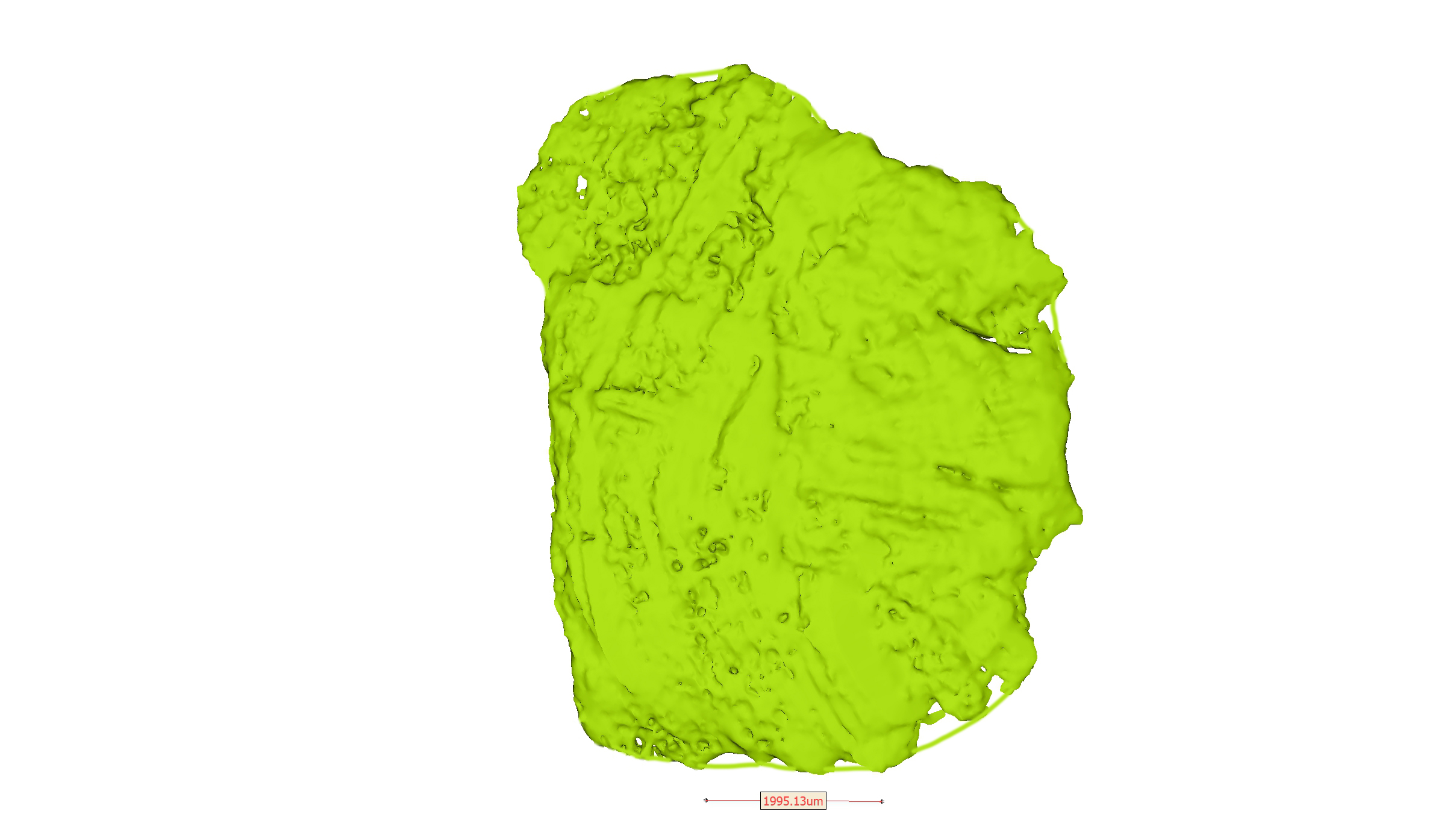

Supplement: Supplementary file 5 — Supplementary Data 2 [file 41467_2023_43557_MOESM5_ESM.zip › Supplementary Data 2/Supplementary Data 2 Raw data of Geometric Morphometric Analyses/12 Morphotypes/Morphotype 3/l2v05.jpg]

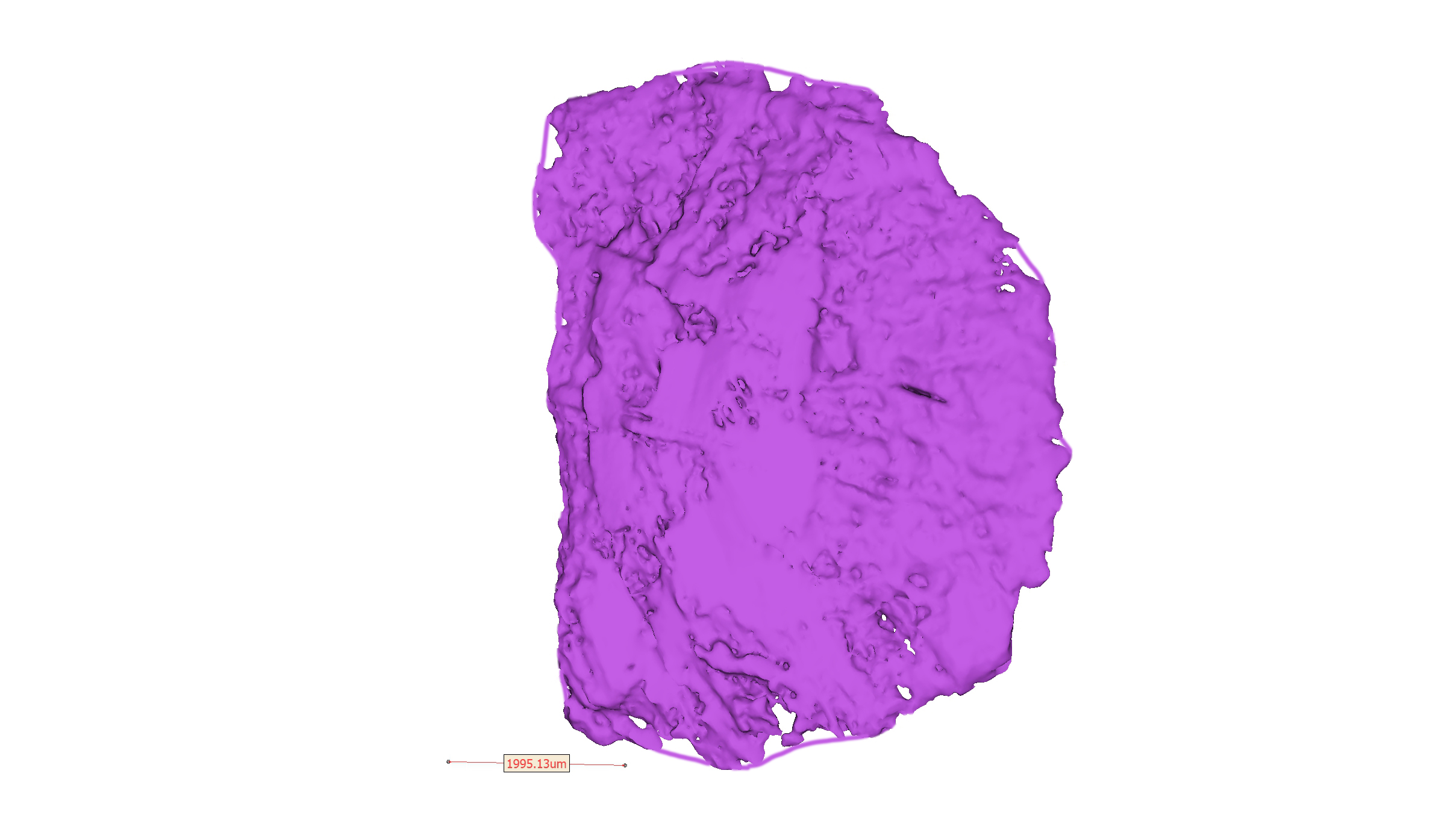

Supplement: Supplementary file 5 — Supplementary Data 2 [file 41467_2023_43557_MOESM5_ESM.zip › Supplementary Data 2/Supplementary Data 2 Raw data of Geometric Morphometric Analyses/12 Morphotypes/Morphotype 3/l2v06.jpg]

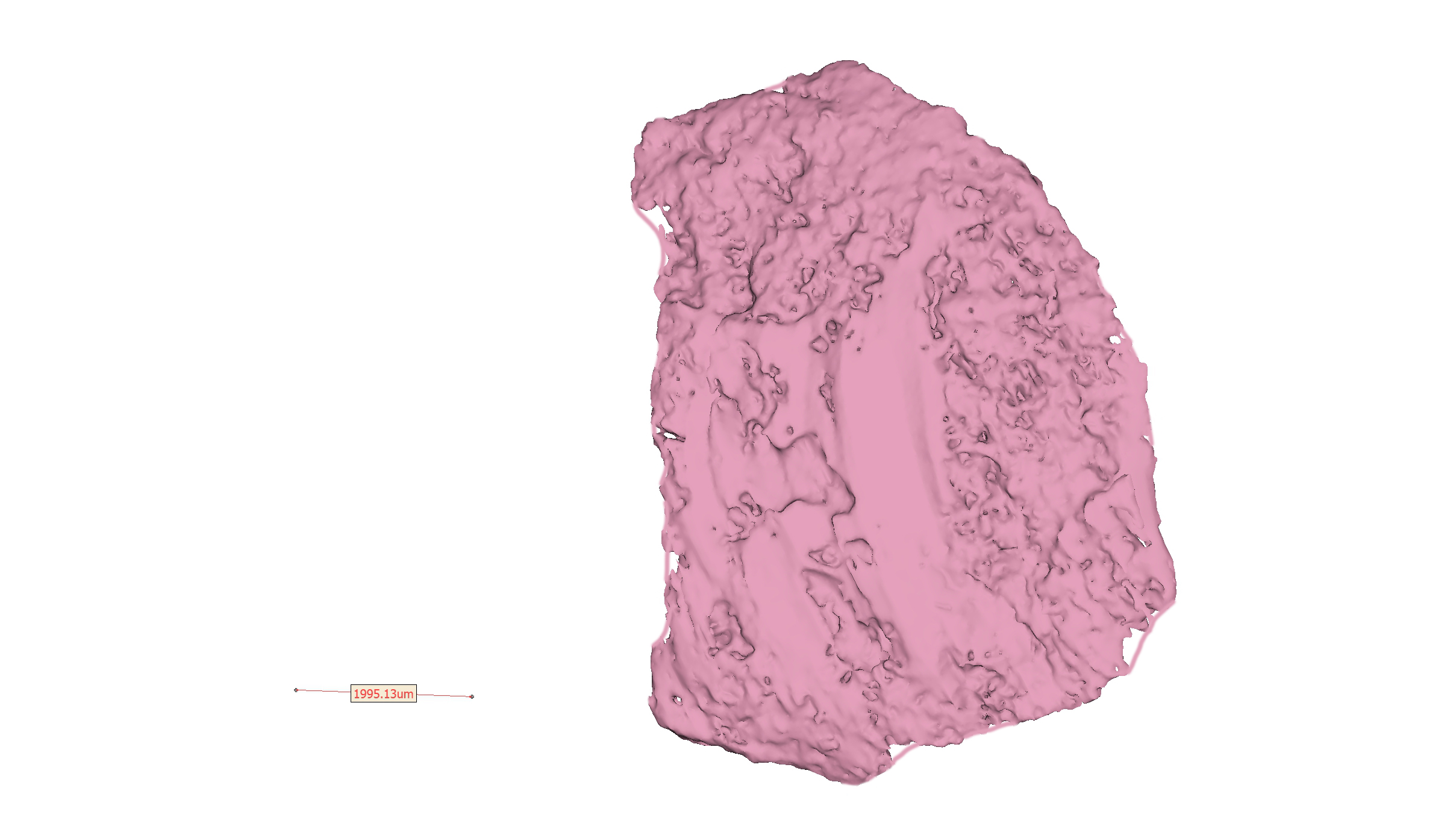

Supplement: Supplementary file 5 — Supplementary Data 2 [file 41467_2023_43557_MOESM5_ESM.zip › Supplementary Data 2/Supplementary Data 2 Raw data of Geometric Morphometric Analyses/12 Morphotypes/Morphotype 3/l2v07.jpg]

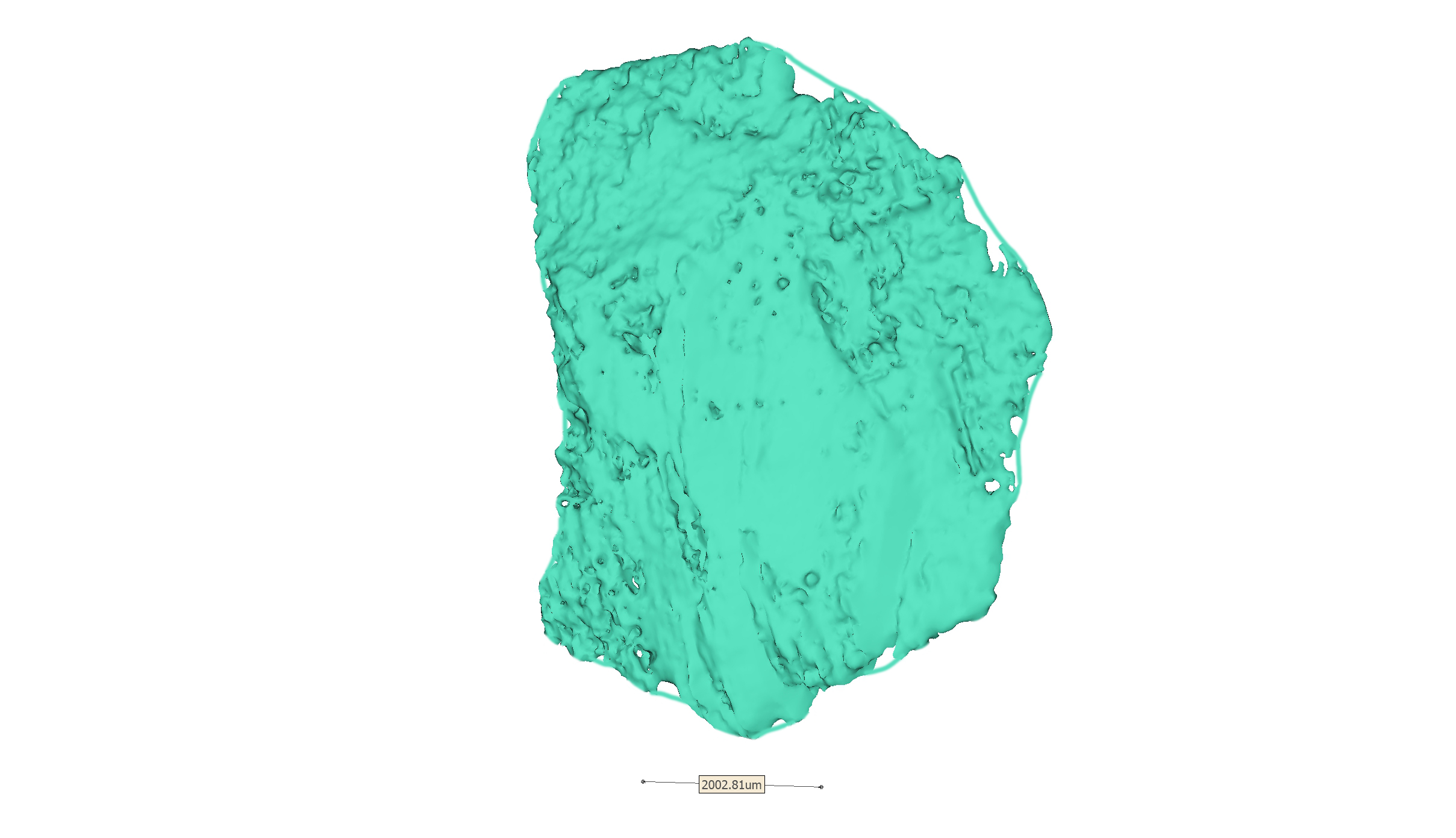

Supplement: Supplementary file 5 — Supplementary Data 2 [file 41467_2023_43557_MOESM5_ESM.zip › Supplementary Data 2/Supplementary Data 2 Raw data of Geometric Morphometric Analyses/12 Morphotypes/Morphotype 3/l2v08.jpg]

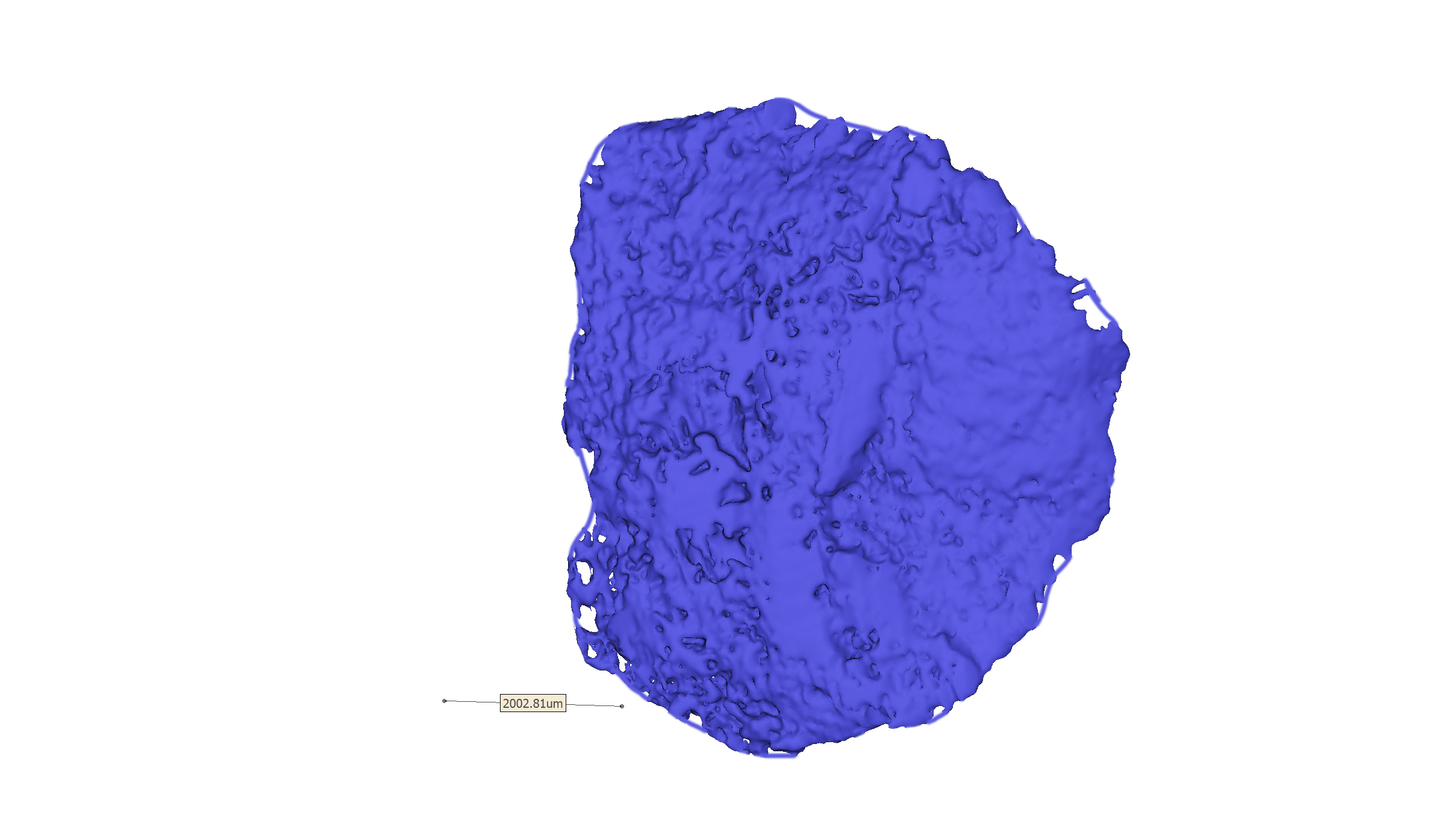

Supplement: Supplementary file 5 — Supplementary Data 2 [file 41467_2023_43557_MOESM5_ESM.zip › Supplementary Data 2/Supplementary Data 2 Raw data of Geometric Morphometric Analyses/12 Morphotypes/Morphotype 3/l2v09.jpg]

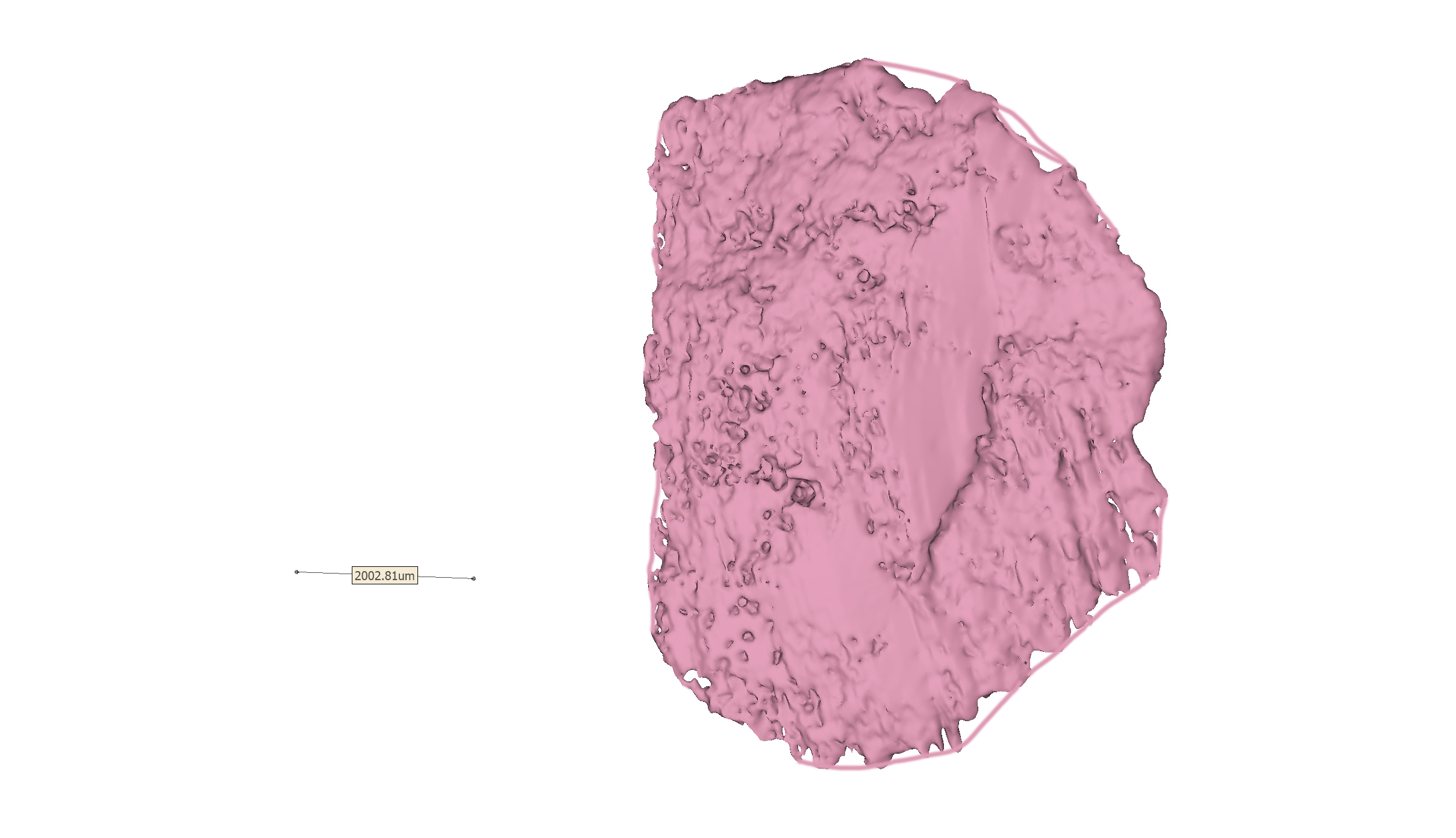

Supplement: Supplementary file 5 — Supplementary Data 2 [file 41467_2023_43557_MOESM5_ESM.zip › Supplementary Data 2/Supplementary Data 2 Raw data of Geometric Morphometric Analyses/12 Morphotypes/Morphotype 3/l2v10.jpg]

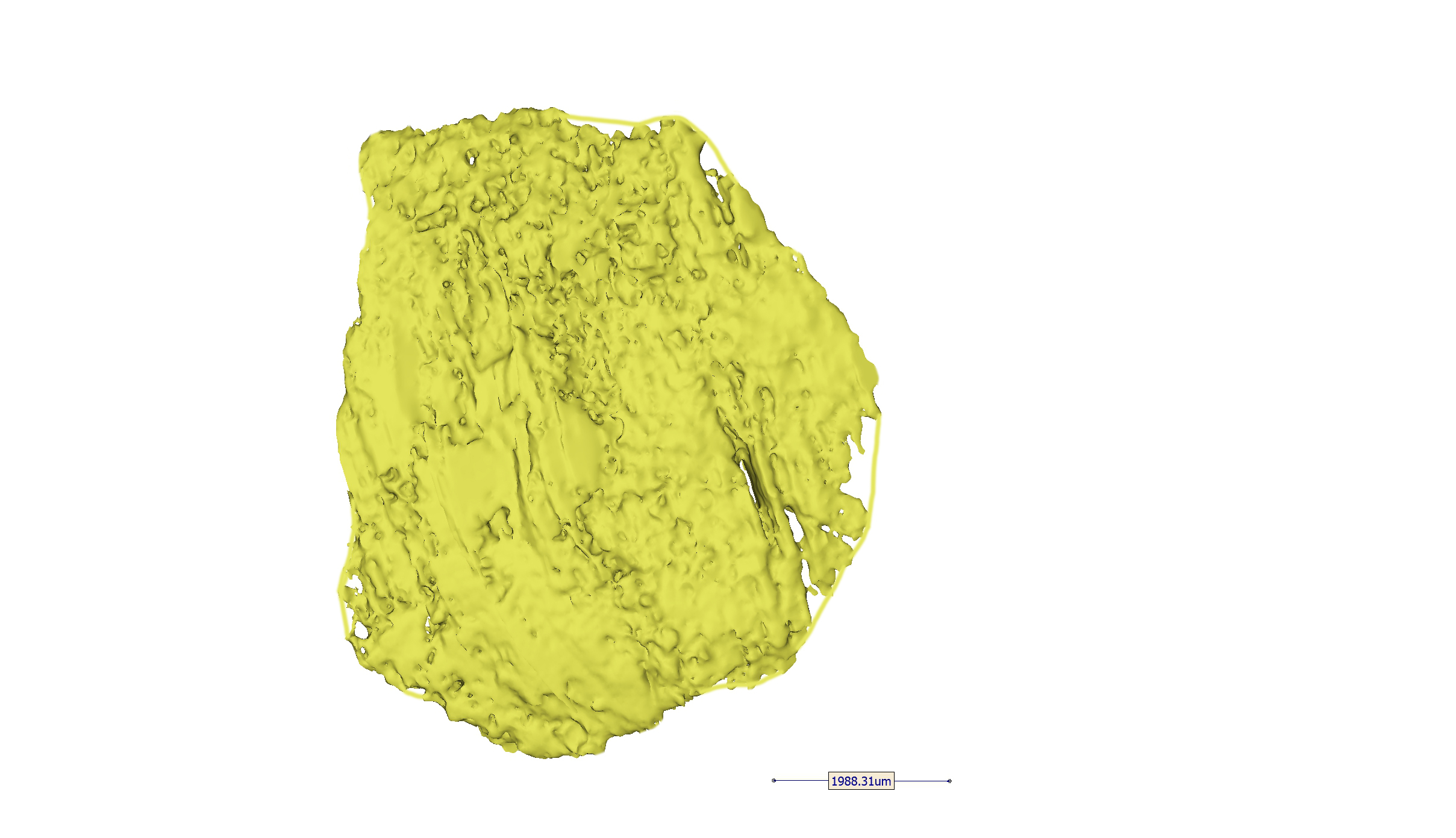

Supplement: Supplementary file 5 — Supplementary Data 2 [file 41467_2023_43557_MOESM5_ESM.zip › Supplementary Data 2/Supplementary Data 2 Raw data of Geometric Morphometric Analyses/12 Morphotypes/Morphotype 3/l2v11.jpg]

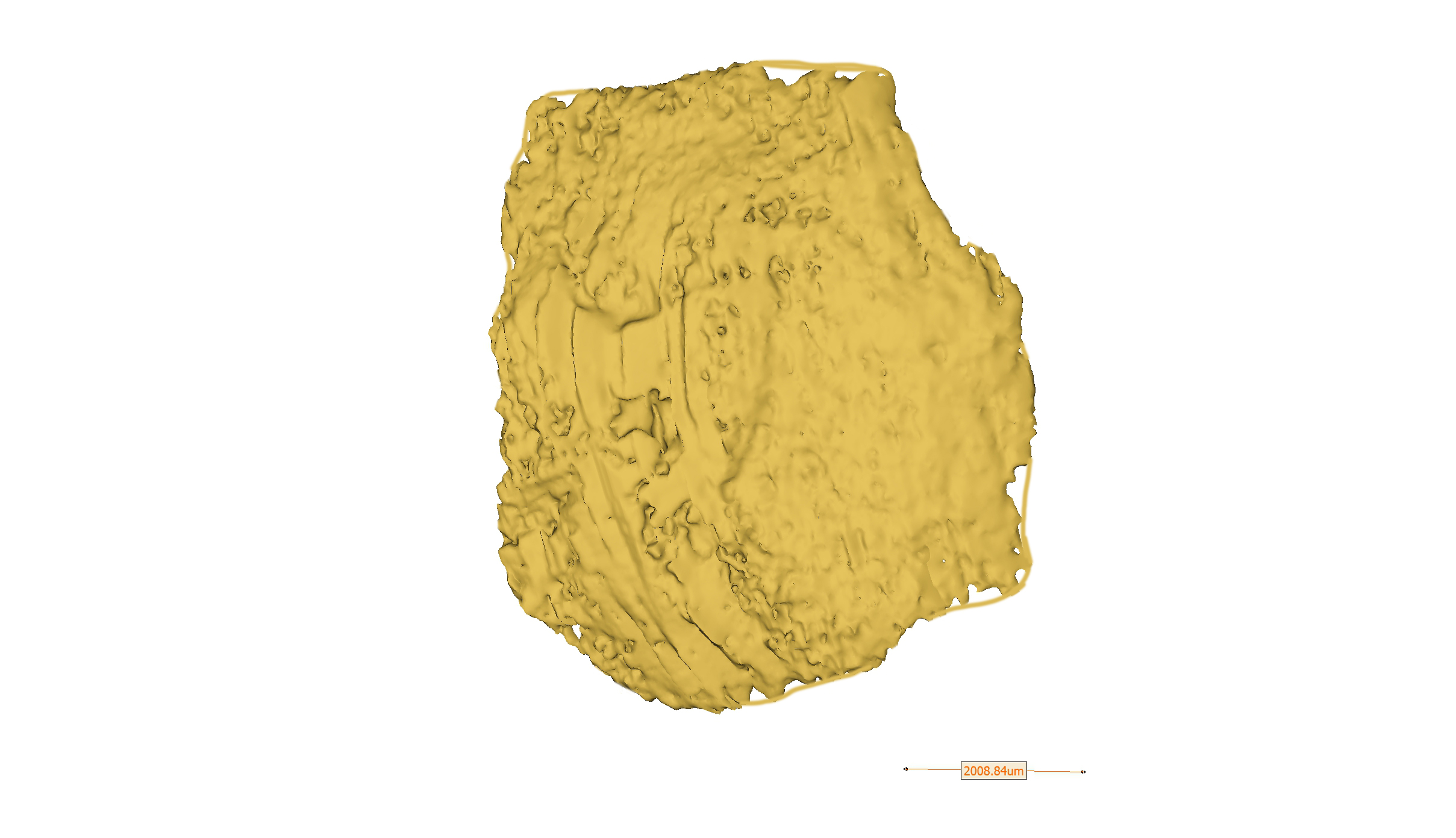

Supplement: Supplementary file 5 — Supplementary Data 2 [file 41467_2023_43557_MOESM5_ESM.zip › Supplementary Data 2/Supplementary Data 2 Raw data of Geometric Morphometric Analyses/12 Morphotypes/Morphotype 3/l2v12.jpg]

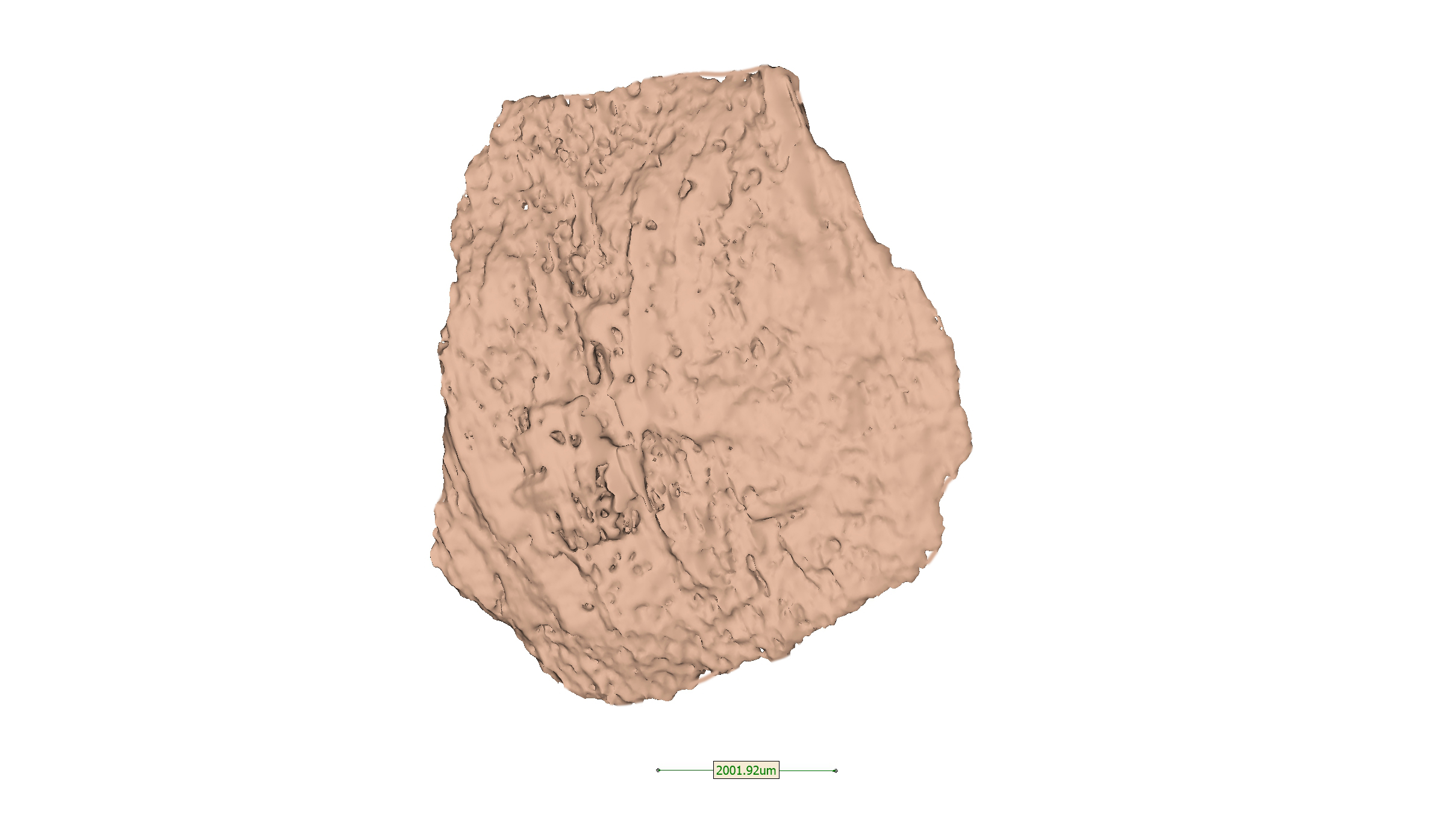

Supplement: Supplementary file 5 — Supplementary Data 2 [file 41467_2023_43557_MOESM5_ESM.zip › Supplementary Data 2/Supplementary Data 2 Raw data of Geometric Morphometric Analyses/12 Morphotypes/Morphotype 3/l2v13.jpg]

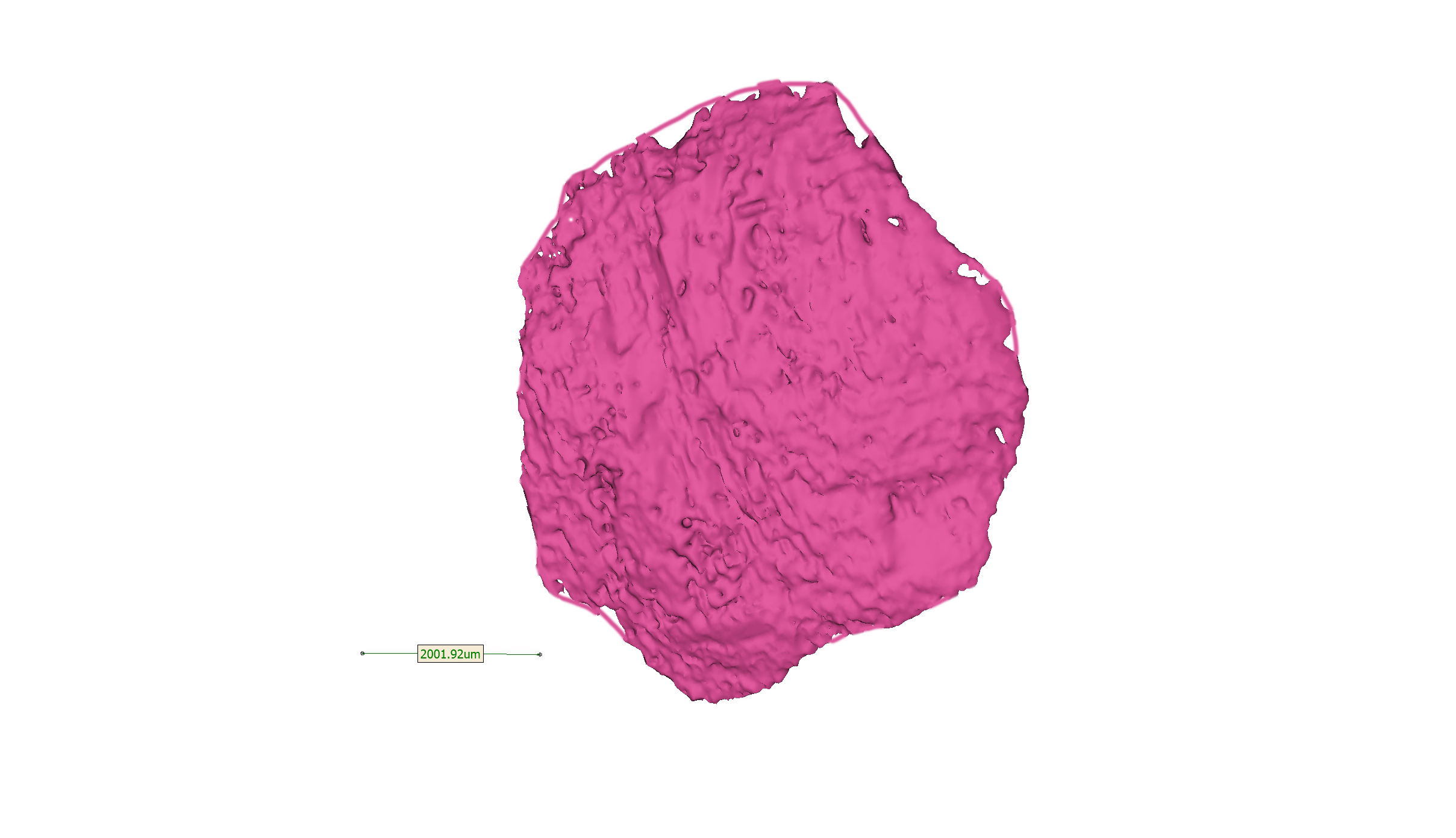

Supplement: Supplementary file 5 — Supplementary Data 2 [file 41467_2023_43557_MOESM5_ESM.zip › Supplementary Data 2/Supplementary Data 2 Raw data of Geometric Morphometric Analyses/12 Morphotypes/Morphotype 3/l2v14.jpg]

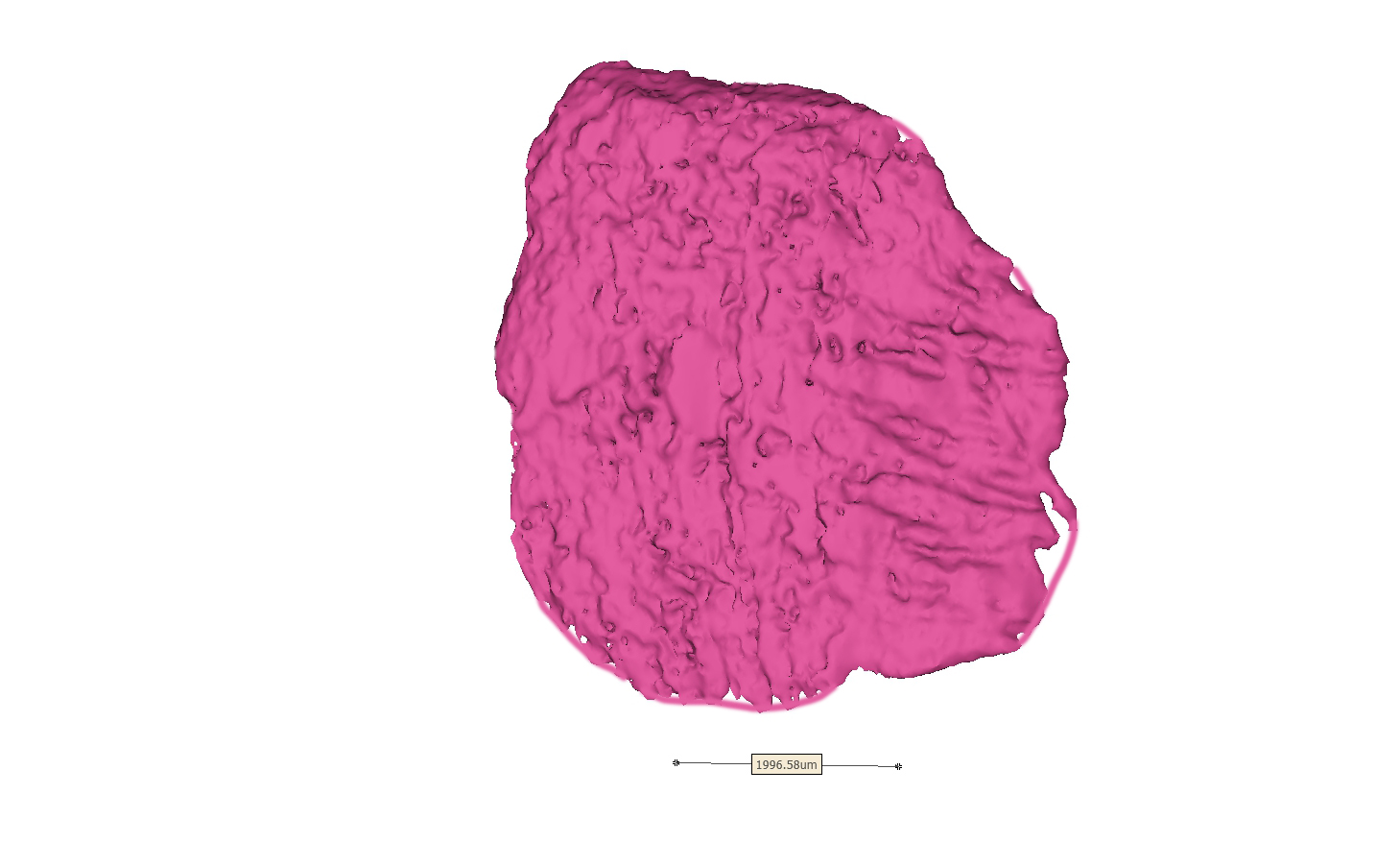

Supplement: Supplementary file 5 — Supplementary Data 2 [file 41467_2023_43557_MOESM5_ESM.zip › Supplementary Data 2/Supplementary Data 2 Raw data of Geometric Morphometric Analyses/12 Morphotypes/Morphotype 3/l3d01.jpg]

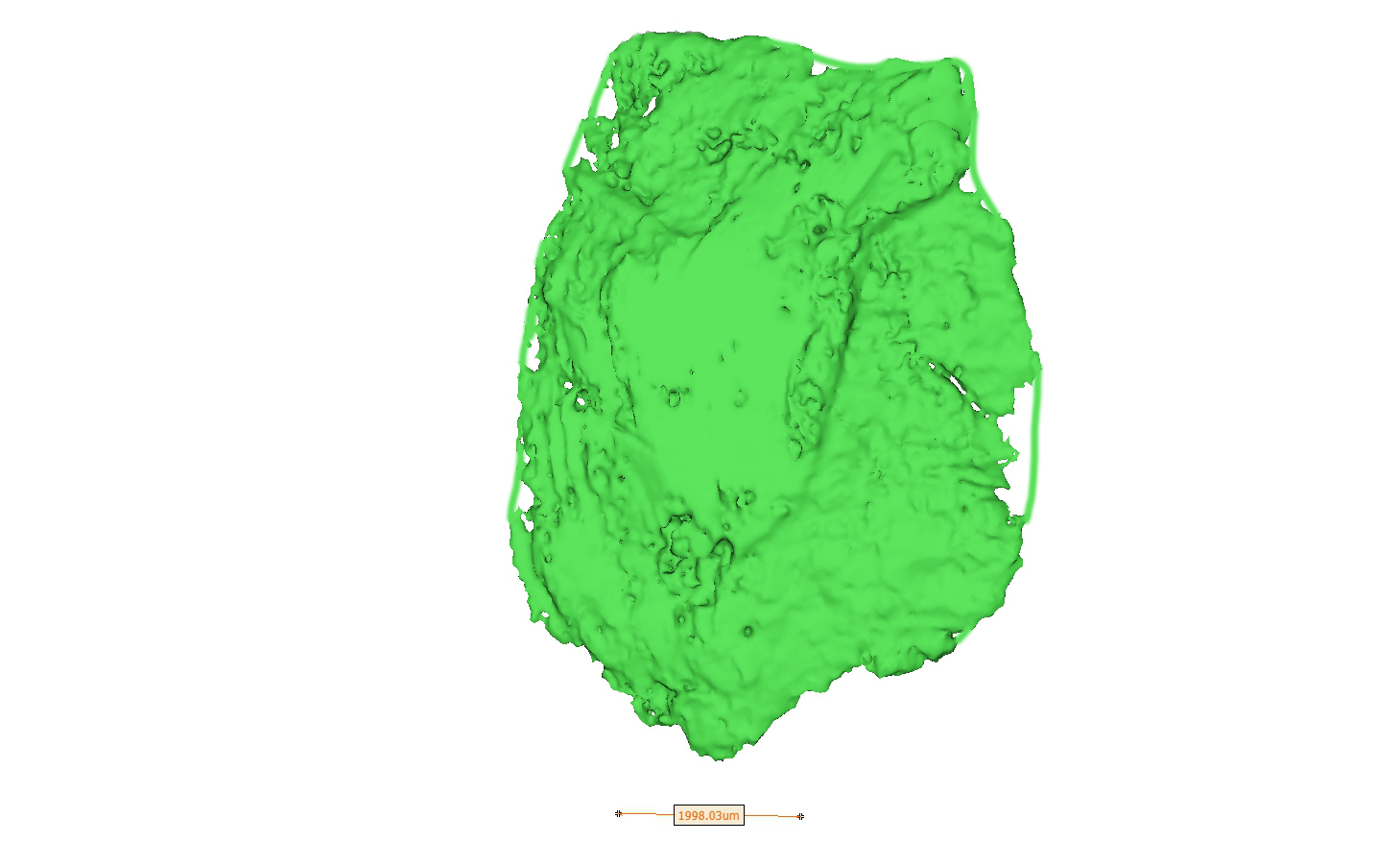

Supplement: Supplementary file 5 — Supplementary Data 2 [file 41467_2023_43557_MOESM5_ESM.zip › Supplementary Data 2/Supplementary Data 2 Raw data of Geometric Morphometric Analyses/12 Morphotypes/Morphotype 3/l3v06.jpg]

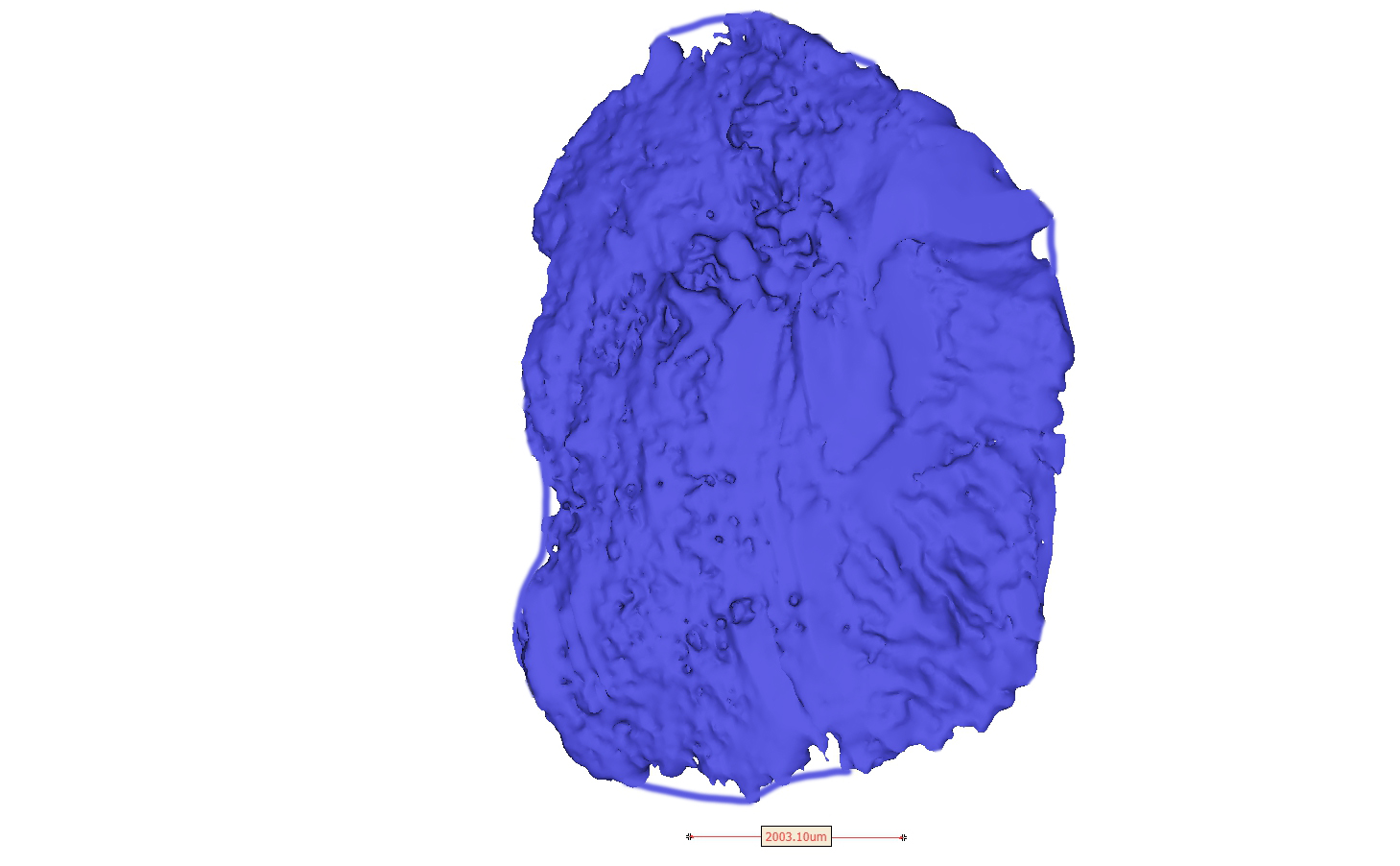

Supplement: Supplementary file 5 — Supplementary Data 2 [file 41467_2023_43557_MOESM5_ESM.zip › Supplementary Data 2/Supplementary Data 2 Raw data of Geometric Morphometric Analyses/12 Morphotypes/Morphotype 3/l4v06.jpg]

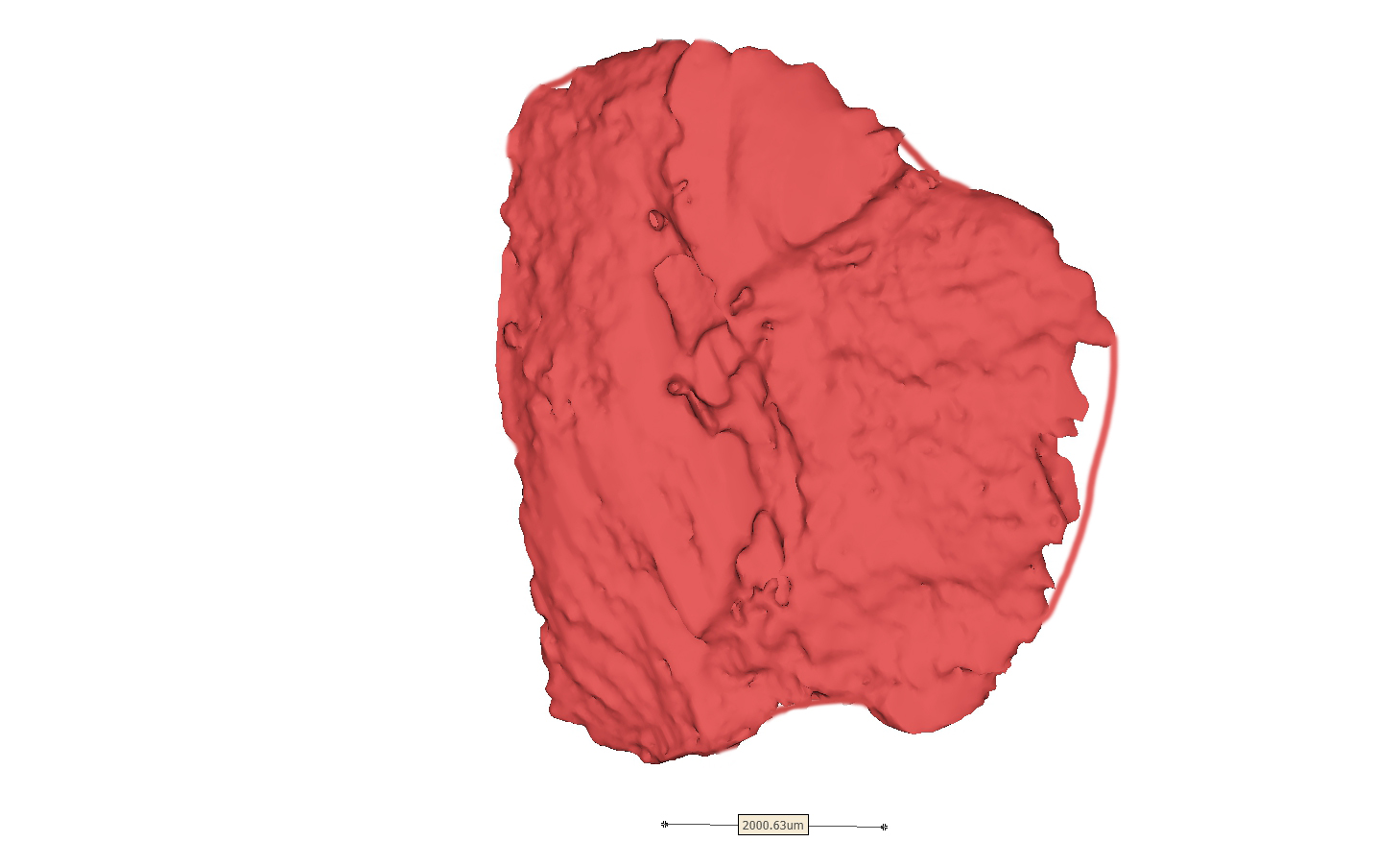

Supplement: Supplementary file 5 — Supplementary Data 2 [file 41467_2023_43557_MOESM5_ESM.zip › Supplementary Data 2/Supplementary Data 2 Raw data of Geometric Morphometric Analyses/12 Morphotypes/Morphotype 3/l4v07.jpg]

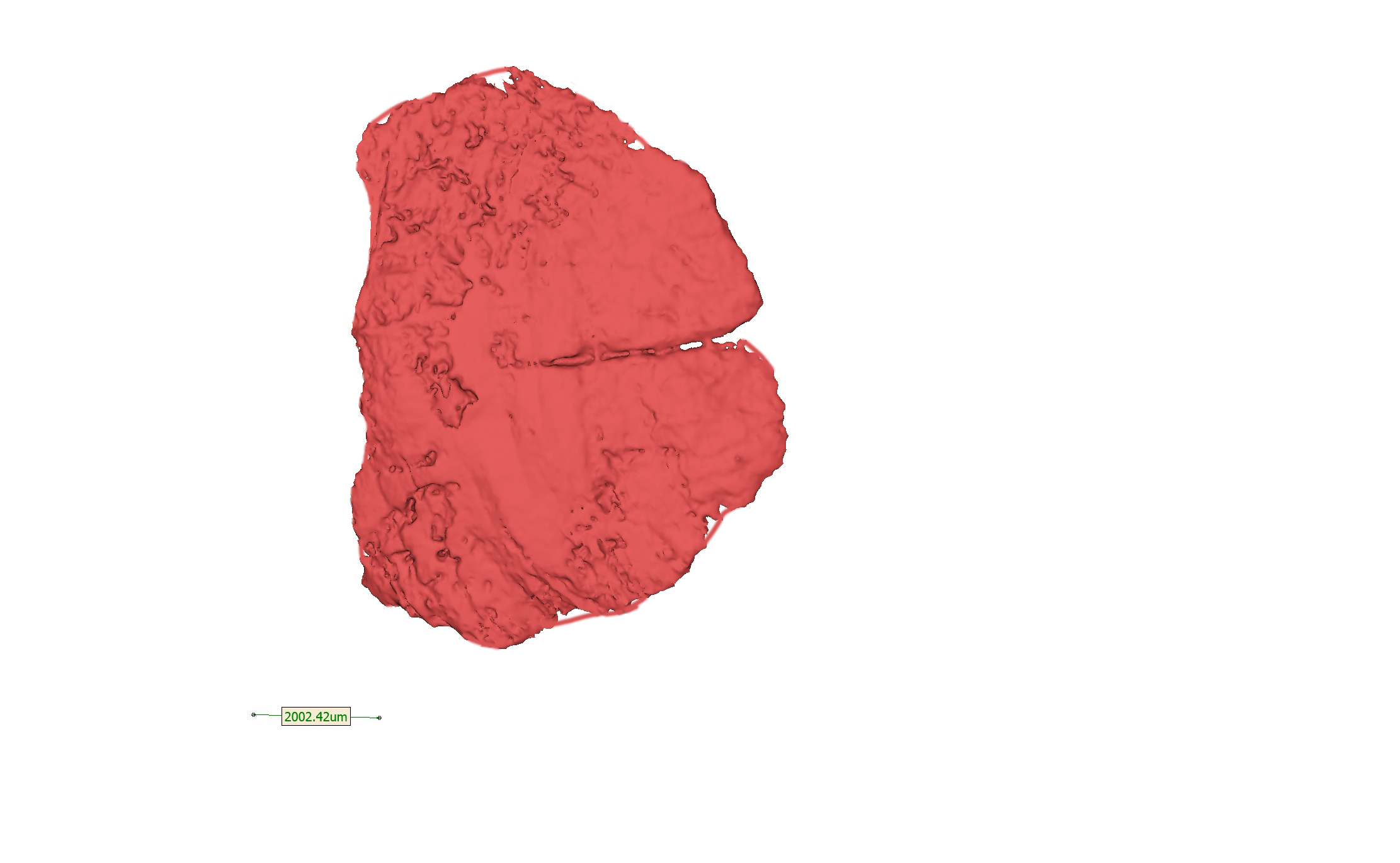

Supplement: Supplementary file 5 — Supplementary Data 2 [file 41467_2023_43557_MOESM5_ESM.zip › Supplementary Data 2/Supplementary Data 2 Raw data of Geometric Morphometric Analyses/12 Morphotypes/Morphotype 3/ll07.jpg]

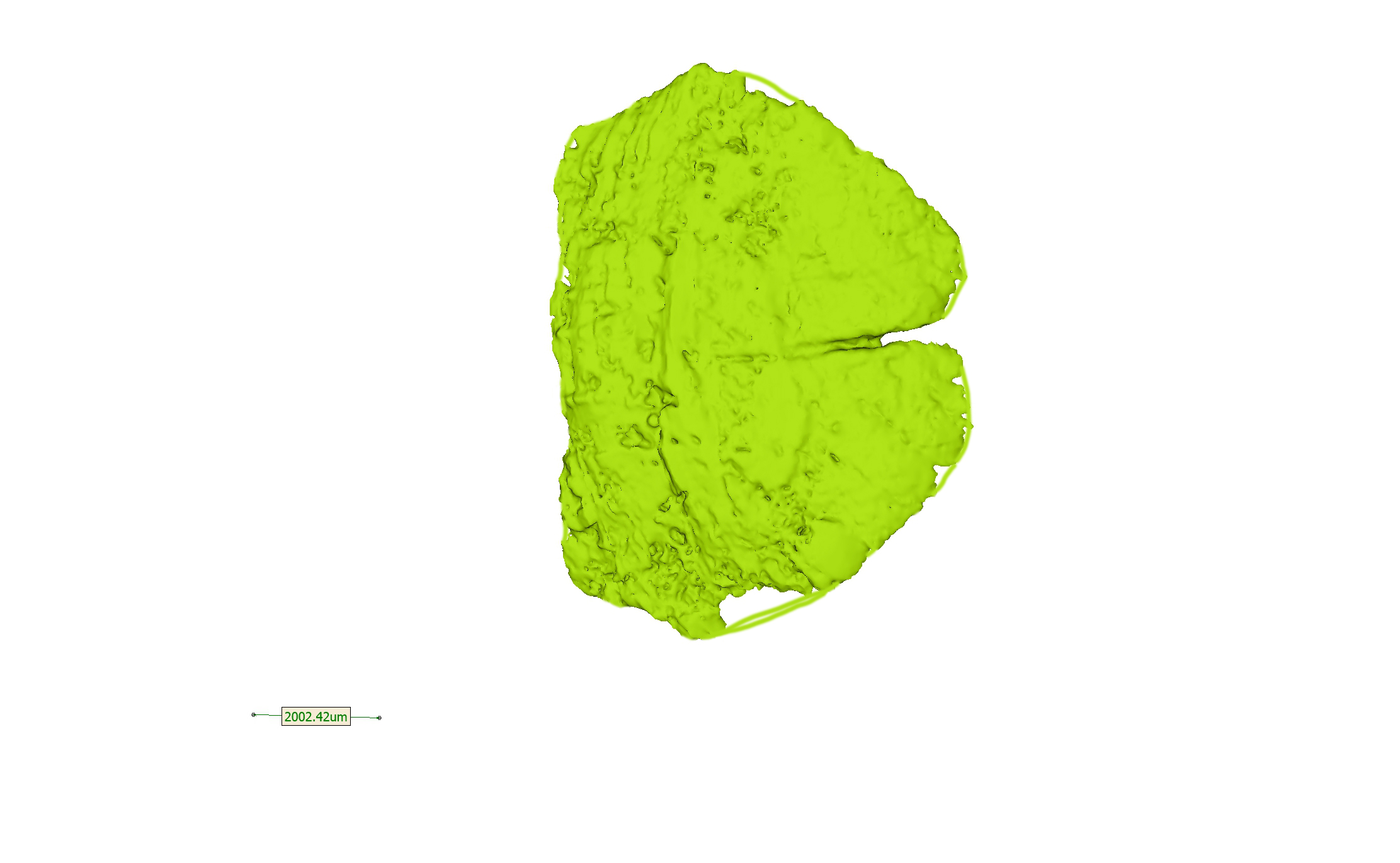

Supplement: Supplementary file 5 — Supplementary Data 2 [file 41467_2023_43557_MOESM5_ESM.zip › Supplementary Data 2/Supplementary Data 2 Raw data of Geometric Morphometric Analyses/12 Morphotypes/Morphotype 3/ll08.jpg]

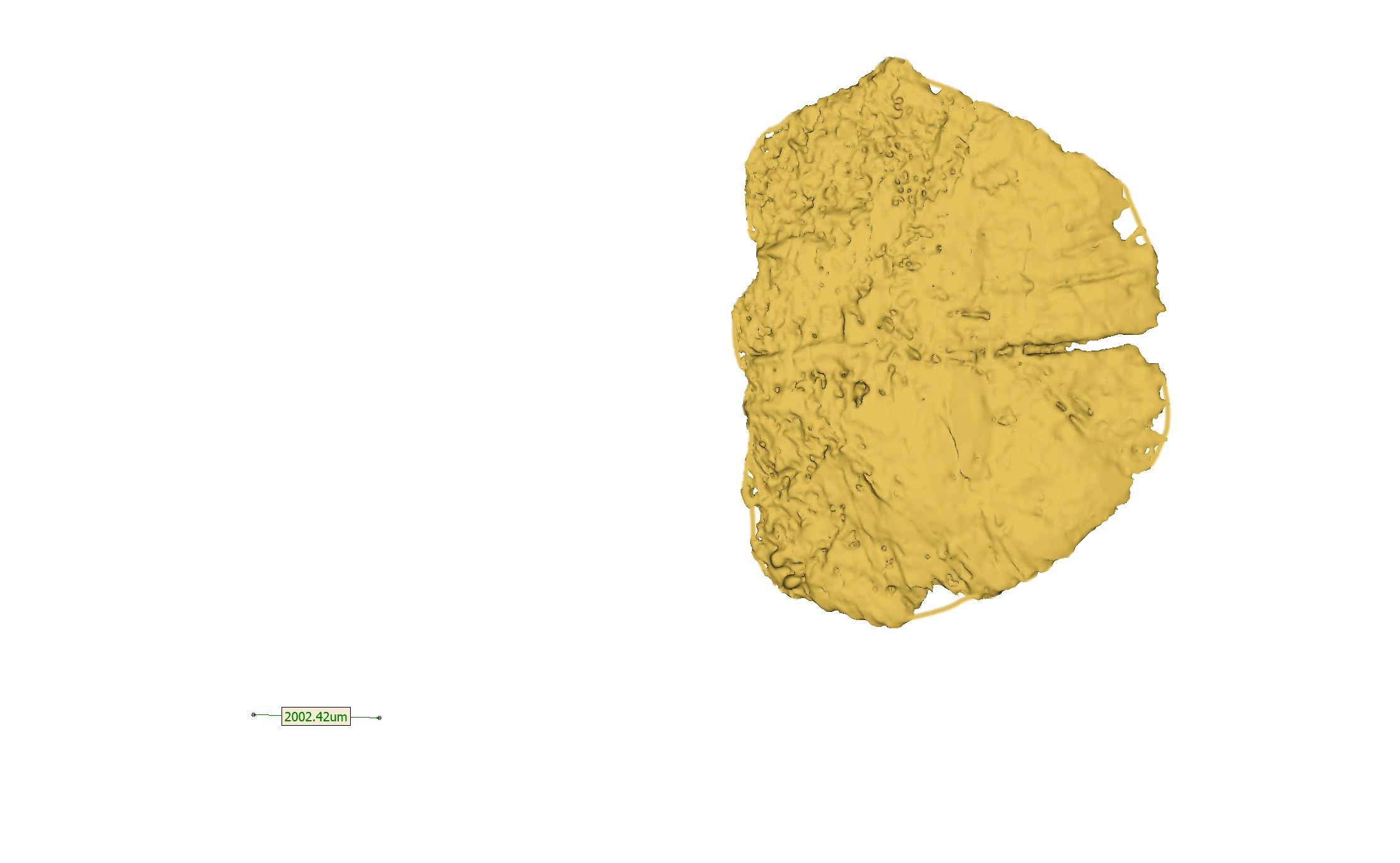

Supplement: Supplementary file 5 — Supplementary Data 2 [file 41467_2023_43557_MOESM5_ESM.zip › Supplementary Data 2/Supplementary Data 2 Raw data of Geometric Morphometric Analyses/12 Morphotypes/Morphotype 3/ll09.jpg]

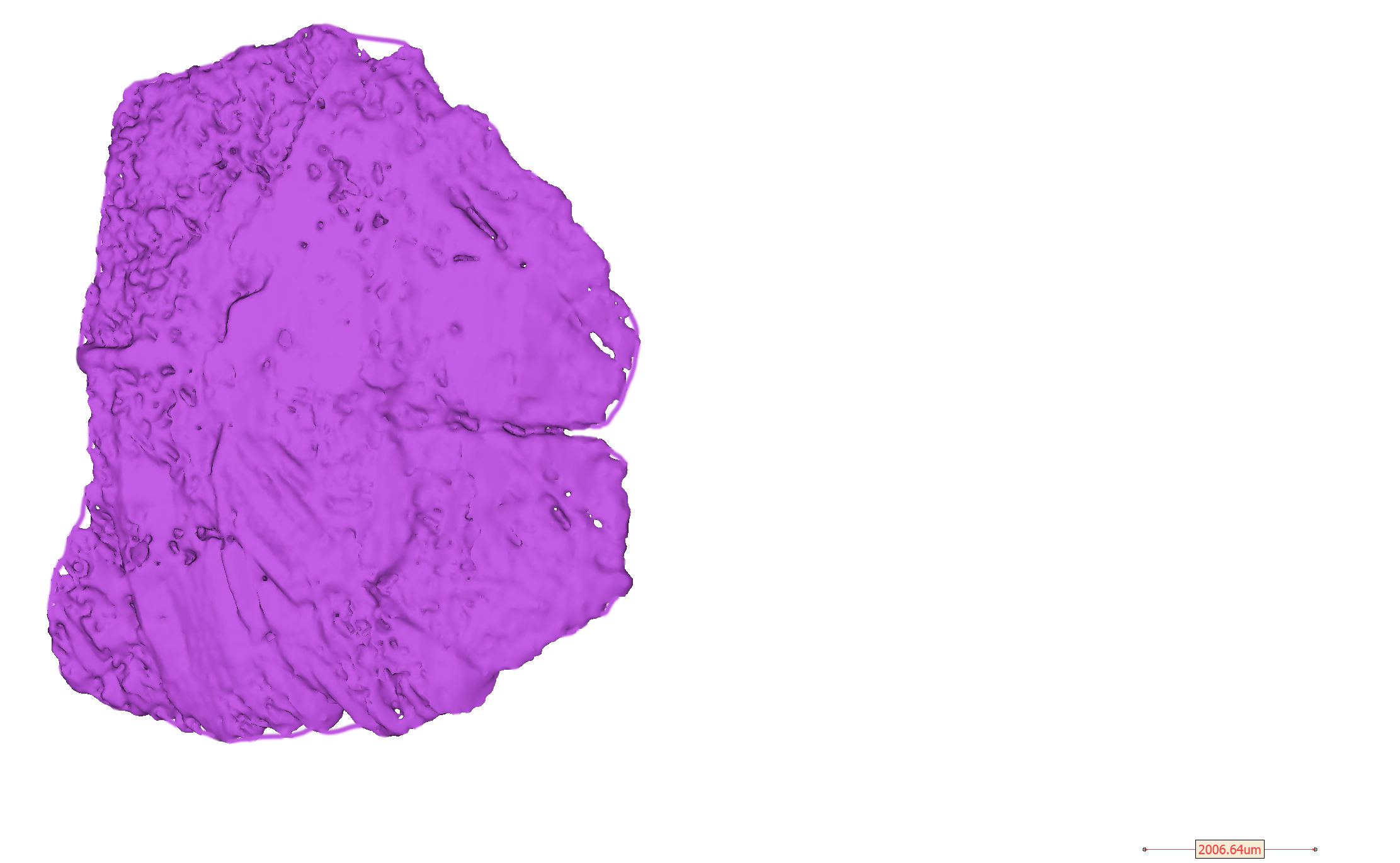

Supplement: Supplementary file 5 — Supplementary Data 2 [file 41467_2023_43557_MOESM5_ESM.zip › Supplementary Data 2/Supplementary Data 2 Raw data of Geometric Morphometric Analyses/12 Morphotypes/Morphotype 3/ll10.jpg]

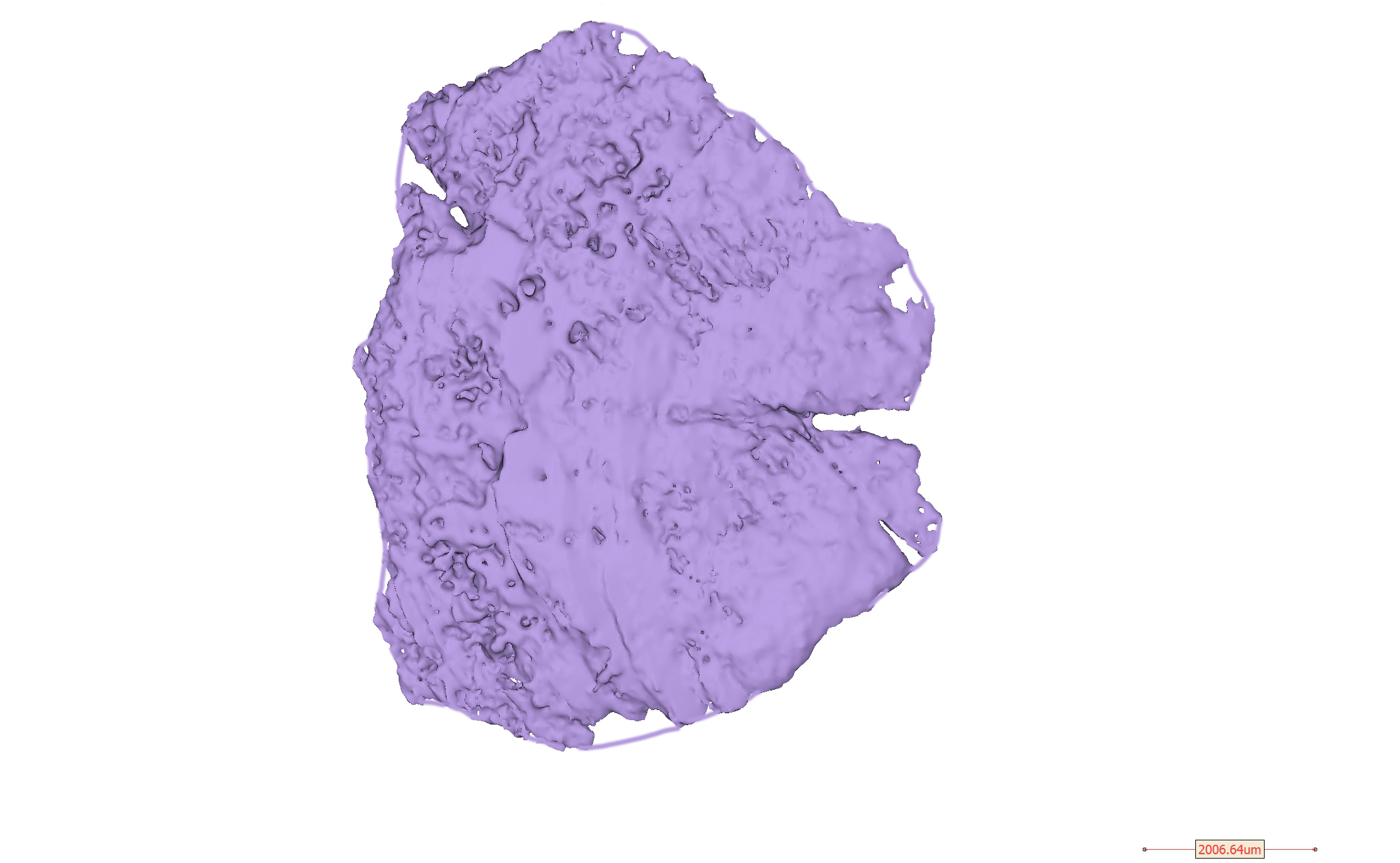

Supplement: Supplementary file 5 — Supplementary Data 2 [file 41467_2023_43557_MOESM5_ESM.zip › Supplementary Data 2/Supplementary Data 2 Raw data of Geometric Morphometric Analyses/12 Morphotypes/Morphotype 3/ll11.jpg]

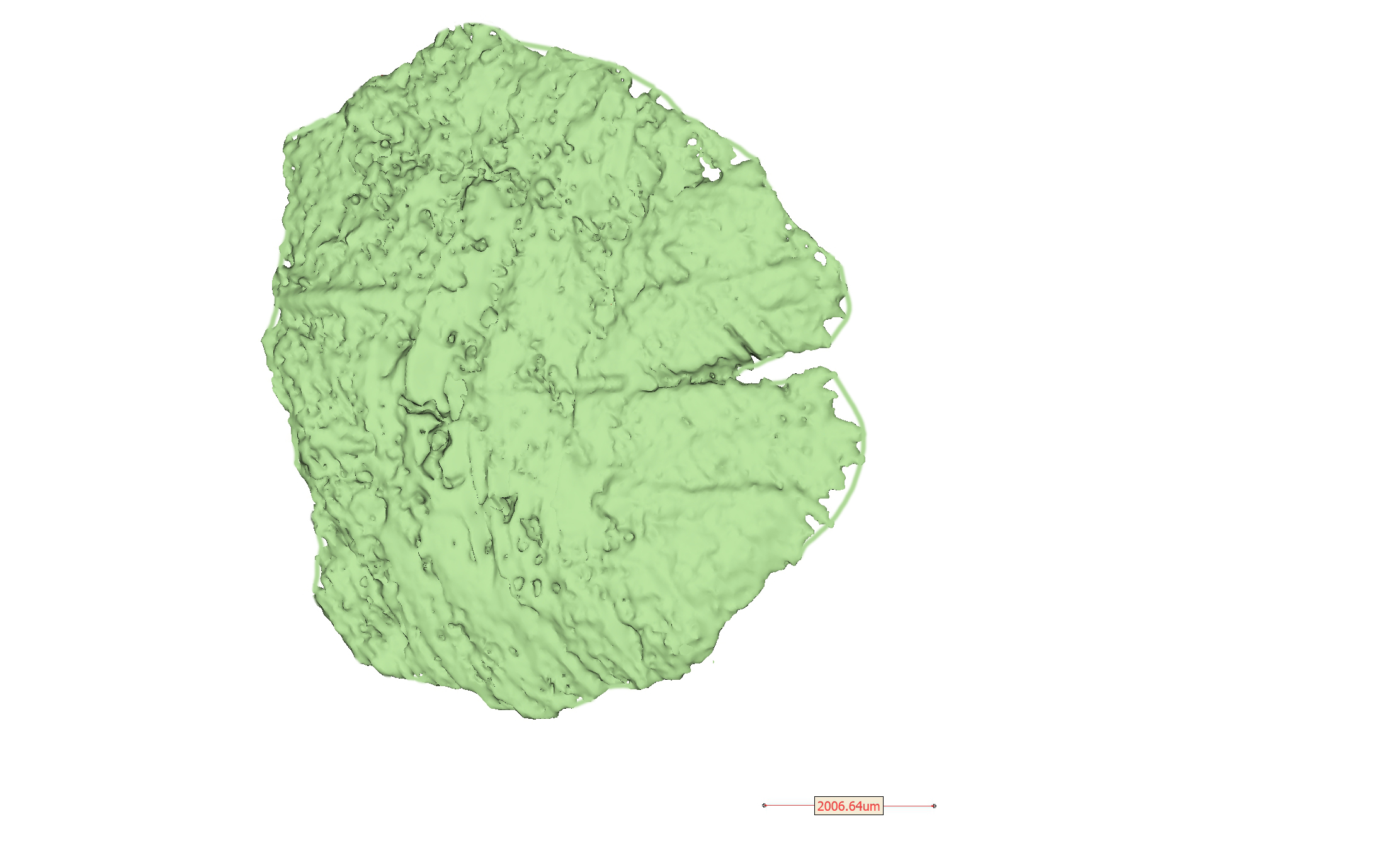

Supplement: Supplementary file 5 — Supplementary Data 2 [file 41467_2023_43557_MOESM5_ESM.zip › Supplementary Data 2/Supplementary Data 2 Raw data of Geometric Morphometric Analyses/12 Morphotypes/Morphotype 3/ll12.jpg]

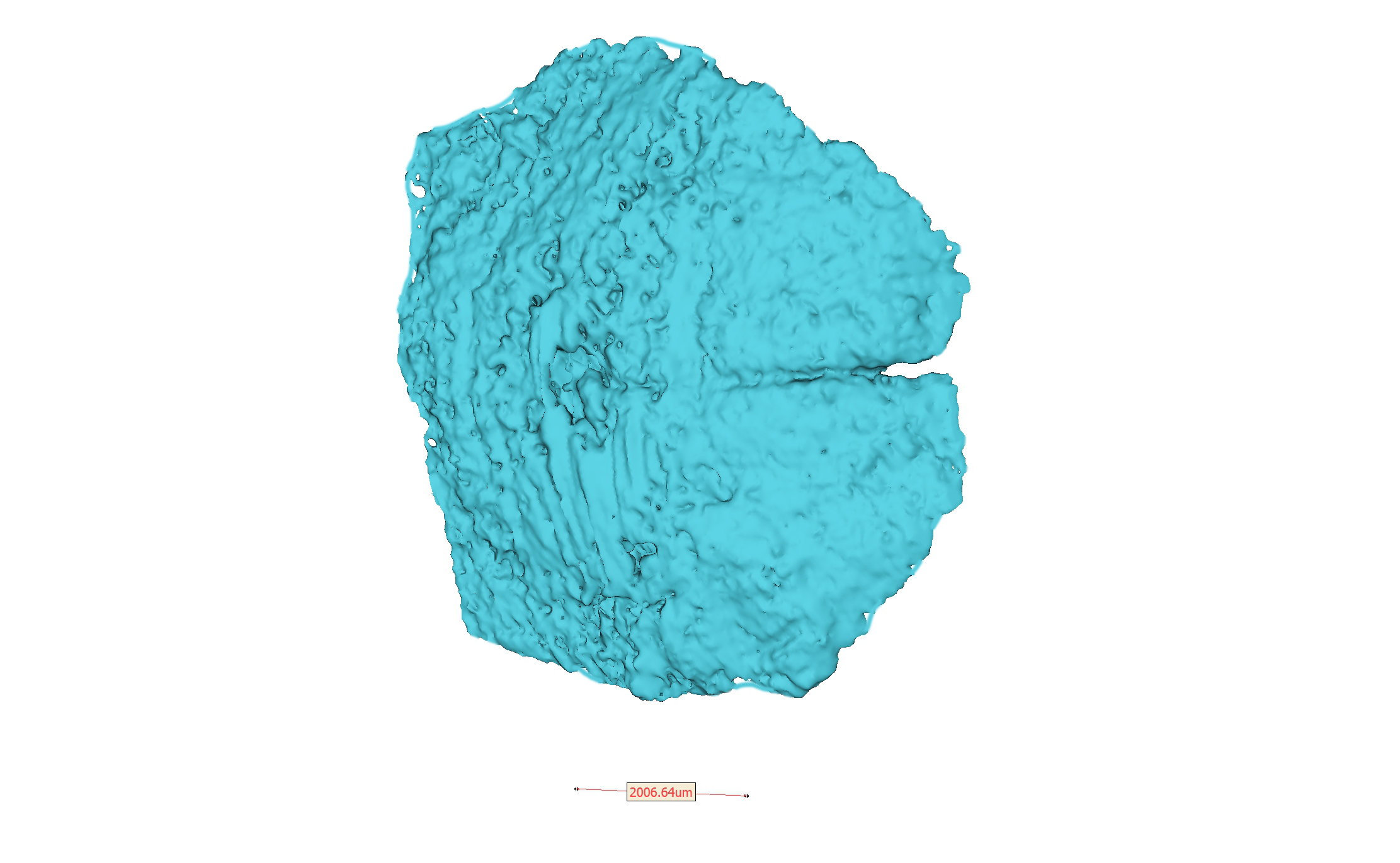

Supplement: Supplementary file 5 — Supplementary Data 2 [file 41467_2023_43557_MOESM5_ESM.zip › Supplementary Data 2/Supplementary Data 2 Raw data of Geometric Morphometric Analyses/12 Morphotypes/Morphotype 3/ll13.jpg]

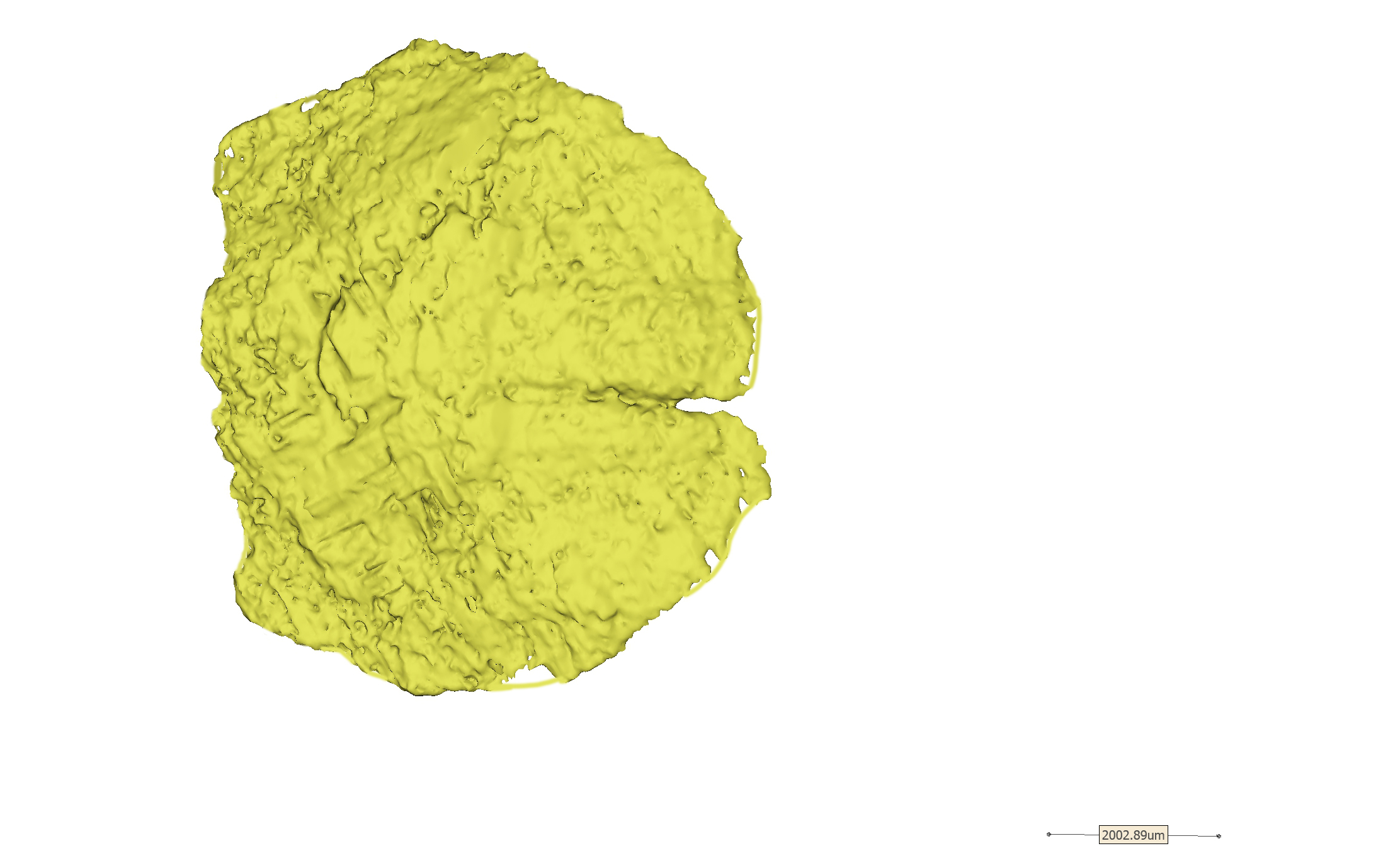

Supplement: Supplementary file 5 — Supplementary Data 2 [file 41467_2023_43557_MOESM5_ESM.zip › Supplementary Data 2/Supplementary Data 2 Raw data of Geometric Morphometric Analyses/12 Morphotypes/Morphotype 3/ll14.jpg]

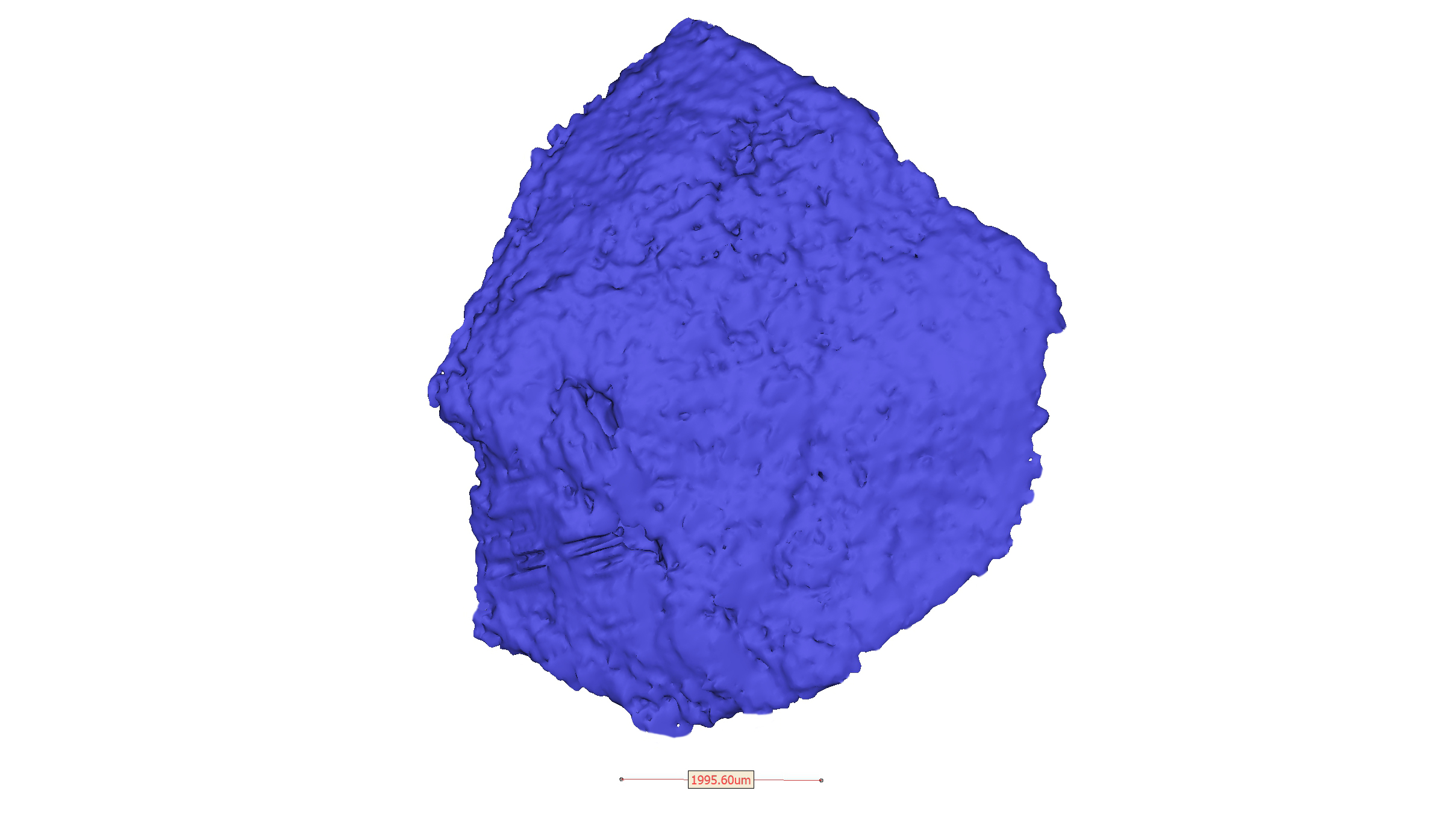

Supplement: Supplementary file 5 — Supplementary Data 2 [file 41467_2023_43557_MOESM5_ESM.zip › Supplementary Data 2/Supplementary Data 2 Raw data of Geometric Morphometric Analyses/12 Morphotypes/Morphotype 4/l1d16.jpg]

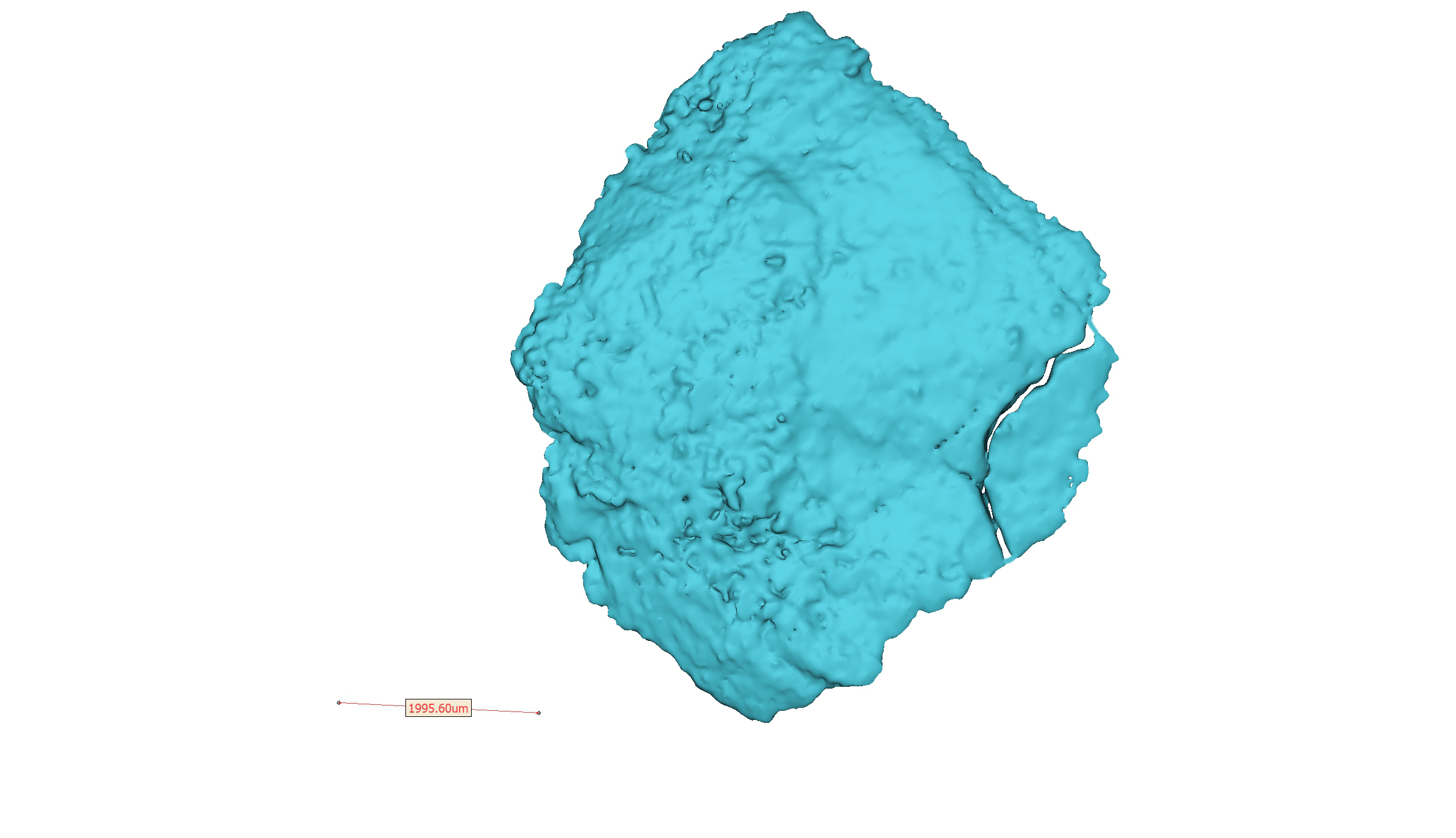

Supplement: Supplementary file 5 — Supplementary Data 2 [file 41467_2023_43557_MOESM5_ESM.zip › Supplementary Data 2/Supplementary Data 2 Raw data of Geometric Morphometric Analyses/12 Morphotypes/Morphotype 4/l1d17.jpg]

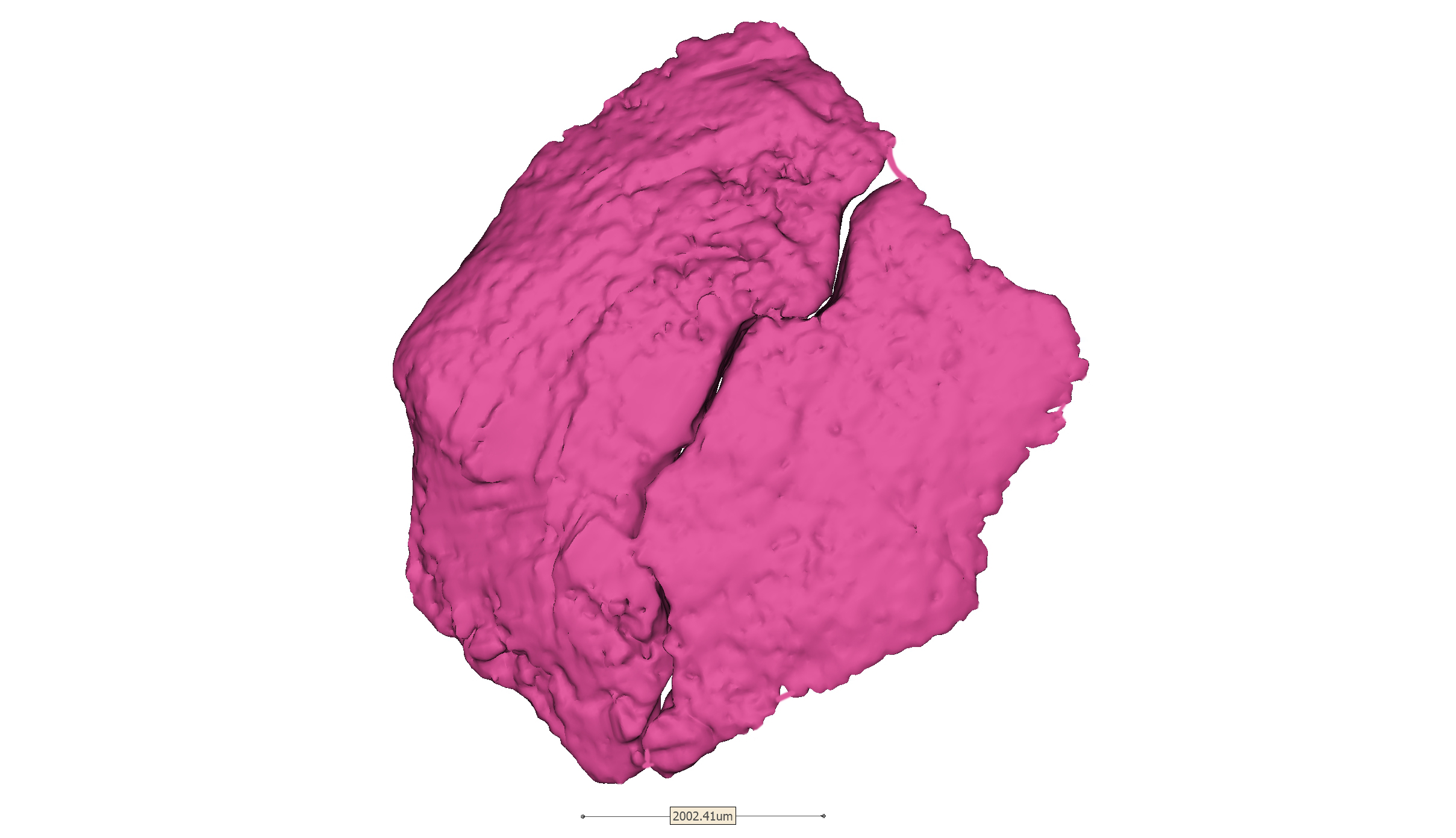

Supplement: Supplementary file 5 — Supplementary Data 2 [file 41467_2023_43557_MOESM5_ESM.zip › Supplementary Data 2/Supplementary Data 2 Raw data of Geometric Morphometric Analyses/12 Morphotypes/Morphotype 4/l1d18.jpg]

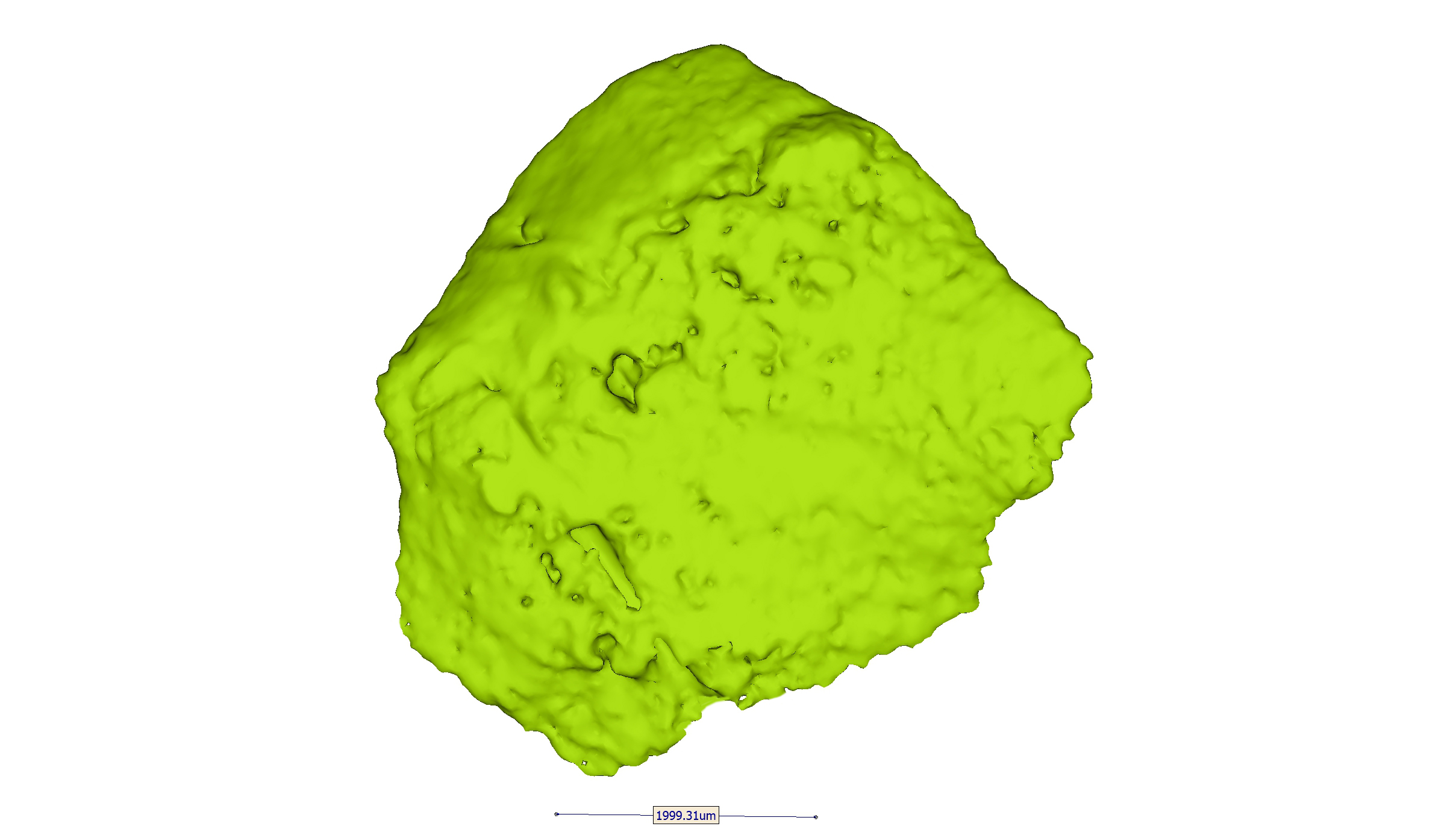

Supplement: Supplementary file 5 — Supplementary Data 2 [file 41467_2023_43557_MOESM5_ESM.zip › Supplementary Data 2/Supplementary Data 2 Raw data of Geometric Morphometric Analyses/12 Morphotypes/Morphotype 4/l1d19.jpg]

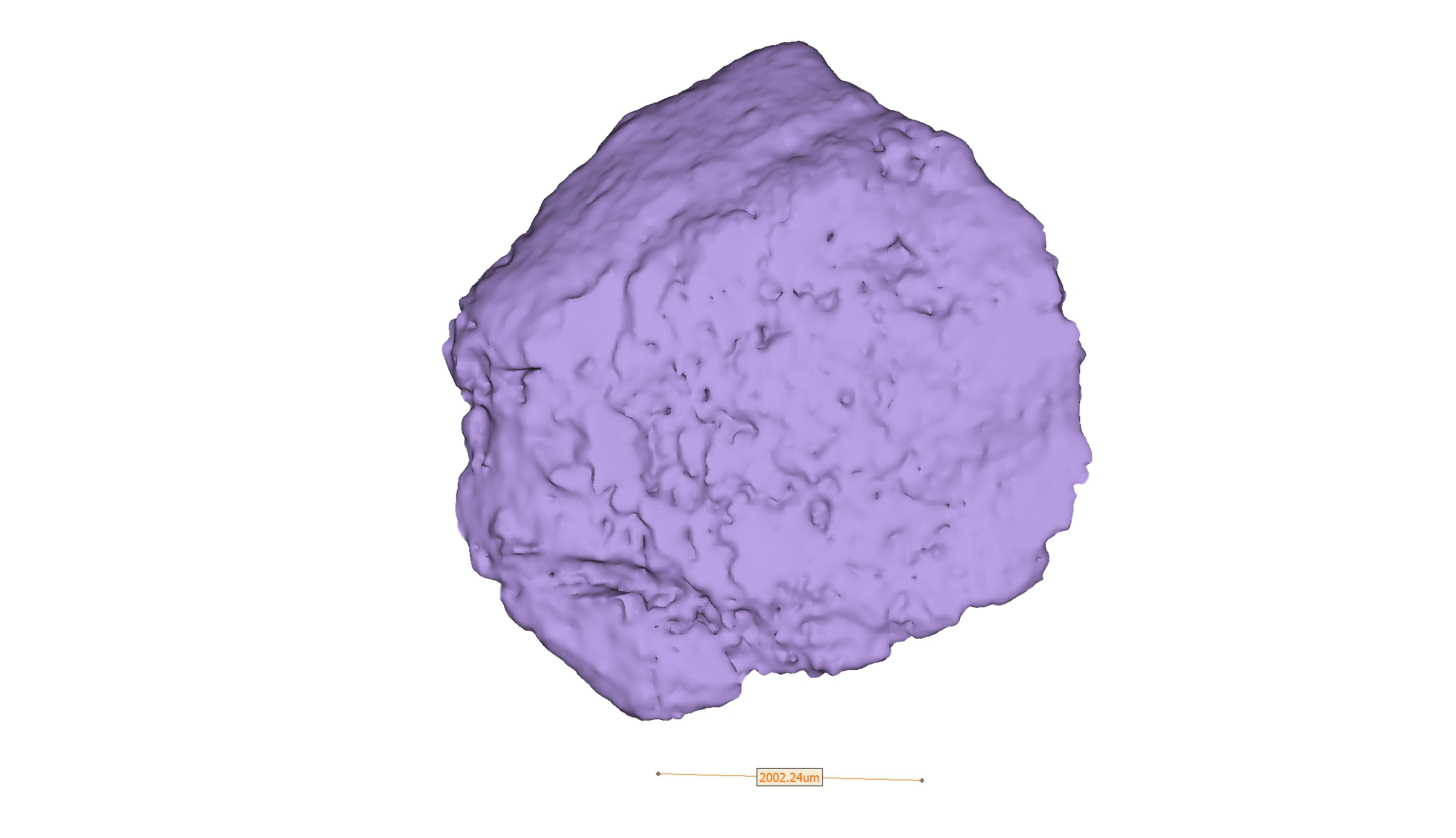

Supplement: Supplementary file 5 — Supplementary Data 2 [file 41467_2023_43557_MOESM5_ESM.zip › Supplementary Data 2/Supplementary Data 2 Raw data of Geometric Morphometric Analyses/12 Morphotypes/Morphotype 4/l1d20.jpg]

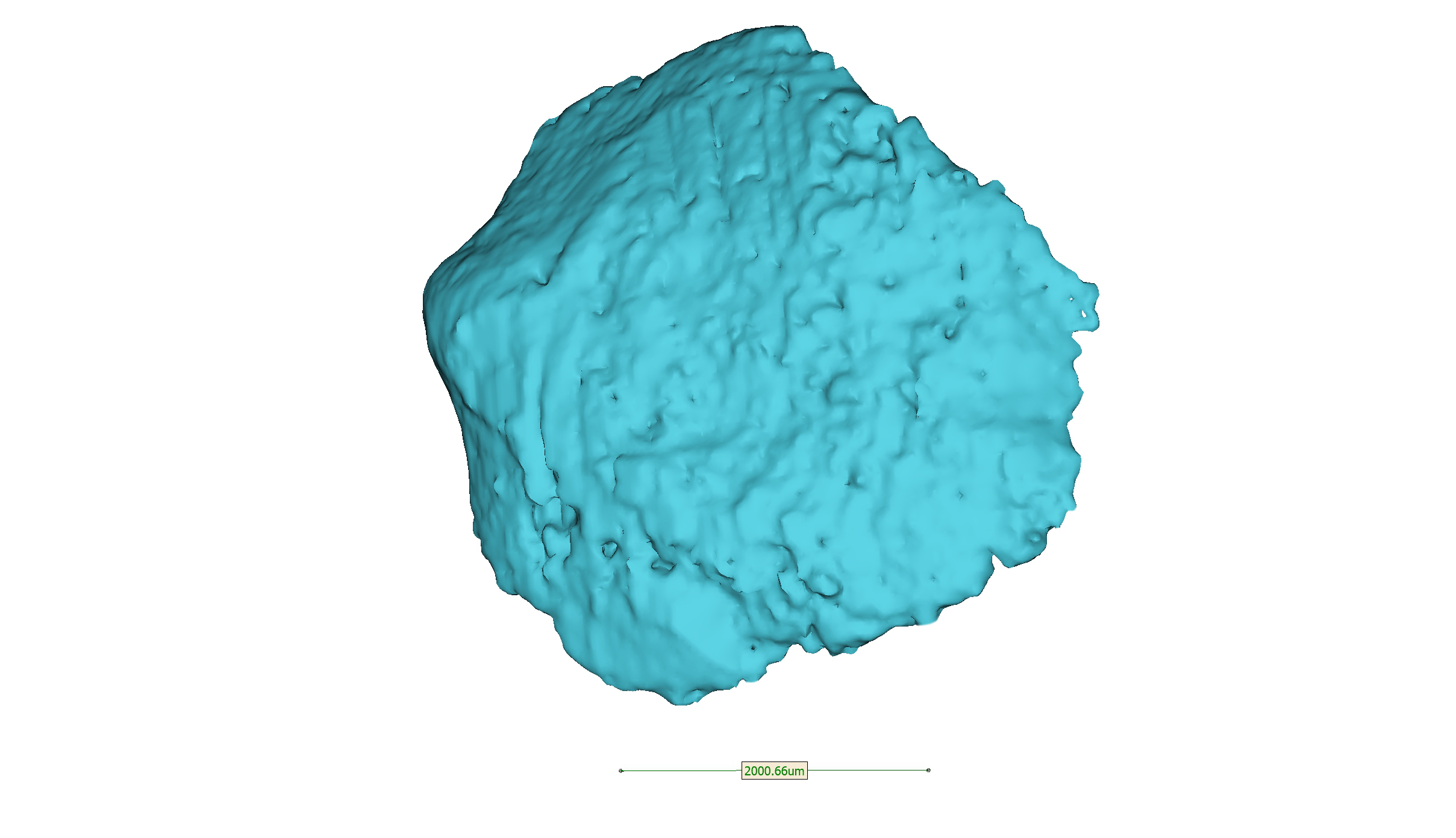

Supplement: Supplementary file 5 — Supplementary Data 2 [file 41467_2023_43557_MOESM5_ESM.zip › Supplementary Data 2/Supplementary Data 2 Raw data of Geometric Morphometric Analyses/12 Morphotypes/Morphotype 4/l1d21.jpg]

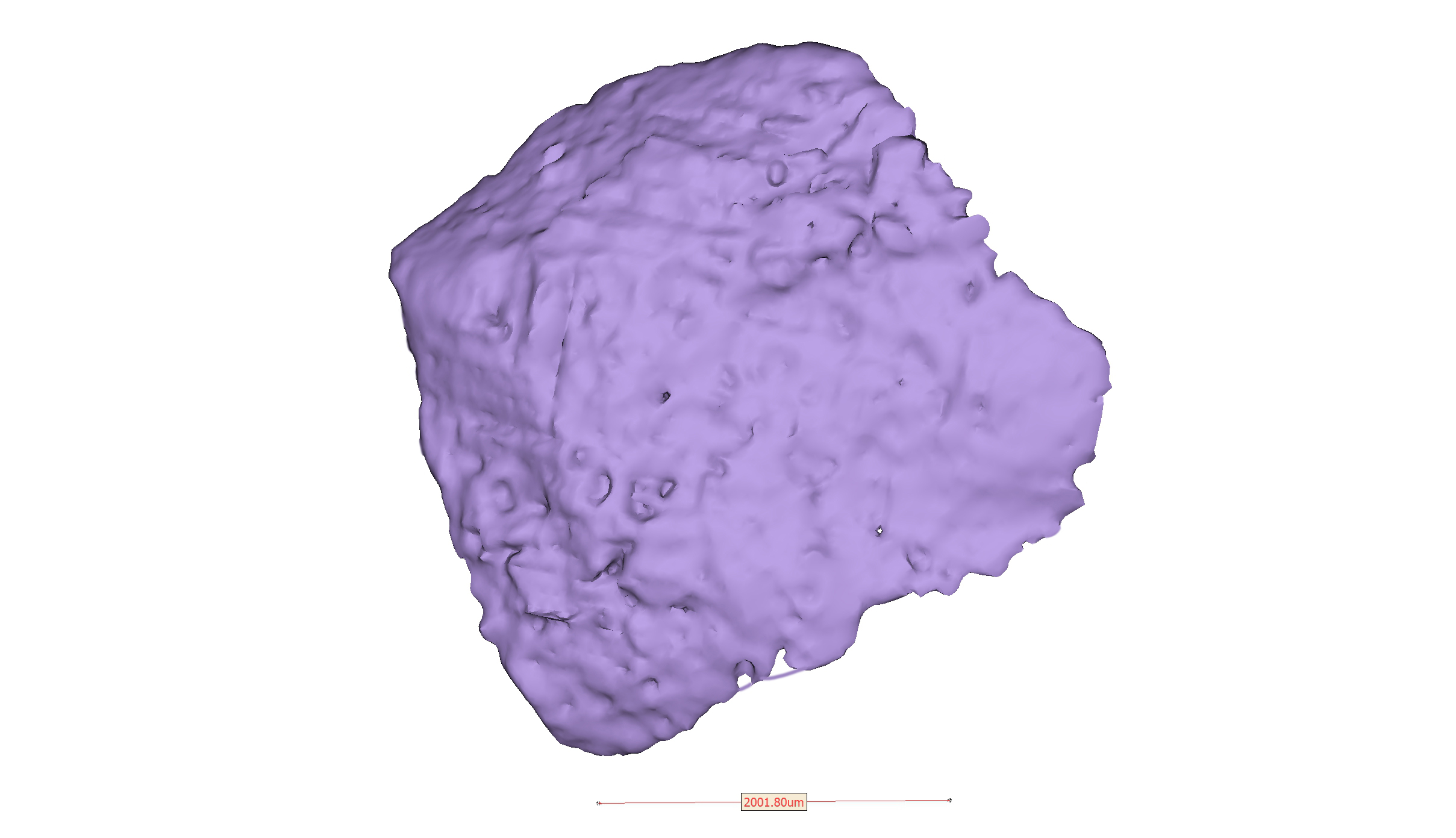

Supplement: Supplementary file 5 — Supplementary Data 2 [file 41467_2023_43557_MOESM5_ESM.zip › Supplementary Data 2/Supplementary Data 2 Raw data of Geometric Morphometric Analyses/12 Morphotypes/Morphotype 4/l1d22.jpg]

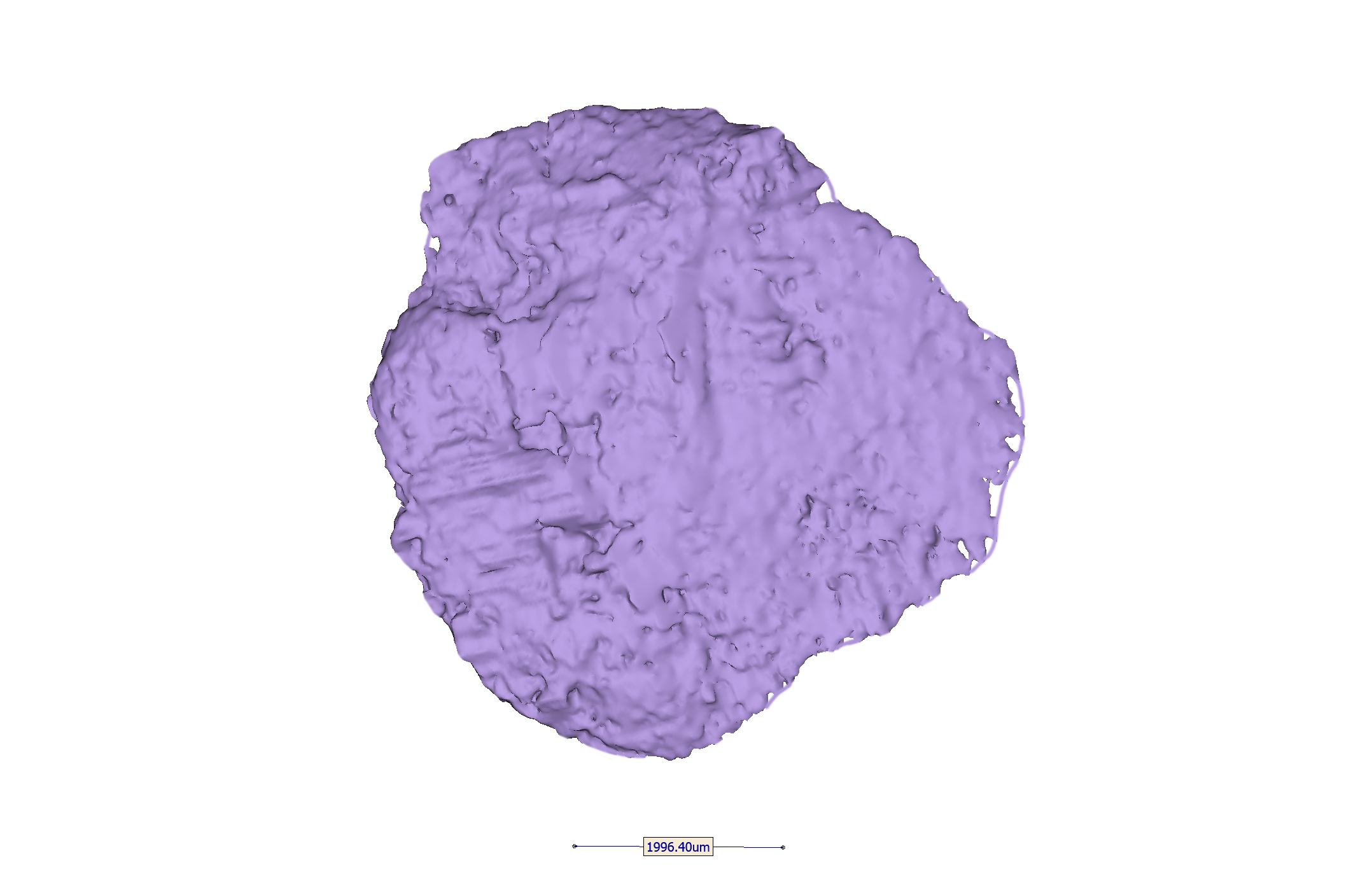

Supplement: Supplementary file 5 — Supplementary Data 2 [file 41467_2023_43557_MOESM5_ESM.zip › Supplementary Data 2/Supplementary Data 2 Raw data of Geometric Morphometric Analyses/12 Morphotypes/Morphotype 4/l1v17.jpg]

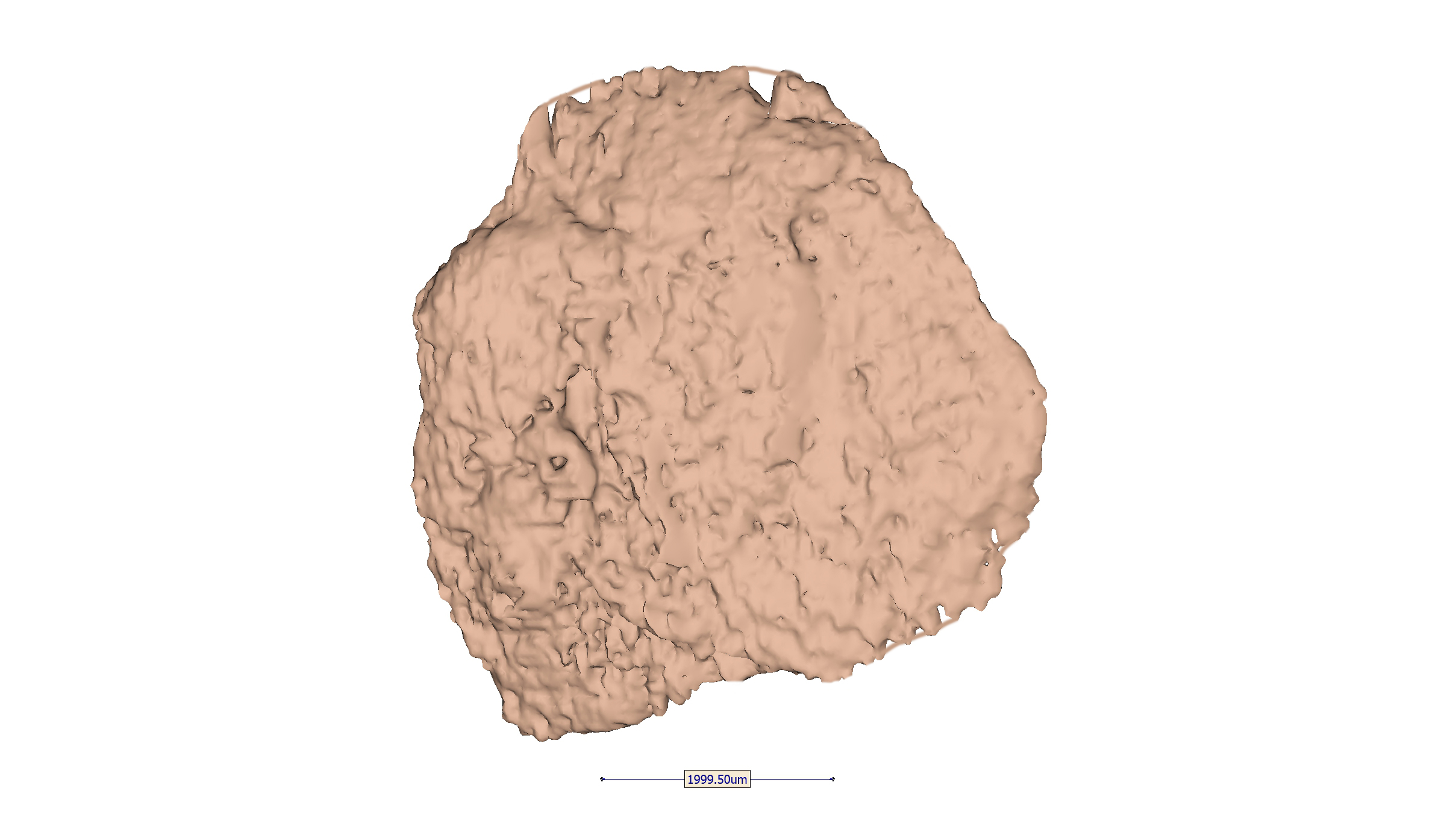

Supplement: Supplementary file 5 — Supplementary Data 2 [file 41467_2023_43557_MOESM5_ESM.zip › Supplementary Data 2/Supplementary Data 2 Raw data of Geometric Morphometric Analyses/12 Morphotypes/Morphotype 4/l1v18.jpg]

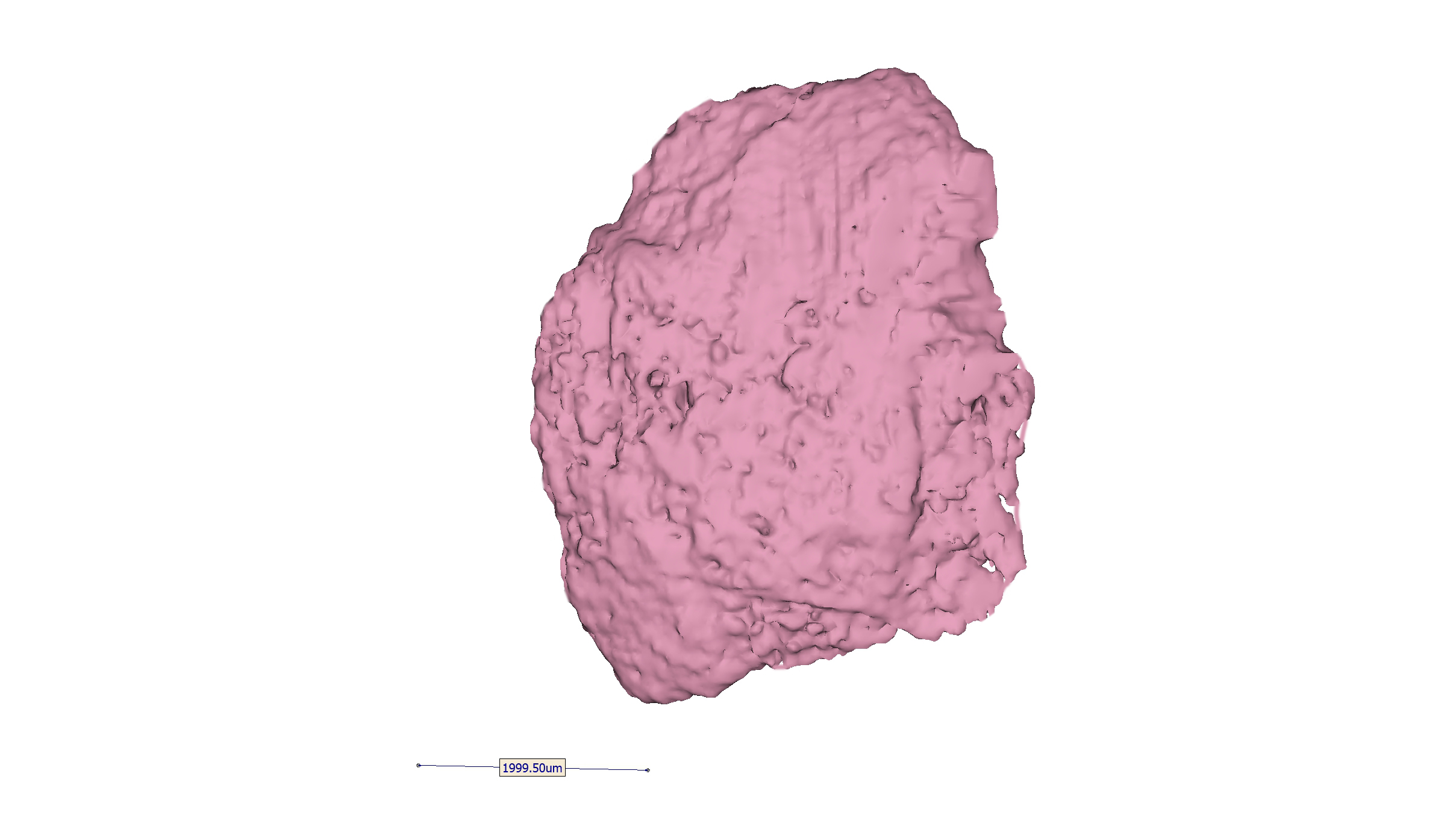

Supplement: Supplementary file 5 — Supplementary Data 2 [file 41467_2023_43557_MOESM5_ESM.zip › Supplementary Data 2/Supplementary Data 2 Raw data of Geometric Morphometric Analyses/12 Morphotypes/Morphotype 4/l1v19.jpg]

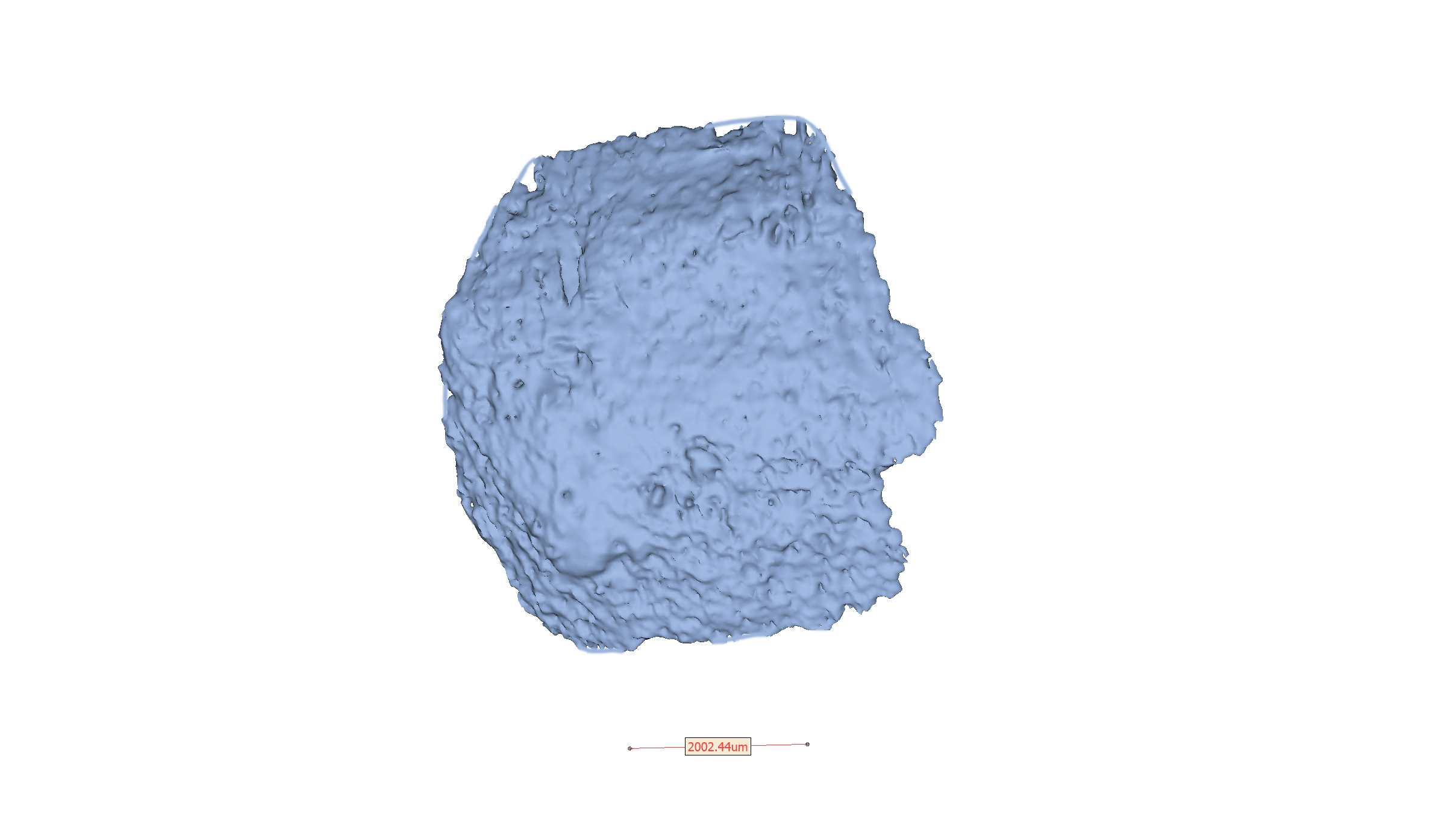

Supplement: Supplementary file 5 — Supplementary Data 2 [file 41467_2023_43557_MOESM5_ESM.zip › Supplementary Data 2/Supplementary Data 2 Raw data of Geometric Morphometric Analyses/12 Morphotypes/Morphotype 4/l2v15.jpg]

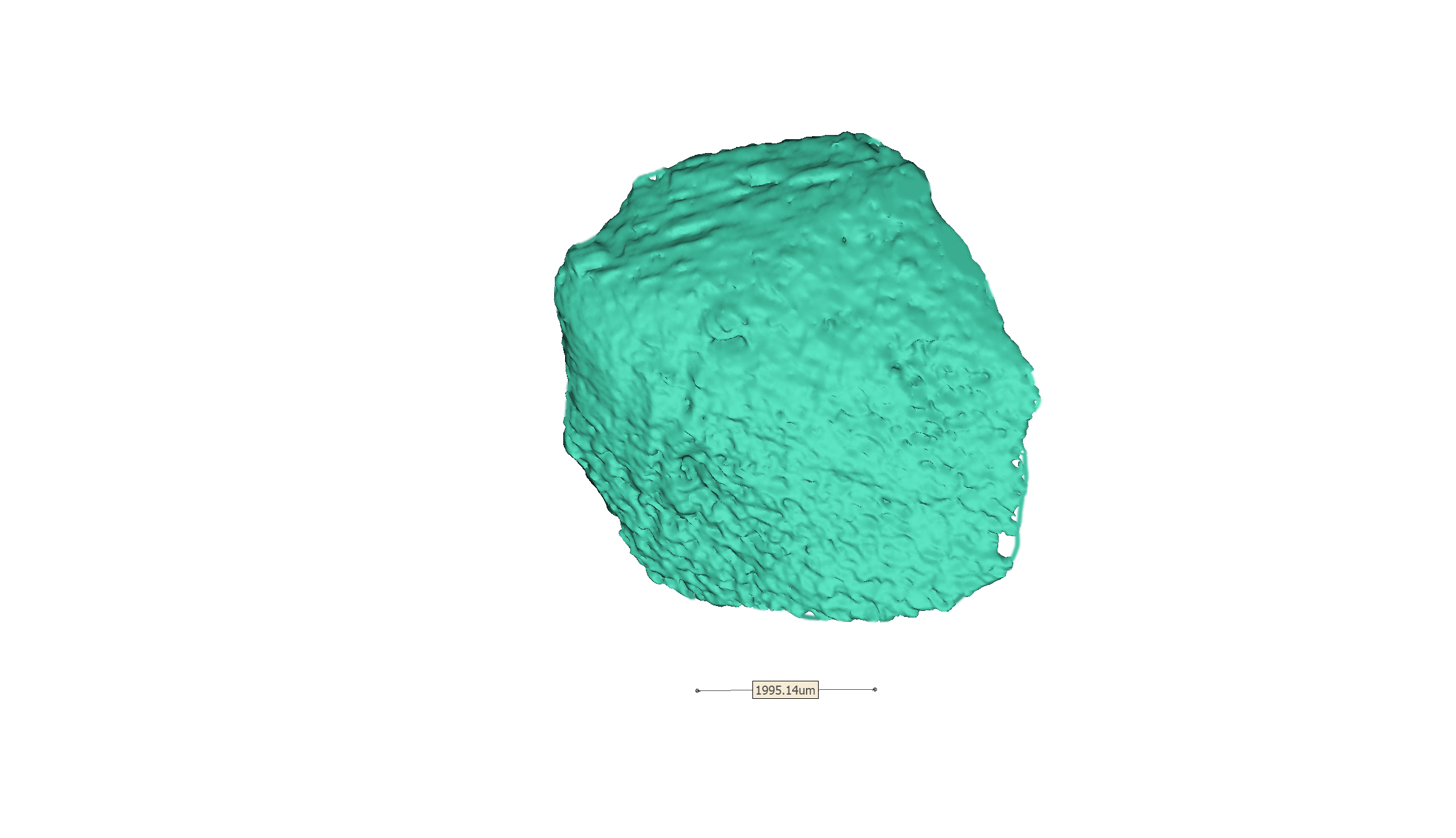

Supplement: Supplementary file 5 — Supplementary Data 2 [file 41467_2023_43557_MOESM5_ESM.zip › Supplementary Data 2/Supplementary Data 2 Raw data of Geometric Morphometric Analyses/12 Morphotypes/Morphotype 4/l2v16.jpg]

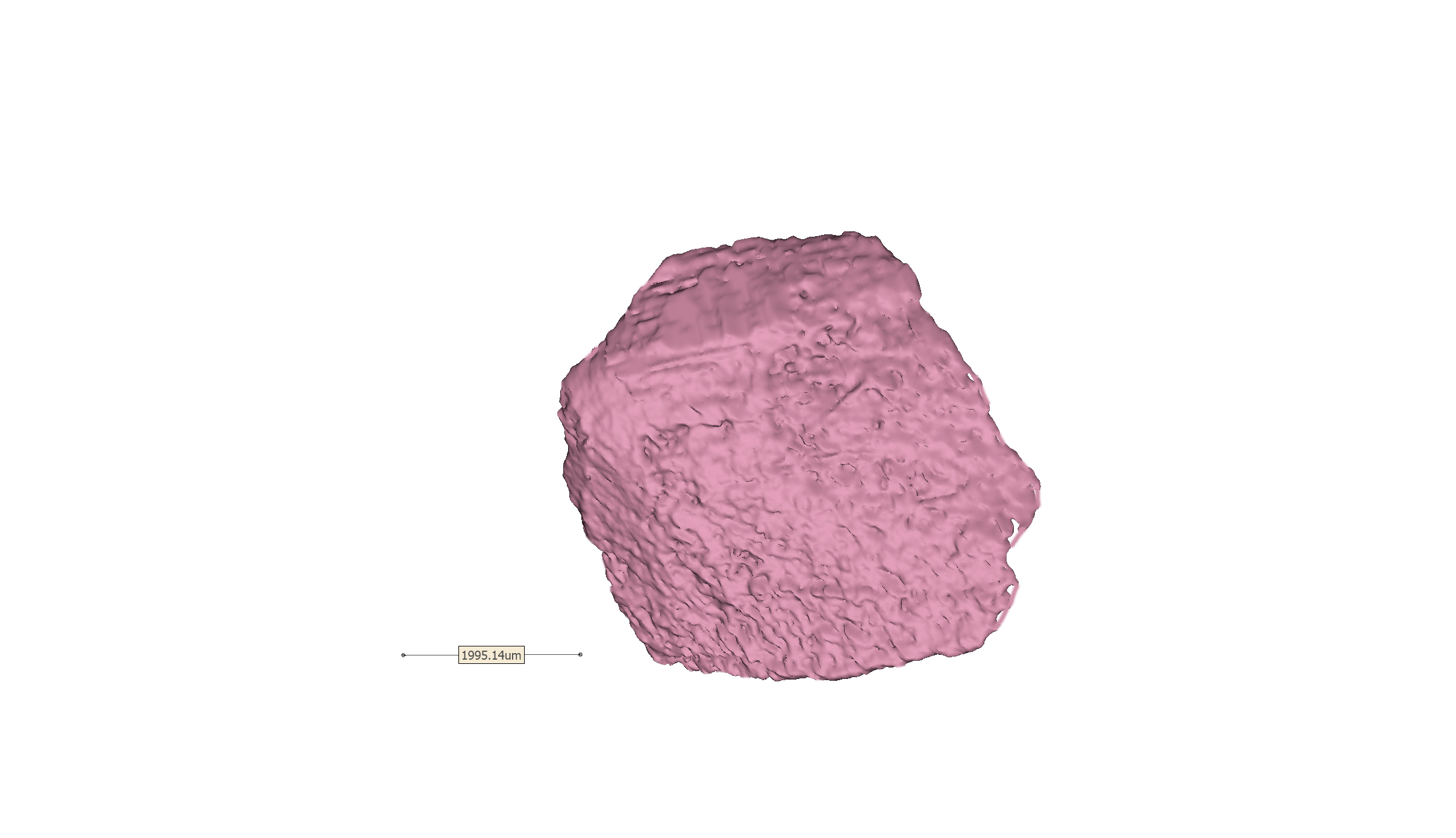

Supplement: Supplementary file 5 — Supplementary Data 2 [file 41467_2023_43557_MOESM5_ESM.zip › Supplementary Data 2/Supplementary Data 2 Raw data of Geometric Morphometric Analyses/12 Morphotypes/Morphotype 4/l2v17.jpg]

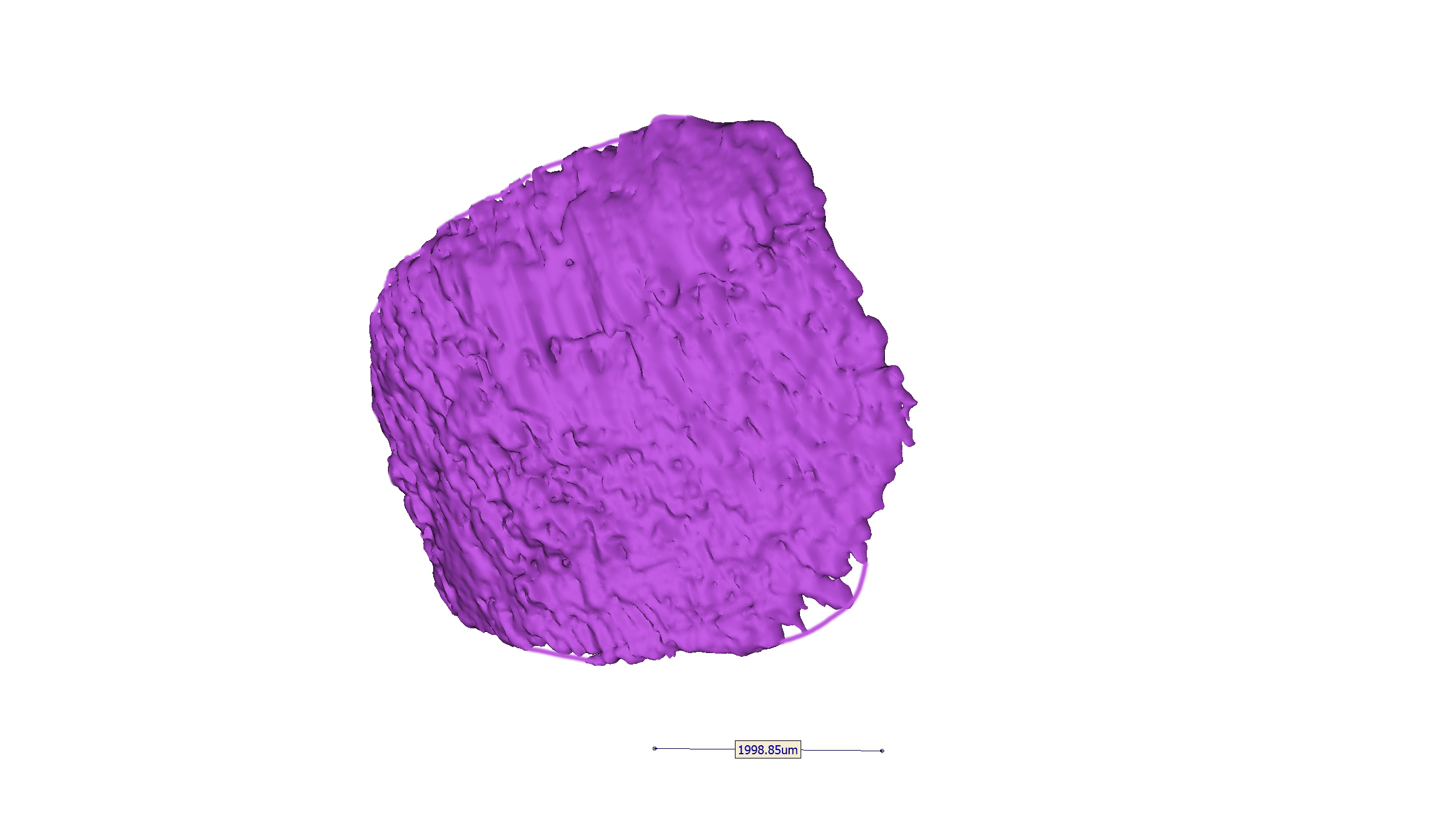

Supplement: Supplementary file 5 — Supplementary Data 2 [file 41467_2023_43557_MOESM5_ESM.zip › Supplementary Data 2/Supplementary Data 2 Raw data of Geometric Morphometric Analyses/12 Morphotypes/Morphotype 4/l2v18.jpg]

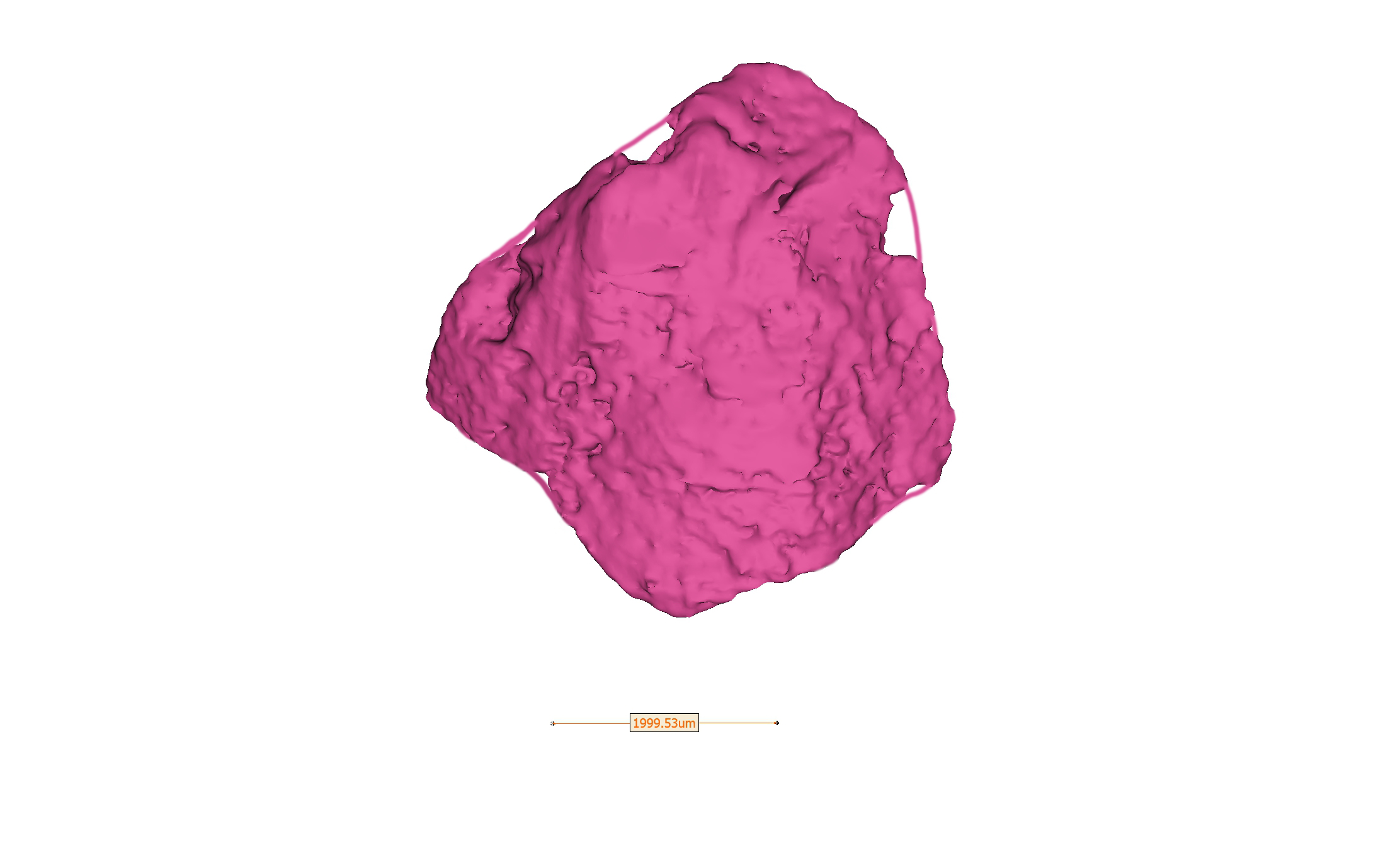

Supplement: Supplementary file 5 — Supplementary Data 2 [file 41467_2023_43557_MOESM5_ESM.zip › Supplementary Data 2/Supplementary Data 2 Raw data of Geometric Morphometric Analyses/12 Morphotypes/Morphotype 4/l6v10.jpg]

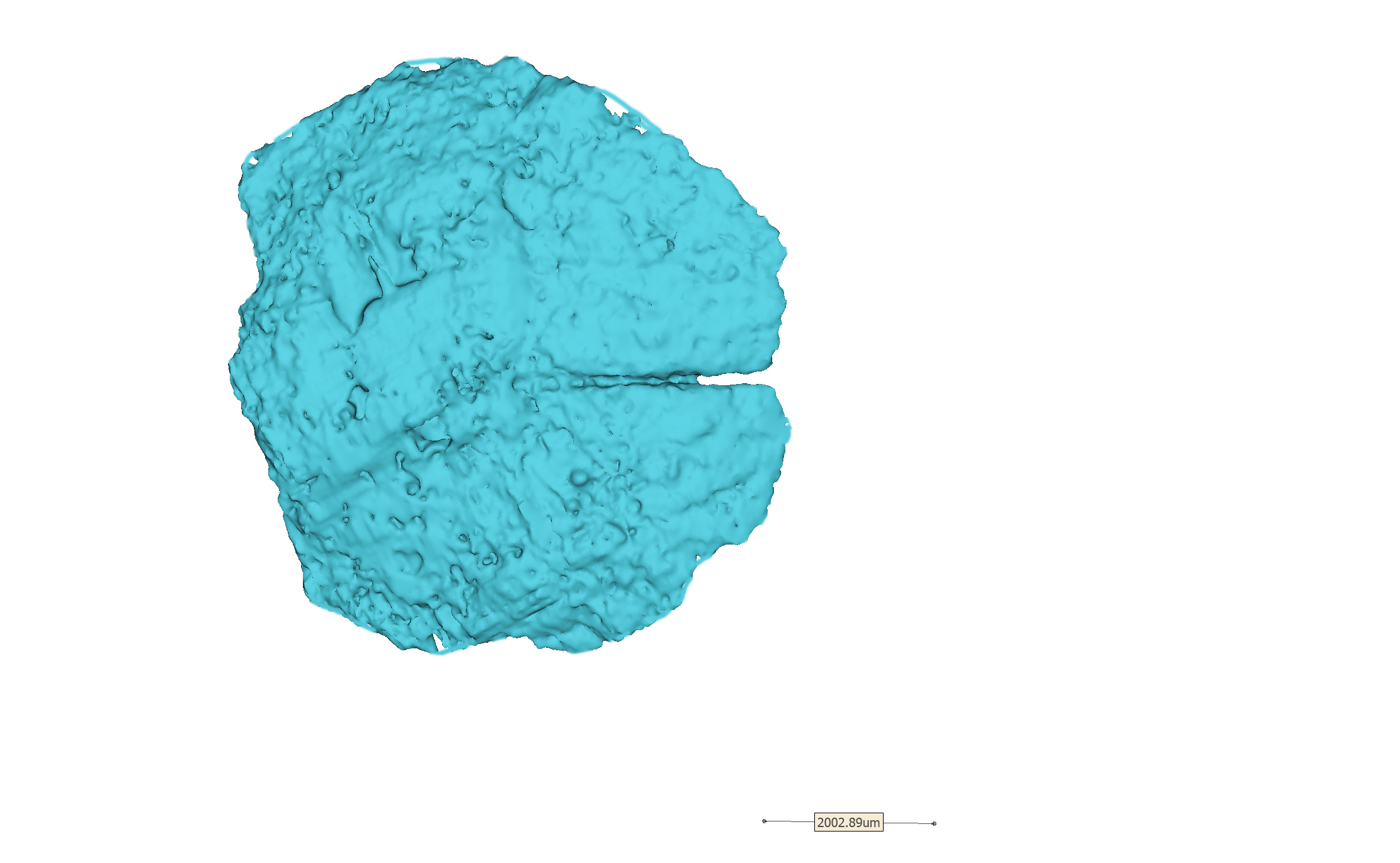

Supplement: Supplementary file 5 — Supplementary Data 2 [file 41467_2023_43557_MOESM5_ESM.zip › Supplementary Data 2/Supplementary Data 2 Raw data of Geometric Morphometric Analyses/12 Morphotypes/Morphotype 4/ll15.jpg]

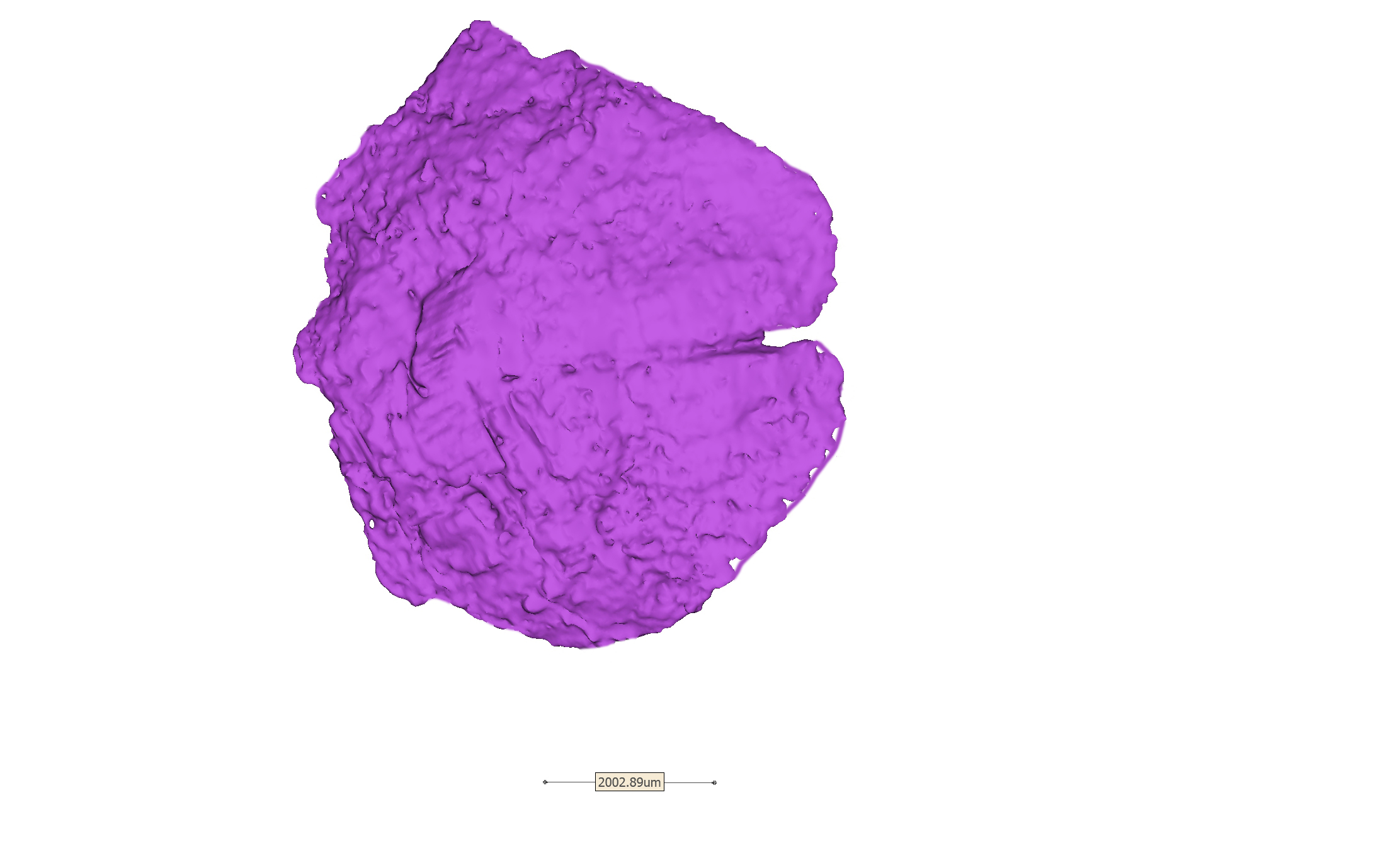

Supplement: Supplementary file 5 — Supplementary Data 2 [file 41467_2023_43557_MOESM5_ESM.zip › Supplementary Data 2/Supplementary Data 2 Raw data of Geometric Morphometric Analyses/12 Morphotypes/Morphotype 4/ll16.jpg]

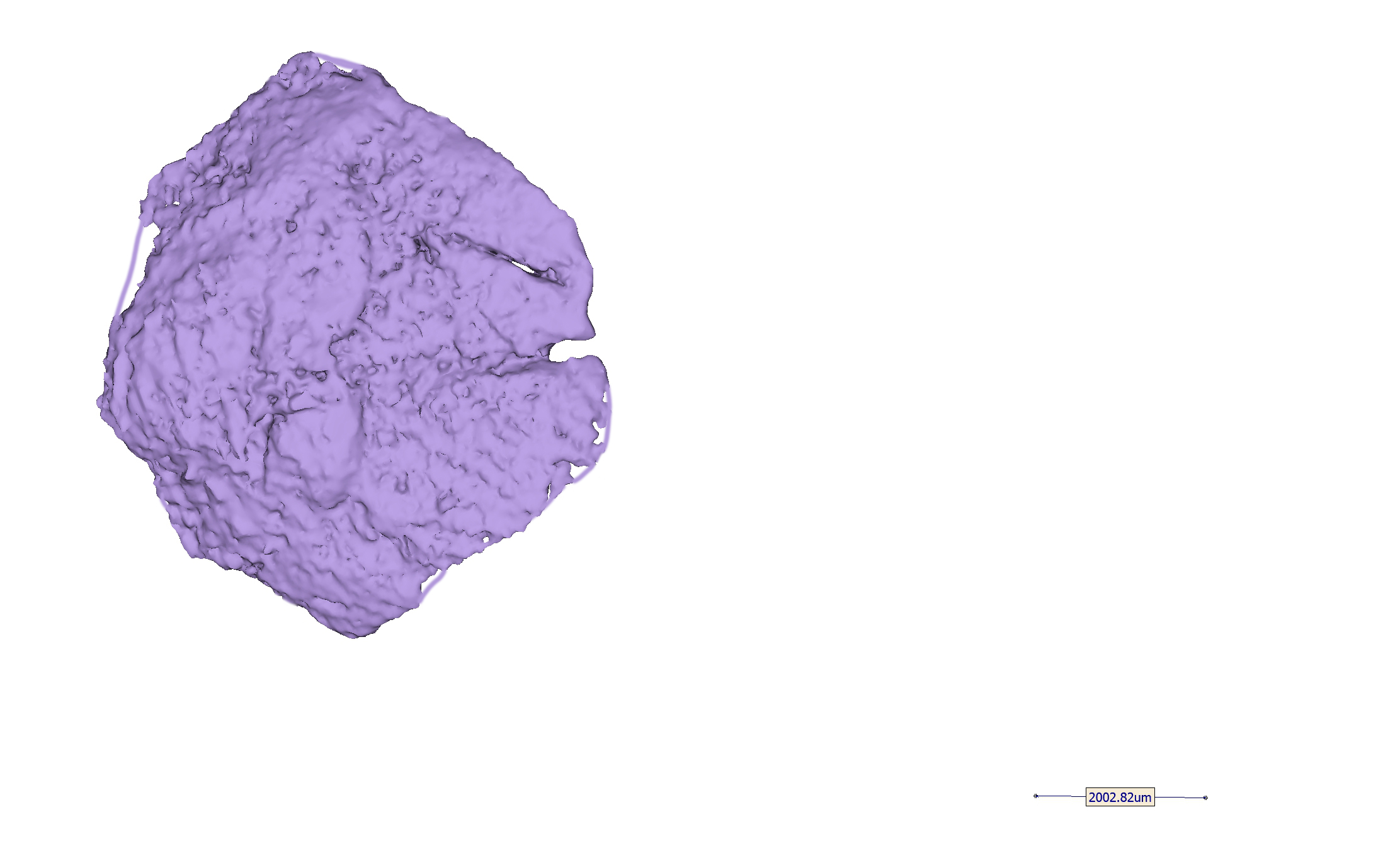

Supplement: Supplementary file 5 — Supplementary Data 2 [file 41467_2023_43557_MOESM5_ESM.zip › Supplementary Data 2/Supplementary Data 2 Raw data of Geometric Morphometric Analyses/12 Morphotypes/Morphotype 4/ll17.jpg]

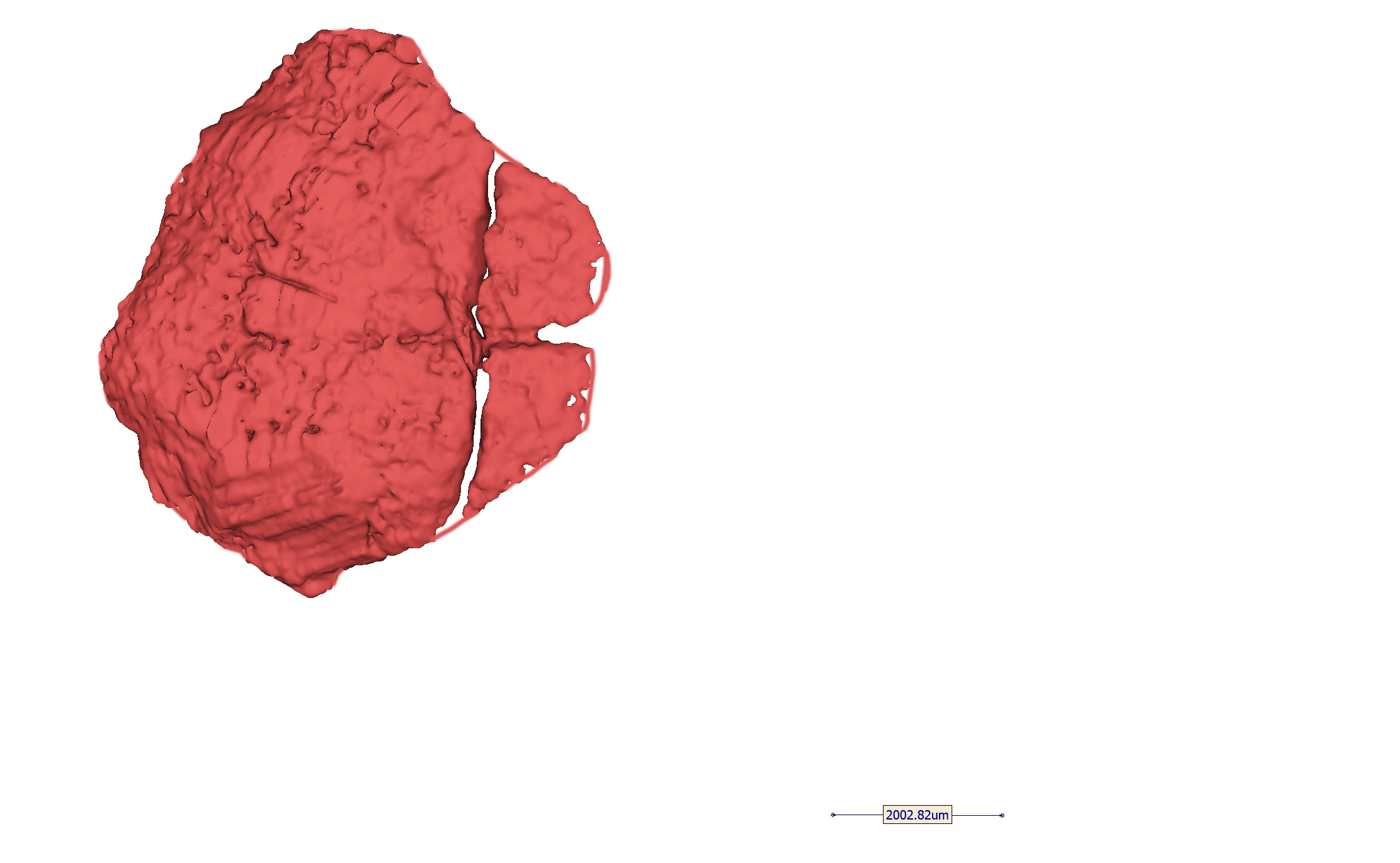

Supplement: Supplementary file 5 — Supplementary Data 2 [file 41467_2023_43557_MOESM5_ESM.zip › Supplementary Data 2/Supplementary Data 2 Raw data of Geometric Morphometric Analyses/12 Morphotypes/Morphotype 4/ll18.jpg]

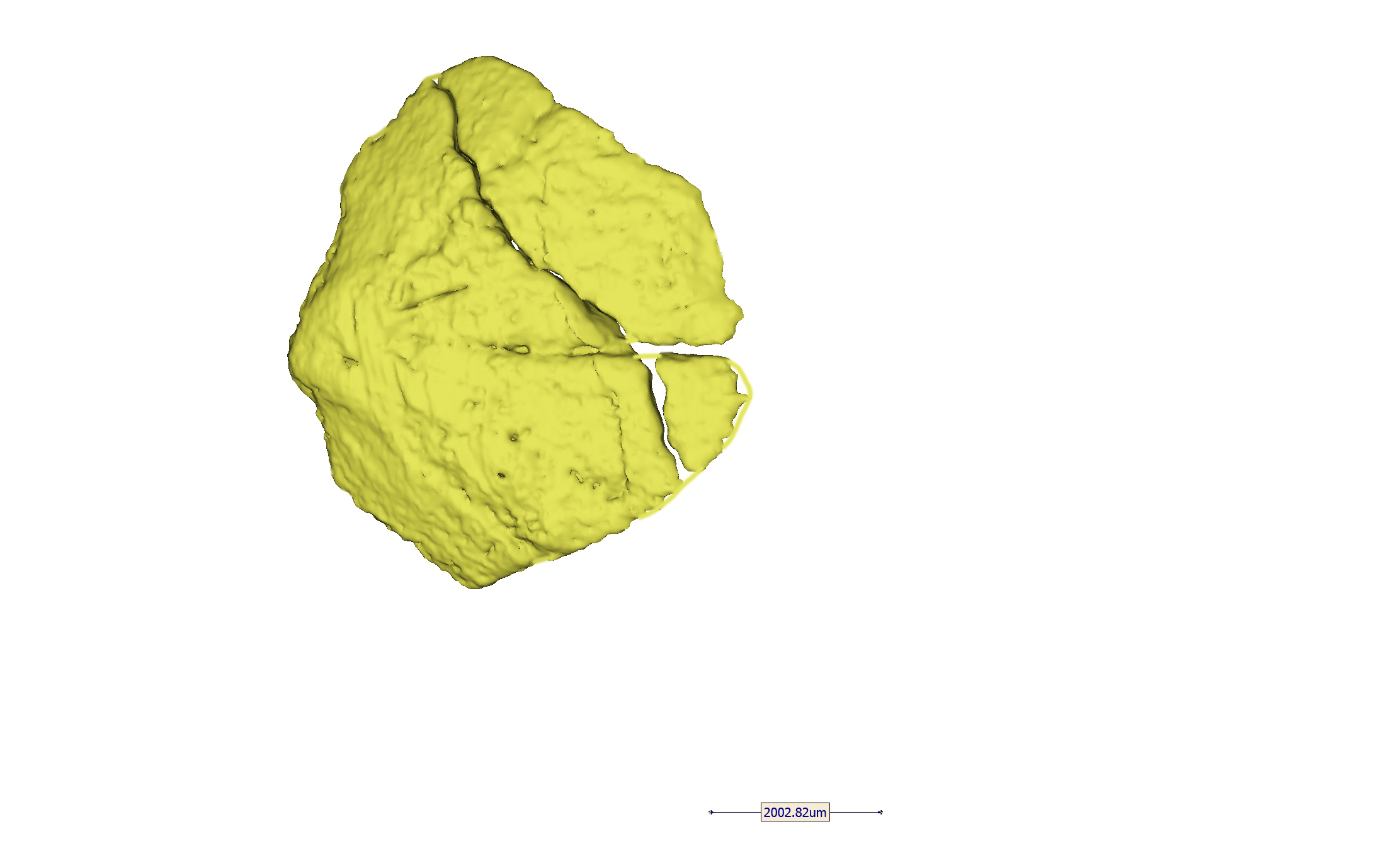

Supplement: Supplementary file 5 — Supplementary Data 2 [file 41467_2023_43557_MOESM5_ESM.zip › Supplementary Data 2/Supplementary Data 2 Raw data of Geometric Morphometric Analyses/12 Morphotypes/Morphotype 4/ll19.jpg]

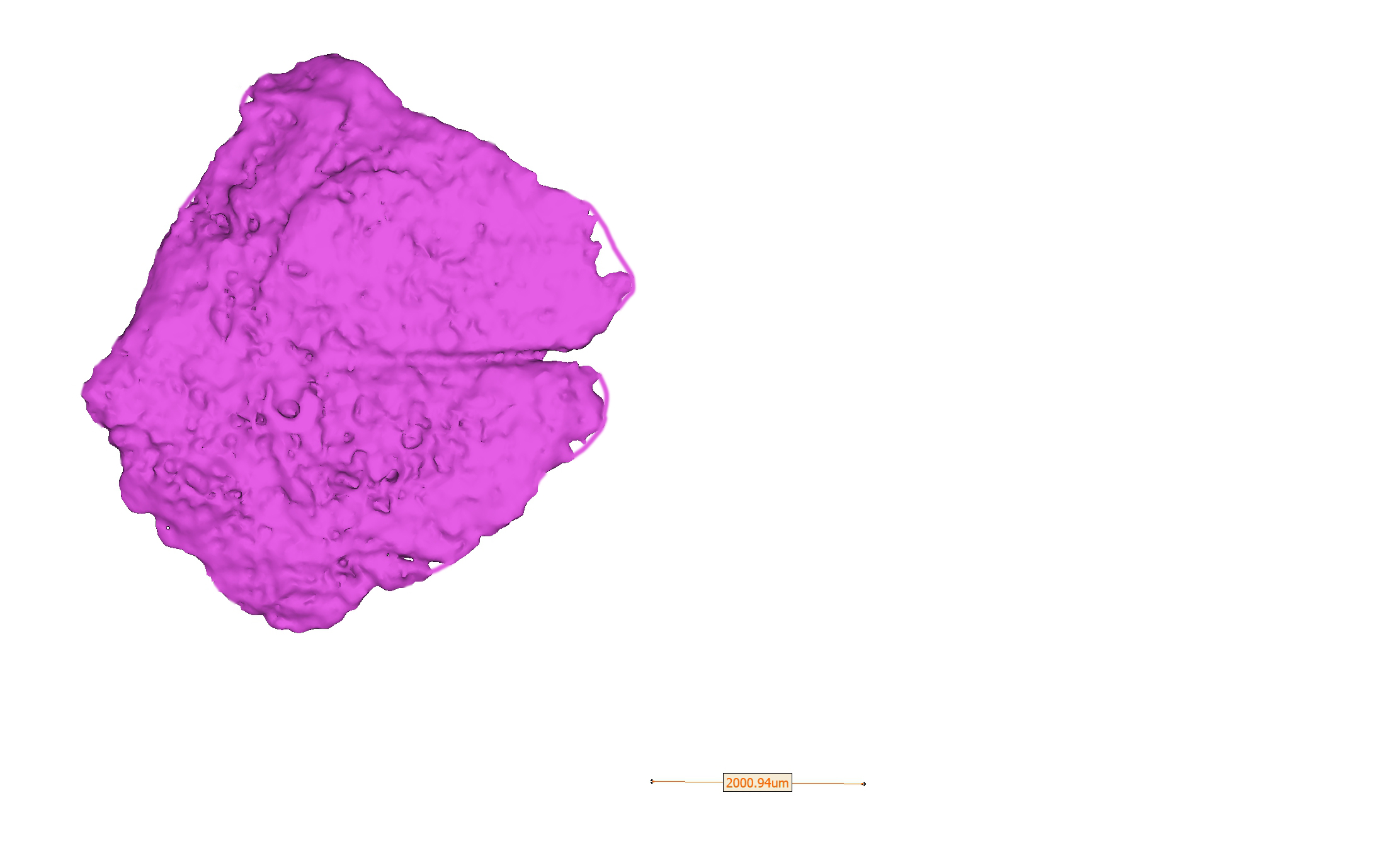

Supplement: Supplementary file 5 — Supplementary Data 2 [file 41467_2023_43557_MOESM5_ESM.zip › Supplementary Data 2/Supplementary Data 2 Raw data of Geometric Morphometric Analyses/12 Morphotypes/Morphotype 4/ll20.jpg]

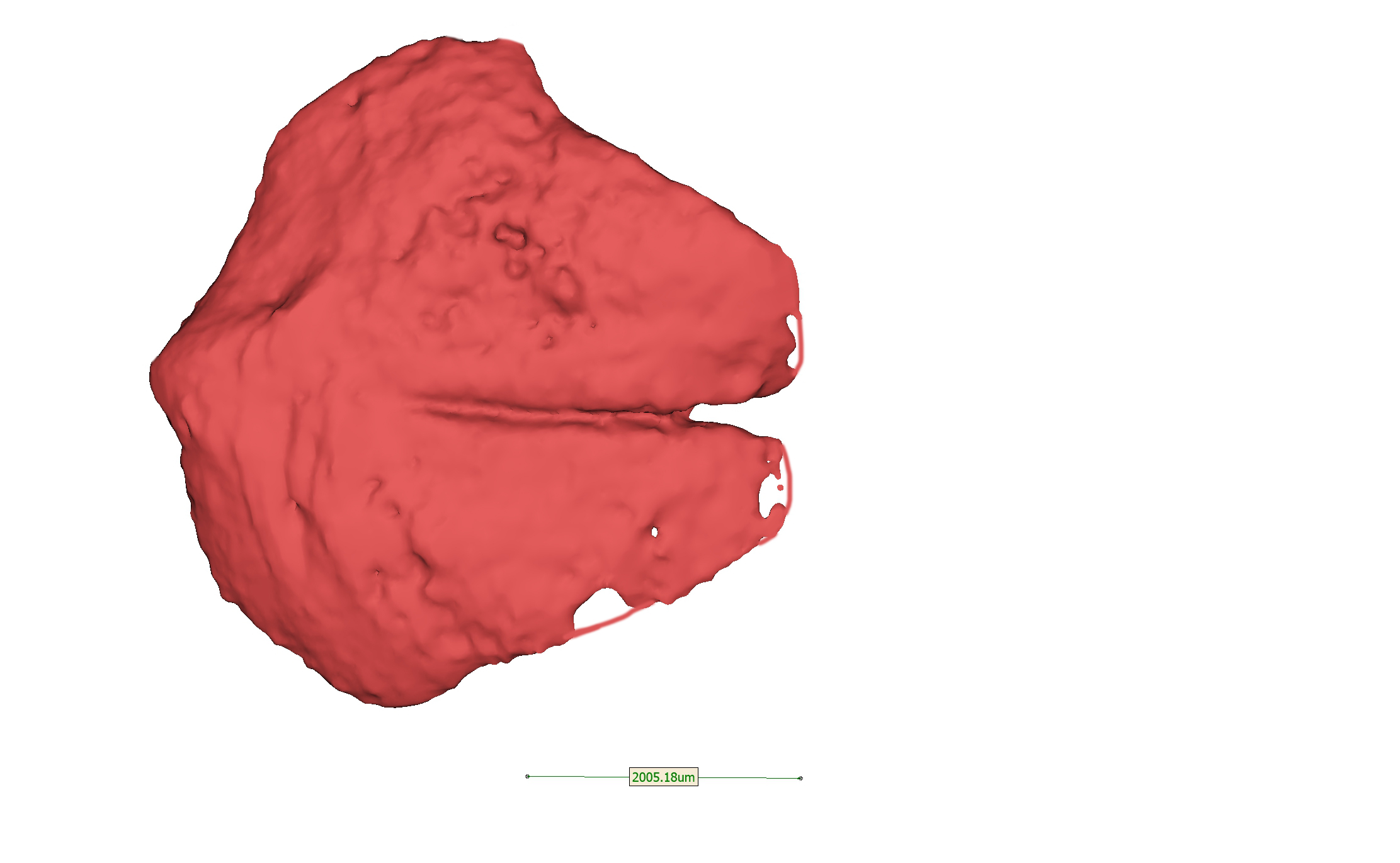

Supplement: Supplementary file 5 — Supplementary Data 2 [file 41467_2023_43557_MOESM5_ESM.zip › Supplementary Data 2/Supplementary Data 2 Raw data of Geometric Morphometric Analyses/12 Morphotypes/Morphotype 4/ll21.jpg]

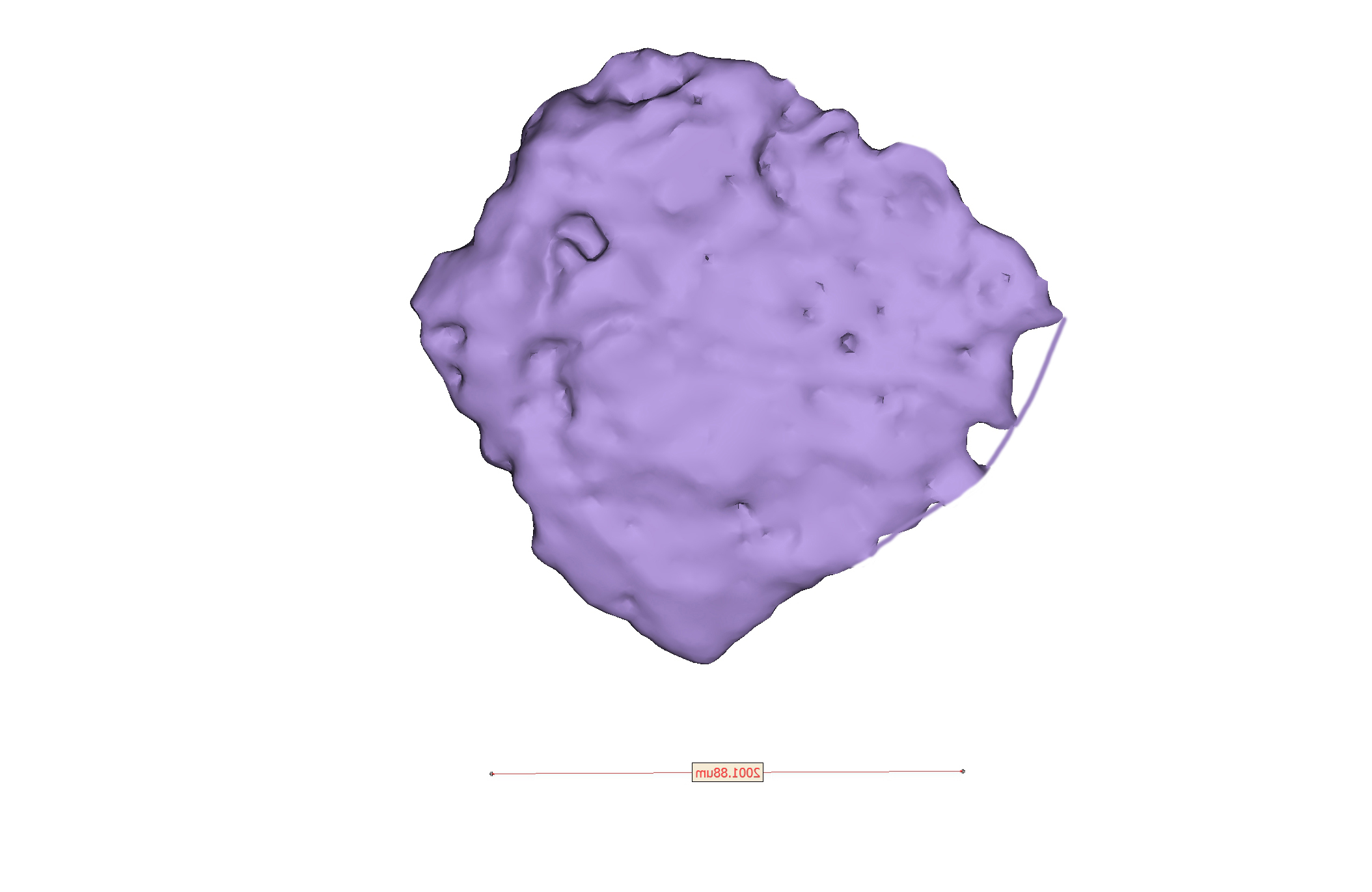

Supplement: Supplementary file 5 — Supplementary Data 2 [file 41467_2023_43557_MOESM5_ESM.zip › Supplementary Data 2/Supplementary Data 2 Raw data of Geometric Morphometric Analyses/12 Morphotypes/Morphotype 4/t14r.jpg]

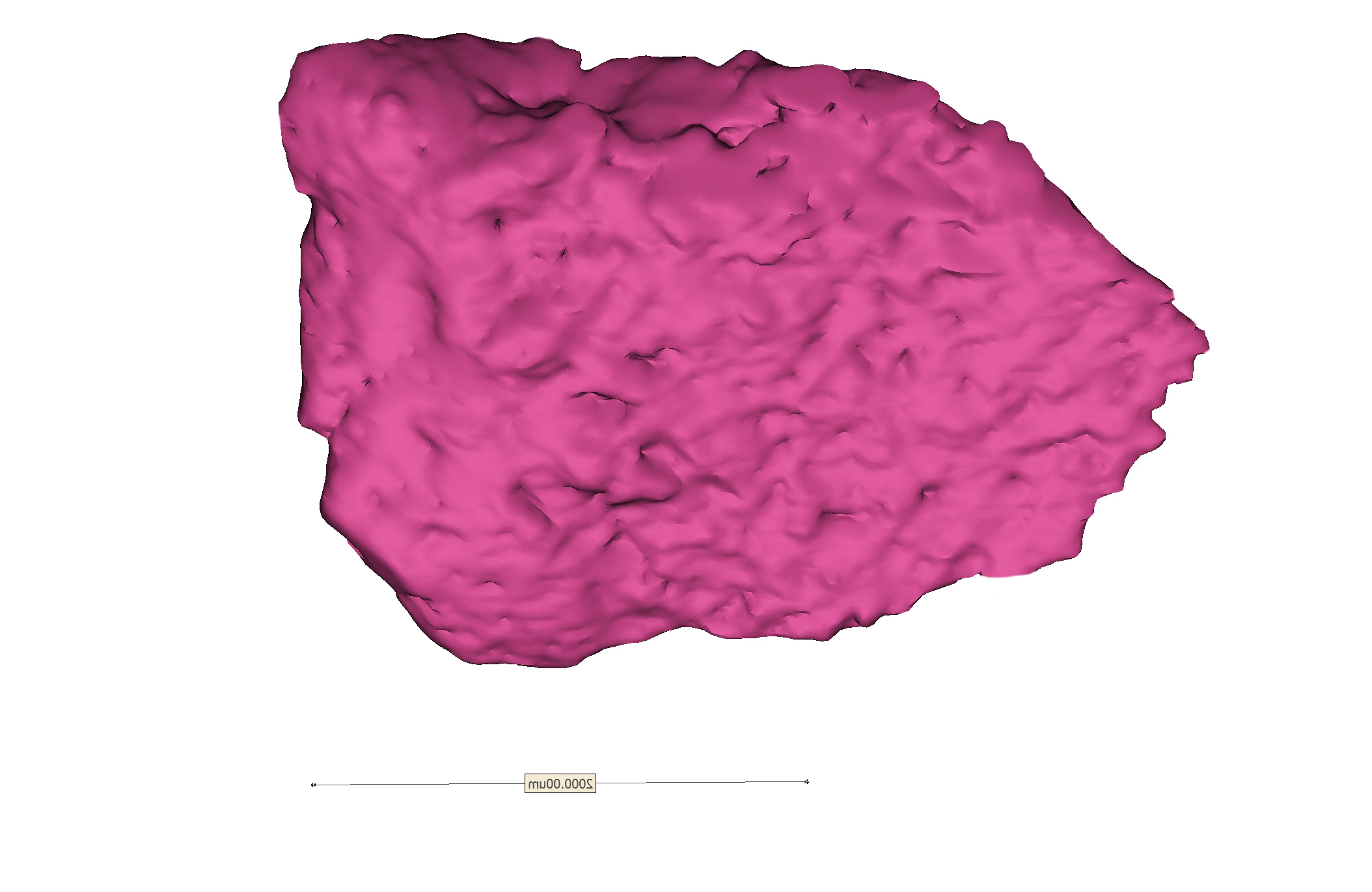

Supplement: Supplementary file 5 — Supplementary Data 2 [file 41467_2023_43557_MOESM5_ESM.zip › Supplementary Data 2/Supplementary Data 2 Raw data of Geometric Morphometric Analyses/12 Morphotypes/Morphotype 5/l1d23-.jpg]

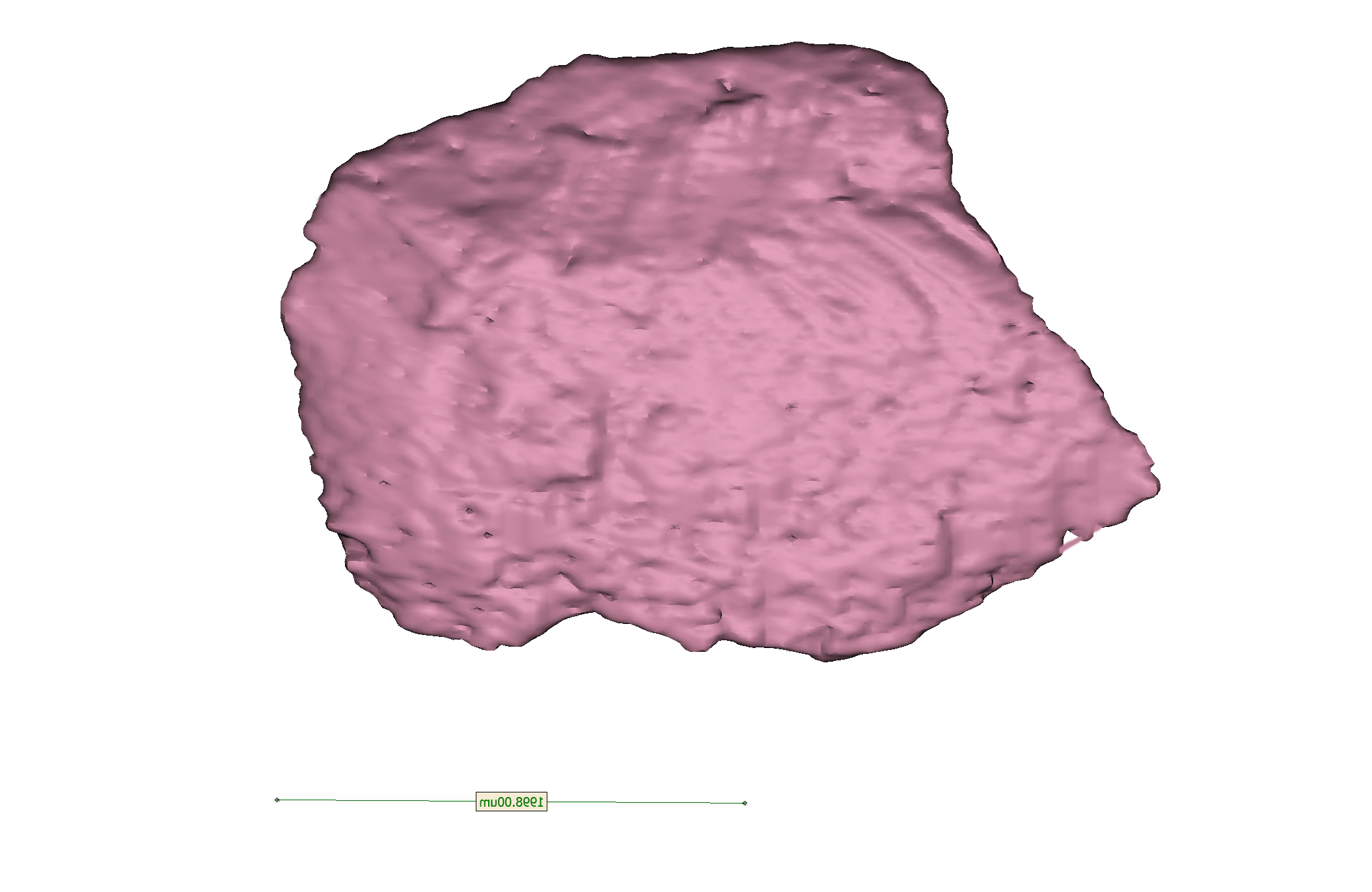

Supplement: Supplementary file 5 — Supplementary Data 2 [file 41467_2023_43557_MOESM5_ESM.zip › Supplementary Data 2/Supplementary Data 2 Raw data of Geometric Morphometric Analyses/12 Morphotypes/Morphotype 5/l1d24-.jpg]

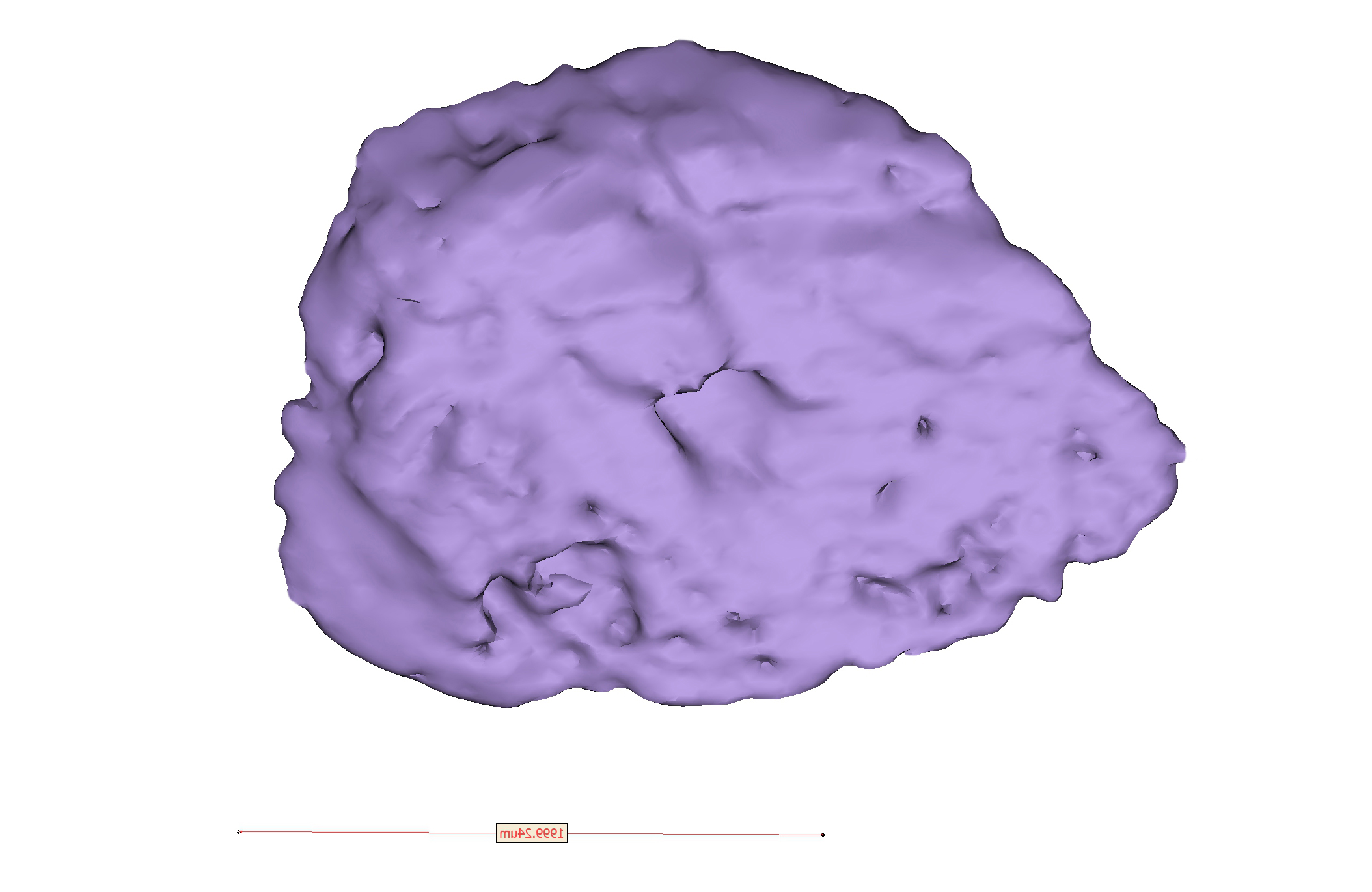

Supplement: Supplementary file 5 — Supplementary Data 2 [file 41467_2023_43557_MOESM5_ESM.zip › Supplementary Data 2/Supplementary Data 2 Raw data of Geometric Morphometric Analyses/12 Morphotypes/Morphotype 5/l1d25-.jpg]

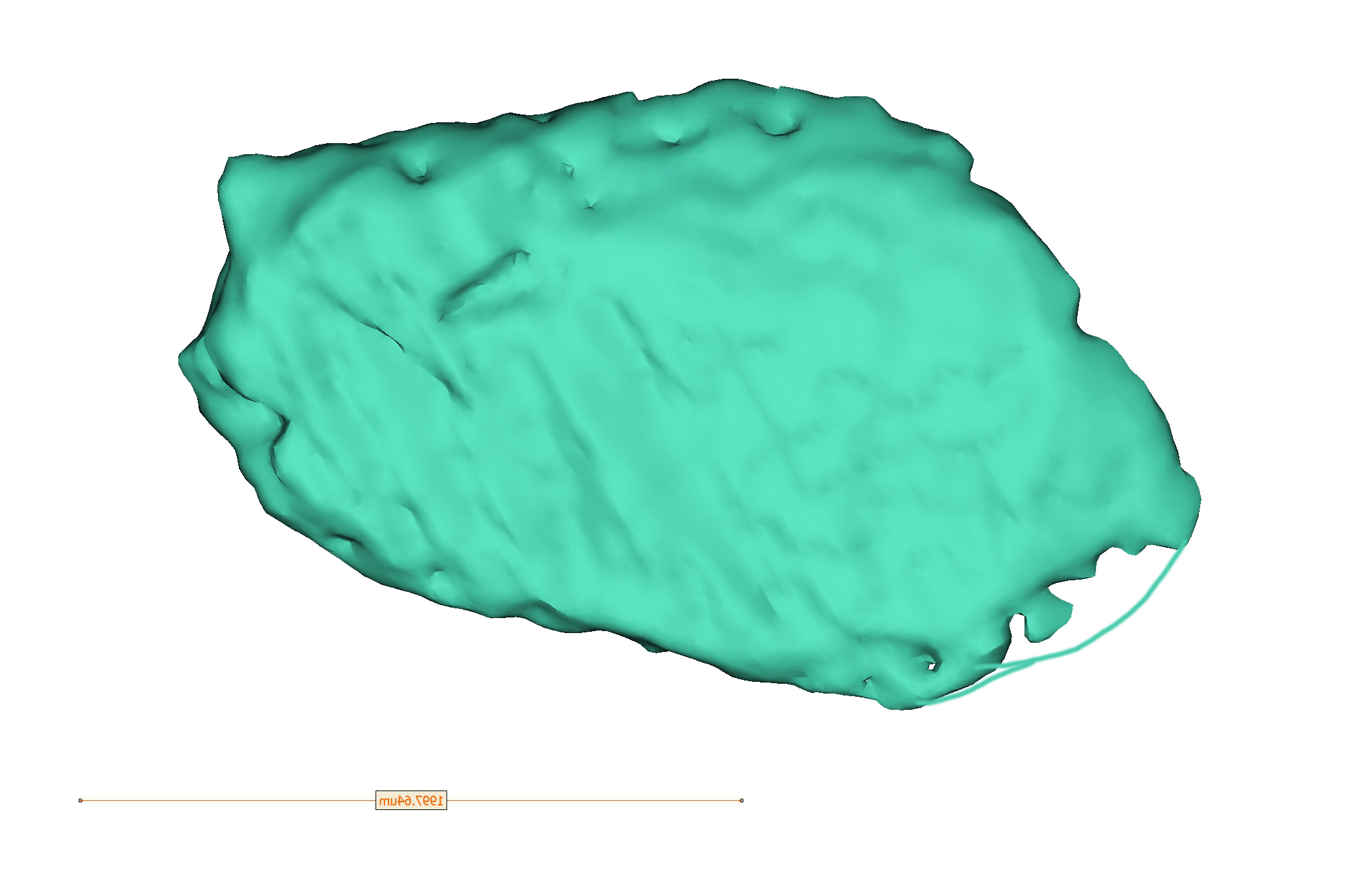

Supplement: Supplementary file 5 — Supplementary Data 2 [file 41467_2023_43557_MOESM5_ESM.zip › Supplementary Data 2/Supplementary Data 2 Raw data of Geometric Morphometric Analyses/12 Morphotypes/Morphotype 5/l1d26-.jpg]

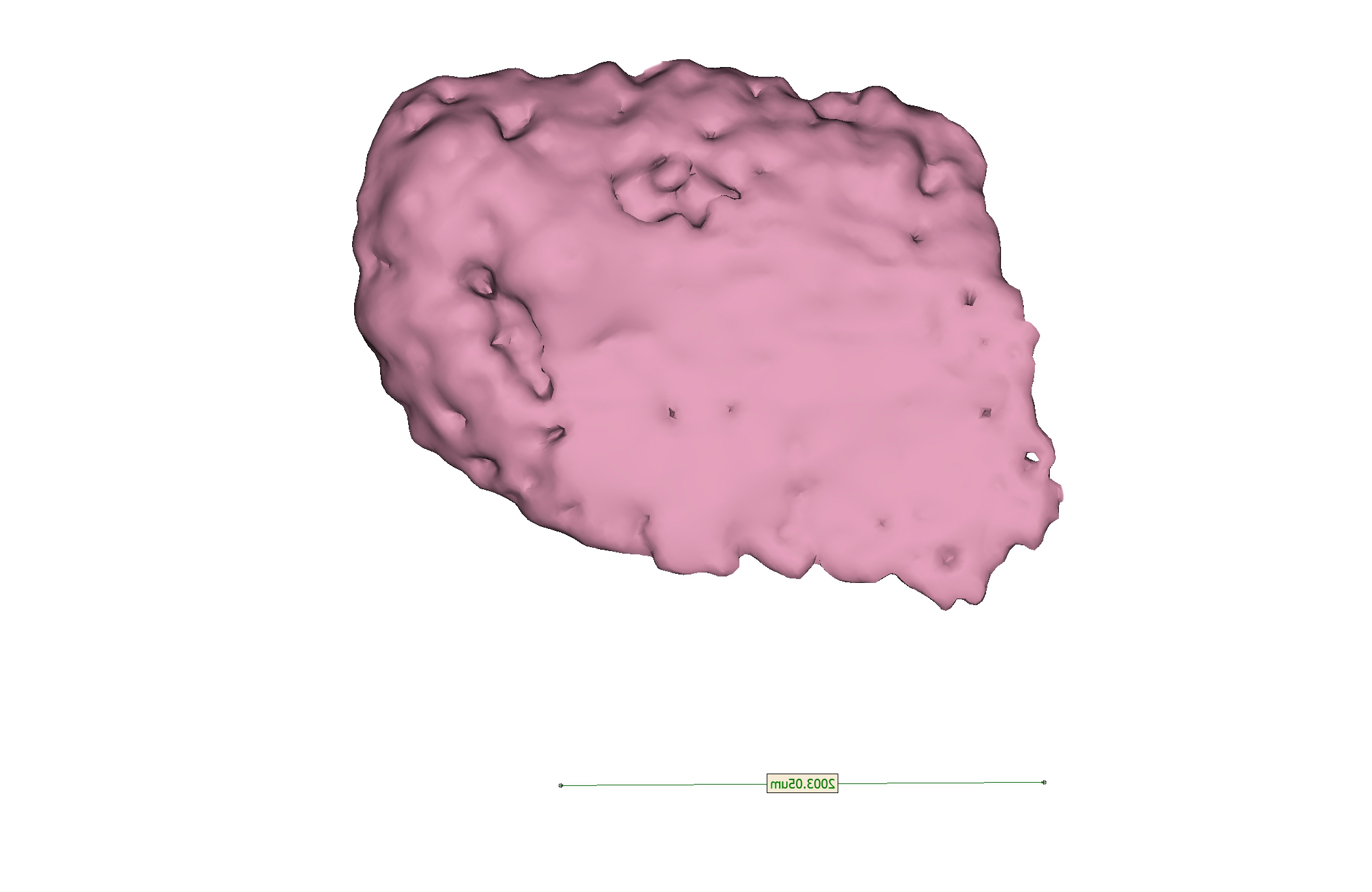

Supplement: Supplementary file 5 — Supplementary Data 2 [file 41467_2023_43557_MOESM5_ESM.zip › Supplementary Data 2/Supplementary Data 2 Raw data of Geometric Morphometric Analyses/12 Morphotypes/Morphotype 5/l1d27-.jpg]

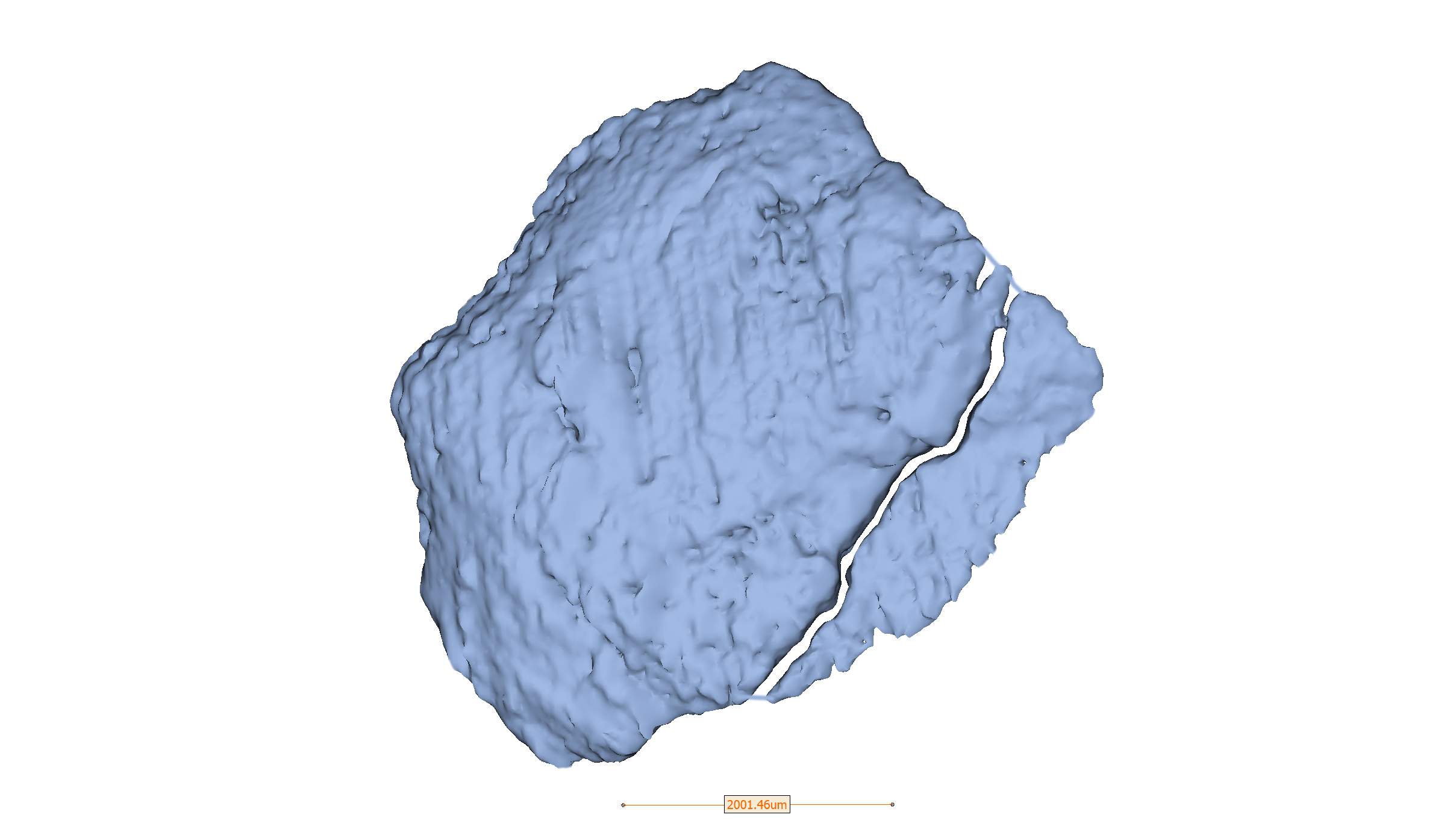

Supplement: Supplementary file 5 — Supplementary Data 2 [file 41467_2023_43557_MOESM5_ESM.zip › Supplementary Data 2/Supplementary Data 2 Raw data of Geometric Morphometric Analyses/12 Morphotypes/Morphotype 5/l1v20.jpg]

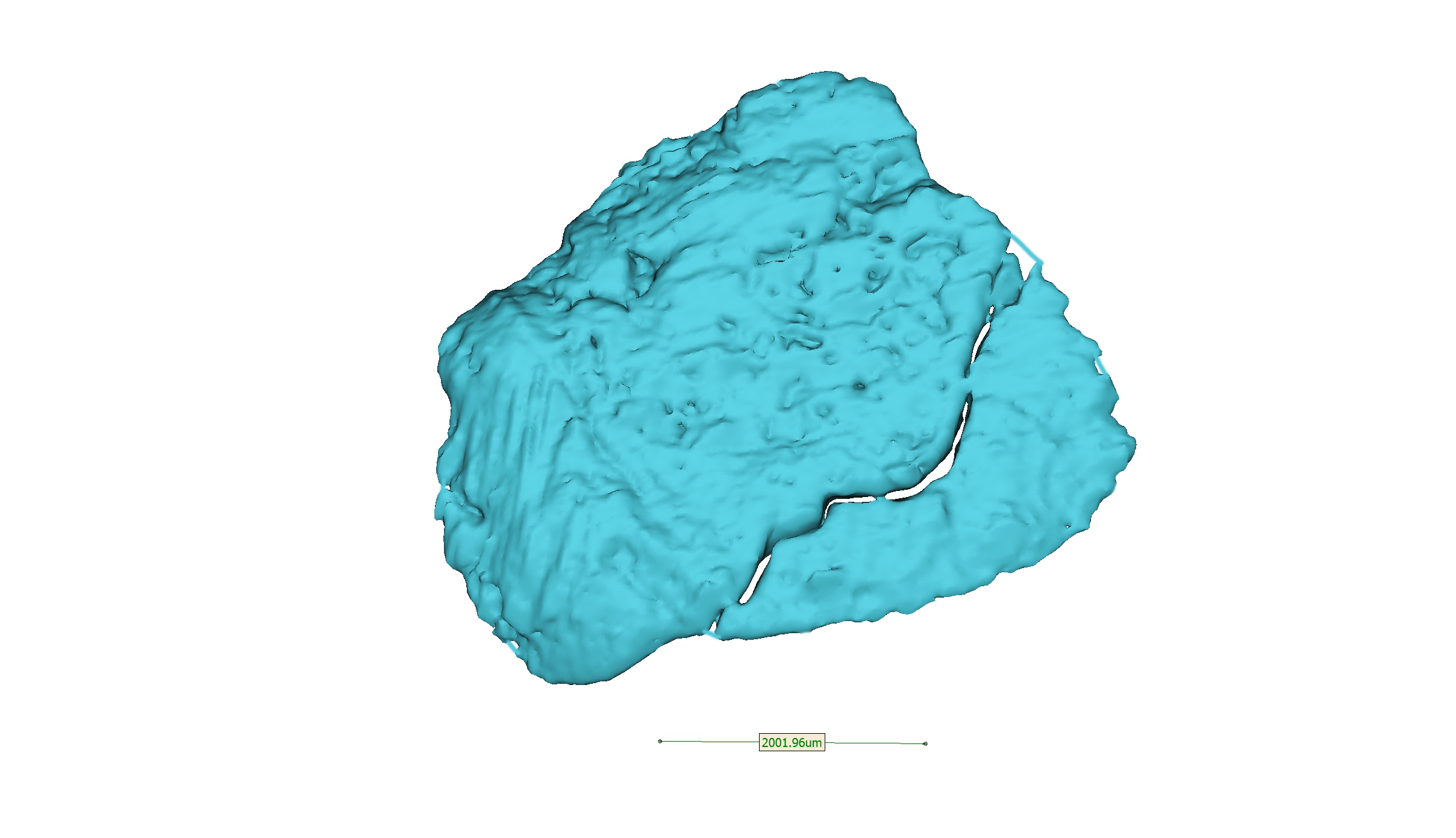

Supplement: Supplementary file 5 — Supplementary Data 2 [file 41467_2023_43557_MOESM5_ESM.zip › Supplementary Data 2/Supplementary Data 2 Raw data of Geometric Morphometric Analyses/12 Morphotypes/Morphotype 5/l1v21.jpg]

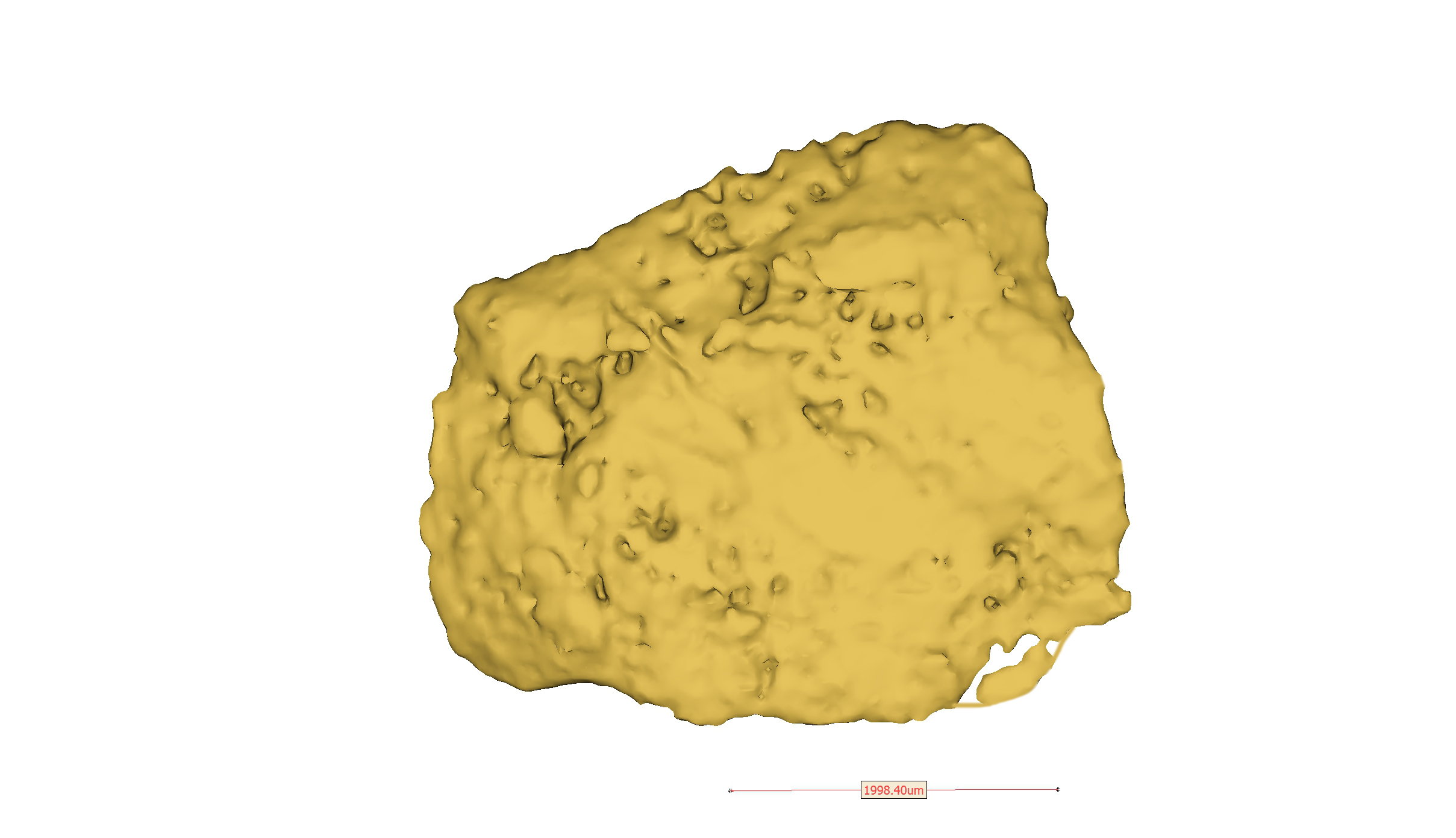

Supplement: Supplementary file 5 — Supplementary Data 2 [file 41467_2023_43557_MOESM5_ESM.zip › Supplementary Data 2/Supplementary Data 2 Raw data of Geometric Morphometric Analyses/12 Morphotypes/Morphotype 5/l1v22.jpg]

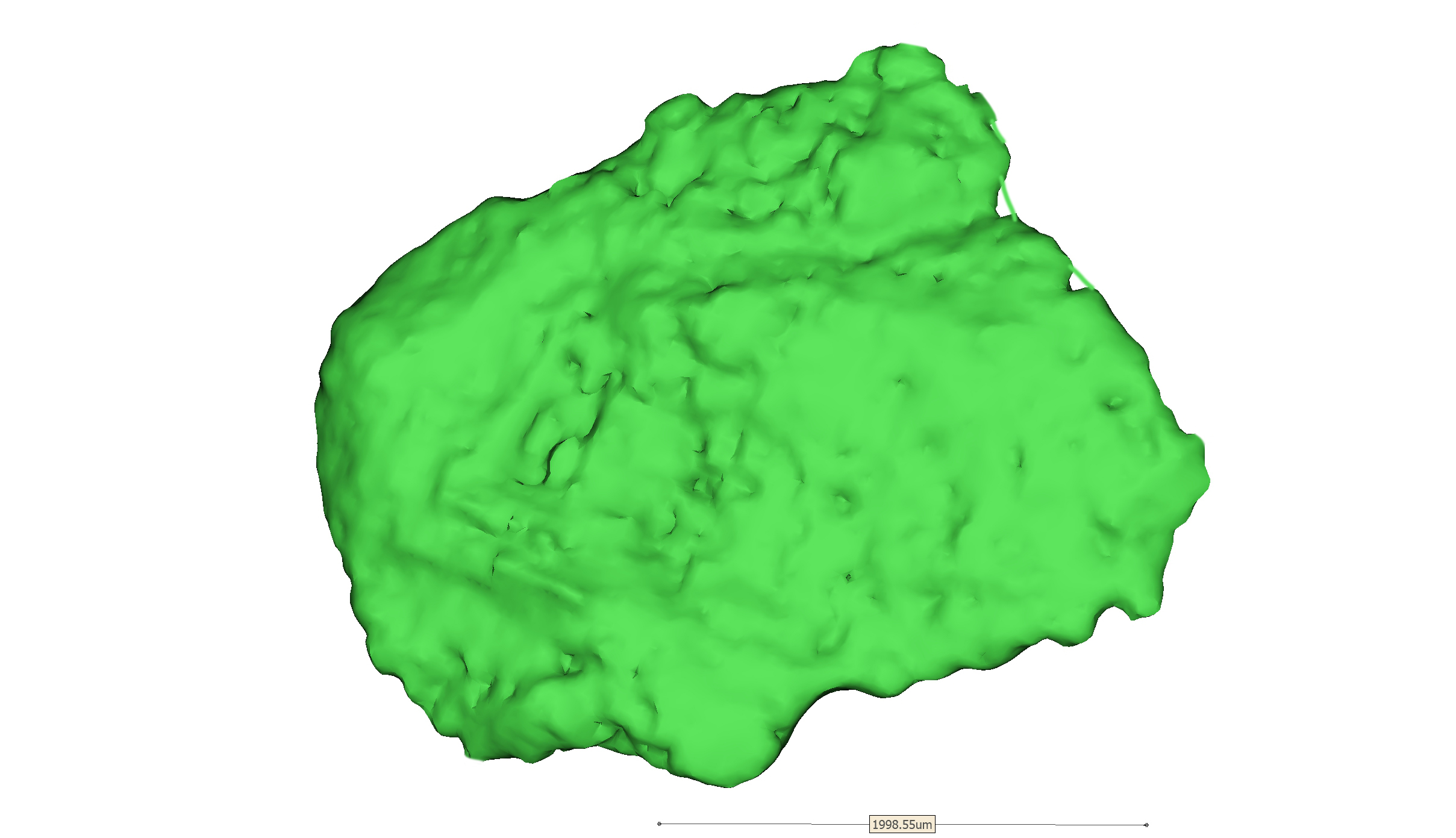

Supplement: Supplementary file 5 — Supplementary Data 2 [file 41467_2023_43557_MOESM5_ESM.zip › Supplementary Data 2/Supplementary Data 2 Raw data of Geometric Morphometric Analyses/12 Morphotypes/Morphotype 5/l1v23.jpg]

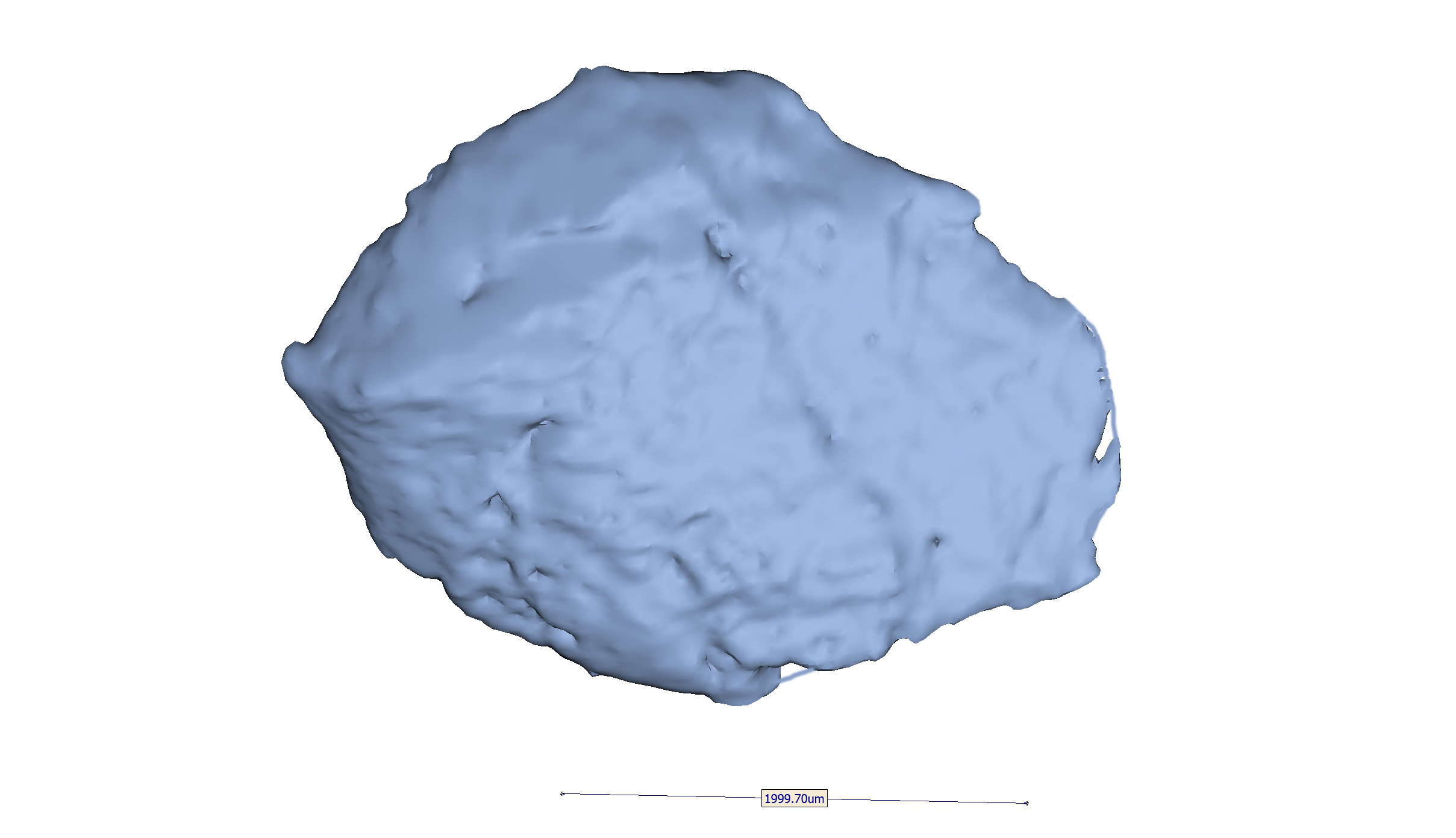

Supplement: Supplementary file 5 — Supplementary Data 2 [file 41467_2023_43557_MOESM5_ESM.zip › Supplementary Data 2/Supplementary Data 2 Raw data of Geometric Morphometric Analyses/12 Morphotypes/Morphotype 5/l1v24.jpg]

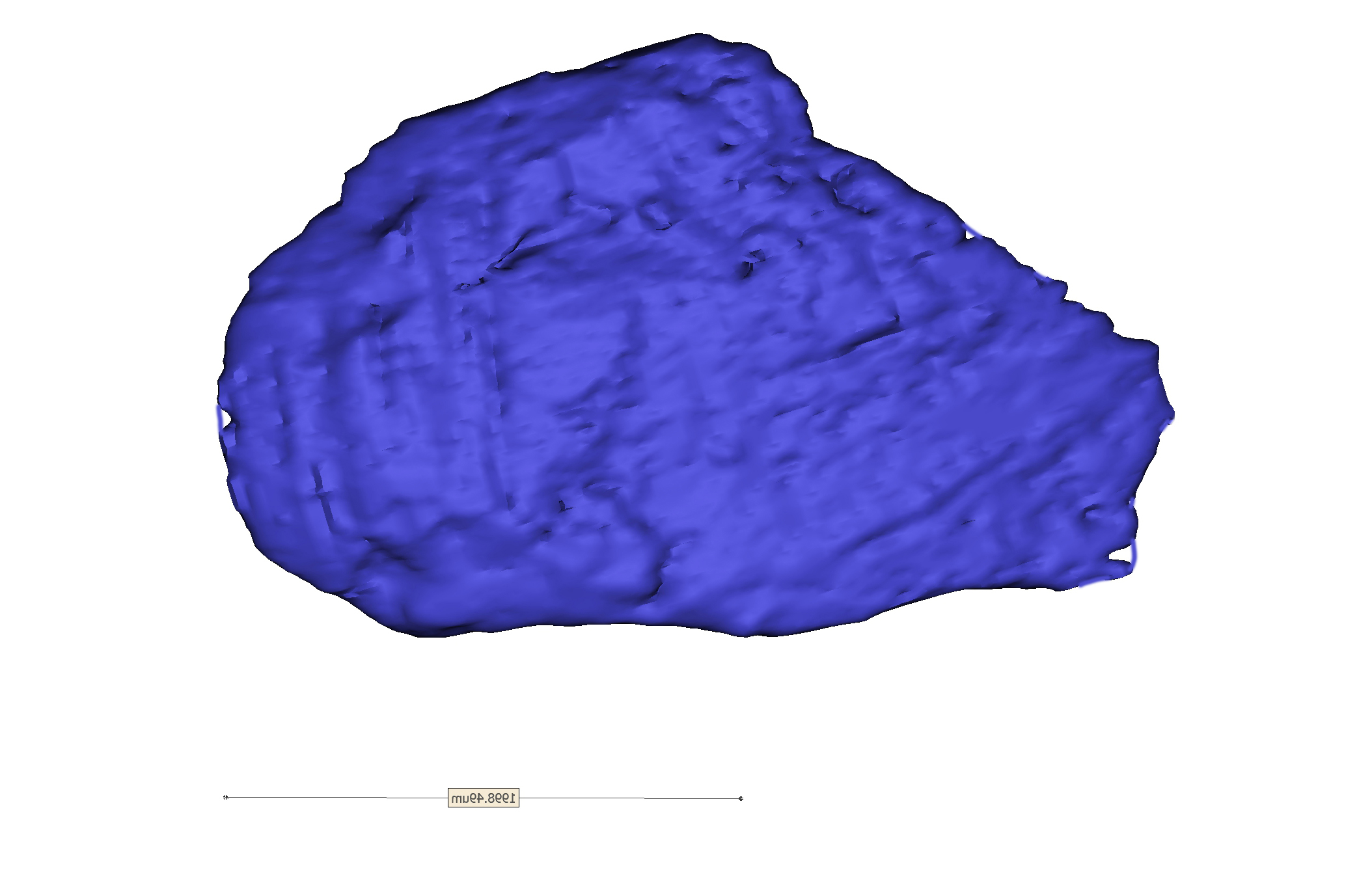

Supplement: Supplementary file 5 — Supplementary Data 2 [file 41467_2023_43557_MOESM5_ESM.zip › Supplementary Data 2/Supplementary Data 2 Raw data of Geometric Morphometric Analyses/12 Morphotypes/Morphotype 5/l2d22-.jpg]

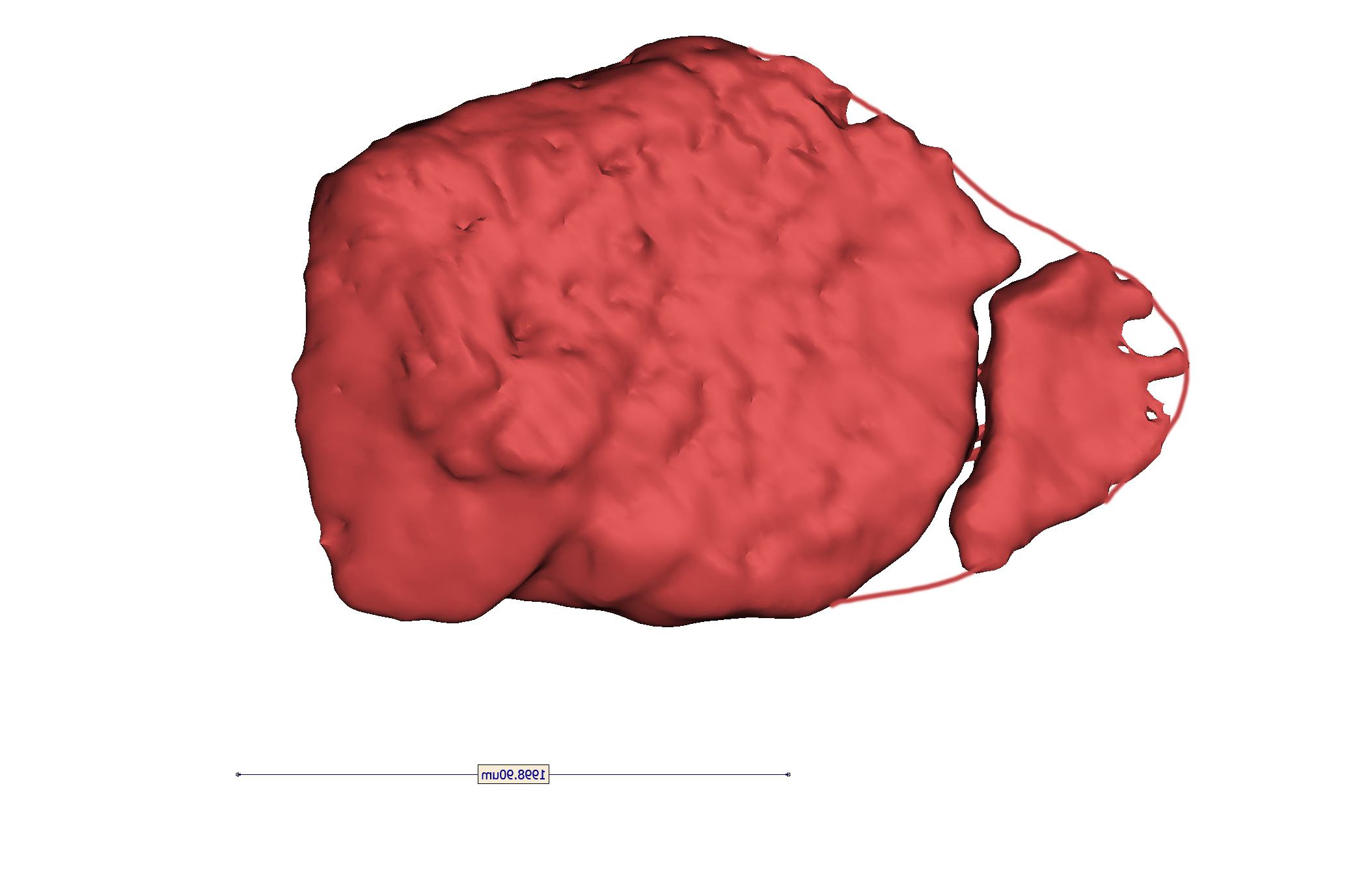

Supplement: Supplementary file 5 — Supplementary Data 2 [file 41467_2023_43557_MOESM5_ESM.zip › Supplementary Data 2/Supplementary Data 2 Raw data of Geometric Morphometric Analyses/12 Morphotypes/Morphotype 5/l2d23-.jpg]

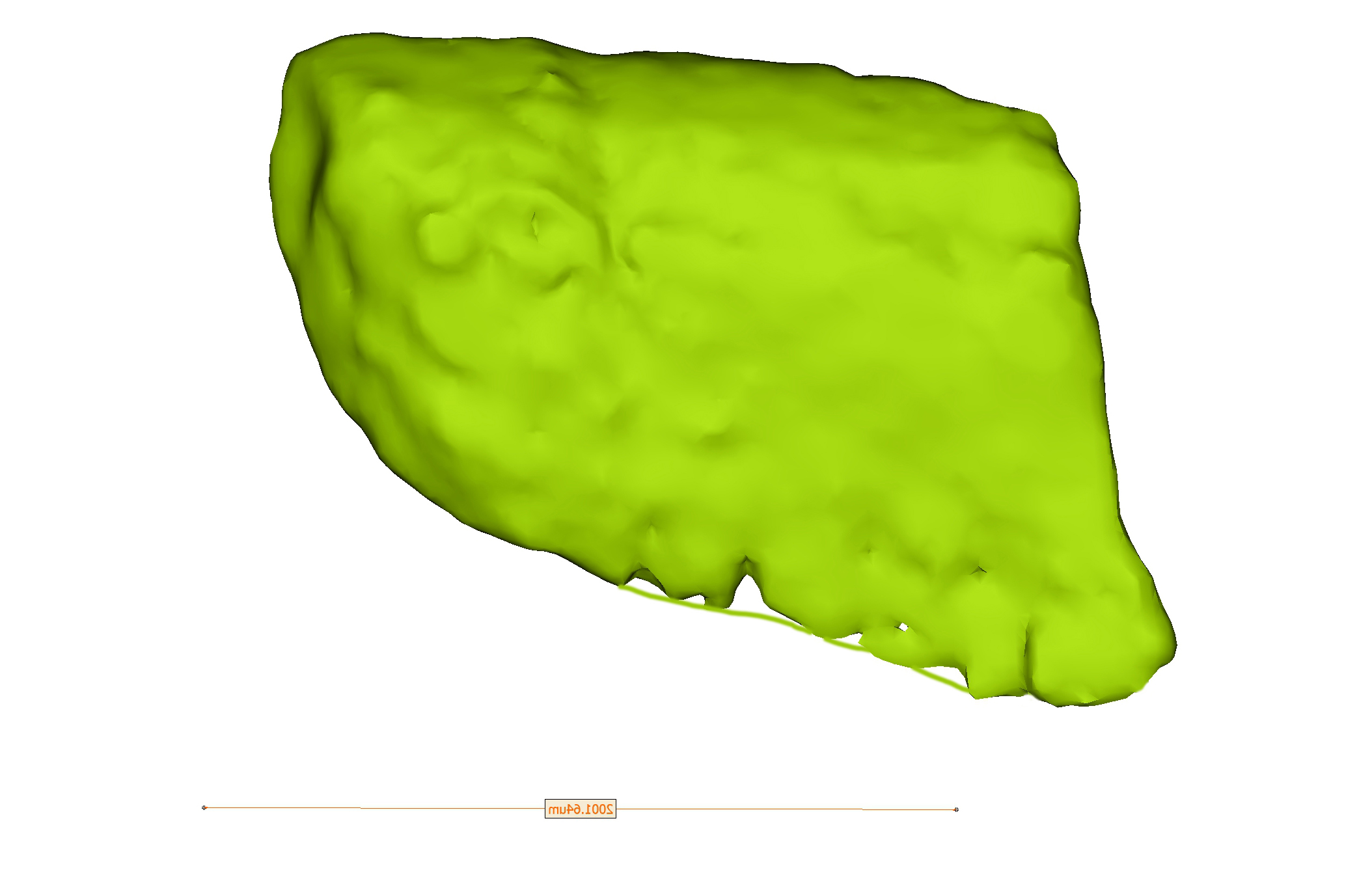

Supplement: Supplementary file 5 — Supplementary Data 2 [file 41467_2023_43557_MOESM5_ESM.zip › Supplementary Data 2/Supplementary Data 2 Raw data of Geometric Morphometric Analyses/12 Morphotypes/Morphotype 5/l2d24-.jpg]

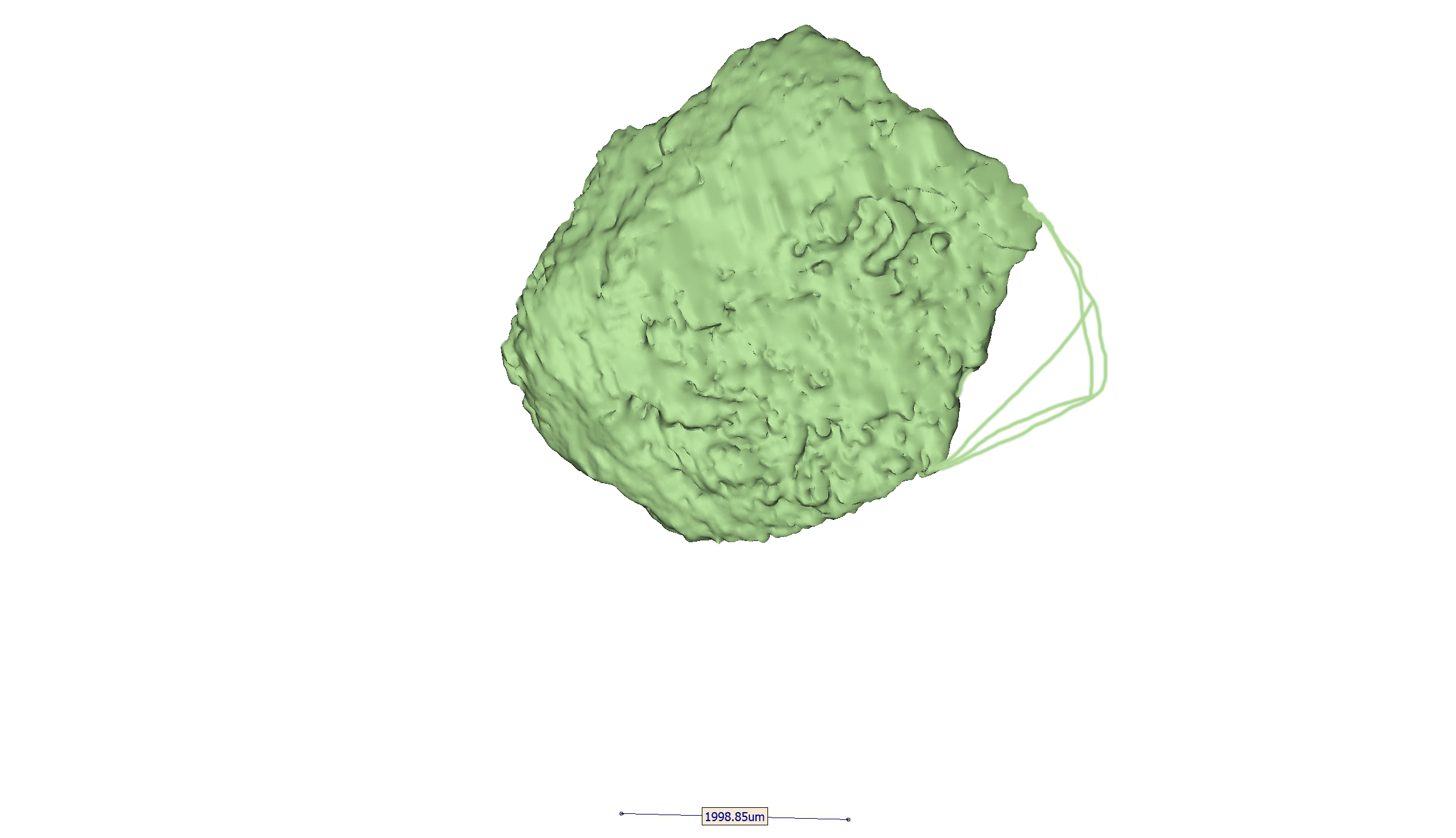

Supplement: Supplementary file 5 — Supplementary Data 2 [file 41467_2023_43557_MOESM5_ESM.zip › Supplementary Data 2/Supplementary Data 2 Raw data of Geometric Morphometric Analyses/12 Morphotypes/Morphotype 5/l2v19.jpg]

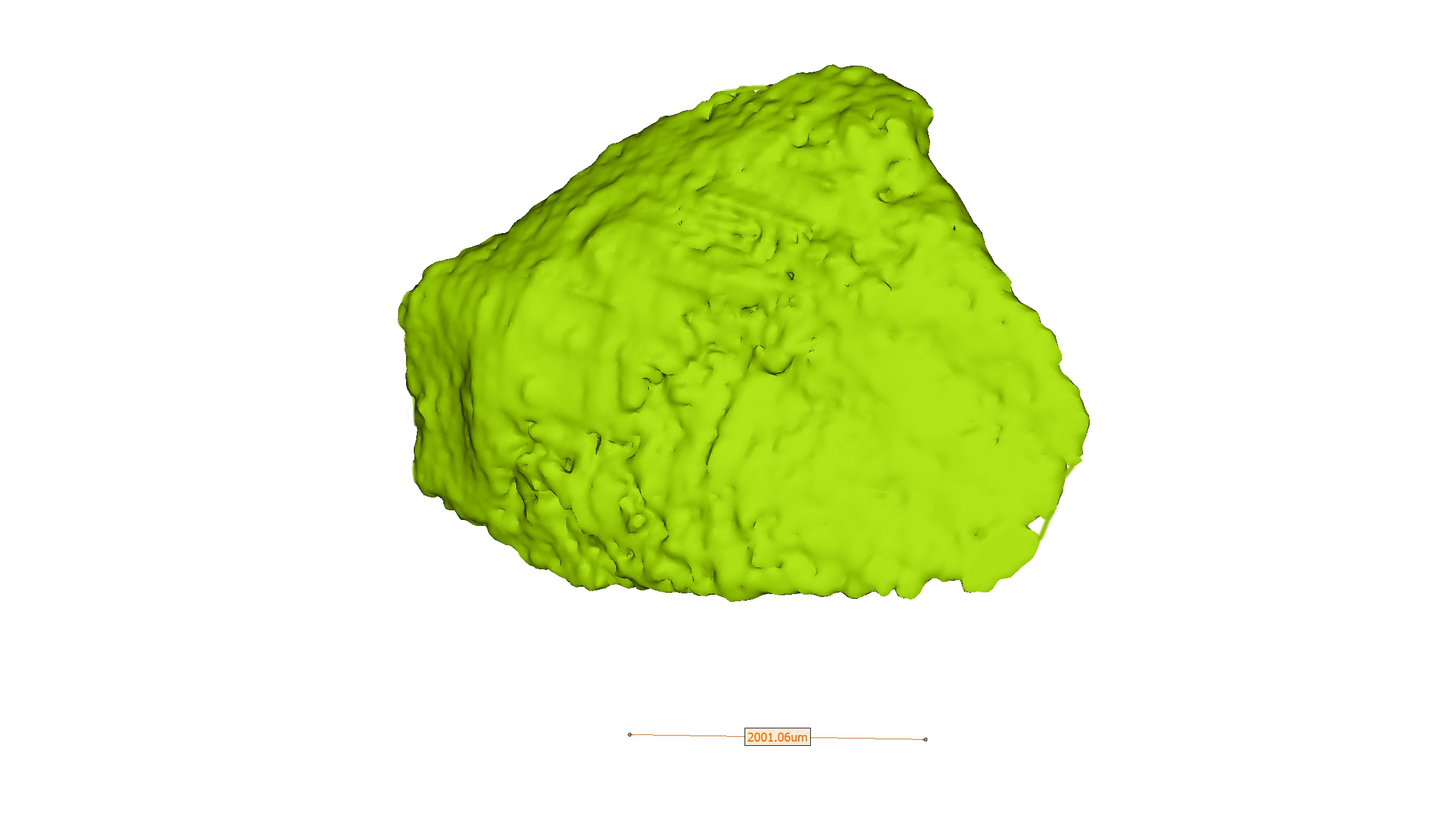

Supplement: Supplementary file 5 — Supplementary Data 2 [file 41467_2023_43557_MOESM5_ESM.zip › Supplementary Data 2/Supplementary Data 2 Raw data of Geometric Morphometric Analyses/12 Morphotypes/Morphotype 5/l2v20.jpg]

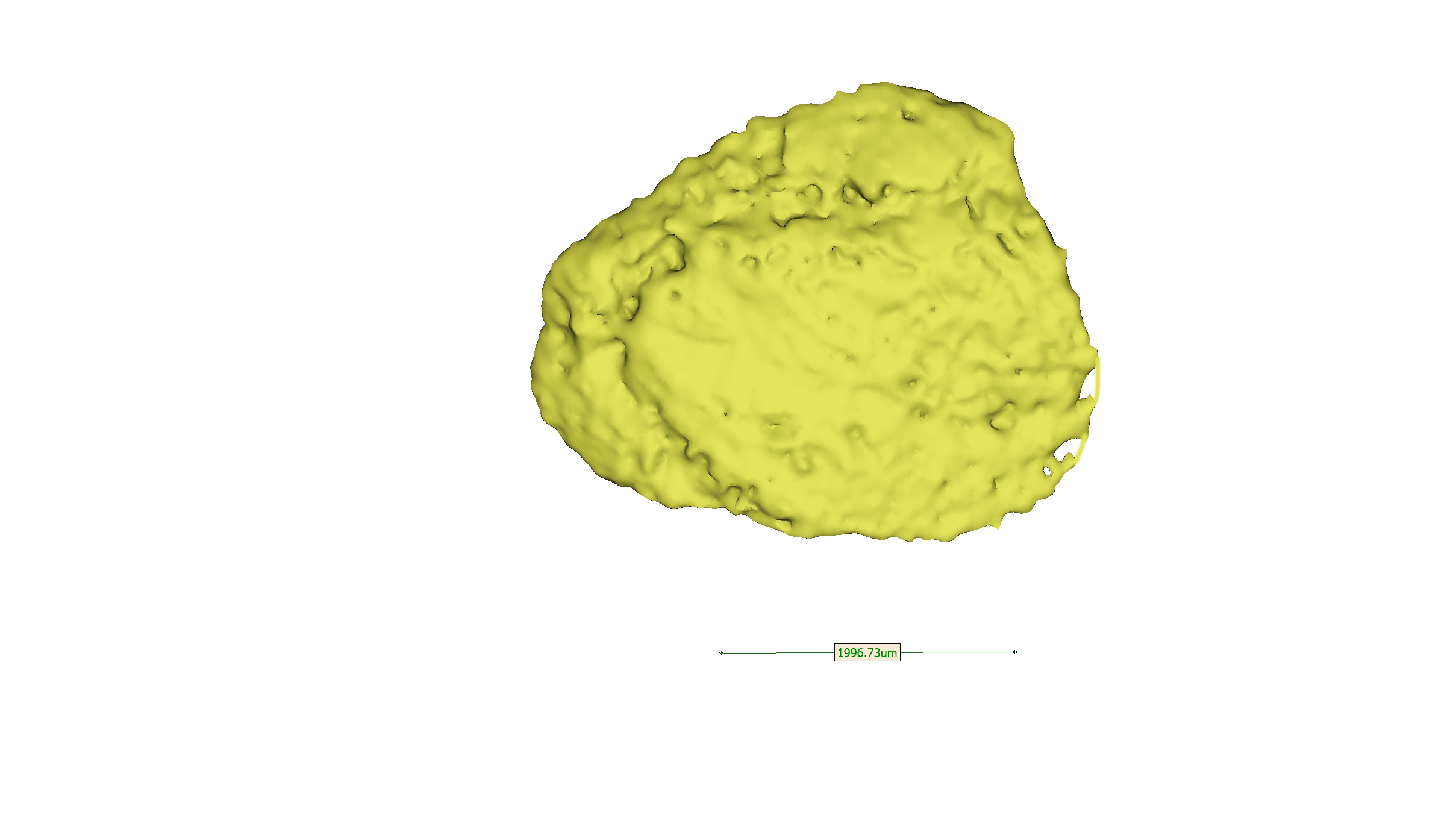

Supplement: Supplementary file 5 — Supplementary Data 2 [file 41467_2023_43557_MOESM5_ESM.zip › Supplementary Data 2/Supplementary Data 2 Raw data of Geometric Morphometric Analyses/12 Morphotypes/Morphotype 5/l2v21.jpg]

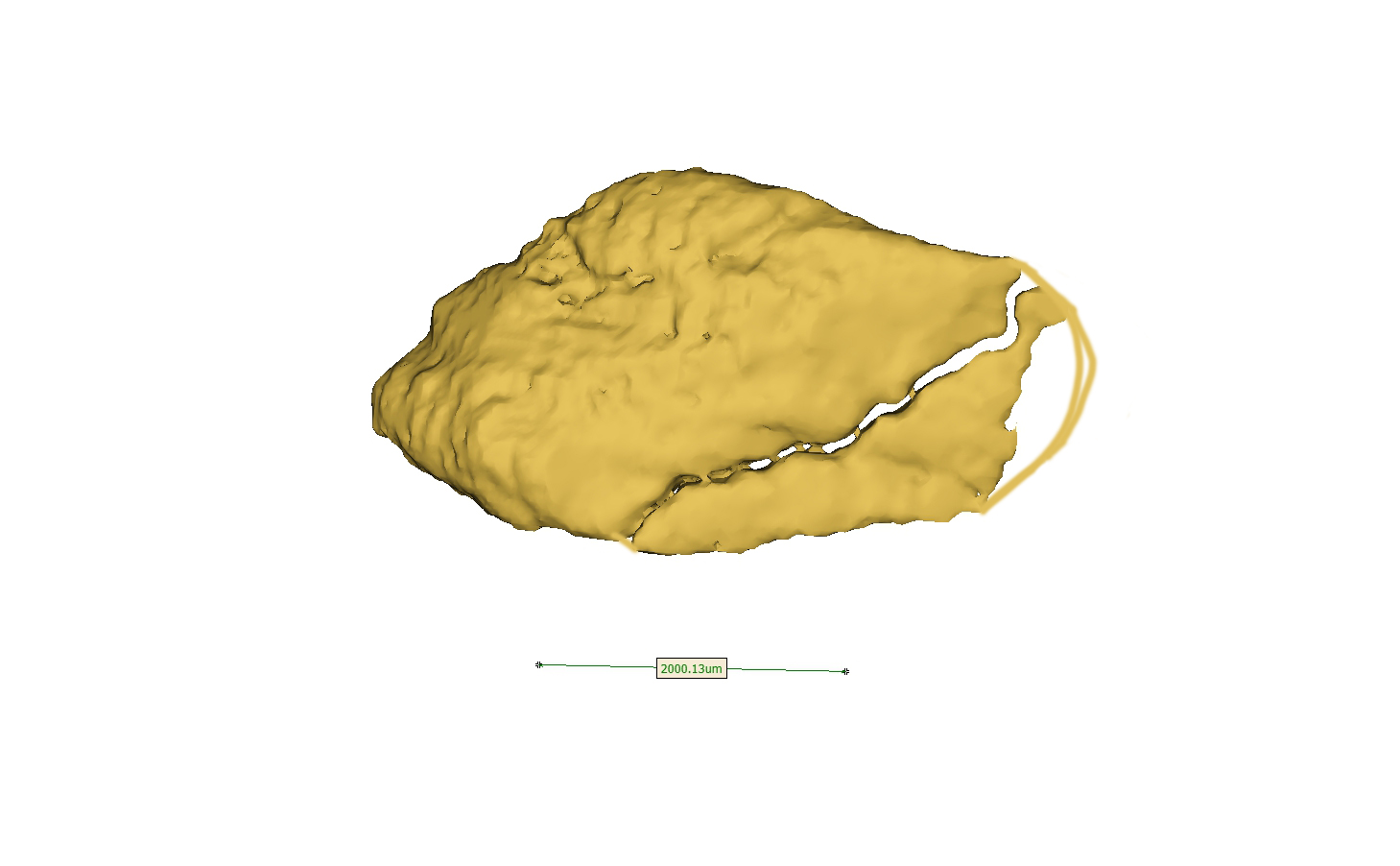

Supplement: Supplementary file 5 — Supplementary Data 2 [file 41467_2023_43557_MOESM5_ESM.zip › Supplementary Data 2/Supplementary Data 2 Raw data of Geometric Morphometric Analyses/12 Morphotypes/Morphotype 5/l2v23.jpg]

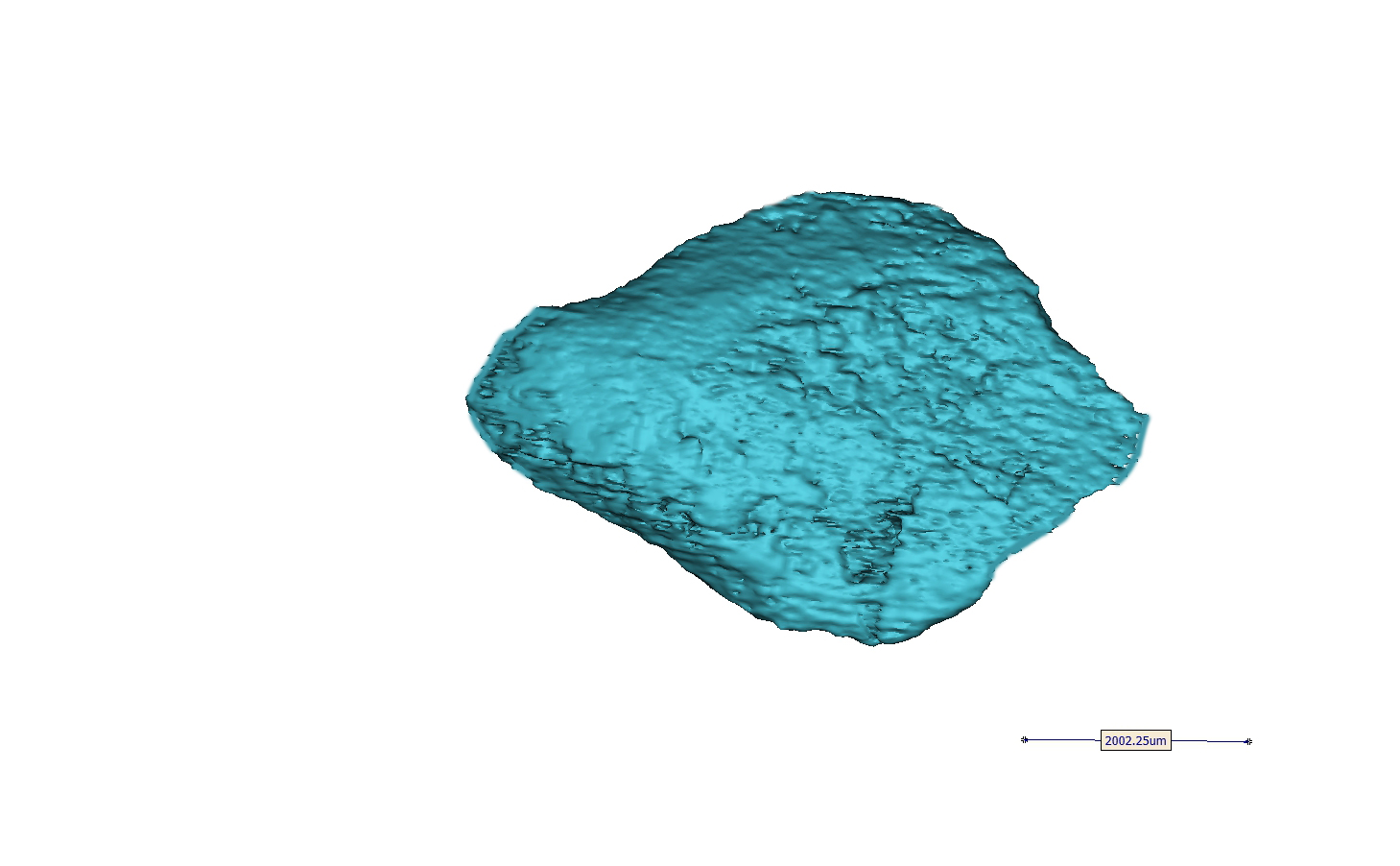

Supplement: Supplementary file 5 — Supplementary Data 2 [file 41467_2023_43557_MOESM5_ESM.zip › Supplementary Data 2/Supplementary Data 2 Raw data of Geometric Morphometric Analyses/12 Morphotypes/Morphotype 5/l3v18.jpg]

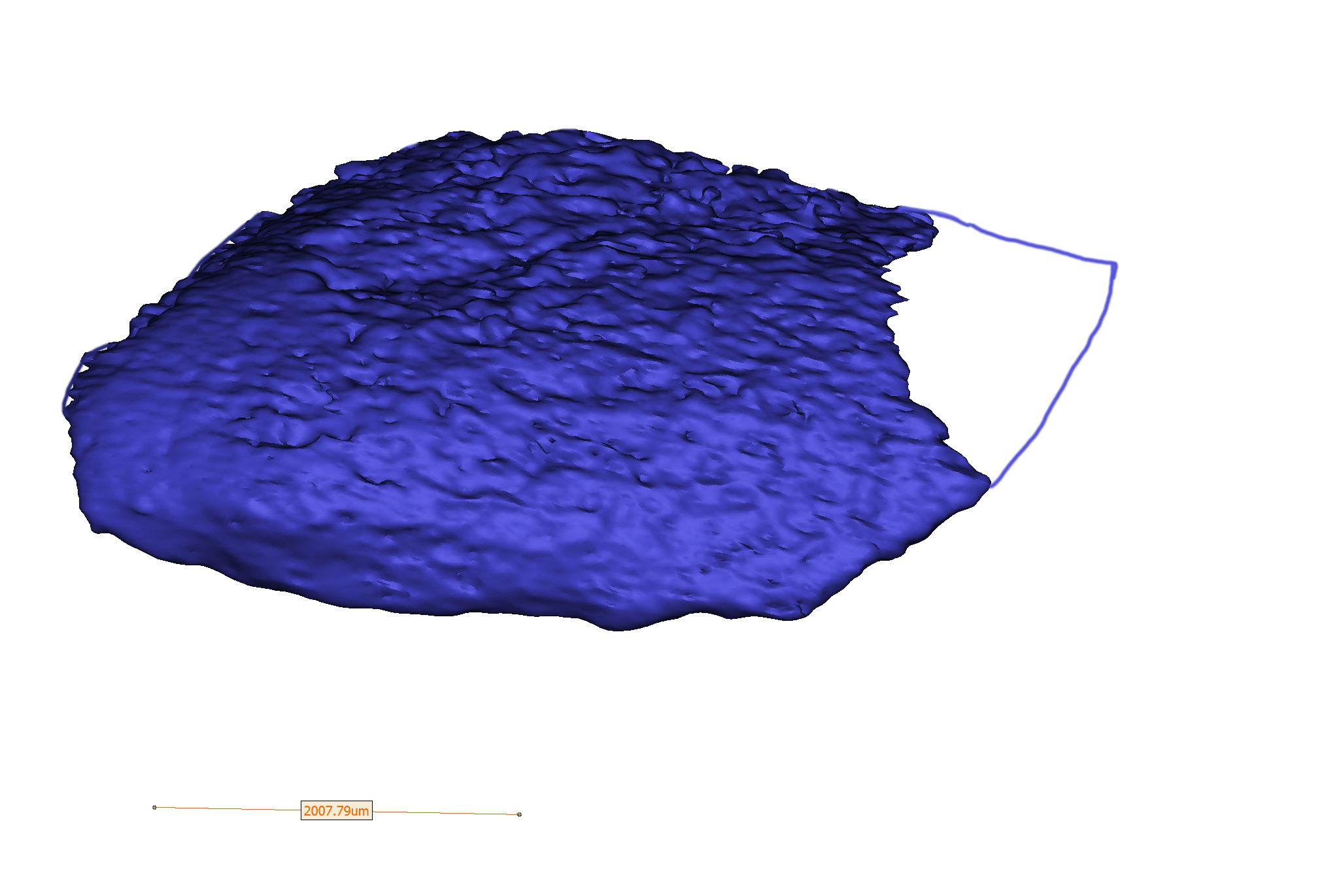

Supplement: Supplementary file 5 — Supplementary Data 2 [file 41467_2023_43557_MOESM5_ESM.zip › Supplementary Data 2/Supplementary Data 2 Raw data of Geometric Morphometric Analyses/12 Morphotypes/Morphotype 5/l3v19.jpg]

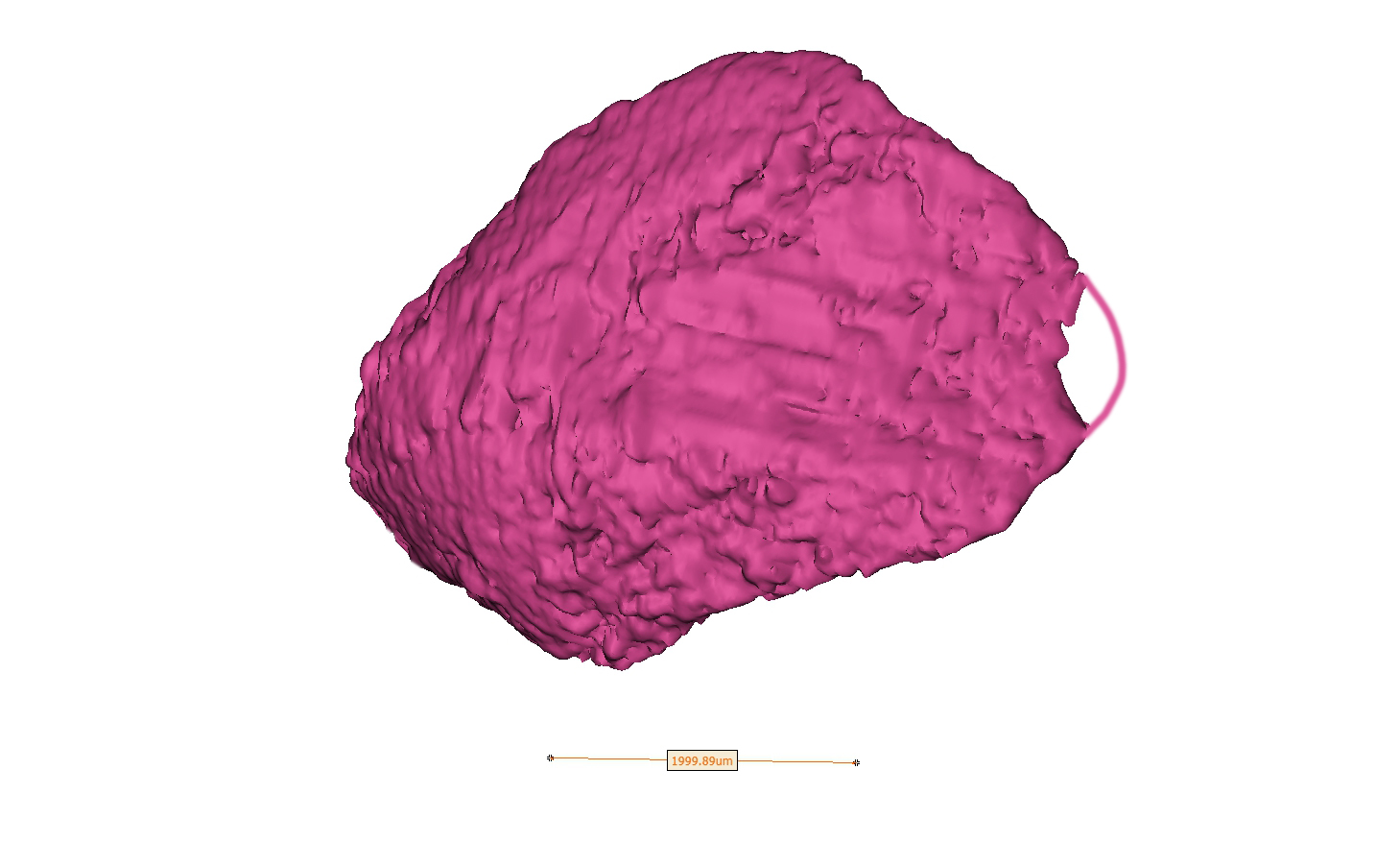

Supplement: Supplementary file 5 — Supplementary Data 2 [file 41467_2023_43557_MOESM5_ESM.zip › Supplementary Data 2/Supplementary Data 2 Raw data of Geometric Morphometric Analyses/12 Morphotypes/Morphotype 5/l3v20.jpg]

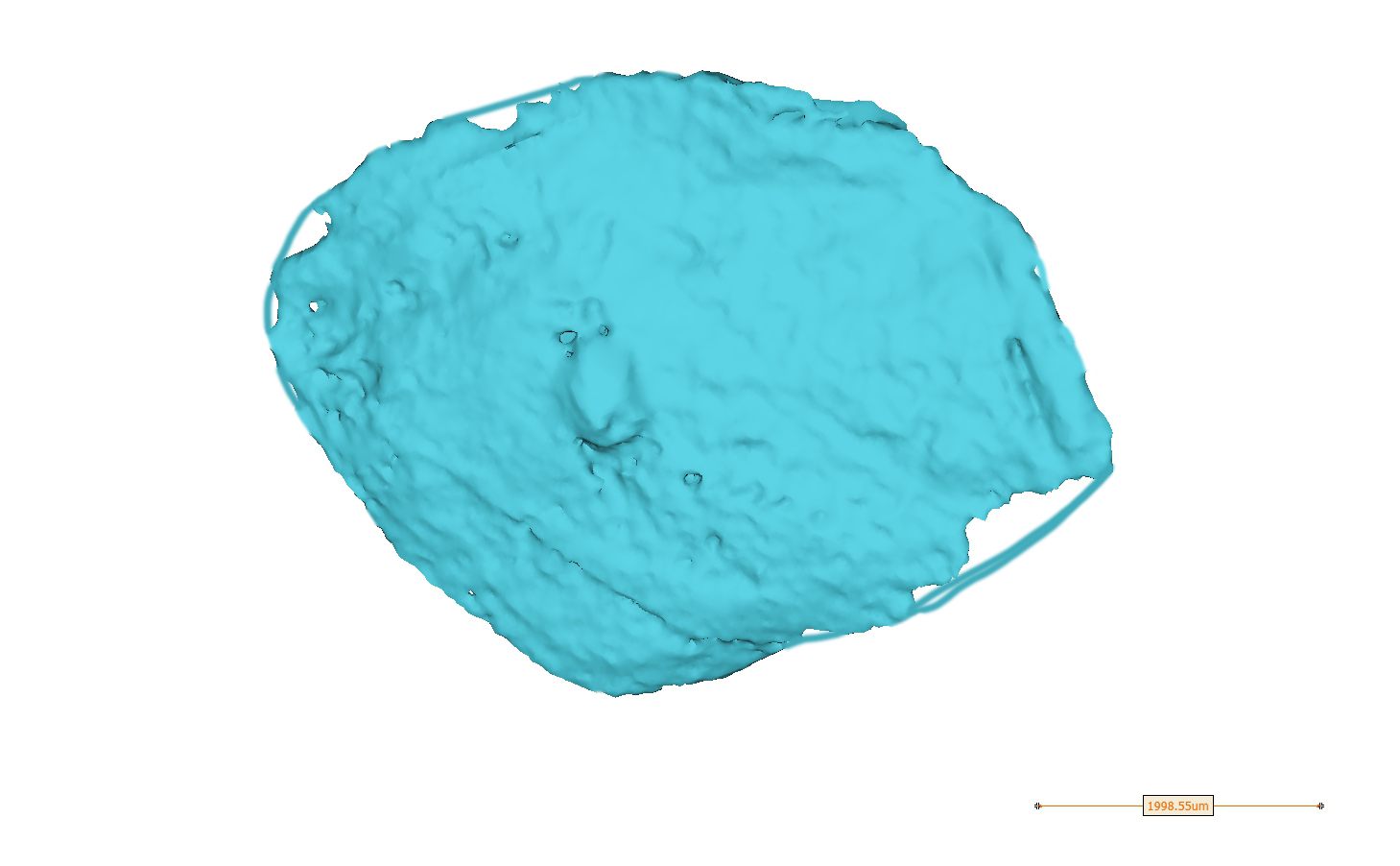

Supplement: Supplementary file 5 — Supplementary Data 2 [file 41467_2023_43557_MOESM5_ESM.zip › Supplementary Data 2/Supplementary Data 2 Raw data of Geometric Morphometric Analyses/12 Morphotypes/Morphotype 5/l5v08.jpg]

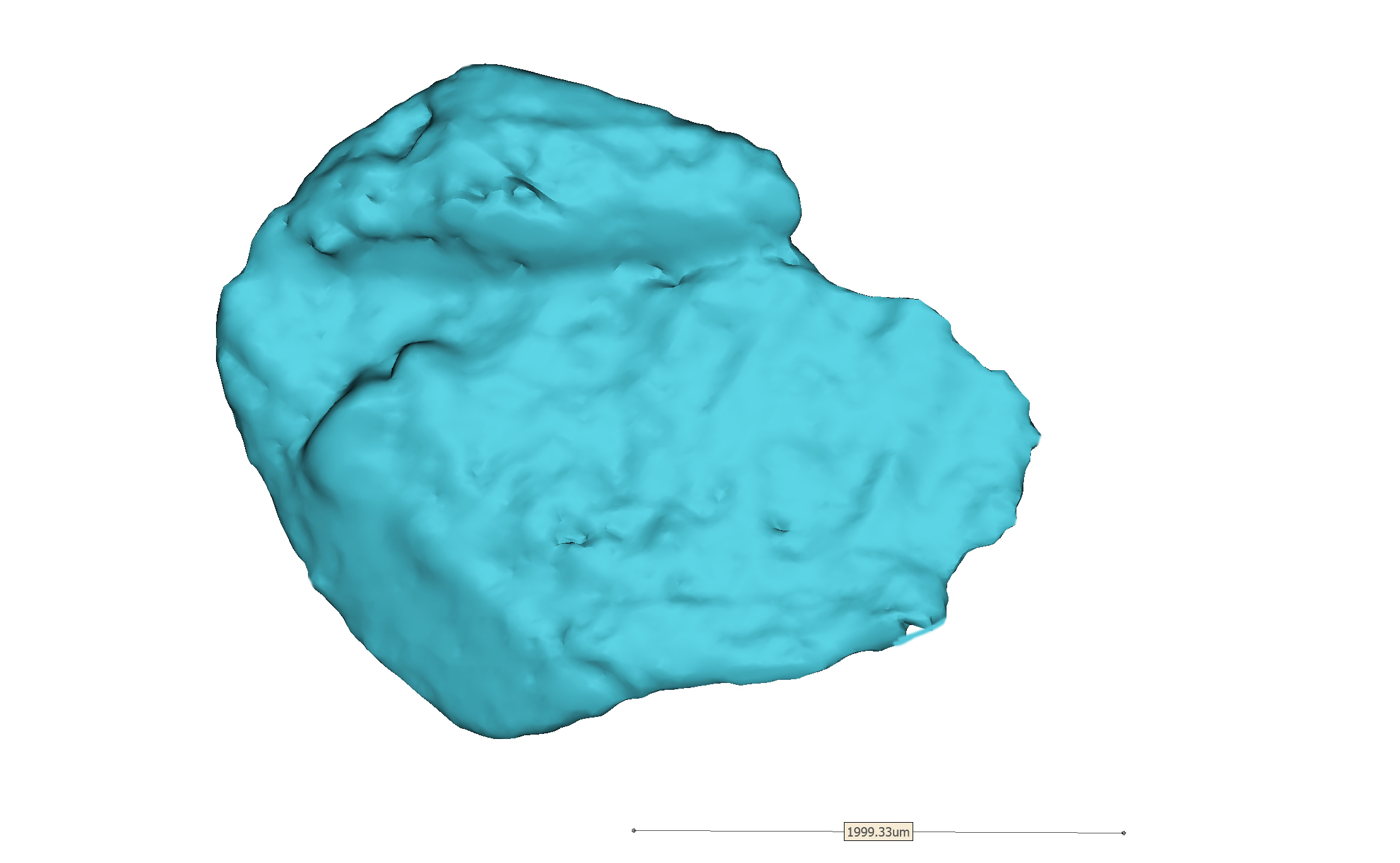

Supplement: Supplementary file 5 — Supplementary Data 2 [file 41467_2023_43557_MOESM5_ESM.zip › Supplementary Data 2/Supplementary Data 2 Raw data of Geometric Morphometric Analyses/12 Morphotypes/Morphotype 5/ll24-.jpg]

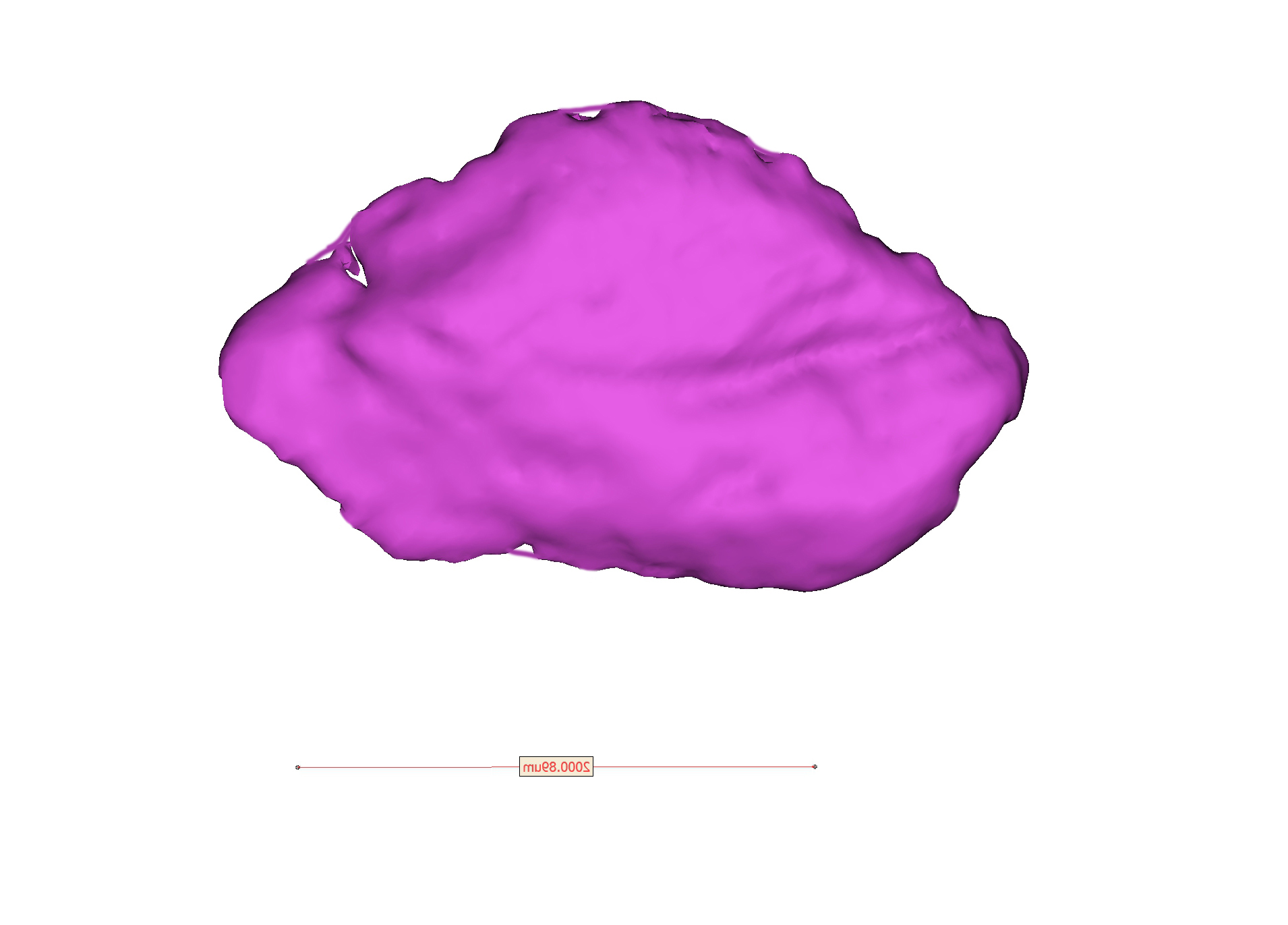

Supplement: Supplementary file 5 — Supplementary Data 2 [file 41467_2023_43557_MOESM5_ESM.zip › Supplementary Data 2/Supplementary Data 2 Raw data of Geometric Morphometric Analyses/12 Morphotypes/Morphotype 5/ll27-.jpg]

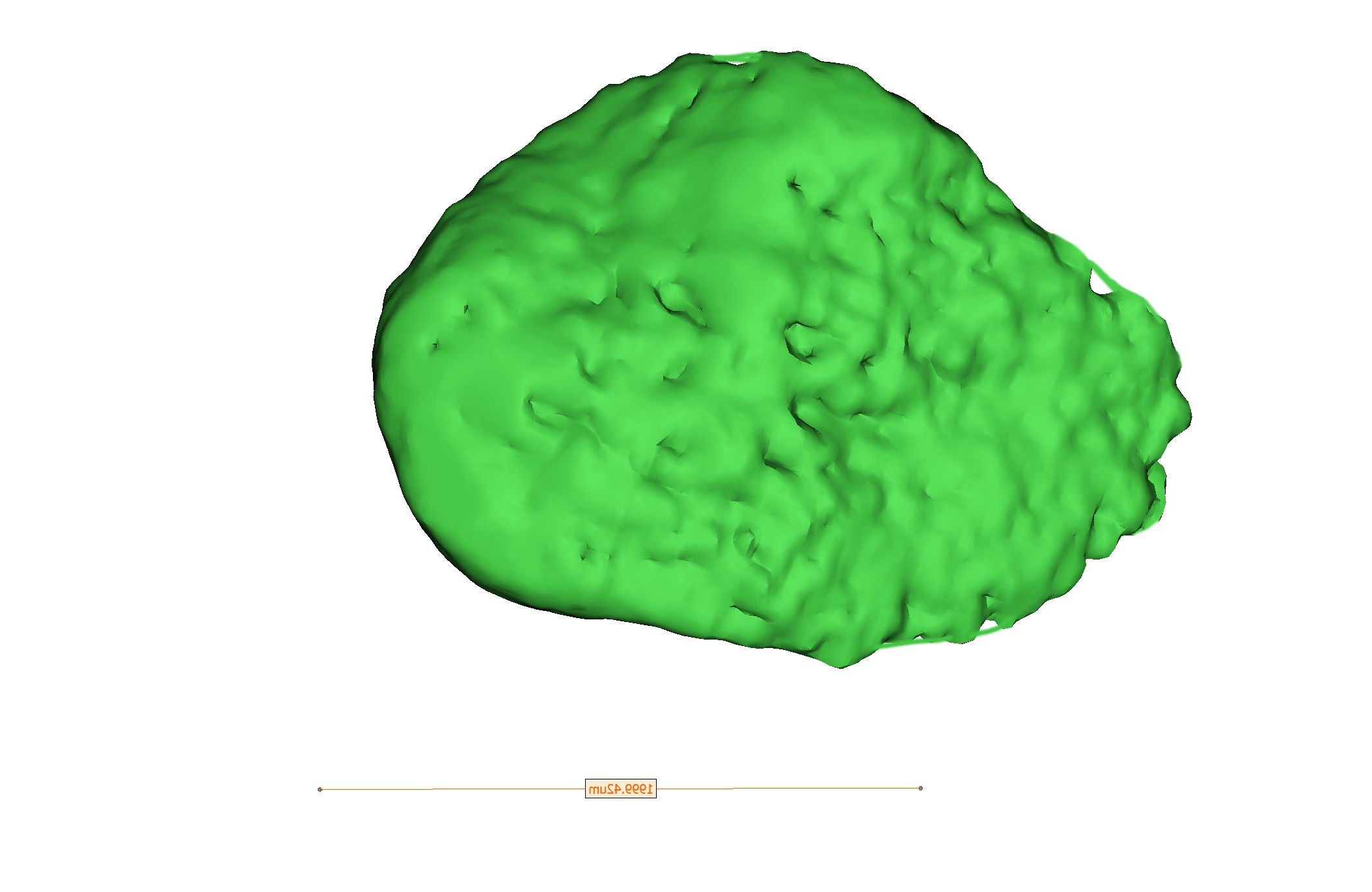

Supplement: Supplementary file 5 — Supplementary Data 2 [file 41467_2023_43557_MOESM5_ESM.zip › Supplementary Data 2/Supplementary Data 2 Raw data of Geometric Morphometric Analyses/12 Morphotypes/Morphotype 5/t02r.jpg]

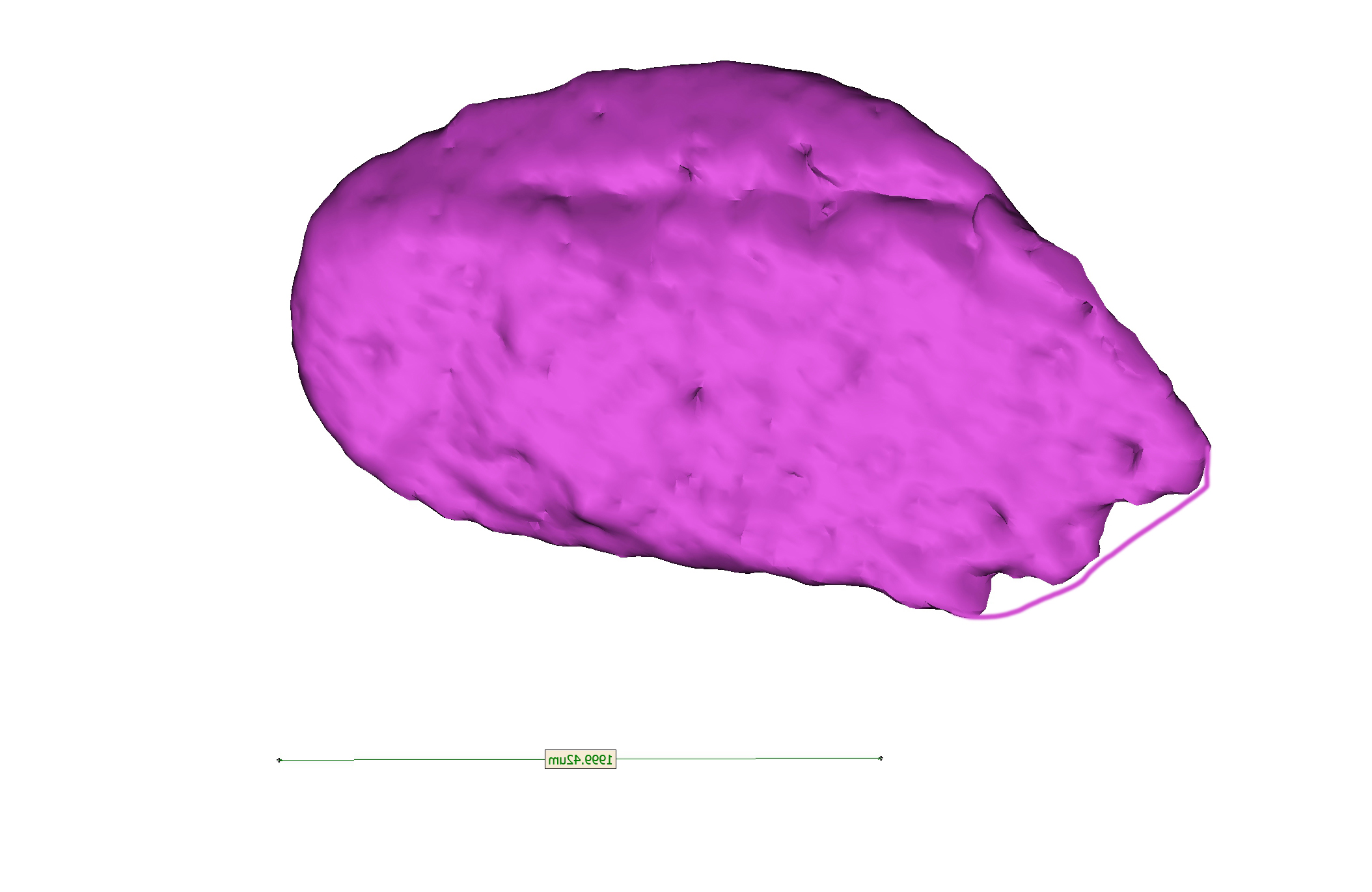

Supplement: Supplementary file 5 — Supplementary Data 2 [file 41467_2023_43557_MOESM5_ESM.zip › Supplementary Data 2/Supplementary Data 2 Raw data of Geometric Morphometric Analyses/12 Morphotypes/Morphotype 5/t03r.jpg]

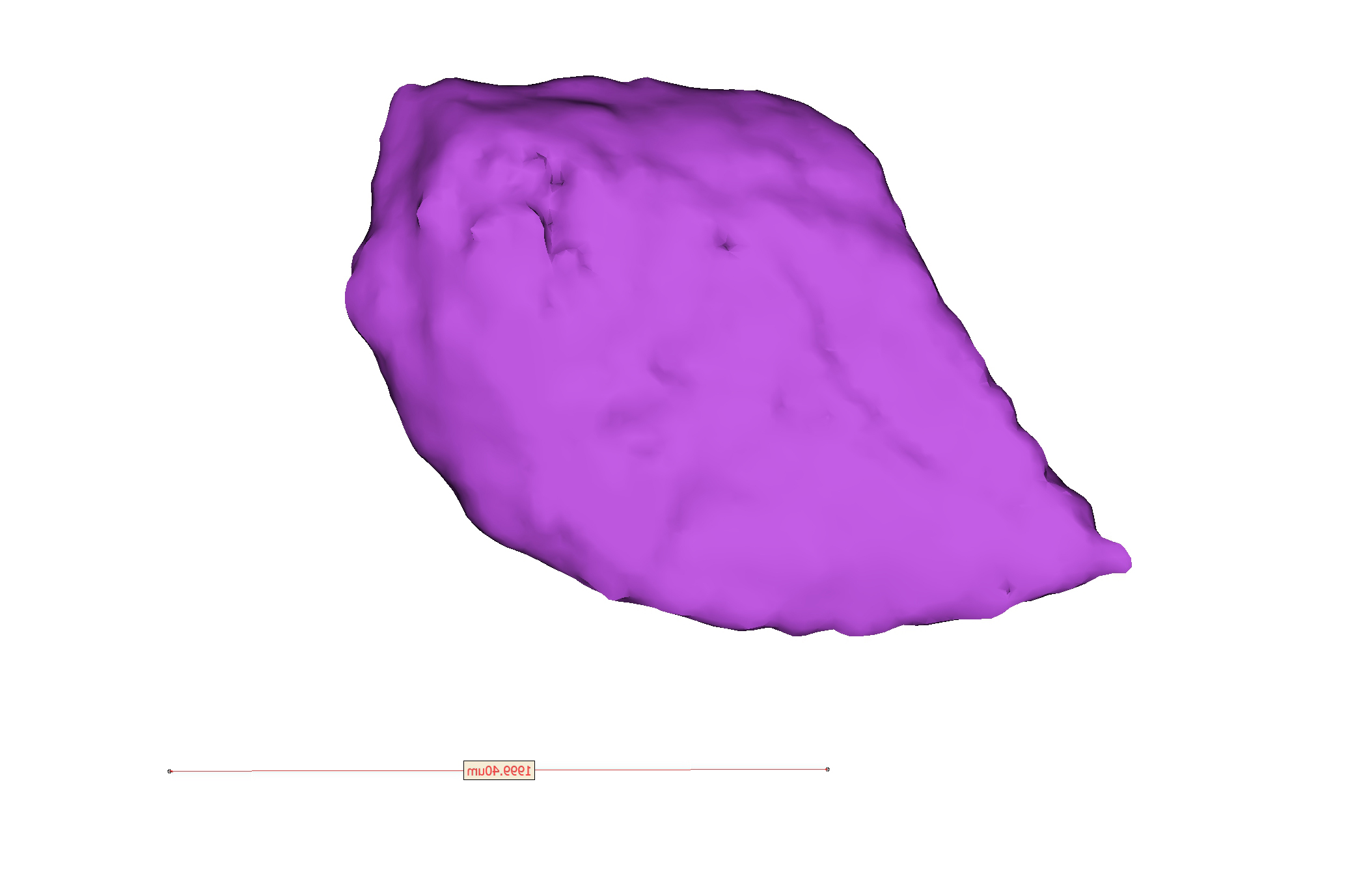

Supplement: Supplementary file 5 — Supplementary Data 2 [file 41467_2023_43557_MOESM5_ESM.zip › Supplementary Data 2/Supplementary Data 2 Raw data of Geometric Morphometric Analyses/12 Morphotypes/Morphotype 5/t04r.jpg]

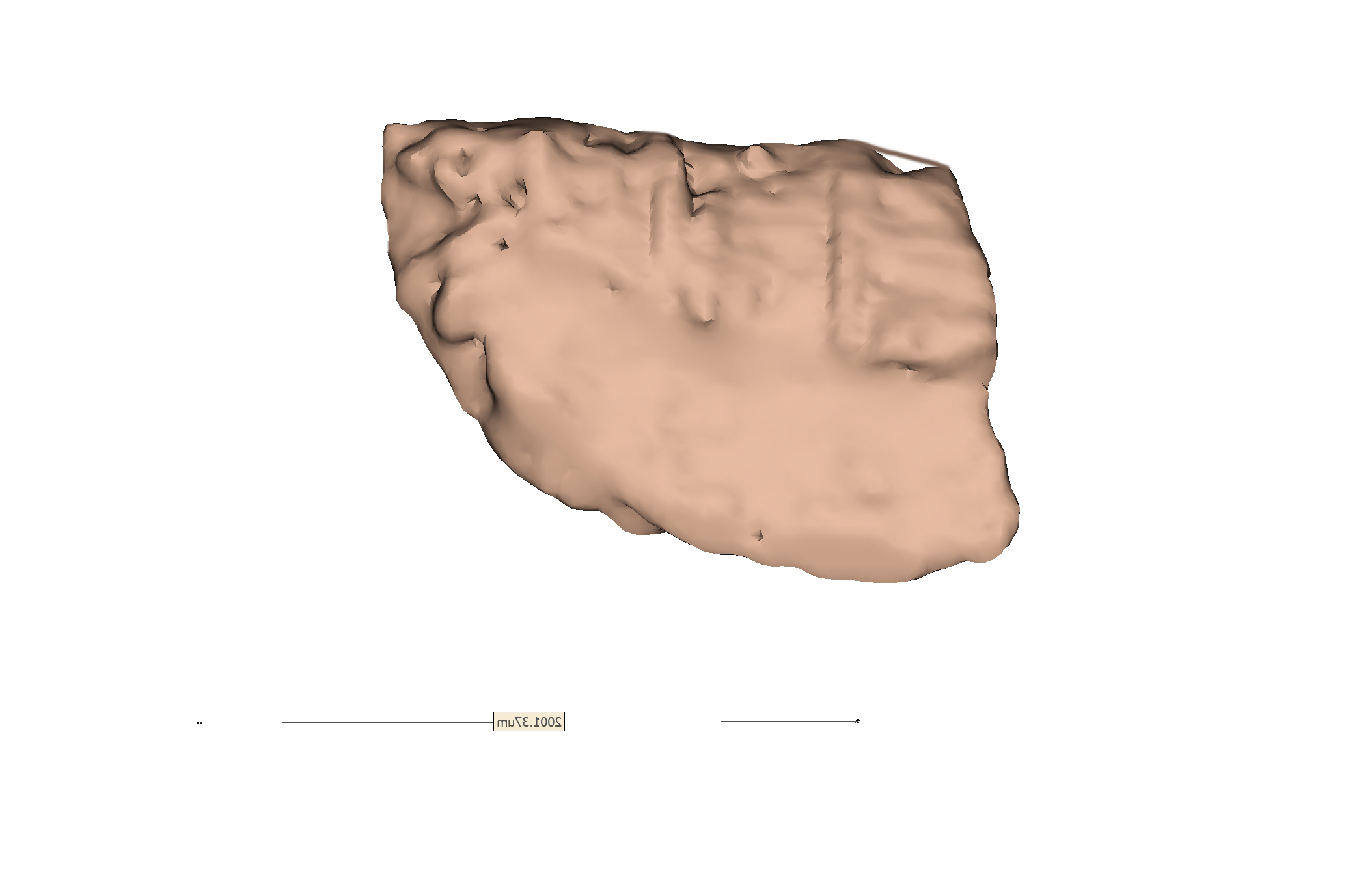

Supplement: Supplementary file 5 — Supplementary Data 2 [file 41467_2023_43557_MOESM5_ESM.zip › Supplementary Data 2/Supplementary Data 2 Raw data of Geometric Morphometric Analyses/12 Morphotypes/Morphotype 5/t05r.jpg]
