# Supplementary figures and images for: Bony-fish-like scales in a Silurian maxillate placoderm (part 1 of 2)
Source: Nat Commun. 2023 Nov 22;14:7622. doi: 10.1038/s41467-023-43557-9 (PMC10665347; doi:10.1038/s41467-023-43557-9)

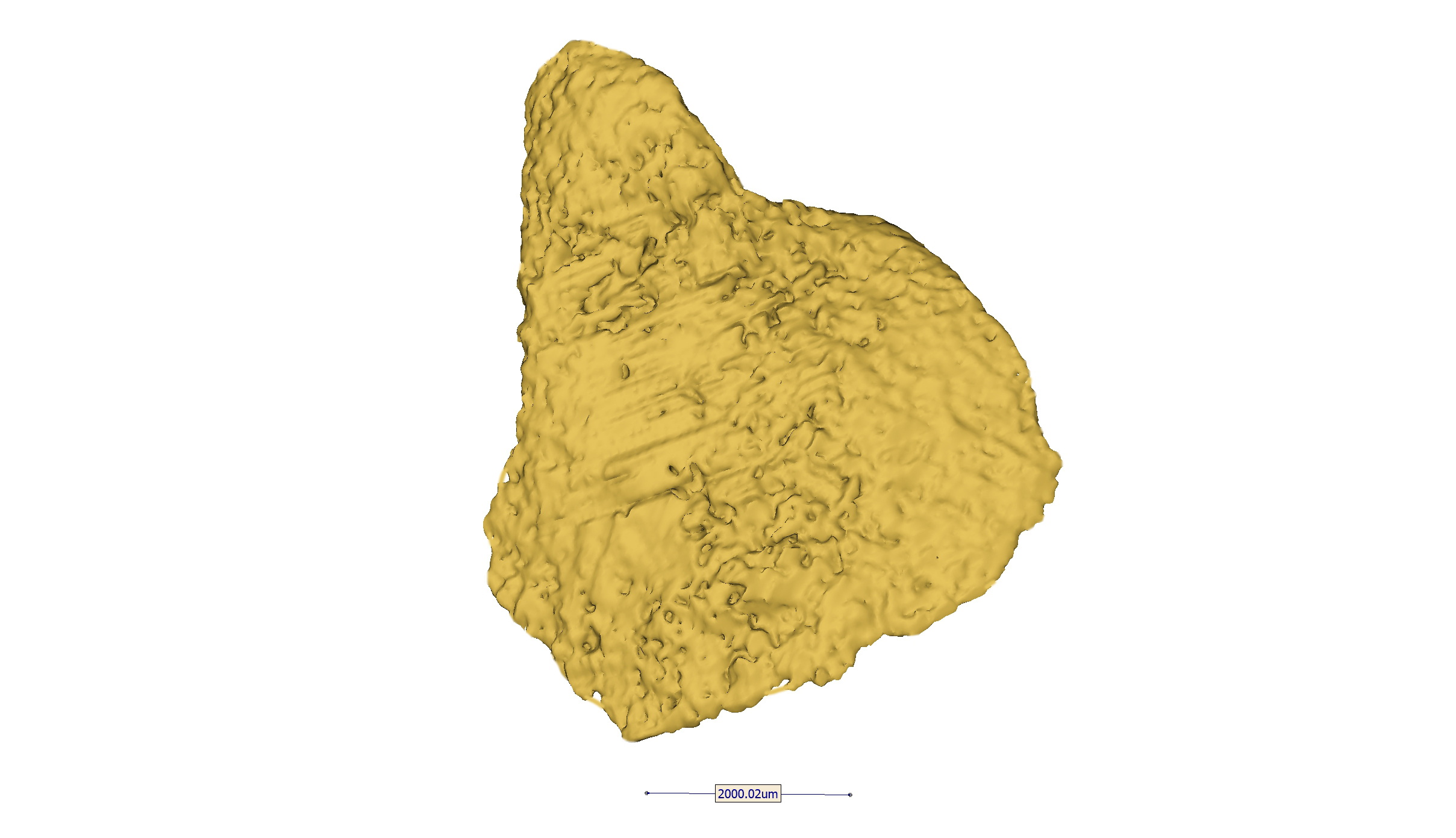

Supplement: Supplementary file 5 — Supplementary Data 2 [file 41467_2023_43557_MOESM5_ESM.zip › Supplementary Data 2/Supplementary Data 2 Raw data of Geometric Morphometric Analyses/12 Morphotypes/l2d13.jpg]

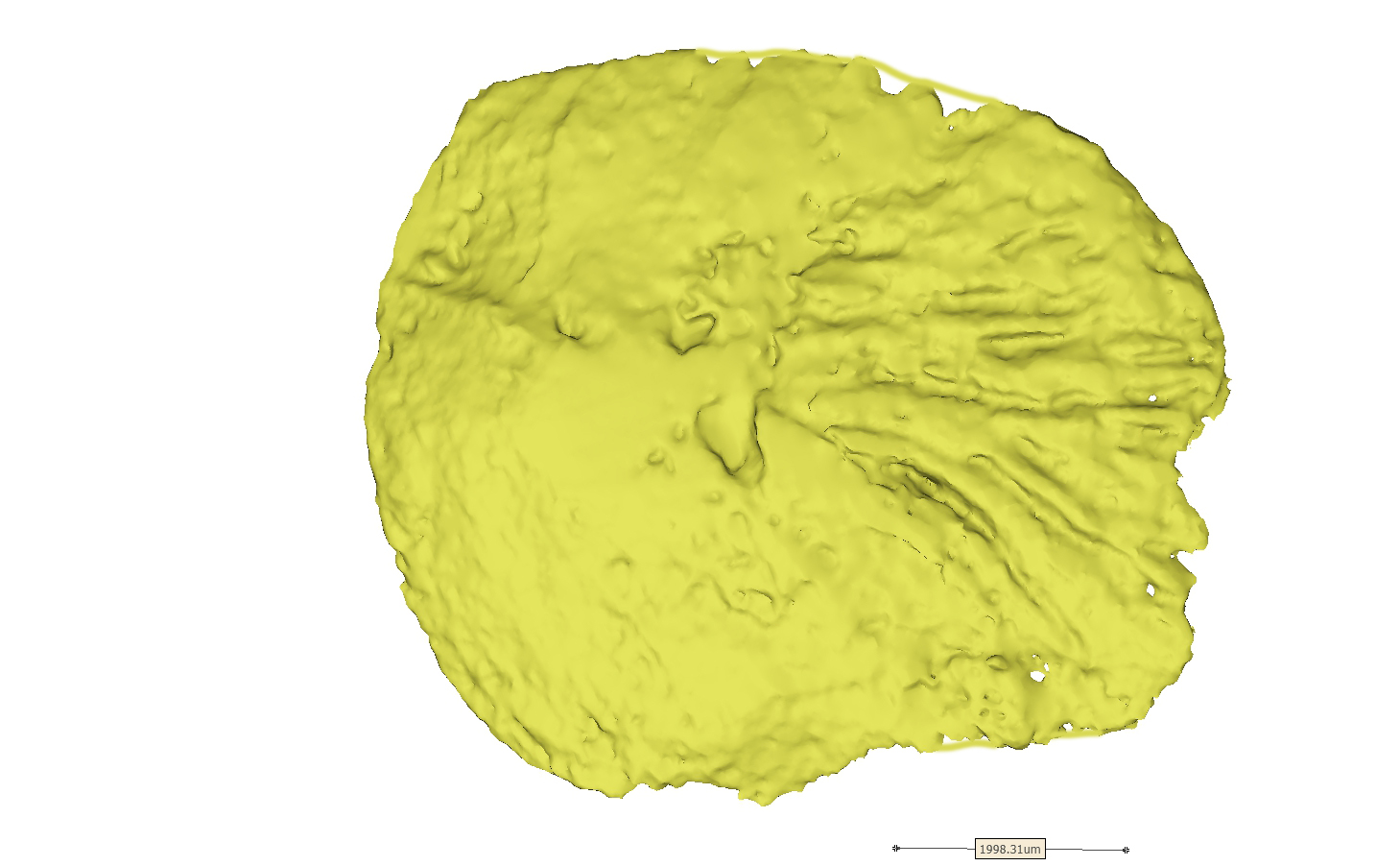

Supplement: Supplementary file 5 — Supplementary Data 2 [file 41467_2023_43557_MOESM5_ESM.zip › Supplementary Data 2/Supplementary Data 2 Raw data of Geometric Morphometric Analyses/12 Morphotypes/l3v04.jpg]

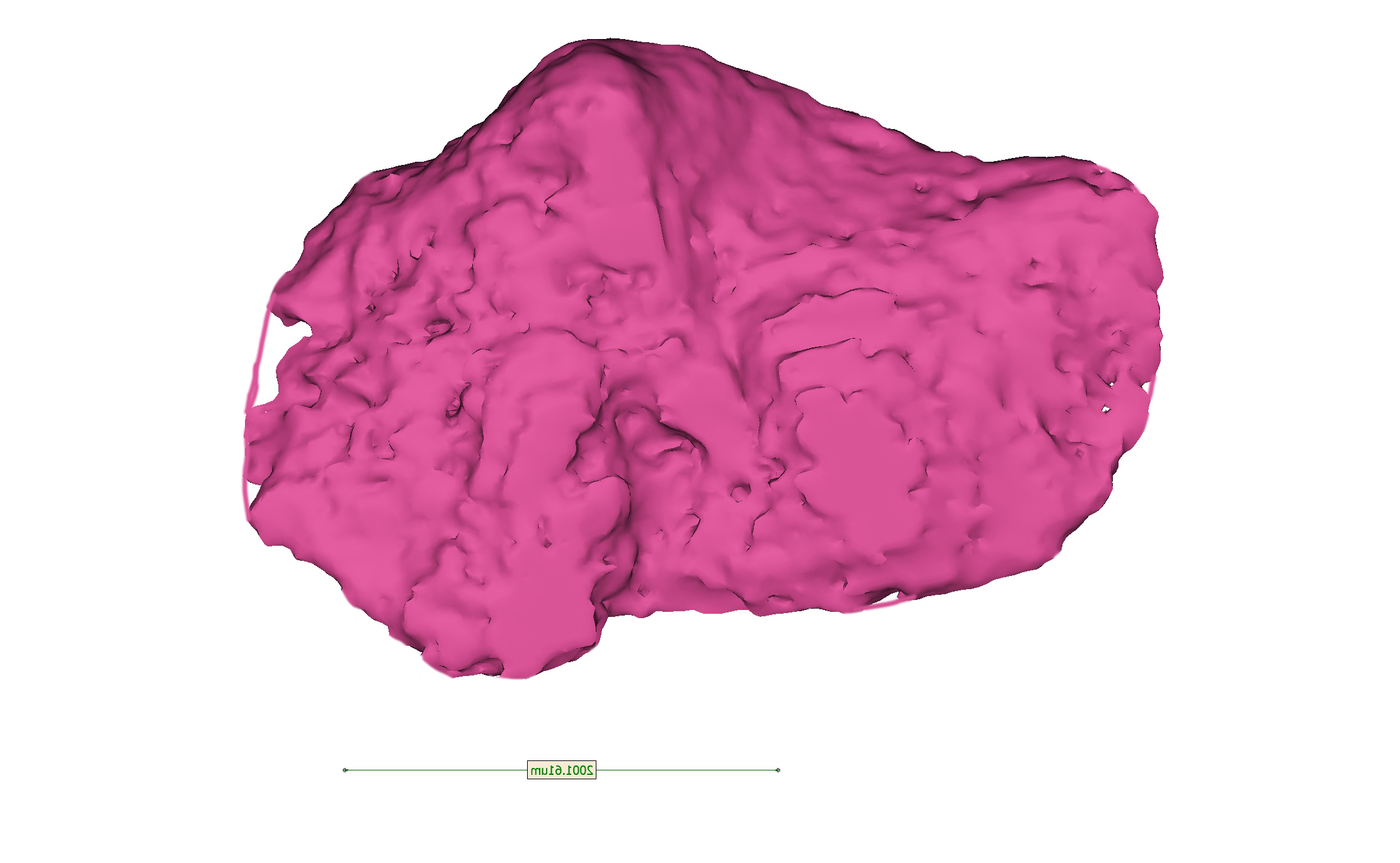

Supplement: Supplementary file 5 — Supplementary Data 2 [file 41467_2023_43557_MOESM5_ESM.zip › Supplementary Data 2/Supplementary Data 2 Raw data of Geometric Morphometric Analyses/12 Morphotypes/l6v05-.jpg]

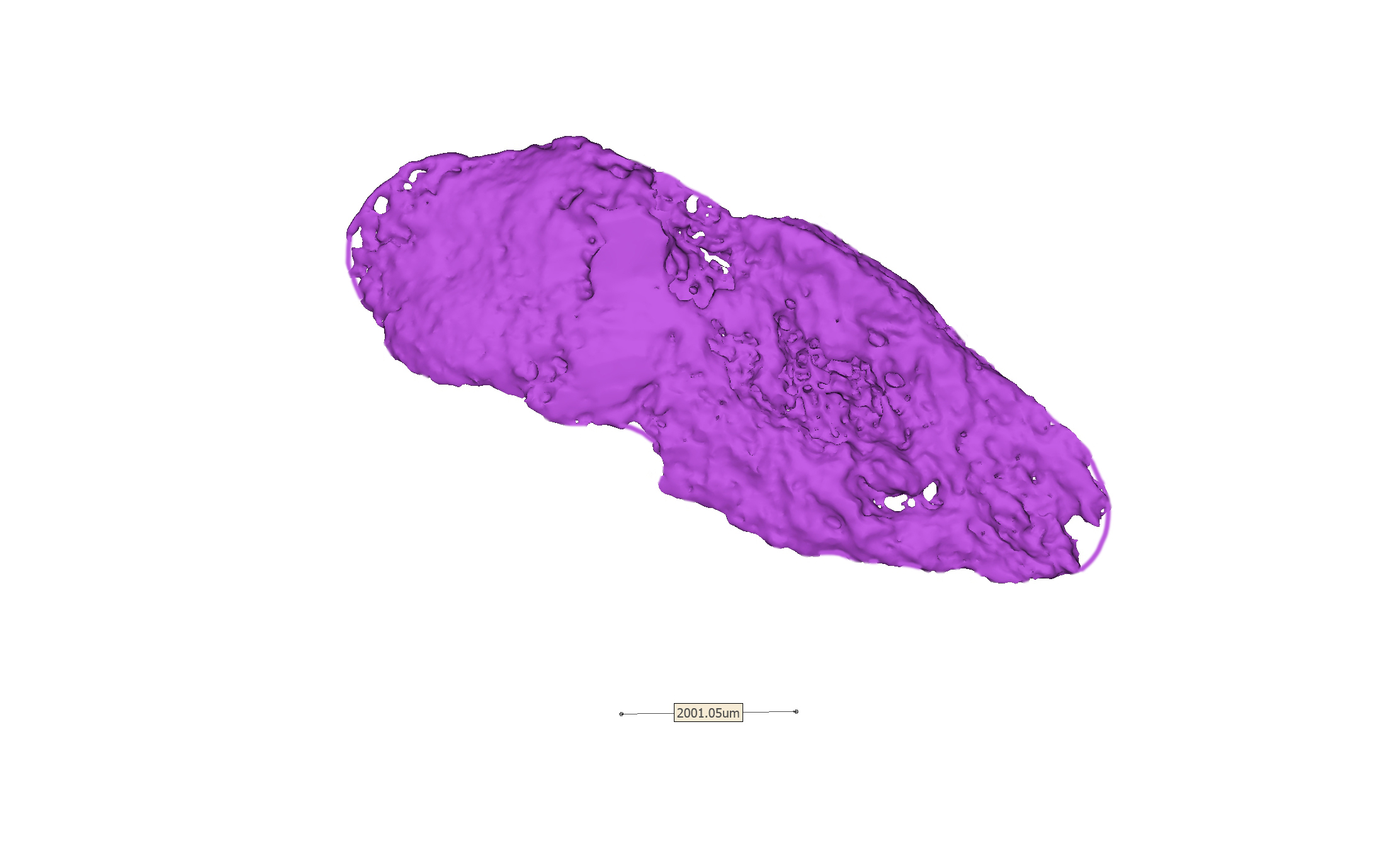

Supplement: Supplementary file 5 — Supplementary Data 2 [file 41467_2023_43557_MOESM5_ESM.zip › Supplementary Data 2/Supplementary Data 2 Raw data of Geometric Morphometric Analyses/12 Morphotypes/l6v08-.jpg]

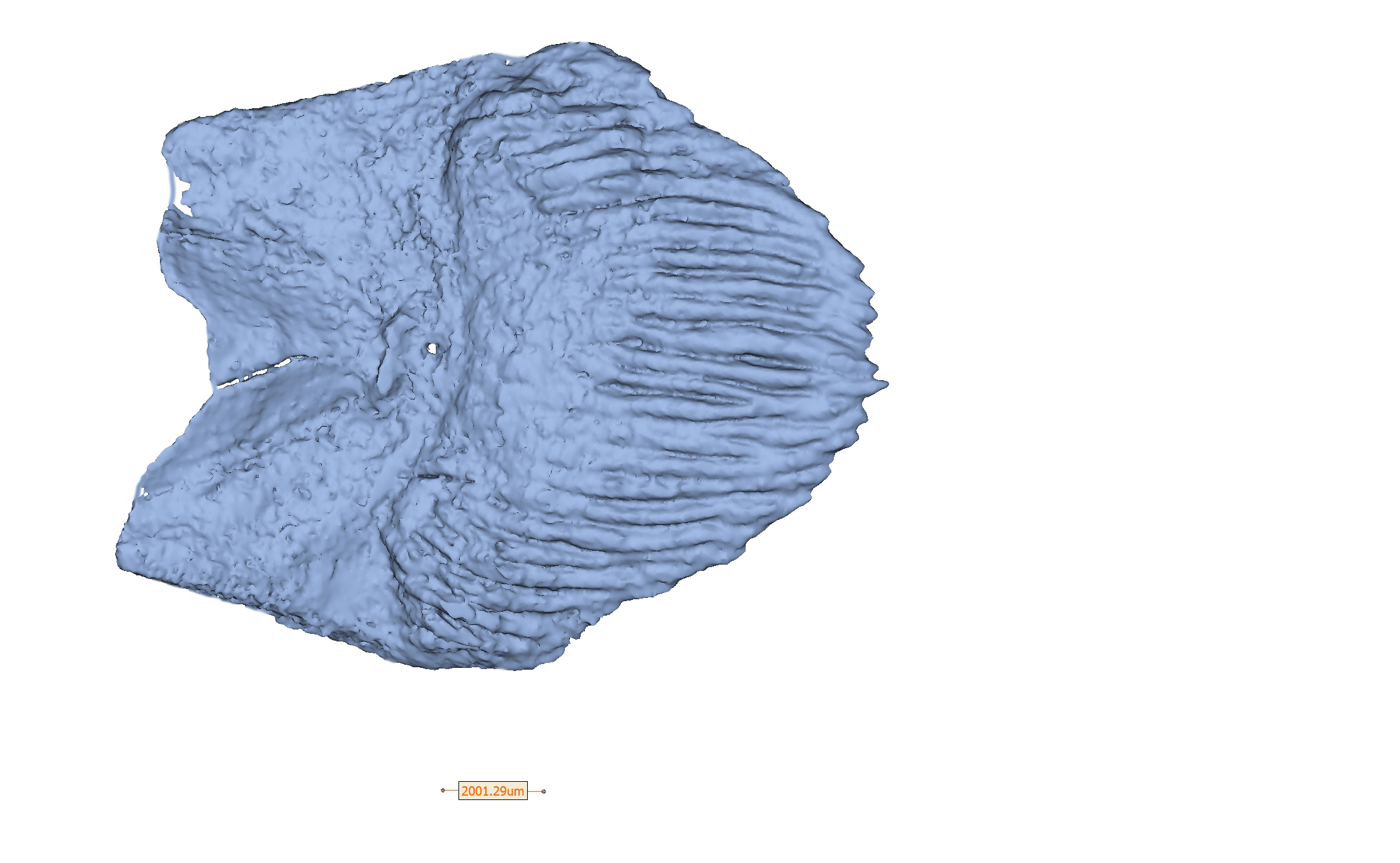

Supplement: Supplementary file 5 — Supplementary Data 2 [file 41467_2023_43557_MOESM5_ESM.zip › Supplementary Data 2/Supplementary Data 2 Raw data of Geometric Morphometric Analyses/12 Morphotypes/md1.jpg]

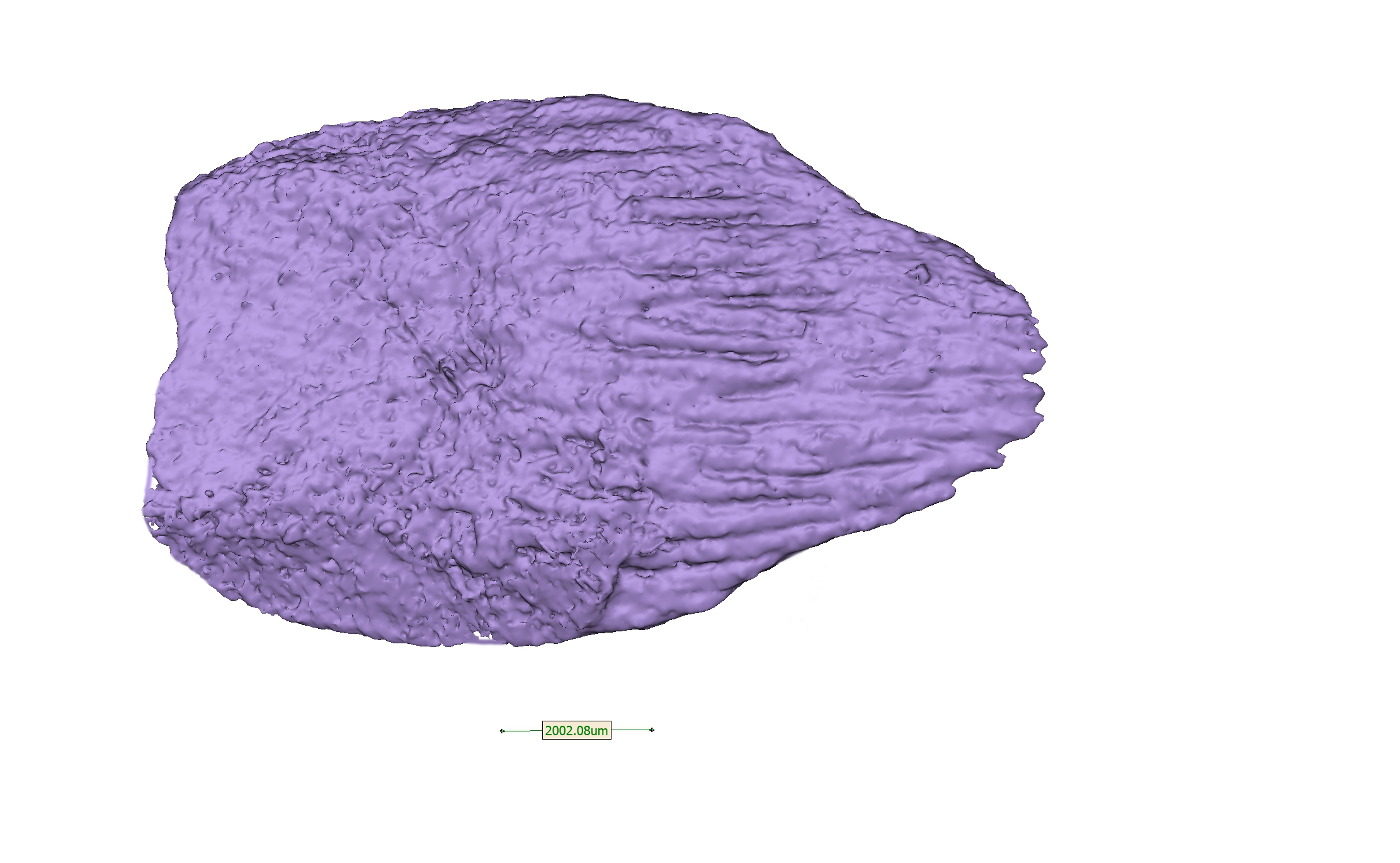

Supplement: Supplementary file 5 — Supplementary Data 2 [file 41467_2023_43557_MOESM5_ESM.zip › Supplementary Data 2/Supplementary Data 2 Raw data of Geometric Morphometric Analyses/12 Morphotypes/md2.jpg]

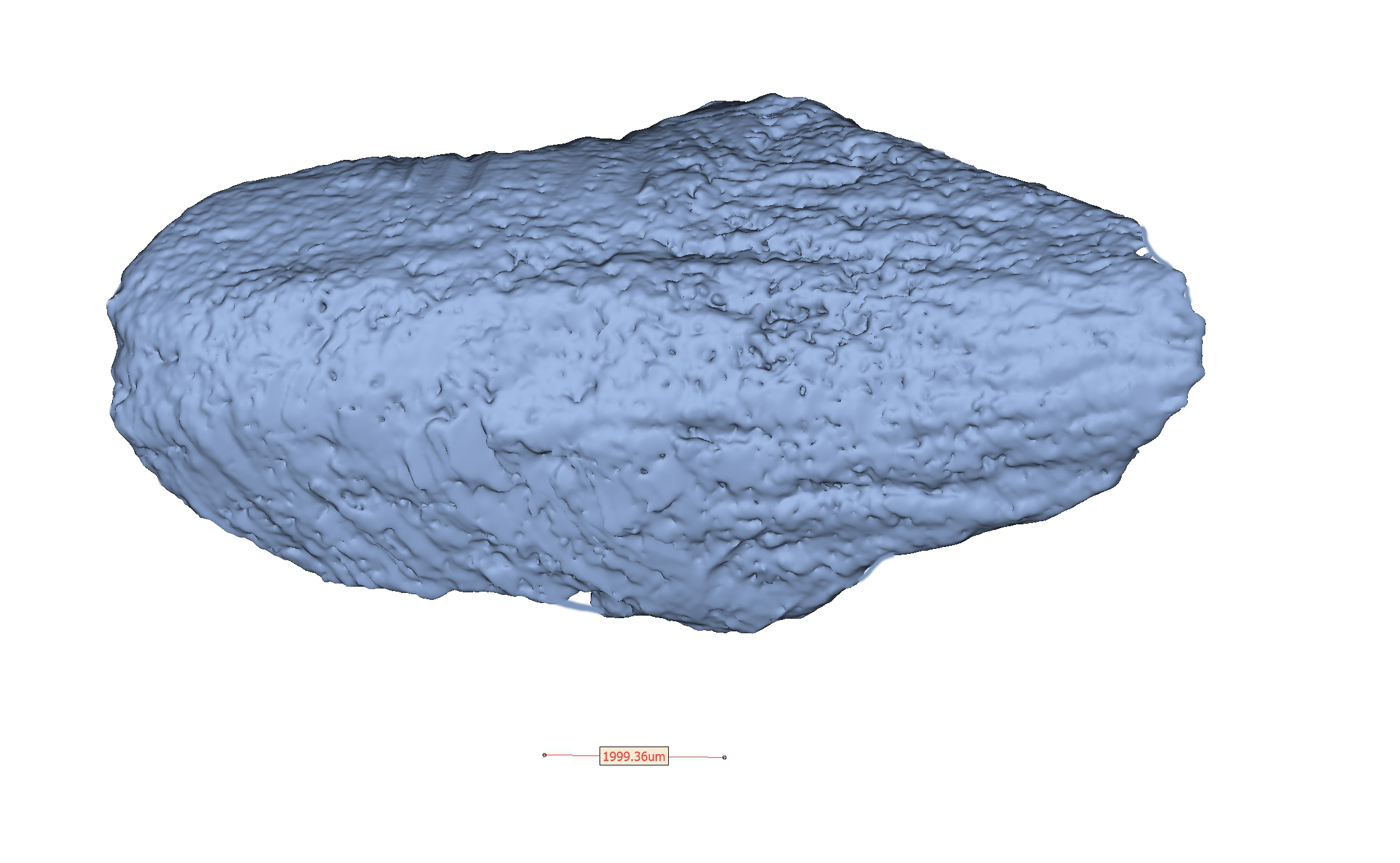

Supplement: Supplementary file 5 — Supplementary Data 2 [file 41467_2023_43557_MOESM5_ESM.zip › Supplementary Data 2/Supplementary Data 2 Raw data of Geometric Morphometric Analyses/12 Morphotypes/md3.jpg]

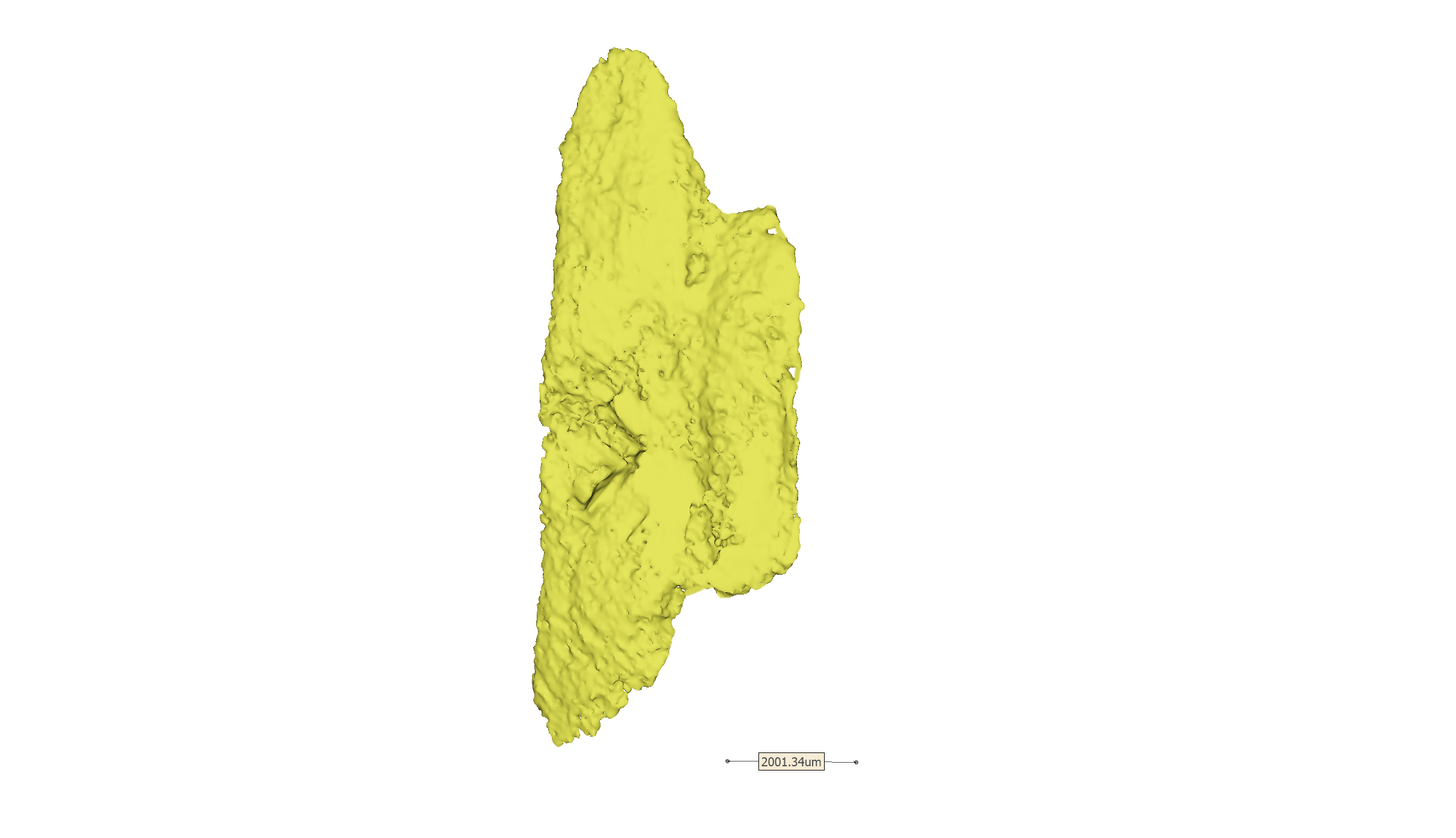

Supplement: Supplementary file 5 — Supplementary Data 2 [file 41467_2023_43557_MOESM5_ESM.zip › Supplementary Data 2/Supplementary Data 2 Raw data of Geometric Morphometric Analyses/12 Morphotypes/Morphotype 1/l1v01.jpg]

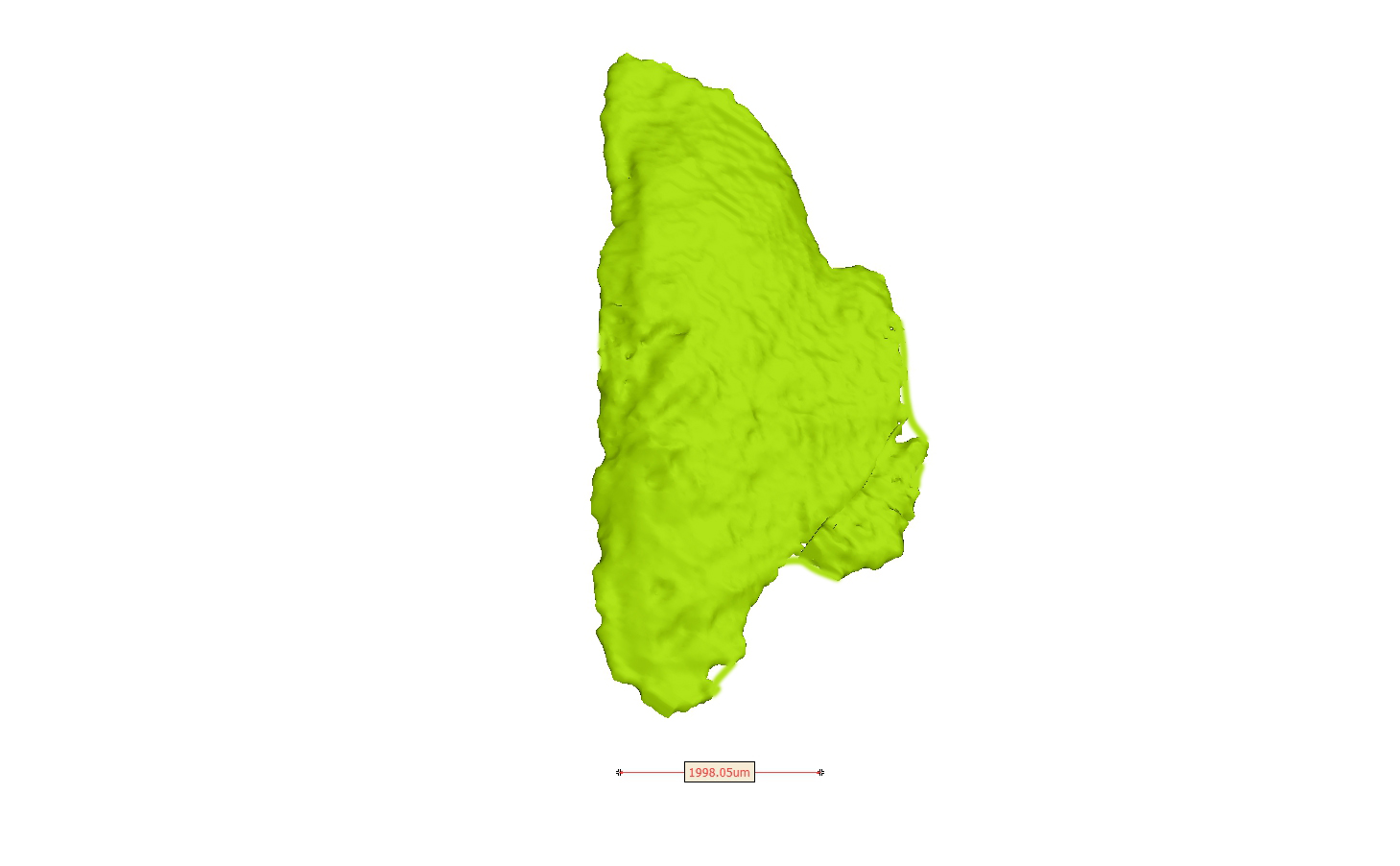

Supplement: Supplementary file 5 — Supplementary Data 2 [file 41467_2023_43557_MOESM5_ESM.zip › Supplementary Data 2/Supplementary Data 2 Raw data of Geometric Morphometric Analyses/12 Morphotypes/Morphotype 1/l4v01.jpg]

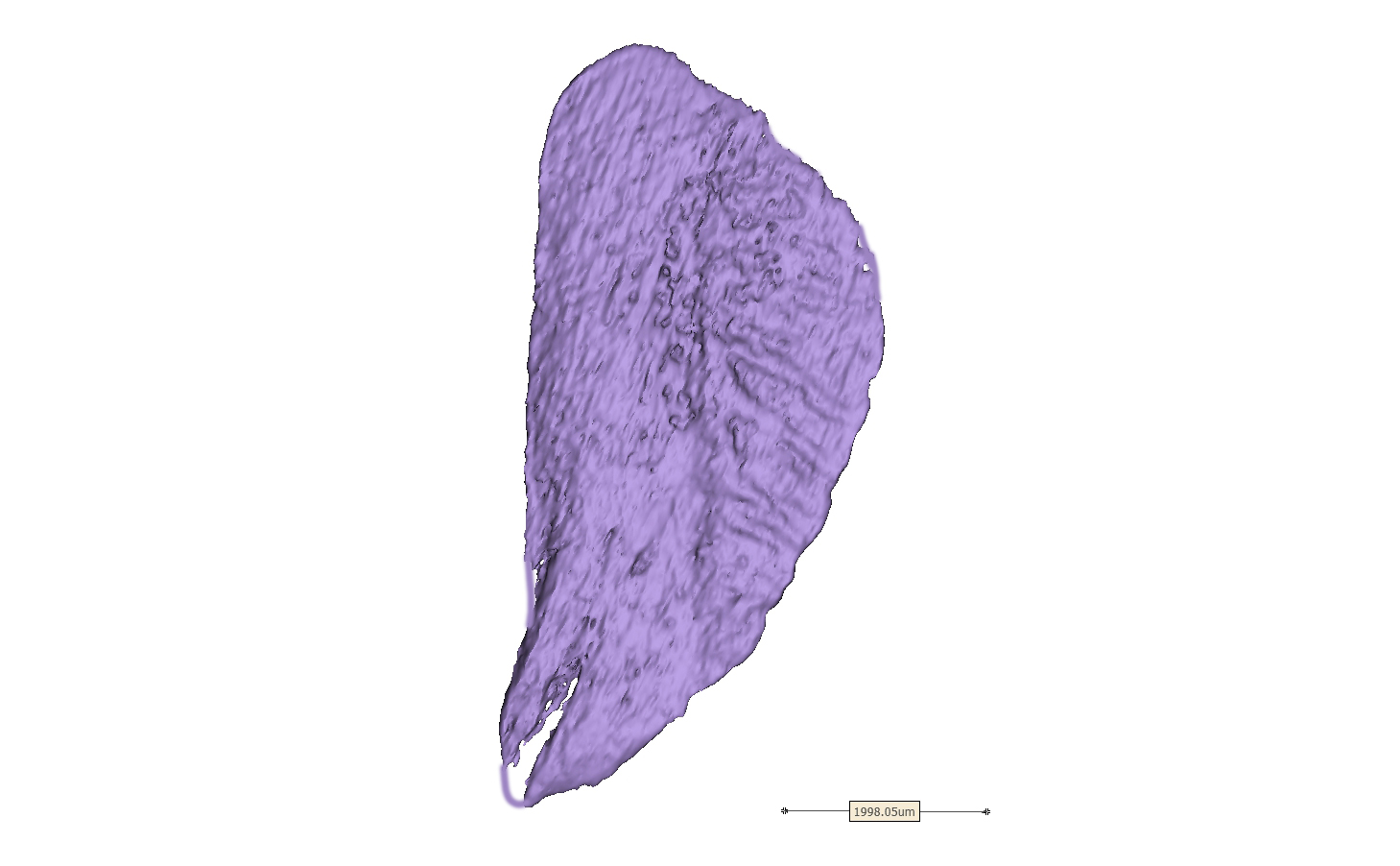

Supplement: Supplementary file 5 — Supplementary Data 2 [file 41467_2023_43557_MOESM5_ESM.zip › Supplementary Data 2/Supplementary Data 2 Raw data of Geometric Morphometric Analyses/12 Morphotypes/Morphotype 1/l4v02.jpg]

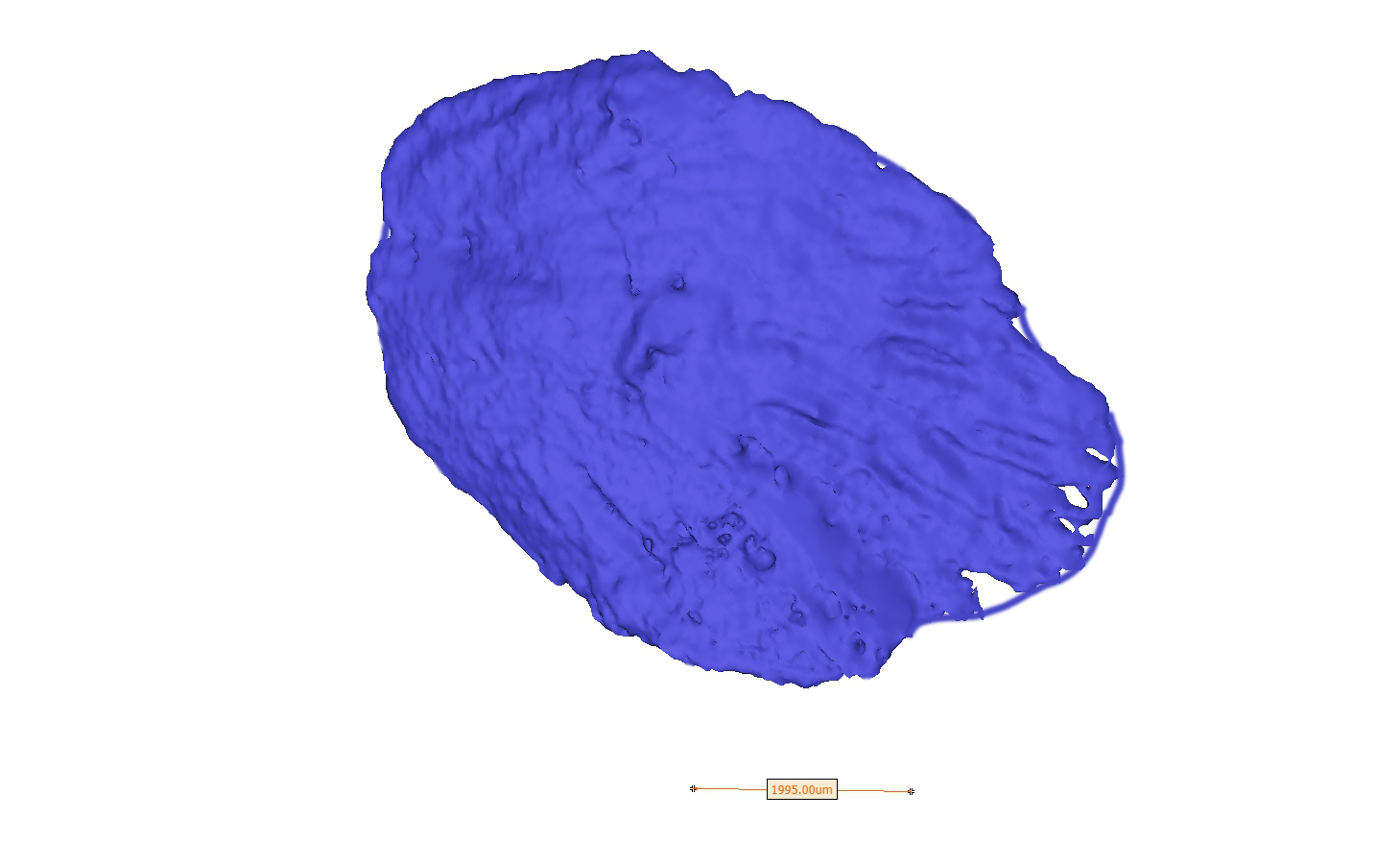

Supplement: Supplementary file 5 — Supplementary Data 2 [file 41467_2023_43557_MOESM5_ESM.zip › Supplementary Data 2/Supplementary Data 2 Raw data of Geometric Morphometric Analyses/12 Morphotypes/Morphotype 10/l4v04.jpg]

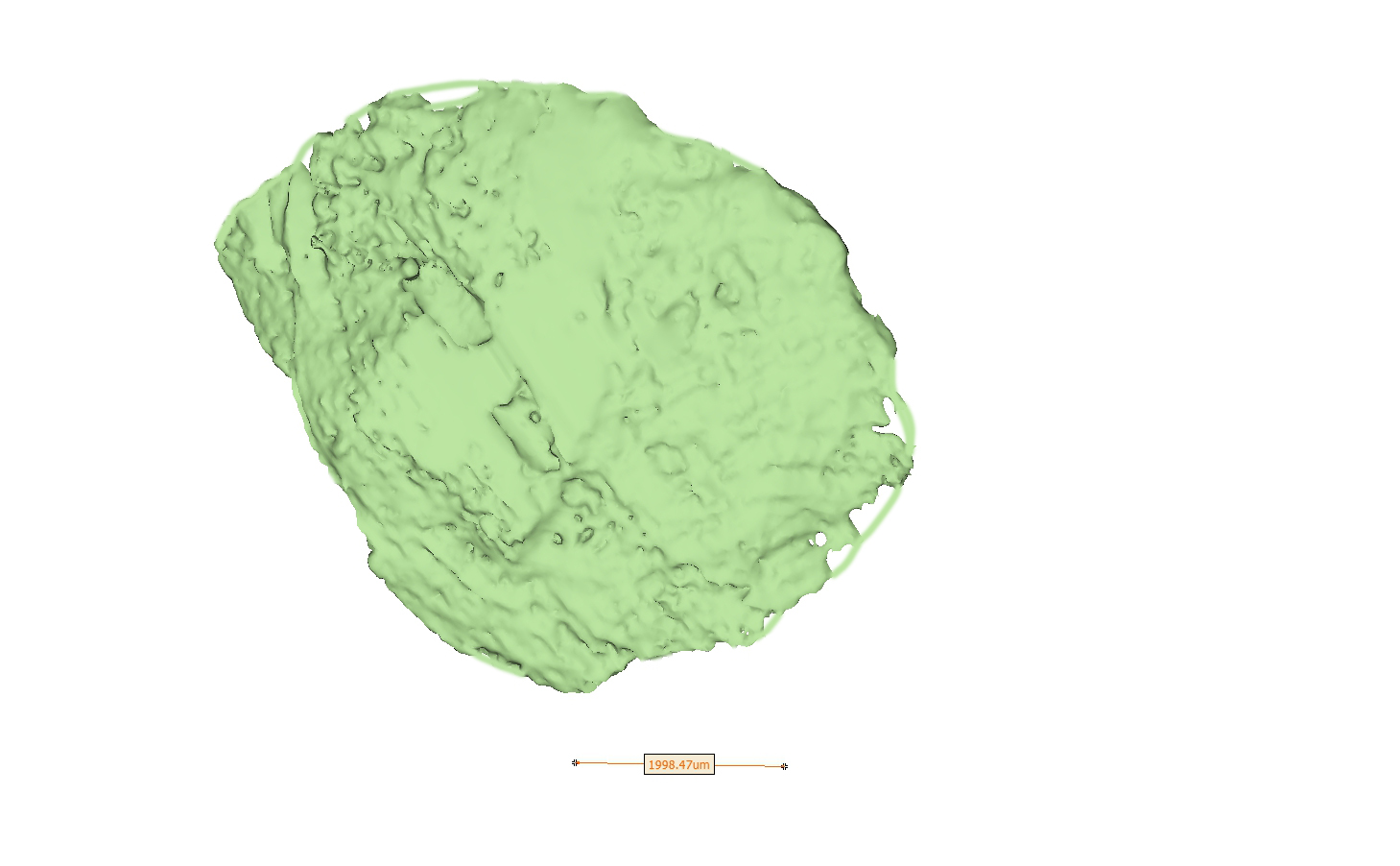

Supplement: Supplementary file 5 — Supplementary Data 2 [file 41467_2023_43557_MOESM5_ESM.zip › Supplementary Data 2/Supplementary Data 2 Raw data of Geometric Morphometric Analyses/12 Morphotypes/Morphotype 10/l4v08.jpg]

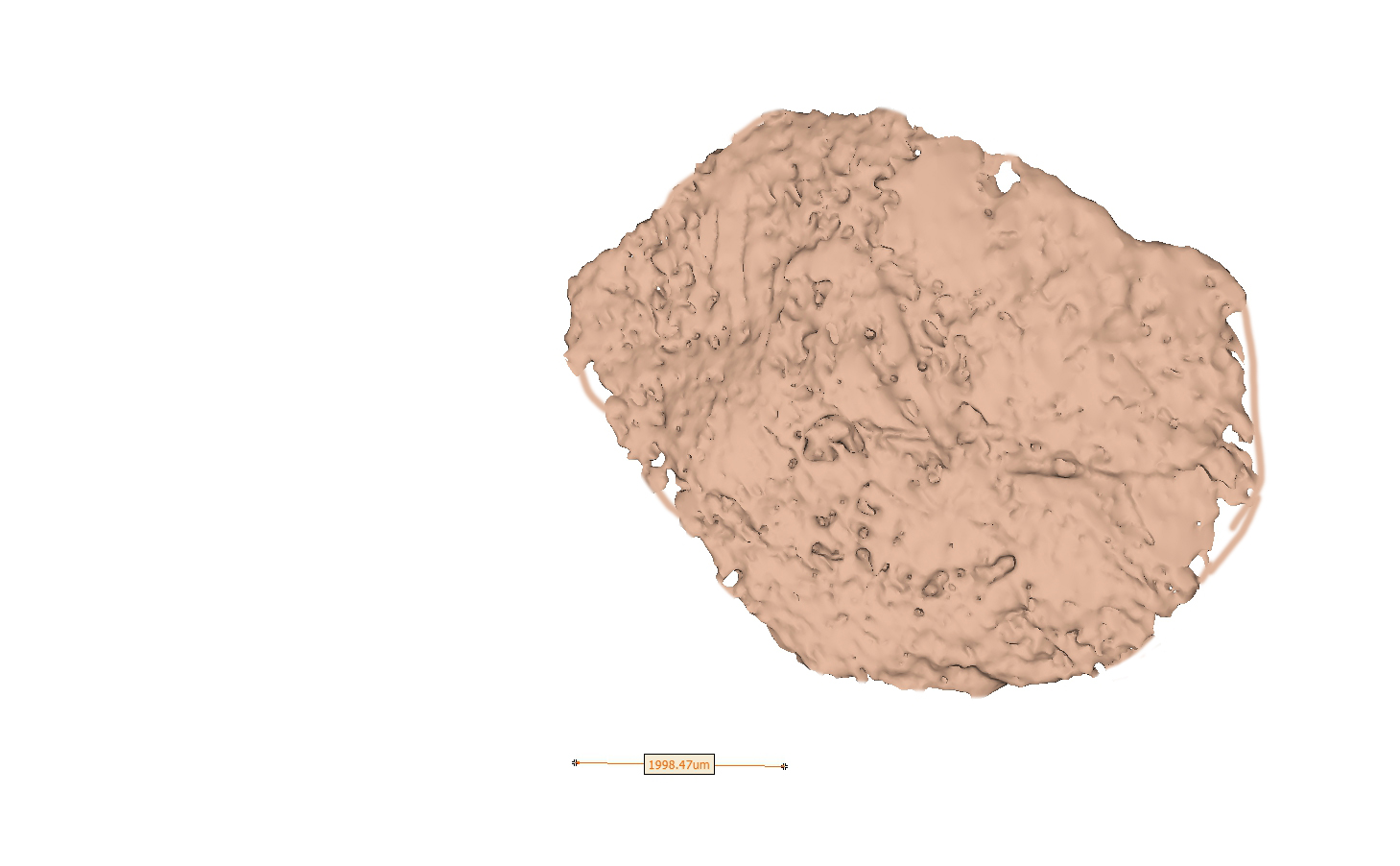

Supplement: Supplementary file 5 — Supplementary Data 2 [file 41467_2023_43557_MOESM5_ESM.zip › Supplementary Data 2/Supplementary Data 2 Raw data of Geometric Morphometric Analyses/12 Morphotypes/Morphotype 10/l4v09.jpg]

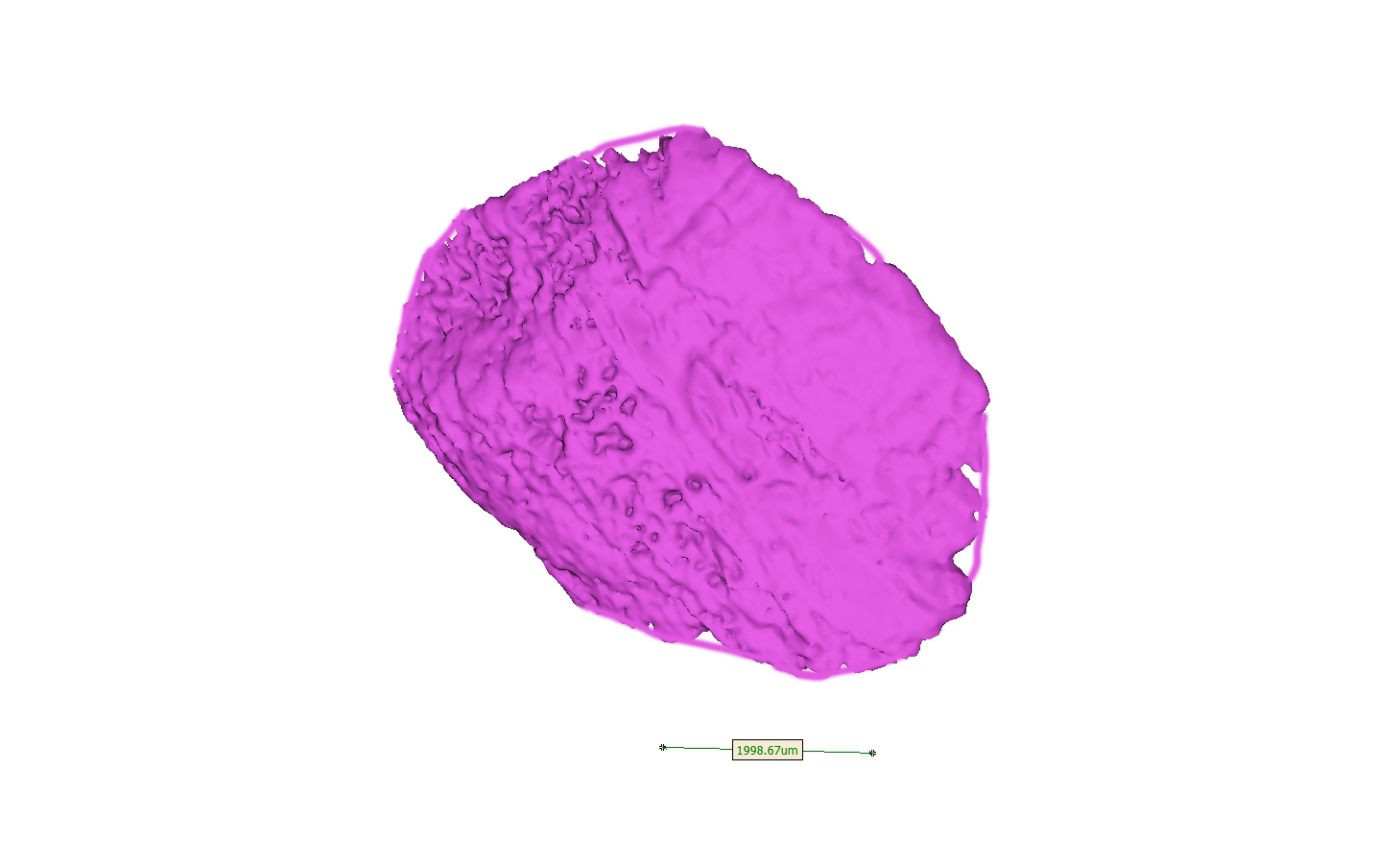

Supplement: Supplementary file 5 — Supplementary Data 2 [file 41467_2023_43557_MOESM5_ESM.zip › Supplementary Data 2/Supplementary Data 2 Raw data of Geometric Morphometric Analyses/12 Morphotypes/Morphotype 10/l4v10.jpg]

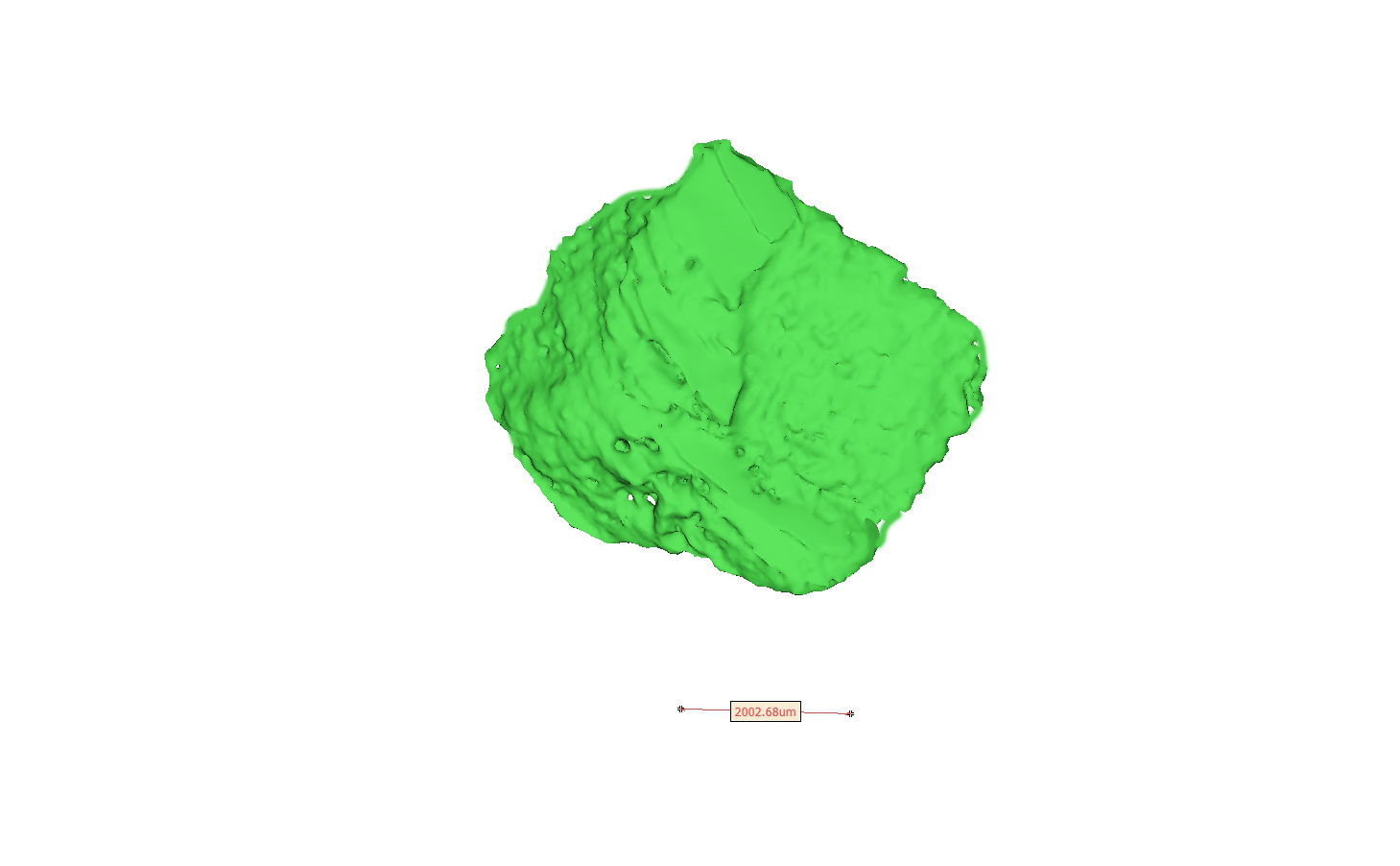

Supplement: Supplementary file 5 — Supplementary Data 2 [file 41467_2023_43557_MOESM5_ESM.zip › Supplementary Data 2/Supplementary Data 2 Raw data of Geometric Morphometric Analyses/12 Morphotypes/Morphotype 10/l4v11.jpg]

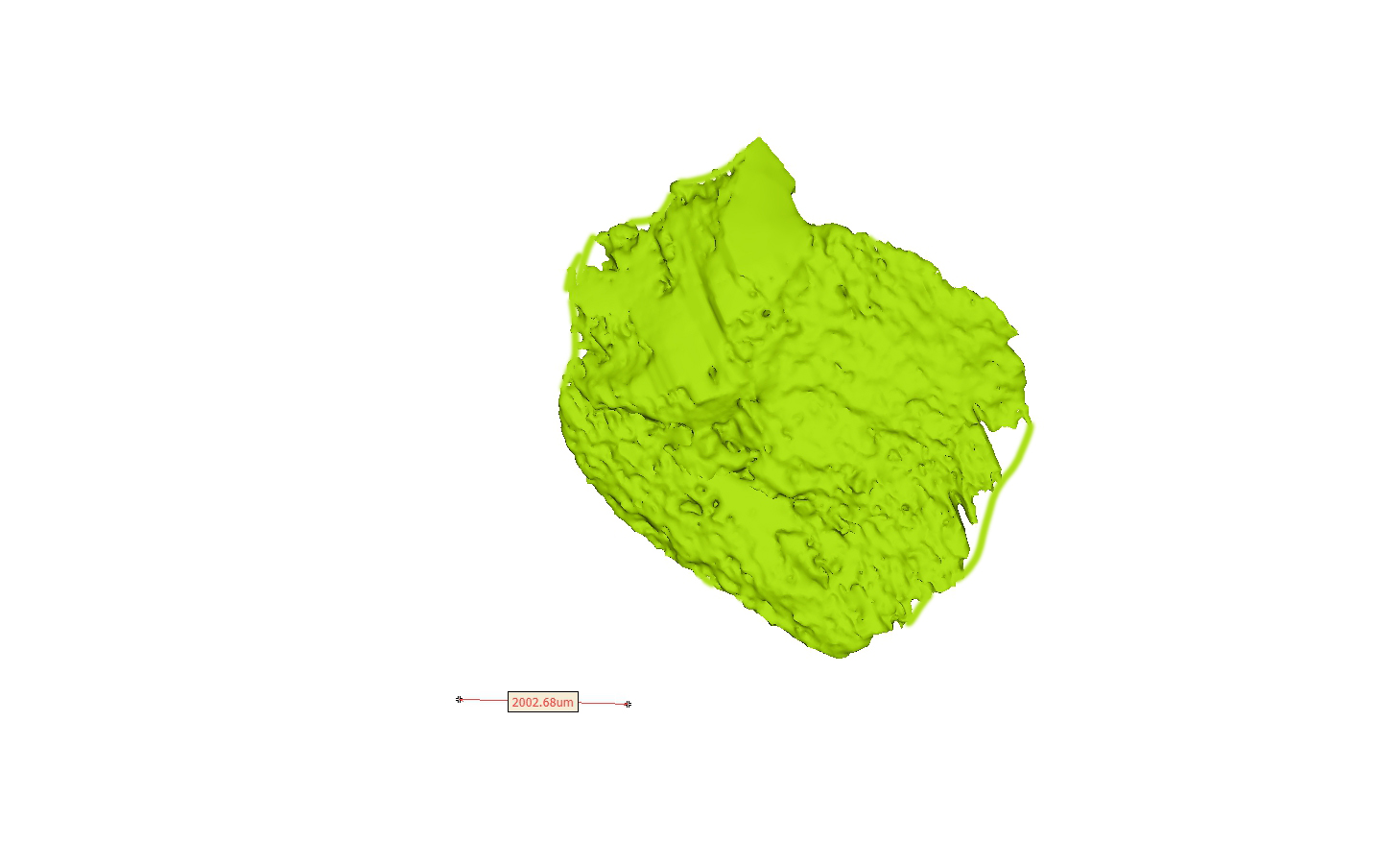

Supplement: Supplementary file 5 — Supplementary Data 2 [file 41467_2023_43557_MOESM5_ESM.zip › Supplementary Data 2/Supplementary Data 2 Raw data of Geometric Morphometric Analyses/12 Morphotypes/Morphotype 10/l4v12.jpg]

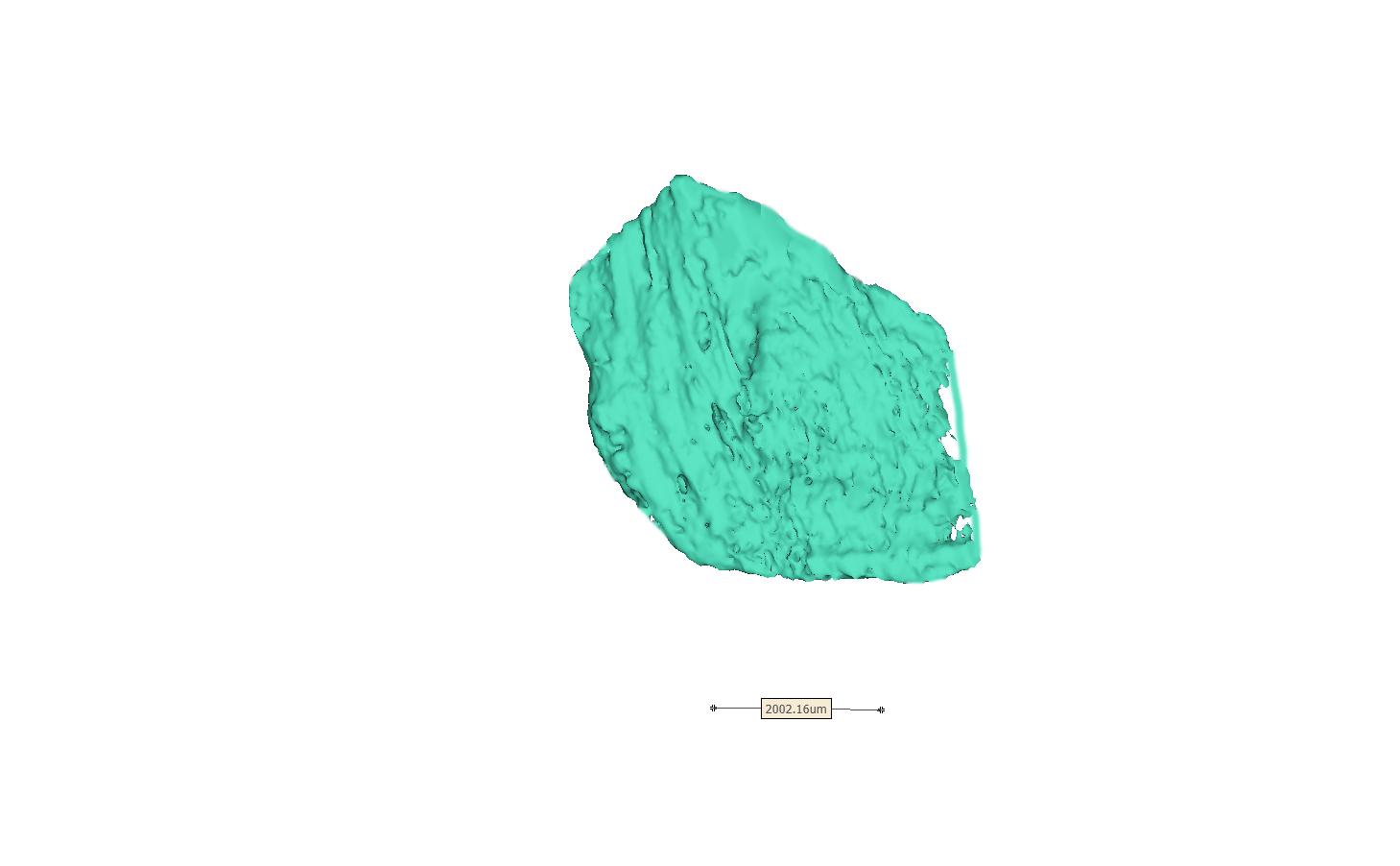

Supplement: Supplementary file 5 — Supplementary Data 2 [file 41467_2023_43557_MOESM5_ESM.zip › Supplementary Data 2/Supplementary Data 2 Raw data of Geometric Morphometric Analyses/12 Morphotypes/Morphotype 10/l4v13.jpg]

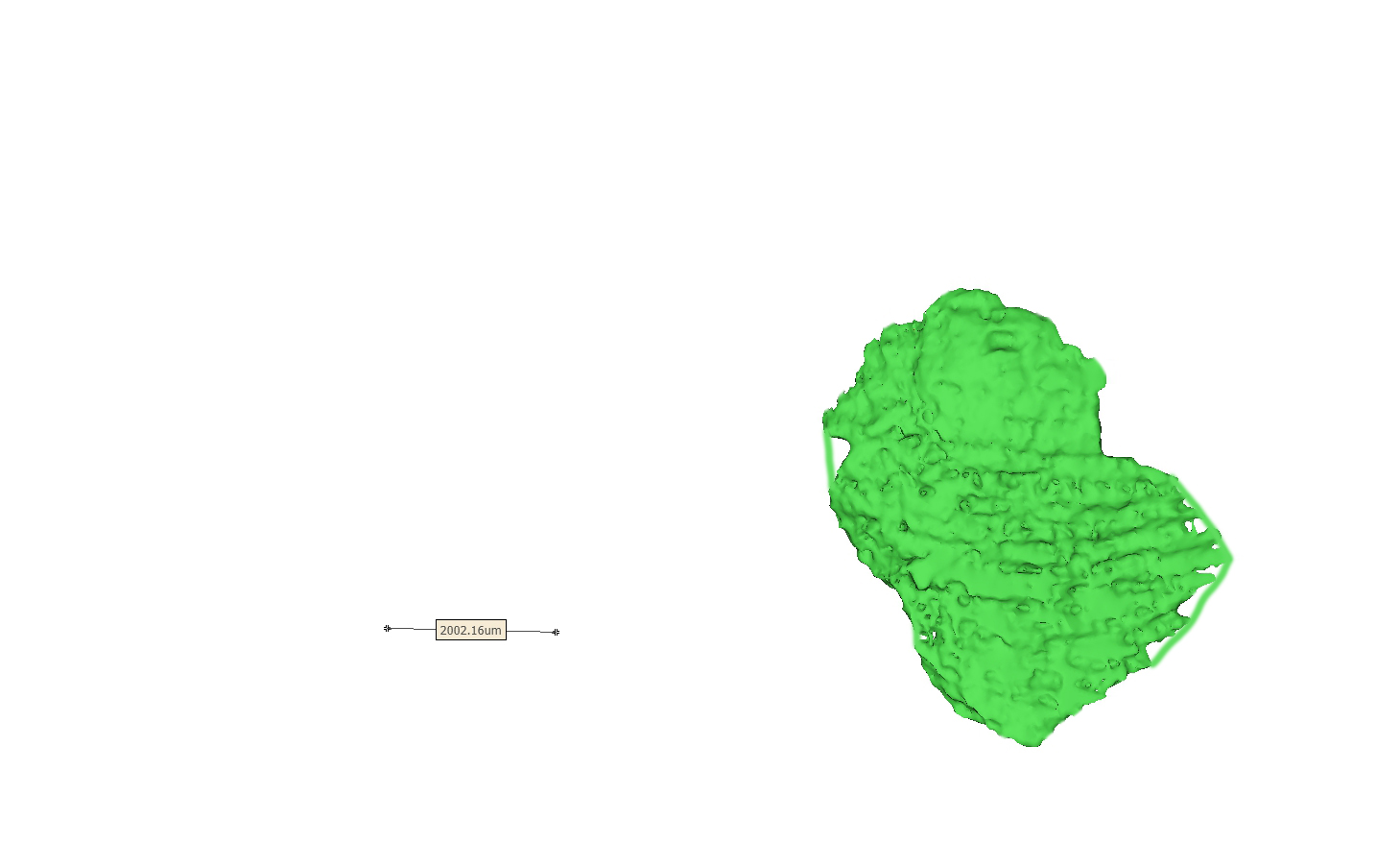

Supplement: Supplementary file 5 — Supplementary Data 2 [file 41467_2023_43557_MOESM5_ESM.zip › Supplementary Data 2/Supplementary Data 2 Raw data of Geometric Morphometric Analyses/12 Morphotypes/Morphotype 10/l4v14.jpg]

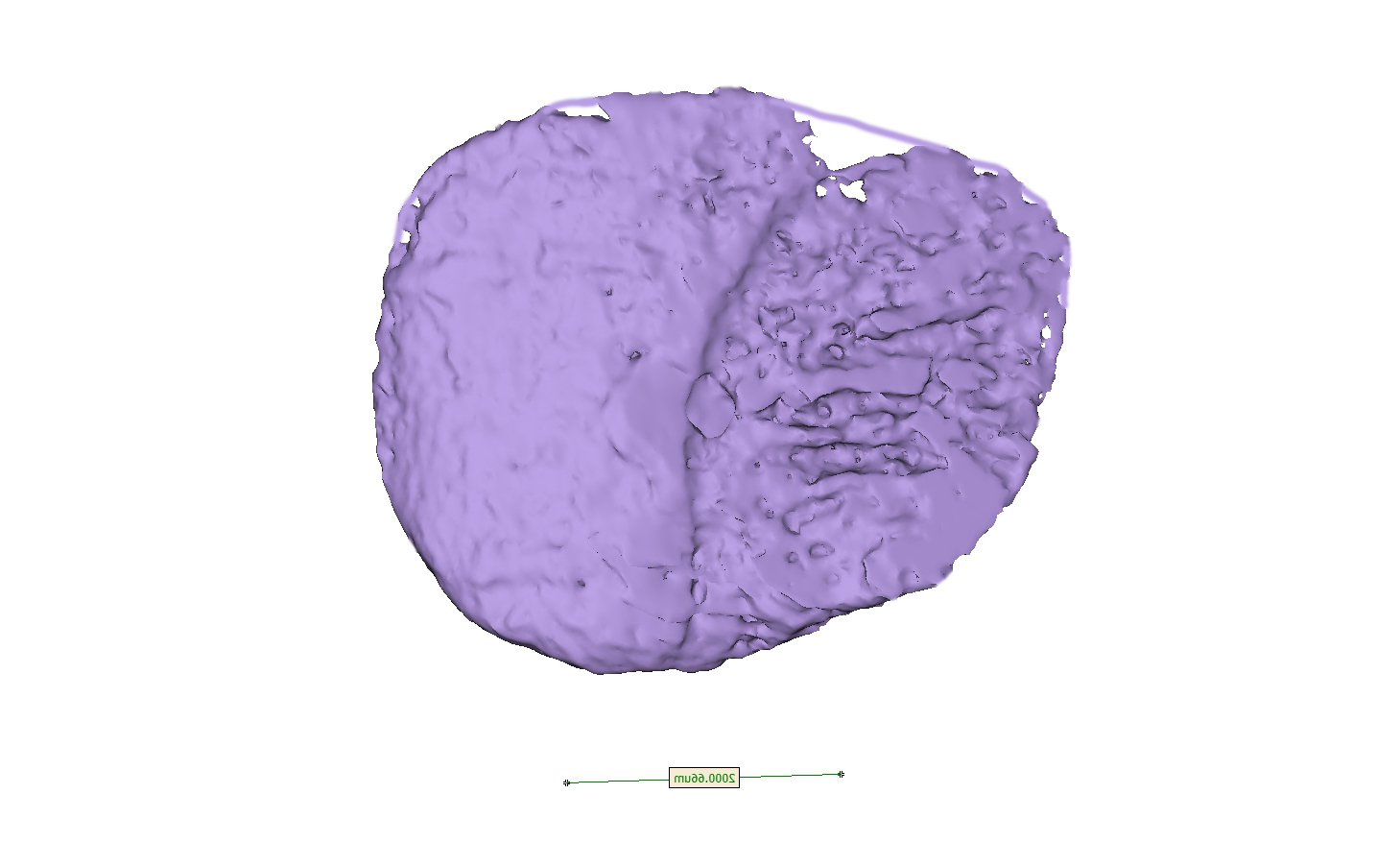

Supplement: Supplementary file 5 — Supplementary Data 2 [file 41467_2023_43557_MOESM5_ESM.zip › Supplementary Data 2/Supplementary Data 2 Raw data of Geometric Morphometric Analyses/12 Morphotypes/Morphotype 10/l5v05-.jpg]

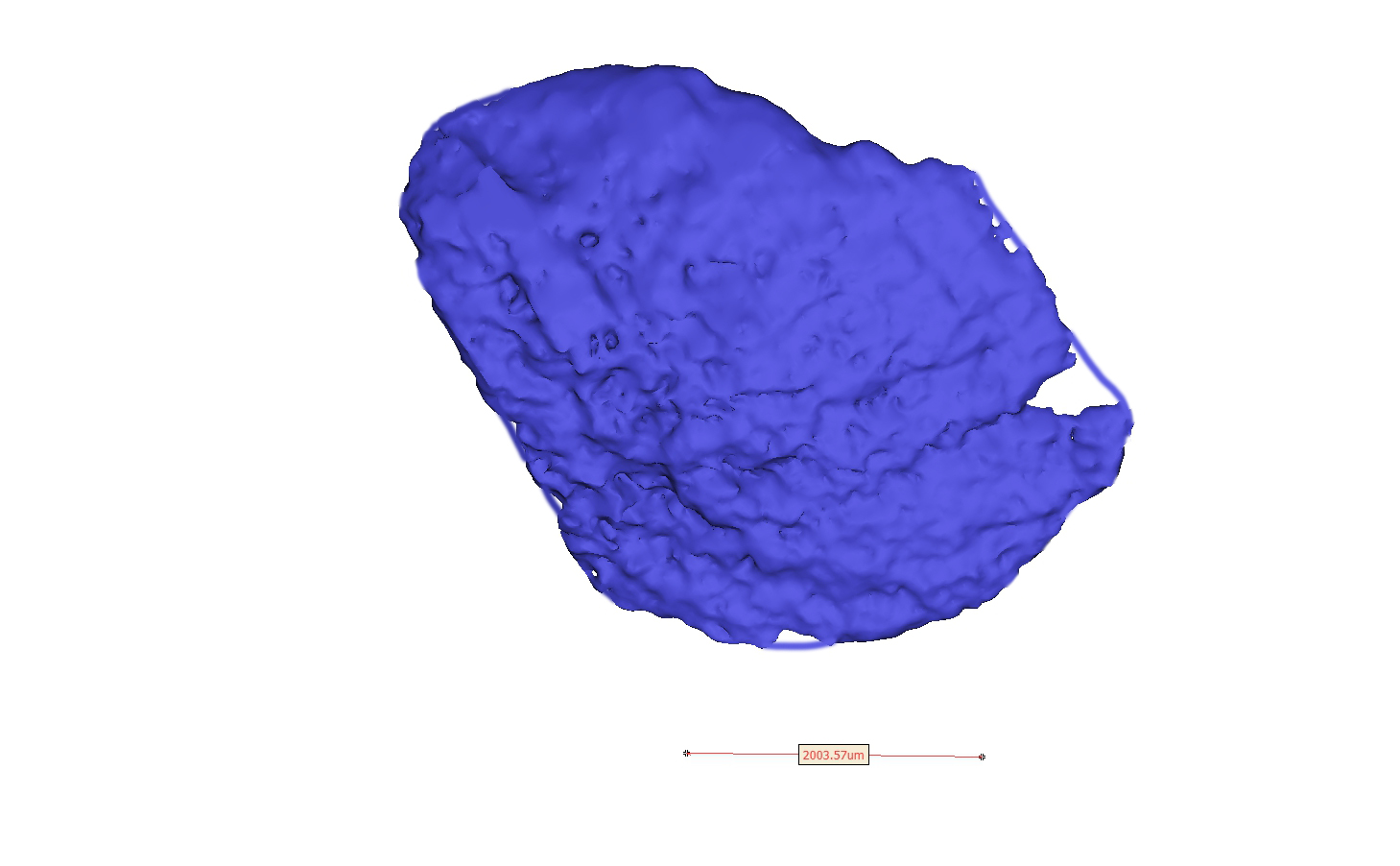

Supplement: Supplementary file 5 — Supplementary Data 2 [file 41467_2023_43557_MOESM5_ESM.zip › Supplementary Data 2/Supplementary Data 2 Raw data of Geometric Morphometric Analyses/12 Morphotypes/Morphotype 10/l5v10.jpg]

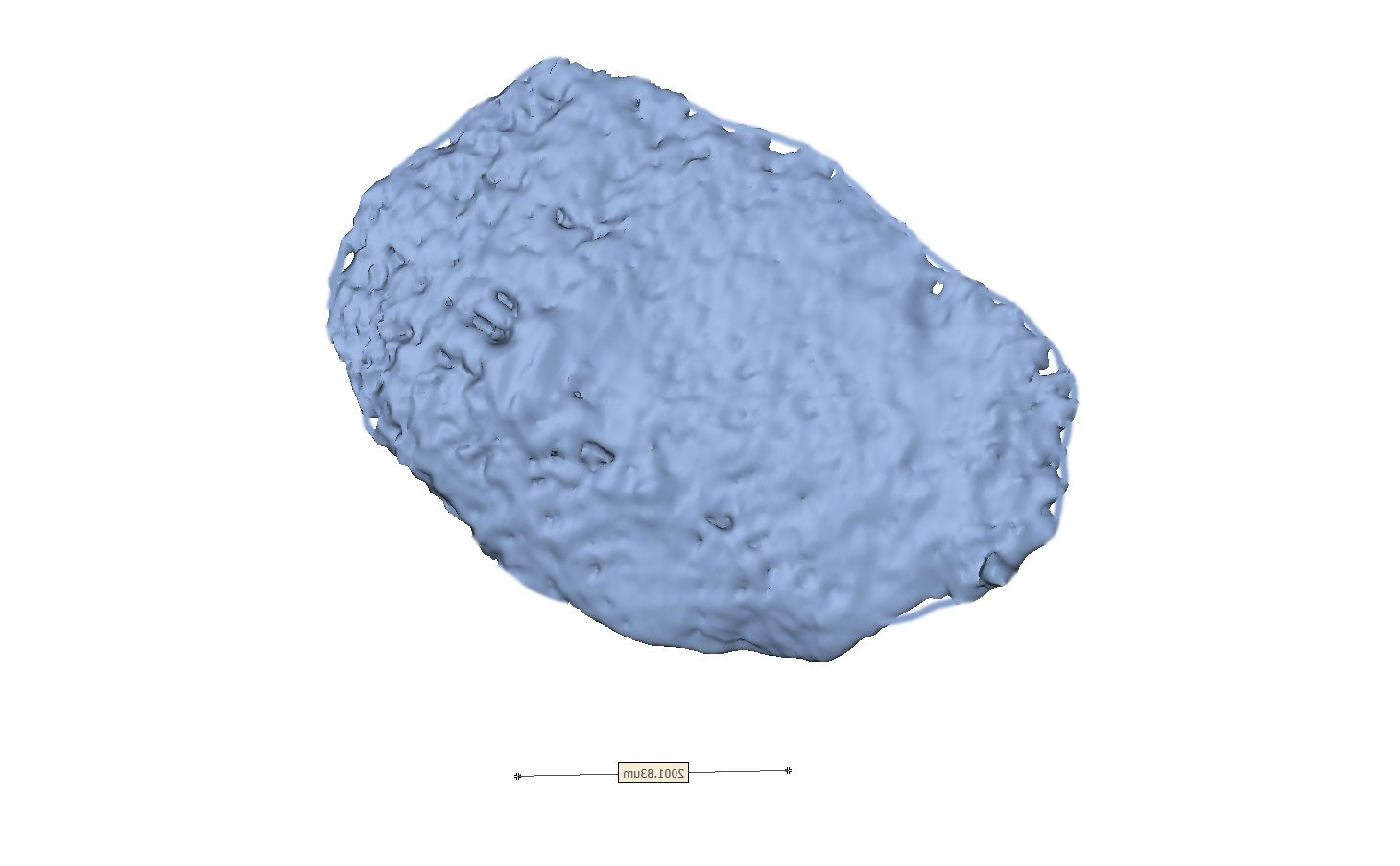

Supplement: Supplementary file 5 — Supplementary Data 2 [file 41467_2023_43557_MOESM5_ESM.zip › Supplementary Data 2/Supplementary Data 2 Raw data of Geometric Morphometric Analyses/12 Morphotypes/Morphotype 10/l5v11-.jpg]

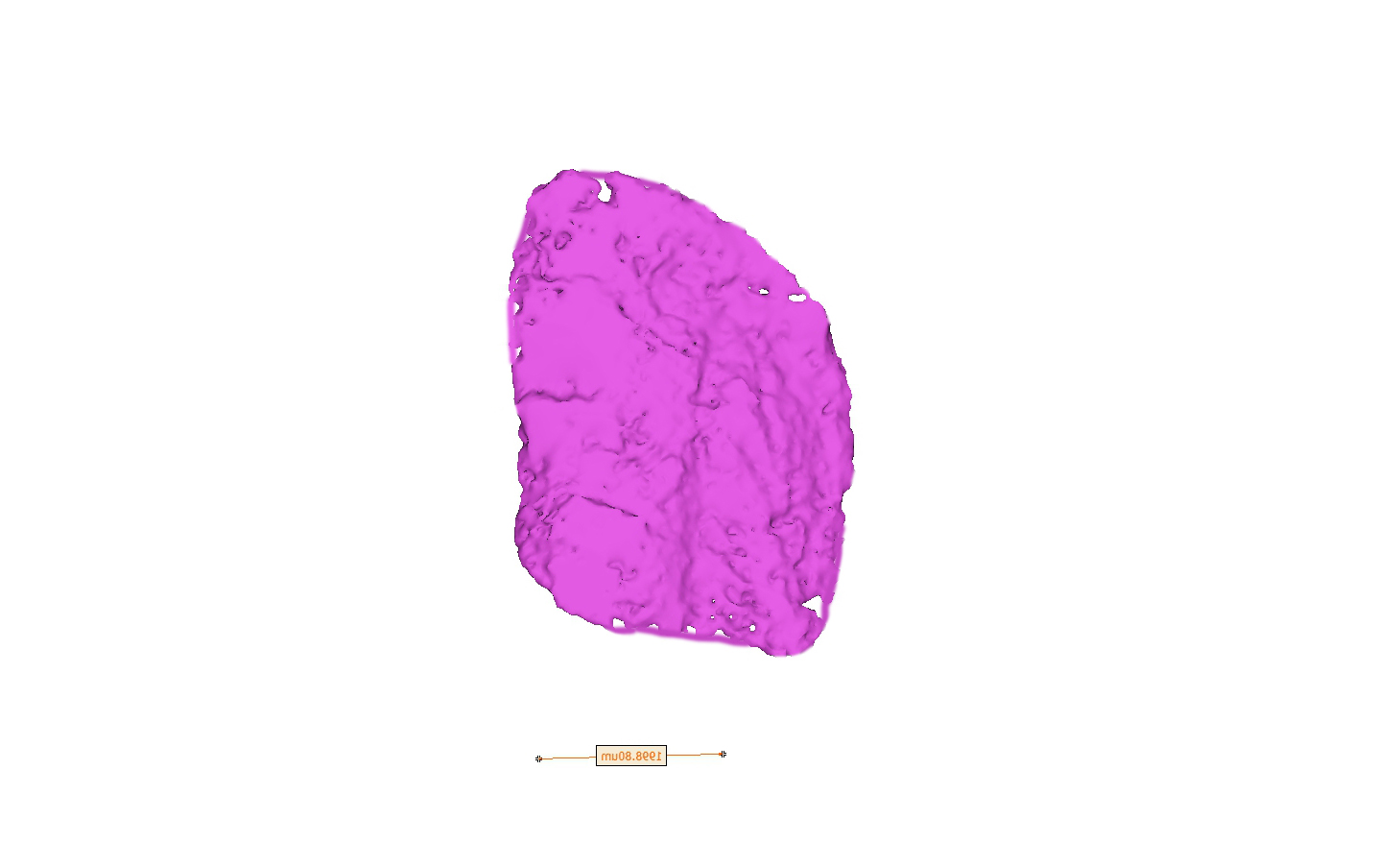

Supplement: Supplementary file 5 — Supplementary Data 2 [file 41467_2023_43557_MOESM5_ESM.zip › Supplementary Data 2/Supplementary Data 2 Raw data of Geometric Morphometric Analyses/12 Morphotypes/Morphotype 10/l6v02-.jpg]

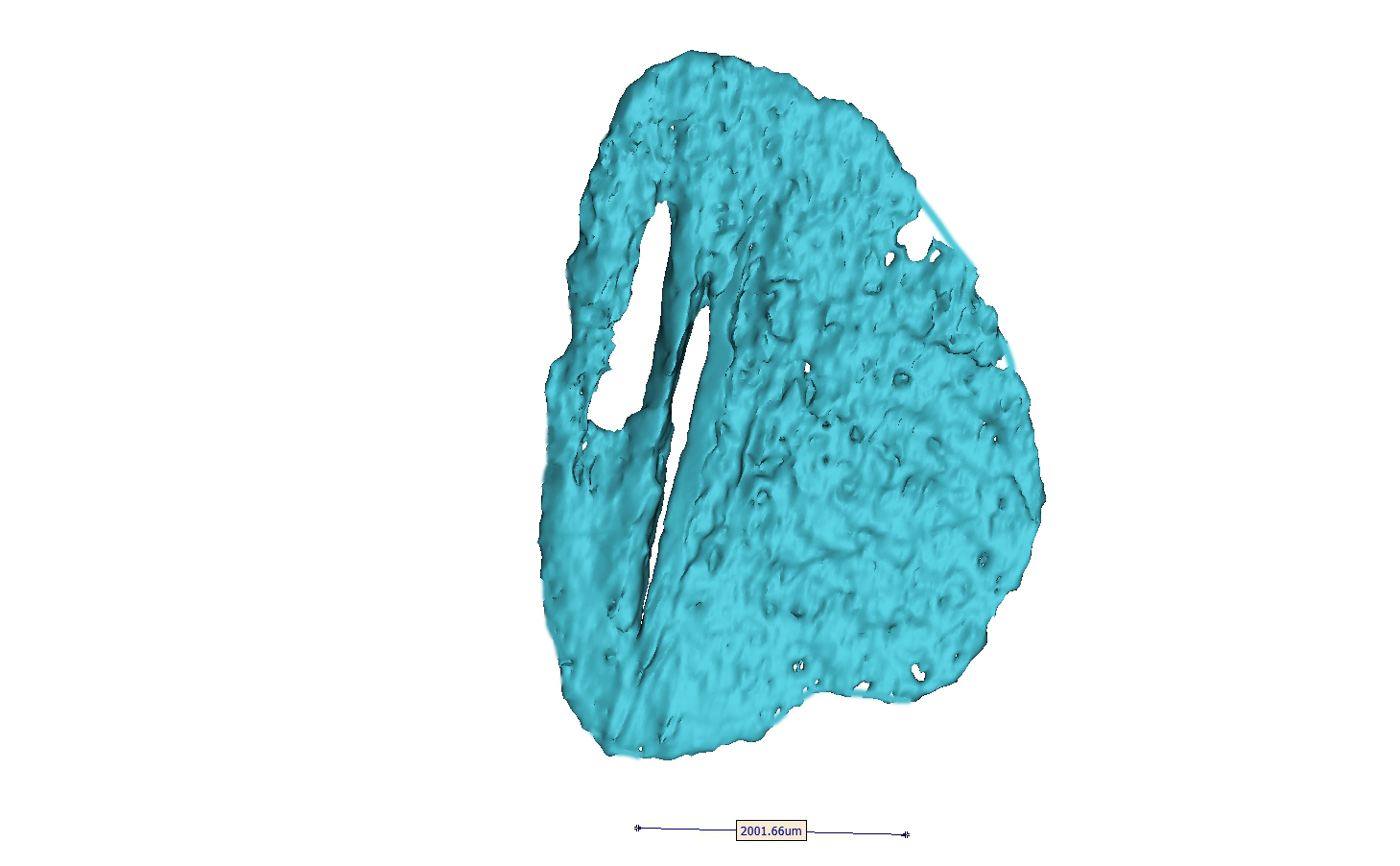

Supplement: Supplementary file 5 — Supplementary Data 2 [file 41467_2023_43557_MOESM5_ESM.zip › Supplementary Data 2/Supplementary Data 2 Raw data of Geometric Morphometric Analyses/12 Morphotypes/Morphotype 11/l5v01.jpg]

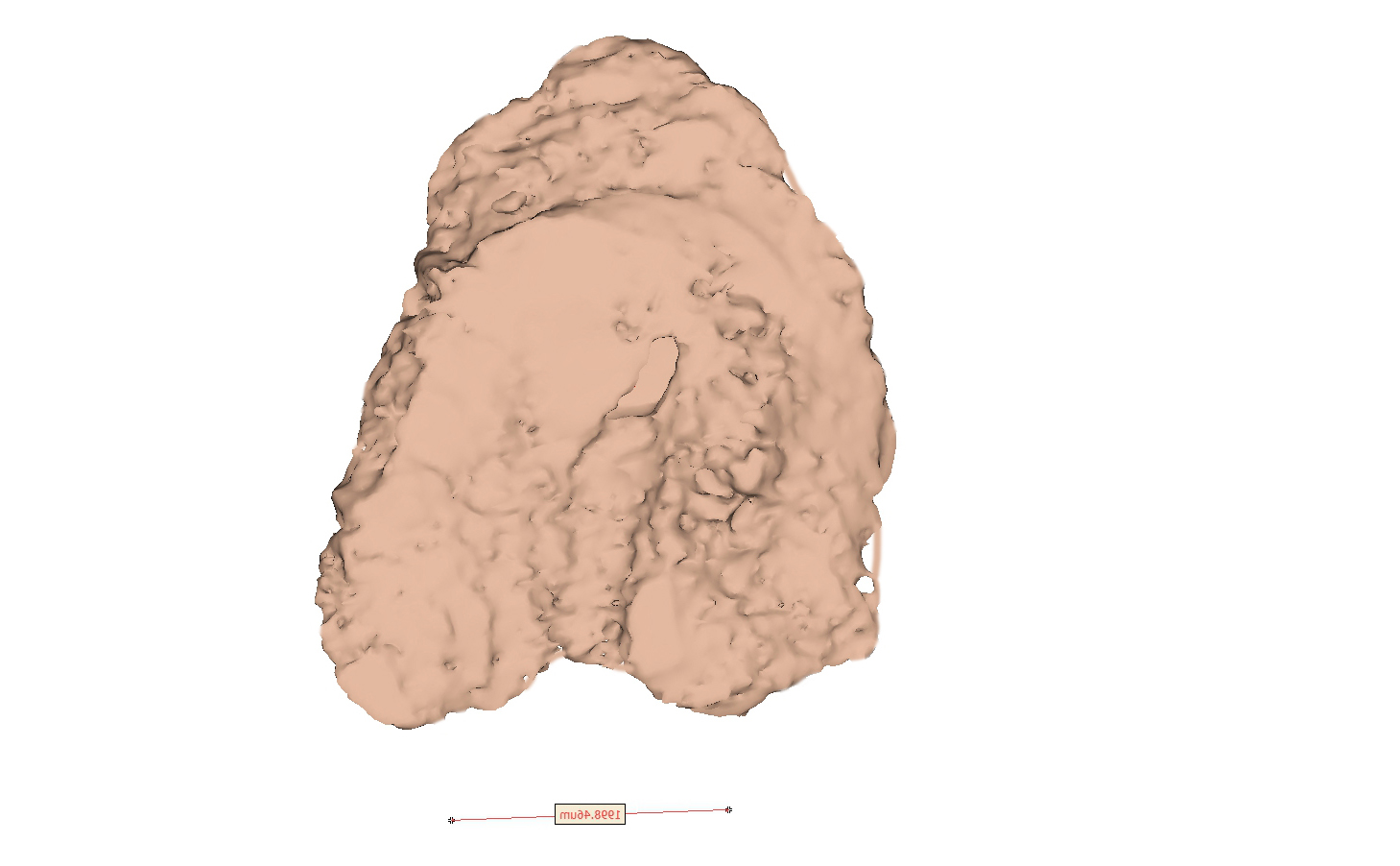

Supplement: Supplementary file 5 — Supplementary Data 2 [file 41467_2023_43557_MOESM5_ESM.zip › Supplementary Data 2/Supplementary Data 2 Raw data of Geometric Morphometric Analyses/12 Morphotypes/Morphotype 11/l5v02-.jpg]

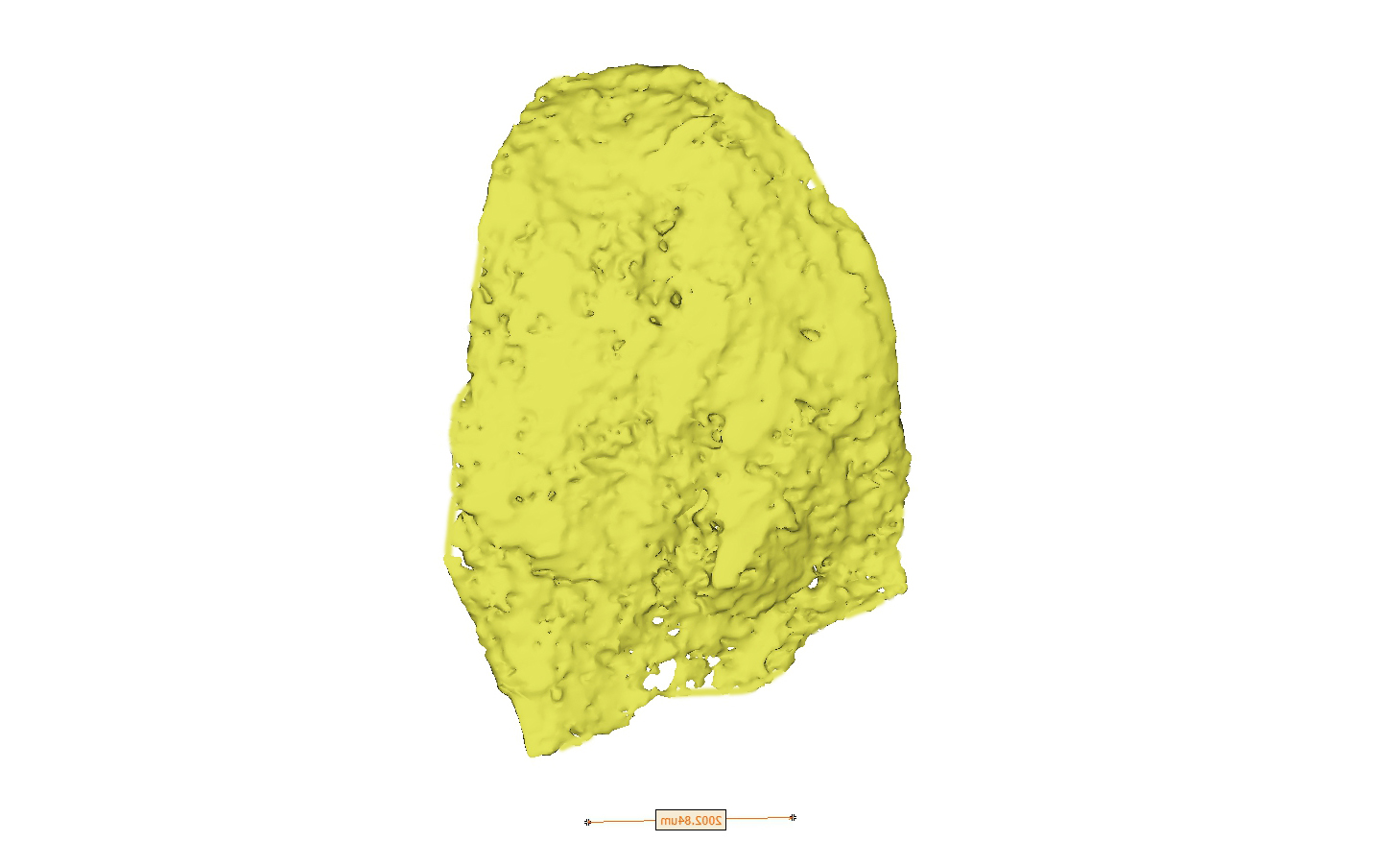

Supplement: Supplementary file 5 — Supplementary Data 2 [file 41467_2023_43557_MOESM5_ESM.zip › Supplementary Data 2/Supplementary Data 2 Raw data of Geometric Morphometric Analyses/12 Morphotypes/Morphotype 11/l5v03-.jpg]

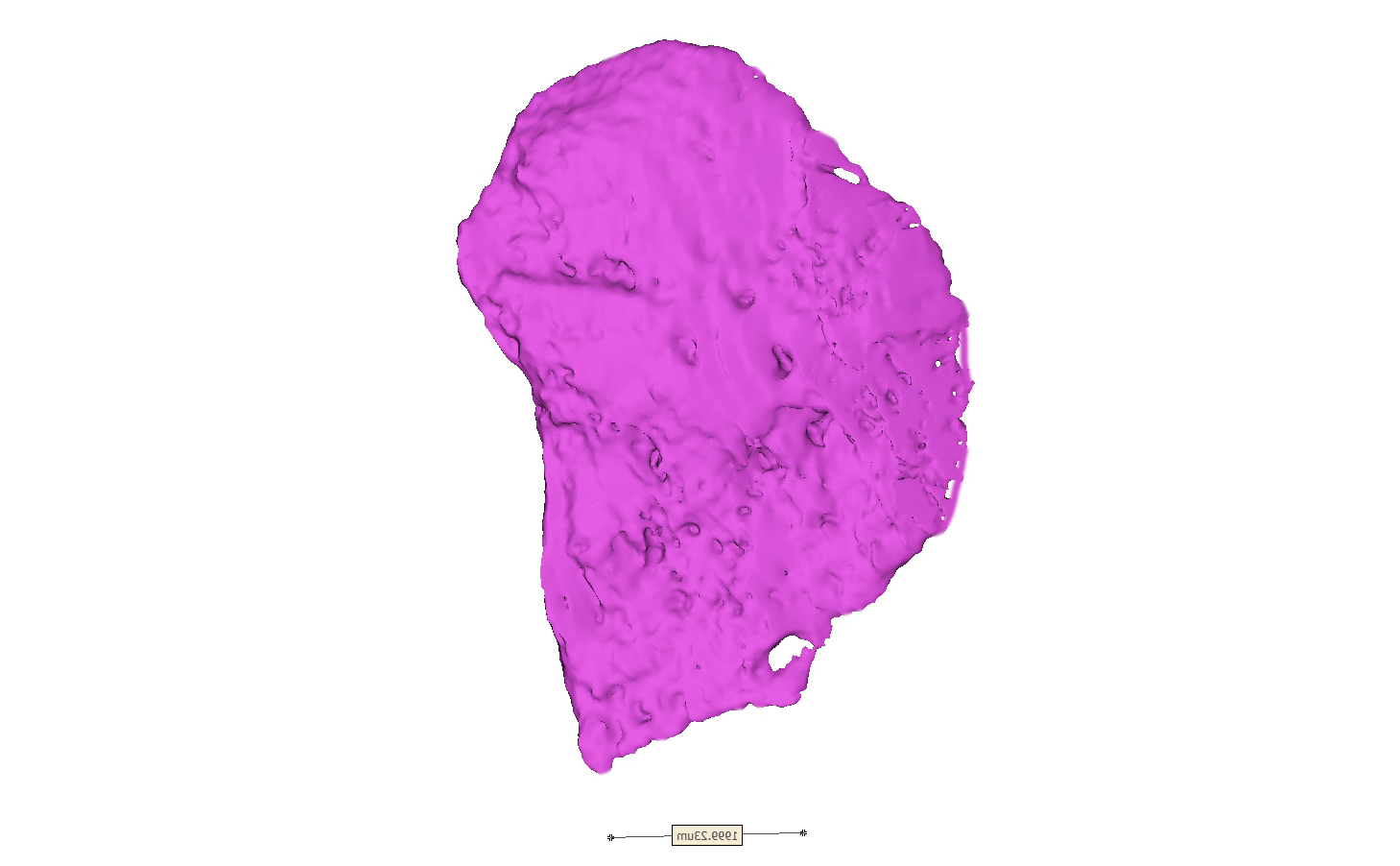

Supplement: Supplementary file 5 — Supplementary Data 2 [file 41467_2023_43557_MOESM5_ESM.zip › Supplementary Data 2/Supplementary Data 2 Raw data of Geometric Morphometric Analyses/12 Morphotypes/Morphotype 11/l5v06-.jpg]

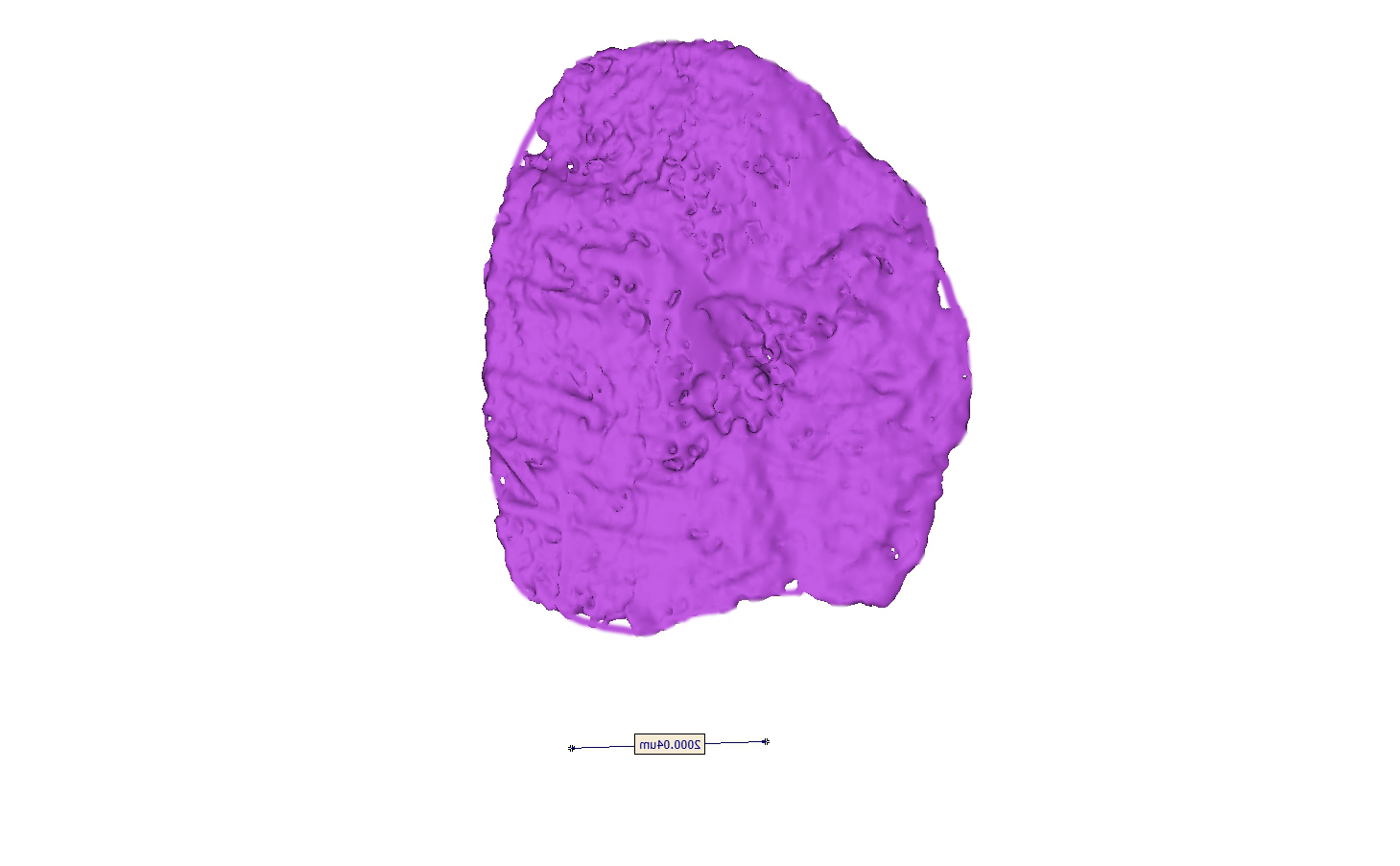

Supplement: Supplementary file 5 — Supplementary Data 2 [file 41467_2023_43557_MOESM5_ESM.zip › Supplementary Data 2/Supplementary Data 2 Raw data of Geometric Morphometric Analyses/12 Morphotypes/Morphotype 11/l5v07-.jpg]

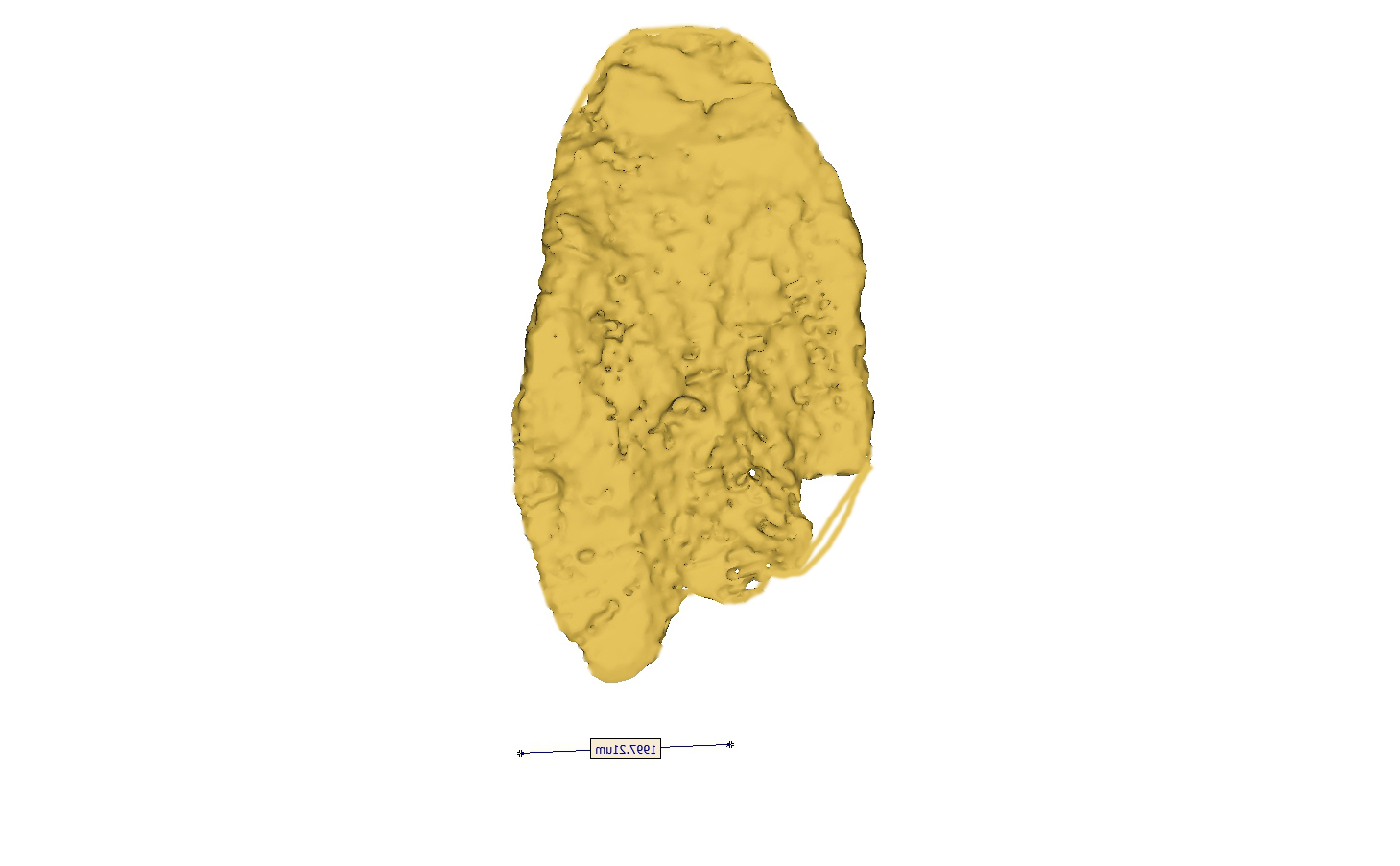

Supplement: Supplementary file 5 — Supplementary Data 2 [file 41467_2023_43557_MOESM5_ESM.zip › Supplementary Data 2/Supplementary Data 2 Raw data of Geometric Morphometric Analyses/12 Morphotypes/Morphotype 11/l6v01-.jpg]

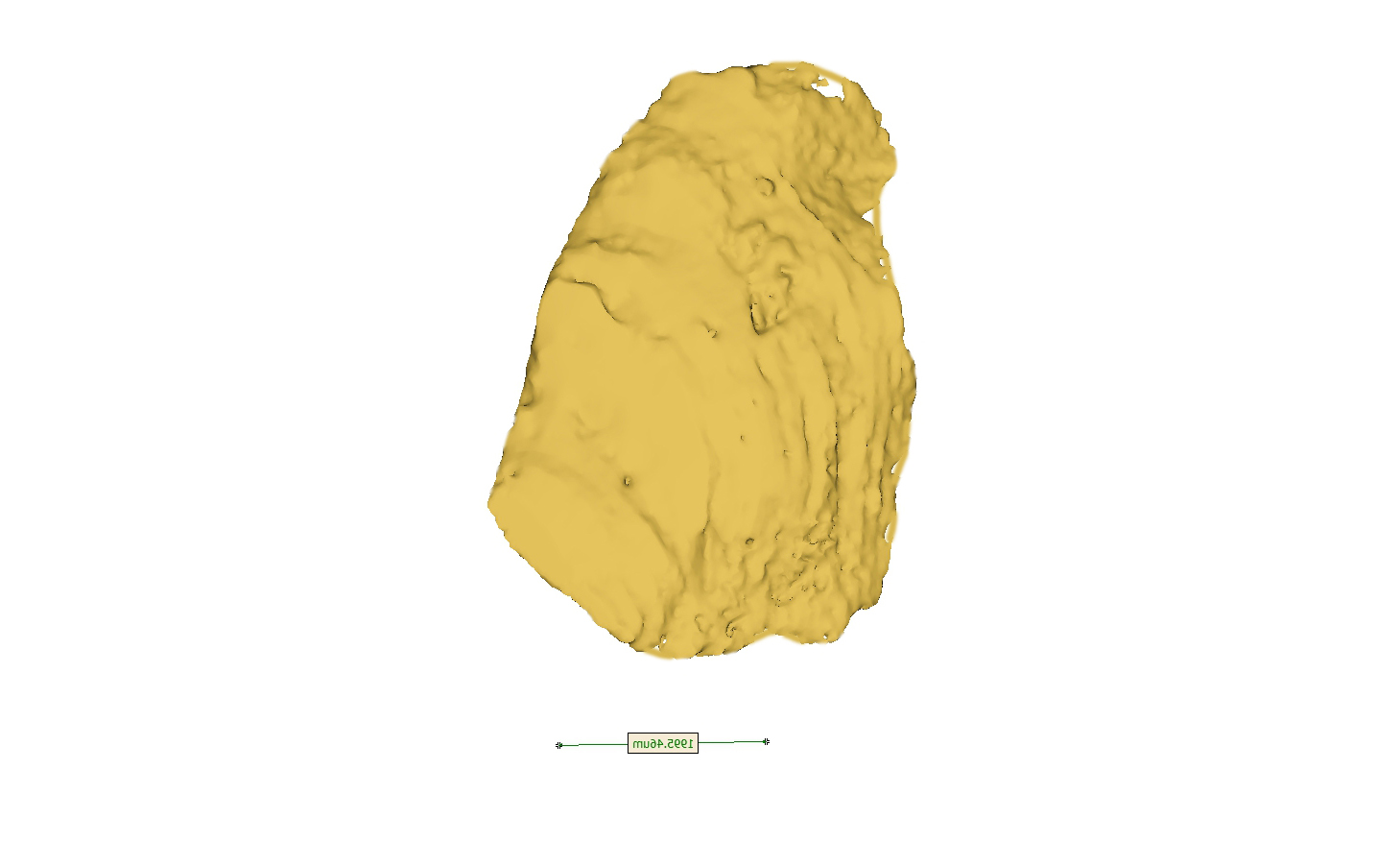

Supplement: Supplementary file 5 — Supplementary Data 2 [file 41467_2023_43557_MOESM5_ESM.zip › Supplementary Data 2/Supplementary Data 2 Raw data of Geometric Morphometric Analyses/12 Morphotypes/Morphotype 11/l6v03-.jpg]

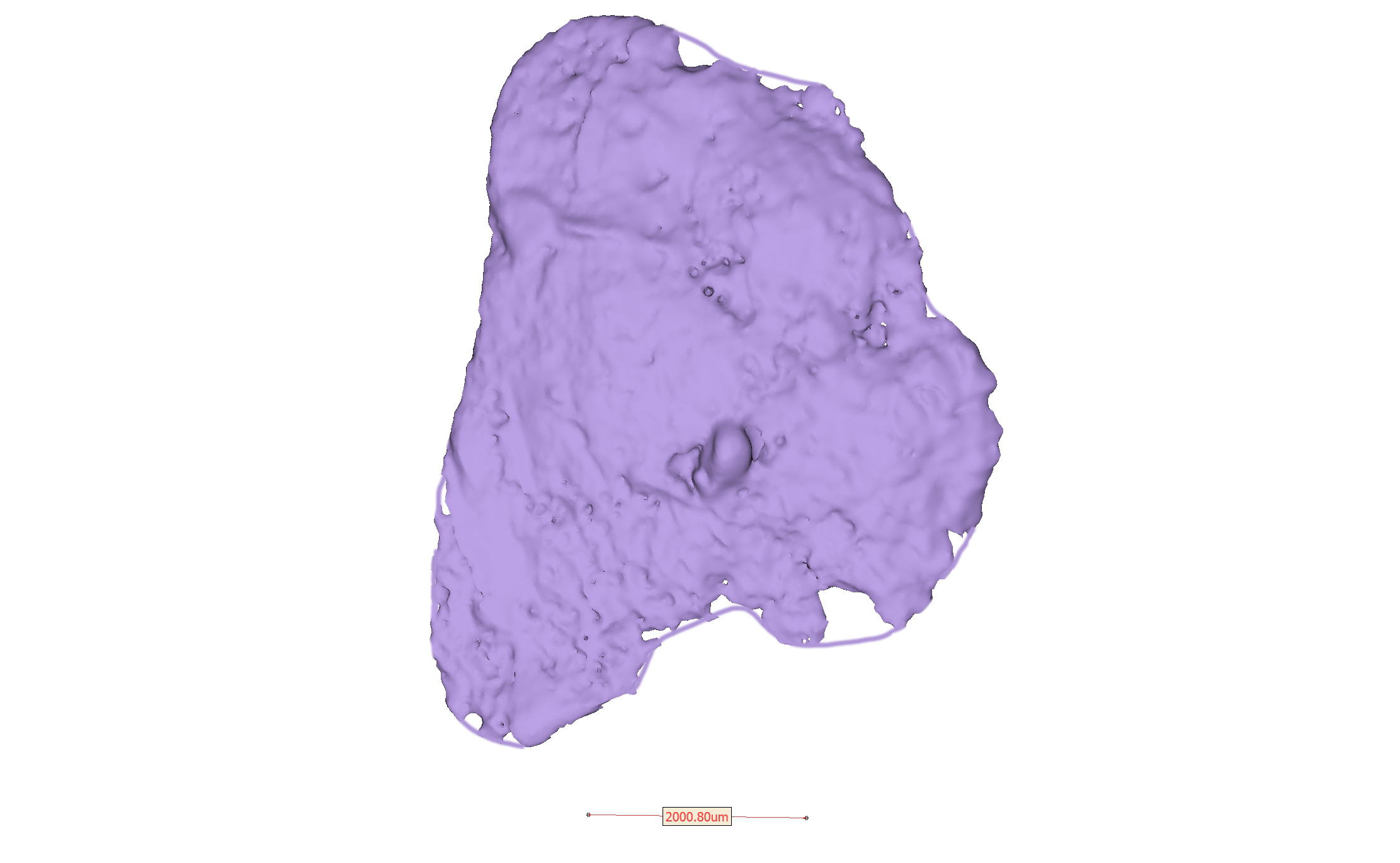

Supplement: Supplementary file 5 — Supplementary Data 2 [file 41467_2023_43557_MOESM5_ESM.zip › Supplementary Data 2/Supplementary Data 2 Raw data of Geometric Morphometric Analyses/12 Morphotypes/Morphotype 11/l6v06-.jpg]

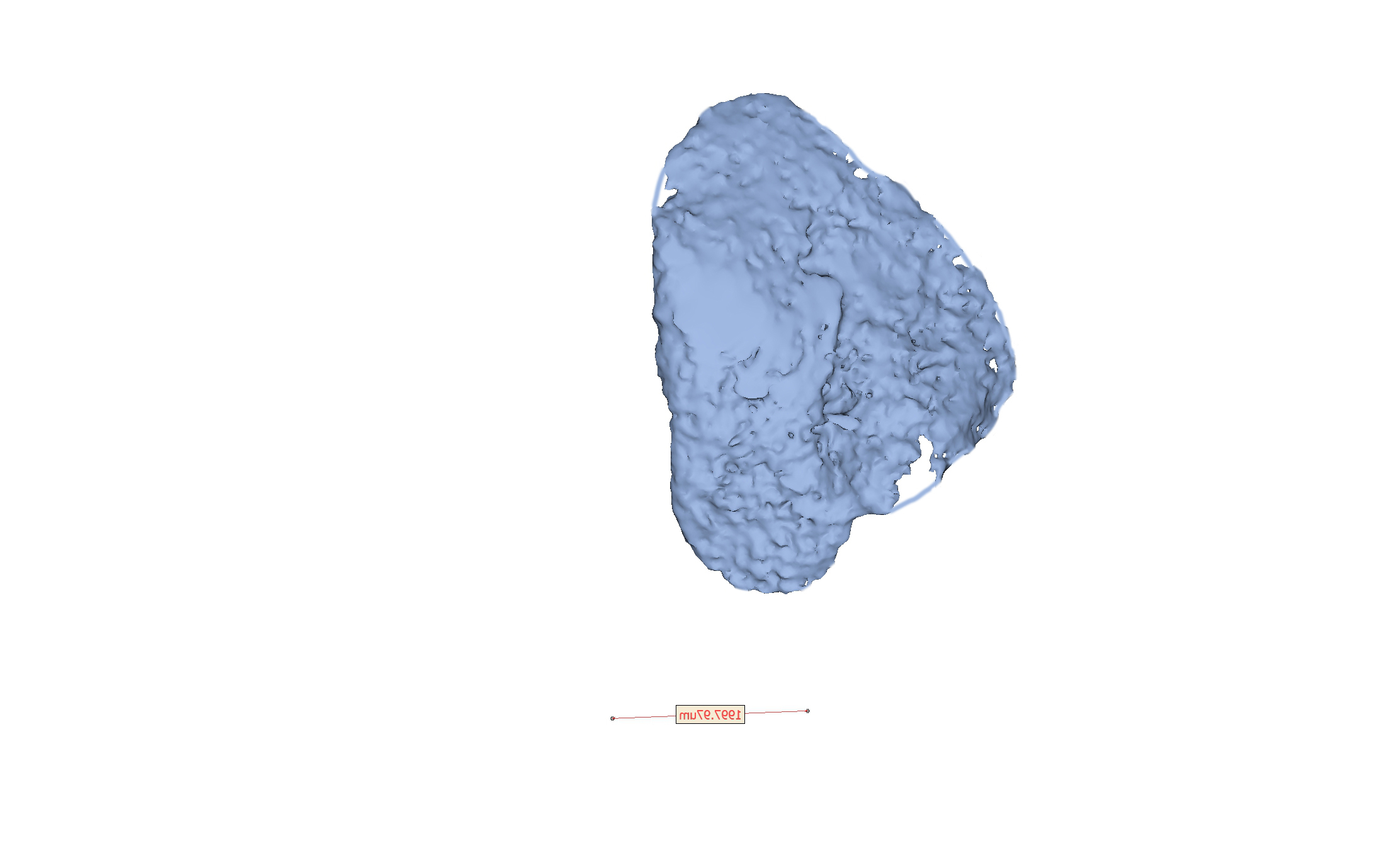

Supplement: Supplementary file 5 — Supplementary Data 2 [file 41467_2023_43557_MOESM5_ESM.zip › Supplementary Data 2/Supplementary Data 2 Raw data of Geometric Morphometric Analyses/12 Morphotypes/Morphotype 11/l7v03-.jpg]

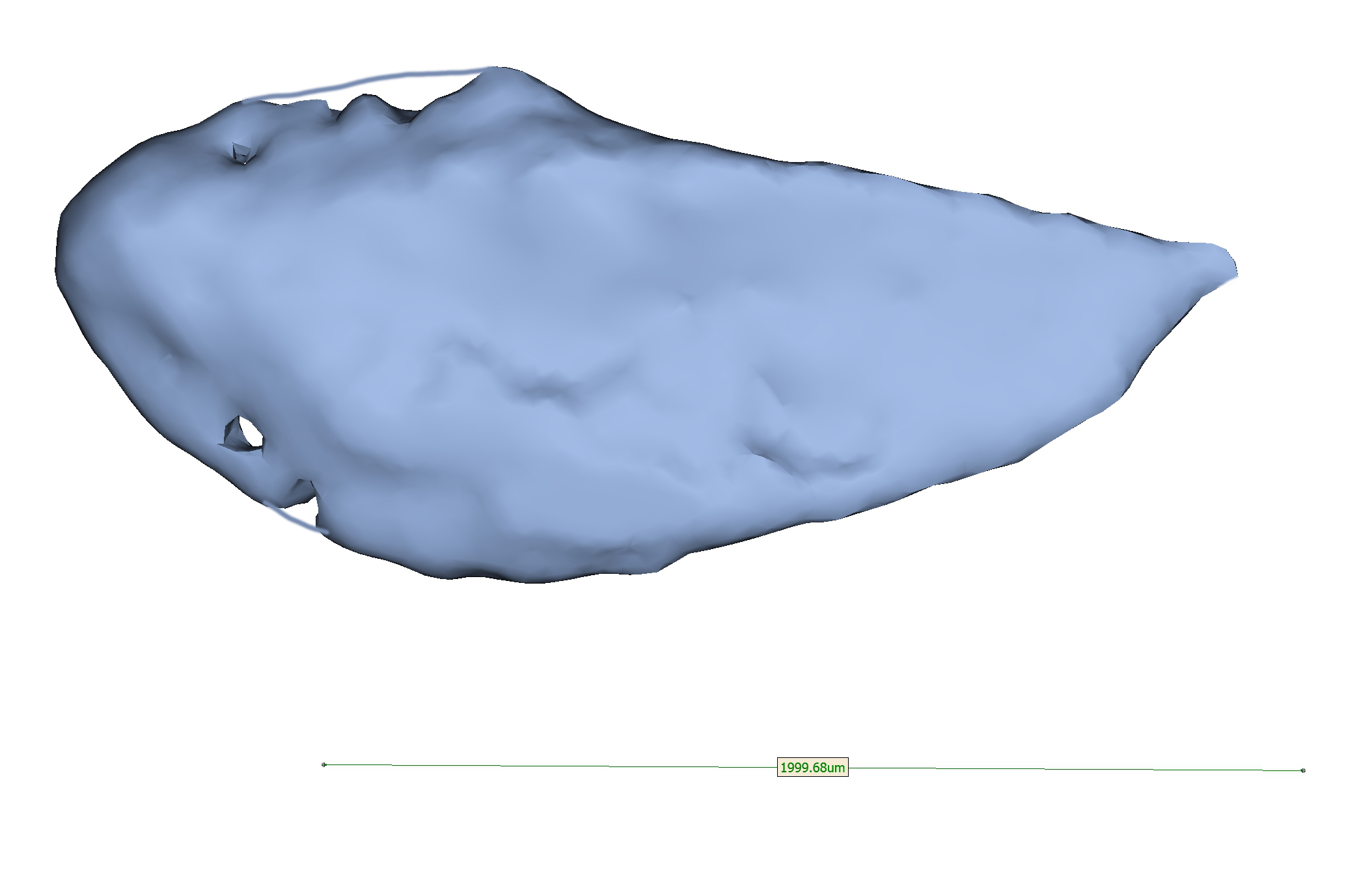

Supplement: Supplementary file 5 — Supplementary Data 2 [file 41467_2023_43557_MOESM5_ESM.zip › Supplementary Data 2/Supplementary Data 2 Raw data of Geometric Morphometric Analyses/12 Morphotypes/Morphotype 12/ts01l.jpg]

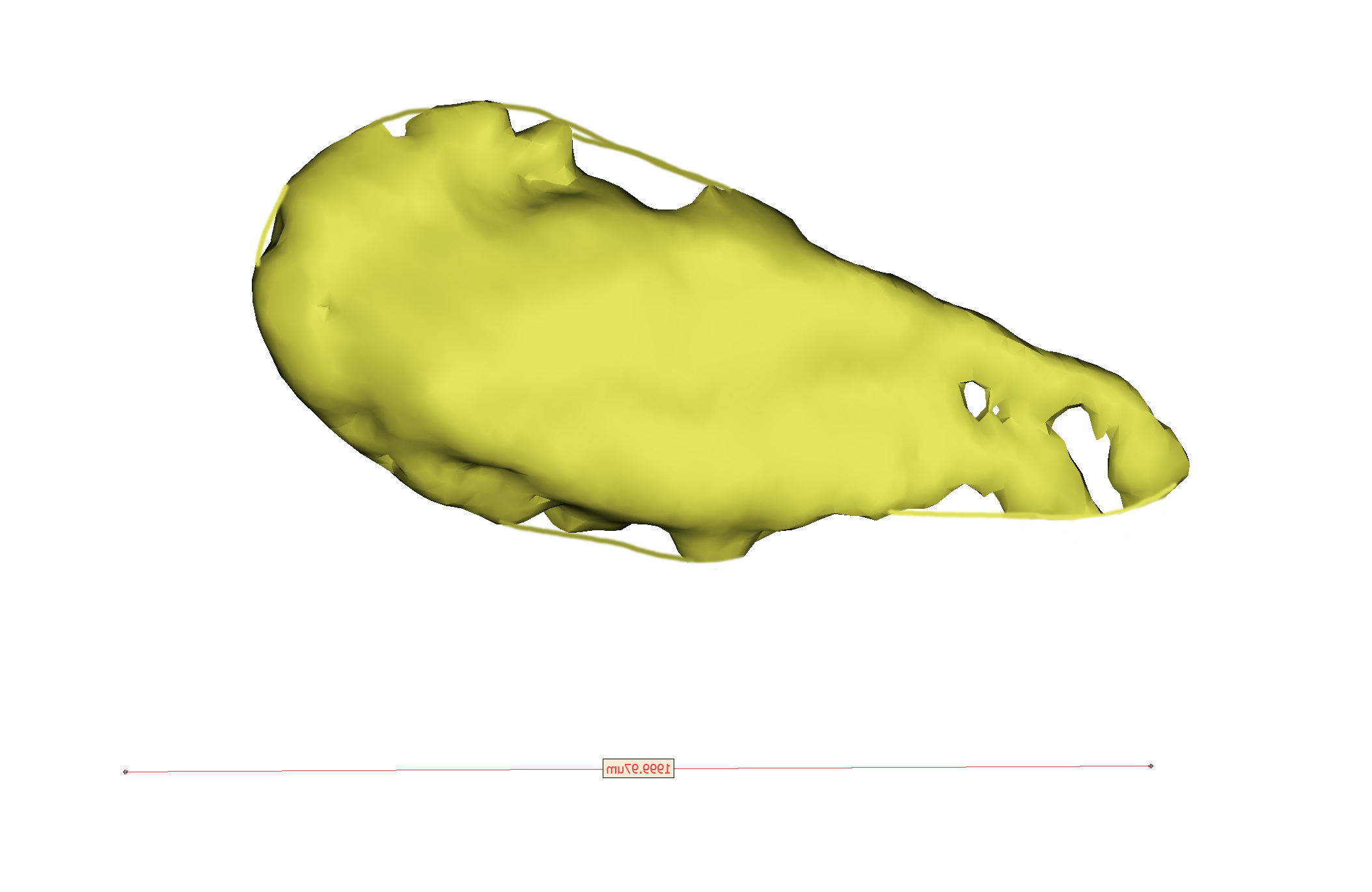

Supplement: Supplementary file 5 — Supplementary Data 2 [file 41467_2023_43557_MOESM5_ESM.zip › Supplementary Data 2/Supplementary Data 2 Raw data of Geometric Morphometric Analyses/12 Morphotypes/Morphotype 12/ts03r.jpg]

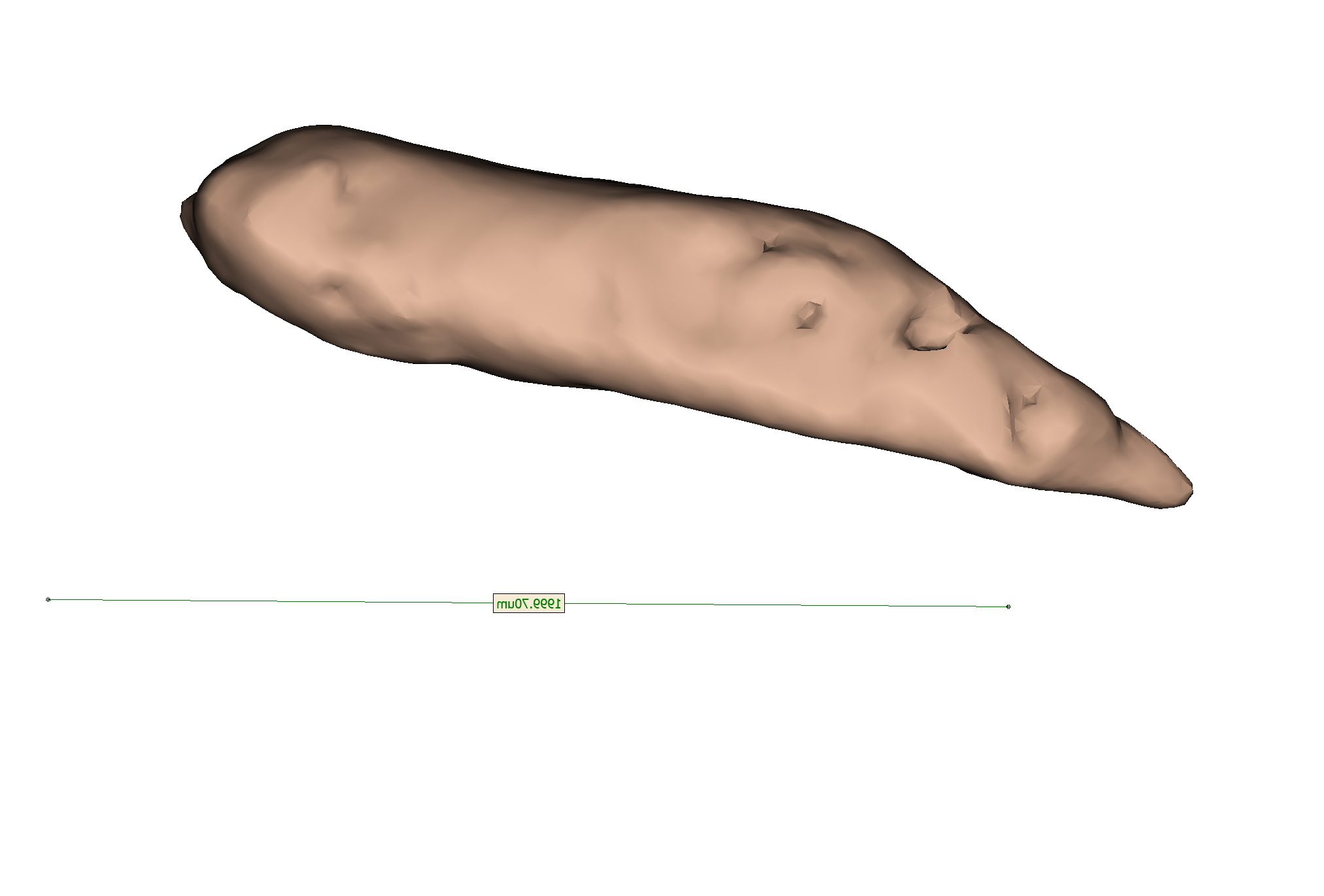

Supplement: Supplementary file 5 — Supplementary Data 2 [file 41467_2023_43557_MOESM5_ESM.zip › Supplementary Data 2/Supplementary Data 2 Raw data of Geometric Morphometric Analyses/12 Morphotypes/Morphotype 12/ts04r.jpg]

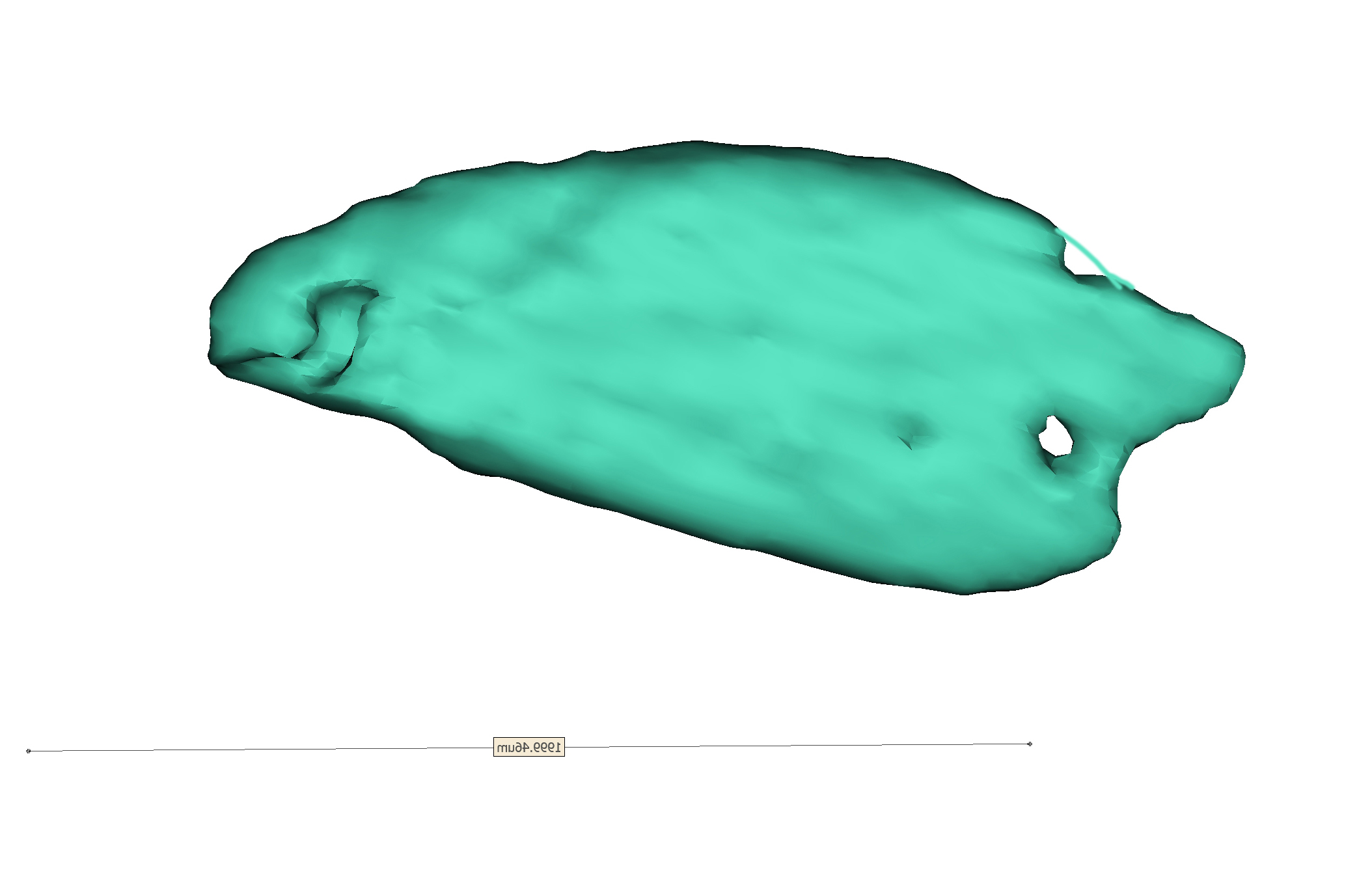

Supplement: Supplementary file 5 — Supplementary Data 2 [file 41467_2023_43557_MOESM5_ESM.zip › Supplementary Data 2/Supplementary Data 2 Raw data of Geometric Morphometric Analyses/12 Morphotypes/Morphotype 12/ts05r.jpg]

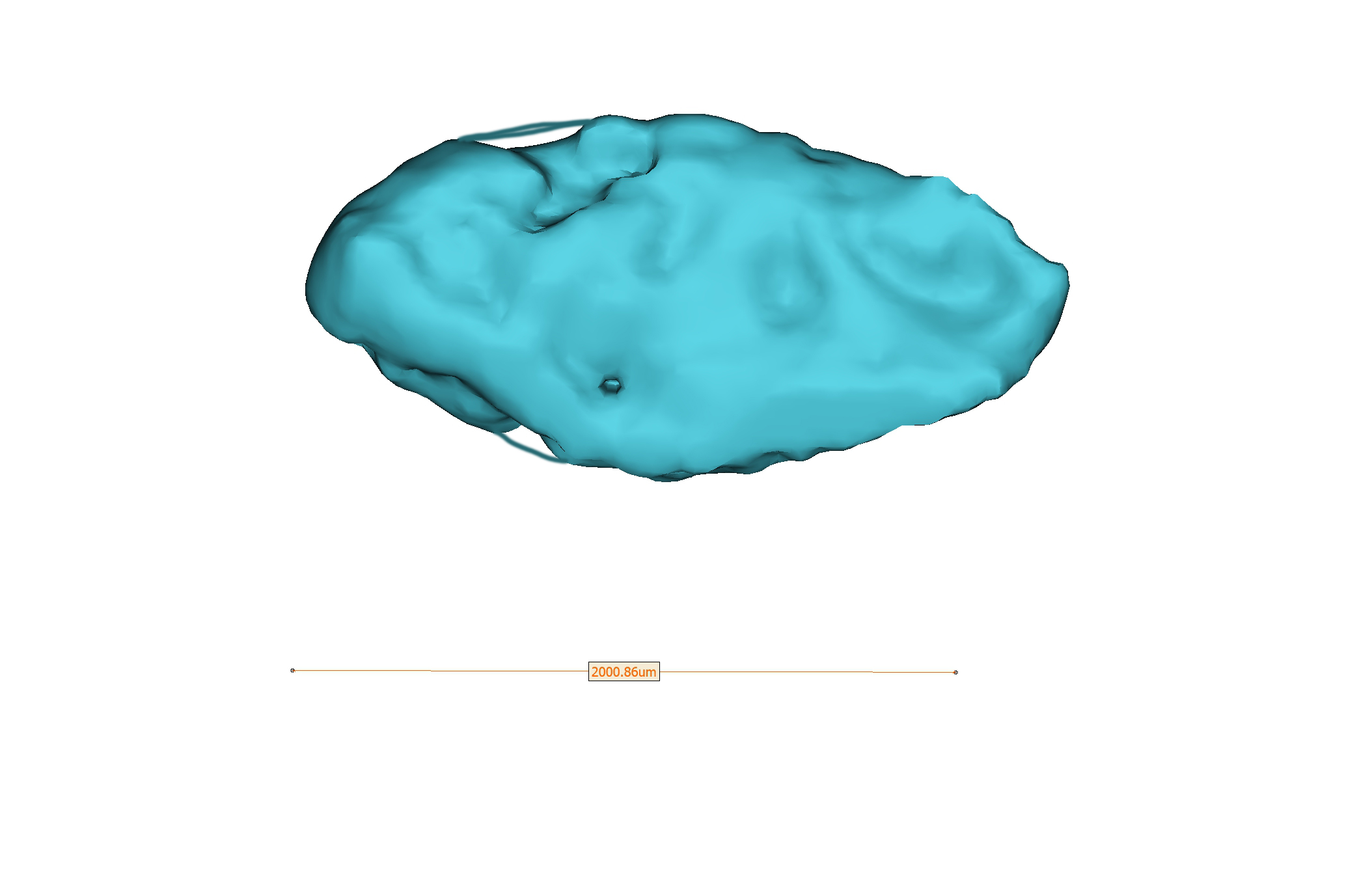

Supplement: Supplementary file 5 — Supplementary Data 2 [file 41467_2023_43557_MOESM5_ESM.zip › Supplementary Data 2/Supplementary Data 2 Raw data of Geometric Morphometric Analyses/12 Morphotypes/Morphotype 12/ts06l.jpg]

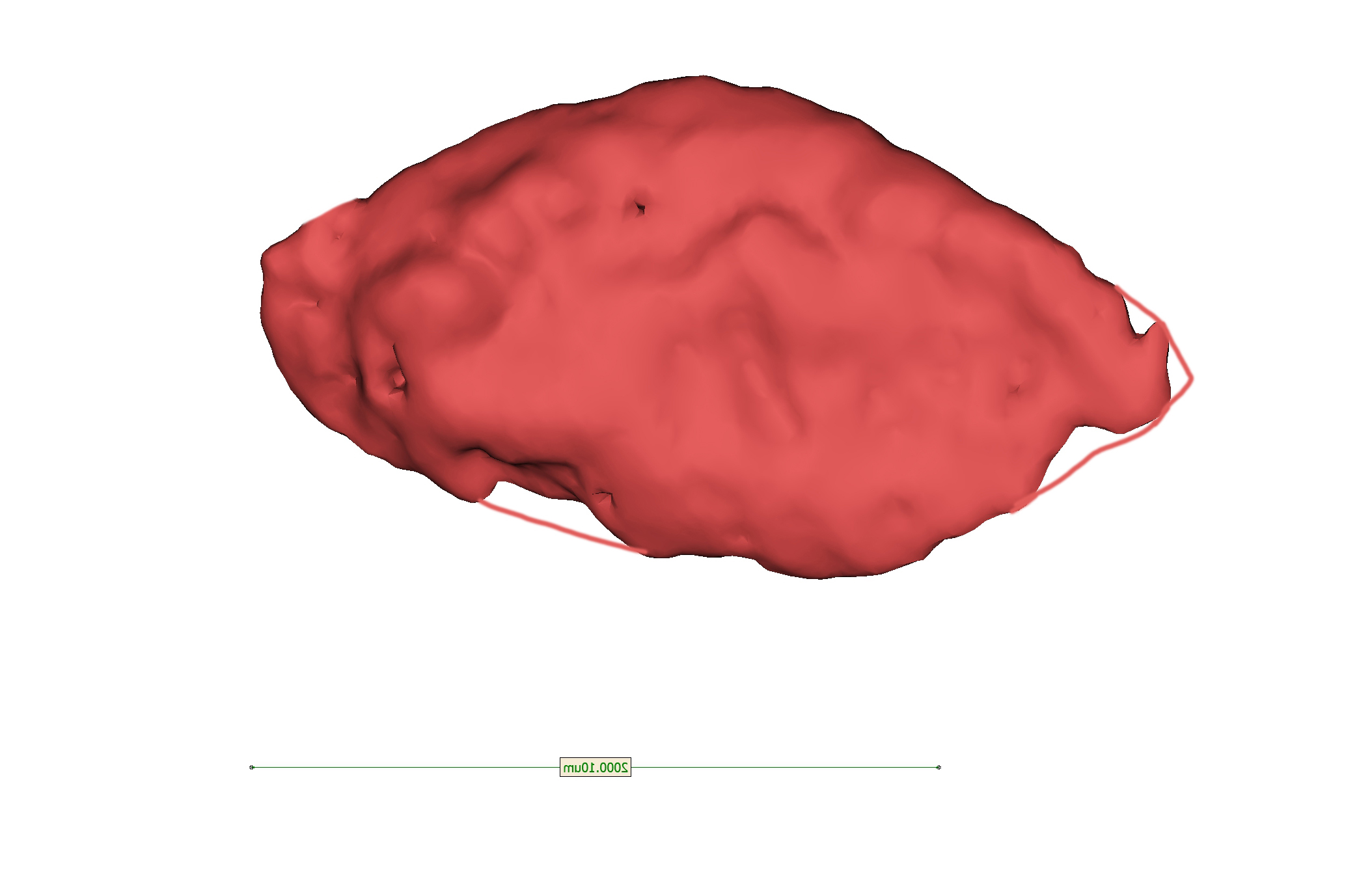

Supplement: Supplementary file 5 — Supplementary Data 2 [file 41467_2023_43557_MOESM5_ESM.zip › Supplementary Data 2/Supplementary Data 2 Raw data of Geometric Morphometric Analyses/12 Morphotypes/Morphotype 12/ts07r.jpg]

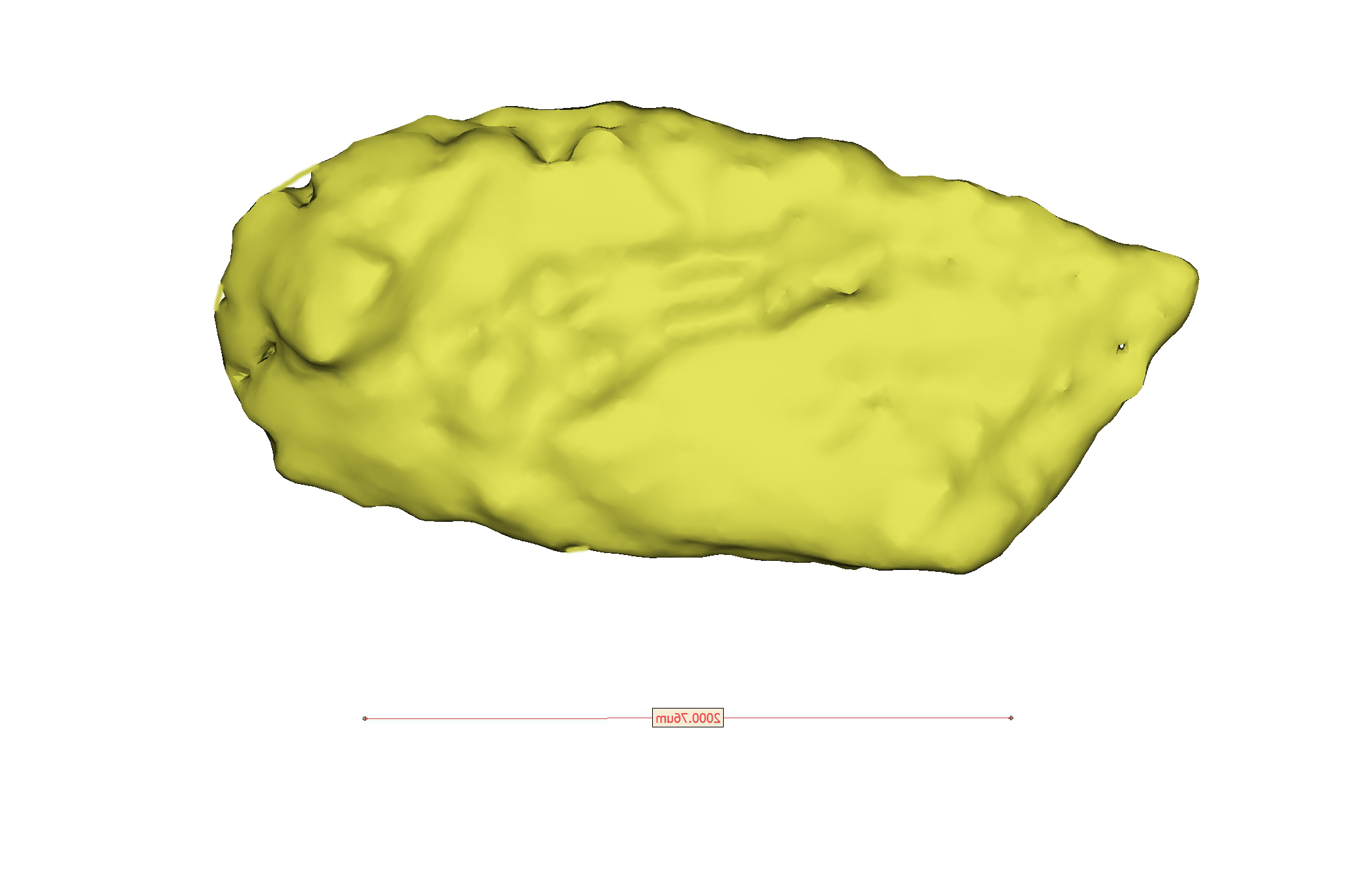

Supplement: Supplementary file 5 — Supplementary Data 2 [file 41467_2023_43557_MOESM5_ESM.zip › Supplementary Data 2/Supplementary Data 2 Raw data of Geometric Morphometric Analyses/12 Morphotypes/Morphotype 12/ts08r.jpg]

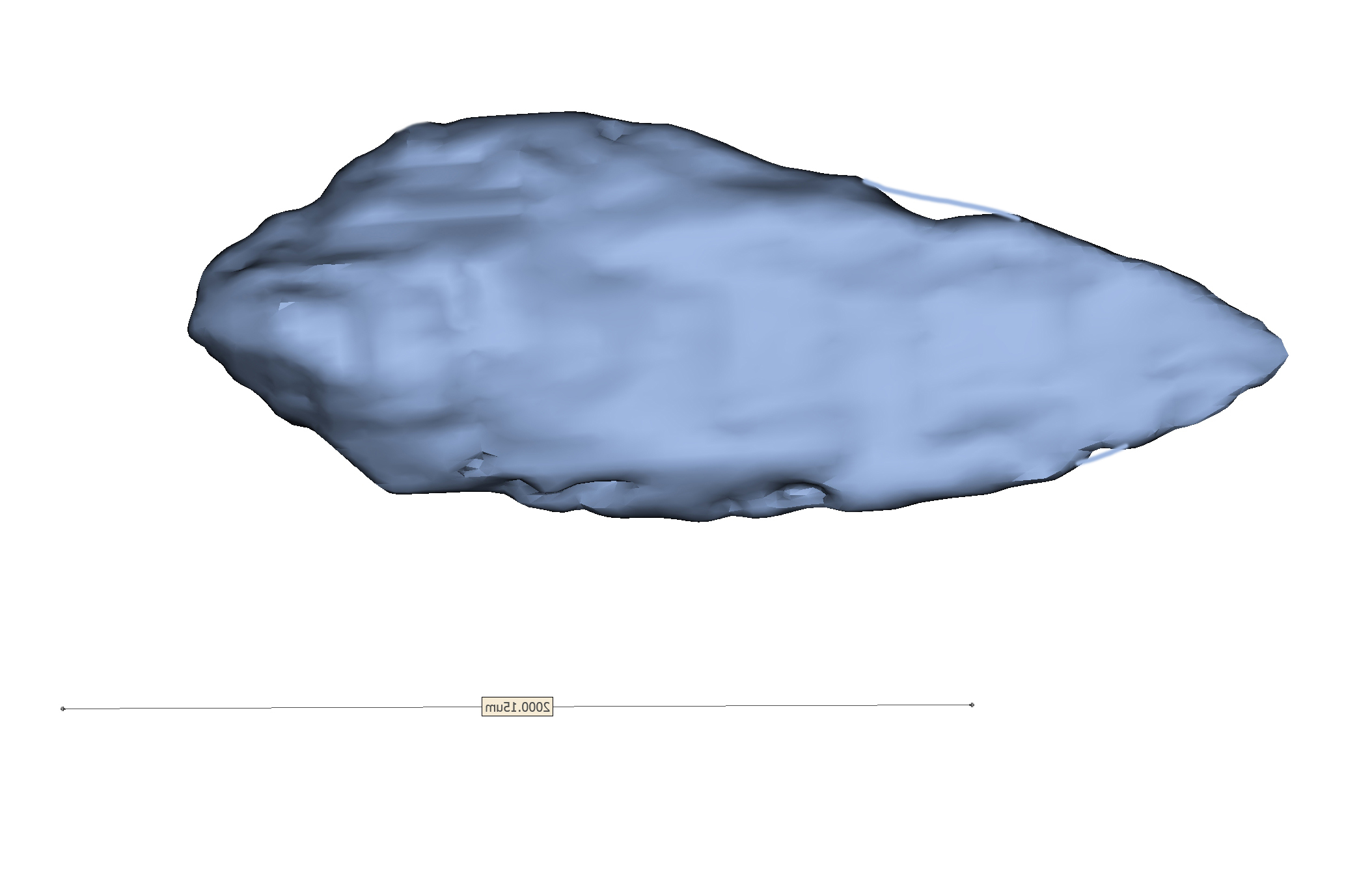

Supplement: Supplementary file 5 — Supplementary Data 2 [file 41467_2023_43557_MOESM5_ESM.zip › Supplementary Data 2/Supplementary Data 2 Raw data of Geometric Morphometric Analyses/12 Morphotypes/Morphotype 12/ts09r.jpg]

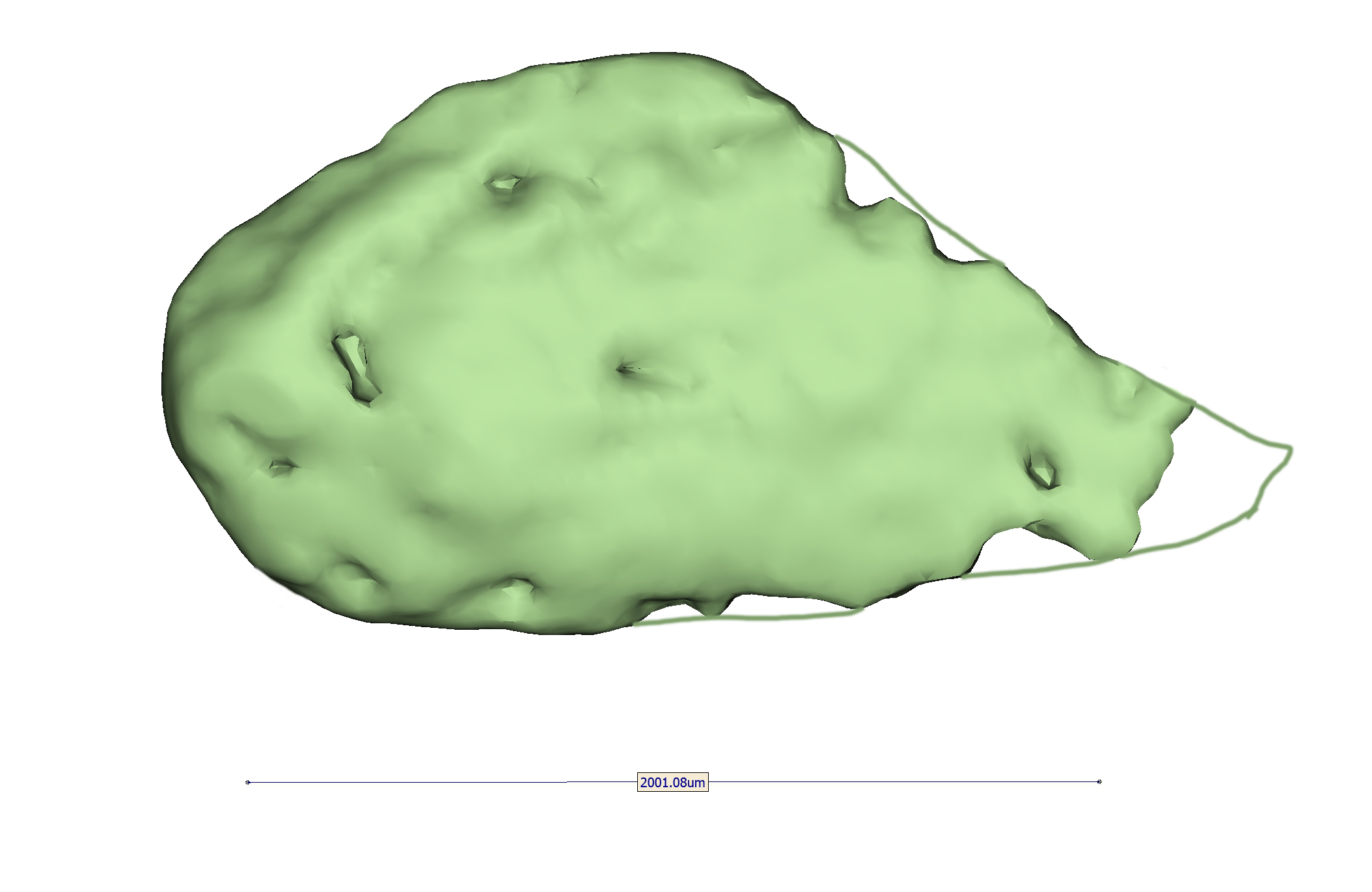

Supplement: Supplementary file 5 — Supplementary Data 2 [file 41467_2023_43557_MOESM5_ESM.zip › Supplementary Data 2/Supplementary Data 2 Raw data of Geometric Morphometric Analyses/12 Morphotypes/Morphotype 12/ts10l.jpg]

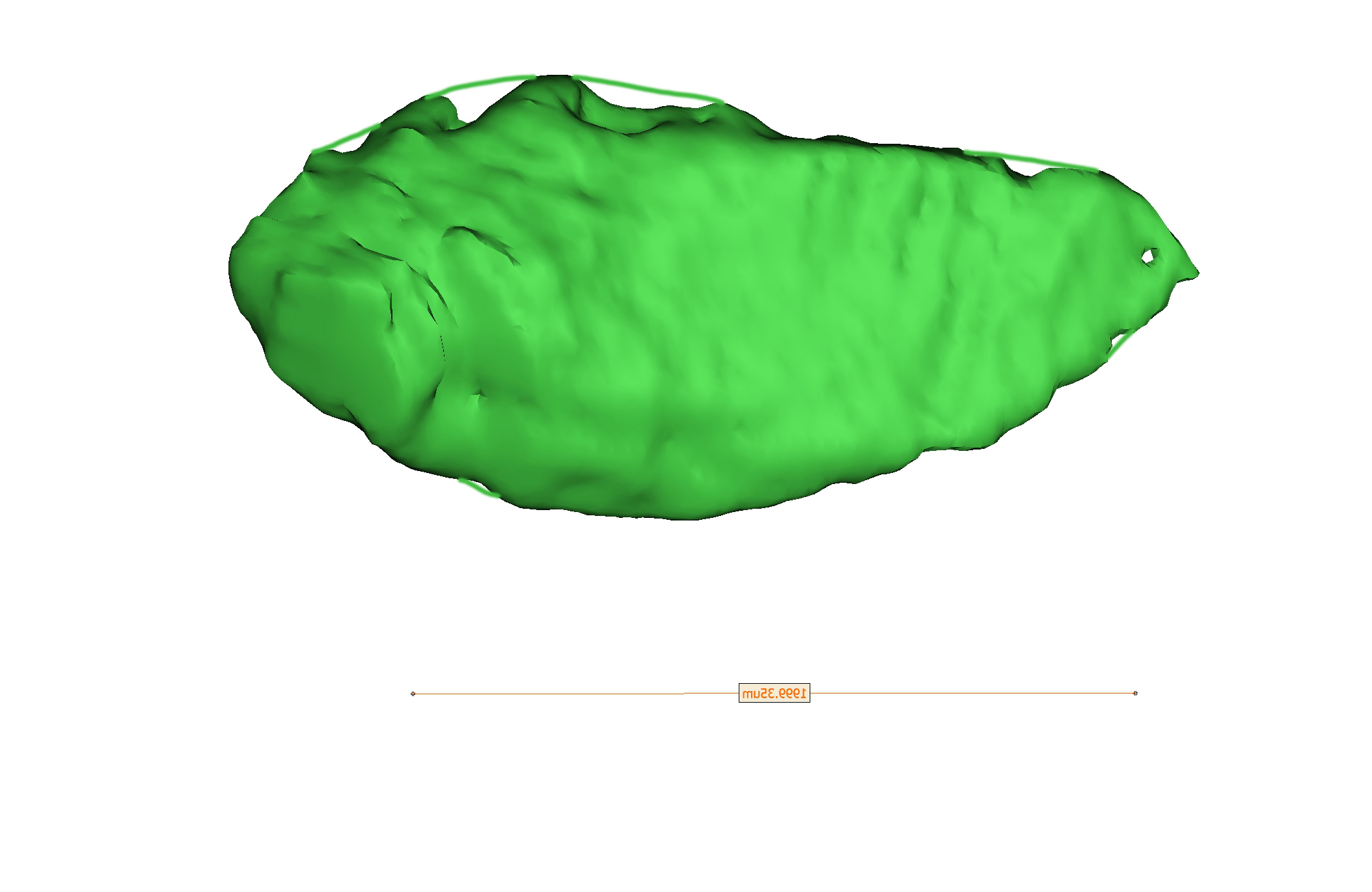

Supplement: Supplementary file 5 — Supplementary Data 2 [file 41467_2023_43557_MOESM5_ESM.zip › Supplementary Data 2/Supplementary Data 2 Raw data of Geometric Morphometric Analyses/12 Morphotypes/Morphotype 12/ts11r.jpg]

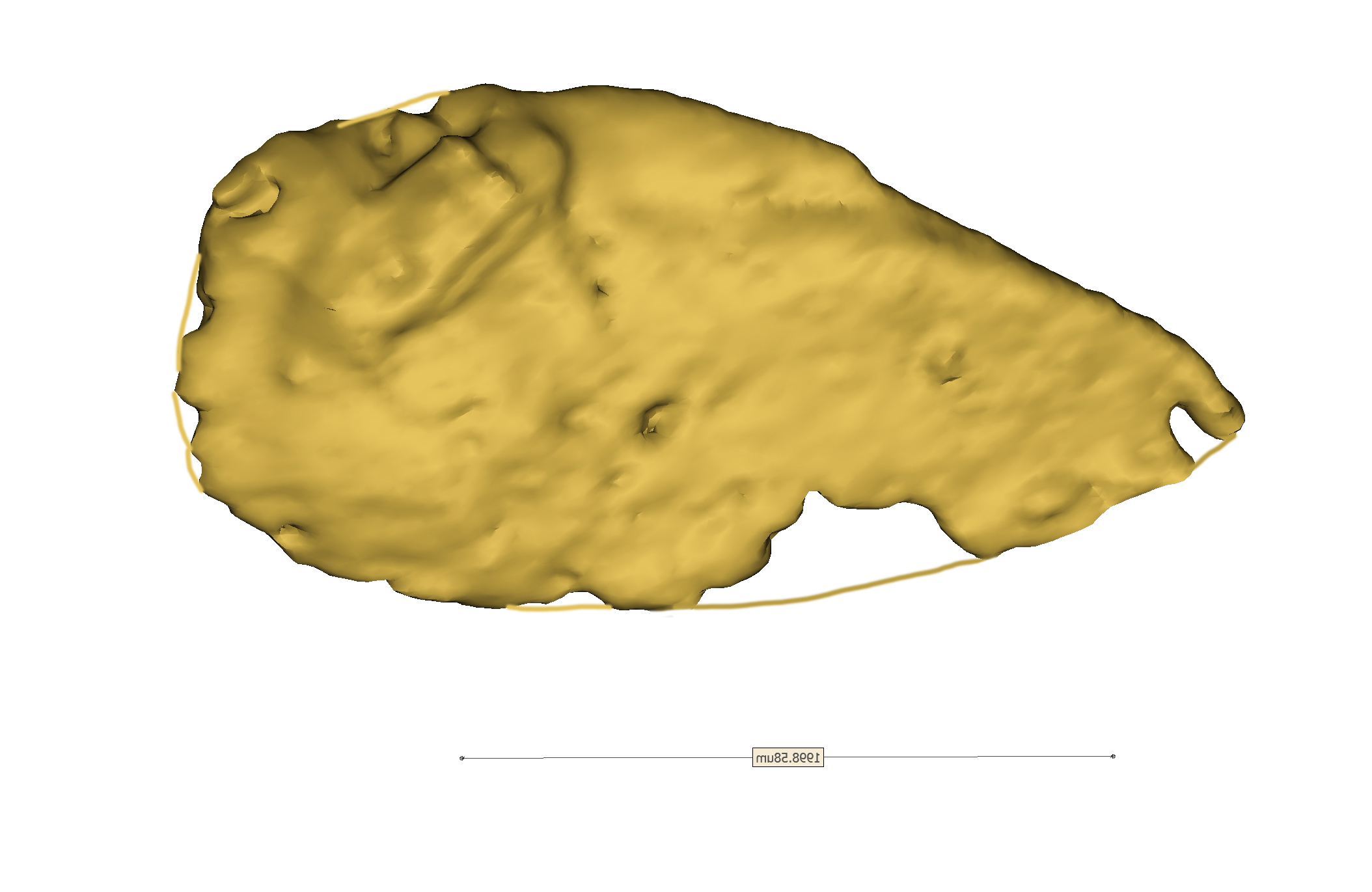

Supplement: Supplementary file 5 — Supplementary Data 2 [file 41467_2023_43557_MOESM5_ESM.zip › Supplementary Data 2/Supplementary Data 2 Raw data of Geometric Morphometric Analyses/12 Morphotypes/Morphotype 12/ts14r.jpg]

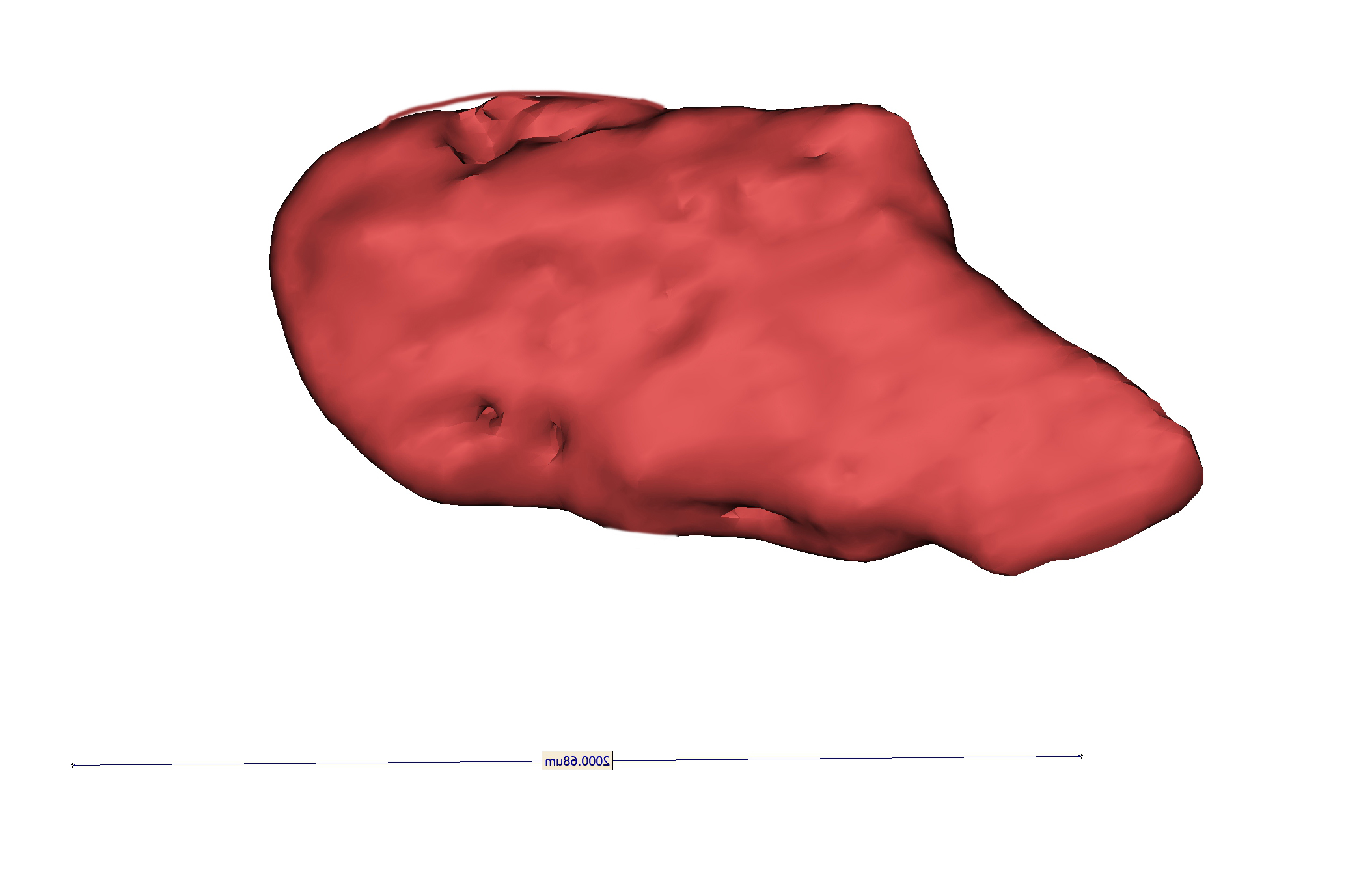

Supplement: Supplementary file 5 — Supplementary Data 2 [file 41467_2023_43557_MOESM5_ESM.zip › Supplementary Data 2/Supplementary Data 2 Raw data of Geometric Morphometric Analyses/12 Morphotypes/Morphotype 12/ts15r.jpg]

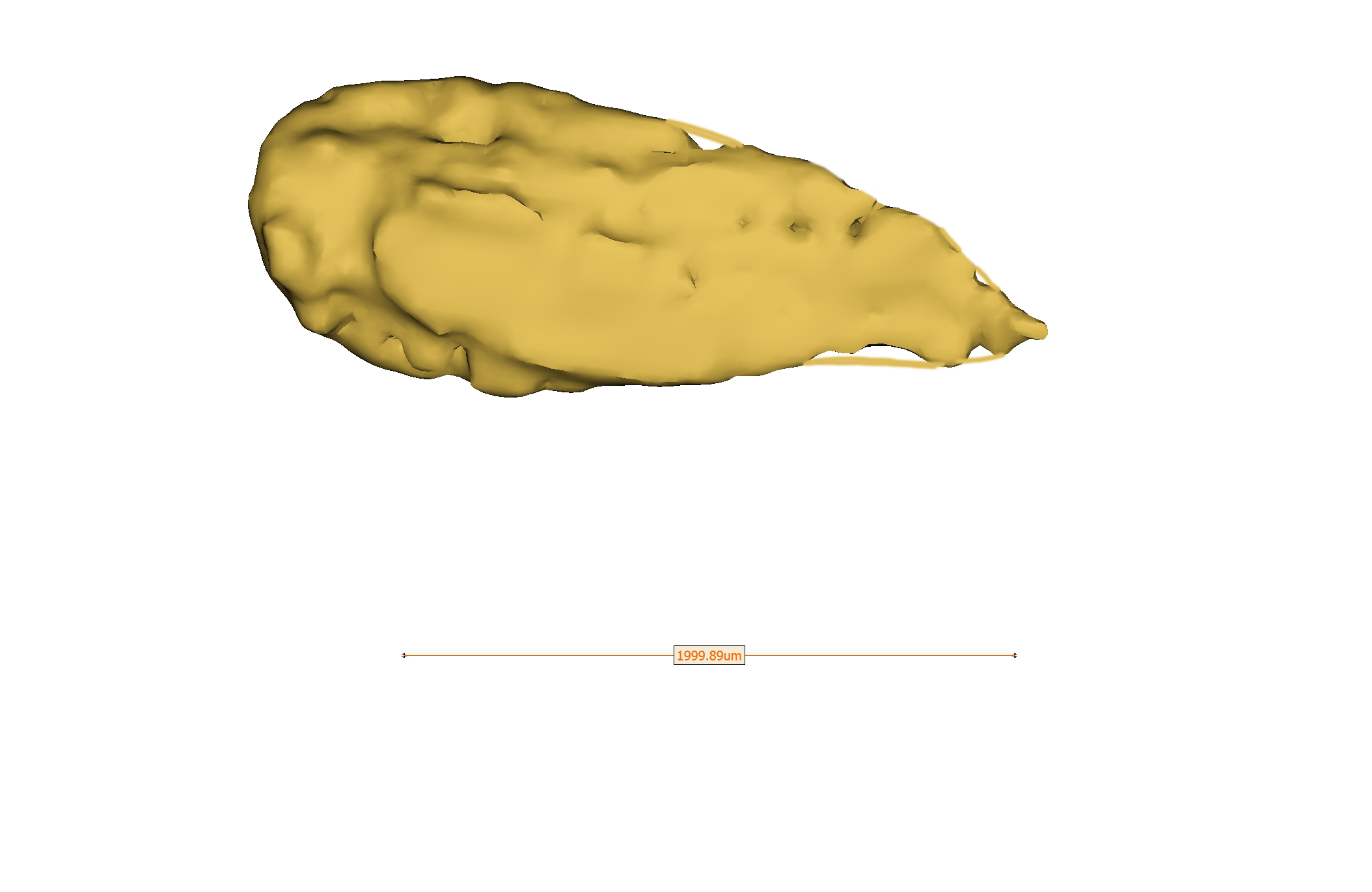

Supplement: Supplementary file 5 — Supplementary Data 2 [file 41467_2023_43557_MOESM5_ESM.zip › Supplementary Data 2/Supplementary Data 2 Raw data of Geometric Morphometric Analyses/12 Morphotypes/Morphotype 12/ts16l.jpg]

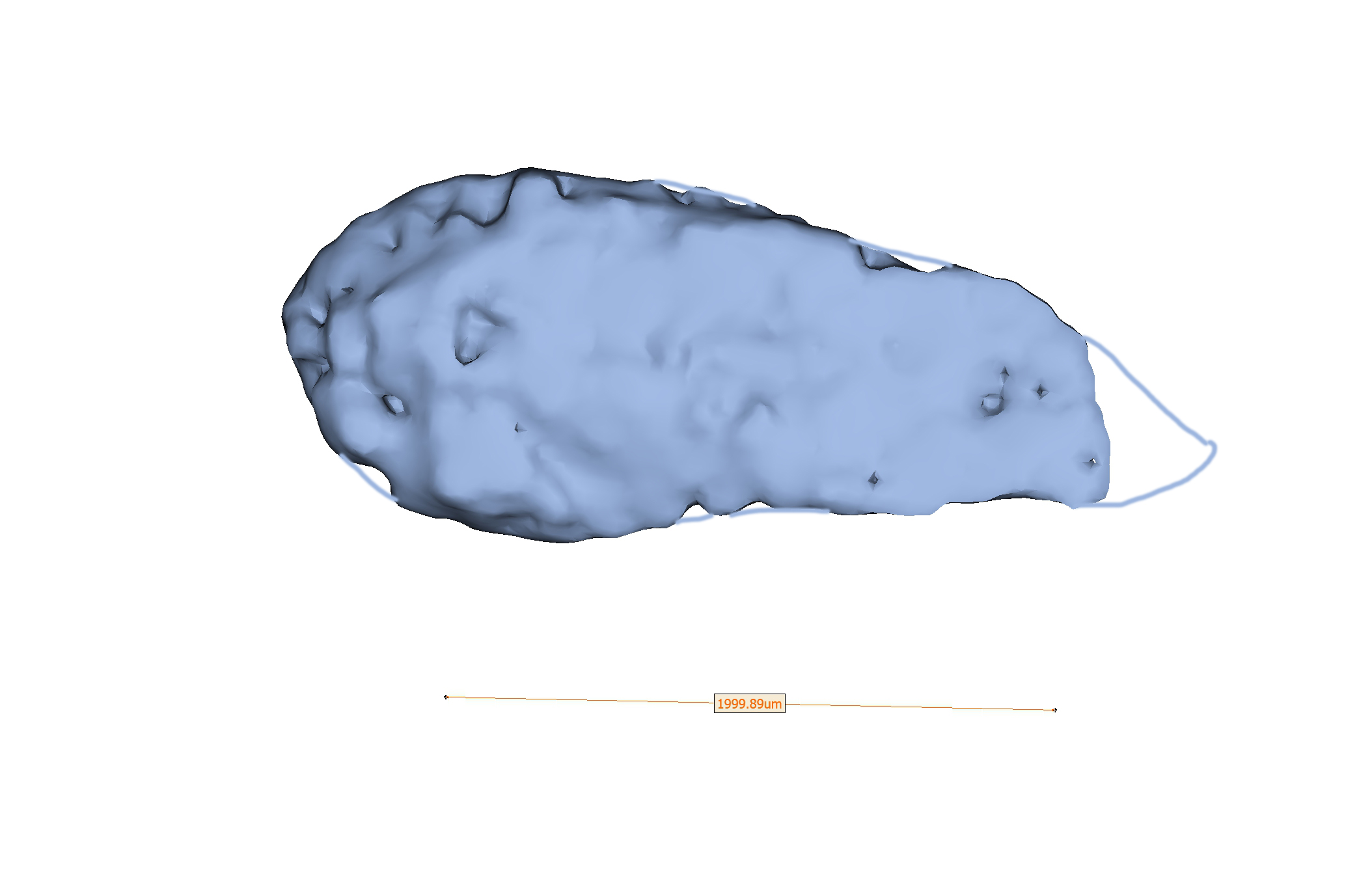

Supplement: Supplementary file 5 — Supplementary Data 2 [file 41467_2023_43557_MOESM5_ESM.zip › Supplementary Data 2/Supplementary Data 2 Raw data of Geometric Morphometric Analyses/12 Morphotypes/Morphotype 12/ts17l.jpg]

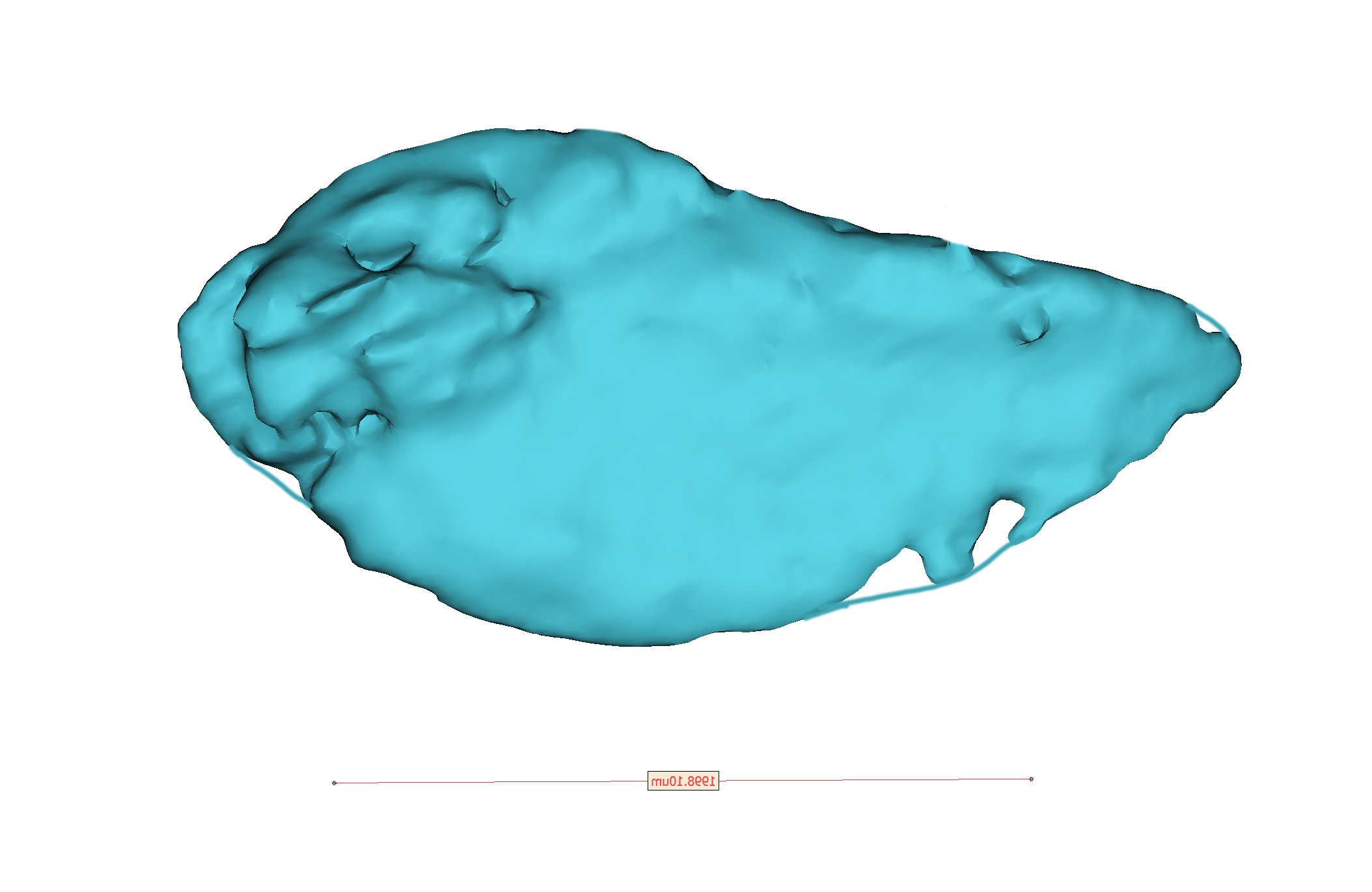

Supplement: Supplementary file 5 — Supplementary Data 2 [file 41467_2023_43557_MOESM5_ESM.zip › Supplementary Data 2/Supplementary Data 2 Raw data of Geometric Morphometric Analyses/12 Morphotypes/Morphotype 12/ts19r.jpg]

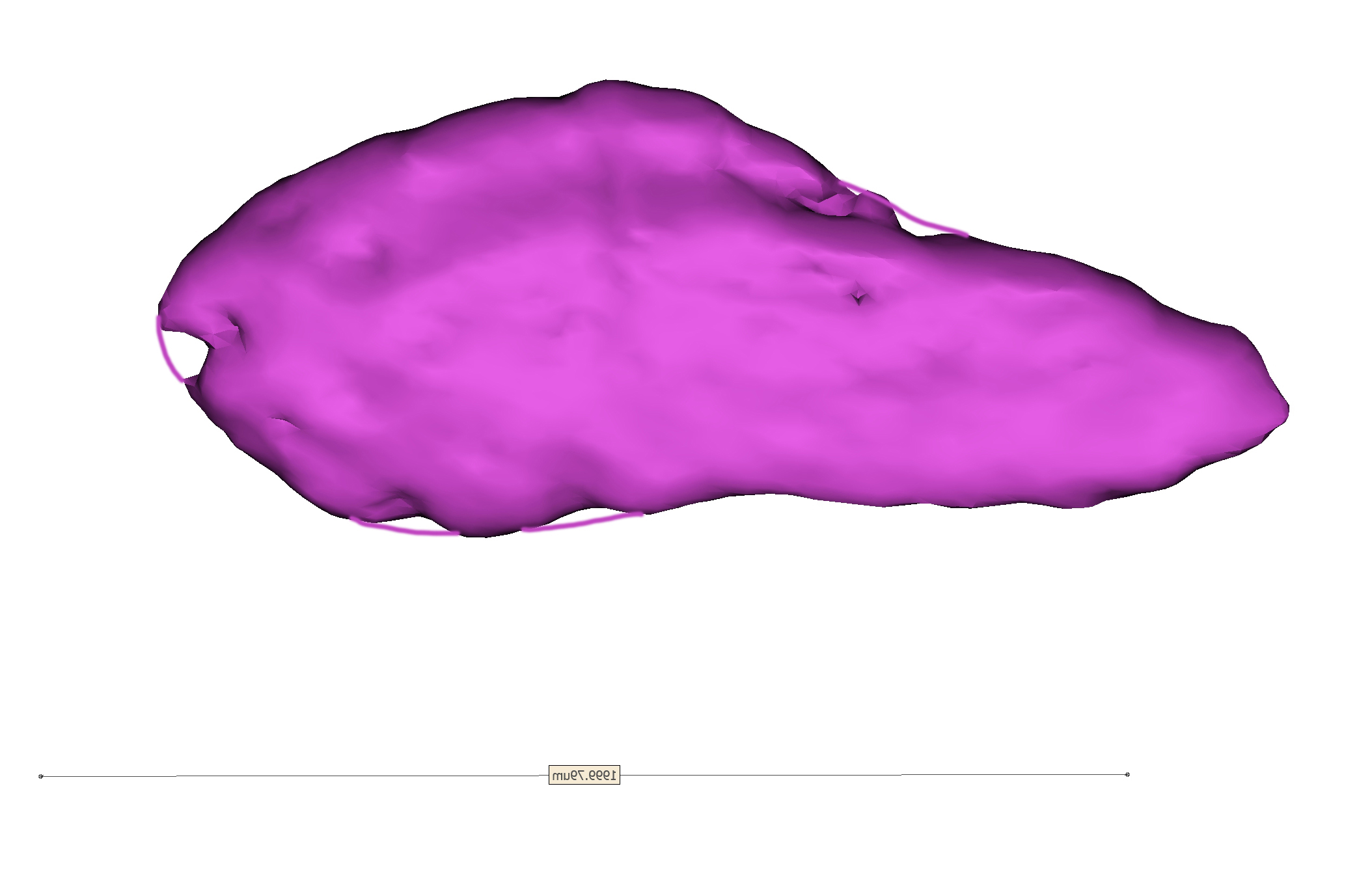

Supplement: Supplementary file 5 — Supplementary Data 2 [file 41467_2023_43557_MOESM5_ESM.zip › Supplementary Data 2/Supplementary Data 2 Raw data of Geometric Morphometric Analyses/12 Morphotypes/Morphotype 12/ts20r.jpg]

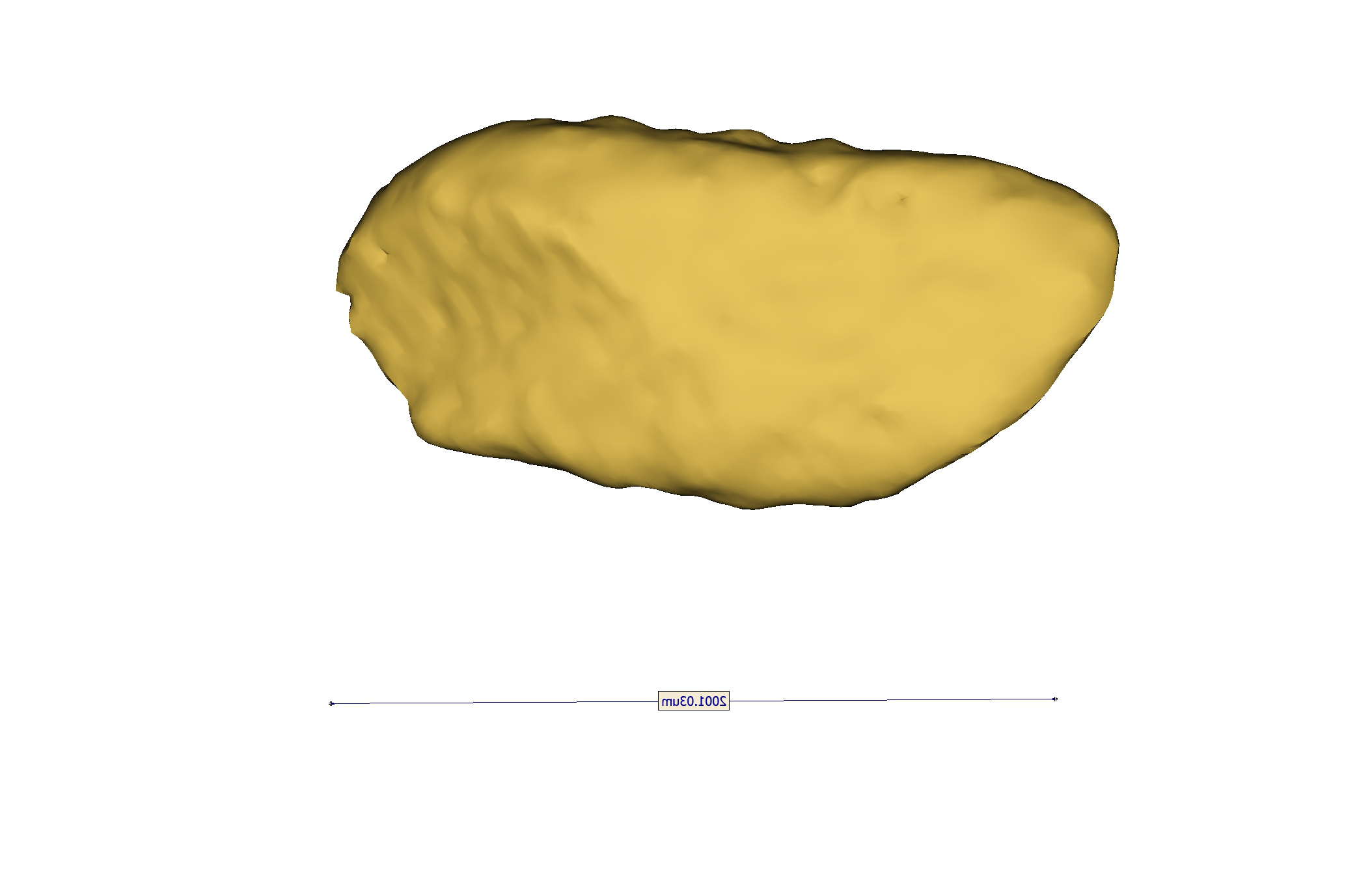

Supplement: Supplementary file 5 — Supplementary Data 2 [file 41467_2023_43557_MOESM5_ESM.zip › Supplementary Data 2/Supplementary Data 2 Raw data of Geometric Morphometric Analyses/12 Morphotypes/Morphotype 12/ts21r.jpg]

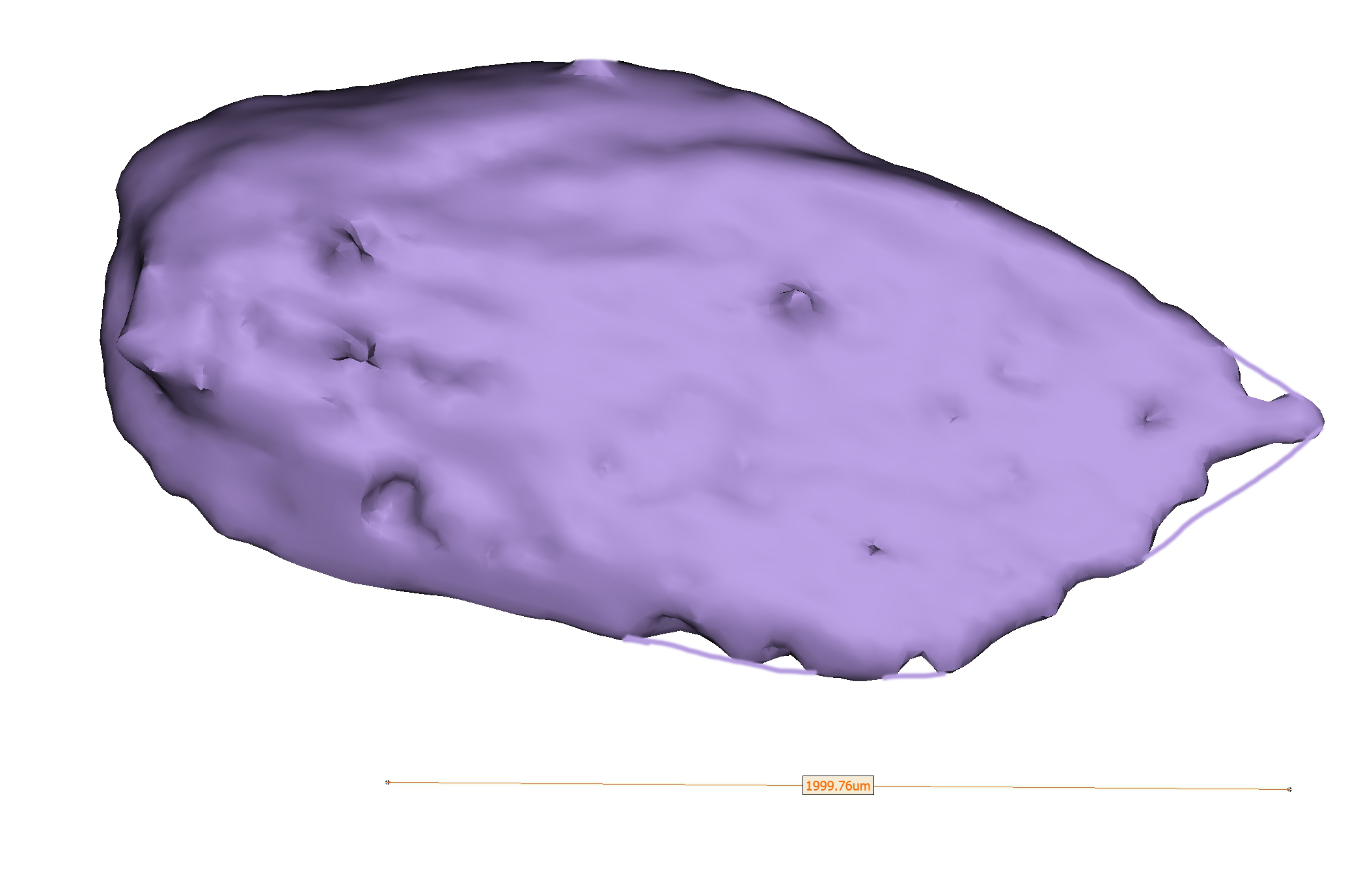

Supplement: Supplementary file 5 — Supplementary Data 2 [file 41467_2023_43557_MOESM5_ESM.zip › Supplementary Data 2/Supplementary Data 2 Raw data of Geometric Morphometric Analyses/12 Morphotypes/Morphotype 12/ts23l.jpg]

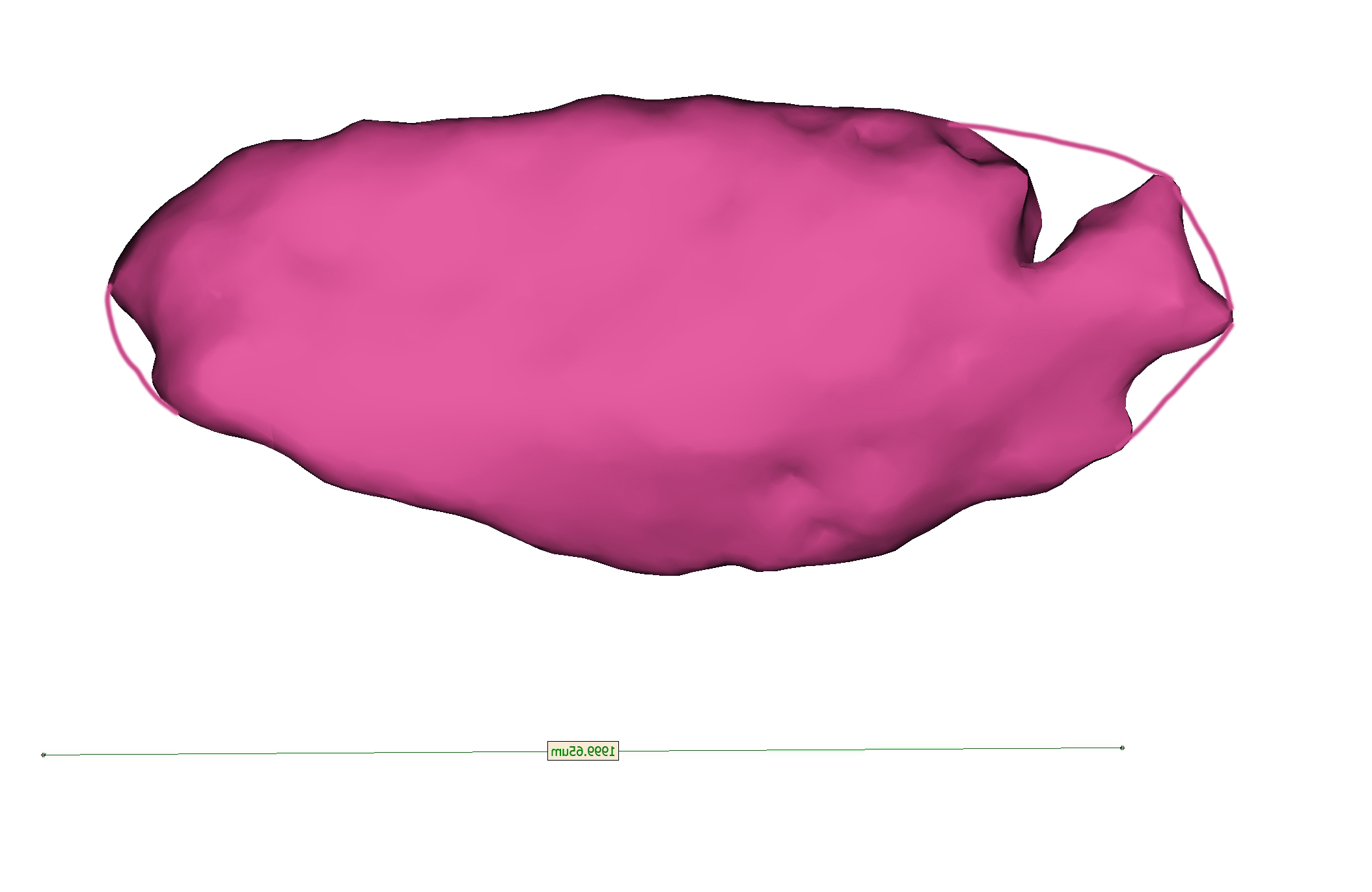

Supplement: Supplementary file 5 — Supplementary Data 2 [file 41467_2023_43557_MOESM5_ESM.zip › Supplementary Data 2/Supplementary Data 2 Raw data of Geometric Morphometric Analyses/12 Morphotypes/Morphotype 12/ts25r.jpg]

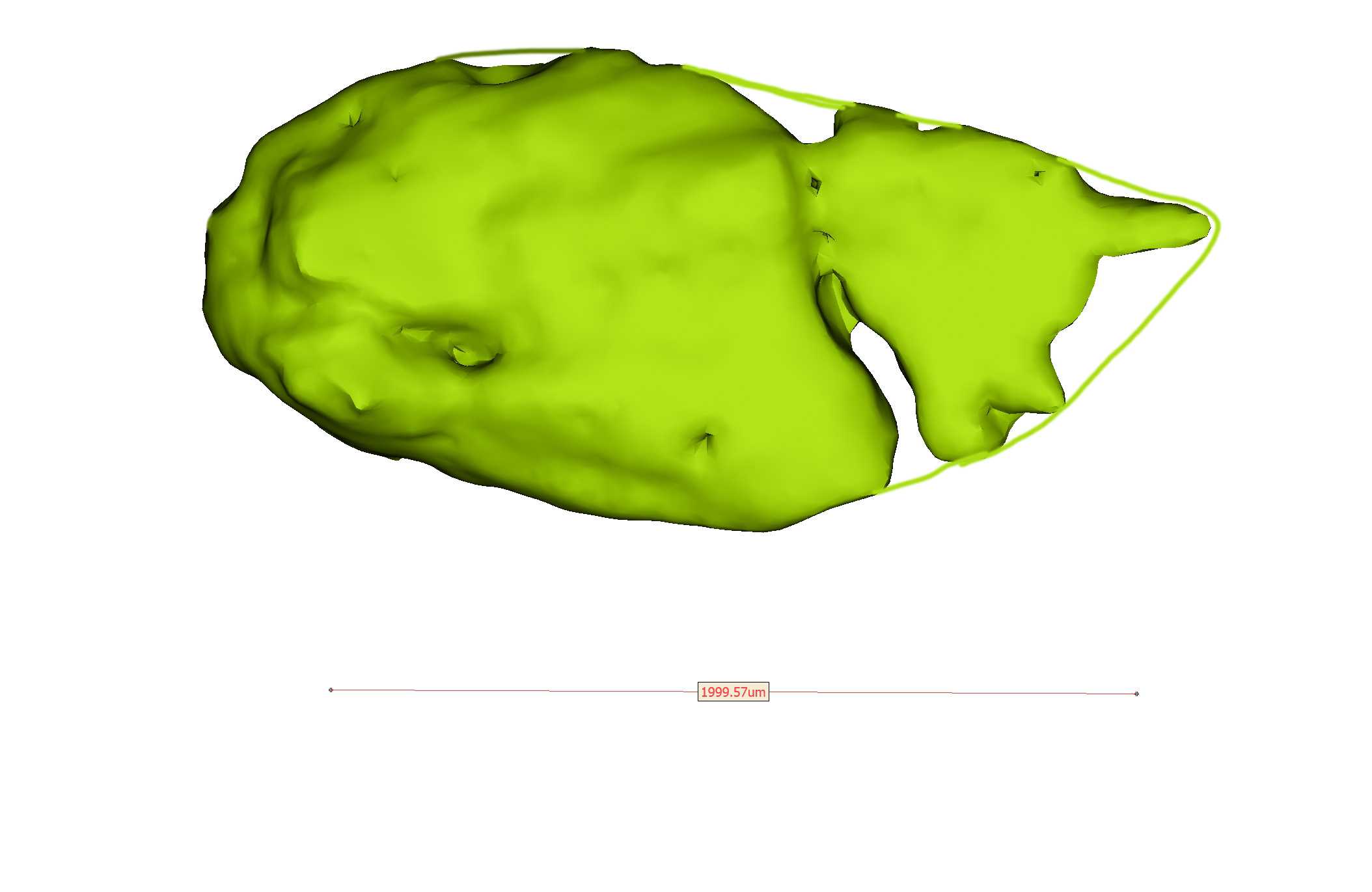

Supplement: Supplementary file 5 — Supplementary Data 2 [file 41467_2023_43557_MOESM5_ESM.zip › Supplementary Data 2/Supplementary Data 2 Raw data of Geometric Morphometric Analyses/12 Morphotypes/Morphotype 12/ts26l.jpg]

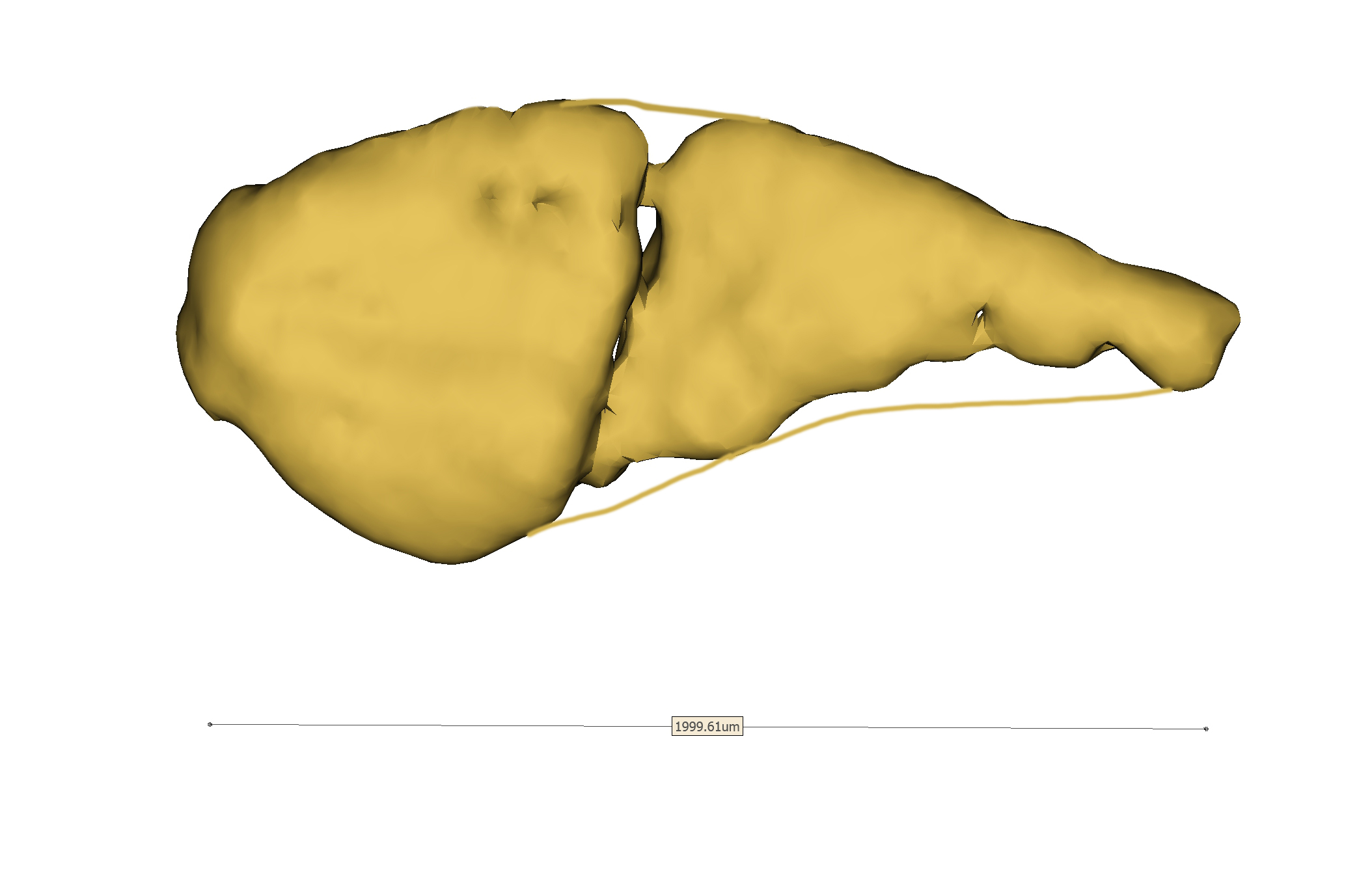

Supplement: Supplementary file 5 — Supplementary Data 2 [file 41467_2023_43557_MOESM5_ESM.zip › Supplementary Data 2/Supplementary Data 2 Raw data of Geometric Morphometric Analyses/12 Morphotypes/Morphotype 12/ts27l.jpg]

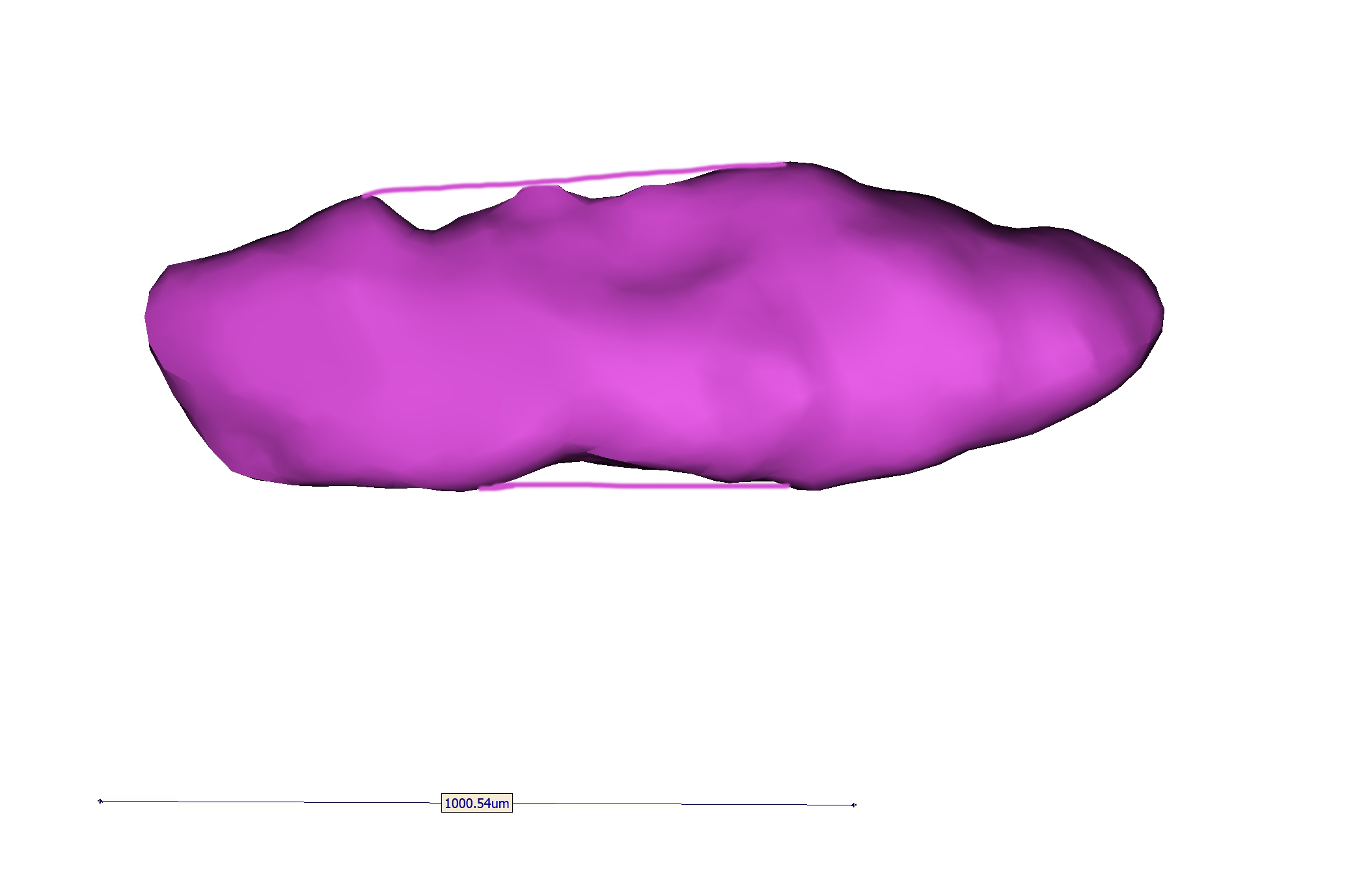

Supplement: Supplementary file 5 — Supplementary Data 2 [file 41467_2023_43557_MOESM5_ESM.zip › Supplementary Data 2/Supplementary Data 2 Raw data of Geometric Morphometric Analyses/12 Morphotypes/Morphotype 12/ts30(long).jpg]

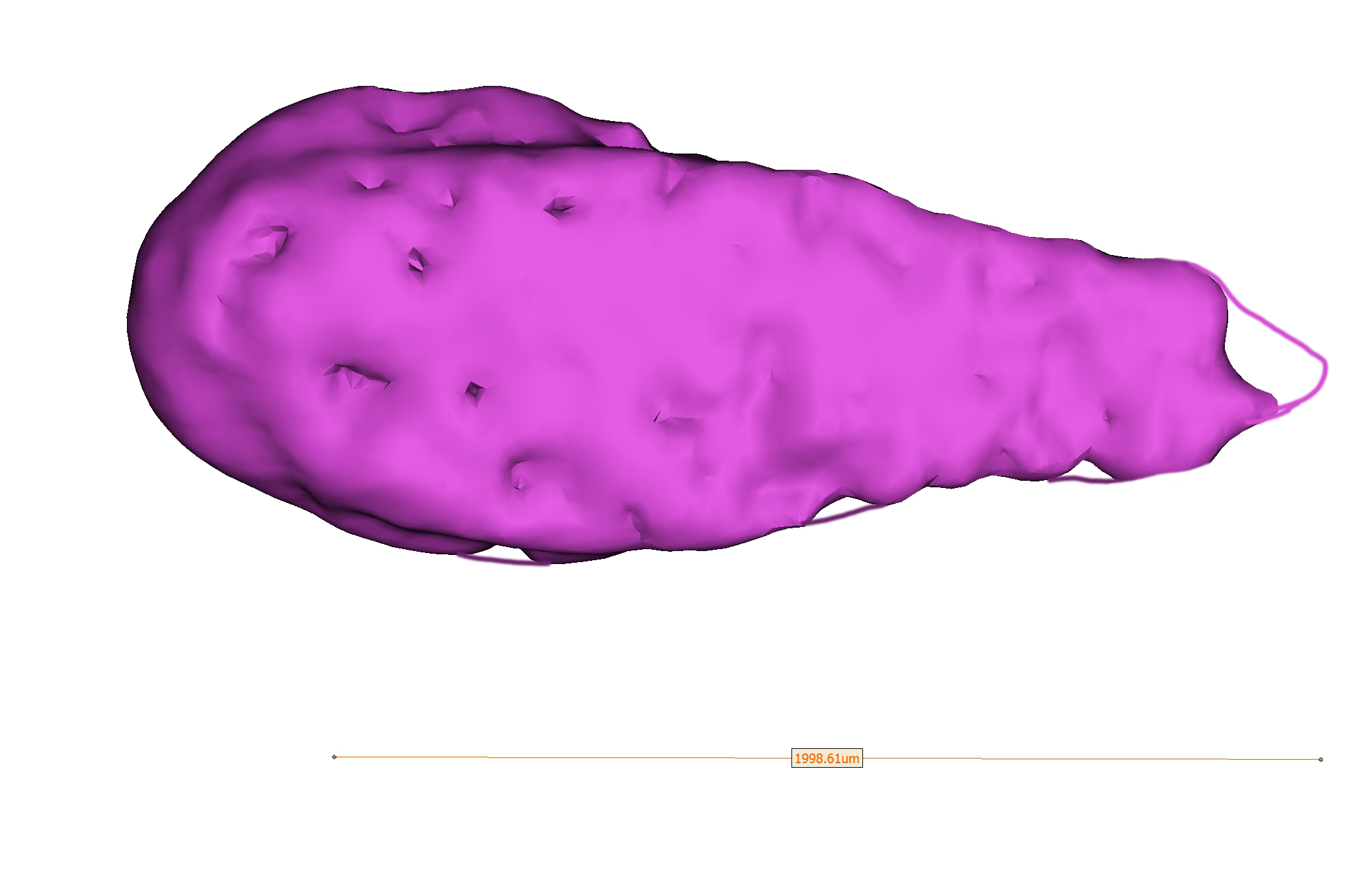

Supplement: Supplementary file 5 — Supplementary Data 2 [file 41467_2023_43557_MOESM5_ESM.zip › Supplementary Data 2/Supplementary Data 2 Raw data of Geometric Morphometric Analyses/12 Morphotypes/Morphotype 12/ts33l.jpg]

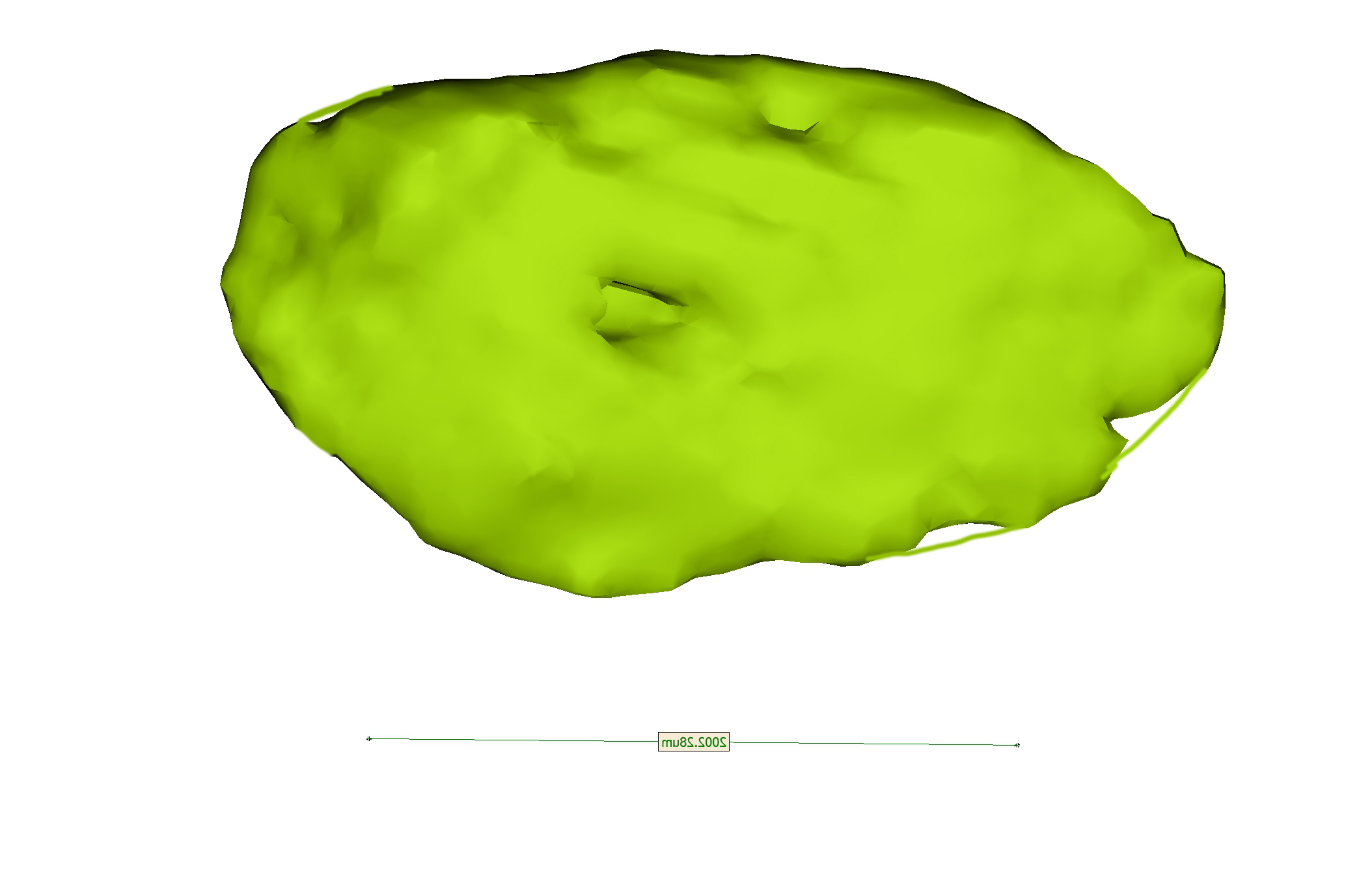

Supplement: Supplementary file 5 — Supplementary Data 2 [file 41467_2023_43557_MOESM5_ESM.zip › Supplementary Data 2/Supplementary Data 2 Raw data of Geometric Morphometric Analyses/12 Morphotypes/Morphotype 12/ts34r.jpg]

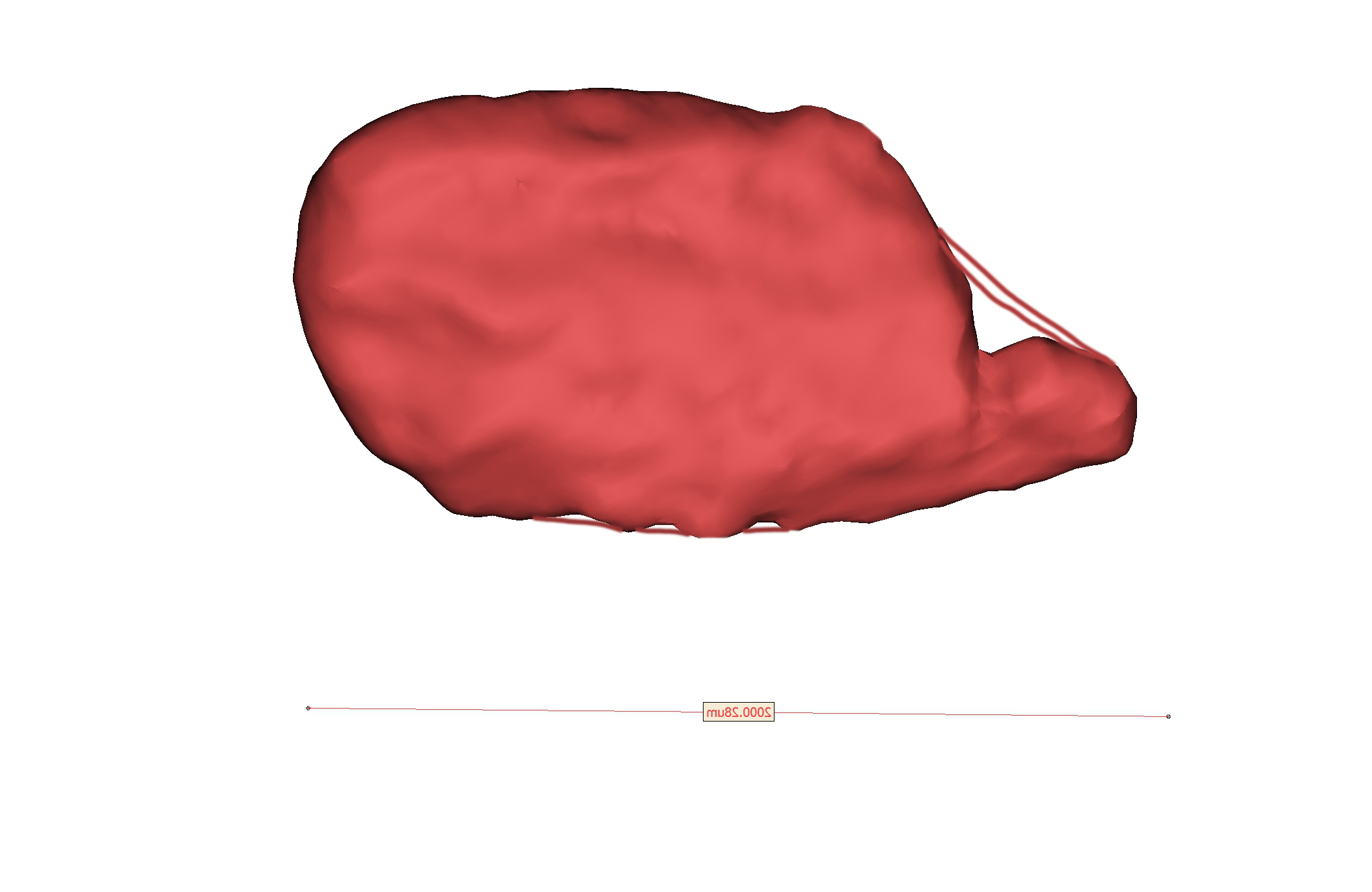

Supplement: Supplementary file 5 — Supplementary Data 2 [file 41467_2023_43557_MOESM5_ESM.zip › Supplementary Data 2/Supplementary Data 2 Raw data of Geometric Morphometric Analyses/12 Morphotypes/Morphotype 12/ts37r.jpg]

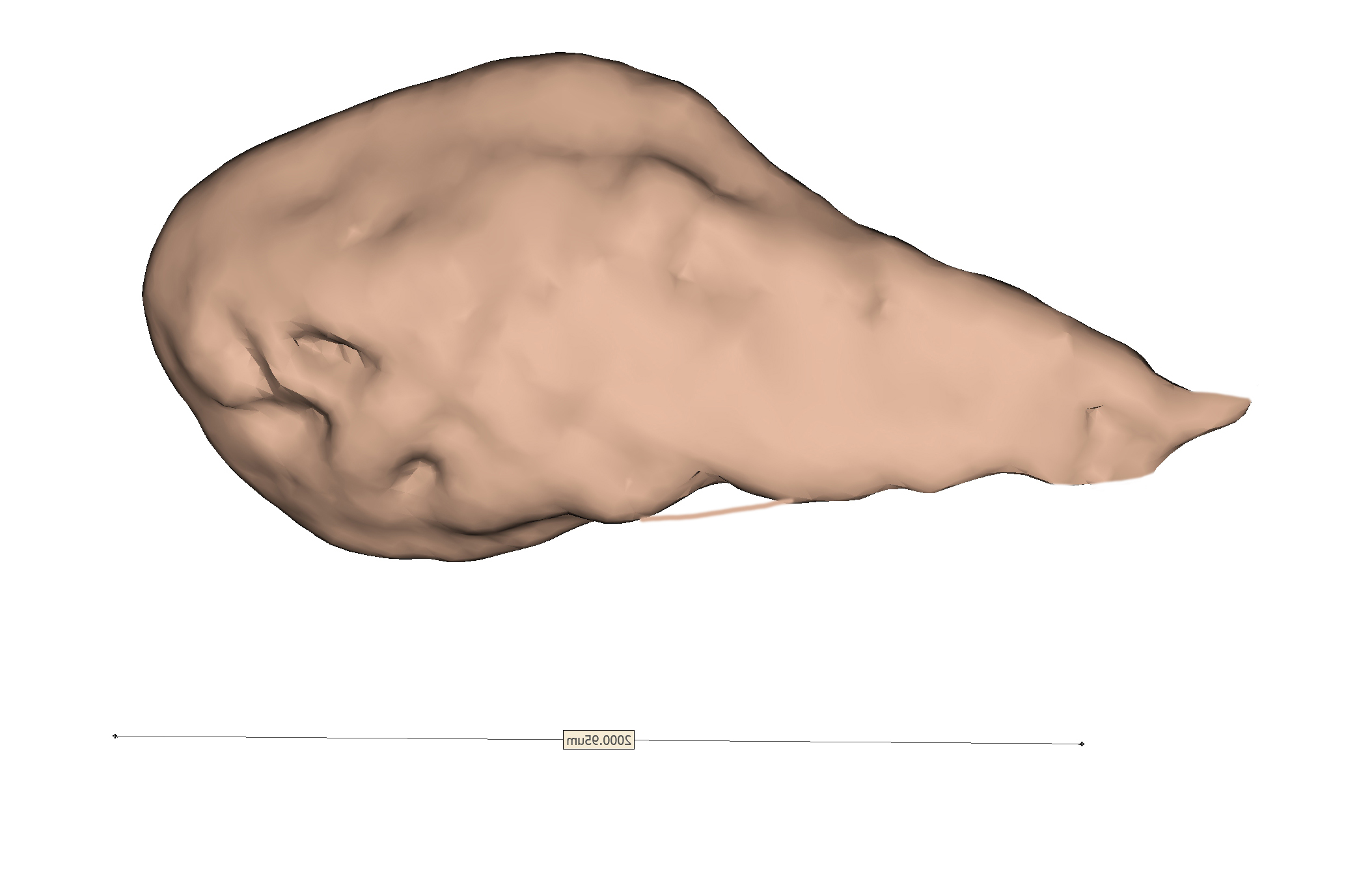

Supplement: Supplementary file 5 — Supplementary Data 2 [file 41467_2023_43557_MOESM5_ESM.zip › Supplementary Data 2/Supplementary Data 2 Raw data of Geometric Morphometric Analyses/12 Morphotypes/Morphotype 12/ts38r.jpg]

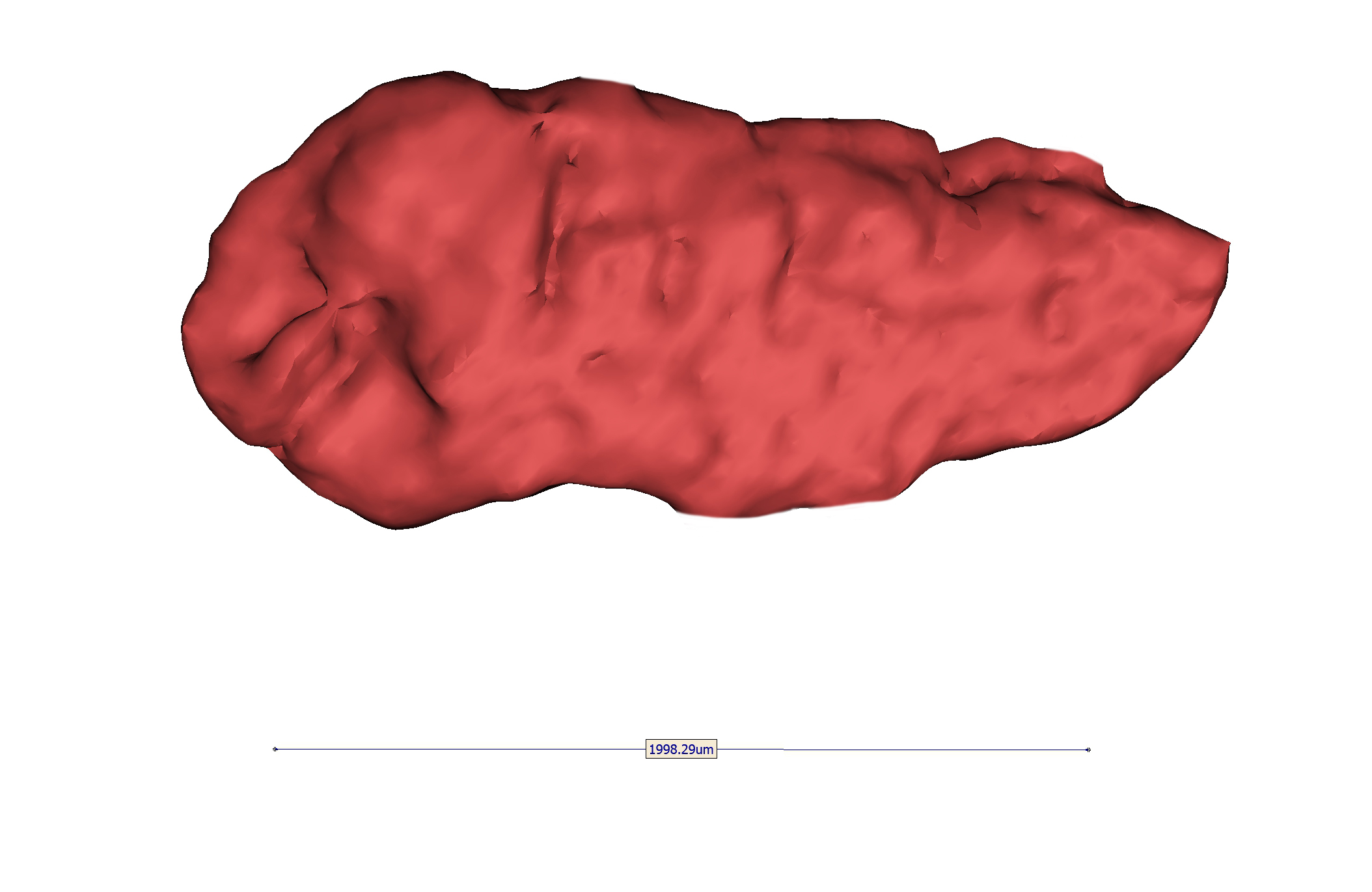

Supplement: Supplementary file 5 — Supplementary Data 2 [file 41467_2023_43557_MOESM5_ESM.zip › Supplementary Data 2/Supplementary Data 2 Raw data of Geometric Morphometric Analyses/12 Morphotypes/Morphotype 12/ts39l.jpg]

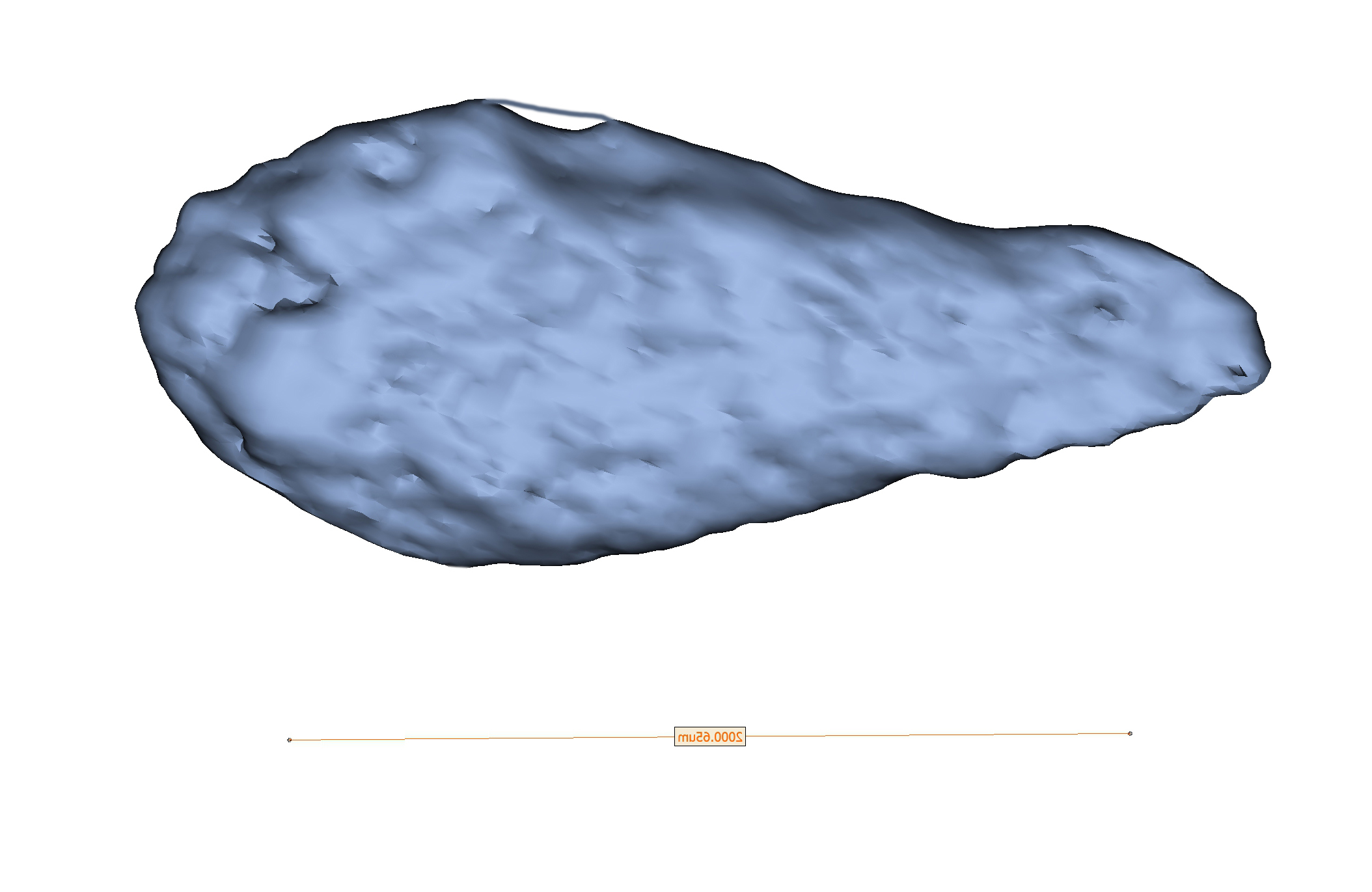

Supplement: Supplementary file 5 — Supplementary Data 2 [file 41467_2023_43557_MOESM5_ESM.zip › Supplementary Data 2/Supplementary Data 2 Raw data of Geometric Morphometric Analyses/12 Morphotypes/Morphotype 12/ts40r.jpg]

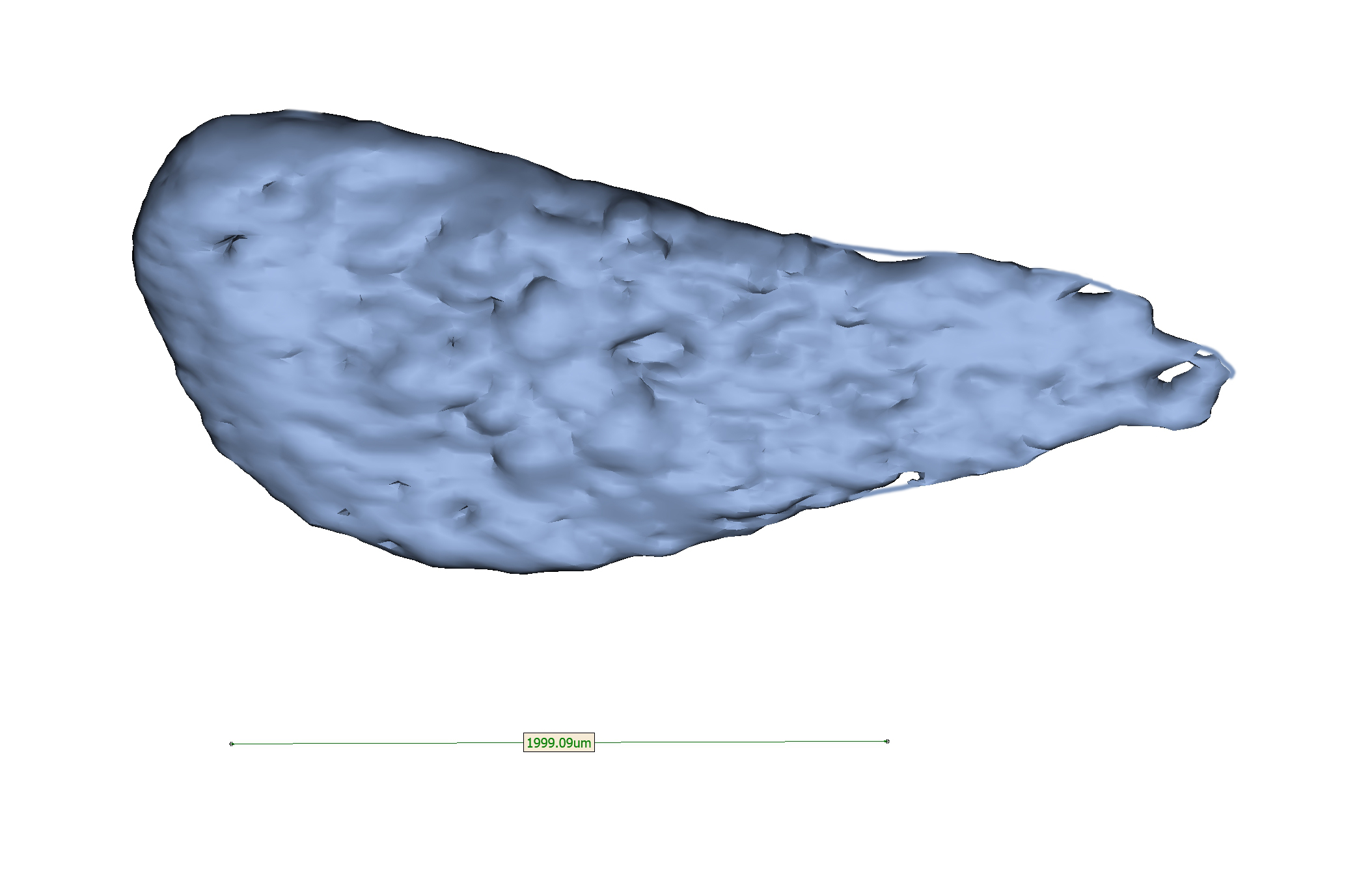

Supplement: Supplementary file 5 — Supplementary Data 2 [file 41467_2023_43557_MOESM5_ESM.zip › Supplementary Data 2/Supplementary Data 2 Raw data of Geometric Morphometric Analyses/12 Morphotypes/Morphotype 12/ts41l.jpg]

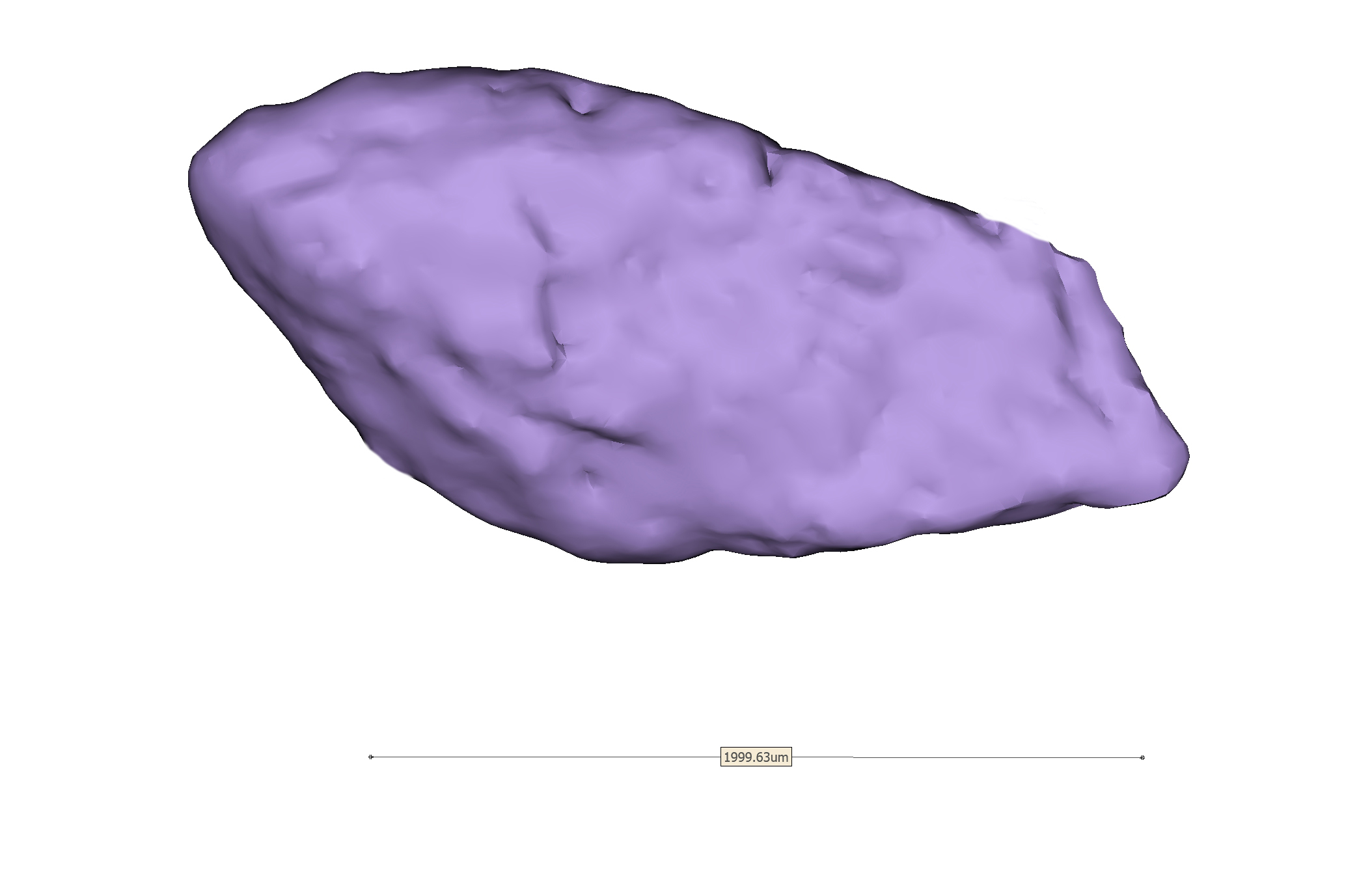

Supplement: Supplementary file 5 — Supplementary Data 2 [file 41467_2023_43557_MOESM5_ESM.zip › Supplementary Data 2/Supplementary Data 2 Raw data of Geometric Morphometric Analyses/12 Morphotypes/Morphotype 12/ts43l.jpg]

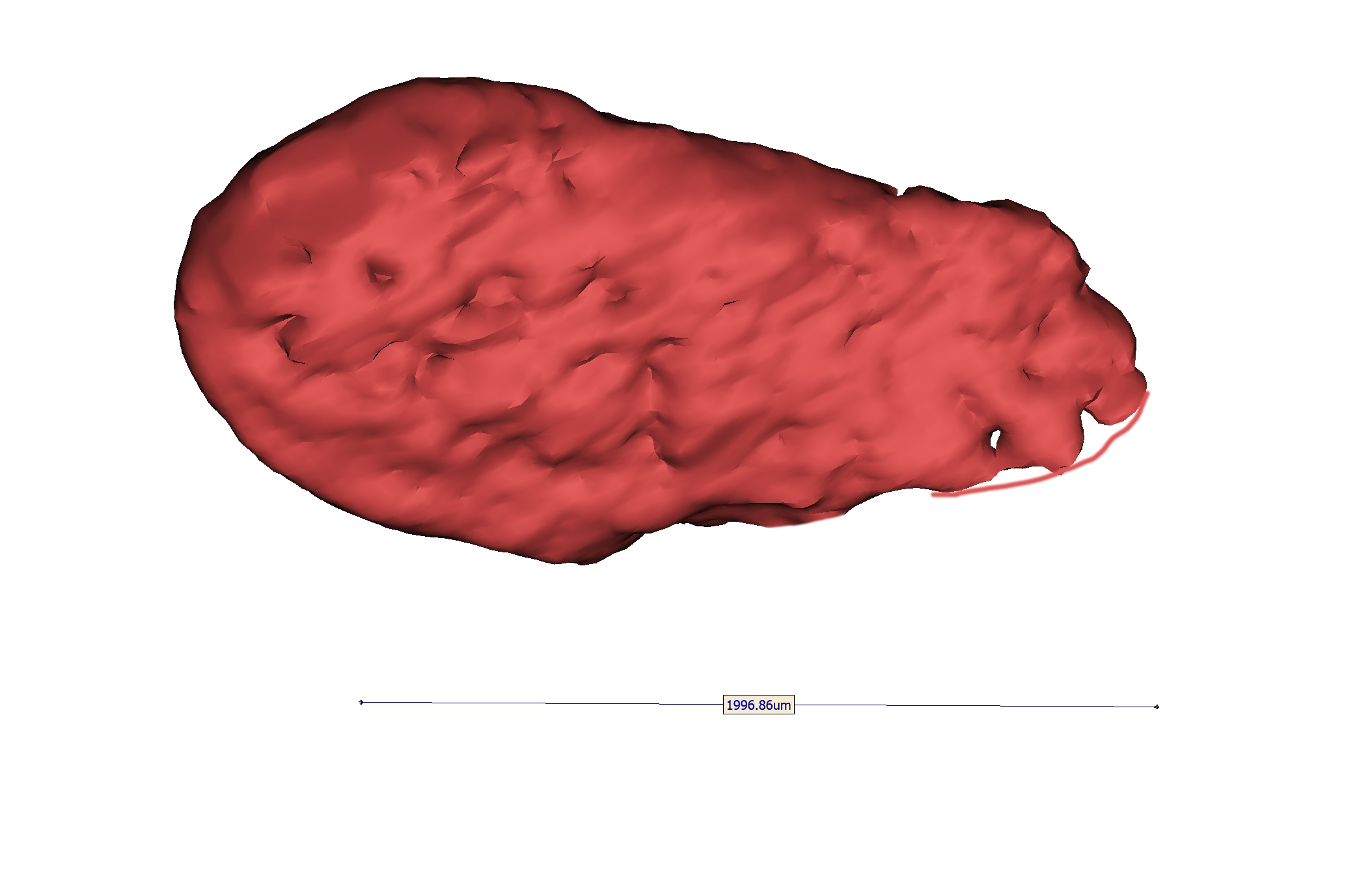

Supplement: Supplementary file 5 — Supplementary Data 2 [file 41467_2023_43557_MOESM5_ESM.zip › Supplementary Data 2/Supplementary Data 2 Raw data of Geometric Morphometric Analyses/12 Morphotypes/Morphotype 12/ts44l.jpg]

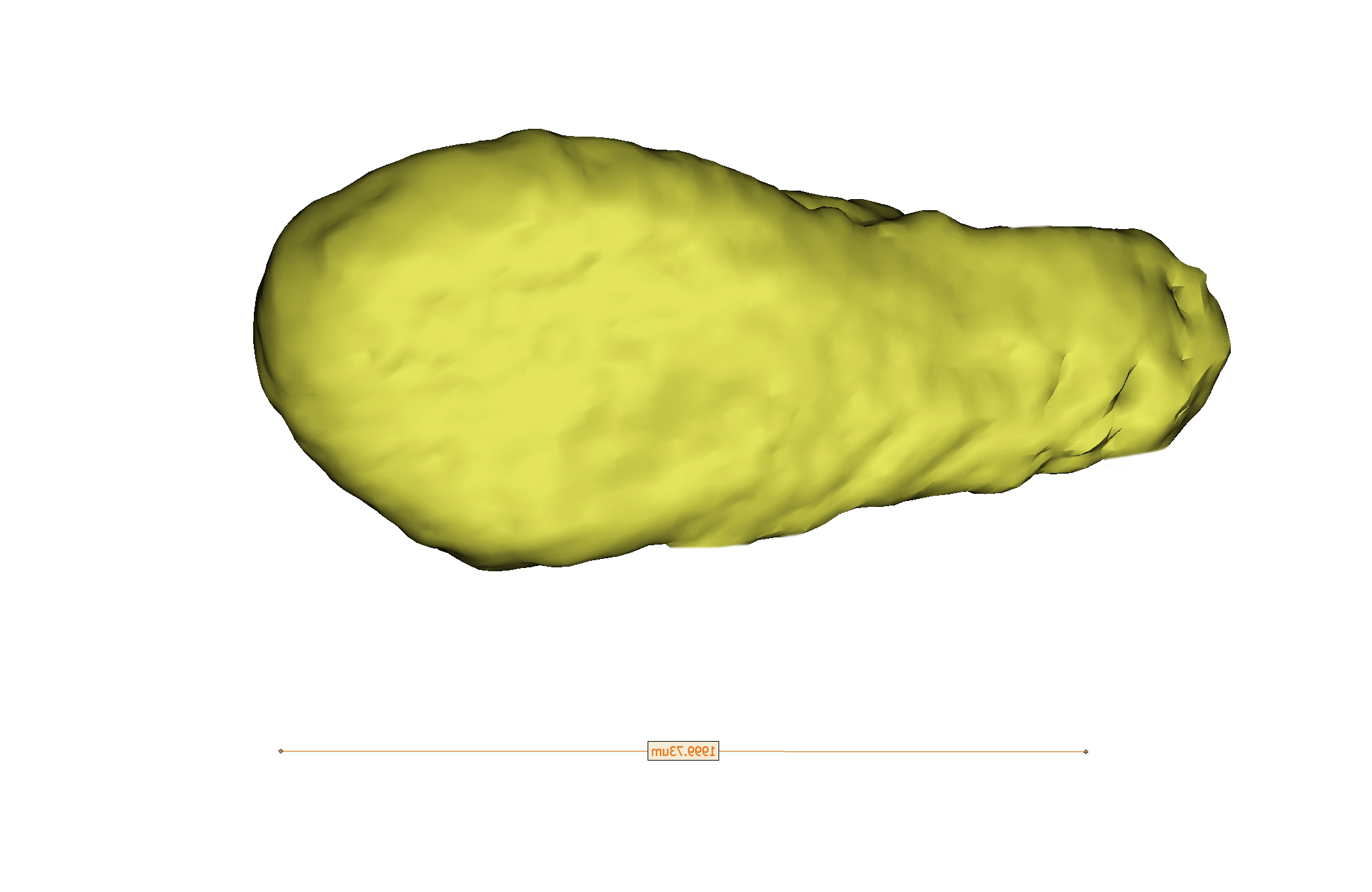

Supplement: Supplementary file 5 — Supplementary Data 2 [file 41467_2023_43557_MOESM5_ESM.zip › Supplementary Data 2/Supplementary Data 2 Raw data of Geometric Morphometric Analyses/12 Morphotypes/Morphotype 12/ts45r.jpg]

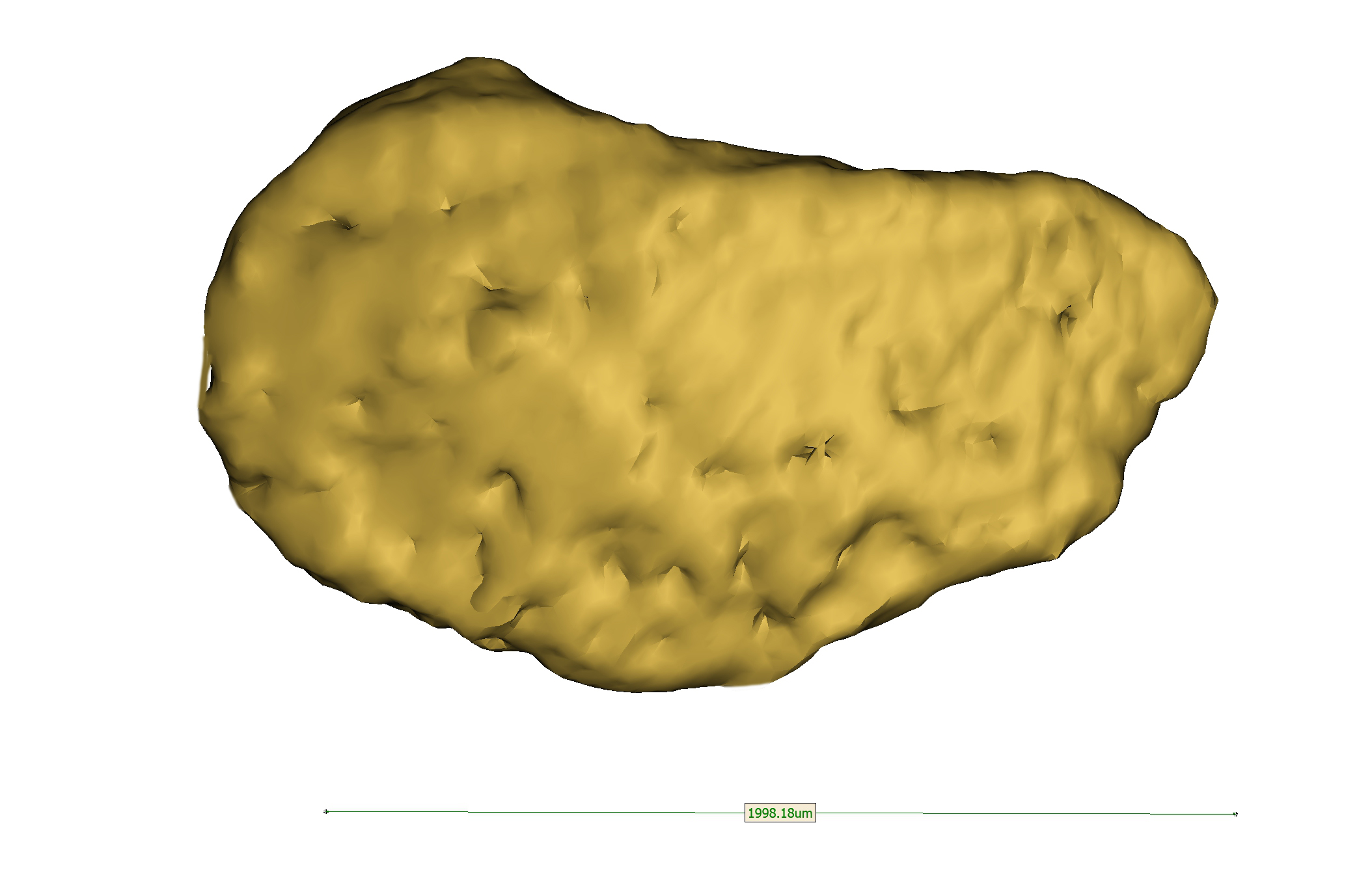

Supplement: Supplementary file 5 — Supplementary Data 2 [file 41467_2023_43557_MOESM5_ESM.zip › Supplementary Data 2/Supplementary Data 2 Raw data of Geometric Morphometric Analyses/12 Morphotypes/Morphotype 12/ts46l.jpg]

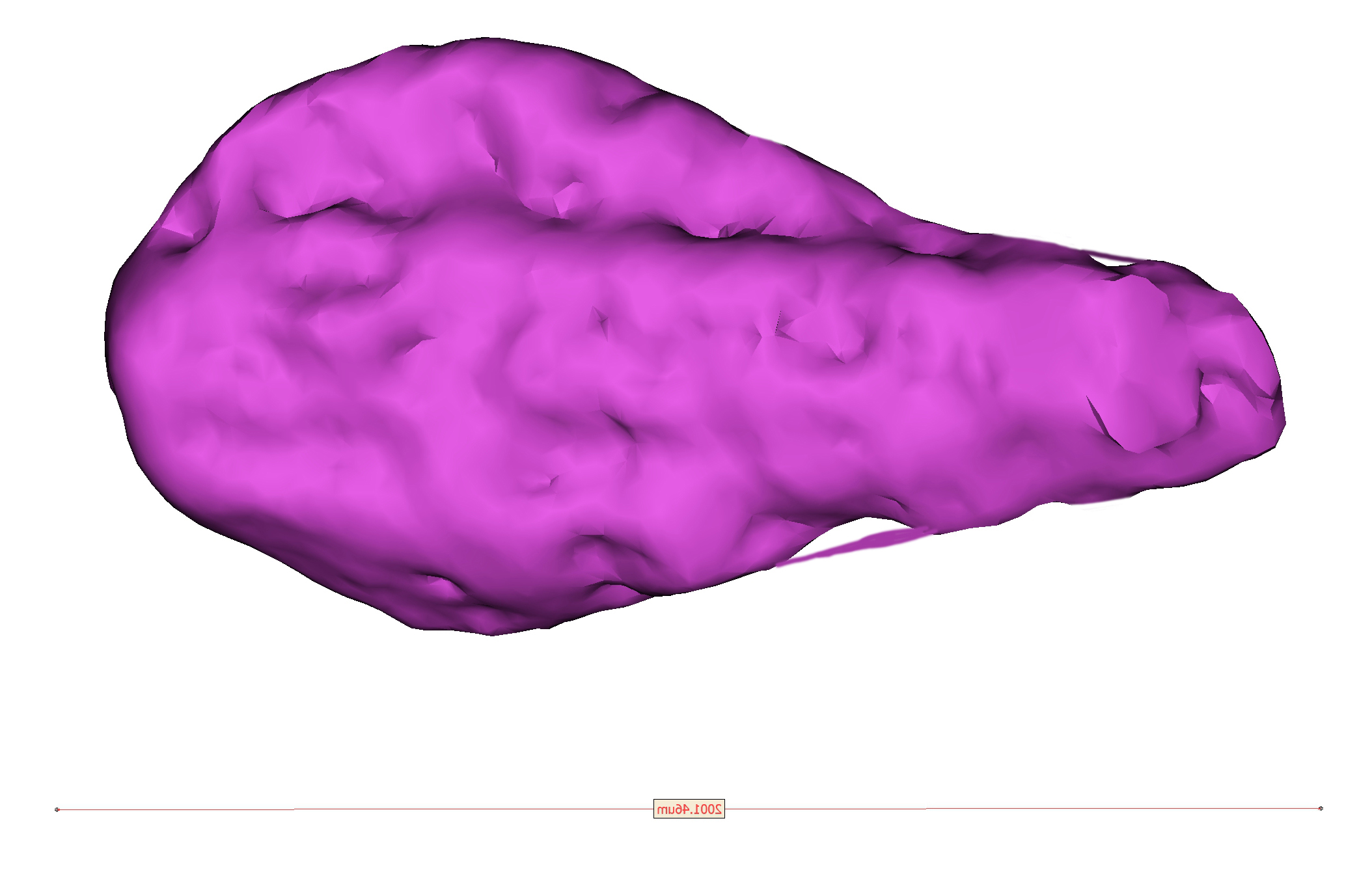

Supplement: Supplementary file 5 — Supplementary Data 2 [file 41467_2023_43557_MOESM5_ESM.zip › Supplementary Data 2/Supplementary Data 2 Raw data of Geometric Morphometric Analyses/12 Morphotypes/Morphotype 12/ts47r.jpg]

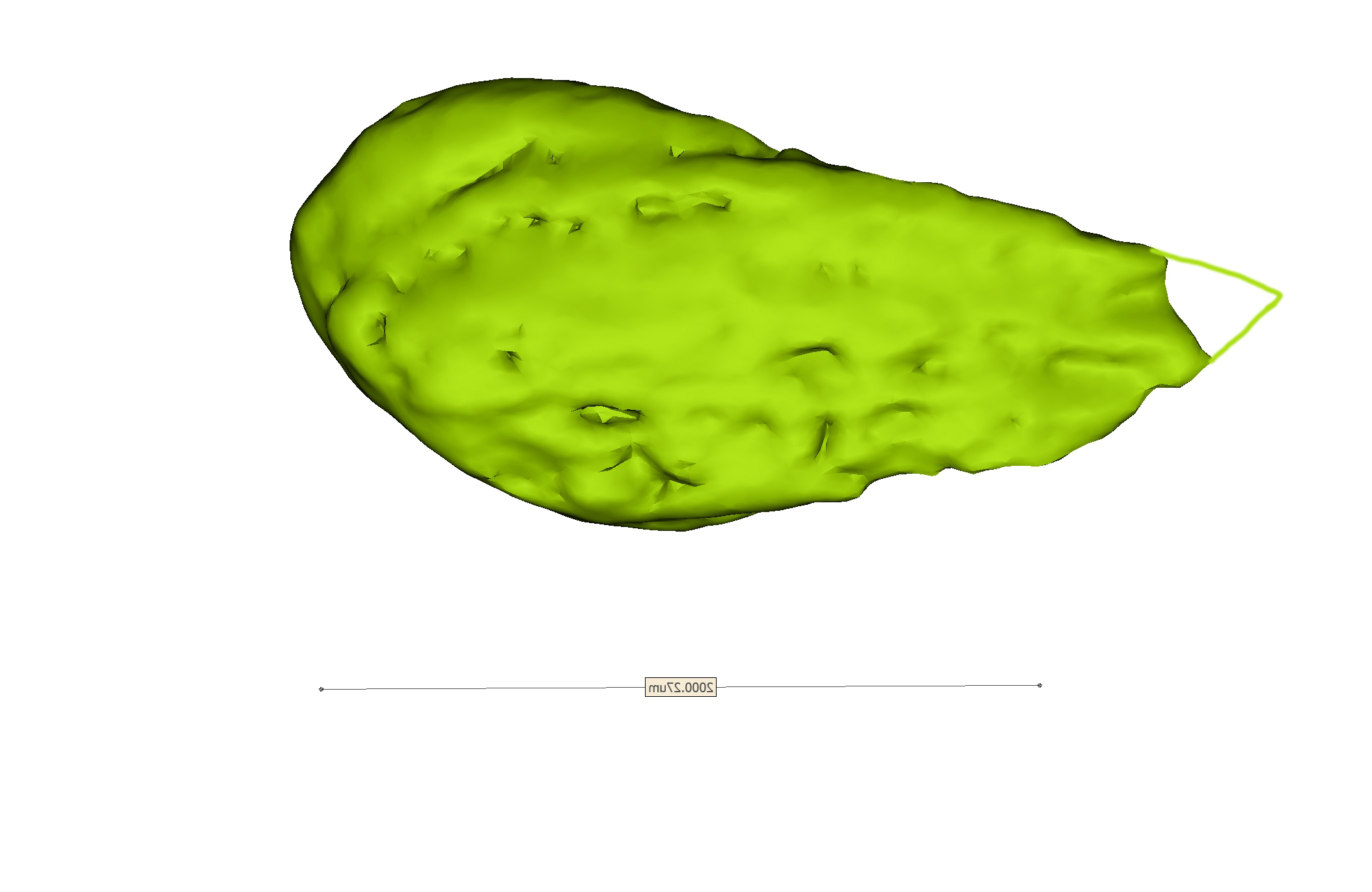

Supplement: Supplementary file 5 — Supplementary Data 2 [file 41467_2023_43557_MOESM5_ESM.zip › Supplementary Data 2/Supplementary Data 2 Raw data of Geometric Morphometric Analyses/12 Morphotypes/Morphotype 12/vf04r.jpg]

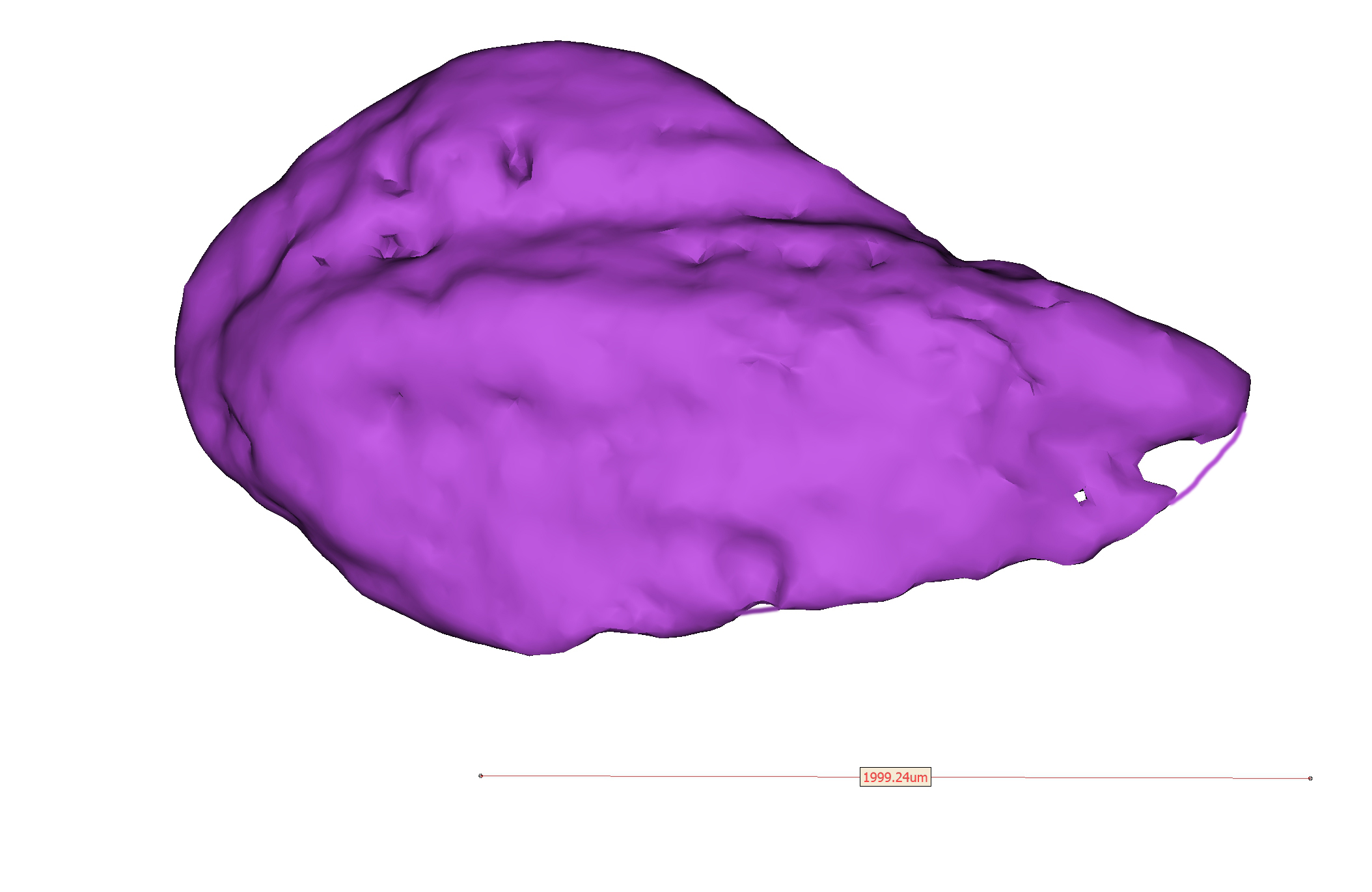

Supplement: Supplementary file 5 — Supplementary Data 2 [file 41467_2023_43557_MOESM5_ESM.zip › Supplementary Data 2/Supplementary Data 2 Raw data of Geometric Morphometric Analyses/12 Morphotypes/Morphotype 12/vf05l.jpg]

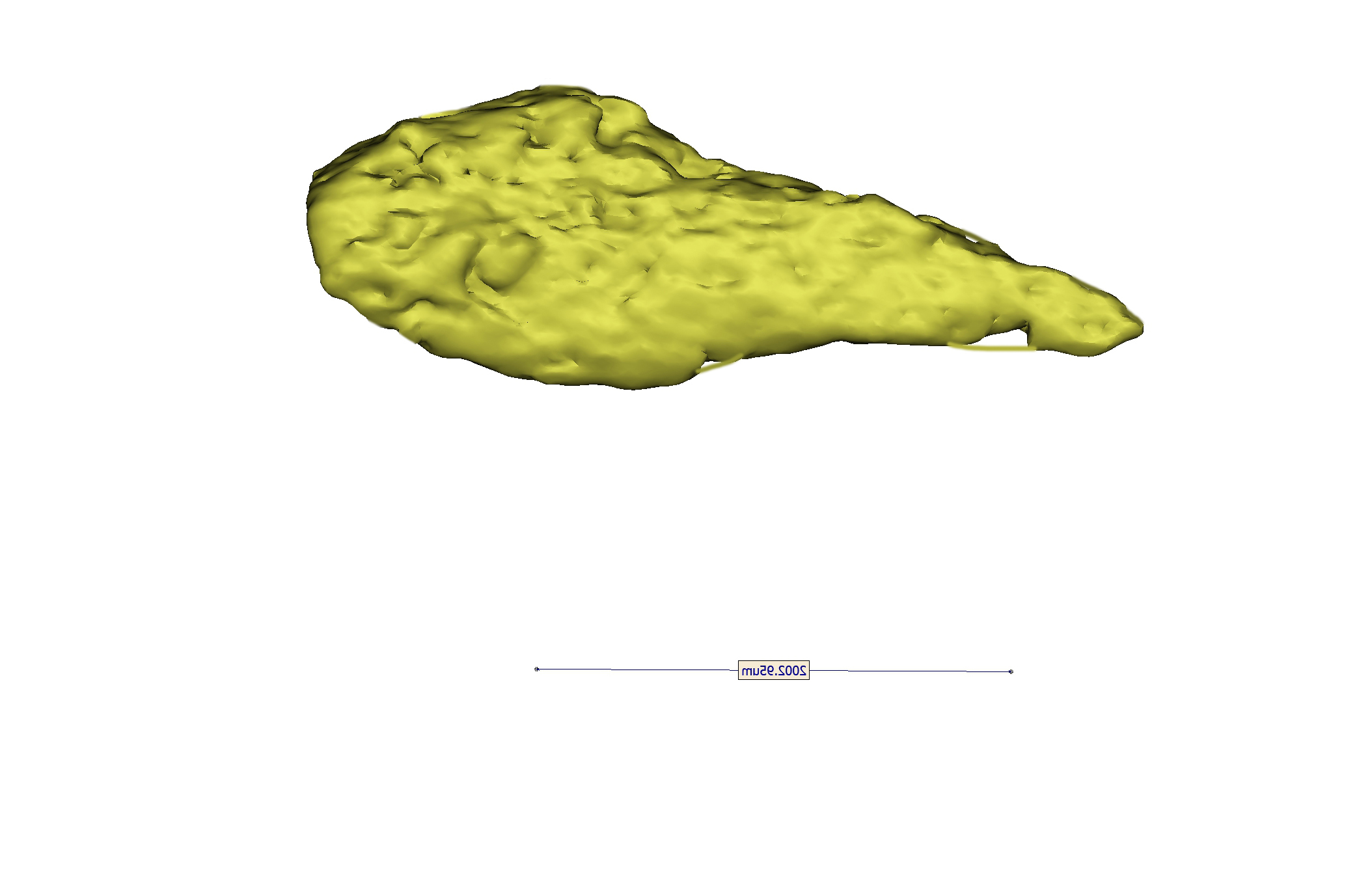

Supplement: Supplementary file 5 — Supplementary Data 2 [file 41467_2023_43557_MOESM5_ESM.zip › Supplementary Data 2/Supplementary Data 2 Raw data of Geometric Morphometric Analyses/12 Morphotypes/Morphotype 12/vf07r.jpg]

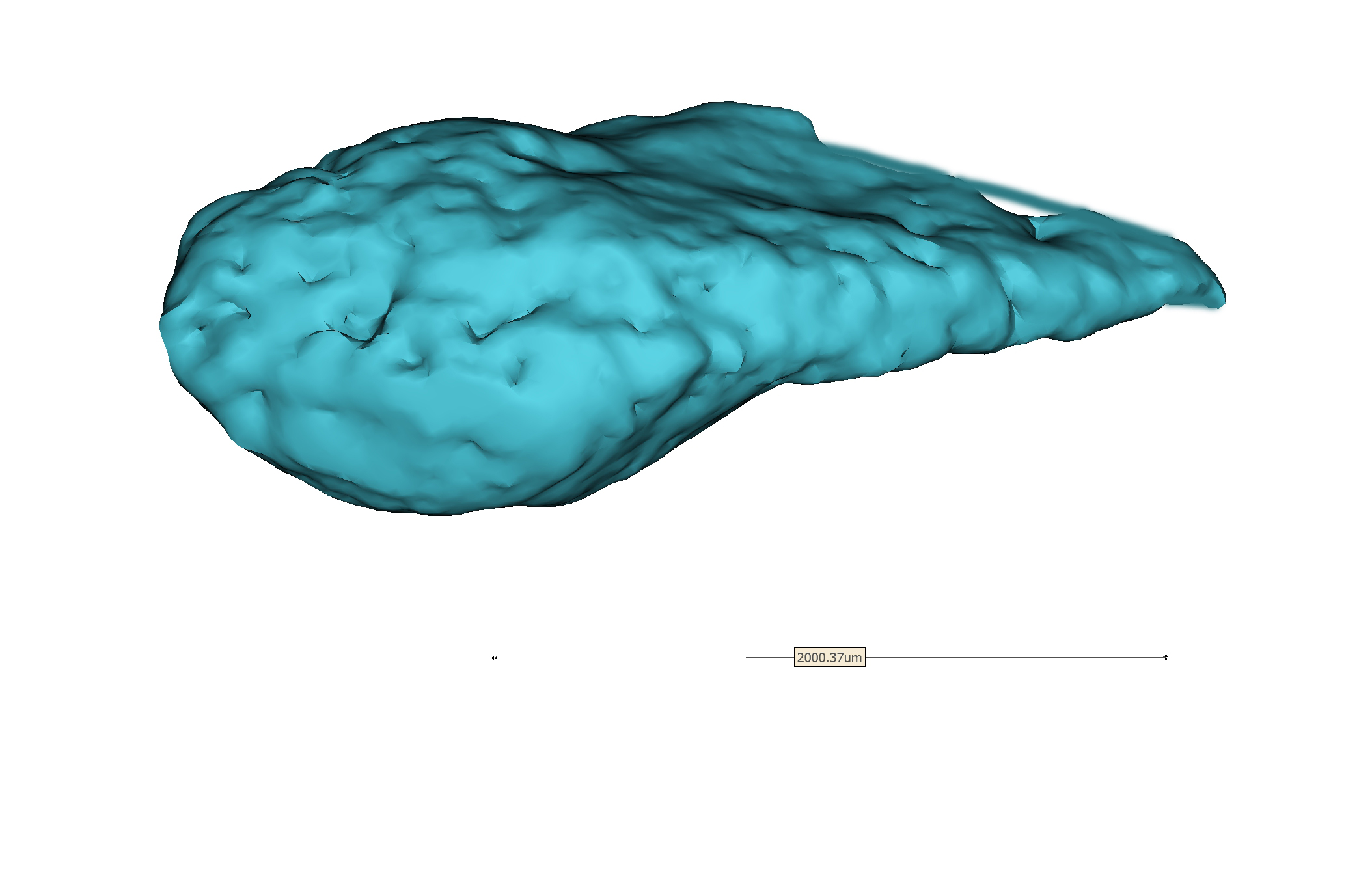

Supplement: Supplementary file 5 — Supplementary Data 2 [file 41467_2023_43557_MOESM5_ESM.zip › Supplementary Data 2/Supplementary Data 2 Raw data of Geometric Morphometric Analyses/12 Morphotypes/Morphotype 12/vf08l.jpg]

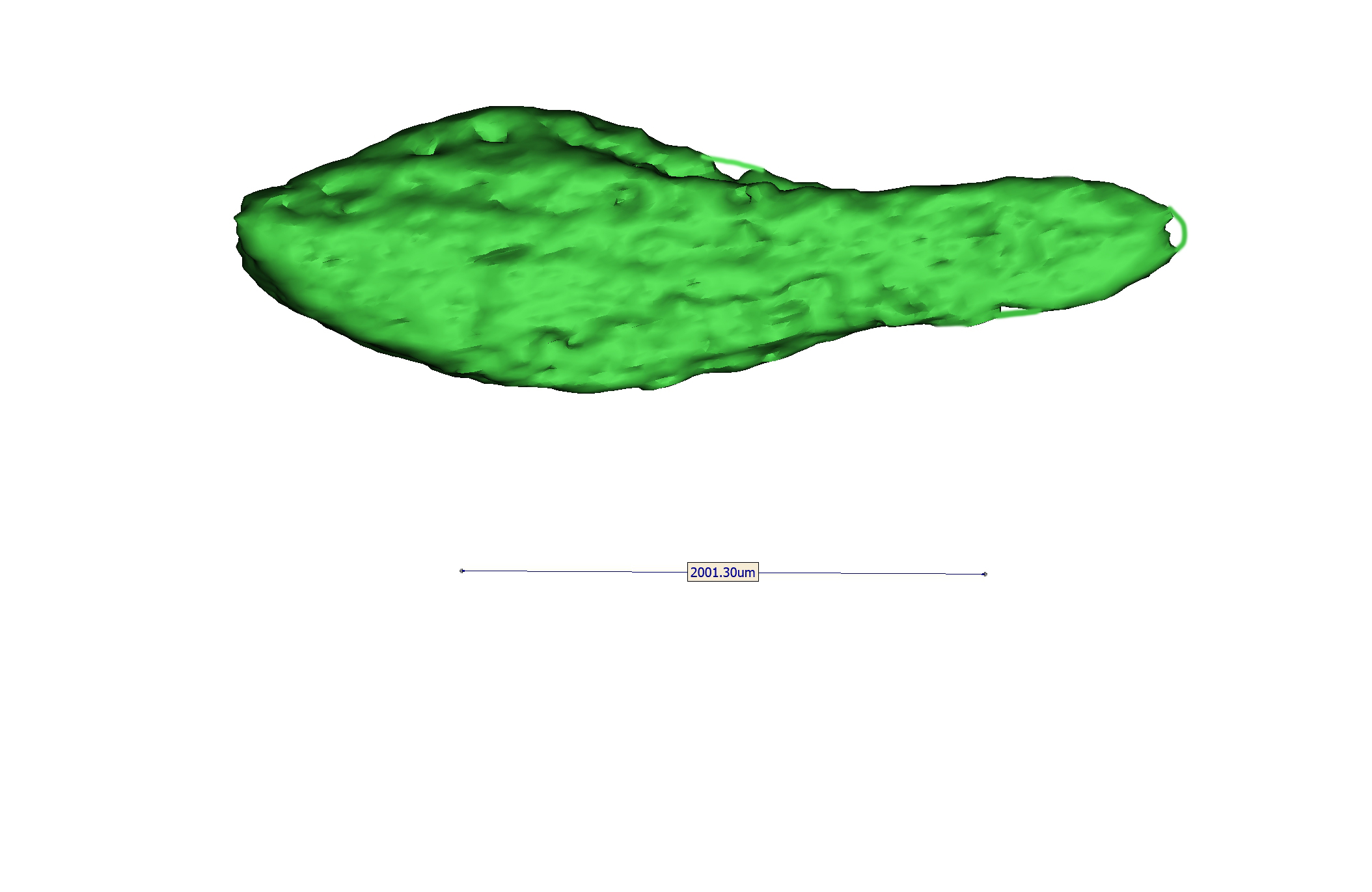

Supplement: Supplementary file 5 — Supplementary Data 2 [file 41467_2023_43557_MOESM5_ESM.zip › Supplementary Data 2/Supplementary Data 2 Raw data of Geometric Morphometric Analyses/12 Morphotypes/Morphotype 12/vf09l.jpg]

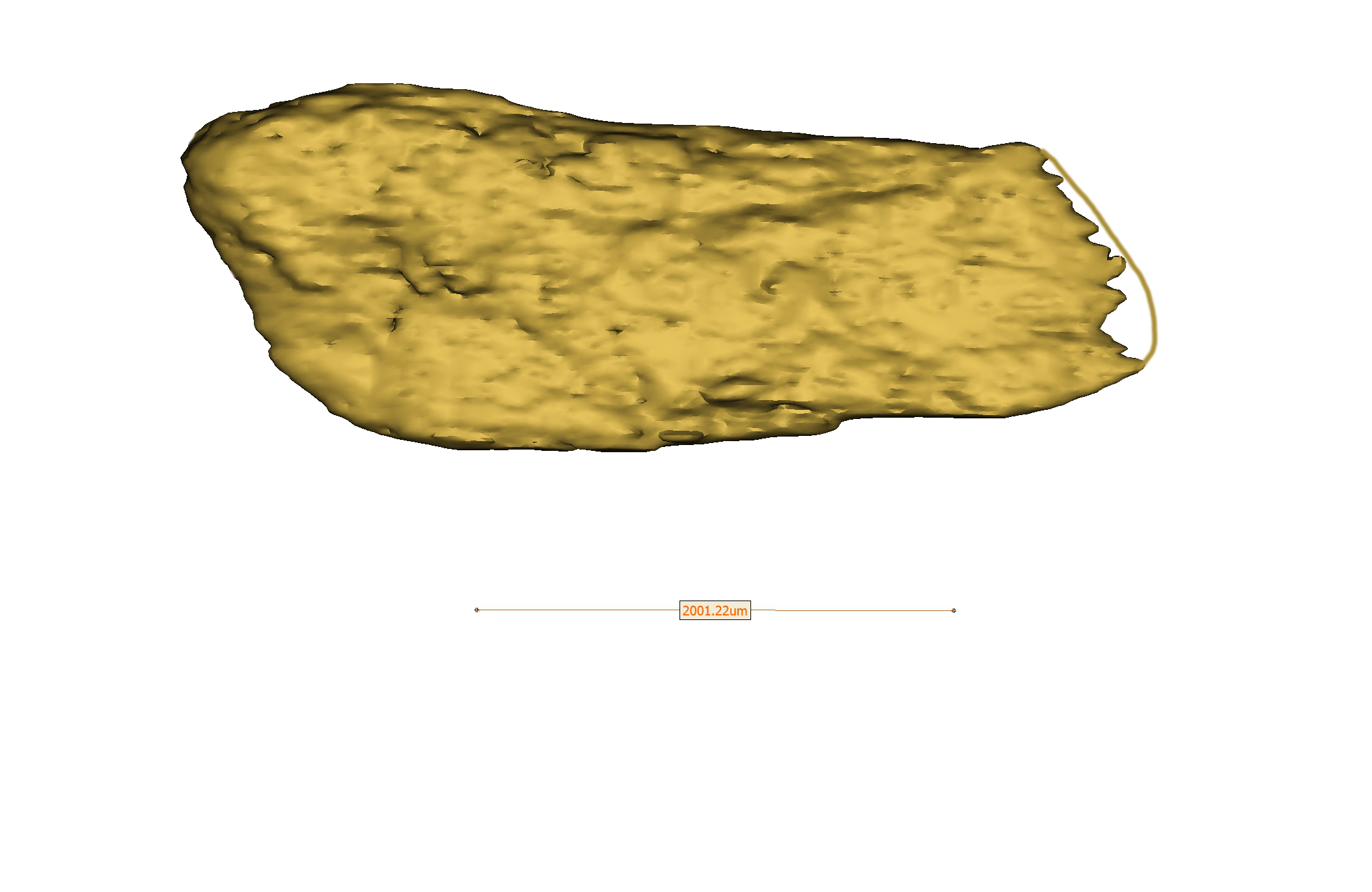

Supplement: Supplementary file 5 — Supplementary Data 2 [file 41467_2023_43557_MOESM5_ESM.zip › Supplementary Data 2/Supplementary Data 2 Raw data of Geometric Morphometric Analyses/12 Morphotypes/Morphotype 12/vf11l.jpg]

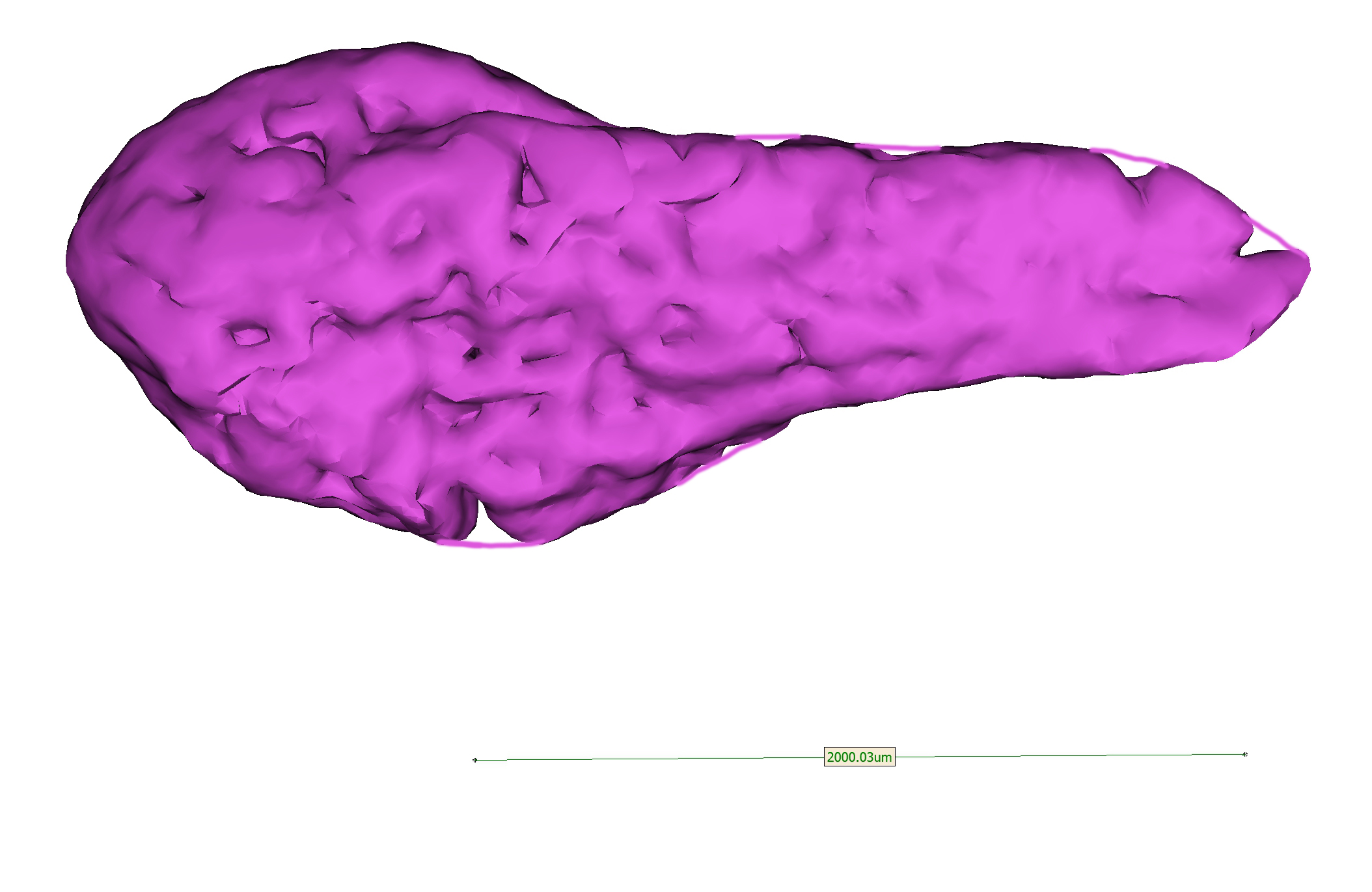

Supplement: Supplementary file 5 — Supplementary Data 2 [file 41467_2023_43557_MOESM5_ESM.zip › Supplementary Data 2/Supplementary Data 2 Raw data of Geometric Morphometric Analyses/12 Morphotypes/Morphotype 12/vf12l.jpg]

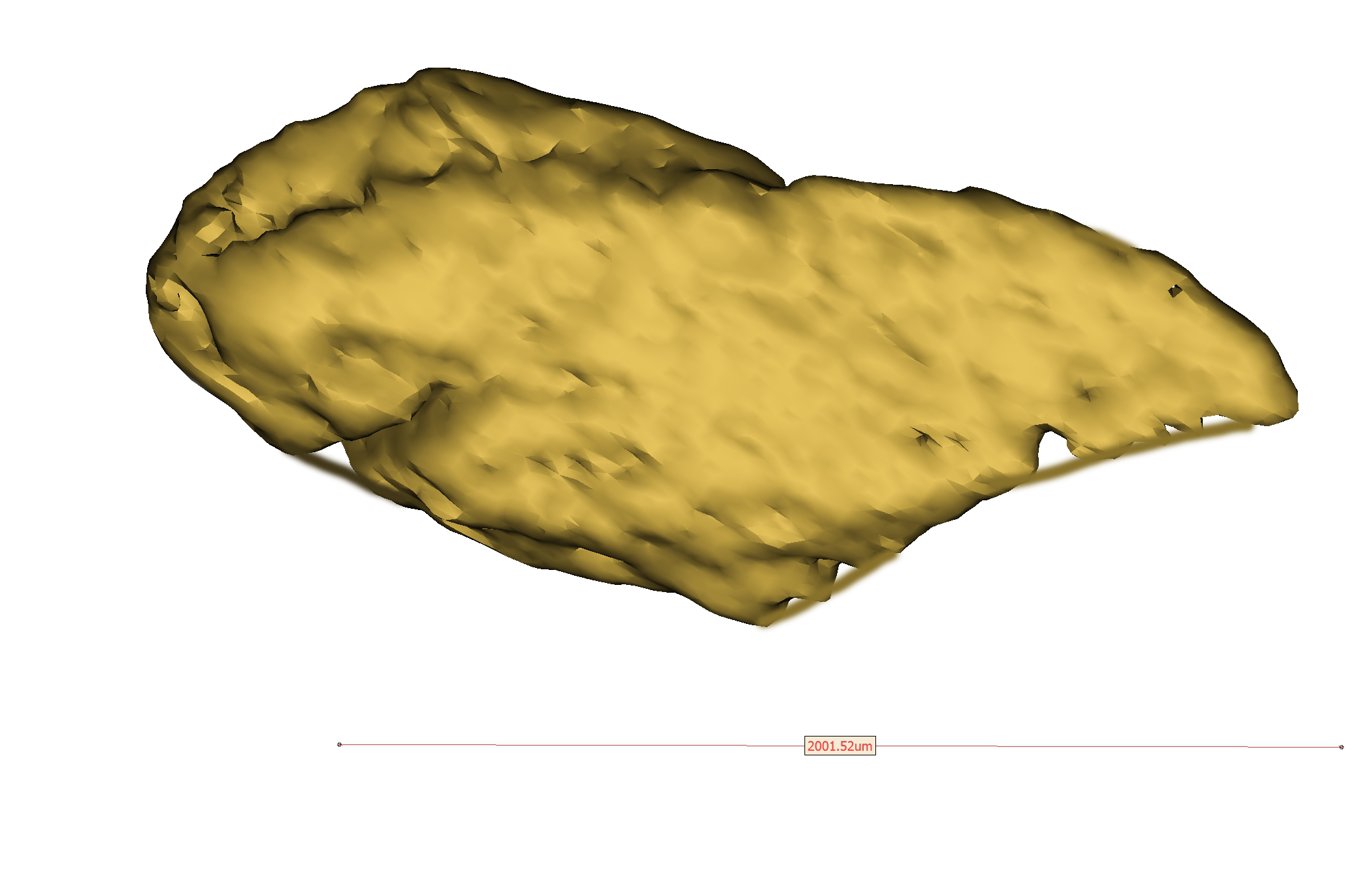

Supplement: Supplementary file 5 — Supplementary Data 2 [file 41467_2023_43557_MOESM5_ESM.zip › Supplementary Data 2/Supplementary Data 2 Raw data of Geometric Morphometric Analyses/12 Morphotypes/Morphotype 12/vf13l.jpg]

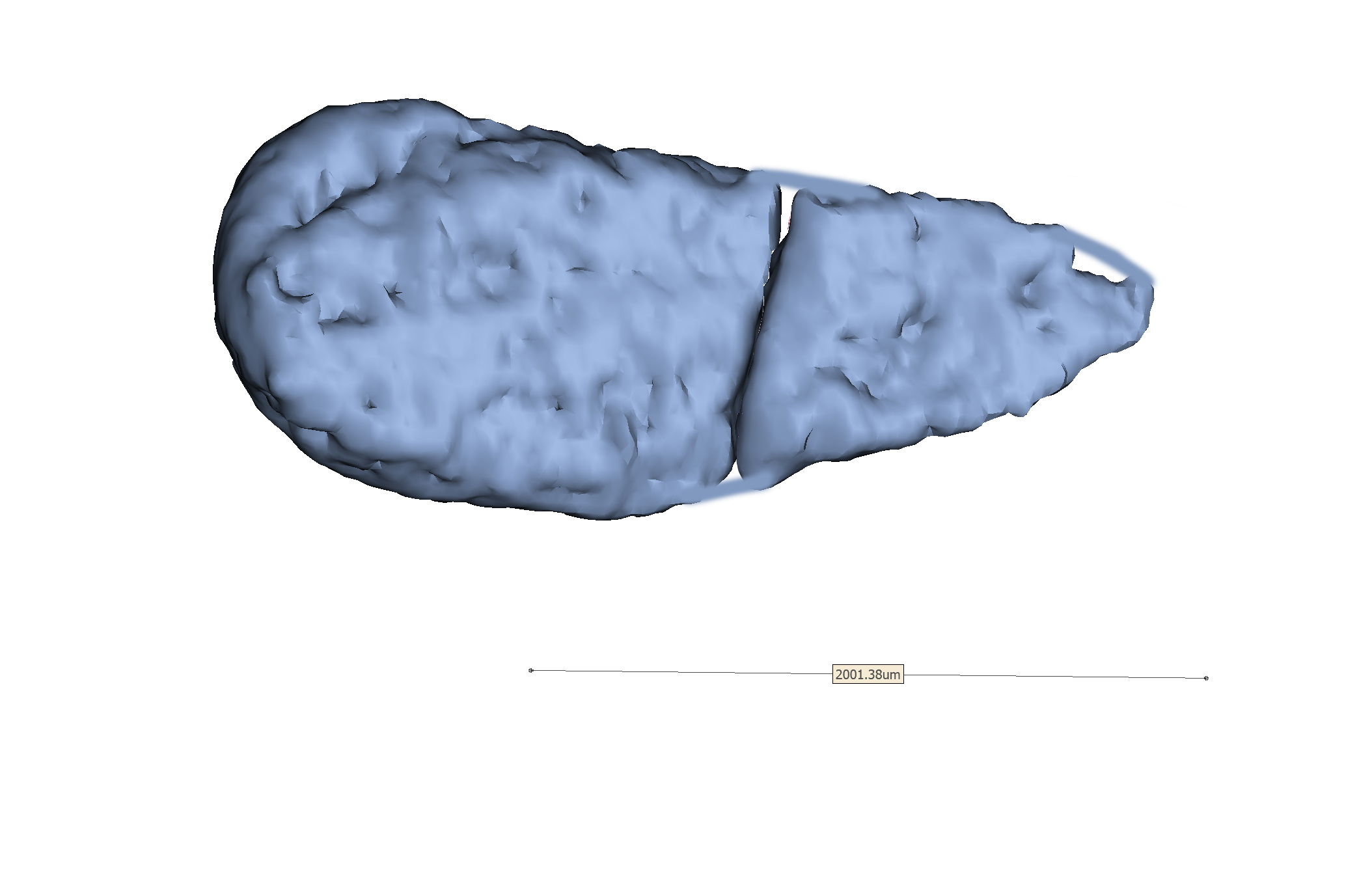

Supplement: Supplementary file 5 — Supplementary Data 2 [file 41467_2023_43557_MOESM5_ESM.zip › Supplementary Data 2/Supplementary Data 2 Raw data of Geometric Morphometric Analyses/12 Morphotypes/Morphotype 12/vf14l.jpg]

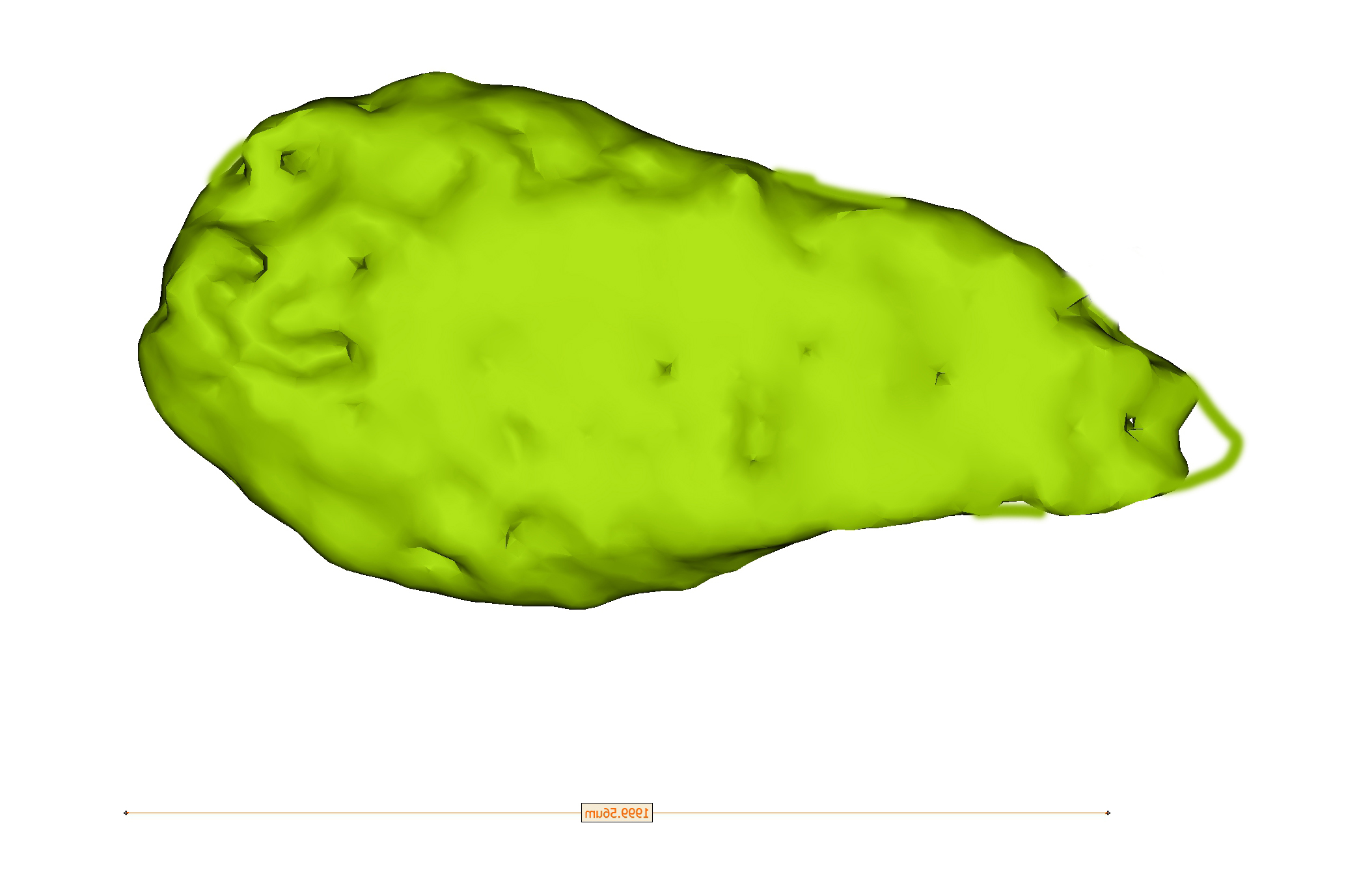

Supplement: Supplementary file 5 — Supplementary Data 2 [file 41467_2023_43557_MOESM5_ESM.zip › Supplementary Data 2/Supplementary Data 2 Raw data of Geometric Morphometric Analyses/12 Morphotypes/Morphotype 12/vf15r.jpg]

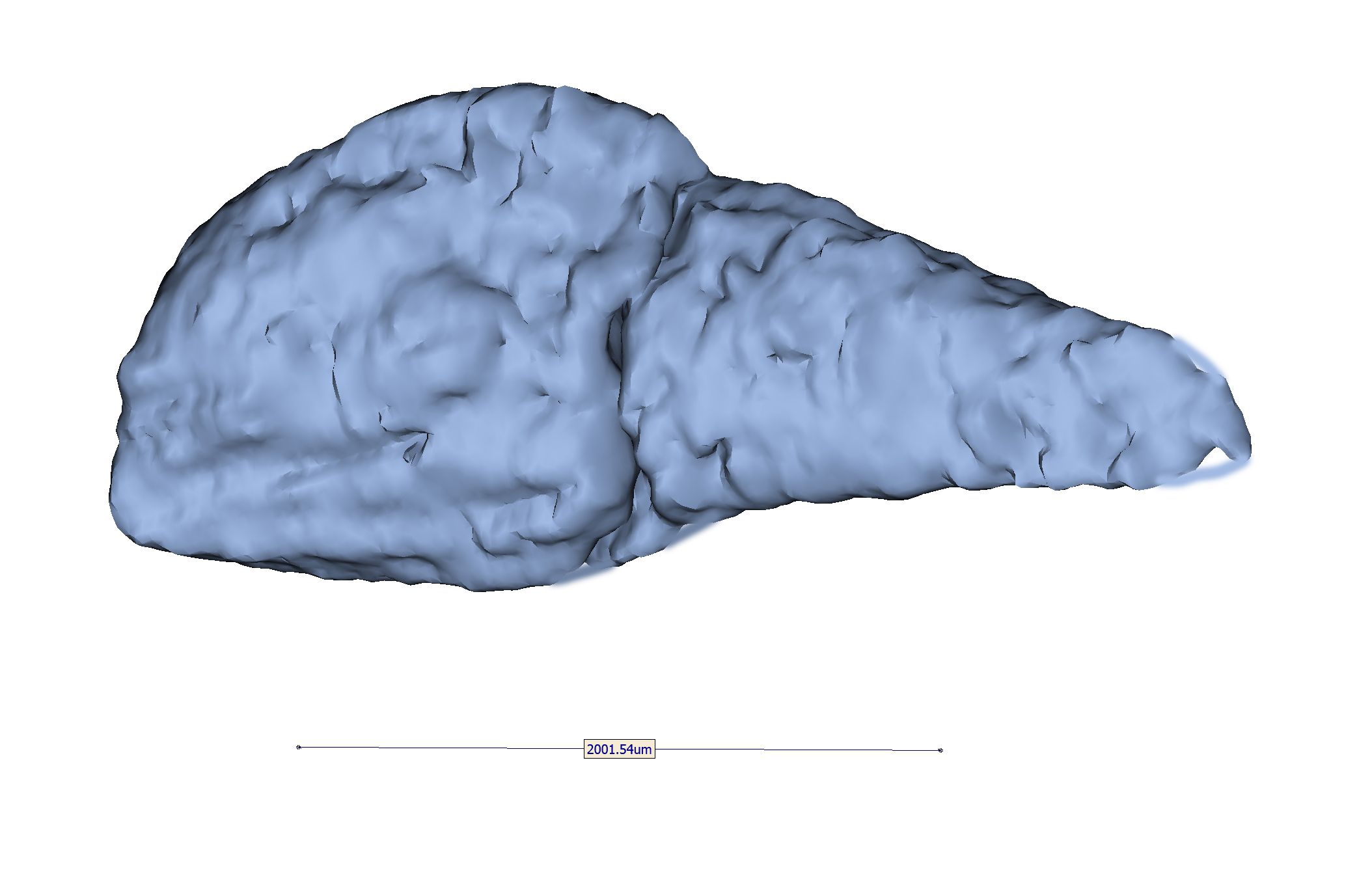

Supplement: Supplementary file 5 — Supplementary Data 2 [file 41467_2023_43557_MOESM5_ESM.zip › Supplementary Data 2/Supplementary Data 2 Raw data of Geometric Morphometric Analyses/12 Morphotypes/Morphotype 12/vf16l.jpg]

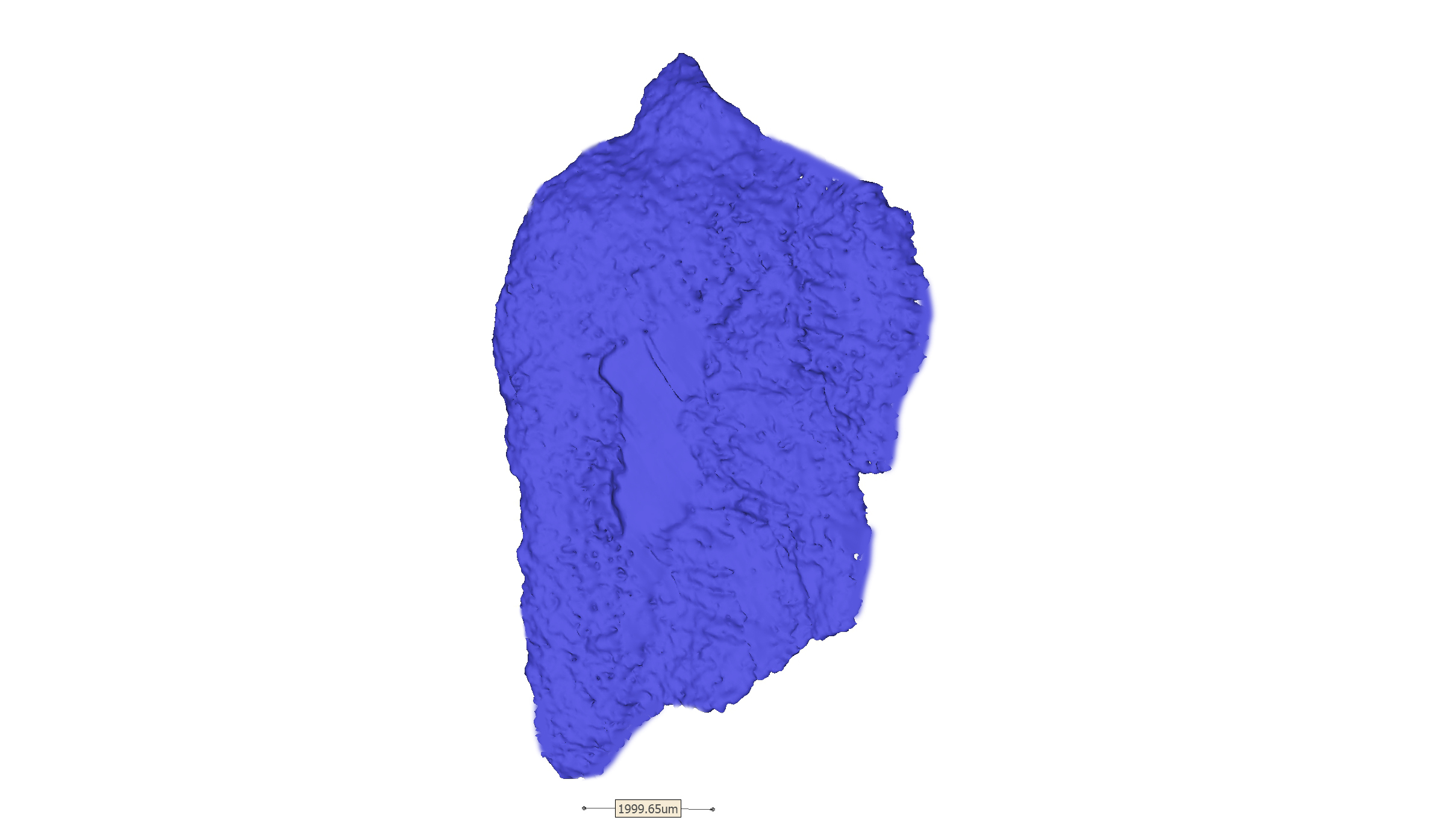

Supplement: Supplementary file 5 — Supplementary Data 2 [file 41467_2023_43557_MOESM5_ESM.zip › Supplementary Data 2/Supplementary Data 2 Raw data of Geometric Morphometric Analyses/12 Morphotypes/Morphotype 2/l1d01.jpg]

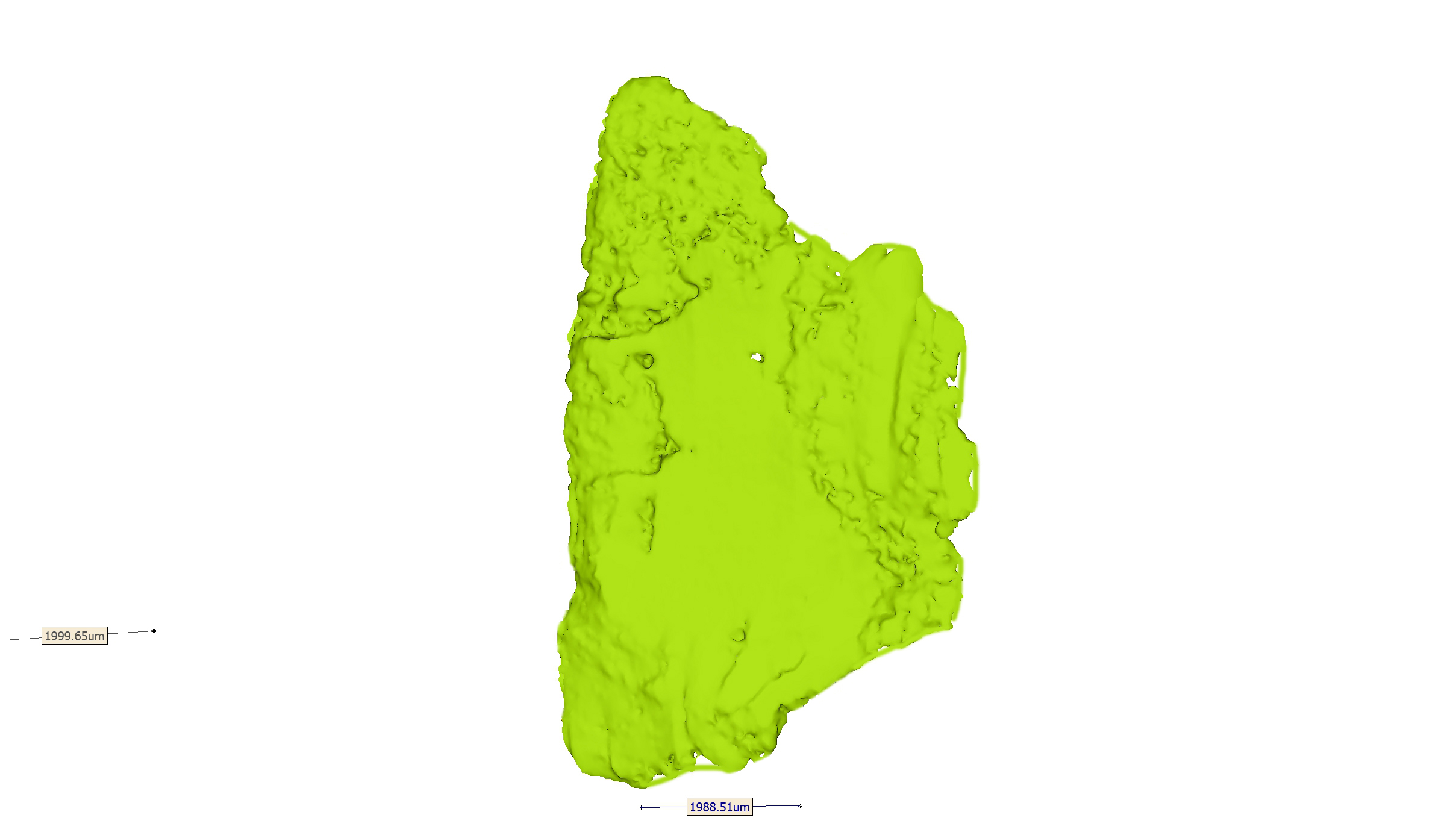

Supplement: Supplementary file 5 — Supplementary Data 2 [file 41467_2023_43557_MOESM5_ESM.zip › Supplementary Data 2/Supplementary Data 2 Raw data of Geometric Morphometric Analyses/12 Morphotypes/Morphotype 2/l1d02.jpg]

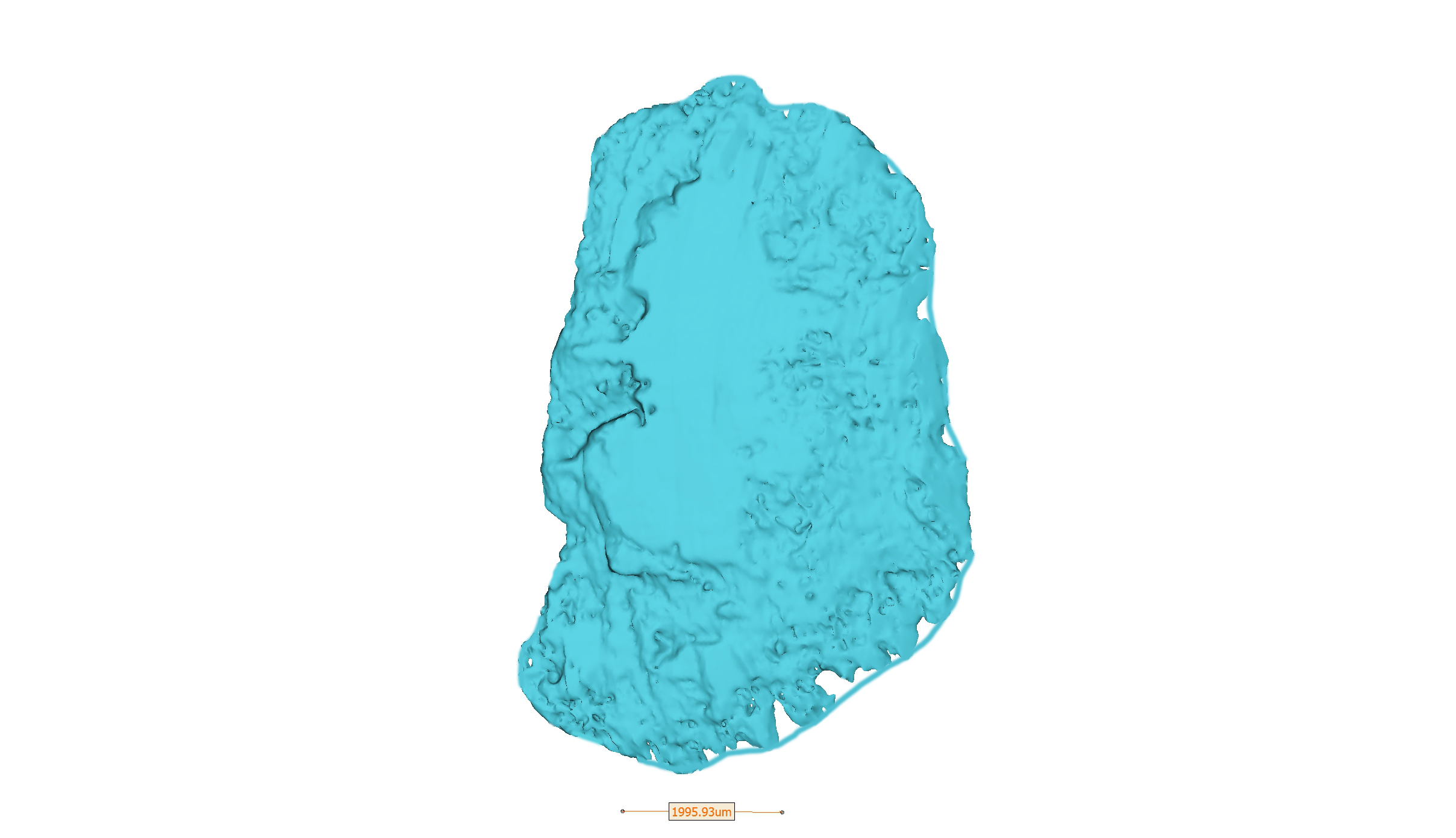

Supplement: Supplementary file 5 — Supplementary Data 2 [file 41467_2023_43557_MOESM5_ESM.zip › Supplementary Data 2/Supplementary Data 2 Raw data of Geometric Morphometric Analyses/12 Morphotypes/Morphotype 2/l1d03.jpg]

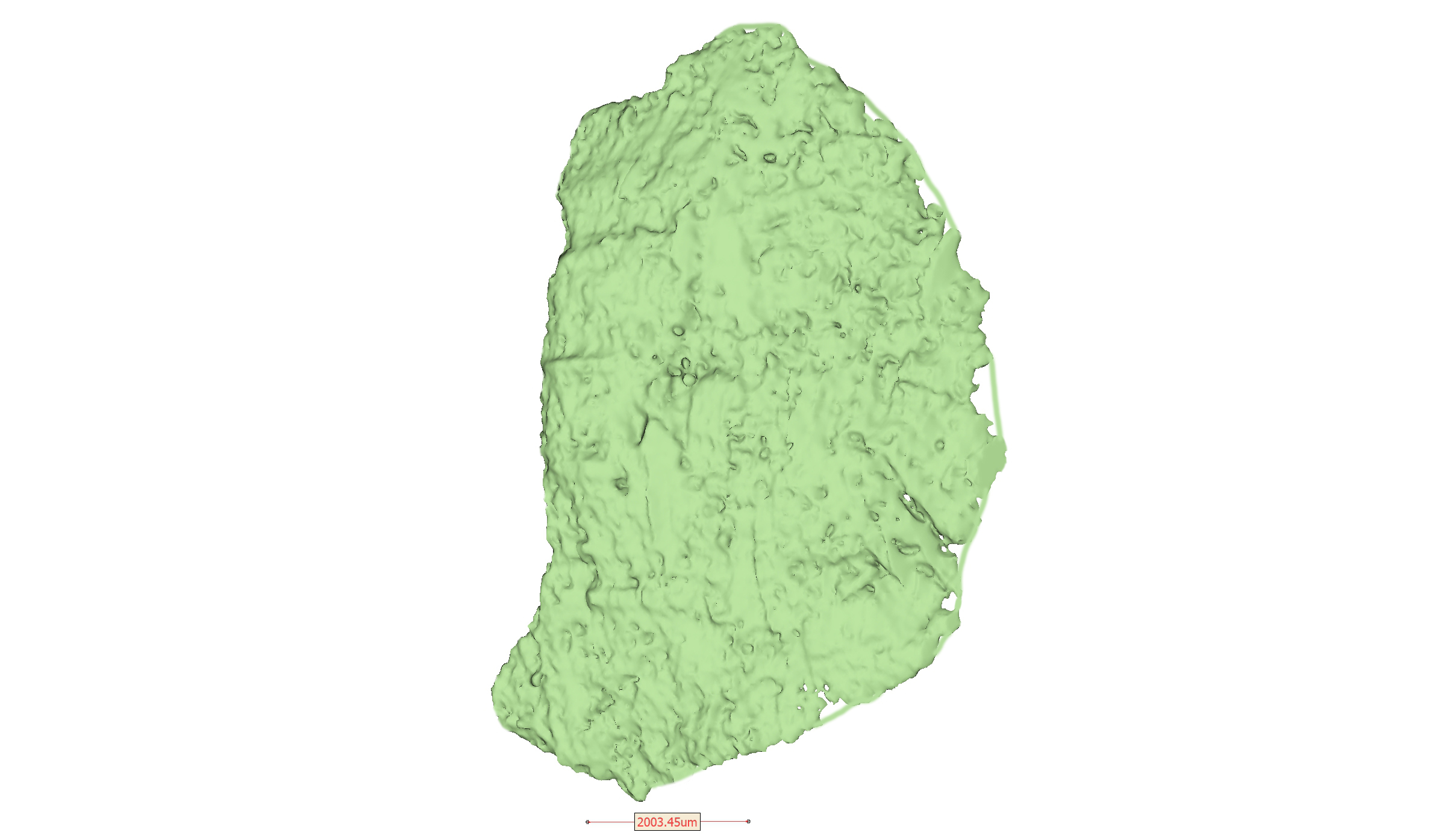

Supplement: Supplementary file 5 — Supplementary Data 2 [file 41467_2023_43557_MOESM5_ESM.zip › Supplementary Data 2/Supplementary Data 2 Raw data of Geometric Morphometric Analyses/12 Morphotypes/Morphotype 2/l1d04.jpg]

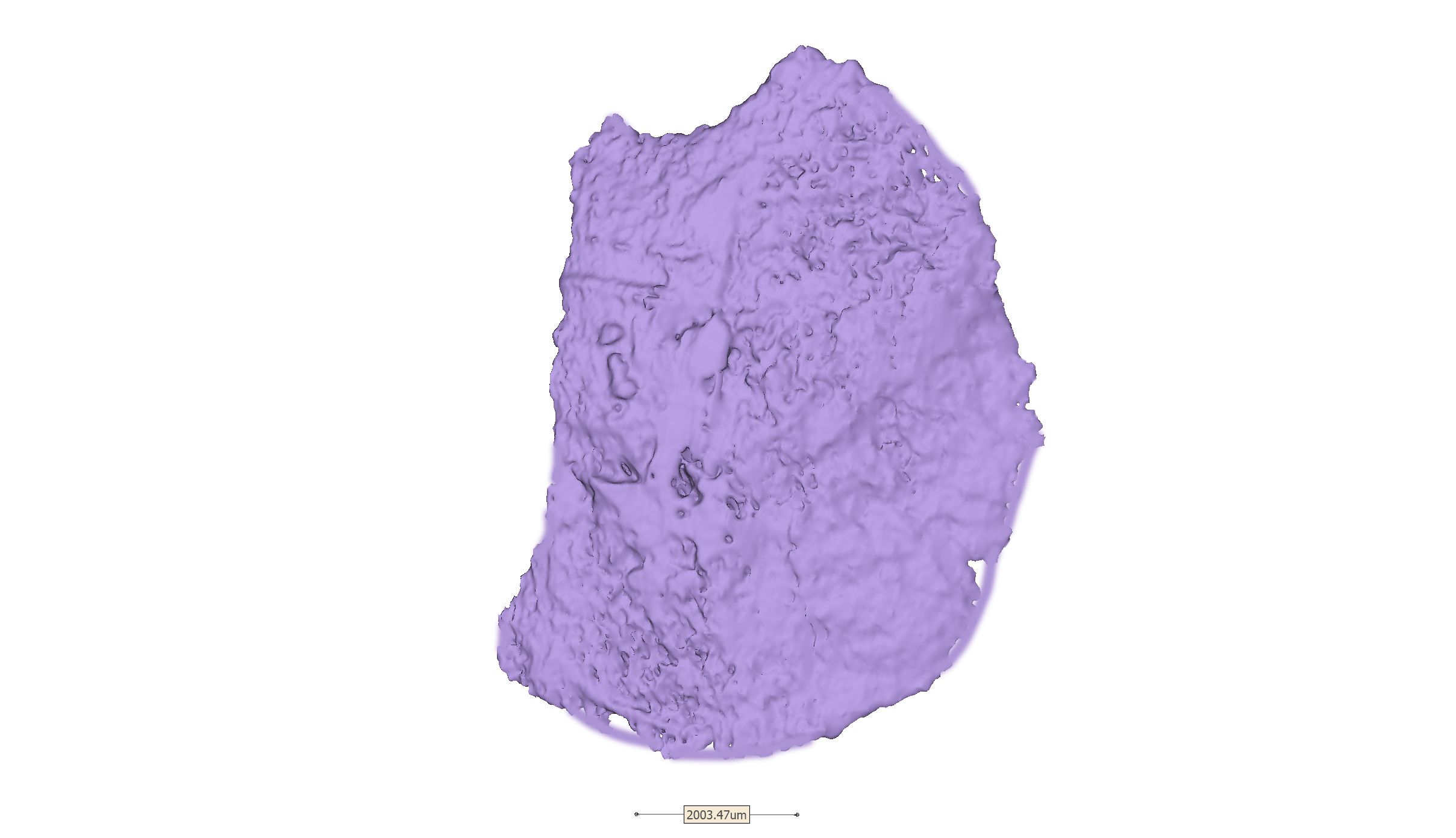

Supplement: Supplementary file 5 — Supplementary Data 2 [file 41467_2023_43557_MOESM5_ESM.zip › Supplementary Data 2/Supplementary Data 2 Raw data of Geometric Morphometric Analyses/12 Morphotypes/Morphotype 2/l1d05.jpg]

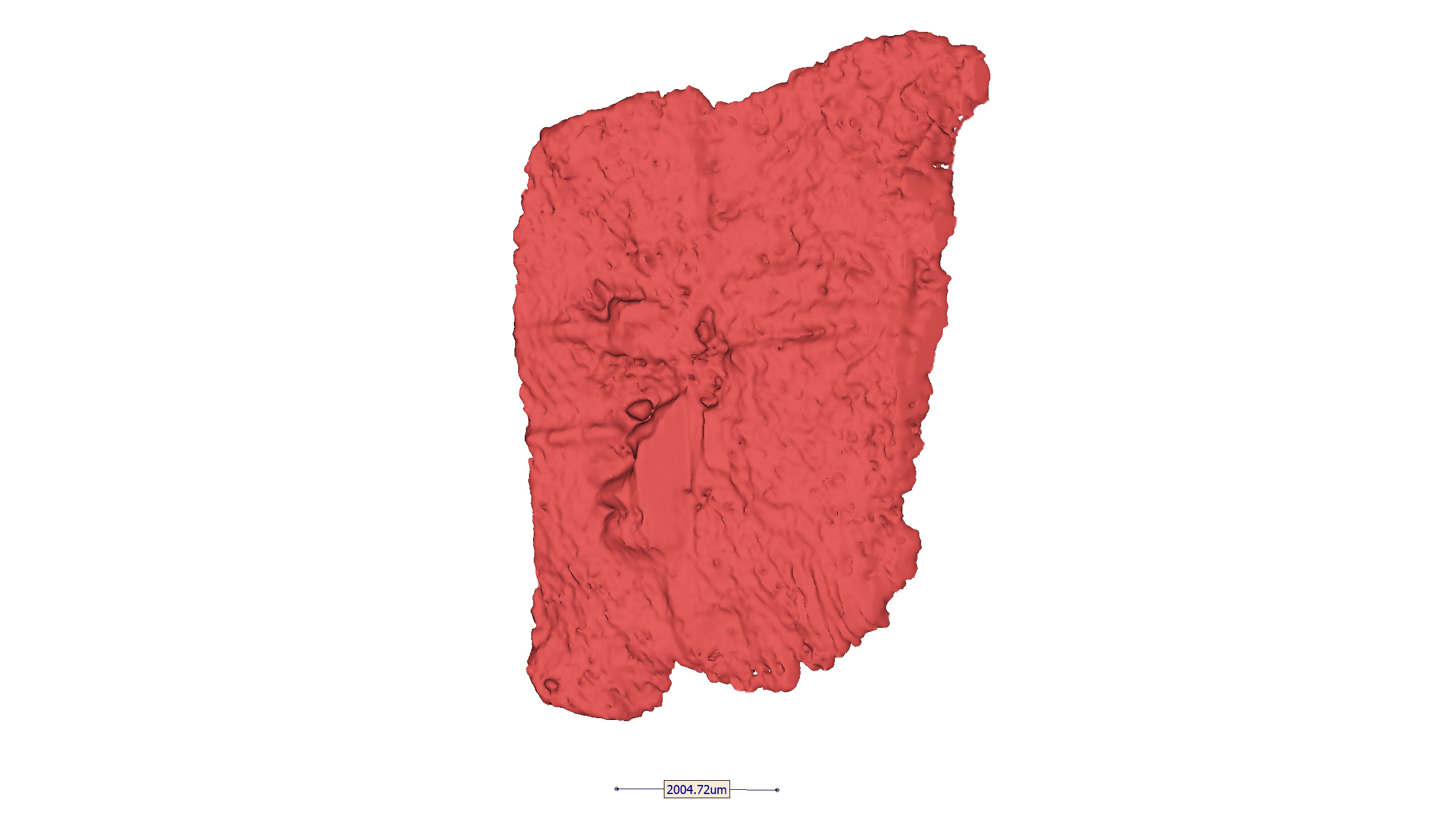

Supplement: Supplementary file 5 — Supplementary Data 2 [file 41467_2023_43557_MOESM5_ESM.zip › Supplementary Data 2/Supplementary Data 2 Raw data of Geometric Morphometric Analyses/12 Morphotypes/Morphotype 2/l1v02.jpg]

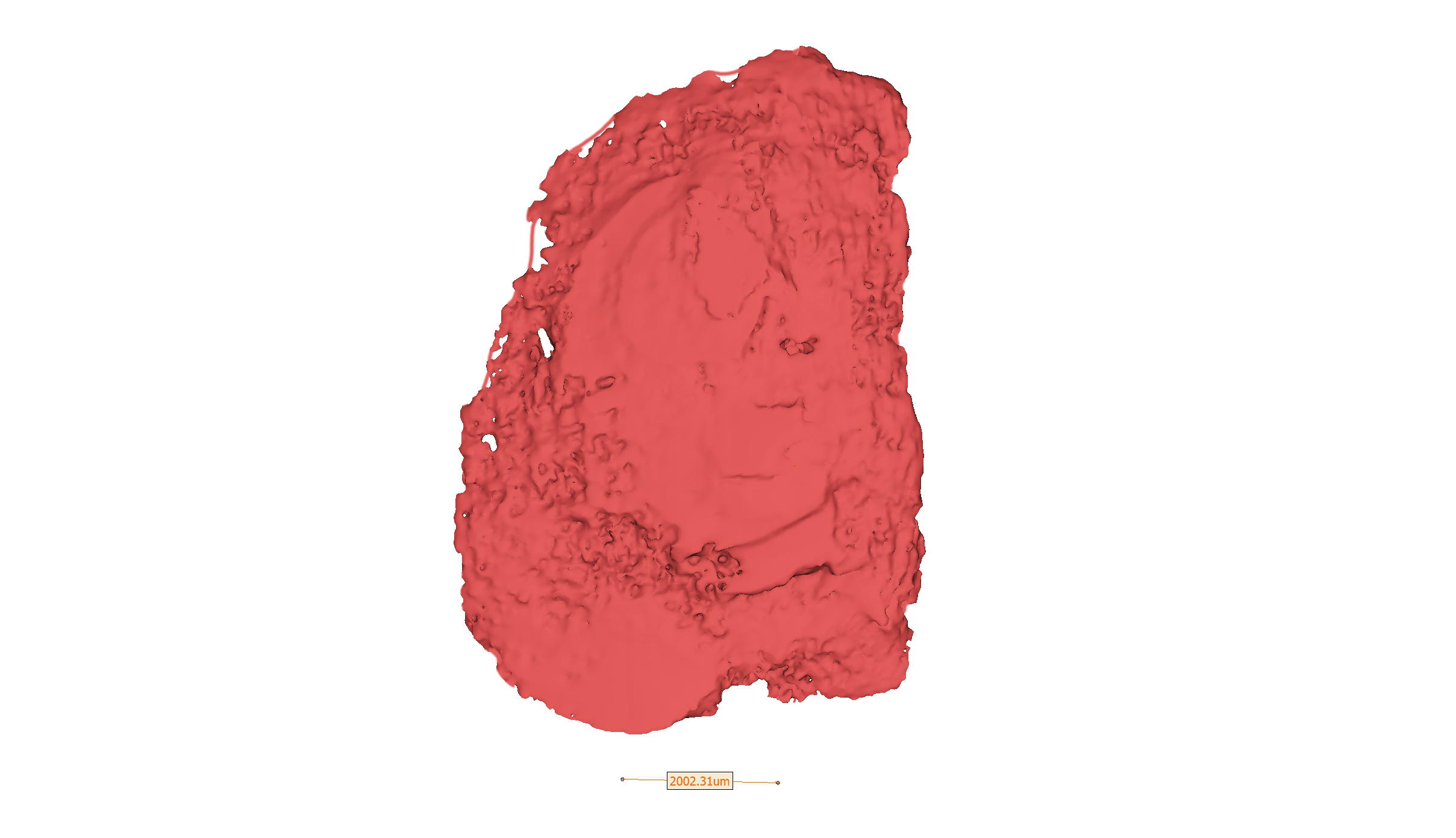

Supplement: Supplementary file 5 — Supplementary Data 2 [file 41467_2023_43557_MOESM5_ESM.zip › Supplementary Data 2/Supplementary Data 2 Raw data of Geometric Morphometric Analyses/12 Morphotypes/Morphotype 2/l1v03.jpg]

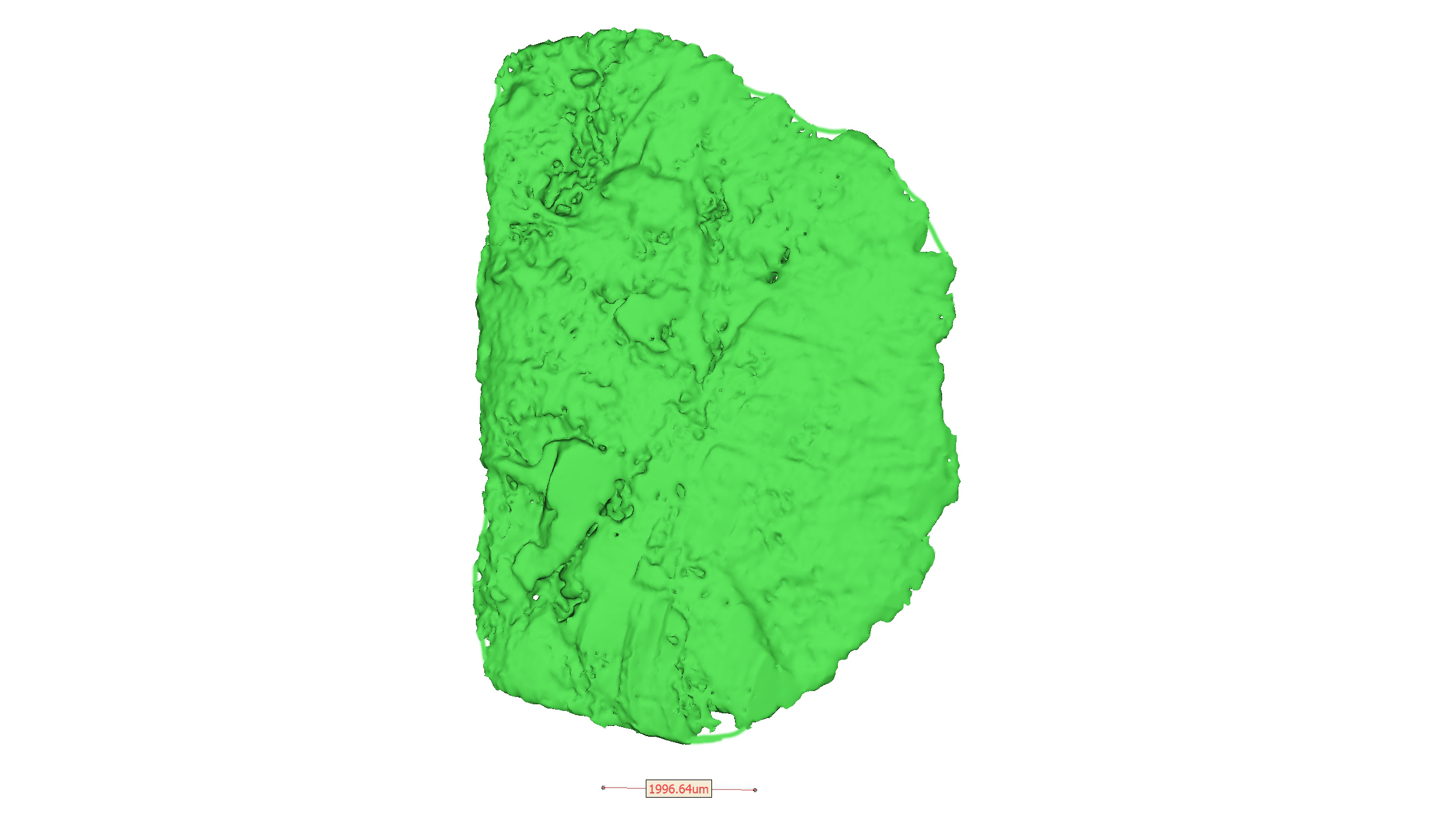

Supplement: Supplementary file 5 — Supplementary Data 2 [file 41467_2023_43557_MOESM5_ESM.zip › Supplementary Data 2/Supplementary Data 2 Raw data of Geometric Morphometric Analyses/12 Morphotypes/Morphotype 2/l1v05.jpg]

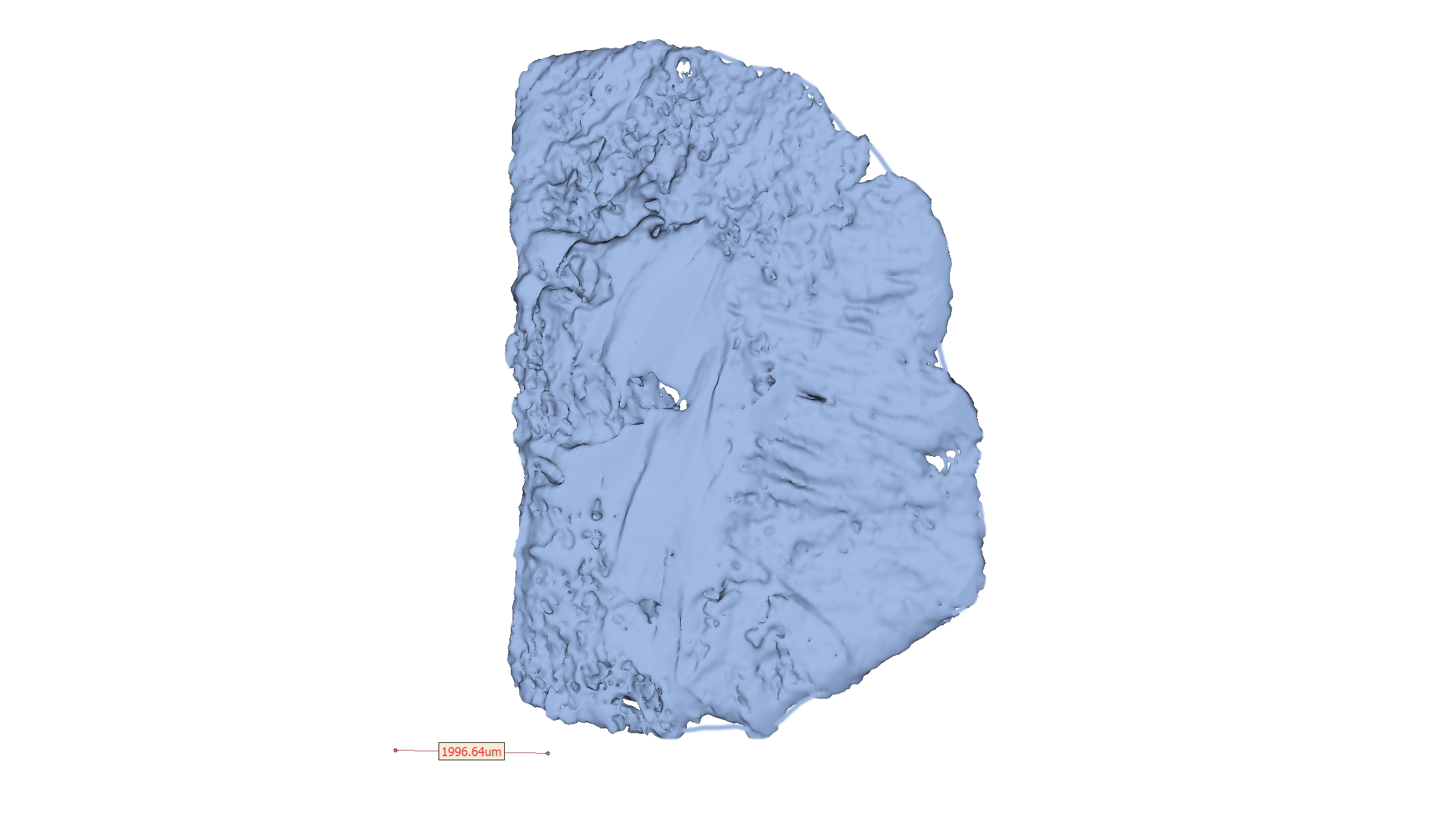

Supplement: Supplementary file 5 — Supplementary Data 2 [file 41467_2023_43557_MOESM5_ESM.zip › Supplementary Data 2/Supplementary Data 2 Raw data of Geometric Morphometric Analyses/12 Morphotypes/Morphotype 2/l1v06.jpg]

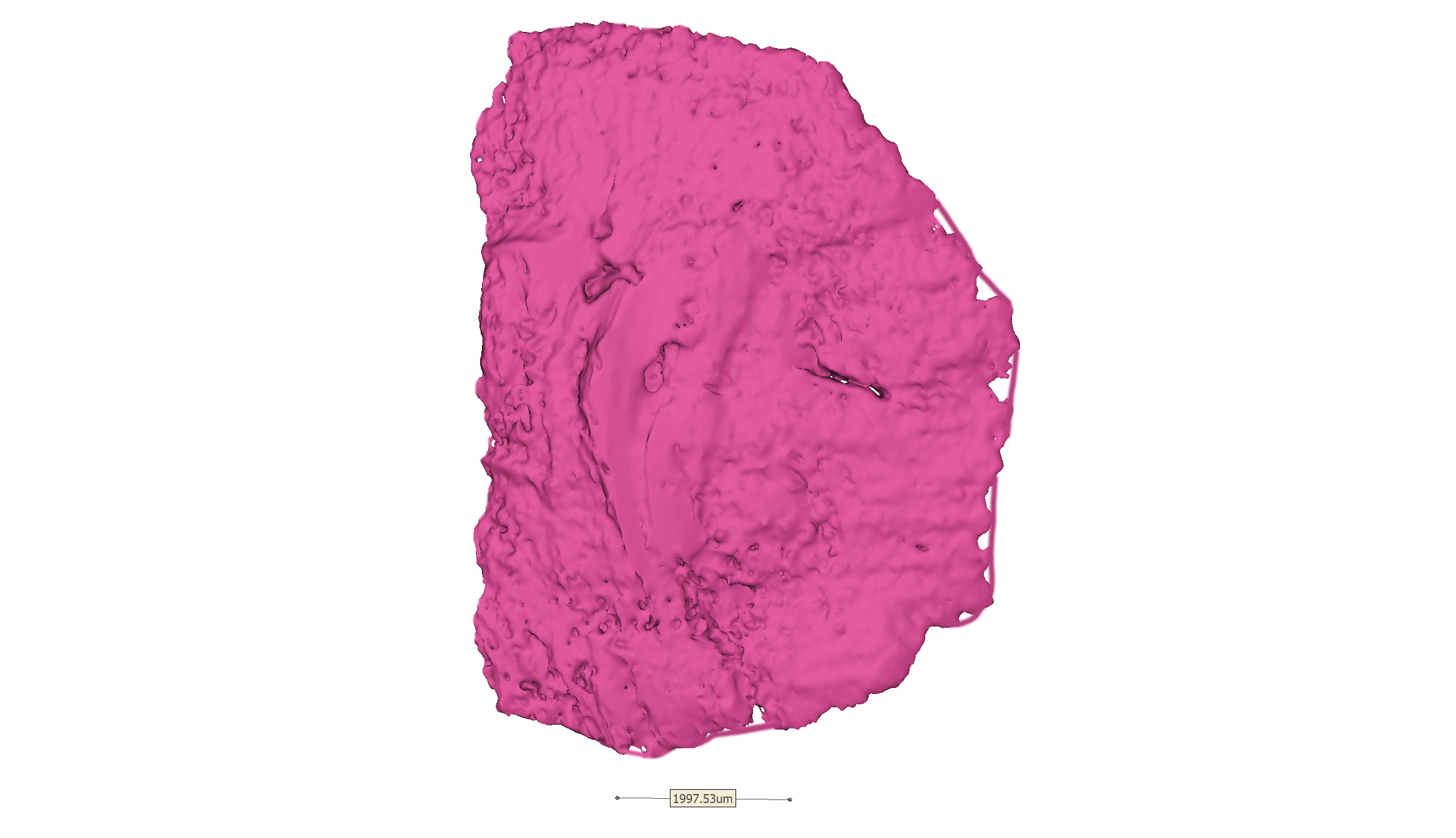

Supplement: Supplementary file 5 — Supplementary Data 2 [file 41467_2023_43557_MOESM5_ESM.zip › Supplementary Data 2/Supplementary Data 2 Raw data of Geometric Morphometric Analyses/12 Morphotypes/Morphotype 2/l1v08.jpg]

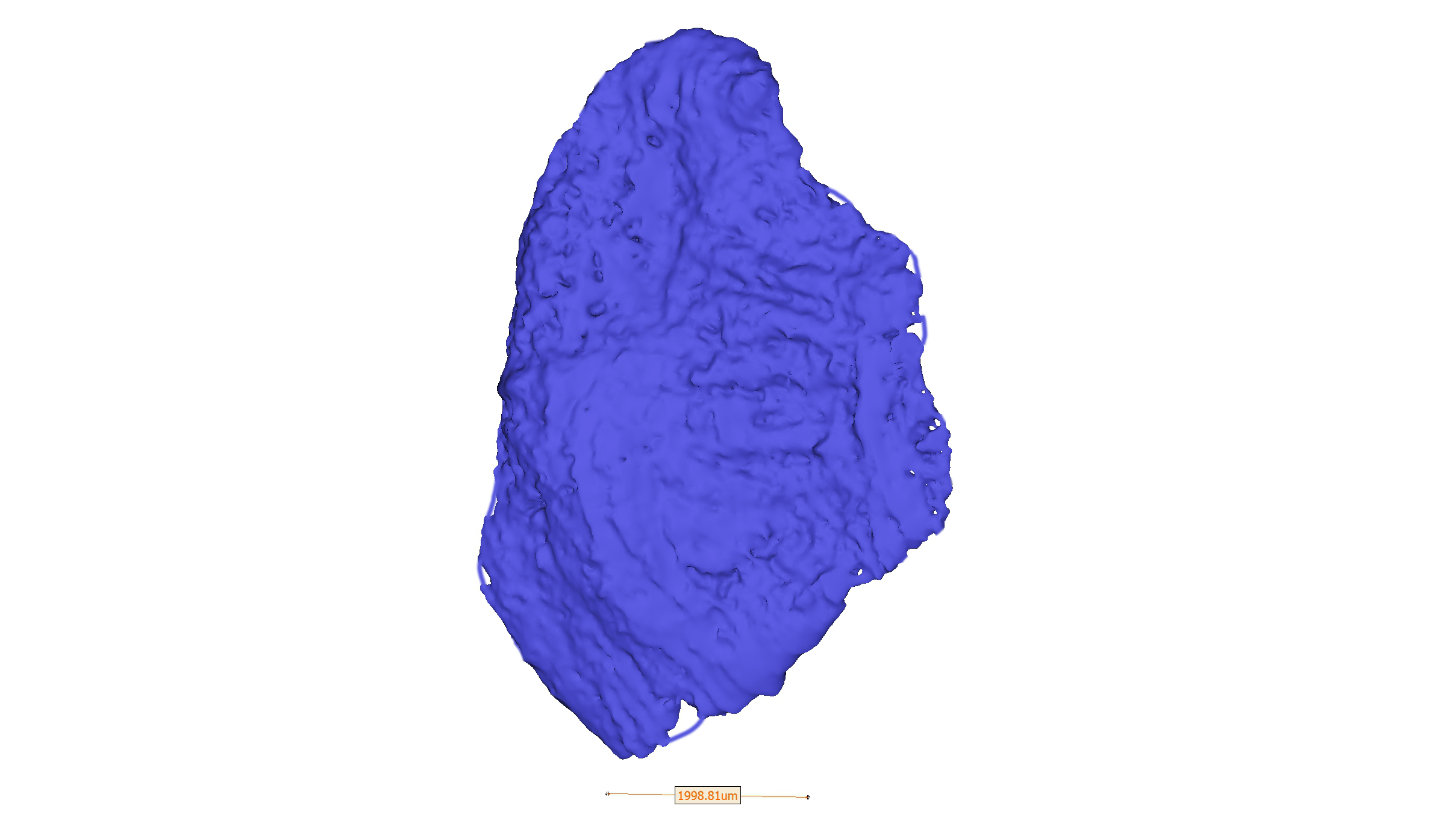

Supplement: Supplementary file 5 — Supplementary Data 2 [file 41467_2023_43557_MOESM5_ESM.zip › Supplementary Data 2/Supplementary Data 2 Raw data of Geometric Morphometric Analyses/12 Morphotypes/Morphotype 2/l2d02.jpg]

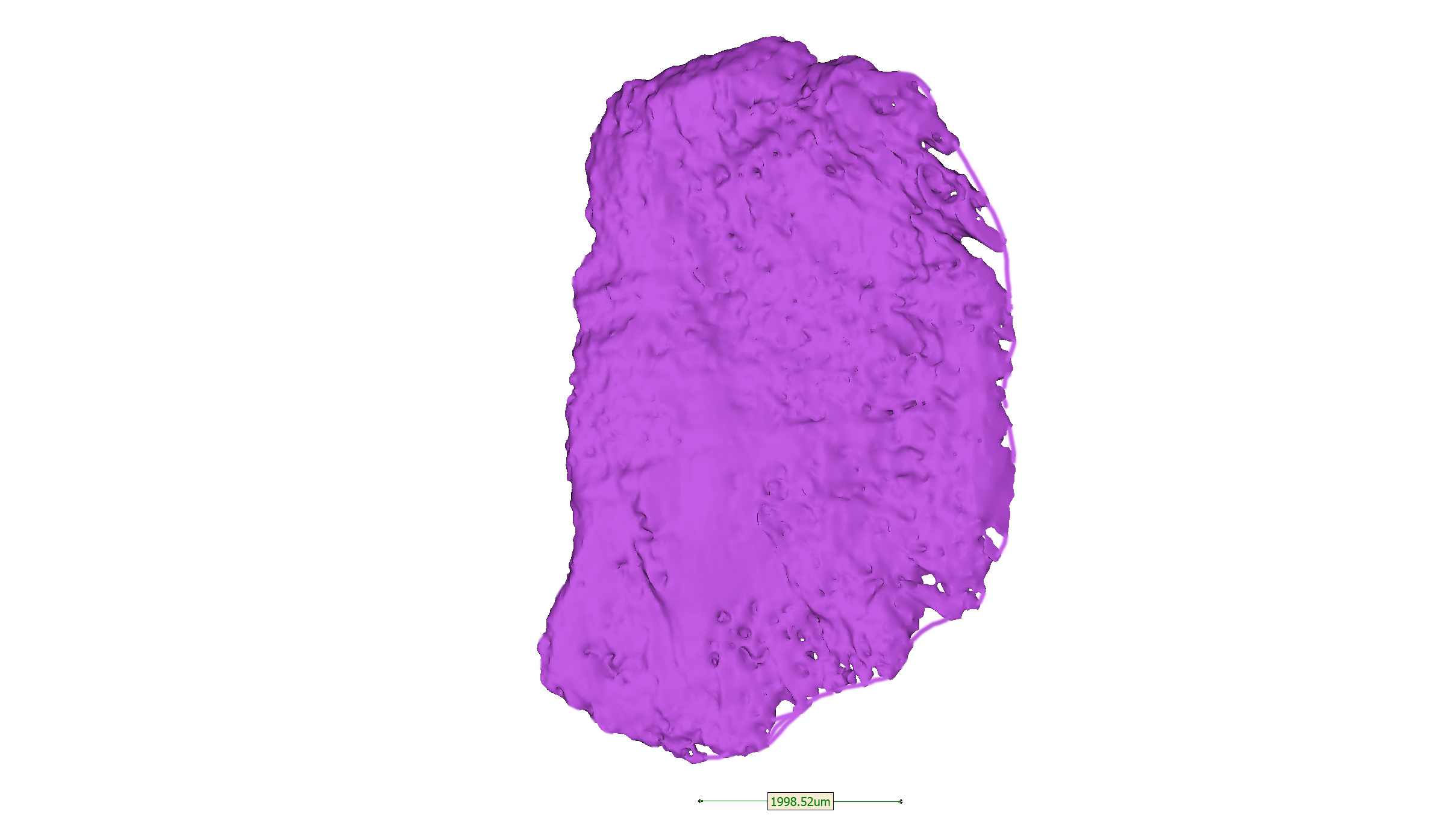

Supplement: Supplementary file 5 — Supplementary Data 2 [file 41467_2023_43557_MOESM5_ESM.zip › Supplementary Data 2/Supplementary Data 2 Raw data of Geometric Morphometric Analyses/12 Morphotypes/Morphotype 2/l2d03.jpg]

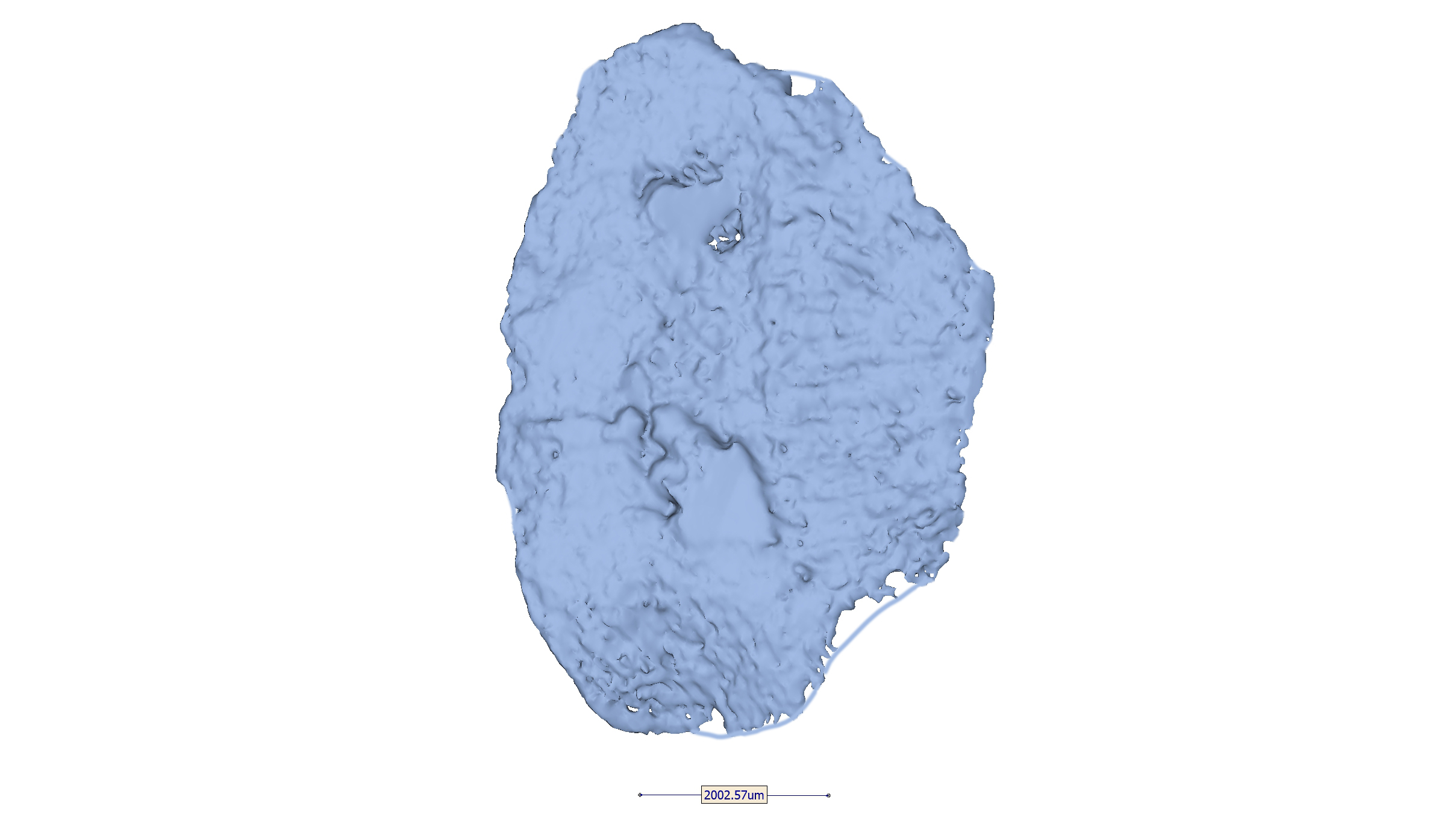

Supplement: Supplementary file 5 — Supplementary Data 2 [file 41467_2023_43557_MOESM5_ESM.zip › Supplementary Data 2/Supplementary Data 2 Raw data of Geometric Morphometric Analyses/12 Morphotypes/Morphotype 2/l2v01.jpg]

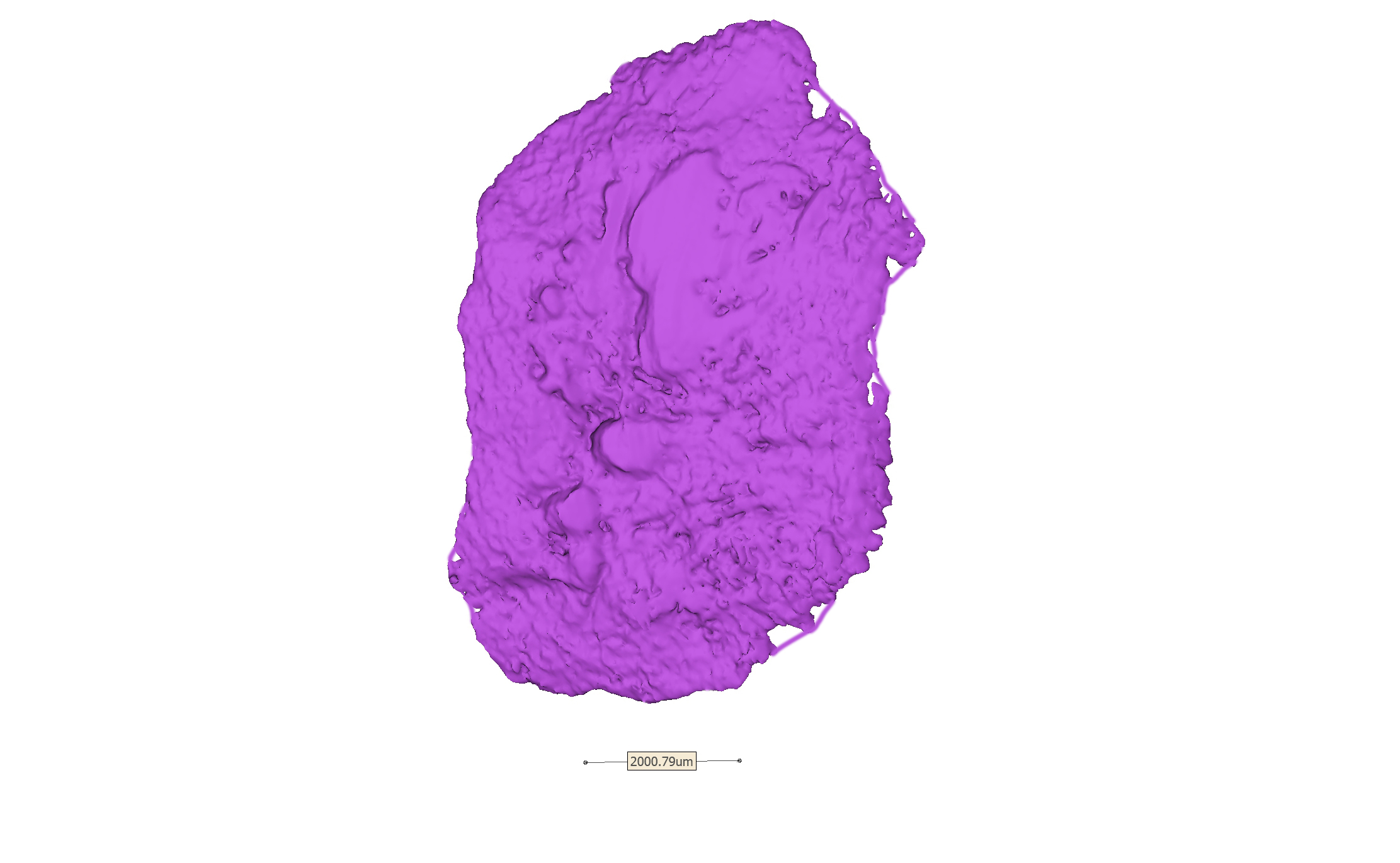

Supplement: Supplementary file 5 — Supplementary Data 2 [file 41467_2023_43557_MOESM5_ESM.zip › Supplementary Data 2/Supplementary Data 2 Raw data of Geometric Morphometric Analyses/12 Morphotypes/Morphotype 2/ll01.jpg]

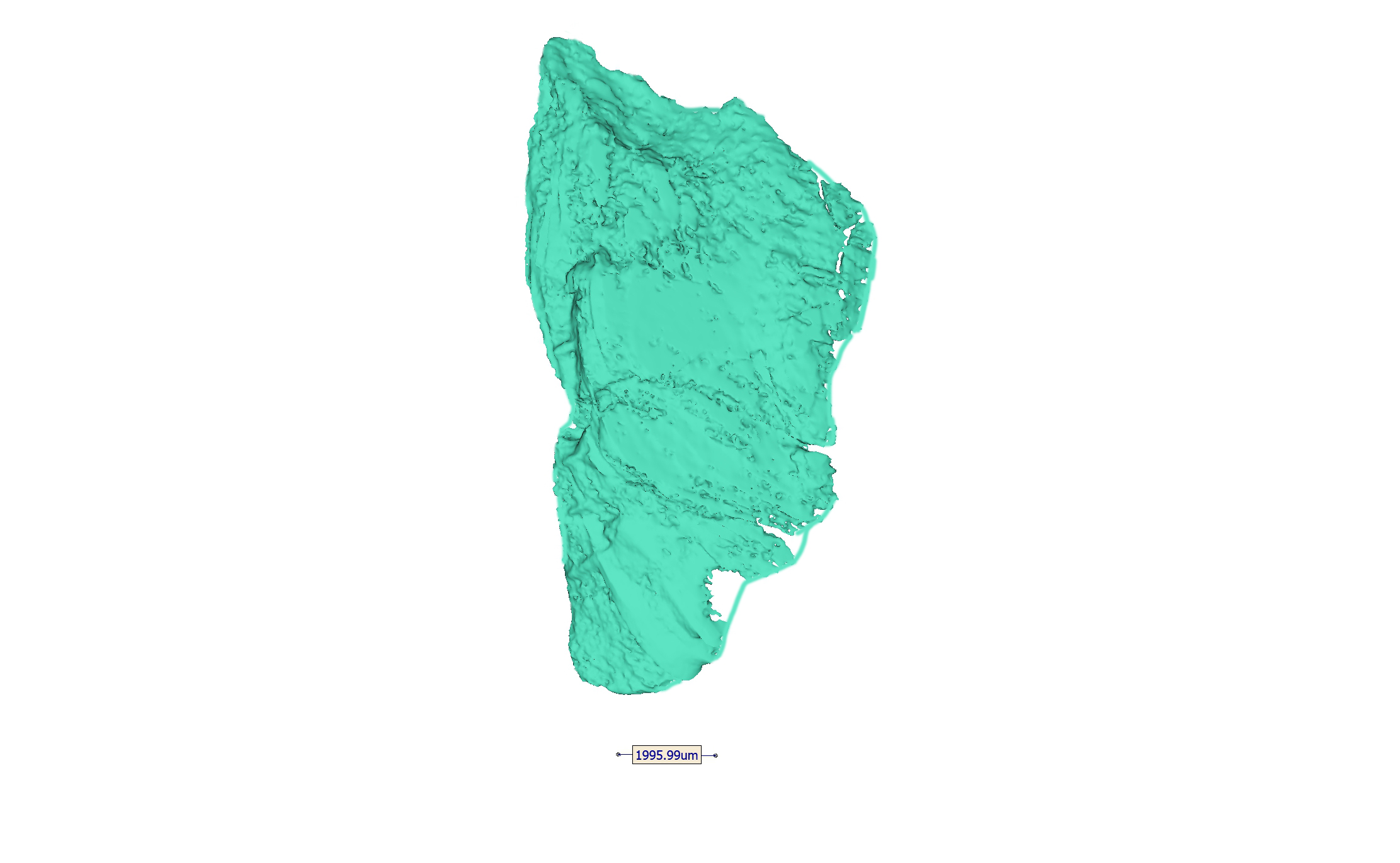

Supplement: Supplementary file 5 — Supplementary Data 2 [file 41467_2023_43557_MOESM5_ESM.zip › Supplementary Data 2/Supplementary Data 2 Raw data of Geometric Morphometric Analyses/12 Morphotypes/Morphotype 2/ll02.jpg]

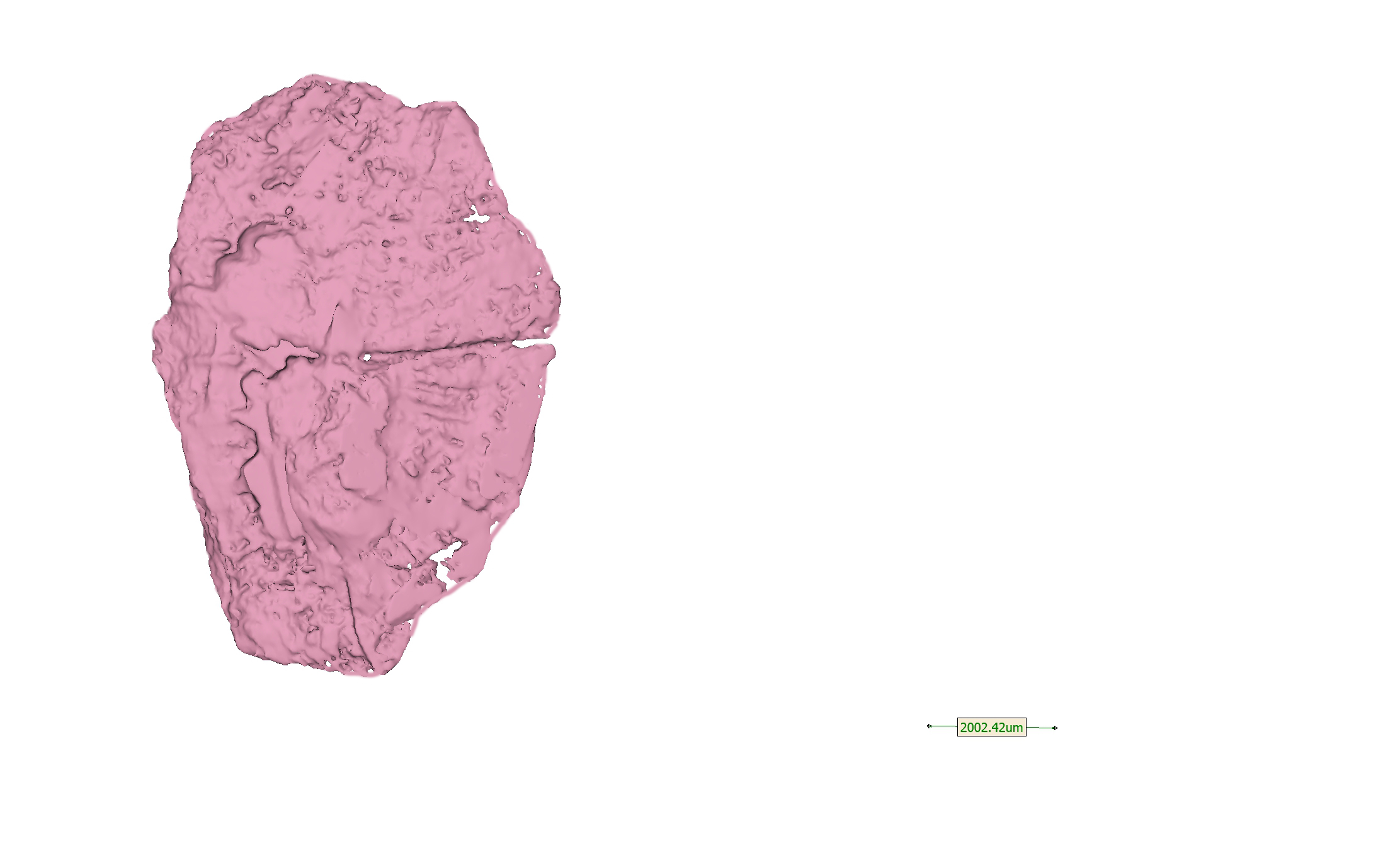

Supplement: Supplementary file 5 — Supplementary Data 2 [file 41467_2023_43557_MOESM5_ESM.zip › Supplementary Data 2/Supplementary Data 2 Raw data of Geometric Morphometric Analyses/12 Morphotypes/Morphotype 2/ll03.jpg]

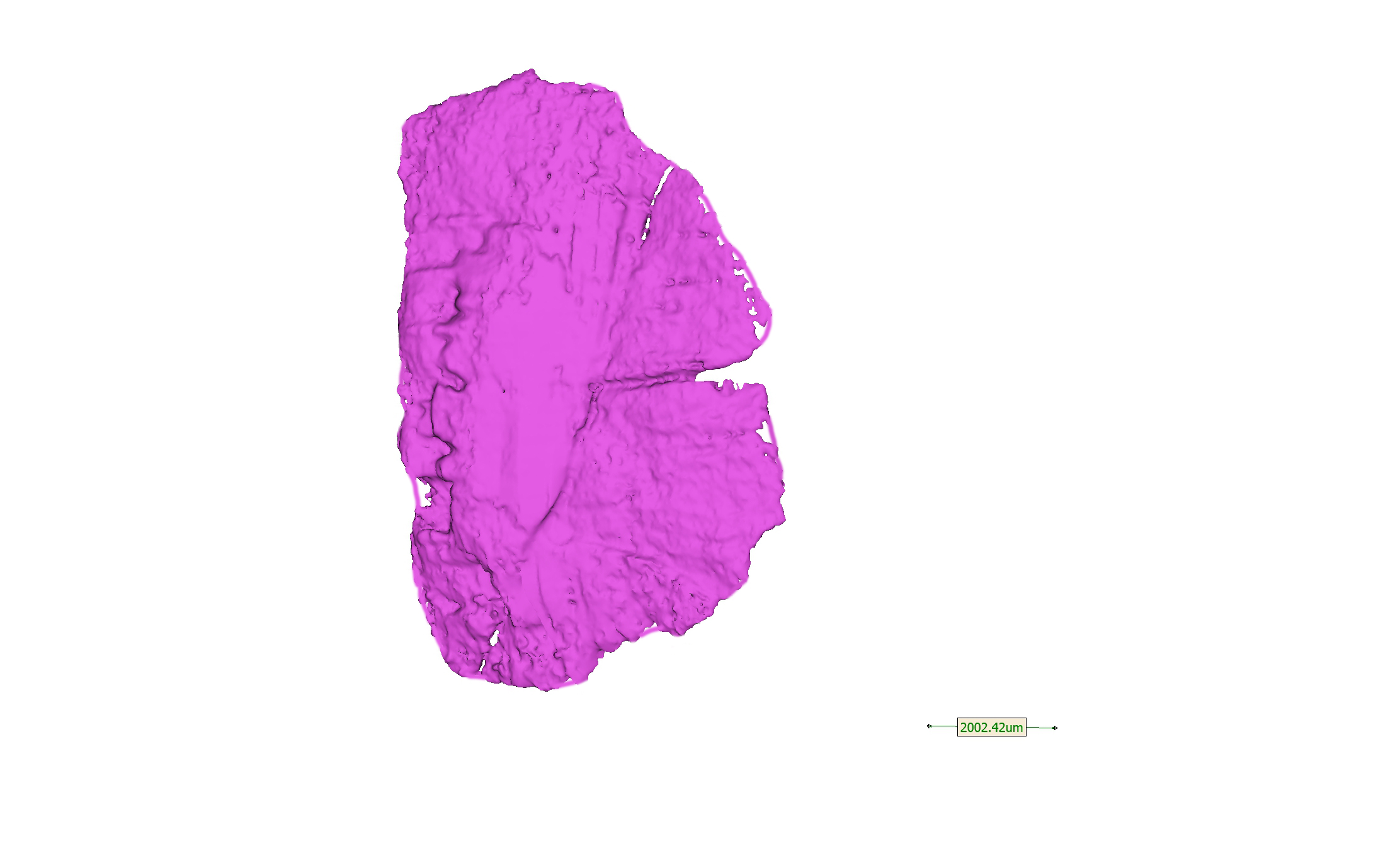

Supplement: Supplementary file 5 — Supplementary Data 2 [file 41467_2023_43557_MOESM5_ESM.zip › Supplementary Data 2/Supplementary Data 2 Raw data of Geometric Morphometric Analyses/12 Morphotypes/Morphotype 2/ll04.jpg]

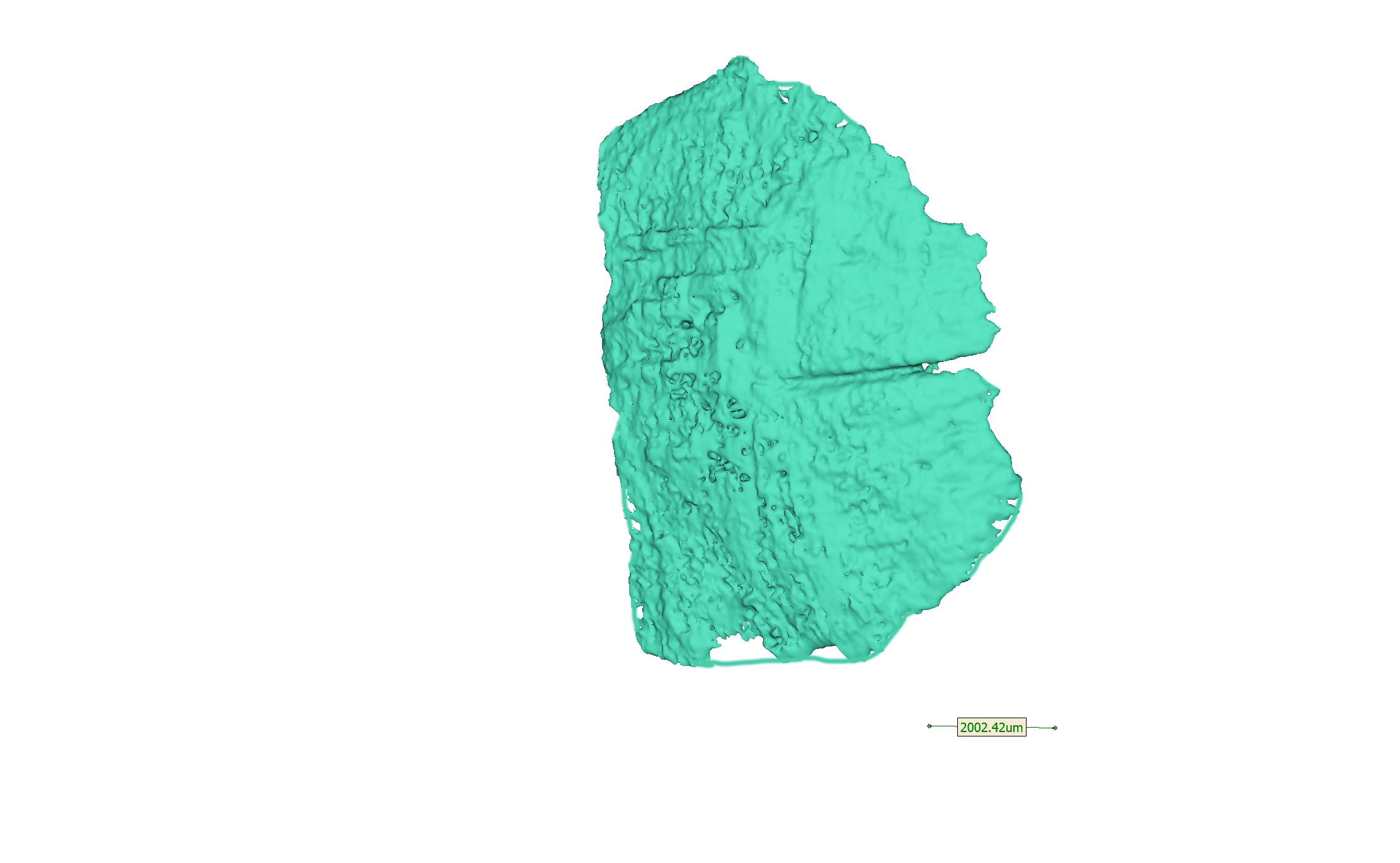

Supplement: Supplementary file 5 — Supplementary Data 2 [file 41467_2023_43557_MOESM5_ESM.zip › Supplementary Data 2/Supplementary Data 2 Raw data of Geometric Morphometric Analyses/12 Morphotypes/Morphotype 2/ll05.jpg]

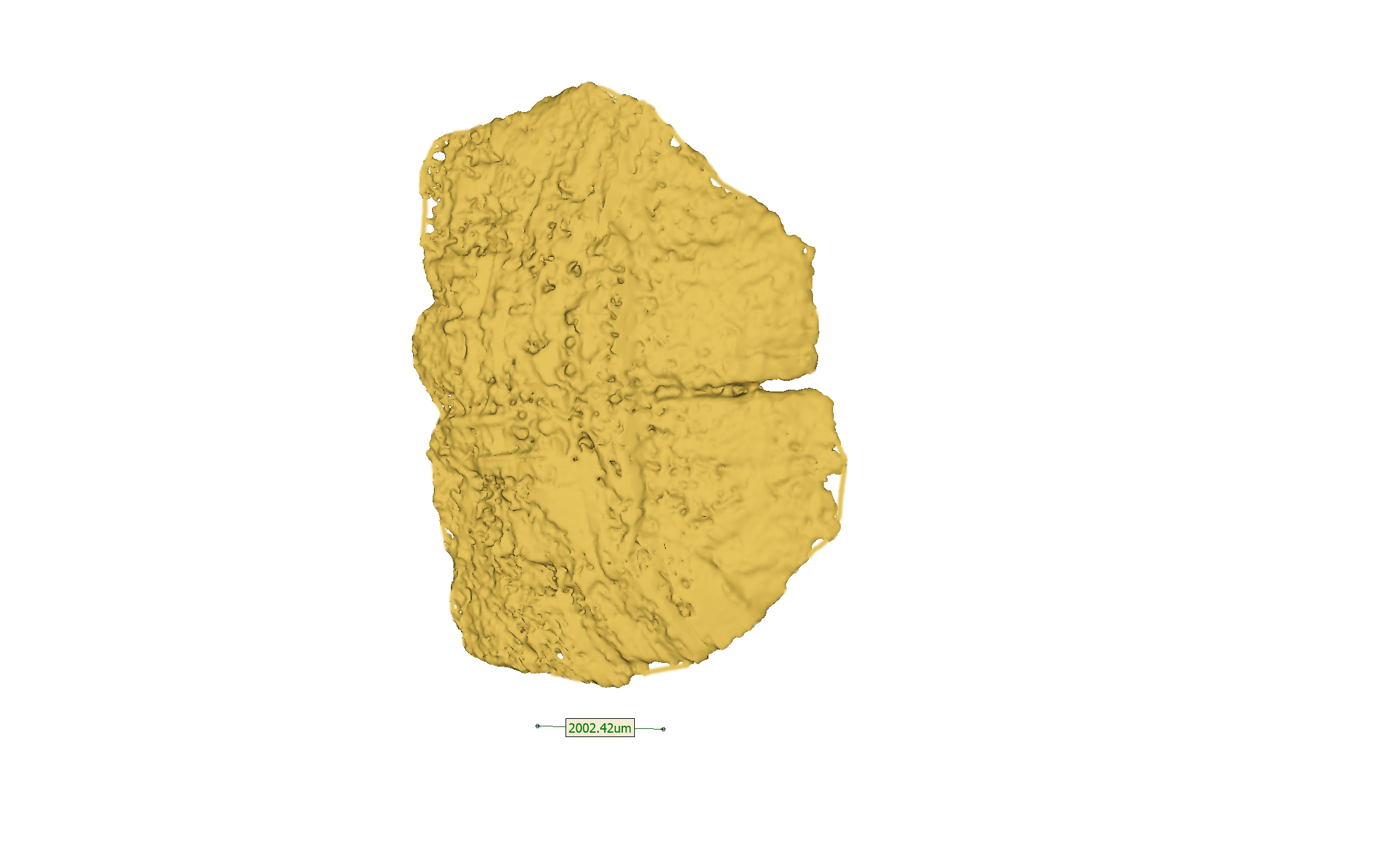

Supplement: Supplementary file 5 — Supplementary Data 2 [file 41467_2023_43557_MOESM5_ESM.zip › Supplementary Data 2/Supplementary Data 2 Raw data of Geometric Morphometric Analyses/12 Morphotypes/Morphotype 2/ll06.jpg]

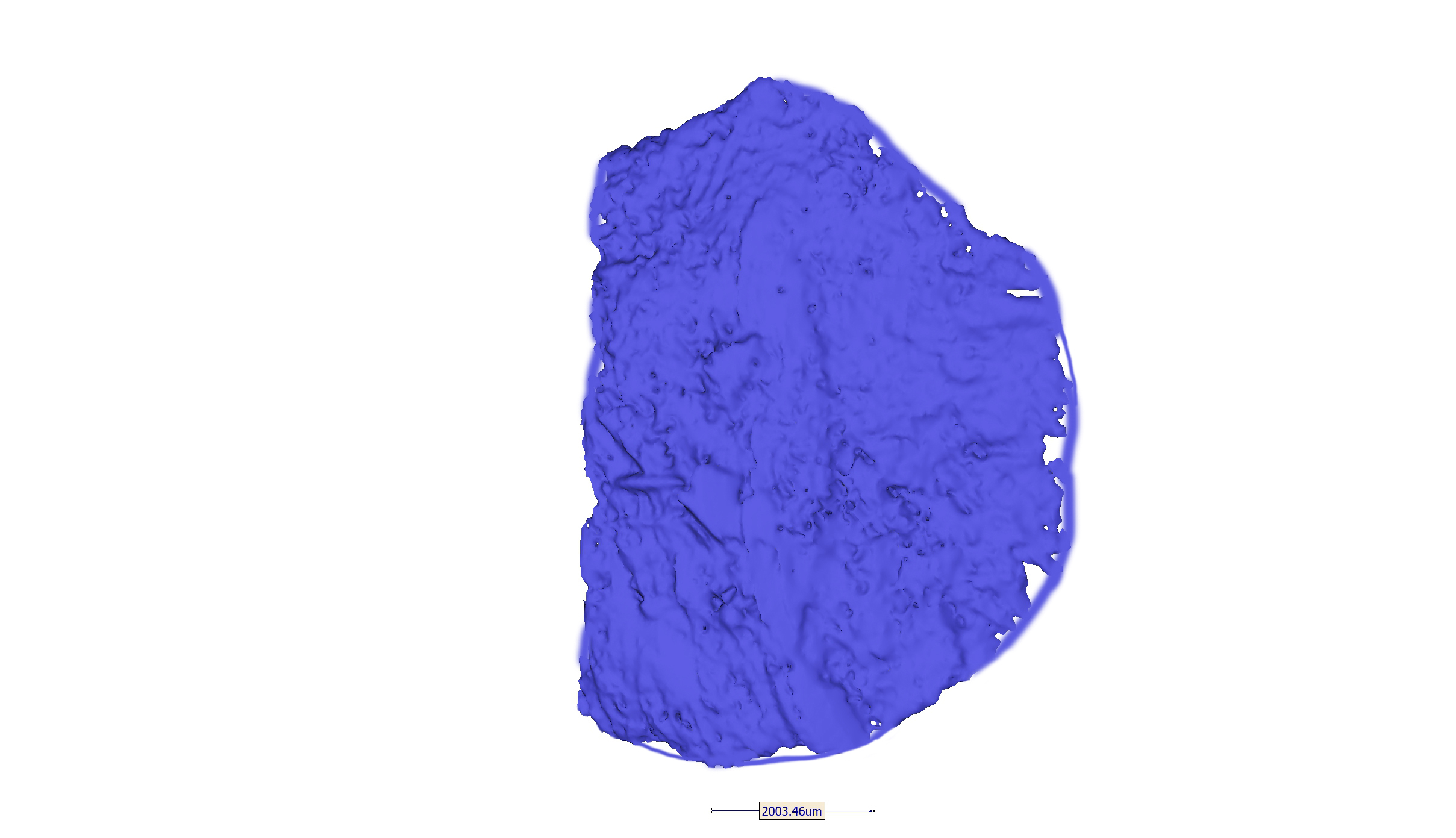

Supplement: Supplementary file 5 — Supplementary Data 2 [file 41467_2023_43557_MOESM5_ESM.zip › Supplementary Data 2/Supplementary Data 2 Raw data of Geometric Morphometric Analyses/12 Morphotypes/Morphotype 3/l1d06.jpg]

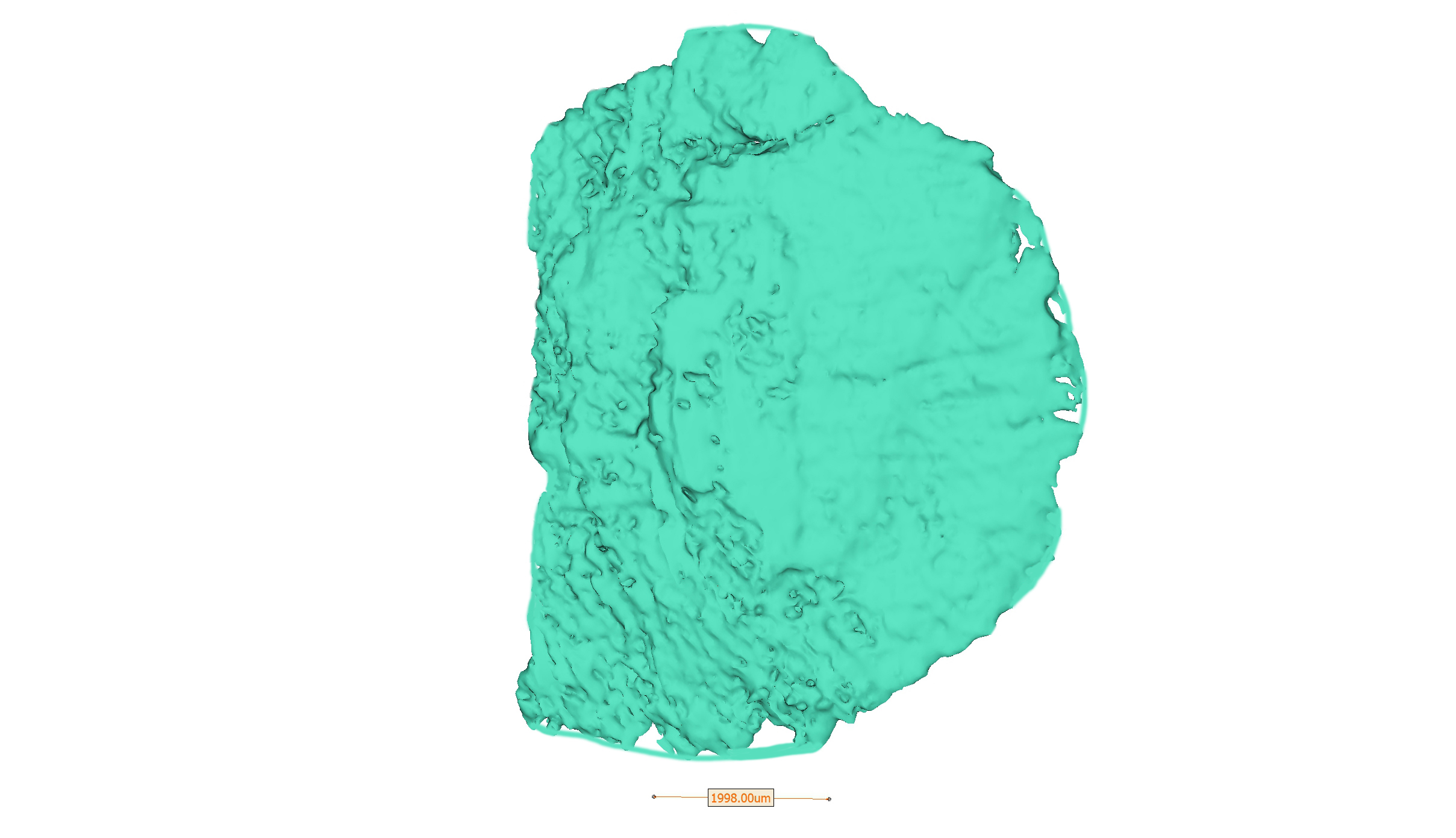

Supplement: Supplementary file 5 — Supplementary Data 2 [file 41467_2023_43557_MOESM5_ESM.zip › Supplementary Data 2/Supplementary Data 2 Raw data of Geometric Morphometric Analyses/12 Morphotypes/Morphotype 3/l1d07.jpg]

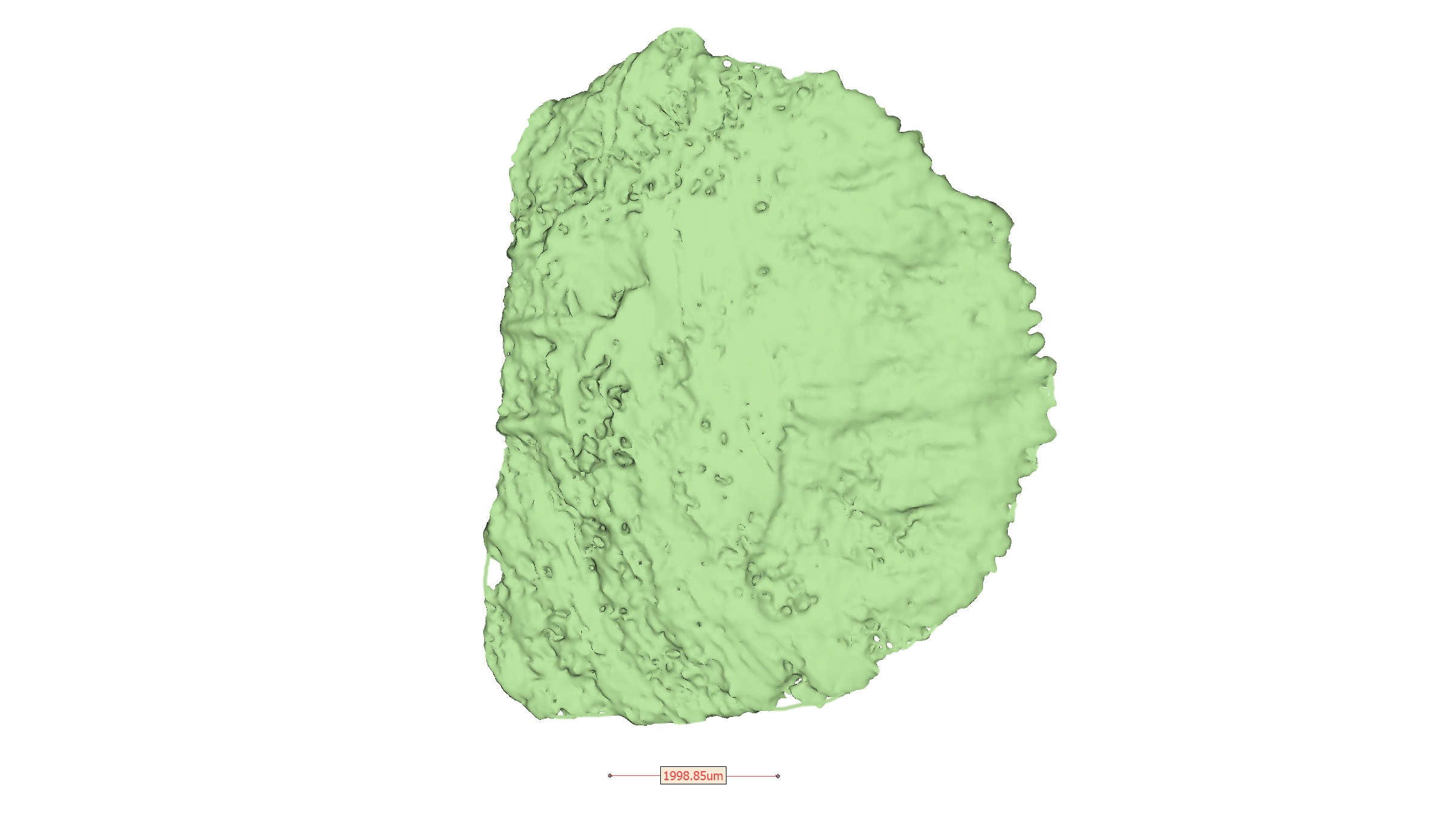

Supplement: Supplementary file 5 — Supplementary Data 2 [file 41467_2023_43557_MOESM5_ESM.zip › Supplementary Data 2/Supplementary Data 2 Raw data of Geometric Morphometric Analyses/12 Morphotypes/Morphotype 3/l1d08.jpg]

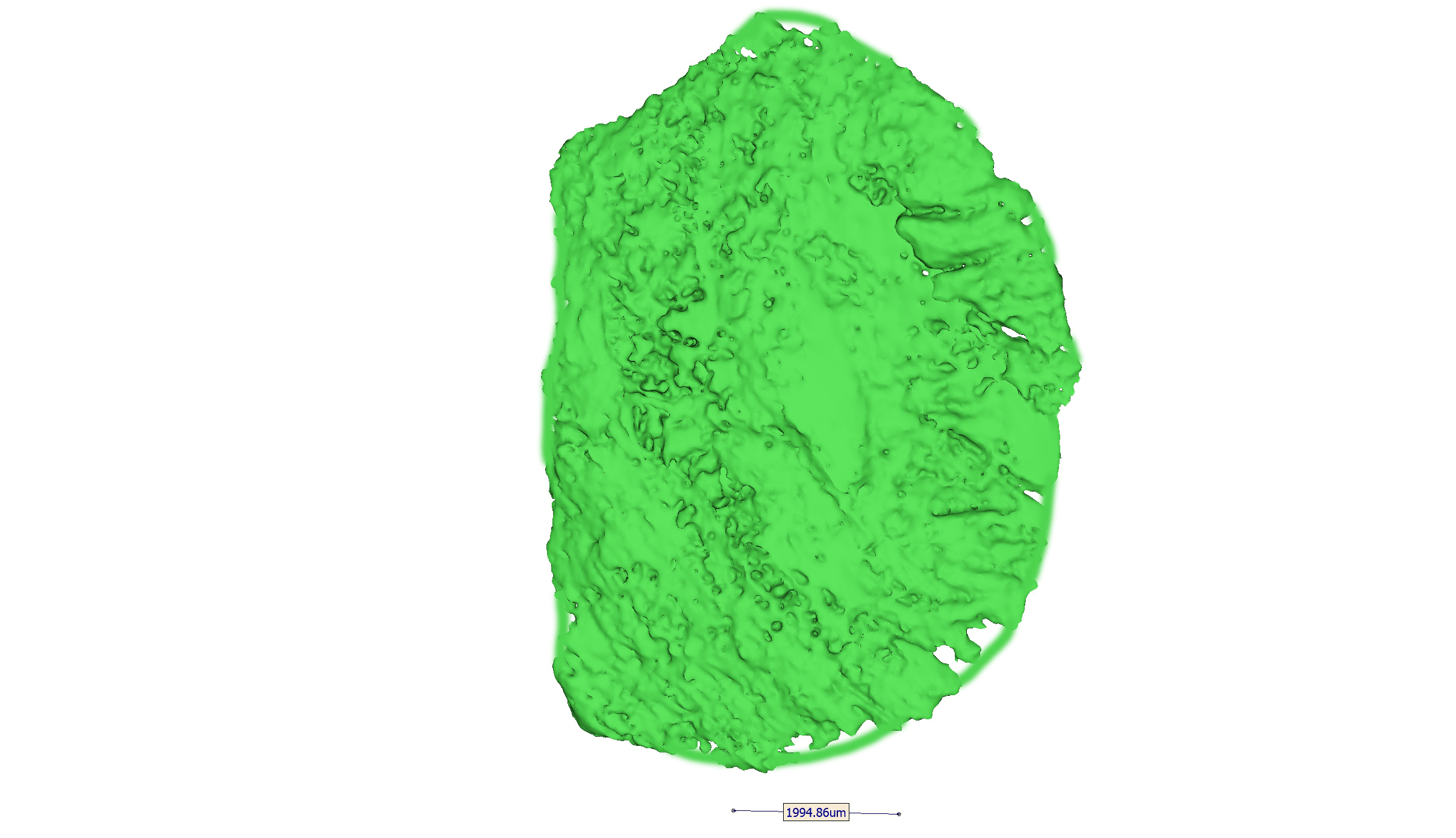

Supplement: Supplementary file 5 — Supplementary Data 2 [file 41467_2023_43557_MOESM5_ESM.zip › Supplementary Data 2/Supplementary Data 2 Raw data of Geometric Morphometric Analyses/12 Morphotypes/Morphotype 3/l1d09.jpg]

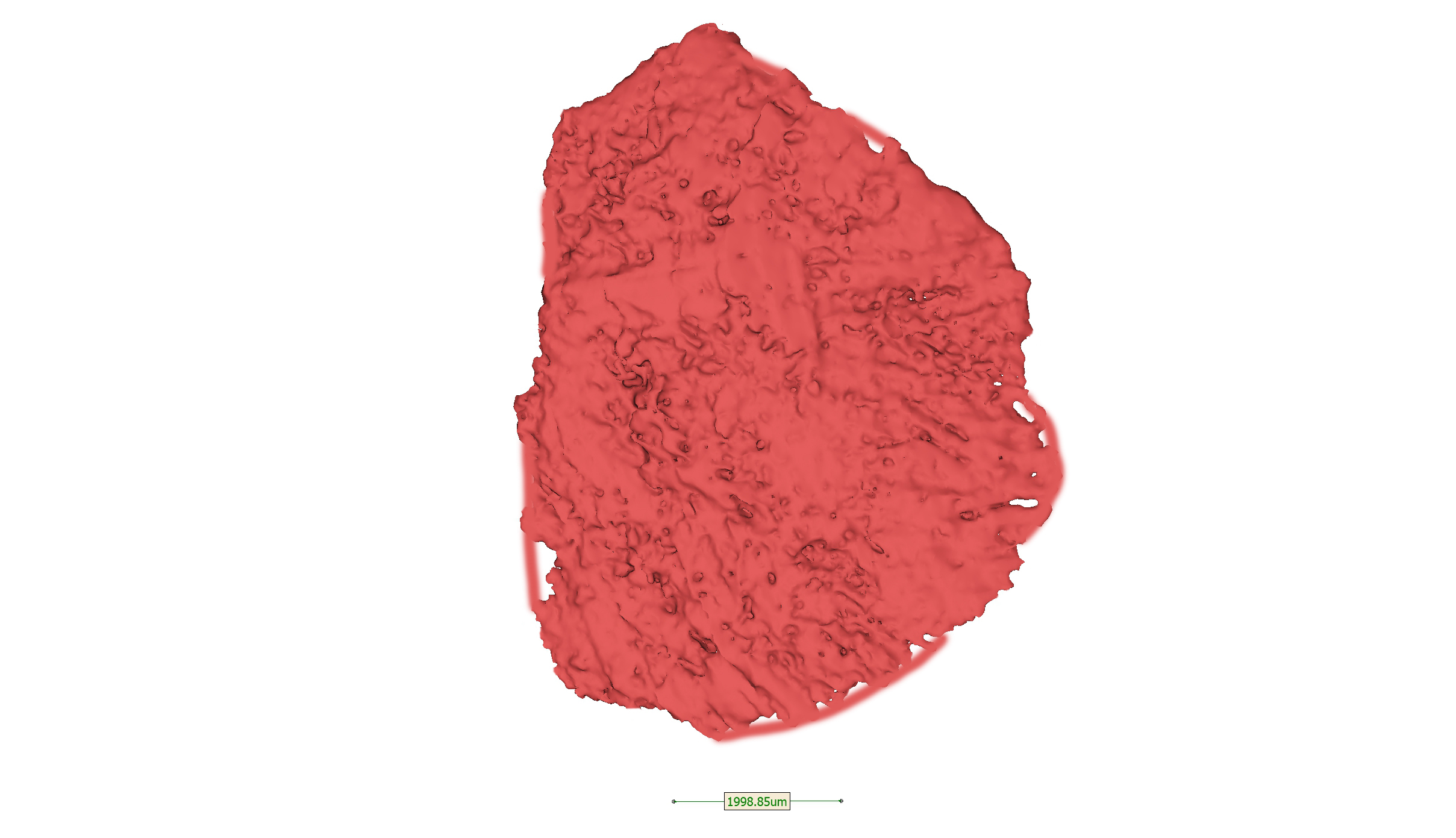

Supplement: Supplementary file 5 — Supplementary Data 2 [file 41467_2023_43557_MOESM5_ESM.zip › Supplementary Data 2/Supplementary Data 2 Raw data of Geometric Morphometric Analyses/12 Morphotypes/Morphotype 3/l1d10.jpg]
