# Supplementary material for: Bony-fish-like scales in a Silurian maxillate placoderm
Source: Nat Commun. 2023 Nov 22;14:7622. doi: 10.1038/s41467-023-43557-9 (PMC10665347; doi:10.1038/s41467-023-43557-9)
Supplement: Supplementary file 10 — Reporting Summary [file 41467_2023_43557_MOESM10_ESM.pdf]

nature portfolio

Corresponding author(s): Min Zhu and You-an Zhu
Last updated by author(s): Nov 2, 2023

Reporting Summary

Nature Portfolio wishes to improve the reproducibility of the work that we publish. This form provides structure for consistency and transparency in reporting. For further information on Nature Portfolio policies, see our Editorial Policies and the Editorial Policy Checklist.

Statistics

For all statistical analyses, confirm that the following items are present in the figure legend, table legend, main text, or Methods section.

n/a	Confirmed	
☐	☒	The exact sample size (*n*) for each experimental group/condition, given as a discrete number and unit of measurement
☒	☐	A statement on whether measurements were taken from distinct samples or whether the same sample was measured repeatedly
☒	☐	The statistical test(s) used AND whether they are one- or two-sided<br>*Only common tests should be described solely by name; describe more complex techniques in the Methods section.*
☒	☐	A description of all covariates tested
☒	☐	A description of any assumptions or corrections, such as tests of normality and adjustment for multiple comparisons
☒	☐	A full description of the statistical parameters including central tendency (e.g. means) or other basic estimates (e.g. regression coefficient) AND variation (e.g. standard deviation) or associated estimates of uncertainty (e.g. confidence intervals)
☒	☐	For null hypothesis testing, the test statistic (e.g. *F*, *t*, *r*) with confidence intervals, effect sizes, degrees of freedom and *P* value noted<br>*Give P values as exact values whenever suitable.*
☒	☐	For Bayesian analysis, information on the choice of priors and Markov chain Monte Carlo settings
☒	☐	For hierarchical and complex designs, identification of the appropriate level for tests and full reporting of outcomes
☒	☐	Estimates of effect sizes (e.g. Cohen's *d*, Pearson's *r*), indicating how they were calculated

Our web collection on statistics for biologists contains articles on many of the points above.

Software and code

Policy information about availability of computer code

Data collection	The specimens were scanned using a GE phoenix v|tome|x m300&180 micro-computed tomography scanner at the Institute of Vertebrate Paleontology and Paleoanthropology (IVPP), Chinese Academy of Sciences. Tomographic data were segmented using the software Mimics 25.0, with images of models rendered in Blender. The images of 237 scales (Supplementary Data 2) for geometric morphometric analysis are printed within Mimics 25.0. Then, each scale outline was scaled before being digitized in a counterclockwise direction from a common starting location, the posteroventral corner, and saved as 50 equidistant semilandmark coordinate points (Supplementary Data 3) with TPSDig2.32 software. The phylogenetic matrix (Supplementary Data 1) used in the analysis was compiled in Mesquite 3.61.
Data analysis	A parsimony analysis was conducted in TNT 1.5. We conducted geometric morphometric analysis using PAST and R (Supplementary Code 1; Supplementary Data 4).

For manuscripts utilizing custom algorithms or software that are central to the research but not yet described in published literature, software must be made available to editors and reviewers. We strongly encourage code deposition in a community repository (e.g. GitHub). See the Nature Portfolio guidelines for submitting code & software for further information.

Data

Policy information about availability of data

All manuscripts must include a data availability statement. This statement should provide the following information, where applicable:

- Accession codes, unique identifiers, or web links for publicly available datasets
- A description of any restrictions on data availability
- For clinical datasets or third party data, please ensure that the statement adheres to our policy

The 3D models used in this study are available in figshare, and available here: https://figshare.com/s/f388c2c162e962aab711. All other files underlying this study, including the phylogenetic dataset and additional notes, are available in the supplementary information and data files.

Research involving human participants, their data, or biological material

Policy information about studies with human participants or human data. See also policy information about sex, gender (identity/presentation), and sexual orientation and race, ethnicity and racism.

Reporting on sex and gender	N/A.
Reporting on race, ethnicity, or other socially relevant groupings	N/A.
Population characteristics	N/A.
Recruitment	N/A.
Ethics oversight	N/A.

Note that full information on the approval of the study protocol must also be provided in the manuscript.

Field-specific reporting

Please select the one below that is the best fit for your research. If you are not sure, read the appropriate sections before making your selection.

☐ Life sciences ☐ Behavioural & social sciences ☒ Ecological, evolutionary & environmental sciences

For a reference copy of the document with all sections, see nature.com/documents/nr-reporting-summary-flat.pdf

Ecological, evolutionary & environmental sciences study design

All studies must disclose on these points even when the disclosure is negative.

Study description	This study described an articulated specimen of a 425 million-years-old fish, Entelognathus primordialis, combining an unusual mosaic of characters typically associated with jawed stem gnathostomes ("placoderms") or crown gnathostomes (osteichthyans and chondrichthyans), and analyzed the early evolution of living jawed vertebrates' dermal skeletons.
Research sample	This study is based on one articulated specimen (IVPP V32322), two isolated plates (V32323.1 and V32323.2) and three isolated scales (V32323.3, V32323.4, V32323.5) of Entelognathus primordialis, and a lower jaw (V32324.1) plus one scale (V32324.2) of Guiyu oneiros, housed at the Institute of Vertebrate Paleontology and Paleoanthropology (IVPP), Chinese Academy of Sciences. They are collected from the muddy limestone from the Kuanti Formation (late Ludlow, Silurian) of Qujing, Yunnan Province, China.
Sampling strategy	We selected the 3D virtual models of left flank scales to do the geometric morphometric analysis. We printed their images in crown view. If a scale is missing or incomplete at one position, a mirror image of the right flank scale (if present) at the same position is used instead. 237 scales were selected (Supplementary Data 2).
Data collection	Sediment samples from a horizon of the Kuanti Formation were collected by the authors during field trips to the site from 2019-2020. Data collection involving observation and recording of specimen features employing light microscopy, micro-CT tomography was performed from 2021 to early 2022. Since then, the specimens were studied by the authors.
Timing and spatial scale	N/A.
Data exclusions	No data is excluded.
Reproducibility	The results of the analyses in this study can be reproduced and verified by re-analyzing the given data set.
Randomization	N/A. Not applicable to the study because it does not have an experimental component.

Blinding	N/A. The study is not subject to selection bias and thus blinding is not applicable to the investigation of this previously undescribed fossil material.

Did the study involve field work? [x] Yes [ ] No

Field work, collection and transport

Field conditions	Annual average temperature is about 14.5°C and annual precipitation is about 590mm.
Location	Dongpo Village (25°26′37″ N, 103°41′39″ E, 1968m), Qujing, Yunnan, China
Access & import/export	Access and collection were carried out with permission of Qujing government, following the national laws.
Disturbance	No disturbances were caused during the study. After collection, the outcrops were restored to the original status.

Reporting for specific materials, systems and methods

We require information from authors about some types of materials, experimental systems and methods used in many studies. Here, indicate whether each material, system or method listed is relevant to your study. If you are not sure if a list item applies to your research, read the appropriate section before selecting a response.

Materials & experimental systems

n/a	Involved in the study
[x]	[ ] Antibodies
[x]	[ ] Eukaryotic cell lines
[ ]	[x] Palaeontology and archaeology
[x]	[ ] Animals and other organisms
[x]	[ ] Clinical data
[x]	[ ] Dual use research of concern
[x]	[ ] Plants

Methods

n/a	Involved in the study
[x]	[ ] ChIP-seq
[x]	[ ] Flow cytometry
[x]	[ ] MRI-based neuroimaging

Palaeontology and Archaeology

Specimen provenance	The specimens, which were collected and processed by the authors, belong to the Institute of Vertebrate Paleontology and Paleoanthropology, Chinese Academy of Sciences.
Specimen deposition	The specimens are housed at the Institute of Vertebrate Paleontology and Paleoanthropology, Chinese Academy of Sciences. They are available on request.
Dating methods	The late Ludlow (Silurian) age of the Kuanti Formation is mainly derived from the conodont zonation (Walliser and Wang, 1989). The new fish specimens were recovered from the muddy limestone of the Kuanti Formation immediately beneath the first appearance point of Ozarkodina crispa (Wang 2001).

[x] Tick this box to confirm that the raw and calibrated dates are available in the paper or in Supplementary Information.

Ethics oversight	Access and collection of the fossil specimens in this study were carried out with permission of Qujing government, following the national laws.

Note that full information on the approval of the study protocol must also be provided in the manuscript.
